# Supplementary material for: Health co-inquiry in migraine: Online participation and stakeholder experiences before and during the COVID-19 pandemic
Source: PLoS One. 2021 Nov 29;16(11):e0260376. doi: 10.1371/journal.pone.0260376 (PMC8629224; doi:10.1371/journal.pone.0260376)
Supplement: S1 File — (PDF) [file pone.0260376.s001.pdf]

Appendices and Supporting Materials to Accompany:

Health Co-Inquiry in Migraine: Online Participation and Stakeholder Experiences Before and During the COVID-19 Pandemic

Camden L. Baucke, MS[1], Lauren S. Seifert, PhD[2], and Kara Kaelber, PhD[3]

[1] Counseling, Eastern Michigan University

[2]Counseling & Psychology Department, Malone University

[3]Counseling & Psychology Department, Malone University

Appendix A: A Bifurcated Method for Evaluating Online Narratives of Migraine Stakeholders

<table>
<tr><td colspan="2">Step 1: Recognize migraine as a chronic condition for inquiry related to the online narratives of stakeholders.</td></tr>
<tr><td colspan="2">Step 2: Borrow the global dictionary of health terms created by Seifert et al.[9] and build a condition-specific dictionary for migraine. These dictionaries provide search terms for the web-crawl.</td></tr>
<tr><td colspan="2">Step 3: Utilize search engines to identify websites with postings by migraine stakeholders.</td></tr>
<tr><td colspan="2">Step 4: Use inclusion and exclusion criteria to inspect websites from Step 3.*</td></tr>
<tr><td colspan="2">Bifurcation: Steps 5 and 6</td></tr>
<tr><td>Inductive, Thematic Analysis

Step 5: Each investigator assesses content and distinguishes themes at 10 randomly selected websites (from the vetted Step 4 list).**</td><td>Webcrawl and Quantitizing

Step 5: Find/develop a robot program to crawl URLs for online narratives of migraine stakeholders.</td></tr>
<tr><td>Step 6: Investigators meet to confer about their reflections. This leads to assembling a list of key themes from narratives online of migraine stakeholders.**</td><td>Step 6: Utilize the robot program to search URLs for all global and condition-specific terms from the dictionaries. And use the program to quantitize frequencies of those terms at the URLs.</td></tr>
<tr><td colspan="2">Step 7: Add rigor to the Bifurcated Method by using data from the web-crawling program to inform the researchers' thematic, inductive analyses. Assess correspondence between key themes from the researchers' qualitative analysis and those from the robot program.</td></tr>
<tr><td colspan="2">Step 8: Another step might be necessary if Step 7 reveals inconsistencies between inductively-derived themes and quantitized themes. Researchers may need to further evaluate URL narratives and the web-crawl data.</td></tr>
</table>

* Criteria are described by Seifert et al.[9]
** Steps 5 and 6 of the inductive analysis occur without consulting web-crawl results.

Appendix B: Condition-Specific Dictionary for Migraine (with // to conserve space)* **

Migraine//Sinus//Otolaryngology//Pathophysiology//Pain//Mechanism//Anatomy//Aura//Differential diagnosis//Diagnosis//Workup//Genesis//Tension//Vascular//Intracranial hypertension//Cluster headache//Cluster//Neuroimage//Neurimaging//Evaluation//Intracranial hypotension//Medication overuse//Cerebrospinal fluid//CSF//Calcium channel//Calcium channel blocker//Lab test//Laboratory test//Pharmacological//Abortive//Serotonin//Serotonin receptor//Serotonin receptor agonist//Agonist//5HT//5-HT //Triptan//Ergot//Derivative//Antihistamine//Opioid//Steroid//Corticosteroid//Isometheptene//Barbiturate//Benzodiaz epine//NSAID//Non-opioid analgesic//Anti-inflammatory//Intranasal//Lidocaine//Intranasal lidocaine//Preemptive//Prophylaxis//Prophylactic//Botox//Botulinum Toxin//Toxin//Anti-hypertensive//Beta-blocker//Blocker//Angiotensin //Angiotensin Converting Enzyme//ACE//Inhibitor//Angiotensin Converting Enzyme Inhibitor//ACE inhibitor//Angiotensin receptor blocker//ARB//Antidepressants//Anticonvulsants//Magnesium//Treatment//Inpatient //Outpatient//Intractable//Type A//Type-A//Allodynia//Rhinogenic//Ptosis//Treatment failure//Injection//Glabellar site//Temporal site//Occipital site//Glabellar//Temporal //Occipital//BTX-A//Nose//nasal//rhinosinusitis//procedure//surgery //surgical//CT scan//CAT scan//Mucosal contact point//Symptom//Genetic//Gene//Subtype//Sub-type//Comorbid //Comorbidity//Pathogenesis//Pharmacologic //Therapy//Pharmacologic therapy//Acute migraine//Chronic migraine//Acute//Chronic//Preventive//Neuromodulation//Neuromodulator //Hormone//Patterns//Accepting//Behavioral therapy//Thought//Attitude//Prevalence//Impact //Risk factor//Trigger//Comorbidities//Prognosis//Stages //Migraine attack//Clinical Evaluation//Diagnostic criteria//History//Headache diary//Structured diagnostic interviews//Headache-related disability//Physical examination//Psychiatric assessment//Neuroimaging//Education//Lifestyle//Sleep management//Diet//Stress//Weight loss//Pharmacotherapy //Acute medications//Acute pharmacotherapy//Ergotamine derivatives//Nonsteroidal anti-inflammatory//Drugs//Combination analgesics//Opioid analgesics//Butalbital//Acetaminophen//Emergency//Preventative medicine//Preventative pharmacotherapy//Tricyclic//Beta-Blockers//Antiepileptic//Anti-epileptic//Antiseizure//Anti-seizure//Nonprescription preventive medications//Menstrually related migraine//Calcitonin //Gene-related//Peptide agent//Ineffective//Medical therapy//Medical therapies//Electrical neuromodulation//Magnetic neuromodulation//Intervention//Surgical treatment //Nonpharmacologic//Progressive muscle relaxation//Biofeedback//Behavioral Intervention //Behavioral Therapy//Biochemistry//Genetic//Hypothalamic regulation//Computer age//Testing//Telemedicine//Screening//Lumbar puncture//Electroencephalography//Recognition //Epidemiology /Acephalgic //Acephalalgic//Amigrainous//Spectrum//Precipitating factor//Secondary headache//Inheritance//Progression//Complications//Complicated migraine//Aura//Visual disturbance//Transient global amnesia//Cadasil//HaNDL syndrome//Migralepsy//Occipital Seizure//Sensory complication//Cerebrovascular disease//Episodic migraine//Special population//Management//Continuity of care//Continuum of care//Consultation//Referral//Gender-based issue//Contraception//Pregnancy//Lactation //Menopause//Circadian feature//Circannual feature//Trigeminal autonomic cephalalgia//Tension-type headache//Clinical presentation//Diagnostic testing//Post-traumatic headache//Traumatic brain injury//Concussion//Post-concussive syndrome//Phenotype//Military setting//ICHD-111 Criteria//Eye//Bedside eye examination//Visual symptom//Eye symptom//Photophobia//Eye pain//Red eye//Visual loss//Abnormal pupil//Double vision//Cranial neuralgia//Sinus headache//Vestibular migraine//Facial pain//Motion sickness//Cervicogenic headache//Neck pain//Neck disorder//Characteristic//Pediatric headache//Mood disorder//Psychiatric illness//Medication//Suicide//Coping style//Psychological approach//Detoxification//Emergency department//Discharge care//Headache clinic//Reimbursement issue//Inpatient treatment//Admission criteria//Advantage//Study design//Outcome measure//Statistical analysis//Frequency//Nausea//Sensitivity to light//Sensitivity to sound//Duration//Pain severity //Population//Correlate//Incidence//Course of disease//Hemiplegic migraine//Aggravating factor//Trigeminocervical complex//Neural substrate of pain//Trigeminovascular nociceptive //Transmission//Pain processing //Sensitization

//Descending inhibition//Peripheral //Central//Muscle-referred pain//Myofascial trigger point//Musculoskeletal pain//Referred pain//Headache models//Descending pain modulation//Placebo effect//Anti-migraine drug//Efficacy//Adverse effect//Preventative therapy //Female hormone//Menstrual migraine//Hormonal contraception//Postpartum period//Prophylactic treatment//Juvenile migraine//Case report//Manual therapies//Manual therapy//Thoracic spine//Cervical spine//Spine intervention//Muscle energy technique//Neuromuscular therapies//Neuromuscular therapy//Central sensitization//Dysfunctional peripheral feature//NMT//Neuromusculoskeletal assessment//Neurodynamic mechanism //Neurodynamic dysfunction//Exercise//Blood flow//Nerve//Cranial Region//Cranium//Cranial nerve//Trigger point//Dry needling//Neck muscle//Head muscle//Shoulder muscle//Vestibular rehabilitation//Motion sensitivity//Visual stimuli//Visual stimulus//Assessment //Randomized controlled trial//Homeopathy//Biobehavioral management//Motivation //Adherence//Biobehavioral therapies//Biobehavioral therapy//Quality of life//Productivity//Hypnosis//Pain management//Pain modulation//Chronic pain//Occipital nerve//Transcranial magnetic stimulation//Sphenopalatine ganglion electrical stimulation//Vagus nerve stimulation//Supra-orbital nerve stimulation//Nerve block//Transcranial direct current stimulation//Chinese medicine//East Asian medicine//Acupuncture//Herbal medicine//Immunoglobulin E//Mast cell//Histamine//Food allergies//Food allergy//Mitochondrial dysfunction//Coenzyme Q10MTHFR//Riboflavin//Homocysteine//Folate//Vitamin B12//Vitamin B6//Glutamate//Dopamine//Trace amine receptor//Melatonin//Nutritional association//Dietary approach//Normobaric oxygen therapy//Hyperbaric oxygen therapy//Oxygen therapy//Adverse effect//Contradiction//Neurobiology//Neural system change//Trigeminovascular pathway//Interictal migraine brain state//Episodic//Iron accumulation//Brain lesion//Clinical utility//Objective measure//Imaging migraine//Midbrain//Diffusion-weighted imaging//Diffusion tensor imaging//Abnormalities//Abnormality//Morphometric change//Voxel-based morphometry//Surface-based structural change//Changing receptor//Metabolite//Magnetoencephalographic//Magnetic resonance angiography//Repetitive transcranial magnetic stimulation//Cortical excitability//Drug effect//Special case//Complementary treatment//Alternative treatment //Integrative treatment//Lifestyle change//Emergency care//Stroke//Cardiovascular disorder//Foramen ovale//Epilepsy//Pain disorder//Obesity//Temporomandibular disorder//Contact point//Optimal management//Positive side effect//Anesthesia dolorosa*

*Terms that were added in May 2019: CGRP//calcitonin//gene-related //Aimovig//Emgality//Ajovy//Nurtec//Ubrelvy//monoclonal//monoclonal antibodies//antibodies

**Terms that were added in May 2021: Sars//Sars-CoV-2//CoVid//CoVid-19//pandemic

Appendix C: Sources for Constructing the Condition-Specific Dictionary

Borsook, D. (2012). *The migraine brain: imaging structure and function*. Oxford University Press.

Diamond, S., Cady, R., Diamond, M. L., Green, M. W., & Martin, V. T. (2015). *Headache and migraine biology and management*. Elsevier/AP.

Dodick, D.W., & Silberstein, S.D. (2016). *Migraine* (Third ed.). New York, NY: Oxford University Press. Fernández-de-las-Peñas, C., Chaitow, L., & Schoenen, J. (2013). *Multidisciplinary management of migraine: pharmacological, manual, and other therapies*. Jones & Bartlett Learning.

Kroner, J. (2015). *Associated Symptoms of Chronic Migraine in Children and Adolescents.* Electronic Thesis or Dissertation). Retrieved from https://etd.ohiolink.edu/

Marcus, D. A. (2016). *Discussing migraine with your patients: a common sense guide for clinicians*. Springer.

Mehle, M. (Ed.). (2017). *Sinus headache, migraine, and the otolaryngologist: A comprehensive* clinical guide. Springer.

Rizzoli, P., Loder, E., & Neporent, L. (2012). *The migraine solution: a complete guide to diagnosis, treatment, and pain management*. St. Martins Press.

Schoenen, J., Dodick, D. W., & Sandor, P. (2011). *Comorbidity in Migraine*. John Wiley & Sons.

Smitherman, T. A. (2016). *Clinicians manual on migraine*. Adis

Appendix D: Key Themes by Stakeholder Group in Online Narratives*

**Migraneur**	**Caregiver/Family**	**Provider**	**Organization or Agency**	**Researcher**	**Employer**	**Key Theme**
Promoting or selling an app or product	Giving advice	Providing up-to-date information and/or resources about diagnosis, treatment, research results, products	Providing up-to-date information and/or resources about diagnosis and/or treatment	Providing information and/or resources about diagnosis, treatment, research results	Discussing Ergonomics, person-environment interactions	**Promoting or giving advice, information, or products**
Providing help/support	Providing help/support	Providing help/support				**Providing help/support (sub-themes: nurturing, caring)**
Seeking help and information (usually from peers)	Seeking help or suggestions (alternative medicine, medical information)				Seeking information about disability accommodations	**Seeking information, advice, suggestions**
Personal stories &Testimonials						**Telling personal stories & giving testimonials (sub-themes: pain, suffering, venting emotions, & telling about things that work/don't)**
Distributing misinformation (perhaps, unintentionally)		Distributing misinformation (perhaps, unintentionally)	Hiding "About Us" information, perhaps to deceive?	Distributing information that seems false and/or obfuscating researcher identities & contact information		**Distributing misinformation or "hiding" About Us information**

* If blank, then the theme was not found in this stakeholder group.

Appendix E: Top Ten* Terms (by Frequency) for Migraine Before COVID-19**

**Migraine Webcrawl Data January-2018***** (of 155,420 words from 64 URLs, without CGRP-related terms)	**Migraine Webcrawl Data from Seifert et al.****** (of 154,646 words from 64 URLs; without CGRP-related terms)	**Migraine Webcrawl Data May-2019***** (of 25,521 words from 10 URLs; searched 15 months after the initial search; CGRP-related terms were added to the condition-specific dictionary)	**Migraine Webcrawl Data August-2019** (of 244,977 words from 91 URLs; searched 18 months after the initial search, with CGRP-related terms)~
Migraine (2311)<br>Ache (764)<br>Ill (616)<br>Pain (498)<br>Work (422)<br>Doctor (220)<br>Suffer (208)<br>Trigger (205)<br>Symptom (195)<br>Treatment (192)	Migraine (2201)<br>Ache (750)<br>Ill (604)<br>Pain (461)<br>Work (404)<br>Doctor (212)<br>Trigger (191)<br>Suffer (189)<br>Symptom (182)<br>Treatment (175)	Migraine (308)<br>Ache (128)<br>Ill (89)<br>Pain (54)<br>Son (44)<br>*Work (41)*<br>*Doctor (41)*<br>*Trigger (41)*<br>Symptom (37)<br>Treatment (33)<br>*Chronic (29)*<br>*Suffer (29)*<br>Gene (28)	Migraine (3400)<br>Ache (1090)<br>Ill (992)<br>Pain (736)<br>Work (675)<br>Drug (455)<br>Doctor (365)<br>Treatment (348)<br>Trigger (314)<br>Symptom (305)

* Top-ten, rank-ordered frequencies with "ties" represented in italics.
** The total number of URLs that is "crawled" depends on whether a URL will allow a robot program to search it. While the original list was 92 URLs, all 92 were not always crawlable.
*** Italics signify a "tie".
**** For comparison, a search of 64 URLs related to migraine, as reported by Seifert et al.[7]
~In the August-2019 webcrawl, with respect to the CGRP terms that were added, "calcitonin" and "gene-related" were mentioned once at each of 3 URLs (frequency count = 3). No other CGRP-related terms were noted.

Appendix F: Top Ten* Terms (by Frequency) for Migraine at the start of COVID-19 and as COVID-19 restrictions were being lifted in the US and in numerous other English-speaking nations (e.g., Canada, UK, Australia)**

**Migraine Webcrawl Data November-2019** (of 44,715 words from 10 URLs; searched 21 months after the initial search, with CGRP-related terms)**	**Migraine Webcrawl Data March-2021** (of 154,646 words from 65* URLs; searched 37 months after the initial search, with CGRP-related terms and with COVID-19 terms)**	**Migraine Webcrawl Data May-2021** (of 244,417 words from 90* URLs; searched 39 months after the initial search, with CGRP-related terms and with COVID-19 terms)***
Migraine (865)<br>Ill (238)<br>Pain (190)<br>Ache (183)<br>Work (158)<br>Evaluation (90)<br>*Trigger (88)*<br>*Doctor (88)*<br>Triptan (70)<br>Medicine (66)<br>Drug (65)	Migraine (2201)<br>Ache (750)<br>Ill (604)<br>Pain (461)<br>Work (404)<br>Doctor (212)<br>Trigger (191)<br>Suffer (189)<br>Symptom (182)<br>Treatment (175)	Migraine (3400)<br>Ache (1090)<br>Ill (992)<br>Pain (736)<br>Work (675)<br>Drug (449)<br>Doctor (365)<br>Treatment (348)<br>Trigger (314)<br>Symptom (305)

*Our total vetted list of URLs from 2018 was 92. Of those 65 were crawlable in March-2021 and 90 were crawlable in May-2021, and 2 sites had been taken down. Over time, sites are available/not available and block/do not block robot crawls. This accounts for some differences in total numbers on days when crawls of all URLs are attempted. In addition, crawls require a large amount of random access memory (RAM) on a server. Thus, the authors' home institution's server "times-out" on some occasions, which may account for a failure to crawl all URLs.

**In the October-2019 and March-2021 webcrawls, in regard to the CGRP terms that were added, "calcitonin" and "gene-related" were mentioned once at 1 URL (frequency count = 1). No other CGRP-related terms were noted.

***In the May-2021 webcrawl, among the CGRP terms that were added, "calcitonin" and "gene-related" were mentioned once at each of 3 URLs (frequency count = 3). No other CGRP-related terms were noted.

Migraine Webcrawl Dataset: Baucke, Seifert, and Kaelber

Health Co-Inquiry in Migraine: Online Participation and Stakeholder Experiences Before and During the COVID-19 Pandemic

This .pdf file includes data from:

January 2018
May 2019
August 2019
November 2019
March 2021
May 2021

In order to review the data:

(1) Use a .pdf file reader to open the file.
(2) Use the Dynamic Zoom (or similar) function in order to expand the view.
(3) Headings indicate when data were collected.
(4) Each row is word frequency data for a specific URL.
(5) The total word count for a given URL is listed in the "Total Number of Words" column.
(6) The frequency of a given word at a specific URL is listed to the RIGHT of the word.

January 2018 Migraine Webcrawl Data

Condition	URL Id	URL	Total number of words	P0a	P0b	P1a	P1b	P2a	P2b
Migraine	707737	https://patient.info/forums/discuss/browse/migraine-1415	3620	Migraine	55	Ache	24	Ill	9
Migraine	858398	http://www.thedailyheadache.com/	2345	Migraine	46	Ache	9	Ill	25
Migraine	858725	http://www.healthcentral.com/migraine/	559	Migraine	23	Ache	1	Ill	2
Migraine	858794	http://migrainepage.forumotion.net/f1-migraines	1254	Migraine	7	Ache	0	Ill	0
Migraine	859735	https://www.facebook.com/MigraineDotCom	978	Migraine	23	Ache	0	Ill	2
Migraine	859952	http://www.migraines.org/help/helpform.htm	340	Migraine	8	Ache	2	Ill	1
Migraine	859953	http://www.topix.com/forum/health/migraine	1695	Migraine	26	Ache	5	Ill	4
Migraine	859954	http://migrainepage.forumotion.net/	201	Migraine	0	Ache	0	Ill	0
Migraine	859957	http://www.healthfulchat.org/migraine-chat-room.html	1071	Migraine	25	Ache	8	Ill	1
Migraine	859969	http://helpforheadaches.com/forum.htm	902	Migraine	15	Ache	12	Ill	3
Migraine	860425	http://www.healthcentral.com/migraine/c/123/177401/experience-spring-migraine	1266	Migraine	41	Ache	4	Ill	3
Migraine	860462	http://www.helpforheadaches.com/	389	Migraine	13	Ache	7	Ill	4
Migraine	866666	https://www.patientslikeme.com/conditions/135-migraine	3638	Migraine	164	Ache	15	Ill	1
Migraine	866670	http://www.medhelp.org/forums/Migraines--Headaches/show/123	1401	Migraine	14	Ache	33	Ill	1
Migraine	866775	http://migrainetalk.forumotion.co.uk/	921	Migraine	15	Ache	0	Ill	0
Migraine	867244	https://migrainetalkblog.wordpress.com/	1178	Migraine	57	Ache	9	Ill	3
Migraine	867708	https://www.curezone.org/forums/am.asp?i=60104	1275	Migraine	23	Ache	6	Ill	2
Migraine	869442	https://www.curezone.org/forums/f3.asp?f=68	5175	Migraine	25	Ache	39	Ill	13
Migraine	875100	http://www.thedailymigraine.com/forum-moot/	126	Migraine	4	Ache	0	Ill	0
Migraine	875101	http://forums.phoenixrising.me/index.php?threads/questions-about-migraine-aura.34477/	4384	Migraine	64	Ache	11	Ill	19
Migraine	875102	https://forum.bulletproof.com/discussion/1406/bulletproof-migraine-specific-diet	1770	Migraine	21	Ache	11	Ill	5
Migraine	875103	http://www.migrainebuddy.com/understanding-migraines-the-gender-divide/	1928	Migraine	106	Ache	2	Ill	5
Migraine	875104	http://www.migrainebuddy.com/migraine-stories/	427	Migraine	18	Ache	0	Ill	0
Migraine	875105	https://ehealthforum.com/health/headache-and-migraines-question-t504544.html	738	Migraine	1	Ache	8	Ill	1
Migraine	875106	http://www.migraine.org.uk/	322	Migraine	7	Ache	1	Ill	0
Migraine	875107	http://www.migraine.org.uk/get-advice/	440	Migraine	9	Ache	0	Ill	0
Migraine	875108	https://www.physicsforums.com/threads/the-four-stages-of-migraine.305792/	2068	Migraine	64	Ache	24	Ill	2
Migraine	875111	https://www.healingwell.com/community/?f=31	684	Migraine	6	Ache	5	Ill	1
Migraine	892508	https://migraineresearchfoundation.org/resources/resources-and-links/	1690	Migraine	37	Ache	34	Ill	11
Migraine	900794	https://migraine.newlifeoutlook.com/community/discussion/17/complex-migraine	680	Migraine	13	Ache	8	Ill	1
Migraine	900795	https://www.dinet.org/forums/topic/27868-anybody-else-have-an-intractable-migraine/	2056	Migraine	21	Ache	11	Ill	6
Migraine	900796	https://migraine.newlifeoutlook.com/community/	511	Migraine	7	Ache	1	Ill	0
Migraine	900797	https://www.medschat.com/Discuss/Migraine-Tension-Headache/	3700	Migraine	18	Ache	14	Ill	22
Migraine	900798	https://exchanges.webmd.com/migraines-and-headaches-exchange	1228	Migraine	7	Ache	2	Ill	3
Migraine	900799	https://hubpages.com/health/forum/27466/any-ocular-migraine-sufferers-here	2218	Migraine	19	Ache	5	Ill	6
Migraine	900800	http://www.experienceproject.com/groups/Have-Vestibular-Migraine/117436	3092	Migraine	24	Ache	6	Ill	14
Migraine	900801	http://www.migrainepage.com/	28	Migraine	0	Ache	0	Ill	0
Migraine	900802	https://www.chronofhorse.com/forum/archive/index.php/t-318339.html	5475	Migraine	38	Ache	12	Ill	28
Migraine	900803	http://www.mvertigo.org/	360	Migraine	2	Ache	1	Ill	1
Migraine	900804	https://ergoweb.com/forums/topic/treatment-of-light-induced-migraine-headaches-in-the-office/	1237	Migraine	11	Ache	6	Ill	4
Migraine	900805	http://curetogether.com/Migraine/discuss/	34761	Migraine	487	Ache	137	Ill	204
Migraine	900806	http://www.experienceproject.com/groups/Get-Migraine-Headaches/102	2720	Migraine	25	Ache	15	Ill	10
Migraine	900807	https://www.tapatalk.com/groups/headachepainfreeforums/topic/766/Silent-Migraine	1496	Migraine	18	Ache	4	Ill	7
Migraine	900808	https://coloradopain.co/patient-forms/	378	Migraine	0	Ache	0	Ill	2
Migraine	900809	https://forum.grasscity.com/threads/migraines.453628/	2427	Migraine	46	Ache	14	Ill	9
Migraine	900810	http://www.migrainepage.com/help.html	287	Migraine	10	Ache	8	Ill	0
Migraine	900811	http://www.tmswiki.org/forum/threads/my-migraines.2072/	2514	Migraine	30	Ache	9	Ill	8
Migraine	900812	http://www.thedailymigraine.com/support-groups/	295	Migraine	11	Ache	0	Ill	2
Migraine	900813	https://community.qvc.com/t5/Wellness/MIGRAINE-sufferers/td-p/1182015	2964	Migraine	34	Ache	3	Ill	6
Migraine	900814	http://www.fightingfatigue.org/headache-migraine-websites-forums/	415	Migraine	5	Ache	4	Ill	0
Migraine	900815	https://www.tapatalk.com/groups/vets/topic/67841/Migraines?page=2	8698	Migraine	134	Ache	71	Ill	29
Migraine	900816	https://www.inspire.com/groups/headaches-and-migraines/discussion/migraines-each-day-unbareable/	5325	Migraine	86	Ache	40	Ill	16
Migraine	900817	http://www.usmle-forums.com/usmle-step-1-forum/217482-migraine.html	691	Migraine	4	Ache	1	Ill	0
Migraine	900818	https://forum.dontpayfull.com/threads/migraine-medicine.25791/	3332	Migraine	36	Ache	29	Ill	18
Migraine	900819	http://www.themigrainerevolution.com/yahoo-group/	501	Migraine	16	Ache	0	Ill	2
Migraine	900820	http://www.fibromyalgiaforums.org/forum/general-discussions-about-fibromyalgia/26893-fibro-migraines.html	2552	Migraine	27	Ache	20	Ill	4
Migraine	900821	http://forums.prohealth.com/forums/index.php?threads/migraine-from-hell-called-status-migrainosus.110648/	1389	Migraine	17	Ache	4	Ill	4
Migraine	900822	http://forums.whirlpool.net.au/archive/1924289	8824	Migraine	88	Ache	38	Ill	42
Migraine	900823	http://www.nomorepanic.co.uk/showthread.php?t=106093	1914	Migraine	16	Ache	0	Ill	5
Migraine	900824	http://www.relieve-migraine-headache.com/basilar-migraines.html	774	Migraine	20	Ache	5	Ill	2
Migraine	900825	https://cassiopaea.org/forum/?topic=17971.0	3636	Migraine	71	Ache	4	Ill	25
Migraine	900826	https://www.fodors.com/community/air-travel/long-flight-migraine.cfm	2745	Migraine	28	Ache	16	Ill	7
Migraine	900827	https://www.infantrisk.com/forum/forum/medications-and-breastfeeding-mothers/other-uncategorized-medications/152-migraine-medications	2082	Migraine	15	Ache	3	Ill	7
Migraine	900842	https://www.cumom.ie/forums/topic/bad-bad-migraine/	1330	Migraine	10	Ache	3	Ill	4
			155420		2311		764		616

P69a	P69b	P70a	P70b	P71a	P71b	P72a	P72b	P73a	P73b	P74a	P74b	P75a	P75b	P76a	P76b	P77a	P77b	P78a	P78b	P79a	P79b	P80a	P80b	P81a	P81b	P82a	P82b	P83a	P83b	P84a	P84b	P85a	P85b	P86a	P86b	P87a	P87b	P88a	P88b	P89a	P89b	P90a	P90b	P91a	P91b
Ergot	7	Mother	0	Blocker	0	Child	0	travel	0	Cluster	1	nasal	0	pray	0	Management	0	husband	0	Prophylactic	0	Daughter	0	Acetaminophen	0	Parent	0	Present	0	Disability	0	Menopause	1	employ	0	Support group	0	Hemiplegic migraine	2	Neck pain	0	Brother	0	Herbal medicine	0
Ergot	0	Mother	0	Blocker	0	Child	0	travel	2	Cluster	0	nasal	0	pray	0	Management	0	husband	0	Prophylactic	0	Daughter	0	Acetaminophen	0	Parent	0	Present	1	Disability	2	Menopause	0	employ	0	Support group	0	Hemiplegic migraine	0	Neck pain	0	Brother	0	Herbal medicine	0
Ergot	0	Mother	0	Blocker	0	Child	0	travel	1	Cluster	0	nasal	0	pray	0	Management	1	husband	0	Prophylactic	0	Daughter	0	Acetaminophen	0	Parent	0	Present	1	Disability	0	Menopause	0	employ	0	Support group	0	Hemiplegic migraine	3	Neck pain	0	Brother	0	Herbal medicine	0
Ergot	0	Mother	0	Blocker	0	Child	0	travel	0	Cluster	0	nasal	0	pray	0	Management	0	husband	0	Prophylactic	0	Daughter	0	Acetaminophen	0	Parent	0	Present	0	Disability	0	Menopause	0	employ	0	Support group	0	Hemiplegic migraine	0	Neck pain	0	Brother	0	Herbal medicine	0
Ergot	0	Mother	0	Blocker	0	Child	1	travel	0	Cluster	0	nasal	0	pray	0	Management	0	husband	0	Prophylactic	0	Daughter	0	Acetaminophen	0	Parent	2	Present	0	Disability	0	Menopause	0	employ	0	Support group	0	Hemiplegic migraine	0	Neck pain	0	Brother	1	Herbal medicine	0
Ergot	0	Mother	0	Blocker	0	Child	0	travel	0	Cluster	0	nasal	0	pray	0	Management	0	husband	0	Prophylactic	0	Daughter	0	Acetaminophen	0	Parent	0	Present	0	Disability	0	Menopause	0	employ	0	Support group	0	Hemiplegic migraine	0	Neck pain	0	Brother	0	Herbal medicine	0
Ergot	0	Mother	0	Blocker	0	Child	1	travel	0	Cluster	0	nasal	1	pray	1	Management	0	husband	0	Prophylactic	0	Daughter	0	Acetaminophen	0	Parent	0	Present	0	Disability	0	Menopause	0	employ	0	Support group	0	Hemiplegic migraine	0	Neck pain	0	Brother	0	Herbal medicine	0
Ergot	0	Mother	0	Blocker	0	Child	0	travel	0	Cluster	0	nasal	0	pray	0	Management	0	husband	0	Prophylactic	0	Daughter	0	Acetaminophen	0	Parent	0	Present	0	Disability	0	Menopause	0	employ	0	Support group	0	Hemiplegic migraine	0	Neck pain	0	Brother	0	Herbal medicine	0
Ergot	0	Mother	0	Blocker	0	Child	0	travel	0	Cluster	0	nasal	0	pray	0	Management	0	husband	0	Prophylactic	0	Daughter	0	Acetaminophen	0	Parent	0	Present	0	Disability	2	Menopause	0	employ	0	Support group	1	Hemiplegic migraine	0	Neck pain	0	Brother	0	Herbal medicine	0
Ergot	0	Mother	0	Blocker	0	Child	0	travel	0	Cluster	0	nasal	0	pray	0	Management	0	husband	0	Prophylactic	0	Daughter	0	Acetaminophen	0	Parent	0	Present	0	Disability	0	Menopause	0	employ	0	Support group	0	Hemiplegic migraine	0	Neck pain	0	Brother	0	Herbal medicine	0
Ergot	0	Mother	0	Blocker	0	Child	0	travel	1	Cluster	0	nasal	0	pray	0	Management	1	husband	0	Prophylactic	0	Daughter	0	Acetaminophen	0	Parent	0	Present	0	Disability	0	Menopause	0	employ	0	Support group	0	Hemiplegic migraine	0	Neck pain	0	Brother	0	Herbal medicine	0
Ergot	0	Mother	0	Blocker	0	Child	0	travel	0	Cluster	1	nasal	0	pray	0	Management	1	husband	0	Prophylactic	0	Daughter	0	Acetaminophen	0	Parent	0	Present	1	Disability	0	Menopause	0	employ	0	Support group	0	Hemiplegic migraine	0	Neck pain	0	Brother	0	Herbal medicine	0
Ergot	0	Mother	0	Blocker	0	Child	0	travel	0	Cluster	0	nasal	0	pray	0	Management	0	husband	0	Prophylactic	0	Daughter	0	Acetaminophen	9	Parent	0	Present	0	Disability	0	Menopause	0	employ	0	Support group	0	Hemiplegic migraine	0	Neck pain	0	Brother	0	Herbal medicine	0
Ergot	0	Mother	0	Blocker	2	Child	1	travel	0	Cluster	1	nasal	0	pray	0	Management	0	husband	0	Prophylactic	0	Daughter	2	Acetaminophen	0	Parent	0	Present	1	Disability	1	Menopause	0	employ	0	Support group	0	Hemiplegic migraine	0	Neck pain	1	Brother	0	Herbal medicine	0
Ergot	0	Mother	0	Blocker	0	Child	0	travel	0	Cluster	0	nasal	0	pray	0	Management	0	husband	0	Prophylactic	0	Daughter	0	Acetaminophen	0	Parent	0	Present	0	Disability	0	Menopause	0	employ	0	Support group	0	Hemiplegic migraine	0	Neck pain	0	Brother	0	Herbal medicine	0
Ergot	0	Mother	0	Blocker	0	Child	0	travel	0	Cluster	0	nasal	0	pray	0	Management	2	husband	0	Prophylactic	0	Daughter	0	Acetaminophen	0	Parent	0	Present	0	Disability	0	Menopause	0	employ	0	Support group	0	Hemiplegic migraine	0	Neck pain	0	Brother	0	Herbal medicine	0
Ergot	0	Mother	0	Blocker	0	Child	0	travel	0	Cluster	0	nasal	0	pray	0	Management	0	husband	0	Prophylactic	0	Daughter	0	Acetaminophen	0	Parent	0	Present	1	Disability	0	Menopause	0	employ	0	Support group	0	Hemiplegic migraine	0	Neck pain	0	Brother	0	Herbal medicine	0
Ergot	0	Mother	0	Blocker	0	Child	1	travel	1	Cluster	2	nasal	0	pray	0	Management	0	husband	0	Prophylactic	0	Daughter	0	Acetaminophen	0	Parent	0	Present	0	Disability	0	Menopause	0	employ	0	Support group	0	Hemiplegic migraine	0	Neck pain	0	Brother	0	Herbal medicine	0
Ergot	0	Mother	0	Blocker	0	Child	0	travel	0	Cluster	0	nasal	0	pray	0	Management	0	husband	0	Prophylactic	0	Daughter	0	Acetaminophen	0	Parent	0	Present	0	Disability	0	Menopause	0	employ	0	Support group	0	Hemiplegic migraine	0	Neck pain	0	Brother	0	Herbal medicine	0
Ergot	0	Mother	3	Blocker	0	Child	1	travel	0	Cluster	0	nasal	0	pray	0	Management	0	husband	0	Prophylactic	1	Daughter	0	Acetaminophen	0	Parent	3	Present	1	Disability	0	Menopause	4	employ	0	Support group	0	Hemiplegic migraine	0	Neck pain	0	Brother	1	Herbal medicine	0
Ergot	0	Mother	0	Blocker	0	Child	0	travel	0	Cluster	0	nasal	0	pray	0	Management	0	husband	0	Prophylactic	0	Daughter	0	Acetaminophen	0	Parent	0	Present	1	Disability	0	Menopause	0	employ	0	Support group	0	Hemiplegic migraine	0	Neck pain	0	Brother	4	Herbal medicine	0
Ergot	0	Mother	0	Blocker	0	Child	2	travel	0	Cluster	0	nasal	0	pray	0	Management	0	husband	0	Prophylactic	0	Daughter	0	Acetaminophen	0	Parent	0	Present	0	Disability	0	Menopause	0	employ	0	Support group	2	Hemiplegic migraine	0	Neck pain	0	Brother	0	Herbal medicine	0
Ergot	0	Mother	0	Blocker	0	Child	0	travel	0	Cluster	0	nasal	0	pray	0	Management	0	husband	0	Prophylactic	0	Daughter	0	Acetaminophen	0	Parent	0	Present	0	Disability	0	Menopause	0	employ	0	Support group	0	Hemiplegic migraine	0	Neck pain	0	Brother	0	Herbal medicine	0
Ergot	0	Mother	0	Blocker	0	Child	0	travel	0	Cluster	0	nasal	0	pray	0	Management	0	husband	0	Prophylactic	0	Daughter	0	Acetaminophen	0	Parent	0	Present	0	Disability	0	Menopause	0	employ	0	Support group	0	Hemiplegic migraine	0	Neck pain	0	Brother	0	Herbal medicine	0
Ergot	0	Mother	0	Blocker	0	Child	0	travel	0	Cluster	0	nasal	0	pray	0	Management	0	husband	0	Prophylactic	0	Daughter	0	Acetaminophen	0	Parent	0	Present	0	Disability	0	Menopause	0	employ	0	Support group	0	Hemiplegic migraine	0	Neck pain	0	Brother	0	Herbal medicine	0
Ergot	0	Mother	0	Blocker	0	Child	0	travel	0	Cluster	0	nasal	0	pray	0	Management	0	husband	0	Prophylactic	0	Daughter	0	Acetaminophen	0	Parent	0	Present	0	Disability	0	Menopause	0	employ	0	Support group	0	Hemiplegic migraine	0	Neck pain	0	Brother	0	Herbal medicine	0
Ergot	0	Mother	0	Blocker	0	Child	0	travel	1	Cluster	0	nasal	4	pray	0	Management	0	husband	0	Prophylactic	0	Daughter	0	Acetaminophen	0	Parent	2	Present	1	Disability	0	Menopause	0	employ	0	Support group	0	Hemiplegic migraine	0	Neck pain	0	Brother	0	Herbal medicine	0
Ergot	0	Mother	0	Blocker	0	Child	0	travel	0	Cluster	1	nasal	0	pray	0	Management	0	husband	0	Prophylactic	0	Daughter	0	Acetaminophen	0	Parent	0	Present	0	Disability	0	Menopause	0	employ	0	Support group	0	Hemiplegic migraine	0	Neck pain	0	Brother	0	Herbal medicine	0
Ergot	0	Mother	1	Blocker	0	Child	3	travel	0	Cluster	1	nasal	0	pray	0	Management	2	husband	0	Prophylactic	0	Daughter	0	Acetaminophen	1	Parent	1	Present	0	Disability	0	Menopause	0	employ	0	Support group	0	Hemiplegic migraine	0	Neck pain	0	Brother	0	Herbal medicine	0
Ergot	0	Mother	1	Blocker	0	Child	0	travel	0	Cluster	0	nasal	0	pray	1	Management	0	husband	1	Prophylactic	0	Daughter	0	Acetaminophen	0	Parent	1	Present	0	Disability	0	Menopause	0	employ	0	Support group	0	Hemiplegic migraine	0	Neck pain	0	Brother	0	Herbal medicine	0
Ergot	0	Mother	0	Blocker	3	Child	0	travel	0	Cluster	1	nasal	1	pray	0	Management	0	husband	0	Prophylactic	0	Daughter	3	Acetaminophen	0	Parent	0	Present	1	Disability	0	Menopause	0	employ	0	Support group	0	Hemiplegic migraine	0	Neck pain	0	Brother	0	Herbal medicine	0
Ergot	0	Mother	0	Blocker	0	Child	0	travel	0	Cluster	0	nasal	0	pray	0	Management	0	husband	0	Prophylactic	0	Daughter	0	Acetaminophen	0	Parent	0	Present	0	Disability	0	Menopause	0	employ	1	Support group	0	Hemiplegic migraine	0	Neck pain	0	Brother	0	Herbal medicine	0
Ergot	0	Mother	0	Blocker	0	Child	0	travel	0	Cluster	0	nasal	0	pray	0	Management	0	husband	1	Prophylactic	0	Daughter	0	Acetaminophen	2	Parent	0	Present	0	Disability	0	Menopause	0	employ	0	Support group	0	Hemiplegic migraine	0	Neck pain	0	Brother	0	Herbal medicine	0
Ergot	0	Mother	0	Blocker	0	Child	1	travel	0	Cluster	0	nasal	0	pray	0	Management	0	husband	0	Prophylactic	0	Daughter	0	Acetaminophen	0	Parent	0	Present	1	Disability	0	Menopause	0	employ	0	Support group	2	Hemiplegic migraine	0	Neck pain	0	Brother	0	Herbal medicine	0
Ergot	0	Mother	1	Blocker	0	Child	0	travel	0	Cluster	0	nasal	0	pray	0	Management	0	husband	2	Prophylactic	0	Daughter	4	Acetaminophen	0	Parent	2	Present	0	Disability	0	Menopause	0	employ	0	Support group	0	Hemiplegic migraine	0	Neck pain	0	Brother	0	Herbal medicine	0
Ergot	0	Mother	0	Blocker	0	Child	0	travel	1	Cluster	0	nasal	0	pray	1	Management	0	husband	0	Prophylactic	0	Daughter	0	Acetaminophen	0	Parent	1	Present	0	Disability	0	Menopause	0	employ	0	Support group	1	Hemiplegic migraine	0	Neck pain	0	Brother	0	Herbal medicine	0
Ergot	0	Mother	0	Blocker	0	Child	0	travel	0	Cluster	0	nasal	0	pray	0	Management	0	husband	0	Prophylactic	0	Daughter	0	Acetaminophen	0	Parent	0	Present	0	Disability	0	Menopause	0	employ	0	Support group	0	Hemiplegic migraine	0	Neck pain	0	Brother	0	Herbal medicine	0
Ergot	0	Mother	1	Blocker	0	Child	1	travel	0	Cluster	0	nasal	0	pray	0	Management	0	husband	1	Prophylactic	0	Daughter	0	Acetaminophen	0	Parent	0	Present	1	Disability	0	Menopause	3	employ	0	Support group	0	Hemiplegic migraine	0	Neck pain	0	Brother	1	Herbal medicine	0
Ergot	0	Mother	0	Blocker	0	Child	0	travel	0	Cluster	0	nasal	0	pray	0	Management	0	husband	0	Prophylactic	0	Daughter	0	Acetaminophen	0	Parent	0	Present	1	Disability	0	Menopause	0	employ	0	Support group	0	Hemiplegic migraine	0	Neck pain	0	Brother	0	Herbal medicine	0
Ergot	0	Mother	0	Blocker	0	Child	0	travel	0	Cluster	0	nasal	0	pray	0	Management	1	husband	0	Prophylactic	0	Daughter	0	Acetaminophen	0	Parent	1	Present	0	Disability	0	Menopause	0	employ	4	Support group	0	Hemiplegic migraine	0	Neck pain	0	Brother	0	Herbal medicine	0
Ergot	9	Mother	5	Blocker	3	Child	2	travel	2	Cluster	0	nasal	10	pray	11	Management	2	husband	9	Prophylactic	4	Daughter	2	Acetaminophen	4	Parent	2	Present	3	Disability	0	Menopause	5	employ	3	Support group	0	Hemiplegic migraine	1	Neck pain	14	Brother	3	Herbal medicine	15
Ergot	0	Mother	2	Blocker	0	Child	2	travel	0	Cluster	0	nasal	0	pray	0	Management	0	husband	0	Prophylactic	0	Daughter	0	Acetaminophen	0	Parent	0	Present	0	Disability	1	Menopause	0	employ	0	Support group	1	Hemiplegic migraine	0	Neck pain	0	Brother	0	Herbal medicine	0
Ergot	0	Mother	0	Blocker	1	Child	0	travel	1	Cluster	0	nasal	0	pray	0	Management	0	husband	0	Prophylactic	0	Daughter	0	Acetaminophen	0	Parent	0	Present	0	Disability	0	Menopause	0	employ	0	Support group	0	Hemiplegic migraine	0	Neck pain	0	Brother	3	Herbal medicine	0
Ergot	0	Mother	0	Blocker	0	Child	0	travel	0	Cluster	0	nasal	0	pray	0	Management	1	husband	0	Prophylactic	0	Daughter	0	Acetaminophen	0	Parent	0	Present	0	Disability	0	Menopause	0	employ	0	Support group	0	Hemiplegic migraine	0	Neck pain	0	Brother	0	Herbal medicine	0
Ergot	0	Mother	0	Blocker	0	Child	0	travel	0	Cluster	4	nasal	0	pray	0	Management	0	husband	0	Prophylactic	0	Daughter	0	Acetaminophen	0	Parent	3	Present	0	Disability	0	Menopause	0	employ	0	Support group	0	Hemiplegic migraine	0	Neck pain	0	Brother	0	Herbal medicine	0
Ergot	0	Mother	0	Blocker	0	Child	0	travel	0	Cluster	1	nasal	0	pray	0	Management	0	husband	0	Prophylactic	0	Daughter	0	Acetaminophen	0	Parent	0	Present	0	Disability	0	Menopause	0	employ	0	Support group	3	Hemiplegic migraine	0	Neck pain	0	Brother	0	Herbal medicine	0
Ergot	0	Mother	1	Blocker	0	Child	0	travel	0	Cluster	0	nasal	0	pray	0	Management	7	husband	0	Prophylactic	0	Daughter	0	Acetaminophen	0	Parent	0	Present	0	Disability	0	Menopause	0	employ	0	Support group	0	Hemiplegic migraine	0	Neck pain	0	Brother	0	Herbal medicine	0
Ergot	0	Mother	0	Blocker	0	Child	0	travel	0	Cluster	0	nasal	0	pray	0	Management	0	husband	0	Prophylactic	0	Daughter	0	Acetaminophen	0	Parent	0	Present	0	Disability	0	Menopause	0	employ	0	Support group	5	Hemiplegic migraine	3	Neck pain	0	Brother	0	Herbal medicine	0
Ergot	0	Mother	0	Blocker	0	Child	0	travel	0	Cluster	0	nasal	1	pray	1	Management	1	husband	0	Prophylactic	0	Daughter	0	Acetaminophen	0	Parent	0	Present	1	Disability	0	Menopause	2	employ	0	Support group	0	Hemiplegic migraine	0	Neck pain	1	Brother	0	Herbal medicine	0
Ergot	0	Mother	0	Blocker	0	Child	0	travel	0	Cluster	0	nasal	0	pray	0	Management	0	husband	0	Prophylactic	0	Daughter	0	Acetaminophen	0	Parent	0	Present	0	Disability	0	Menopause	0	employ	0	Support group	1	Hemiplegic migraine	0	Neck pain	0	Brother	0	Herbal medicine	0
Ergot	0	Mother	0	Blocker	0	Child	0	travel	0	Cluster	5	nasal	0	pray	0	Management	0	husband	0	Prophylactic	0	Daughter	0	Acetaminophen	0	Parent	0	Present	0	Disability	12	Menopause	0	employ	9	Support group	0	Hemiplegic migraine	0	Neck pain	0	Brother	2	Herbal medicine	0
Ergot	3	Mother	1	Blocker	7	Child	2	travel	1	Cluster	0	nasal	1	pray	0	Management	1	husband	0	Prophylactic	13	Daughter	2	Acetaminophen	0	Parent	0	Present	2	Disability	0	Menopause	0	employ	0	Support group	1	Hemiplegic migraine	4	Neck pain	0	Brother	0	Herbal medicine	0
Ergot	0	Mother	0	Blocker	1	Child	0	travel	0	Cluster	0	nasal	0	pray	0	Management	0	husband	0	Prophylactic	0	Daughter	0	Acetaminophen	0	Parent	0	Present	0	Disability	0	Menopause	0	employ	0	Support group	0	Hemiplegic migraine	0	Neck pain	0	Brother	0	Herbal medicine	0
Ergot	0	Mother	0	Blocker	0	Child	0	travel	0	Cluster	0	nasal	0	pray	0	Management	0	husband	0	Prophylactic	0	Daughter	0	Acetaminophen	3	Parent	0	Present	0	Disability	0	Menopause	0	employ	0	Support group	0	Hemiplegic migraine	0	Neck pain	0	Brother	1	Herbal medicine	0
Ergot	0	Mother	0	Blocker	0	Child	0	travel	0	Cluster	0	nasal	0	pray	0	Management	0	husband	0	Prophylactic	0	Daughter	0	Acetaminophen	0	Parent	0	Present	0	Disability	0	Menopause	0	employ	0	Support group	0	Hemiplegic migraine	0	Neck pain	0	Brother	0	Herbal medicine	0
Ergot	0	Mother	0	Blocker	0	Child	0	travel	0	Cluster	1	nasal	0	pray	0	Management	0	husband	0	Prophylactic	0	Daughter	0	Acetaminophen	0	Parent	0	Present	0	Disability	0	Menopause	0	employ	0	Support group	0	Hemiplegic migraine	0	Neck pain	0	Brother	0	Herbal medicine	0
Ergot	0	Mother	0	Blocker	0	Child	0	travel	0	Cluster	0	nasal	0	pray	2	Management	0	husband	0	Prophylactic	0	Daughter	3	Acetaminophen	0	Parent	0	Present	0	Disability	0	Menopause	3	employ	0	Support group	0	Hemiplegic migraine	0	Neck pain	0	Brother	0	Herbal medicine	0
Ergot	0	Mother	2	Blocker	0	Child	3	travel	1	Cluster	1	nasal	2	pray	2	Management	0	husband	1	Prophylactic	0	Daughter	0	Acetaminophen	0	Parent	0	Present	1	Disability	0	Menopause	0	employ	0	Support group	0	Hemiplegic migraine	0	Neck pain	0	Brother	0	Herbal medicine	0
Ergot	0	Mother	1	Blocker	0	Child	0	travel	0	Cluster	3	nasal	0	pray	0	Management	0	husband	0	Prophylactic	0	Daughter	1	Acetaminophen	0	Parent	0	Present	0	Disability	0	Menopause	0	employ	0	Support group	0	Hemiplegic migraine	0	Neck pain	0	Brother	0	Herbal medicine	0
Ergot	1	Mother	0	Blocker	1	Child	0	travel	0	Cluster	0	nasal	0	pray	0	Management	0	husband	0	Prophylactic	0	Daughter	0	Acetaminophen	0	Parent	0	Present	0	Disability	0	Menopause	0	employ	0	Support group	0	Hemiplegic migraine	0	Neck pain	0	Brother	0	Herbal medicine	0
Ergot	0	Mother	0	Blocker	1	Child	1	travel	0	Cluster	0	nasal	0	pray	0	Management	0	husband	0	Prophylactic	0	Daughter	0	Acetaminophen	0	Parent	0	Present	0	Disability	0	Menopause	0	employ	0	Support group	0	Hemiplegic migraine	4	Neck pain	0	Brother	0	Herbal medicine	0
Ergot	0	Mother	1	Blocker	0	Child	1	travel	11	Cluster	0	nasal	0	pray	1	Management	0	husband	4	Prophylactic	1	Daughter	2	Acetaminophen	0	Parent	0	Present	0	Disability	0	Menopause	0	employ	0	Support group	0	Hemiplegic migraine	0	Neck pain	0	Brother	0	Herbal medicine	0
Ergot	4	Mother	3	Blocker	2	Child	0	travel	0	Cluster	0	nasal	0	pray	0	Management	0	husband	0	Prophylactic	0	Daughter	0	Acetaminophen	0	Parent	0	Present	0	Disability	0	Menopause	0	employ	0	Support group	0	Hemiplegic migraine	0	Neck pain	0	Brother	0	Herbal medicine	0
Ergot	0	Mother	0	Blocker	2	Child	0	travel	0	Cluster	0	nasal	0	pray	0	Management	0	husband	0	Prophylactic	0	Daughter	0	Acetaminophen	0	Parent	0	Present	0	Disability	0	Menopause	0	employ	0	Support group	0	Hemiplegic migraine	0	Neck pain	0	Brother	0	Herbal medicine	0
	24		23		23		23		23		23		20		20		20		19		19		19		19		19		19		18		18		17		17		17		16		16		15

P92a	P92b	P93a	P93b	P94a	P94b	P95a	P95b	P96a	P96b	P97a	P97b	P98a	P98b	P99a	P99b	P100a	P100b	P101a	P101b	P102a	P102b	P103a	P103b	P104a	P104b	P105a	P105b	P106a	P106b	P107a	P107b	P108a	P108b	P109a	P109b	P110a	P110b	P111a	P111b	P112a	P112b	P113a	P113b	P114a	P114b
Genetic	0	ARB	0	Cluster headache	0	Abortive	0	Depressed	1	Visual disturbance	0	Photophobia	0	Inhibitor	0	Vestibular migraine	0	Stages	0	Sensitivity to light	0	Education	0	Surgery	0	Drive	0	Acute	0	Wife	0	Lifestyle	1	Impact	1	History	1	Allodynia	0	Pregnancy	0	Antidepressants	0	Sister	0
Genetic	0	ARB	0	Cluster headache	0	Abortive	2	Depressed	0	Visual disturbance	0	Photophobia	0	Inhibitor	0	Vestibular migraine	0	Stages	0	Sensitivity to light	0	Education	0	Surgery	0	Drive	0	Acute	1	Wife	0	Lifestyle	0	Impact	2	History	0	Allodynia	0	Pregnancy	0	Antidepressants	0	Sister	0
Genetic	0	ARB	0	Cluster headache	0	Abortive	0	Depressed	0	Visual disturbance	0	Photophobia	0	Inhibitor	0	Vestibular migraine	0	Stages	0	Sensitivity to light	0	Education	0	Surgery	0	Drive	1	Acute	0	Wife	0	Lifestyle	0	Impact	0	History	1	Allodynia	0	Pregnancy	0	Antidepressants	0	Sister	0
Genetic	0	ARB	0	Cluster headache	0	Abortive	0	Depressed	0	Visual disturbance	0	Photophobia	0	Inhibitor	0	Vestibular migraine	0	Stages	0	Sensitivity to light	0	Education	0	Surgery	1	Drive	0	Acute	0	Wife	0	Lifestyle	0	Impact	0	History	0	Allodynia	0	Pregnancy	0	Antidepressants	0	Sister	0
Genetic	0	ARB	0	Cluster headache	0	Abortive	0	Depressed	0	Visual disturbance	0	Photophobia	1	Inhibitor	0	Vestibular migraine	0	Stages	0	Sensitivity to light	0	Education	0	Surgery	0	Drive	0	Acute	1	Wife	0	Lifestyle	0	Impact	2	History	0	Allodynia	0	Pregnancy	0	Antidepressants	0	Sister	0
Genetic	0	ARB	0	Cluster headache	0	Abortive	0	Depressed	0	Visual disturbance	0	Photophobia	0	Inhibitor	0	Vestibular migraine	0	Stages	0	Sensitivity to light	0	Education	1	Surgery	0	Drive	0	Acute	0	Wife	0	Lifestyle	0	Impact	0	History	0	Allodynia	0	Pregnancy	0	Antidepressants	0	Sister	0
Genetic	0	ARB	0	Cluster headache	0	Abortive	1	Depressed	0	Visual disturbance	0	Photophobia	0	Inhibitor	0	Vestibular migraine	0	Stages	0	Sensitivity to light	0	Education	0	Surgery	0	Drive	0	Acute	0	Wife	1	Lifestyle	0	Impact	0	History	0	Allodynia	0	Pregnancy	0	Antidepressants	0	Sister	0
Genetic	0	ARB	0	Cluster headache	0	Abortive	0	Depressed	0	Visual disturbance	0	Photophobia	0	Inhibitor	0	Vestibular migraine	0	Stages	0	Sensitivity to light	0	Education	0	Surgery	0	Drive	0	Acute	0	Wife	0	Lifestyle	0	Impact	0	History	0	Allodynia	0	Pregnancy	0	Antidepressants	0	Sister	0
Genetic	0	ARB	0	Cluster headache	0	Abortive	0	Depressed	0	Visual disturbance	0	Photophobia	0	Inhibitor	0	Vestibular migraine	0	Stages	0	Sensitivity to light	1	Education	0	Surgery	0	Drive	0	Acute	0	Wife	0	Lifestyle	0	Impact	0	History	0	Allodynia	0	Pregnancy	0	Antidepressants	0	Sister	0
Genetic	0	ARB	0	Cluster headache	0	Abortive	0	Depressed	0	Visual disturbance	0	Photophobia	0	Inhibitor	0	Vestibular migraine	0	Stages	0	Sensitivity to light	0	Education	1	Surgery	0	Drive	1	Acute	0	Wife	0	Lifestyle	0	Impact	0	History	0	Allodynia	0	Pregnancy	0	Antidepressants	0	Sister	0
Genetic	0	ARB	0	Cluster headache	0	Abortive	2	Depressed	0	Visual disturbance	0	Photophobia	0	Inhibitor	0	Vestibular migraine	0	Stages	0	Sensitivity to light	0	Education	0	Surgery	0	Drive	0	Acute	7	Wife	0	Lifestyle	0	Impact	0	History	0	Allodynia	0	Pregnancy	0	Antidepressants	0	Sister	0
Genetic	0	ARB	0	Cluster headache	1	Abortive	0	Depressed	0	Visual disturbance	0	Photophobia	0	Inhibitor	0	Vestibular migraine	0	Stages	0	Sensitivity to light	0	Education	1	Surgery	0	Drive	0	Acute	0	Wife	0	Lifestyle	0	Impact	1	History	0	Allodynia	0	Pregnancy	0	Antidepressants	0	Sister	0
Genetic	0	ARB	1	Cluster headache	0	Abortive	0	Depressed	5	Visual disturbance	0	Photophobia	5	Inhibitor	0	Vestibular migraine	0	Stages	0	Sensitivity to light	0	Education	0	Surgery	0	Drive	0	Acute	0	Wife	0	Lifestyle	0	Impact	0	History	1	Allodynia	0	Pregnancy	0	Antidepressants	0	Sister	0
Genetic	0	ARB	0	Cluster headache	1	Abortive	0	Depressed	0	Visual disturbance	1	Photophobia	0	Inhibitor	0	Vestibular migraine	0	Stages	0	Sensitivity to light	0	Education	1	Surgery	0	Drive	0	Acute	2	Wife	0	Lifestyle	0	Impact	0	History	0	Allodynia	0	Pregnancy	0	Antidepressants	0	Sister	0
Genetic	0	ARB	0	Cluster headache	0	Abortive	0	Depressed	0	Visual disturbance	0	Photophobia	0	Inhibitor	0	Vestibular migraine	0	Stages	0	Sensitivity to light	0	Education	0	Surgery	0	Drive	0	Acute	0	Wife	0	Lifestyle	0	Impact	0	History	0	Allodynia	0	Pregnancy	0	Antidepressants	0	Sister	0
Genetic	1	ARB	0	Cluster headache	0	Abortive	0	Depressed	0	Visual disturbance	1	Photophobia	0	Inhibitor	0	Vestibular migraine	0	Stages	9	Sensitivity to light	0	Education	1	Surgery	0	Drive	0	Acute	0	Wife	0	Lifestyle	0	Impact	0	History	0	Allodynia	0	Pregnancy	0	Antidepressants	0	Sister	0
Genetic	0	ARB	0	Cluster headache	0	Abortive	0	Depressed	0	Visual disturbance	0	Photophobia	0	Inhibitor	0	Vestibular migraine	0	Stages	0	Sensitivity to light	0	Education	0	Surgery	0	Drive	0	Acute	0	Wife	0	Lifestyle	0	Impact	0	History	0	Allodynia	0	Pregnancy	0	Antidepressants	0	Sister	0
Genetic	0	ARB	0	Cluster headache	2	Abortive	0	Depressed	0	Visual disturbance	0	Photophobia	0	Inhibitor	0	Vestibular migraine	0	Stages	0	Sensitivity to light	0	Education	0	Surgery	1	Drive	0	Acute	0	Wife	1	Lifestyle	0	Impact	0	History	1	Allodynia	0	Pregnancy	0	Antidepressants	0	Sister	0
Genetic	0	ARB	0	Cluster headache	0	Abortive	0	Depressed	0	Visual disturbance	0	Photophobia	0	Inhibitor	0	Vestibular migraine	0	Stages	0	Sensitivity to light	0	Education	0	Surgery	0	Drive	0	Acute	0	Wife	0	Lifestyle	0	Impact	0	History	0	Allodynia	0	Pregnancy	0	Antidepressants	0	Sister	0
Genetic	0	ARB	1	Cluster headache	0	Abortive	0	Depressed	0	Visual disturbance	4	Photophobia	0	Inhibitor	0	Vestibular migraine	0	Stages	0	Sensitivity to light	0	Education	0	Surgery	0	Drive	0	Acute	0	Wife	0	Lifestyle	0	Impact	0	History	1	Allodynia	0	Pregnancy	0	Antidepressants	0	Sister	1
Genetic	0	ARB	0	Cluster headache	0	Abortive	0	Depressed	0	Visual disturbance	0	Photophobia	0	Inhibitor	0	Vestibular migraine	0	Stages	0	Sensitivity to light	0	Education	0	Surgery	0	Drive	0	Acute	0	Wife	0	Lifestyle	2	Impact	0	History	0	Allodynia	0	Pregnancy	0	Antidepressants	0	Sister	0
Genetic	0	ARB	0	Cluster headache	0	Abortive	0	Depressed	1	Visual disturbance	0	Photophobia	0	Inhibitor	0	Vestibular migraine	0	Stages	1	Sensitivity to light	1	Education	0	Surgery	0	Drive	0	Acute	0	Wife	0	Lifestyle	4	Impact	1	History	0	Allodynia	0	Pregnancy	0	Antidepressants	0	Sister	0
Genetic	0	ARB	0	Cluster headache	0	Abortive	0	Depressed	0	Visual disturbance	0	Photophobia	0	Inhibitor	0	Vestibular migraine	0	Stages	1	Sensitivity to light	0	Education	0	Surgery	0	Drive	0	Acute	0	Wife	0	Lifestyle	0	Impact	0	History	0	Allodynia	0	Pregnancy	0	Antidepressants	0	Sister	1
Genetic	0	ARB	0	Cluster headache	0	Abortive	0	Depressed	0	Visual disturbance	0	Photophobia	0	Inhibitor	0	Vestibular migraine	0	Stages	0	Sensitivity to light	0	Education	0	Surgery	0	Drive	0	Acute	0	Wife	0	Lifestyle	0	Impact	0	History	0	Allodynia	0	Pregnancy	0	Antidepressants	0	Sister	0
Genetic	0	ARB	0	Cluster headache	0	Abortive	0	Depressed	0	Visual disturbance	0	Photophobia	0	Inhibitor	0	Vestibular migraine	0	Stages	0	Sensitivity to light	0	Education	0	Surgery	0	Drive	0	Acute	0	Wife	0	Lifestyle	0	Impact	0	History	0	Allodynia	0	Pregnancy	0	Antidepressants	0	Sister	0
Genetic	0	ARB	0	Cluster headache	0	Abortive	0	Depressed	0	Visual disturbance	0	Photophobia	0	Inhibitor	0	Vestibular migraine	0	Stages	0	Sensitivity to light	0	Education	0	Surgery	0	Drive	0	Acute	0	Wife	0	Lifestyle	0	Impact	0	History	0	Allodynia	0	Pregnancy	0	Antidepressants	0	Sister	0
Genetic	3	ARB	0	Cluster headache	0	Abortive	0	Depressed	0	Visual disturbance	0	Photophobia	0	Inhibitor	0	Vestibular migraine	0	Stages	1	Sensitivity to light	0	Education	1	Surgery	0	Drive	0	Acute	0	Wife	0	Lifestyle	0	Impact	0	History	0	Allodynia	0	Pregnancy	0	Antidepressants	0	Sister	0
Genetic	0	ARB	0	Cluster headache	1	Abortive	0	Depressed	0	Visual disturbance	1	Photophobia	0	Inhibitor	0	Vestibular migraine	0	Stages	0	Sensitivity to light	0	Education	0	Surgery	0	Drive	0	Acute	0	Wife	0	Lifestyle	0	Impact	0	History	0	Allodynia	0	Pregnancy	0	Antidepressants	0	Sister	0
Genetic	0	ARB	0	Cluster headache	1	Abortive	0	Depressed	0	Visual disturbance	0	Photophobia	0	Inhibitor	0	Vestibular migraine	0	Stages	0	Sensitivity to light	0	Education	5	Surgery	0	Drive	0	Acute	0	Wife	1	Lifestyle	0	Impact	0	History	1	Allodynia	0	Pregnancy	0	Antidepressants	0	Sister	0
Genetic	0	ARB	0	Cluster headache	0	Abortive	0	Depressed	0	Visual disturbance	0	Photophobia	0	Inhibitor	0	Vestibular migraine	0	Stages	0	Sensitivity to light	0	Education	0	Surgery	0	Drive	0	Acute	0	Wife	0	Lifestyle	0	Impact	0	History	0	Allodynia	0	Pregnancy	0	Antidepressants	0	Sister	0
Genetic	0	ARB	0	Cluster headache	1	Abortive	1	Depressed	0	Visual disturbance	0	Photophobia	0	Inhibitor	0	Vestibular migraine	0	Stages	0	Sensitivity to light	0	Education	0	Surgery	0	Drive	0	Acute	0	Wife	0	Lifestyle	0	Impact	0	History	0	Allodynia	0	Pregnancy	0	Antidepressants	0	Sister	0
Genetic	0	ARB	0	Cluster headache	0	Abortive	0	Depressed	0	Visual disturbance	0	Photophobia	0	Inhibitor	0	Vestibular migraine	0	Stages	0	Sensitivity to light	0	Education	0	Surgery	0	Drive	0	Acute	0	Wife	0	Lifestyle	0	Impact	0	History	0	Allodynia	0	Pregnancy	0	Antidepressants	0	Sister	0
Genetic	0	ARB	0	Cluster headache	0	Abortive	0	Depressed	0	Visual disturbance	0	Photophobia	0	Inhibitor	0	Vestibular migraine	0	Stages	0	Sensitivity to light	0	Education	0	Surgery	0	Drive	0	Acute	0	Wife	0	Lifestyle	0	Impact	0	History	0	Allodynia	0	Pregnancy	0	Antidepressants	0	Sister	0
Genetic	0	ARB	0	Cluster headache	0	Abortive	0	Depressed	0	Visual disturbance	0	Photophobia	0	Inhibitor	0	Vestibular migraine	0	Stages	0	Sensitivity to light	0	Education	0	Surgery	0	Drive	0	Acute	0	Wife	0	Lifestyle	0	Impact	0	History	0	Allodynia	0	Pregnancy	0	Antidepressants	0	Sister	0
Genetic	0	ARB	0	Cluster headache	0	Abortive	0	Depressed	0	Visual disturbance	0	Photophobia	0	Inhibitor	0	Vestibular migraine	0	Stages	0	Sensitivity to light	2	Education	0	Surgery	0	Drive	1	Acute	0	Wife	0	Lifestyle	0	Impact	0	History	0	Allodynia	0	Pregnancy	0	Antidepressants	0	Sister	1
Genetic	0	ARB	0	Cluster headache	0	Abortive	0	Depressed	0	Visual disturbance	0	Photophobia	0	Inhibitor	0	Vestibular migraine	13	Stages	0	Sensitivity to light	0	Education	0	Surgery	1	Drive	2	Acute	0	Wife	1	Lifestyle	0	Impact	0	History	0	Allodynia	0	Pregnancy	0	Antidepressants	0	Sister	1
Genetic	0	ARB	0	Cluster headache	0	Abortive	0	Depressed	0	Visual disturbance	0	Photophobia	0	Inhibitor	0	Vestibular migraine	0	Stages	0	Sensitivity to light	0	Education	0	Surgery	0	Drive	0	Acute	0	Wife	0	Lifestyle	0	Impact	0	History	0	Allodynia	0	Pregnancy	0	Antidepressants	0	Sister	0
Genetic	1	ARB	1	Cluster headache	0	Abortive	0	Depressed	0	Visual disturbance	0	Photophobia	0	Inhibitor	0	Vestibular migraine	0	Stages	0	Sensitivity to light	0	Education	0	Surgery	0	Drive	1	Acute	0	Wife	0	Lifestyle	0	Impact	1	History	0	Allodynia	0	Pregnancy	0	Antidepressants	0	Sister	0
Genetic	0	ARB	0	Cluster headache	0	Abortive	0	Depressed	0	Visual disturbance	0	Photophobia	0	Inhibitor	0	Vestibular migraine	0	Stages	0	Sensitivity to light	0	Education	0	Surgery	0	Drive	0	Acute	0	Wife	0	Lifestyle	0	Impact	0	History	0	Allodynia	0	Pregnancy	0	Antidepressants	0	Sister	0
Genetic	1	ARB	0	Cluster headache	0	Abortive	0	Depressed	0	Visual disturbance	0	Photophobia	0	Inhibitor	0	Vestibular migraine	0	Stages	0	Sensitivity to light	0	Education	0	Surgery	0	Drive	0	Acute	0	Wife	0	Lifestyle	0	Impact	0	History	0	Allodynia	0	Pregnancy	0	Antidepressants	0	Sister	0
Genetic	6	ARB	8	Cluster headache	0	Abortive	6	Depressed	8	Visual disturbance	2	Photophobia	8	Inhibitor	0	Vestibular migraine	0	Stages	0	Sensitivity to light	7	Education	0	Surgery	2	Drive	2	Acute	1	Wife	5	Lifestyle	4	Impact	1	History	1	Allodynia	10	Pregnancy	4	Antidepressants	2	Sister	4
Genetic	1	ARB	0	Cluster headache	0	Abortive	0	Depressed	0	Visual disturbance	0	Photophobia	0	Inhibitor	0	Vestibular migraine	0	Stages	0	Sensitivity to light	0	Education	0	Surgery	0	Drive	0	Acute	0	Wife	0	Lifestyle	0	Impact	0	History	0	Allodynia	0	Pregnancy	0	Antidepressants	0	Sister	0
Genetic	0	ARB	0	Cluster headache	0	Abortive	0	Depressed	0	Visual disturbance	2	Photophobia	0	Inhibitor	0	Vestibular migraine	0	Stages	0	Sensitivity to light	0	Education	0	Surgery	0	Drive	0	Acute	0	Wife	0	Lifestyle	0	Impact	0	History	0	Allodynia	0	Pregnancy	0	Antidepressants	0	Sister	0
Genetic	0	ARB	0	Cluster headache	0	Abortive	0	Depressed	0	Visual disturbance	0	Photophobia	0	Inhibitor	0	Vestibular migraine	0	Stages	0	Sensitivity to light	0	Education	0	Surgery	0	Drive	0	Acute	0	Wife	0	Lifestyle	0	Impact	0	History	0	Allodynia	0	Pregnancy	0	Antidepressants	0	Sister	0
Genetic	0	ARB	0	Cluster headache	2	Abortive	0	Depressed	0	Visual disturbance	0	Photophobia	0	Inhibitor	0	Vestibular migraine	0	Stages	0	Sensitivity to light	0	Education	0	Surgery	0	Drive	0	Acute	0	Wife	0	Lifestyle	0	Impact	0	History	0	Allodynia	0	Pregnancy	0	Antidepressants	0	Sister	0
Genetic	0	ARB	0	Cluster headache	1	Abortive	0	Depressed	0	Visual disturbance	0	Photophobia	0	Inhibitor	0	Vestibular migraine	0	Stages	0	Sensitivity to light	0	Education	1	Surgery	0	Drive	0	Acute	0	Wife	0	Lifestyle	0	Impact	0	History	0	Allodynia	0	Pregnancy	0	Antidepressants	0	Sister	0
Genetic	0	ARB	0	Cluster headache	0	Abortive	0	Depressed	0	Visual disturbance	0	Photophobia	0	Inhibitor	0	Vestibular migraine	0	Stages	0	Sensitivity to light	0	Education	0	Surgery	1	Drive	0	Acute	0	Wife	0	Lifestyle	0	Impact	1	History	0	Allodynia	0	Pregnancy	0	Antidepressants	0	Sister	0
Genetic	0	ARB	0	Cluster headache	0	Abortive	0	Depressed	0	Visual disturbance	0	Photophobia	0	Inhibitor	0	Vestibular migraine	0	Stages	0	Sensitivity to light	0	Education	0	Surgery	0	Drive	0	Acute	0	Wife	0	Lifestyle	0	Impact	0	History	0	Allodynia	0	Pregnancy	0	Antidepressants	0	Sister	0
Genetic	0	ARB	0	Cluster headache	0	Abortive	0	Depressed	0	Visual disturbance	0	Photophobia	0	Inhibitor	0	Vestibular migraine	0	Stages	0	Sensitivity to light	0	Education	0	Surgery	0	Drive	0	Acute	0	Wife	0	Lifestyle	0	Impact	0	History	0	Allodynia	0	Pregnancy	0	Antidepressants	0	Sister	0
Genetic	0	ARB	0	Cluster headache	0	Abortive	0	Depressed	0	Visual disturbance	0	Photophobia	0	Inhibitor	0	Vestibular migraine	0	Stages	0	Sensitivity to light	0	Education	0	Surgery	0	Drive	0	Acute	0	Wife	0	Lifestyle	0	Impact	0	History	0	Allodynia	0	Pregnancy	0	Antidepressants	0	Sister	0
Genetic	0	ARB	0	Cluster headache	3	Abortive	1	Depressed	0	Visual disturbance	0	Photophobia	0	Inhibitor	0	Vestibular migraine	0	Stages	0	Sensitivity to light	0	Education	0	Surgery	0	Drive	0	Acute	0	Wife	0	Lifestyle	0	Impact	0	History	2	Allodynia	0	Pregnancy	0	Antidepressants	0	Sister	0
Genetic	0	ARB	3	Cluster headache	0	Abortive	2	Depressed	0	Visual disturbance	0	Photophobia	0	Inhibitor	12	Vestibular migraine	0	Stages	0	Sensitivity to light	0	Education	1	Surgery	1	Drive	0	Acute	0	Wife	0	Lifestyle	0	Impact	0	History	0	Allodynia	0	Pregnancy	2	Antidepressants	5	Sister	0
Genetic	0	ARB	0	Cluster headache	0	Abortive	0	Depressed	0	Visual disturbance	0	Photophobia	0	Inhibitor	0	Vestibular migraine	0	Stages	0	Sensitivity to light	0	Education	0	Surgery	0	Drive	0	Acute	0	Wife	0	Lifestyle	0	Impact	0	History	0	Allodynia	0	Pregnancy	0	Antidepressants	0	Sister	0
Genetic	0	ARB	0	Cluster headache	0	Abortive	0	Depressed	0	Visual disturbance	0	Photophobia	0	Inhibitor	0	Vestibular migraine	0	Stages	0	Sensitivity to light	0	Education	0	Surgery	0	Drive	0	Acute	0	Wife	0	Lifestyle	0	Impact	0	History	0	Allodynia	0	Pregnancy	0	Antidepressants	0	Sister	0
Genetic	1	ARB	0	Cluster headache	0	Abortive	0	Depressed	0	Visual disturbance	0	Photophobia	0	Inhibitor	0	Vestibular migraine	0	Stages	0	Sensitivity to light	0	Education	0	Surgery	0	Drive	0	Acute	0	Wife	0	Lifestyle	0	Impact	0	History	0	Allodynia	0	Pregnancy	0	Antidepressants	0	Sister	0
Genetic	0	ARB	0	Cluster headache	1	Abortive	0	Depressed	0	Visual disturbance	0	Photophobia	0	Inhibitor	0	Vestibular migraine	0	Stages	0	Sensitivity to light	0	Education	0	Surgery	0	Drive	0	Acute	0	Wife	0	Lifestyle	0	Impact	0	History	0	Allodynia	0	Pregnancy	0	Antidepressants	0	Sister	0
Genetic	0	ARB	0	Cluster headache	0	Abortive	0	Depressed	0	Visual disturbance	0	Photophobia	0	Inhibitor	0	Vestibular migraine	0	Stages	0	Sensitivity to light	0	Education	0	Surgery	0	Drive	0	Acute	0	Wife	0	Lifestyle	0	Impact	0	History	0	Allodynia	0	Pregnancy	0	Antidepressants	0	Sister	0
Genetic	0	ARB	0	Cluster headache	1	Abortive	0	Depressed	0	Visual disturbance	2	Photophobia	0	Inhibitor	0	Vestibular migraine	0	Stages	0	Sensitivity to light	1	Education	0	Surgery	5	Drive	3	Acute	0	Wife	2	Lifestyle	0	Impact	0	History	0	Allodynia	0	Pregnancy	0	Antidepressants	3	Sister	2
Genetic	0	ARB	0	Cluster headache	0	Abortive	0	Depressed	0	Visual disturbance	1	Photophobia	0	Inhibitor	0	Vestibular migraine	0	Stages	0	Sensitivity to light	0	Education	0	Surgery	0	Drive	0	Acute	0	Wife	0	Lifestyle	0	Impact	0	History	0	Allodynia	0	Pregnancy	0	Antidepressants	0	Sister	0
Genetic	0	ARB	0	Cluster headache	0	Abortive	0	Depressed	0	Visual disturbance	0	Photophobia	0	Inhibitor	0	Vestibular migraine	0	Stages	0	Sensitivity to light	0	Education	0	Surgery	0	Drive	0	Acute	0	Wife	0	Lifestyle	0	Impact	1	History	0	Allodynia	0	Pregnancy	0	Antidepressants	0	Sister	0
Genetic	1	ARB	1	Cluster headache	0	Abortive	0	Depressed	0	Visual disturbance	0	Photophobia	0	Inhibitor	0	Vestibular migraine	0	Stages	1	Sensitivity to light	1	Education	0	Surgery	0	Drive	0	Acute	0	Wife	0	Lifestyle	0	Impact	0	History	1	Allodynia	0	Pregnancy	0	Antidepressants	0	Sister	0
Genetic	0	ARB	0	Cluster headache	0	Abortive	0	Depressed	0	Visual disturbance	0	Photophobia	0	Inhibitor	0	Vestibular migraine	0	Stages	0	Sensitivity to light	0	Education	0	Surgery	0	Drive	1	Acute	0	Wife	0	Lifestyle	0	Impact	0	History	1	Allodynia	0	Pregnancy	0	Antidepressants	0	Sister	0
Genetic	0	ARB	0	Cluster headache	0	Abortive	0	Depressed	0	Visual disturbance	0	Photophobia	0	Inhibitor	1	Vestibular migraine	0	Stages	0	Sensitivity to light	0	Education	0	Surgery	0	Drive	0	Acute	0	Wife	0	Lifestyle	0	Impact	0	History	0	Allodynia	0	Pregnancy	3	Antidepressants	0	Sister	0
Genetic	0	ARB	0	Cluster headache	0	Abortive	0	Depressed	0	Visual disturbance	0	Photophobia	0	Inhibitor	0	Vestibular migraine	0	Stages	0	Sensitivity to light	0	Education	0	Surgery	0	Drive	0	Acute	0	Wife	0	Lifestyle	0	Impact	0	History	0	Allodynia	0	Pregnancy	1	Antidepressants	0	Sister	0
	15		15		15		15		15		14		14		13		13		13		13		13		12		12		12		11		11		11		11		10		10		10		10

P115a	P115b	P116a	P116b	P117a	P117b	P118a	P118b	P119a	P119b	P120a	P120b	P121a	P121b	P122a	P122b	P123a	P123b	P124a	P124b	P125a	P125b	P126a	P126b	P127a	P127b	P128a	P128b	P129a	P129b	P130a	P130b	P131a	P131b	P132a	P132b	P133a	P133b	P134a	P134b	P135a	P135b	P136a
Riboflavin	0	Sore	0	Occipital	0	minister	0	Emergency	1	Butalbital	0	Calcium channel	0	Dopamine	0	Intractable	0	Hurts	0	Toxin	0	Peer	0	procedure	0	Procedure	0	Coping	0	Biofeedback	0	Nurse	0	Vascular	0	Preventive	0	Sad	0	Adverse effect	0	Confuse
Riboflavin	0	Sore	0	Occipital	0	minister	0	Emergency	0	Butalbital	0	Calcium channel	0	Dopamine	0	Intractable	0	Hurts	0	Toxin	0	Peer	0	procedure	0	Procedure	0	Coping	2	Biofeedback	0	Nurse	0	Vascular	0	Preventive	0	Sad	0	Adverse effect	1	Confuse
Riboflavin	0	Sore	0	Occipital	1	minister	1	Emergency	0	Butalbital	0	Calcium channel	0	Dopamine	0	Intractable	0	Hurts	0	Toxin	0	Peer	0	procedure	0	Procedure	0	Coping	0	Biofeedback	0	Nurse	0	Vascular	0	Preventive	0	Sad	0	Adverse effect	0	Confuse
Riboflavin	0	Sore	0	Occipital	0	minister	0	Emergency	0	Butalbital	0	Calcium channel	0	Dopamine	0	Intractable	0	Hurts	0	Toxin	0	Peer	0	procedure	1	Procedure	1	Coping	0	Biofeedback	0	Nurse	0	Vascular	0	Preventive	0	Sad	0	Adverse effect	0	Confuse
Riboflavin	0	Sore	0	Occipital	0	minister	0	Emergency	0	Butalbital	0	Calcium channel	0	Dopamine	0	Intractable	0	Hurts	0	Toxin	0	Peer	0	procedure	0	Procedure	0	Coping	0	Biofeedback	0	Nurse	0	Vascular	0	Preventive	0	Sad	1	Adverse effect	0	Confuse
Riboflavin	0	Sore	0	Occipital	0	minister	0	Emergency	0	Butalbital	0	Calcium channel	0	Dopamine	0	Intractable	0	Hurts	0	Toxin	0	Peer	0	procedure	0	Procedure	0	Coping	0	Biofeedback	0	Nurse	0	Vascular	0	Preventive	0	Sad	0	Adverse effect	0	Confuse
Riboflavin	0	Sore	0	Occipital	0	minister	0	Emergency	0	Butalbital	0	Calcium channel	0	Dopamine	0	Intractable	0	Hurts	0	Toxin	0	Peer	0	procedure	0	Procedure	0	Coping	0	Biofeedback	0	Nurse	0	Vascular	0	Preventive	0	Sad	0	Adverse effect	0	Confuse
Riboflavin	0	Sore	0	Occipital	0	minister	0	Emergency	0	Butalbital	0	Calcium channel	0	Dopamine	0	Intractable	0	Hurts	0	Toxin	0	Peer	0	procedure	0	Procedure	0	Coping	0	Biofeedback	0	Nurse	0	Vascular	0	Preventive	0	Sad	0	Adverse effect	0	Confuse
Riboflavin	0	Sore	0	Occipital	0	minister	0	Emergency	0	Butalbital	0	Calcium channel	0	Dopamine	0	Intractable	0	Hurts	0	Toxin	0	Peer	6	procedure	0	Procedure	0	Coping	0	Biofeedback	0	Nurse	0	Vascular	0	Preventive	0	Sad	0	Adverse effect	0	Confuse
Riboflavin	0	Sore	0	Occipital	0	minister	0	Emergency	0	Butalbital	0	Calcium channel	0	Dopamine	0	Intractable	0	Hurts	0	Toxin	0	Peer	0	procedure	0	Procedure	0	Coping	0	Biofeedback	0	Nurse	0	Vascular	0	Preventive	1	Sad	0	Adverse effect	0	Confuse
Riboflavin	0	Sore	0	Occipital	0	minister	0	Emergency	0	Butalbital	0	Calcium channel	0	Dopamine	0	Intractable	0	Hurts	0	Toxin	0	Peer	0	procedure	0	Procedure	0	Coping	0	Biofeedback	0	Nurse	0	Vascular	0	Preventive	0	Sad	0	Adverse effect	0	Confuse
Riboflavin	0	Sore	0	Occipital	0	minister	0	Emergency	0	Butalbital	0	Calcium channel	0	Dopamine	0	Intractable	0	Hurts	0	Toxin	0	Peer	0	procedure	0	Procedure	0	Coping	0	Biofeedback	0	Nurse	0	Vascular	0	Preventive	0	Sad	0	Adverse effect	0	Confuse
Riboflavin	0	Sore	0	Occipital	0	minister	0	Emergency	0	Butalbital	0	Calcium channel	0	Dopamine	0	Intractable	0	Hurts	0	Toxin	0	Peer	0	procedure	0	Procedure	0	Coping	0	Biofeedback	0	Nurse	0	Vascular	0	Preventive	0	Sad	0	Adverse effect	0	Confuse
Riboflavin	0	Sore	0	Occipital	0	minister	0	Emergency	2	Butalbital	0	Calcium channel	0	Dopamine	0	Intractable	0	Hurts	0	Toxin	0	Peer	0	procedure	1	Procedure	1	Coping	0	Biofeedback	0	Nurse	0	Vascular	0	Preventive	0	Sad	0	Adverse effect	0	Confuse
Riboflavin	0	Sore	0	Occipital	0	minister	0	Emergency	0	Butalbital	0	Calcium channel	0	Dopamine	0	Intractable	0	Hurts	0	Toxin	0	Peer	0	procedure	0	Procedure	0	Coping	0	Biofeedback	0	Nurse	0	Vascular	0	Preventive	0	Sad	0	Adverse effect	0	Confuse
Riboflavin	0	Sore	0	Occipital	0	minister	0	Emergency	0	Butalbital	0	Calcium channel	0	Dopamine	0	Intractable	0	Hurts	0	Toxin	0	Peer	0	procedure	0	Procedure	0	Coping	0	Biofeedback	0	Nurse	0	Vascular	0	Preventive	0	Sad	2	Adverse effect	0	Confuse
Riboflavin	0	Sore	0	Occipital	0	minister	0	Emergency	0	Butalbital	0	Calcium channel	0	Dopamine	0	Intractable	0	Hurts	0	Toxin	0	Peer	0	procedure	0	Procedure	0	Coping	0	Biofeedback	0	Nurse	0	Vascular	0	Preventive	0	Sad	0	Adverse effect	0	Confuse
Riboflavin	0	Sore	1	Occipital	0	minister	0	Emergency	0	Butalbital	0	Calcium channel	0	Dopamine	0	Intractable	0	Hurts	0	Toxin	2	Peer	0	procedure	1	Procedure	1	Coping	0	Biofeedback	0	Nurse	0	Vascular	0	Preventive	0	Sad	0	Adverse effect	0	Confuse
Riboflavin	0	Sore	0	Occipital	0	minister	0	Emergency	0	Butalbital	0	Calcium channel	0	Dopamine	0	Intractable	0	Hurts	0	Toxin	0	Peer	0	procedure	0	Procedure	0	Coping	0	Biofeedback	0	Nurse	0	Vascular	0	Preventive	0	Sad	0	Adverse effect	0	Confuse
Riboflavin	1	Sore	0	Occipital	0	minister	0	Emergency	2	Butalbital	0	Calcium channel	0	Dopamine	0	Intractable	0	Hurts	0	Toxin	0	Peer	0	procedure	0	Procedure	0	Coping	0	Biofeedback	0	Nurse	0	Vascular	1	Preventive	0	Sad	1	Adverse effect	4	Confuse
Riboflavin	0	Sore	0	Occipital	0	minister	0	Emergency	0	Butalbital	0	Calcium channel	0	Dopamine	0	Intractable	0	Hurts	0	Toxin	2	Peer	0	procedure	0	Procedure	0	Coping	0	Biofeedback	0	Nurse	0	Vascular	0	Preventive	0	Sad	0	Adverse effect	0	Confuse
Riboflavin	0	Sore	0	Occipital	0	minister	0	Emergency	0	Butalbital	0	Calcium channel	0	Dopamine	0	Intractable	0	Hurts	0	Toxin	0	Peer	0	procedure	0	Procedure	0	Coping	1	Biofeedback	0	Nurse	0	Vascular	0	Preventive	0	Sad	0	Adverse effect	0	Confuse
Riboflavin	0	Sore	0	Occipital	0	minister	0	Emergency	0	Butalbital	0	Calcium channel	0	Dopamine	0	Intractable	0	Hurts	0	Toxin	0	Peer	0	procedure	0	Procedure	0	Coping	0	Biofeedback	0	Nurse	0	Vascular	0	Preventive	0	Sad	0	Adverse effect	0	Confuse
Riboflavin	0	Sore	0	Occipital	0	minister	0	Emergency	0	Butalbital	0	Calcium channel	0	Dopamine	0	Intractable	0	Hurts	1	Toxin	0	Peer	0	procedure	0	Procedure	0	Coping	0	Biofeedback	0	Nurse	0	Vascular	0	Preventive	0	Sad	0	Adverse effect	0	Confuse
Riboflavin	0	Sore	0	Occipital	0	minister	0	Emergency	0	Butalbital	0	Calcium channel	0	Dopamine	0	Intractable	0	Hurts	0	Toxin	0	Peer	0	procedure	0	Procedure	0	Coping	0	Biofeedback	0	Nurse	0	Vascular	0	Preventive	0	Sad	0	Adverse effect	0	Confuse
Riboflavin	0	Sore	0	Occipital	0	minister	0	Emergency	0	Butalbital	0	Calcium channel	0	Dopamine	0	Intractable	0	Hurts	0	Toxin	0	Peer	0	procedure	0	Procedure	0	Coping	0	Biofeedback	0	Nurse	0	Vascular	0	Preventive	0	Sad	0	Adverse effect	0	Confuse
Riboflavin	0	Sore	0	Occipital	0	minister	1	Emergency	0	Butalbital	0	Calcium channel	0	Dopamine	0	Intractable	0	Hurts	0	Toxin	0	Peer	0	procedure	0	Procedure	0	Coping	0	Biofeedback	0	Nurse	0	Vascular	0	Preventive	0	Sad	0	Adverse effect	0	Confuse
Riboflavin	1	Sore	0	Occipital	0	minister	0	Emergency	0	Butalbital	0	Calcium channel	0	Dopamine	0	Intractable	0	Hurts	0	Toxin	0	Peer	0	procedure	0	Procedure	0	Coping	0	Biofeedback	0	Nurse	0	Vascular	0	Preventive	0	Sad	0	Adverse effect	0	Confuse
Riboflavin	0	Sore	0	Occipital	0	minister	0	Emergency	0	Butalbital	0	Calcium channel	0	Dopamine	0	Intractable	0	Hurts	0	Toxin	0	Peer	1	procedure	0	Procedure	0	Coping	0	Biofeedback	0	Nurse	0	Vascular	0	Preventive	0	Sad	0	Adverse effect	0	Confuse
Riboflavin	0	Sore	0	Occipital	0	minister	0	Emergency	0	Butalbital	0	Calcium channel	0	Dopamine	0	Intractable	0	Hurts	0	Toxin	0	Peer	1	procedure	0	Procedure	0	Coping	0	Biofeedback	0	Nurse	0	Vascular	0	Preventive	0	Sad	0	Adverse effect	0	Confuse
Riboflavin	0	Sore	0	Occipital	0	minister	0	Emergency	0	Butalbital	0	Calcium channel	2	Dopamine	1	Intractable	6	Hurts	0	Toxin	0	Peer	0	procedure	0	Procedure	0	Coping	0	Biofeedback	0	Nurse	0	Vascular	0	Preventive	0	Sad	0	Adverse effect	0	Confuse
Riboflavin	0	Sore	0	Occipital	0	minister	0	Emergency	0	Butalbital	0	Calcium channel	0	Dopamine	0	Intractable	0	Hurts	0	Toxin	0	Peer	0	procedure	0	Procedure	0	Coping	0	Biofeedback	0	Nurse	0	Vascular	0	Preventive	0	Sad	0	Adverse effect	0	Confuse
Riboflavin	0	Sore	0	Occipital	0	minister	2	Emergency	0	Butalbital	4	Calcium channel	0	Dopamine	0	Intractable	0	Hurts	0	Toxin	0	Peer	0	procedure	0	Procedure	0	Coping	0	Biofeedback	0	Nurse	0	Vascular	0	Preventive	0	Sad	0	Adverse effect	1	Confuse
Riboflavin	0	Sore	0	Occipital	0	minister	0	Emergency	1	Butalbital	0	Calcium channel	0	Dopamine	0	Intractable	0	Hurts	0	Toxin	1	Peer	0	procedure	0	Procedure	0	Coping	0	Biofeedback	0	Nurse	0	Vascular	0	Preventive	0	Sad	0	Adverse effect	0	Confuse
Riboflavin	0	Sore	0	Occipital	0	minister	0	Emergency	0	Butalbital	0	Calcium channel	0	Dopamine	0	Intractable	0	Hurts	0	Toxin	0	Peer	0	procedure	0	Procedure	0	Coping	0	Biofeedback	0	Nurse	2	Vascular	0	Preventive	0	Sad	0	Adverse effect	0	Confuse
Riboflavin	0	Sore	0	Occipital	0	minister	0	Emergency	0	Butalbital	0	Calcium channel	0	Dopamine	0	Intractable	0	Hurts	0	Toxin	0	Peer	0	procedure	0	Procedure	0	Coping	0	Biofeedback	0	Nurse	0	Vascular	0	Preventive	0	Sad	0	Adverse effect	0	Confuse
Riboflavin	0	Sore	0	Occipital	0	minister	0	Emergency	0	Butalbital	0	Calcium channel	0	Dopamine	0	Intractable	0	Hurts	0	Toxin	0	Peer	0	procedure	0	Procedure	0	Coping	0	Biofeedback	0	Nurse	0	Vascular	0	Preventive	0	Sad	0	Adverse effect	0	Confuse
Riboflavin	0	Sore	0	Occipital	0	minister	0	Emergency	0	Butalbital	0	Calcium channel	0	Dopamine	0	Intractable	0	Hurts	0	Toxin	0	Peer	0	procedure	0	Procedure	0	Coping	2	Biofeedback	0	Nurse	1	Vascular	0	Preventive	0	Sad	0	Adverse effect	0	Confuse
Riboflavin	0	Sore	0	Occipital	0	minister	0	Emergency	0	Butalbital	0	Calcium channel	0	Dopamine	0	Intractable	0	Hurts	0	Toxin	0	Peer	0	procedure	0	Procedure	0	Coping	0	Biofeedback	0	Nurse	0	Vascular	0	Preventive	0	Sad	0	Adverse effect	0	Confuse
Riboflavin	0	Sore	0	Occipital	0	minister	0	Emergency	0	Butalbital	0	Calcium channel	0	Dopamine	0	Intractable	0	Hurts	0	Toxin	0	Peer	0	procedure	0	Procedure	0	Coping	0	Biofeedback	0	Nurse	0	Vascular	0	Preventive	0	Sad	0	Adverse effect	0	Confuse
Riboflavin	2	Sore	6	Occipital	9	minister	3	Emergency	3	Butalbital	5	Calcium channel	1	Dopamine	0	Intractable	2	Hurts	3	Toxin	2	Peer	0	procedure	6	Procedure	6	Coping	1	Biofeedback	8	Nurse	1	Vascular	2	Preventive	2	Sad	4	Adverse effect	0	Confuse
Riboflavin	0	Sore	0	Occipital	0	minister	0	Emergency	0	Butalbital	0	Calcium channel	0	Dopamine	0	Intractable	0	Hurts	4	Toxin	0	Peer	0	procedure	0	Procedure	0	Coping	0	Biofeedback	0	Nurse	0	Vascular	0	Preventive	0	Sad	0	Adverse effect	0	Confuse
Riboflavin	1	Sore	0	Occipital	0	minister	0	Emergency	0	Butalbital	0	Calcium channel	1	Dopamine	0	Intractable	0	Hurts	0	Toxin	0	Peer	0	procedure	0	Procedure	0	Coping	0	Biofeedback	0	Nurse	0	Vascular	0	Preventive	0	Sad	0	Adverse effect	0	Confuse
Riboflavin	0	Sore	0	Occipital	0	minister	0	Emergency	0	Butalbital	0	Calcium channel	0	Dopamine	0	Intractable	0	Hurts	0	Toxin	0	Peer	0	procedure	0	Procedure	0	Coping	0	Biofeedback	0	Nurse	0	Vascular	0	Preventive	0	Sad	0	Adverse effect	0	Confuse
Riboflavin	0	Sore	0	Occipital	0	minister	0	Emergency	1	Butalbital	0	Calcium channel	0	Dopamine	0	Intractable	0	Hurts	0	Toxin	0	Peer	0	procedure	0	Procedure	0	Coping	0	Biofeedback	0	Nurse	0	Vascular	0	Preventive	0	Sad	0	Adverse effect	0	Confuse
Riboflavin	0	Sore	0	Occipital	0	minister	0	Emergency	0	Butalbital	0	Calcium channel	0	Dopamine	0	Intractable	0	Hurts	0	Toxin	0	Peer	0	procedure	0	Procedure	0	Coping	1	Biofeedback	0	Nurse	0	Vascular	0	Preventive	0	Sad	0	Adverse effect	0	Confuse
Riboflavin	0	Sore	0	Occipital	0	minister	1	Emergency	0	Butalbital	0	Calcium channel	0	Dopamine	0	Intractable	0	Hurts	0	Toxin	0	Peer	1	procedure	0	Procedure	0	Coping	0	Biofeedback	0	Nurse	0	Vascular	0	Preventive	0	Sad	0	Adverse effect	0	Confuse
Riboflavin	0	Sore	0	Occipital	0	minister	0	Emergency	0	Butalbital	0	Calcium channel	0	Dopamine	0	Intractable	1	Hurts	0	Toxin	0	Peer	0	procedure	0	Procedure	0	Coping	0	Biofeedback	0	Nurse	0	Vascular	0	Preventive	0	Sad	0	Adverse effect	0	Confuse
Riboflavin	1	Sore	0	Occipital	0	minister	0	Emergency	0	Butalbital	0	Calcium channel	0	Dopamine	0	Intractable	0	Hurts	0	Toxin	1	Peer	0	procedure	0	Procedure	0	Coping	0	Biofeedback	0	Nurse	0	Vascular	0	Preventive	0	Sad	0	Adverse effect	0	Confuse
Riboflavin	0	Sore	0	Occipital	0	minister	0	Emergency	0	Butalbital	0	Calcium channel	0	Dopamine	0	Intractable	0	Hurts	0	Toxin	0	Peer	0	procedure	0	Procedure	0	Coping	0	Biofeedback	0	Nurse	0	Vascular	0	Preventive	0	Sad	0	Adverse effect	0	Confuse
Riboflavin	0	Sore	0	Occipital	0	minister	0	Emergency	0	Butalbital	0	Calcium channel	0	Dopamine	0	Intractable	0	Hurts	0	Toxin	0	Peer	0	procedure	0	Procedure	0	Coping	0	Biofeedback	0	Nurse	1	Vascular	0	Preventive	1	Sad	0	Adverse effect	0	Confuse
Riboflavin	4	Sore	0	Occipital	0	minister	0	Emergency	0	Butalbital	0	Calcium channel	4	Dopamine	1	Intractable	0	Hurts	0	Toxin	1	Peer	0	procedure	0	Procedure	0	Coping	0	Biofeedback	0	Nurse	0	Vascular	1	Preventive	3	Sad	0	Adverse effect	0	Confuse
Riboflavin	0	Sore	0	Occipital	0	minister	0	Emergency	0	Butalbital	0	Calcium channel	1	Dopamine	0	Intractable	0	Hurts	0	Toxin	0	Peer	0	procedure	0	Procedure	0	Coping	0	Biofeedback	0	Nurse	0	Vascular	0	Preventive	0	Sad	0	Adverse effect	0	Confuse
Riboflavin	0	Sore	0	Occipital	0	minister	0	Emergency	0	Butalbital	0	Calcium channel	0	Dopamine	0	Intractable	0	Hurts	0	Toxin	0	Peer	0	procedure	0	Procedure	0	Coping	0	Biofeedback	0	Nurse	0	Vascular	0	Preventive	0	Sad	0	Adverse effect	0	Confuse
Riboflavin	0	Sore	0	Occipital	0	minister	0	Emergency	0	Butalbital	0	Calcium channel	0	Dopamine	0	Intractable	0	Hurts	0	Toxin	0	Peer	0	procedure	0	Procedure	0	Coping	1	Biofeedback	0	Nurse	0	Vascular	0	Preventive	0	Sad	0	Adverse effect	0	Confuse
Riboflavin	0	Sore	1	Occipital	0	minister	0	Emergency	0	Butalbital	0	Calcium channel	0	Dopamine	0	Intractable	0	Hurts	0	Toxin	0	Peer	0	procedure	0	Procedure	0	Coping	0	Biofeedback	0	Nurse	0	Vascular	0	Preventive	0	Sad	0	Adverse effect	0	Confuse
Riboflavin	0	Sore	0	Occipital	0	minister	0	Emergency	0	Butalbital	0	Calcium channel	0	Dopamine	0	Intractable	0	Hurts	0	Toxin	0	Peer	0	procedure	0	Procedure	0	Coping	1	Biofeedback	0	Nurse	1	Vascular	0	Preventive	0	Sad	0	Adverse effect	0	Confuse
Riboflavin	0	Sore	2	Occipital	0	minister	2	Emergency	0	Butalbital	0	Calcium channel	0	Dopamine	0	Intractable	0	Hurts	0	Toxin	0	Peer	0	procedure	0	Procedure	0	Coping	0	Biofeedback	0	Nurse	0	Vascular	3	Preventive	0	Sad	0	Adverse effect	0	Confuse
Riboflavin	0	Sore	0	Occipital	0	minister	0	Emergency	0	Butalbital	0	Calcium channel	0	Dopamine	0	Intractable	0	Hurts	0	Toxin	0	Peer	0	procedure	0	Procedure	0	Coping	0	Biofeedback	0	Nurse	0	Vascular	0	Preventive	0	Sad	0	Adverse effect	0	Confuse
Riboflavin	0	Sore	0	Occipital	0	minister	0	Emergency	0	Butalbital	0	Calcium channel	0	Dopamine	0	Intractable	0	Hurts	0	Toxin	0	Peer	0	procedure	0	Procedure	0	Coping	0	Biofeedback	0	Nurse	0	Vascular	0	Preventive	0	Sad	0	Adverse effect	0	Confuse
Riboflavin	0	Sore	0	Occipital	0	minister	0	Emergency	0	Butalbital	0	Calcium channel	0	Dopamine	7	Intractable	0	Hurts	0	Toxin	0	Peer	0	procedure	0	Procedure	0	Coping	0	Biofeedback	0	Nurse	0	Vascular	0	Preventive	0	Sad	0	Adverse effect	0	Confuse
Riboflavin	0	Sore	0	Occipital	0	minister	0	Emergency	0	Butalbital	0	Calcium channel	0	Dopamine	0	Intractable	0	Hurts	0	Toxin	0	Peer	0	procedure	0	Procedure	0	Coping	0	Biofeedback	0	Nurse	0	Vascular	1	Preventive	1	Sad	0	Adverse effect	0	Confuse
Riboflavin	0	Sore	0	Occipital	0	minister	0	Emergency	0	Butalbital	0	Calcium channel	0	Dopamine	0	Intractable	0	Hurts	0	Toxin	0	Peer	0	procedure	0	Procedure	0	Coping	0	Biofeedback	0	Nurse	2	Vascular	0	Preventive	0	Sad	0	Adverse effect	2	Confuse
Riboflavin	0	Sore	0	Occipital	0	minister	0	Emergency	0	Butalbital	0	Calcium channel	0	Dopamine	0	Intractable	0	Hurts	1	Toxin	0	Peer	0	procedure	0	Procedure	0	Coping	0	Biofeedback	0	Nurse	0	Vascular	0	Preventive	0	Sad	0	Adverse effect	0	Confuse
	10		10		10		10		10		9		9		9		9		9		9		9		9		9		9		8		8		8		8		8		8	

P136b	P137a	P137b	P138a	P138b	P139a	P139b	P140a	P140b	P141a	P141b	P142a	P142b	P143a	P143b	P144a	P144b	P145a	P145b	P146a	P146b	P147a	P147b	P148a	P148b	P149a	P149b	P150a	P150b	P151a	P151b	P152a	P152b	P153a	P153b	P154a	P154b	P155a	P155b	P156a	P156b	P157a	P157b	P158a	P158b	P159a	P159b	P160a	P160b	P161a	P161b	P162a	P162b	P163a	P163b	P164a	P164b	P165a	P165b	P166a	P166b	P167a	P167b	P168a	P168b	P169a	P169b	P170a	P170b	P171a	P171b	P172a	P172b	P173a	P173b	P174a	P174b	P175a	P175b	P176a	P176b	P177a	P177b	P178a	P178b	P179a	P179b	P180a	P180b	P181a	P181b	P182a	P182b	P183a	P183b	P184a	P184b	P185a	P185b	P186a	P186b	P187a	P187b	P188a	P188b	P189a	P189b	P190a	P190b	P191a	P191b	P192a	P192b	P193a	P193b	P194a	P194b	P195a	P195b	P196a	P196b	P197a	P197b	P198a	P198b
2	Prophylaxis	0	Angiotensin	0	Incidence	0	Duration	0	Patterns	0	Anger	0	Central	0	Future	0	Joy	0	Quality of life	0	Aggravate	0	Motion sickness	0	Steroid	0	Calcium channel blocker	0	faith	0	Shock	0	Population	0	boss	0	vacation	0	Neck muscle	0	Trigger point	0	NSAID	0	Agonist	0	CAT scan	0	Weight loss	0	Testing	0	Epilepsy	0	Peer support	0	stigma	0	Acute migraine	0	Cervical spine	0	Characteristic	0	Sensitivity to sound	0	Peripheral	0	Upset	0	Anti-inflammatory	0	Facial pain	0	Mechanism	0	Grandmother	0	Homeopathy	0	surgery	0	Accident	0	regime	0	Trauma	0	Mindfulness	0	Tricyclic	0	Remission	0	Histamine	0	Headache diary	0	Anti-seizure	0	Occipital nerve	0	Headache clinic	0	Caregiver	0	impairment	0	Glutamate	0	Type A	0	Sinus headache	0	Regimen	0	Lactation	0	Abnormalities	0	Blood flow	0	Efficacy	0
0	Prophylaxis	0	Angiotensin	0	Incidence	0	Duration	0	Patterns	0	Anger	0	Central	0	Future	1	Joy	2	Quality of life	2	Aggravate	0	Motion sickness	0	Steroid	0	Calcium channel blocker	0	faith	0	Shock	0	Population	0	boss	0	vacation	2	Neck muscle	0	Trigger point	0	NSAID	0	Agonist	0	CAT scan	0	Weight loss	0	Testing	0	Epilepsy	0	Peer support	0	stigma	1	Acute migraine	1	Cervical spine	0	Characteristic	0	Sensitivity to sound	0	Peripheral	0	Upset	0	Anti-inflammatory	0	Facial pain	0	Mechanism	0	Grandmother	0	Homeopathy	0	surgery	0	Accident	0	regime	0	Trauma	0	Mindfulness	2	Tricyclic	0	Remission	0	Histamine	0	Headache diary	0	Anti-seizure	0	Occipital nerve	0	Headache clinic	0	Caregiver	0	impairment	0	Glutamate	0	Type A	0	Sinus headache	0	Regimen	0	Lactation	0	Abnormalities	0	Blood flow	0	Efficacy	0
1	Prophylaxis	0	Angiotensin	0	Incidence	0	Duration	0	Patterns	0	Anger	0	Central	1	Future	0	Joy	0	Quality of life	0	Aggravate	0	Motion sickness	0	Steroid	0	Calcium channel blocker	0	faith	0	Shock	0	Population	0	boss	0	vacation	0	Neck muscle	0	Trigger point	0	NSAID	0	Agonist	0	CAT scan	0	Weight loss	0	Testing	0	Epilepsy	0	Peer support	0	stigma	0	Acute migraine	0	Cervical spine	0	Characteristic	0	Sensitivity to sound	0	Peripheral	0	Upset	0	Anti-inflammatory	0	Facial pain	0	Mechanism	0	Grandmother	0	Homeopathy	0	surgery	0	Accident	0	regime	0	Trauma	0	Mindfulness	0	Tricyclic	0	Remission	0	Histamine	0	Headache diary	0	Anti-seizure	0	Occipital nerve	0	Headache clinic	0	Caregiver	0	impairment	0	Glutamate	0	Type A	0	Sinus headache	0	Regimen	0	Lactation	0	Abnormalities	0	Blood flow	0	Efficacy	0
0	Prophylaxis	0	Angiotensin	0	Incidence	0	Duration	0	Patterns	0	Anger	0	Central	0	Future	0	Joy	0	Quality of life	0	Aggravate	0	Motion sickness	0	Steroid	0	Calcium channel blocker	0	faith	0	Shock	0	Population	0	boss	0	vacation	0	Neck muscle	0	Trigger point	0	NSAID	0	Agonist	0	CAT scan	0	Weight loss	0	Testing	0	Epilepsy	0	Peer support	0	stigma	0	Acute migraine	0	Cervical spine	0	Characteristic	0	Sensitivity to sound	0	Peripheral	0	Upset	0	Anti-inflammatory	0	Facial pain	0	Mechanism	0	Grandmother	0	Homeopathy	0	surgery	0	Accident	0	regime	0	Trauma	0	Mindfulness	0	Tricyclic	0	Remission	0	Histamine	0	Headache diary	0	Anti-seizure	0	Occipital nerve	0	Headache clinic	0	Caregiver	0	impairment	0	Glutamate	0	Type A	0	Sinus headache	0	Regimen	0	Lactation	0	Abnormalities	0	Blood flow	0	Efficacy	0
0	Prophylaxis	0	Angiotensin	0	Incidence	0	Duration	0	Patterns	0	Anger	0	Central	0	Future	0	Joy	0	Quality of life	0	Aggravate	0	Motion sickness	0	Steroid	0	Calcium channel blocker	0	faith	0	Shock	0	Population	1	boss	1	vacation	0	Neck muscle	0	Trigger point	0	NSAID	0	Agonist	0	CAT scan	0	Weight loss	0	Testing	0	Epilepsy	0	Peer support	0	stigma	0	Acute migraine	0	Cervical spine	0	Characteristic	0	Sensitivity to sound	0	Peripheral	0	Upset	0	Anti-inflammatory	0	Facial pain	0	Mechanism	0	Grandmother	0	Homeopathy	0	surgery	0	Accident	0	regime	0	Trauma	0	Mindfulness	0	Tricyclic	0	Remission	0	Histamine	0	Headache diary	0	Anti-seizure	0	Occipital nerve	0	Headache clinic	0	Caregiver	0	impairment	0	Glutamate	0	Type A	0	Sinus headache	0	Regimen	0	Lactation	0	Abnormalities	0	Blood flow	0	Efficacy	0
0	Prophylaxis	0	Angiotensin	0	Incidence	0	Duration	0	Patterns	0	Anger	0	Central	1	Future	0	Joy	0	Quality of life	0	Aggravate	0	Motion sickness	0	Steroid	0	Calcium channel blocker	0	faith	0	Shock	0	Population	0	boss	0	vacation	0	Neck muscle	0	Trigger point	0	NSAID	0	Agonist	0	CAT scan	0	Weight loss	0	Testing	0	Epilepsy	0	Peer support	0	stigma	0	Acute migraine	0	Cervical spine	0	Characteristic	0	Sensitivity to sound	0	Peripheral	0	Upset	0	Anti-inflammatory	0	Facial pain	0	Mechanism	0	Grandmother	0	Homeopathy	0	surgery	0	Accident	0	regime	0	Trauma	0	Mindfulness	0	Tricyclic	0	Remission	0	Histamine	0	Headache diary	0	Anti-seizure	0	Occipital nerve	0	Headache clinic	0	Caregiver	0	impairment	0	Glutamate	0	Type A	0	Sinus headache	0	Regimen	0	Lactation	0	Abnormalities	0	Blood flow	0	Efficacy	0
0	Prophylaxis	0	Angiotensin	0	Incidence	0	Duration	0	Patterns	0	Anger	0	Central	0	Future	0	Joy	0	Quality of life	0	Aggravate	0	Motion sickness	0	Steroid	0	Calcium channel blocker	0	faith	0	Shock	0	Population	0	boss	0	vacation	0	Neck muscle	0	Trigger point	0	NSAID	0	Agonist	0	CAT scan	0	Weight loss	0	Testing	0	Epilepsy	0	Peer support	0	stigma	0	Acute migraine	0	Cervical spine	0	Characteristic	0	Sensitivity to sound	0	Peripheral	0	Upset	0	Anti-inflammatory	0	Facial pain	0	Mechanism	0	Grandmother	0	Homeopathy	0	surgery	0	Accident	0	regime	0	Trauma	0	Mindfulness	0	Tricyclic	0	Remission	0	Histamine	0	Headache diary	0	Anti-seizure	0	Occipital nerve	0	Headache clinic	0	Caregiver	0	impairment	0	Glutamate	0	Type A	0	Sinus headache	0	Regimen	0	Lactation	0	Abnormalities	0	Blood flow	0	Efficacy	0
0	Prophylaxis	0	Angiotensin	0	Incidence	0	Duration	0	Patterns	0	Anger	0	Central	0	Future	0	Joy	0	Quality of life	0	Aggravate	0	Motion sickness	0	Steroid	0	Calcium channel blocker	0	faith	0	Shock	0	Population	0	boss	0	vacation	0	Neck muscle	0	Trigger point	0	NSAID	0	Agonist	0	CAT scan	0	Weight loss	0	Testing	0	Epilepsy	0	Peer support	0	stigma	0	Acute migraine	0	Cervical spine	0	Characteristic	0	Sensitivity to sound	0	Peripheral	0	Upset	0	Anti-inflammatory	0	Facial pain	0	Mechanism	0	Grandmother	0	Homeopathy	0	surgery	0	Accident	0	regime	0	Trauma	0	Mindfulness	0	Tricyclic	0	Remission	0	Histamine	0	Headache diary	0	Anti-seizure	0	Occipital nerve	0	Headache clinic	0	Caregiver	0	impairment	0	Glutamate	0	Type A	0	Sinus headache	0	Regimen	0	Lactation	0	Abnormalities	0	Blood flow	0	Efficacy	0
0	Prophylaxis	0	Angiotensin	0	Incidence	0	Duration	0	Patterns	0	Anger	0	Central	0	Future	0	Joy	1	Quality of life	0	Aggravate	0	Motion sickness	0	Steroid	0	Calcium channel blocker	0	faith	0	Shock	0	Population	2	boss	0	vacation	0	Neck muscle	0	Trigger point	0	NSAID	0	Agonist	0	CAT scan	0	Weight loss	0	Testing	0	Epilepsy	0	Peer support	4	stigma	0	Acute migraine	0	Cervical spine	0	Characteristic	0	Sensitivity to sound	0	Peripheral	0	Upset	0	Anti-inflammatory	0	Facial pain	0	Mechanism	0	Grandmother	0	Homeopathy	0	surgery	0	Accident	0	regime	0	Trauma	2	Mindfulness	0	Tricyclic	0	Remission	0	Histamine	0	Headache diary	0	Anti-seizure	0	Occipital nerve	0	Headache clinic	0	Caregiver	0	impairment	0	Glutamate	0	Type A	0	Sinus headache	0	Regimen	0	Lactation	0	Abnormalities	0	Blood flow	0	Efficacy	0
0	Prophylaxis	0	Angiotensin	0	Incidence	0	Duration	0	Patterns	0	Anger	0	Central	1	Future	0	Joy	0	Quality of life	0	Aggravate	0	Motion sickness	0	Steroid	0	Calcium channel blocker	0	faith	0	Shock	0	Population	0	boss	2	vacation	0	Neck muscle	0	Trigger point	0	NSAID	0	Agonist	0	CAT scan	0	Weight loss	0	Testing	0	Epilepsy	0	Peer support	0	stigma	0	Acute migraine	0	Cervical spine	0	Characteristic	0	Sensitivity to sound	0	Peripheral	0	Upset	0	Anti-inflammatory	0	Facial pain	0	Mechanism	0	Grandmother	0	Homeopathy	0	surgery	0	Accident	0	regime	1	Trauma	0	Mindfulness	0	Tricyclic	0	Remission	0	Histamine	0	Headache diary	0	Anti-seizure	0	Occipital nerve	0	Headache clinic	0	Caregiver	0	impairment	0	Glutamate	0	Type A	0	Sinus headache	0	Regimen	1	Lactation	0	Abnormalities	0	Blood flow	0	Efficacy	0
0	Prophylaxis	0	Angiotensin	0	Incidence	0	Duration	0	Patterns	0	Anger	1	Central	1	Future	0	Joy	0	Quality of life	0	Aggravate	0	Motion sickness	0	Steroid	0	Calcium channel blocker	0	faith	0	Shock	0	Population	0	boss	0	vacation	0	Neck muscle	0	Trigger point	0	NSAID	0	Agonist	0	CAT scan	0	Weight loss	0	Testing	0	Epilepsy	0	Peer support	0	stigma	0	Acute migraine	2	Cervical spine	0	Characteristic	0	Sensitivity to sound	0	Peripheral	0	Upset	0	Anti-inflammatory	0	Facial pain	0	Mechanism	0	Grandmother	0	Homeopathy	0	surgery	0	Accident	0	regime	0	Trauma	0	Mindfulness	0	Tricyclic	0	Remission	0	Histamine	0	Headache diary	0	Anti-seizure	0	Occipital nerve	0	Headache clinic	0	Caregiver	0	impairment	0	Glutamate	0	Type A	0	Sinus headache	0	Regimen	0	Lactation	0	Abnormalities	0	Blood flow	0	Efficacy	0
0	Prophylaxis	0	Angiotensin	0	Incidence	0	Duration	0	Patterns	0	Anger	0	Central	1	Future	0	Joy	0	Quality of life	0	Aggravate	0	Motion sickness	0	Steroid	0	Calcium channel blocker	0	faith	0	Shock	0	Population	0	boss	0	vacation	0	Neck muscle	0	Trigger point	0	NSAID	0	Agonist	0	CAT scan	0	Weight loss	0	Testing	0	Epilepsy	0	Peer support	0	stigma	1	Acute migraine	0	Cervical spine	0	Characteristic	0	Sensitivity to sound	0	Peripheral	0	Upset	0	Anti-inflammatory	0	Facial pain	0	Mechanism	0	Grandmother	0	Homeopathy	0	surgery	0	Accident	0	regime	1	Trauma	0	Mindfulness	0	Tricyclic	0	Remission	0	Histamine	0	Headache diary	0	Anti-seizure	0	Occipital nerve	0	Headache clinic	0	Caregiver	0	impairment	0	Glutamate	0	Type A	0	Sinus headache	0	Regimen	1	Lactation	0	Abnormalities	0	Blood flow	0	Efficacy	0
0	Prophylaxis	0	Angiotensin	0	Incidence	0	Duration	0	Patterns	0	Anger	0	Central	0	Future	0	Joy	0	Quality of life	0	Aggravate	0	Motion sickness	0	Steroid	0	Calcium channel blocker	0	faith	0	Shock	0	Population	0	boss	0	vacation	0	Neck muscle	0	Trigger point	0	NSAID	0	Agonist	0	CAT scan	0	Weight loss	0	Testing	0	Epilepsy	0	Peer support	0	stigma	0	Acute migraine	0	Cervical spine	0	Characteristic	0	Sensitivity to sound	0	Peripheral	0	Upset	0	Anti-inflammatory	0	Facial pain	0	Mechanism	0	Grandmother	0	Homeopathy	0	surgery	0	Accident	0	regime	0	Trauma	0	Mindfulness	0	Tricyclic	0	Remission	0	Histamine	0	Headache diary	0	Anti-seizure	0	Occipital nerve	0	Headache clinic	0	Caregiver	0	impairment	0	Glutamate	0	Type A	0	Sinus headache	0	Regimen	0	Lactation	0	Abnormalities	0	Blood flow	0	Efficacy	0
0	Prophylaxis	0	Angiotensin	0	Incidence	0	Duration	0	Patterns	1	Anger	0	Central	0	Future	1	Joy	0	Quality of life	0	Aggravate	0	Motion sickness	0	Steroid	0	Calcium channel blocker	0	faith	0	Shock	0	Population	0	boss	0	vacation	0	Neck muscle	0	Trigger point	0	NSAID	0	Agonist	0	CAT scan	0	Weight loss	0	Testing	0	Epilepsy	1	Peer support	0	stigma	0	Acute migraine	2	Cervical spine	0	Characteristic	0	Sensitivity to sound	0	Peripheral	0	Upset	0	Anti-inflammatory	0	Facial pain	0	Mechanism	0	Grandmother	0	Homeopathy	0	surgery	0	Accident	1	regime	0	Trauma	0	Mindfulness	0	Tricyclic	0	Remission	0	Histamine	0	Headache diary	0	Anti-seizure	0	Occipital nerve	0	Headache clinic	0	Caregiver	0	impairment	0	Glutamate	0	Type A	0	Sinus headache	1	Regimen	0	Lactation	0	Abnormalities	0	Blood flow	0	Efficacy	0
0	Prophylaxis	0	Angiotensin	0	Incidence	0	Duration	0	Patterns	0	Anger	0	Central	0	Future	0	Joy	0	Quality of life	0	Aggravate	0	Motion sickness	0	Steroid	0	Calcium channel blocker	0	faith	0	Shock	1	Population	0	boss	0	vacation	0	Neck muscle	0	Trigger point	0	NSAID	0	Agonist	0	CAT scan	0	Weight loss	0	Testing	0	Epilepsy	0	Peer support	0	stigma	0	Acute migraine	0	Cervical spine	0	Characteristic	0	Sensitivity to sound	0	Peripheral	0	Upset	0	Anti-inflammatory	0	Facial pain	0	Mechanism	0	Grandmother	0	Homeopathy	0	surgery	0	Accident	0	regime	0	Trauma	0	Mindfulness	0	Tricyclic	0	Remission	0	Histamine	0	Headache diary	0	Anti-seizure	0	Occipital nerve	0	Headache clinic	0	Caregiver	0	impairment	0	Glutamate	0	Type A	0	Sinus headache	0	Regimen	0	Lactation	0	Abnormalities	0	Blood flow	0	Efficacy	0
0	Prophylaxis	0	Angiotensin	0	Incidence	0	Duration	0	Patterns	0	Anger	0	Central	0	Future	0	Joy	0	Quality of life	0	Aggravate	0	Motion sickness	0	Steroid	0	Calcium channel blocker	0	faith	0	Shock	0	Population	0	boss	0	vacation	0	Neck muscle	0	Trigger point	0	NSAID	0	Agonist	0	CAT scan	0	Weight loss	0	Testing	0	Epilepsy	0	Peer support	0	stigma	0	Acute migraine	0	Cervical spine	0	Characteristic	0	Sensitivity to sound	0	Peripheral	0	Upset	0	Anti-inflammatory	0	Facial pain	0	Mechanism	0	Grandmother	0	Homeopathy	0	surgery	0	Accident	0	regime	0	Trauma	0	Mindfulness	0	Tricyclic	0	Remission	0	Histamine	0	Headache diary	0	Anti-seizure	0	Occipital nerve	0	Headache clinic	0	Caregiver	0	impairment	0	Glutamate	0	Type A	1	Sinus headache	0	Regimen	0	Lactation	0	Abnormalities	0	Blood flow	0	Efficacy	0
0	Prophylaxis	0	Angiotensin	0	Incidence	0	Duration	0	Patterns	0	Anger	0	Central	0	Future	0	Joy	0	Quality of life	0	Aggravate	0	Motion sickness	0	Steroid	0	Calcium channel blocker	0	faith	0	Shock	0	Population	0	boss	0	vacation	0	Neck muscle	0	Trigger point	0	NSAID	0	Agonist	0	CAT scan	0	Weight loss	0	Testing	0	Epilepsy	0	Peer support	0	stigma	0	Acute migraine	0	Cervical spine	0	Characteristic	0	Sensitivity to sound	0	Peripheral	0	Upset	0	Anti-inflammatory	0	Facial pain	0	Mechanism	0	Grandmother	0	Homeopathy	0	surgery	0	Accident	0	regime	0	Trauma	0	Mindfulness	0	Tricyclic	0	Remission	0	Histamine	0	Headache diary	0	Anti-seizure	0	Occipital nerve	0	Headache clinic	0	Caregiver	0	impairment	0	Glutamate	0	Type A	0	Sinus headache	0	Regimen	0	Lactation	0	Abnormalities	0	Blood flow	0	Efficacy	0
0	Prophylaxis	0	Angiotensin	0	Incidence	0	Duration	0	Patterns	0	Anger	0	Central	0	Future	0	Joy	0	Quality of life	0	Aggravate	0	Motion sickness	0	Steroid	0	Calcium channel blocker	0	faith	0	Shock	0	Population	0	boss	0	vacation	0	Neck muscle	0	Trigger point	0	NSAID	0	Agonist	0	CAT scan	0	Weight loss	0	Testing	0	Epilepsy	0	Peer support	0	stigma	0	Acute migraine	0	Cervical spine	0	Characteristic	0	Sensitivity to sound	0	Peripheral	0	Upset	0	Anti-inflammatory	0	Facial pain	0	Mechanism	0	Grandmother	0	Homeopathy	1	surgery	1	Accident	0	regime	0	Trauma	0	Mindfulness	0	Tricyclic	0	Remission	0	Histamine	0	Headache diary	0	Anti-seizure	0	Occipital nerve	0	Headache clinic	0	Caregiver	0	impairment	0	Glutamate	0	Type A	0	Sinus headache	0	Regimen	0	Lactation	0	Abnormalities	0	Blood flow	0	Efficacy	0
0	Prophylaxis	0	Angiotensin	0	Incidence	0	Duration	0	Patterns	0	Anger	0	Central	0	Future	0	Joy	0	Quality of life	0	Aggravate	0	Motion sickness	0	Steroid	0	Calcium channel blocker	0	faith	0	Shock	0	Population	0	boss	0	vacation	0	Neck muscle	0	Trigger point	0	NSAID	0	Agonist	0	CAT scan	0	Weight loss	0	Testing	0	Epilepsy	0	Peer support	0	stigma	0	Acute migraine	0	Cervical spine	0	Characteristic	0	Sensitivity to sound	0	Peripheral	0	Upset	0	Anti-inflammatory	0	Facial pain	0	Mechanism	0	Grandmother	0	Homeopathy	0	surgery	0	Accident	0	regime	0	Trauma	0	Mindfulness	0	Tricyclic	0	Remission	0	Histamine	0	Headache diary	0	Anti-seizure	0	Occipital nerve	0	Headache clinic	0	Caregiver	0	impairment	0	Glutamate	0	Type A	0	Sinus headache	0	Regimen	0	Lactation	0	Abnormalities	0	Blood flow	0	Efficacy	0
0	Prophylaxis	0	Angiotensin	0	Incidence	0	Duration	0	Patterns	0	Anger	0	Central	0	Future	0	Joy	0	Quality of life	0	Aggravate	0	Motion sickness	0	Steroid	0	Calcium channel blocker	0	faith	0	Shock	0	Population	0	boss	0	vacation	0	Neck muscle	0	Trigger point	0	NSAID	0	Agonist	0	CAT scan	0	Weight loss	0	Testing	0	Epilepsy	0	Peer support	0	stigma	0	Acute migraine	0	Cervical spine	0	Characteristic	0	Sensitivity to sound	0	Peripheral	0	Upset	0	Anti-inflammatory	0	Facial pain	0	Mechanism	1	Grandmother	3	Homeopathy	0	surgery	0	Accident	0	regime	0	Trauma	0	Mindfulness	0	Tricyclic	0	Remission	0	Histamine	0	Headache diary	0	Anti-seizure	0	Occipital nerve	0	Headache clinic	0	Caregiver	0	impairment	3	Glutamate	1	Type A	0	Sinus headache	0	Regimen	0	Lactation	0	Abnormalities	0	Blood flow	0	Efficacy	0
0	Prophylaxis	0	Angiotensin	0	Incidence	0	Duration	0	Patterns	0	Anger	0	Central	0	Future	2	Joy	0	Quality of life	0	Aggravate	0	Motion sickness	0	Steroid	0	Calcium channel blocker	0	faith	0	Shock	0	Population	0	boss	0	vacation	0	Neck muscle	0	Trigger point	0	NSAID	0	Agonist	0	CAT scan	0	Weight loss	1	Testing	2	Epilepsy	0	Peer support	0	stigma	0	Acute migraine	0	Cervical spine	0	Characteristic	0	Sensitivity to sound	0	Peripheral	0	Upset	0	Anti-inflammatory	0	Facial pain	0	Mechanism	0	Grandmother	0	Homeopathy	0	surgery	0	Accident	0	regime	0	Trauma	0	Mindfulness	0	Tricyclic	0	Remission	0	Histamine	0	Headache diary	0	Anti-seizure	0	Occipital nerve	0	Headache clinic	0	Caregiver	0	impairment	0	Glutamate	0	Type A	0	Sinus headache	0	Regimen	0	Lactation	0	Abnormalities	0	Blood flow	0	Efficacy	0
0	Prophylaxis	0	Angiotensin	0	Incidence	0	Duration	0	Patterns	0	Anger	0	Central	0	Future	0	Joy	0	Quality of life	1	Aggravate	0	Motion sickness	0	Steroid	0	Calcium channel blocker	0	faith	0	Shock	0	Population	0	boss	0	vacation	0	Neck muscle	0	Trigger point	0	NSAID	0	Agonist	0	CAT scan	0	Weight loss	0	Testing	0	Epilepsy	0	Peer support	0	stigma	0	Acute migraine	0	Cervical spine	0	Characteristic	0	Sensitivity to sound	0	Peripheral	0	Upset	0	Anti-inflammatory	0	Facial pain	0	Mechanism	0	Grandmother	0	Homeopathy	0	surgery	0	Accident	0	regime	0	Trauma	1	Mindfulness	0	Tricyclic	0	Remission	0	Histamine	0	Headache diary	0	Anti-seizure	0	Occipital nerve	0	Headache clinic	0	Caregiver	1	impairment	0	Glutamate	0	Type A	0	Sinus headache	0	Regimen	0	Lactation	0	Abnormalities	0	Blood flow	0	Efficacy	0
0	Prophylaxis	0	Angiotensin	0	Incidence	0	Duration	0	Patterns	0	Anger	0	Central	0	Future	0	Joy	0	Quality of life	0	Aggravate	0	Motion sickness	0	Steroid	0	Calcium channel blocker	0	faith	0	Shock	0	Population	0	boss	0	vacation	0	Neck muscle	0	Trigger point	0	NSAID	0	Agonist	0	CAT scan	0	Weight loss	0	Testing	0	Epilepsy	0	Peer support	0	stigma	0	Acute migraine	0	Cervical spine	0	Characteristic	0	Sensitivity to sound	0	Peripheral	0	Upset	0	Anti-inflammatory	0	Facial pain	0	Mechanism	0	Grandmother	0	Homeopathy	0	surgery	0	Accident	0	regime	0	Trauma	0	Mindfulness	0	Tricyclic	0	Remission	0	Histamine	0	Headache diary	0	Anti-seizure	0	Occipital nerve	0	Headache clinic	0	Caregiver	2	impairment	0	Glutamate	0	Type A	0	Sinus headache	0	Regimen	0	Lactation	0	Abnormalities	0	Blood flow	0	Efficacy	0
0	Prophylaxis	0	Angiotensin	0	Incidence	0	Duration	0	Patterns	0	Anger	0	Central	0	Future	0	Joy	0	Quality of life	0	Aggravate	0	Motion sickness	0	Steroid	0	Calcium channel blocker	0	faith	0	Shock	0	Population	0	boss	0	vacation	0	Neck muscle	0	Trigger point	0	NSAID	0	Agonist	0	CAT scan	0	Weight loss	0	Testing	0	Epilepsy	0	Peer support	0	stigma	0	Acute migraine	0	Cervical spine	0	Characteristic	0	Sensitivity to sound	0	Peripheral	0	Upset	0	Anti-inflammatory	0	Facial pain	0	Mechanism	0	Grandmother	0	Homeopathy	0	surgery	0	Accident	1	regime	0	Trauma	0	Mindfulness	0	Tricyclic	0	Remission	0	Histamine	0	Headache diary	0	Anti-seizure	0	Occipital nerve	0	Headache clinic	0	Caregiver	0	impairment	0	Glutamate	0	Type A	0	Sinus headache	0	Regimen	0	Lactation	0	Abnormalities	0	Blood flow	0	Efficacy	0
0	Prophylaxis	0	Angiotensin	0	Incidence	0	Duration	0	Patterns	0	Anger	0	Central	0	Future	0	Joy	0	Quality of life	0	Aggravate	0	Motion sickness	0	Steroid	0	Calcium channel blocker	0	faith	0	Shock	0	Population	0	boss	0	vacation	0	Neck muscle	0	Trigger point	0	NSAID	0	Agonist	0	CAT scan	0	Weight loss	0	Testing	0	Epilepsy	0	Peer support	0	stigma	0	Acute migraine	0	Cervical spine	0	Characteristic	0	Sensitivity to sound	0	Peripheral	0	Upset	0	Anti-inflammatory	0	Facial pain	0	Mechanism	0	Grandmother	0	Homeopathy	0	surgery	0	Accident	0	regime	0	Trauma	0	Mindfulness	0	Tricyclic	0	Remission	0	Histamine	0	Headache diary	0	Anti-seizure	0	Occipital nerve	0	Headache clinic	0	Caregiver	0	impairment	0	Glutamate	0	Type A	0	Sinus headache	0	Regimen	0	Lactation	0	Abnormalities	0	Blood flow	0	Efficacy	0
0	Prophylaxis	0	Angiotensin	0	Incidence	0	Duration	0	Patterns	0	Anger	0	Central	0	Future	0	Joy	0	Quality of life	0	Aggravate	0	Motion sickness	0	Steroid	0	Calcium channel blocker	0	faith	0	Shock	0	Population	0	boss	0	vacation	0	Neck muscle	0	Trigger point	0	NSAID	0	Agonist	0	CAT scan	0	Weight loss	0	Testing	0	Epilepsy	0	Peer support	0	stigma	0	Acute migraine	0	Cervical spine	0	Characteristic	0	Sensitivity to sound	0	Peripheral	0	Upset	0	Anti-inflammatory	0	Facial pain	0	Mechanism	0	Grandmother	0	Homeopathy	0	surgery	0	Accident	0	regime	0	Trauma	0	Mindfulness	0	Tricyclic	0	Remission	0	Histamine	0	Headache diary	0	Anti-seizure	0	Occipital nerve	0	Headache clinic	0	Caregiver	0	impairment	0	Glutamate	0	Type A	0	Sinus headache	0	Regimen	0	Lactation	0	Abnormalities	0	Blood flow	0	Efficacy	0
0	Prophylaxis	0	Angiotensin	0	Incidence	0	Duration	0	Patterns	0	Anger	0	Central	0	Future	0	Joy	0	Quality of life	0	Aggravate	0	Motion sickness	0	Steroid	0	Calcium channel blocker	0	faith	0	Shock	0	Population	0	boss	0	vacation	0	Neck muscle	0	Trigger point	0	NSAID	0	Agonist	0	CAT scan	0	Weight loss	0	Testing	0	Epilepsy	0	Peer support	0	stigma	0	Acute migraine	0	Cervical spine	0	Characteristic	0	Sensitivity to sound	0	Peripheral	0	Upset	0	Anti-inflammatory	0	Facial pain	0	Mechanism	0	Grandmother	0	Homeopathy	0	surgery	0	Accident	0	regime	0	Trauma	0	Mindfulness	0	Tricyclic	0	Remission	0	Histamine	0	Headache diary	0	Anti-seizure	0	Occipital nerve	0	Headache clinic	0	Caregiver	0	impairment	0	Glutamate	0	Type A	0	Sinus headache	0	Regimen	0	Lactation	0	Abnormalities	0	Blood flow	0	Efficacy	0
0	Prophylaxis	0	Angiotensin	0	Incidence	0	Duration	0	Patterns	0	Anger	0	Central	0	Future	0	Joy	0	Quality of life	0	Aggravate	0	Motion sickness	0	Steroid	0	Calcium channel blocker	0	faith	0	Shock	0	Population	0	boss	0	vacation	0	Neck muscle	0	Trigger point	0	NSAID	0	Agonist	0	CAT scan	0	Weight loss	0	Testing	0	Epilepsy	0	Peer support	0	stigma	0	Acute migraine	0	Cervical spine	0	Characteristic	0	Sensitivity to sound	0	Peripheral	0	Upset	0	Anti-inflammatory	0	Facial pain	0	Mechanism	0	Grandmother	0	Homeopathy	0	surgery	0	Accident	0	regime	0	Trauma	0	Mindfulness	0	Tricyclic	0	Remission	0	Histamine	0	Headache diary	0	Anti-seizure	0	Occipital nerve	0	Headache clinic	0	Caregiver	0	impairment	0	Glutamate	0	Type A	0	Sinus headache	0	Regimen	0	Lactation	0	Abnormalities	0	Blood flow	0	Efficacy	0
0	Prophylaxis	0	Angiotensin	0	Incidence	0	Duration	0	Patterns	0	Anger	0	Central	0	Future	0	Joy	0	Quality of life	0	Aggravate	0	Motion sickness	0	Steroid	0	Calcium channel blocker	0	faith	0	Shock	0	Population	0	boss	0	vacation	0	Neck muscle	0	Trigger point	0	NSAID	0	Agonist	0	CAT scan	0	Weight loss	0	Testing	0	Epilepsy	0	Peer support	0	stigma	0	Acute migraine	0	Cervical spine	0	Characteristic	0	Sensitivity to sound	0	Peripheral	0	Upset	0	Anti-inflammatory	0	Facial pain	0	Mechanism	0	Grandmother	0	Homeopathy	0	surgery	0	Accident	0	regime	0	Trauma	0	Mindfulness	1	Tricyclic	0	Remission	0	Histamine	0	Headache diary	0	Anti-seizure	0	Occipital nerve	0	Headache clinic	0	Caregiver	0	impairment	0	Glutamate	0	Type A	0	Sinus headache	0	Regimen	0	Lactation	0	Abnormalities	0	Blood flow	0	Efficacy	0
0	Prophylaxis	0	Angiotensin	0	Incidence	0	Duration	1	Patterns	0	Anger	0	Central	0	Future	0	Joy	0	Quality of life	0	Aggravate	0	Motion sickness	0	Steroid	0	Calcium channel blocker	0	faith	0	Shock	0	Population	0	boss	0	vacation	0	Neck muscle	0	Trigger point	0	NSAID	0	Agonist	0	CAT scan	0	Weight loss	0	Testing	0	Epilepsy	0	Peer support	0	stigma	0	Acute migraine	0	Cervical spine	0	Characteristic	0	Sensitivity to sound	0	Peripheral	0	Upset	0	Anti-inflammatory	0	Facial pain	0	Mechanism	0	Grandmother	0	Homeopathy	0	surgery	0	Accident	0	regime	0	Trauma	0	Mindfulness	0	Tricyclic	0	Remission	0	Histamine	0	Headache diary	0	Anti-seizure	0	Occipital nerve	0	Headache clinic	0	Caregiver	0	impairment	0	Glutamate	0	Type A	0	Sinus headache	0	Regimen	0	Lactation	0	Abnormalities	0	Blood flow	0	Efficacy	0
0	Prophylaxis	0	Angiotensin	0	Incidence	0	Duration	0	Patterns	0	Anger	0	Central	0	Future	0	Joy	0	Quality of life	0	Aggravate	0	Motion sickness	0	Steroid	0	Calcium channel blocker	2	faith	1	Shock	0	Population	0	boss	0	vacation	0	Neck muscle	0	Trigger point	0	NSAID	0	Agonist	0	CAT scan	0	Weight loss	0	Testing	0	Epilepsy	0	Peer support	0	stigma	0	Acute migraine	0	Cervical spine	0	Characteristic	0	Sensitivity to sound	0	Peripheral	0	Upset	0	Anti-inflammatory	0	Facial pain	3	Mechanism	0	Grandmother	0	Homeopathy	0	surgery	0	Accident	0	regime	0	Trauma	0	Mindfulness	0	Tricyclic	0	Remission	0	Histamine	0	Headache diary	0	Anti-seizure	0	Occipital nerve	0	Headache clinic	0	Caregiver	0	impairment	0	Glutamate	0	Type A	0	Sinus headache	0	Regimen	0	Lactation	0	Abnormalities	0	Blood flow	0	Efficacy	0
0	Prophylaxis	0	Angiotensin	0	Incidence	0	Duration	0	Patterns	0	Anger	0	Central	0	Future	0	Joy	0	Quality of life	0	Aggravate	0	Motion sickness	0	Steroid	0	Calcium channel blocker	0	faith	0	Shock	0	Population	0	boss	0	vacation	0	Neck muscle	0	Trigger point	0	NSAID	0	Agonist	0	CAT scan	0	Weight loss	0	Testing	0	Epilepsy	0	Peer support	0	stigma	0	Acute migraine	0	Cervical spine	0	Characteristic	0	Sensitivity to sound	0	Peripheral	0	Upset	0	Anti-inflammatory	0	Facial pain	0	Mechanism	0	Grandmother	0	Homeopathy	0	surgery	0	Accident	0	regime	0	Trauma	0	Mindfulness	0	Tricyclic	0	Remission	0	Histamine	0	Headache diary	0	Anti-seizure	0	Occipital nerve	0	Headache clinic	1	Caregiver	0	impairment	0	Glutamate	0	Type A	0	Sinus headache	0	Regimen	0	Lactation	0	Abnormalities	0	Blood flow	0	Efficacy	0
0	Prophylaxis	0	Angiotensin	0	Incidence	0	Duration	0	Patterns	0	Anger	0	Central	0	Future	0	Joy	0	Quality of life	0	Aggravate	0	Motion sickness	0	Steroid	1	Calcium channel blocker	0	faith	0	Shock	0	Population	0	boss	0	vacation	0	Neck muscle	0	Trigger point	0	NSAID	0	Agonist	0	CAT scan	0	Weight loss	0	Testing	0	Epilepsy	0	Peer support	0	stigma	0	Acute migraine	0	Cervical spine	0	Characteristic	0	Sensitivity to sound	0	Peripheral	0	Upset	0	Anti-inflammatory	1	Facial pain	0	Mechanism	0	Grandmother	0	Homeopathy	0	surgery	0	Accident	0	regime	0	Trauma	0	Mindfulness	0	Tricyclic	0	Remission	0	Histamine	0	Headache diary	0	Anti-seizure	0	Occipital nerve	0	Headache clinic	0	Caregiver	0	impairment	0	Glutamate	0	Type A	0	Sinus headache	0	Regimen	0	Lactation	0	Abnormalities	0	Blood flow	0	Efficacy	0
0	Prophylaxis	0	Angiotensin	0	Incidence	0	Duration	0	Patterns	0	Anger	0	Central	0	Future	0	Joy	0	Quality of life	0	Aggravate	0	Motion sickness	0	Steroid	0	Calcium channel blocker	0	faith	0	Shock	0	Population	0	boss	0	vacation	0	Neck muscle	0	Trigger point	0	NSAID	0	Agonist	0	CAT scan	0	Weight loss	0	Testing	0	Epilepsy	0	Peer support	0	stigma	0	Acute migraine	0	Cervical spine	0	Characteristic	0	Sensitivity to sound	0	Peripheral	0	Upset	0	Anti-inflammatory	0	Facial pain	0	Mechanism	0	Grandmother	0	Homeopathy	0	surgery	0	Accident	0	regime	0	Trauma	0	Mindfulness	0	Tricyclic	0	Remission	0	Histamine	0	Headache diary	0	Anti-seizure	0	Occipital nerve	0	Headache clinic	0	Caregiver	0	impairment	0	Glutamate	0	Type A	0	Sinus headache	0	Regimen	0	Lactation	0	Abnormalities	0	Blood flow	0	Efficacy	0
1	Prophylaxis	0	Angiotensin	0	Incidence	2	Duration	0	Patterns	1	Anger	0	Central	0	Future	0	Joy	0	Quality of life	0	Aggravate	0	Motion sickness	0	Steroid	0	Calcium channel blocker	0	faith	0	Shock	0	Population	0	boss	0	vacation	0	Neck muscle	0	Trigger point	0	NSAID	0	Agonist	0	CAT scan	1	Weight loss	0	Testing	0	Epilepsy	0	Peer support	0	stigma	0	Acute migraine	0	Cervical spine	0	Characteristic	0	Sensitivity to sound	0	Peripheral	1	Upset	1	Anti-inflammatory	0	Facial pain	0	Mechanism	0	Grandmother	0	Homeopathy	0	surgery	0	Accident	0	regime	0	Trauma	0	Mindfulness	0	Tricyclic	0	Remission	0	Histamine	0	Headache diary	0	Anti-seizure	0	Occipital nerve	0	Headache clinic	0	Caregiver	0	impairment	0	Glutamate	0	Type A	0	Sinus headache	0	Regimen	0	Lactation	0	Abnormalities	0	Blood flow	0	Efficacy	0
0	Prophylaxis	0	Angiotensin	0	Incidence	0	Duration	0	Patterns	0	Anger	1	Central	0	Future	0	Joy	0	Quality of life	0	Aggravate	0	Motion sickness	1	Steroid	1	Calcium channel blocker	0	faith	0	Shock	0	Population	0	boss	0	vacation	0	Neck muscle	0	Trigger point	0	NSAID	0	Agonist	0	CAT scan	0	Weight loss	0	Testing	0	Epilepsy	0	Peer support	0	stigma	0	Acute migraine	0	Cervical spine	0	Characteristic	0	Sensitivity to sound	0	Peripheral	0	Upset	0	Anti-inflammatory	0	Facial pain	0	Mechanism	0	Grandmother	0	Homeopathy	0	surgery	1	Accident	0	regime	0	Trauma	0	Mindfulness	0	Tricyclic	0	Remission	0	Histamine	0	Headache diary	0	Anti-seizure	0	Occipital nerve	0	Headache clinic	0	Caregiver	0	impairment	0	Glutamate	0	Type A	0	Sinus headache	0	Regimen	0	Lactation	0	Abnormalities	0	Blood flow	0	Efficacy	0
0	Prophylaxis	0	Angiotensin	0	Incidence	0	Duration	0	Patterns	0	Anger	0	Central	0	Future	0	Joy	0	Quality of life	0	Aggravate	0	Motion sickness	0	Steroid	0	Calcium channel blocker	0	faith	0	Shock	0	Population	0	boss	0	vacation	0	Neck muscle	0	Trigger point	0	NSAID	0	Agonist	0	CAT scan	0	Weight loss	0	Testing	0	Epilepsy	0	Peer support	0	stigma	0	Acute migraine	0	Cervical spine	0	Characteristic	0	Sensitivity to sound	0	Peripheral	0	Upset	0	Anti-inflammatory	0	Facial pain	0	Mechanism	0	Grandmother	0	Homeopathy	0	surgery	0	Accident	0	regime	0	Trauma	0	Mindfulness	0	Tricyclic	0	Remission	0	Histamine	0	Headache diary	0	Anti-seizure	0	Occipital nerve	0	Headache clinic	0	Caregiver	0	impairment	0	Glutamate	0	Type A	0	Sinus headache	0	Regimen	0	Lactation	0	Abnormalities	0	Blood flow	0	Efficacy	0
0	Prophylaxis	0	Angiotensin	0	Incidence	0	Duration	0	Patterns	0	Anger	1	Central	0	Future	0	Joy	0	Quality of life	0	Aggravate	0	Motion sickness	0	Steroid	0	Calcium channel blocker	0	faith	0	Shock	0	Population	0	boss	0	vacation	2	Neck muscle	0	Trigger point	0	NSAID	0	Agonist	0	CAT scan	0	Weight loss	0	Testing	0	Epilepsy	0	Peer support	0	stigma	0	Acute migraine	0	Cervical spine	0	Characteristic	0	Sensitivity to sound	0	Peripheral	0	Upset	0	Anti-inflammatory	0	Facial pain	0	Mechanism	0	Grandmother	0	Homeopathy	0	surgery	0	Accident	1	regime	0	Trauma	0	Mindfulness	0	Tricyclic	0	Remission	0	Histamine	0	Headache diary	0	Anti-seizure	0	Occipital nerve	0	Headache clinic	0	Caregiver	0	impairment	0	Glutamate	0	Type A	0	Sinus headache	0	Regimen	0	Lactation	0	Abnormalities	0	Blood flow	0	Efficacy	0
0	Prophylaxis	0	Angiotensin	0	Incidence	0	Duration	0	Patterns	0	Anger	0	Central	0	Future	0	Joy	0	Quality of life	0	Aggravate	0	Motion sickness	0	Steroid	0	Calcium channel blocker	0	faith	0	Shock	0	Population	0	boss	0	vacation	0	Neck muscle	0	Trigger point	0	NSAID	0	Agonist	0	CAT scan	0	Weight loss	0	Testing	0	Epilepsy	0	Peer support	0	stigma	0	Acute migraine	0	Cervical spine	0	Characteristic	0	Sensitivity to sound	0	Peripheral	0	Upset	0	Anti-inflammatory	0	Facial pain	0	Mechanism	0	Grandmother	0	Homeopathy	0	surgery	0	Accident	0	regime	0	Trauma	0	Mindfulness	0	Tricyclic	0	Remission	0	Histamine	0	Headache diary	0	Anti-seizure	0	Occipital nerve	0	Headache clinic	0	Caregiver	0	impairment	0	Glutamate	0	Type A	0	Sinus headache	0	Regimen	0	Lactation	0	Abnormalities	0	Blood flow	0	Efficacy	0
0	Prophylaxis	0	Angiotensin	0	Incidence	0	Duration	0	Patterns	1	Anger	0	Central	0	Future	0	Joy	0	Quality of life	0	Aggravate	0	Motion sickness	0	Steroid	0	Calcium channel blocker	0	faith	0	Shock	0	Population	1	boss	0	vacation	0	Neck muscle	0	Trigger point	0	NSAID	0	Agonist	0	CAT scan	0	Weight loss	0	Testing	0	Epilepsy	0	Peer support	0	stigma	0	Acute migraine	0	Cervical spine	0	Characteristic	0	Sensitivity to sound	0	Peripheral	0	Upset	0	Anti-inflammatory	0	Facial pain	0	Mechanism	0	Grandmother	0	Homeopathy	0	surgery	0	Accident	0	regime	0	Trauma	0	Mindfulness	0	Tricyclic	0	Remission	0	Histamine	0	Headache diary	0	Anti-seizure	0	Occipital nerve	0	Headache clinic	0	Caregiver	0	impairment	0	Glutamate	0	Type A	0	Sinus headache	0	Regimen	0	Lactation	0	Abnormalities	0	Blood flow	0	Efficacy	0
4	Prophylaxis	0	Angiotensin	0	Incidence	4	Duration	3	Patterns	2	Anger	0	Central	0	Future	0	Joy	4	Quality of life	1	Aggravate	1	Motion sickness	0	Steroid	4	Calcium channel blocker	1	faith	5	Shock	4	Population	0	boss	0	vacation	2	Neck muscle	3	Trigger point	5	NSAID	1	Agonist	1	CAT scan	0	Weight loss	2	Testing	1	Epilepsy	4	Peer support	0	stigma	3	Acute migraine	0	Cervical spine	0	Characteristic	0	Sensitivity to sound	3	Peripheral	0	Upset	1	Anti-inflammatory	2	Facial pain	0	Mechanism	1	Grandmother	1	Homeopathy	0	surgery	1	Accident	1	regime	1	Trauma	0	Mindfulness	0	Tricyclic	0	Remission	0	Histamine	0	Headache diary	1	Anti-seizure	2	Occipital nerve	3	Headache clinic	0	Caregiver	0	impairment	0	Glutamate	2	Type A	0	Sinus headache	1	Regimen	1	Lactation	0	Abnormalities	0	Blood flow	0	Efficacy	0
0	Prophylaxis	0	Angiotensin	0	Incidence	0	Duration	0	Patterns	0	Anger	0	Central	0	Future	0	Joy	0	Quality of life	0	Aggravate	0	Motion sickness	0	Steroid	0	Calcium channel blocker	0	faith	0	Shock	0	Population	0	boss	1	vacation	0	Neck muscle	0	Trigger point	0	NSAID	0	Agonist	0	CAT scan	0	Weight loss	0	Testing	0	Epilepsy	0	Peer support	0	stigma	0	Acute migraine	0	Cervical spine	0	Characteristic	0	Sensitivity to sound	0	Peripheral	1	Upset	0	Anti-inflammatory	0	Facial pain	0	Mechanism	0	Grandmother	0	Homeopathy	0	surgery	0	Accident	0	regime	0	Trauma	0	Mindfulness	0	Tricyclic	0	Remission	0	Histamine	0	Headache diary	0	Anti-seizure	0	Occipital nerve	0	Headache clinic	0	Caregiver	0	impairment	0	Glutamate	0	Type A	0	Sinus headache	0	Regimen	0	Lactation	0	Abnormalities	0	Blood flow	0	Efficacy	0
0	Prophylaxis	0	Angiotensin	0	Incidence	0	Duration	0	Patterns	0	Anger	0	Central	0	Future	0	Joy	0	Quality of life	0	Aggravate	0	Motion sickness	0	Steroid	0	Calcium channel blocker	1	faith	0	Shock	0	Population	0	boss	0	vacation	0	Neck muscle	0	Trigger point	0	NSAID	0	Agonist	0	CAT scan	0	Weight loss	0	Testing	0	Epilepsy	0	Peer support	0	stigma	0	Acute migraine	0	Cervical spine	0	Characteristic	0	Sensitivity to sound	0	Peripheral	0	Upset	0	Anti-inflammatory	0	Facial pain	0	Mechanism	0	Grandmother	0	Homeopathy	0	surgery	0	Accident	0	regime	0	Trauma	0	Mindfulness	0	Tricyclic	0	Remission	0	Histamine	1	Headache diary	0	Anti-seizure	0	Occipital nerve	0	Headache clinic	0	Caregiver	0	impairment	0	Glutamate	0	Type A	0	Sinus headache	0	Regimen	0	Lactation	0	Abnormalities	0	Blood flow	0	Efficacy	0
0	Prophylaxis	0	Angiotensin	0	Incidence	0	Duration	0	Patterns	0	Anger	0	Central	0	Future	0	Joy	0	Quality of life	0	Aggravate	0	Motion sickness	0	Steroid	0	Calcium channel blocker	0	faith	0	Shock	0	Population	0	boss	0	vacation	0	Neck muscle	0	Trigger point	0	NSAID	0	Agonist	0	CAT scan	0	Weight loss	0	Testing	0	Epilepsy	0	Peer support	0	stigma	0	Acute migraine	0	Cervical spine	0	Characteristic	0	Sensitivity to sound	0	Peripheral	0	Upset	0	Anti-inflammatory	0	Facial pain	0	Mechanism	0	Grandmother	0	Homeopathy	0	surgery	0	Accident	0	regime	0	Trauma	0	Mindfulness	0	Tricyclic	0	Remission	0	Histamine	0	Headache diary	0	Anti-seizure	0	Occipital nerve	0	Headache clinic	0	Caregiver	0	impairment	0	Glutamate	0	Type A	0	Sinus headache	0	Regimen	0	Lactation	0	Abnormalities	0	Blood flow	0	Efficacy	0
0	Prophylaxis	0	Angiotensin	0	Incidence	0	Duration	0	Patterns	0	Anger	0	Central	0	Future	1	Joy	0	Quality of life	0	Aggravate	1	Motion sickness	0	Steroid	0	Calcium channel blocker	0	faith	0	Shock	0	Population	0	boss	0	vacation	0	Neck muscle	0	Trigger point	0	NSAID	0	Agonist	0	CAT scan	1	Weight loss	0	Testing	0	Epilepsy	0	Peer support	0	stigma	0	Acute migraine	0	Cervical spine	0	Characteristic	0	Sensitivity to sound	0	Peripheral	0	Upset	0	Anti-inflammatory	1	Facial pain	0	Mechanism	0	Grandmother	0	Homeopathy	0	surgery	0	Accident	0	regime	0	Trauma	0	Mindfulness	0	Tricyclic	0	Remission	0	Histamine	0	Headache diary	0	Anti-seizure	0	Occipital nerve	0	Headache clinic	0	Caregiver	0	impairment	0	Glutamate	0	Type A	0	Sinus headache	0	Regimen	0	Lactation	0	Abnormalities	0	Blood flow	0	Efficacy	0
0	Prophylaxis	0	Angiotensin	0	Incidence	0	Duration	0	Patterns	0	Anger	0	Central	0	Future	0	Joy	0	Quality of life	0	Aggravate	0	Motion sickness	0	Steroid	0	Calcium channel blocker	0	faith	0	Shock	0	Population	0	boss	0	vacation	0	Neck muscle	0	Trigger point	0	NSAID	0	Agonist	0	CAT scan	0	Weight loss	0	Testing	0	Epilepsy	0	Peer support	0	stigma	0	Acute migraine	0	Cervical spine	0	Characteristic	0	Sensitivity to sound	0	Peripheral	0	Upset	0	Anti-inflammatory	0	Facial pain	0	Mechanism	0	Grandmother	0	Homeopathy	0	surgery	0	Accident	0	regime	0	Trauma	0	Mindfulness	0	Tricyclic	0	Remission	0	Histamine	0	Headache diary	0	Anti-seizure	0	Occipital nerve	0	Headache clinic	0	Caregiver	0	impairment	0	Glutamate	0	Type A	0	Sinus headache	0	Regimen	0	Lactation	0	Abnormalities	0	Blood flow	0	Efficacy	0
0	Prophylaxis	0	Angiotensin	0	Incidence	0	Duration	0	Patterns	0	Anger	0	Central	0	Future	1	Joy	0	Quality of life	3	Aggravate	0	Motion sickness	0	Steroid	0	Calcium channel blocker	0	faith	0	Shock	0	Population	0	boss	0	vacation	0	Neck muscle	0	Trigger point	0	NSAID	0	Agonist	0	CAT scan	0	Weight loss	0	Testing	0	Epilepsy	0	Peer support	1	stigma	0	Acute migraine	0	Cervical spine	0	Characteristic	0	Sensitivity to sound	0	Peripheral	0	Upset	0	Anti-inflammatory	0	Facial pain	0	Mechanism	0	Grandmother	0	Homeopathy	0	surgery	0	Accident	0	regime	0	Trauma	0	Mindfulness	1	Tricyclic	0	Remission	1	Histamine	0	Headache diary	0	Anti-seizure	0	Occipital nerve	0	Headache clinic	0	Caregiver	0	impairment	0	Glutamate	0	Type A	0	Sinus headache	0	Regimen	0	Lactation	0	Abnormalities	0	Blood flow	0	Efficacy	0
0	Prophylaxis	0	Angiotensin	0	Incidence	0	Duration	0	Patterns	0	Anger	0	Central	0	Future	0	Joy	0	Quality of life	0	Aggravate	0	Motion sickness	0	Steroid	0	Calcium channel blocker	0	faith	0	Shock	0	Population	0	boss	0	vacation	0	Neck muscle	0	Trigger point	0	NSAID	0	Agonist	0	CAT scan	0	Weight loss	0	Testing	0	Epilepsy	0	Peer support	0	stigma	0	Acute migraine	0	Cervical spine	0	Characteristic	0	Sensitivity to sound	0	Peripheral	0	Upset	0	Anti-inflammatory	0	Facial pain	0	Mechanism	0	Grandmother	0	Homeopathy	0	surgery	0	Accident	0	regime	0	Trauma	0	Mindfulness	0	Tricyclic	0	Remission	0	Histamine	0	Headache diary	0	Anti-seizure	0	Occipital nerve	0	Headache clinic	0	Caregiver	0	impairment	0	Glutamate	0	Type A	0	Sinus headache	0	Regimen	0	Lactation	0	Abnormalities	0	Blood flow	0	Efficacy	0
0	Prophylaxis	0	Angiotensin	0	Incidence	0	Duration	0	Patterns	0	Anger	0	Central	0	Future	0	Joy	0	Quality of life	0	Aggravate	0	Motion sickness	0	Steroid	0	Calcium channel blocker	0	faith	0	Shock	0	Population	0	boss	0	vacation	0	Neck muscle	0	Trigger point	0	NSAID	1	Agonist	0	CAT scan	0	Weight loss	0	Testing	0	Epilepsy	0	Peer support	0	stigma	0	Acute migraine	0	Cervical spine	0	Characteristic	0	Sensitivity to sound	0	Peripheral	0	Upset	0	Anti-inflammatory	0	Facial pain	0	Mechanism	0	Grandmother	0	Homeopathy	0	surgery	0	Accident	0	regime	0	Trauma	0	Mindfulness	0	Tricyclic	0	Remission	0	Histamine	0	Headache diary	0	Anti-seizure	0	Occipital nerve	0	Headache clinic	0	Caregiver	0	impairment	0	Glutamate	0	Type A	0	Sinus headache	0	Regimen	0	Lactation	0	Abnormalities	0	Blood flow	0	Efficacy	0
0	Prophylaxis	0	Angiotensin	0	Incidence	0	Duration	0	Patterns	0	Anger	0	Central	0	Future	0	Joy	0	Quality of life	0	Aggravate	0	Motion sickness	0	Steroid	0	Calcium channel blocker	0	faith	0	Shock	0	Population	0	boss	0	vacation	0	Neck muscle	0	Trigger point	0	NSAID	0	Agonist	0	CAT scan	0	Weight loss	2	Testing	0	Epilepsy	0	Peer support	0	stigma	0	Acute migraine	0	Cervical spine	0	Characteristic	0	Sensitivity to sound	0	Peripheral	0	Upset	0	Anti-inflammatory	0	Facial pain	0	Mechanism	0	Grandmother	0	Homeopathy	0	surgery	0	Accident	0	regime	0	Trauma	0	Mindfulness	0	Tricyclic	0	Remission	0	Histamine	0	Headache diary	0	Anti-seizure	0	Occipital nerve	0	Headache clinic	0	Caregiver	0	impairment	0	Glutamate	0	Type A	0	Sinus headache	0	Regimen	0	Lactation	0	Abnormalities	0	Blood flow	0	Efficacy	0
0	Prophylaxis	0	Angiotensin	0	Incidence	0	Duration	3	Patterns	0	Anger	0	Central	0	Future	0	Joy	0	Quality of life	0	Aggravate	3	Motion sickness	0	Steroid	0	Calcium channel blocker	0	faith	0	Shock	0	Population	0	boss	2	vacation	0	Neck muscle	0	Trigger point	0	NSAID	0	Agonist	0	CAT scan	0	Weight loss	0	Testing	0	Epilepsy	0	Peer support	0	stigma	0	Acute migraine	0	Cervical spine	4	Characteristic	4	Sensitivity to sound	0	Peripheral	0	Upset	0	Anti-inflammatory	0	Facial pain	0	Mechanism	0	Grandmother	0	Homeopathy	0	surgery	0	Accident	0	regime	0	Trauma	0	Mindfulness	0	Tricyclic	0	Remission	0	Histamine	0	Headache diary	0	Anti-seizure	0	Occipital nerve	0	Headache clinic	0	Caregiver	0	impairment	0	Glutamate	0	Type A	0	Sinus headache	0	Regimen	0	Lactation	0	Abnormalities	0	Blood flow	0	Efficacy	0
0	Prophylaxis	5	Angiotensin	7	Incidence	0	Duration	0	Patterns	1	Anger	2	Central	1	Future	0	Joy	0	Quality of life	0	Aggravate	0	Motion sickness	0	Steroid	0	Calcium channel blocker	2	faith	0	Shock	0	Population	1	boss	0	vacation	0	Neck muscle	0	Trigger point	0	NSAID	0	Agonist	2	CAT scan	0	Weight loss	0	Testing	0	Epilepsy	0	Peer support	0	stigma	0	Acute migraine	0	Cervical spine	0	Characteristic	0	Sensitivity to sound	0	Peripheral	0	Upset	1	Anti-inflammatory	0	Facial pain	0	Mechanism	0	Grandmother	0	Homeopathy	0	surgery	0	Accident	0	regime	0	Trauma	0	Mindfulness	0	Tricyclic	2	Remission	2	Histamine	2	Headache diary	0	Anti-seizure	1	Occipital nerve	0	Headache clinic	2	Caregiver	0	impairment	0	Glutamate	0	Type A	0	Sinus headache	0	Regimen	0	Lactation	0	Abnormalities	0	Blood flow	0	Efficacy	2
0	Prophylaxis	2	Angiotensin	0	Incidence	0	Duration	0	Patterns	0	Anger	0	Central	0	Future	0	Joy	0	Quality of life	0	Aggravate	0	Motion sickness	0	Steroid	0	Calcium channel blocker	0	faith	0	Shock	0	Population	0	boss	0	vacation	0	Neck muscle	0	Trigger point	0	NSAID	0	Agonist	2	CAT scan	0	Weight loss	0	Testing	0	Epilepsy	0	Peer support	0	stigma	0	Acute migraine	0	Cervical spine	0	Characteristic	0	Sensitivity to sound	0	Peripheral	0	Upset	0	Anti-inflammatory	0	Facial pain	0	Mechanism	0	Grandmother	0	Homeopathy	0	surgery	0	Accident	0	regime	0	Trauma	0	Mindfulness	0	Tricyclic	0	Remission	0	Histamine	0	Headache diary	0	Anti-seizure	0	Occipital nerve	0	Headache clinic	0	Caregiver	0	impairment	0	Glutamate	0	Type A	0	Sinus headache	0	Regimen	0	Lactation	0	Abnormalities	0	Blood flow	0	Efficacy	0
0	Prophylaxis	0	Angiotensin	0	Incidence	0	Duration	0	Patterns	0	Anger	0	Central	0	Future	0	Joy	0	Quality of life	0	Aggravate	0	Motion sickness	0	Steroid	0	Calcium channel blocker	0	faith	0	Shock	0	Population	0	boss	0	vacation	0	Neck muscle	0	Trigger point	0	NSAID	0	Agonist	0	CAT scan	0	Weight loss	0	Testing	0	Epilepsy	0	Peer support	0	stigma	0	Acute migraine	0	Cervical spine	0	Characteristic	0	Sensitivity to sound	0	Peripheral	2	Upset	1	Anti-inflammatory	0	Facial pain	0	Mechanism	0	Grandmother	0	Homeopathy	0	surgery	0	Accident	0	regime	0	Trauma	0	Mindfulness	0	Tricyclic	0	Remission	0	Histamine	0	Headache diary	0	Anti-seizure	0	Occipital nerve	0	Headache clinic	0	Caregiver	0	impairment	0	Glutamate	0	Type A	0	Sinus headache	0	Regimen	0	Lactation	0	Abnormalities	0	Blood flow	1	Efficacy	0
0	Prophylaxis	0	Angiotensin	0	Incidence	0	Duration	0	Patterns	0	Anger	0	Central	0	Future	0	Joy	0	Quality of life	0	Aggravate	0	Motion sickness	0	Steroid	0	Calcium channel blocker	0	faith	0	Shock	0	Population	0	boss	0	vacation	0	Neck muscle	0	Trigger point	0	NSAID	0	Agonist	0	CAT scan	0	Weight loss	0	Testing	0	Epilepsy	0	Peer support	0	stigma	0	Acute migraine	0	Cervical spine	0	Characteristic	0	Sensitivity to sound	0	Peripheral	0	Upset	0	Anti-inflammatory	0	Facial pain	0	Mechanism	0	Grandmother	0	Homeopathy	0	surgery	0	Accident	0	regime	1	Trauma	0	Mindfulness	0	Tricyclic	0	Remission	0	Histamine	0	Headache diary	0	Anti-seizure	0	Occipital nerve	0	Headache clinic	0	Caregiver	0	impairment	0	Glutamate	0	Type A	0	Sinus headache	0	Regimen	0	Lactation	0	Abnormalities	0	Blood flow	0	Efficacy	0
0	Prophylaxis	0	Angiotensin	0	Incidence	0	Duration	0	Patterns	0	Anger	0	Central	0	Future	0	Joy	0	Quality of life	0	Aggravate	0	Motion sickness	0	Steroid	0	Calcium channel blocker	0	faith	0	Shock	0	Population	0	boss	0	vacation	0	Neck muscle	0	Trigger point	0	NSAID	0	Agonist	0	CAT scan	0	Weight loss	0	Testing	0	Epilepsy	0	Peer support	0	stigma	0	Acute migraine	0	Cervical spine	0	Characteristic	0	Sensitivity to sound	0	Peripheral	0	Upset	0	Anti-inflammatory	0	Facial pain	0	Mechanism	0	Grandmother	0	Homeopathy	0	surgery	0	Accident	0	regime	0	Trauma	0	Mindfulness	0	Tricyclic	0	Remission	0	Histamine	0	Headache diary	0	Anti-seizure	0	Occipital nerve	0	Headache clinic	0	Caregiver	0	impairment	0	Glutamate	0	Type A	1	Sinus headache	0	Regimen	0	Lactation	0	Abnormalities	0	Blood flow	0	Efficacy	0
0	Prophylaxis	0	Angiotensin	0	Incidence	0	Duration	0	Patterns	0	Anger	0	Central	0	Future	0	Joy	0	Quality of life	0	Aggravate	0	Motion sickness	0	Steroid	0	Calcium channel blocker	0	faith	0	Shock	0	Population	0	boss	0	vacation	0	Neck muscle	1	Trigger point	0	NSAID	1	Agonist	0	CAT scan	0	Weight loss	0	Testing	0	Epilepsy	0	Peer support	0	stigma	0	Acute migraine	0	Cervical spine	0	Characteristic	0	Sensitivity to sound	0	Peripheral	0	Upset	0	Anti-inflammatory	0	Facial pain	0	Mechanism	0	Grandmother	0	Homeopathy	0	surgery	0	Accident	0	regime	0	Trauma	1	Mindfulness	0	Tricyclic	0	Remission	0	Histamine	0	Headache diary	0	Anti-seizure	0	Occipital nerve	0	Headache clinic	0	Caregiver	0	impairment	0	Glutamate	0	Type A	0	Sinus headache	0	Regimen	0	Lactation	0	Abnormalities	0	Blood flow	0	Efficacy	0
0	Prophylaxis	0	Angiotensin	0	Incidence	0	Duration	0	Patterns	1	Anger	0	Central	1	Future	0	Joy	0	Quality of life	0	Aggravate	1	Motion sickness	0	Steroid	0	Calcium channel blocker	0	faith	0	Shock	0	Population	0	boss	0	vacation	0	Neck muscle	0	Trigger point	0	NSAID	0	Agonist	0	CAT scan	1	Weight loss	0	Testing	2	Epilepsy	0	Peer support	0	stigma	0	Acute migraine	0	Cervical spine	0	Characteristic	0	Sensitivity to sound	0	Peripheral	0	Upset	0	Anti-inflammatory	0	Facial pain	1	Mechanism	0	Grandmother	0	Homeopathy	0	surgery	1	Accident	0	regime	0	Trauma	0	Mindfulness	0	Tricyclic	0	Remission	0	Histamine	0	Headache diary	2	Anti-seizure	0	Occipital nerve	0	Headache clinic	0	Caregiver	0	impairment	0	Glutamate	0	Type A	1	Sinus headache	1	Regimen	0	Lactation	0	Abnormalities	0	Blood flow	0	Efficacy	0
0	Prophylaxis	0	Angiotensin	0	Incidence	0	Duration	0	Patterns	0	Anger	0	Central	0	Future	0	Joy	0	Quality of life	0	Aggravate	0	Motion sickness	0	Steroid	0	Calcium channel blocker	0	faith	0	Shock	0	Population	0	boss	0	vacation	0	Neck muscle	0	Trigger point	0	NSAID	0	Agonist	0	CAT scan	0	Weight loss	0	Testing	0	Epilepsy	0	Peer support	0	stigma	0	Acute migraine	0	Cervical spine	0	Characteristic	0	Sensitivity to sound	0	Peripheral	0	Upset	0	Anti-inflammatory	0	Facial pain	0	Mechanism	0	Grandmother	0	Homeopathy	0	surgery	0	Accident	0	regime	0	Trauma	0	Mindfulness	0	Tricyclic	0	Remission	0	Histamine	0	Headache diary	0	Anti-seizure	0	Occipital nerve	0	Headache clinic	0	Caregiver	0	impairment	0	Glutamate	0	Type A	0	Sinus headache	0	Regimen	0	Lactation	0	Abnormalities	0	Blood flow	0	Efficacy	0
0	Prophylaxis	0	Angiotensin	0	Incidence	0	Duration	0	Patterns	0	Anger	0	Central	0	Future	0	Joy	0	Quality of life	0	Aggravate	0	Motion sickness	0	Steroid	0	Calcium channel blocker	0	faith	0	Shock	0	Population	0	boss	0	vacation	0	Neck muscle	0	Trigger point	0	NSAID	0	Agonist	0	CAT scan	0	Weight loss	0	Testing	0	Epilepsy	0	Peer support	0	stigma	0	Acute migraine	0	Cervical spine	0	Characteristic	0	Sensitivity to sound	0	Peripheral	0	Upset	0	Anti-inflammatory	0	Facial pain	0	Mechanism	0	Grandmother	0	Homeopathy	0	surgery	0	Accident	0	regime	0	Trauma	0	Mindfulness	0	Tricyclic	0	Remission	0	Histamine	0	Headache diary	0	Anti-seizure	0	Occipital nerve	0	Headache clinic	0	Caregiver	0	impairment	0	Glutamate	0	Type A	0	Sinus headache	0	Regimen	0	Lactation	0	Abnormalities	0	Blood flow	0	Efficacy	0
0	Prophylaxis	0	Angiotensin	0	Incidence	1	Duration	0	Patterns	0	Anger	2	Central	0	Future	1	Joy	0	Quality of life	0	Aggravate	0	Motion sickness	0	Steroid	0	Calcium channel blocker	0	faith	0	Shock	1	Population	1	boss	0	vacation	0	Neck muscle	1	Trigger point	0	NSAID	0	Agonist	0	CAT scan	0	Weight loss	0	Testing	0	Epilepsy	0	Peer support	0	stigma	0	Acute migraine	0	Cervical spine	0	Characteristic	0	Sensitivity to sound	1	Peripheral	0	Upset	0	Anti-inflammatory	0	Facial pain	0	Mechanism	2	Grandmother	0	Homeopathy	3	surgery	0	Accident	0	regime	0	Trauma	0	Mindfulness	0	Tricyclic	0	Remission	0	Histamine	0	Headache diary	0	Anti-seizure	0	Occipital nerve	0	Headache clinic	0	Caregiver	0	impairment	0	Glutamate	0	Type A	0	Sinus headache	0	Regimen	0	Lactation	0	Abnormalities	2	Blood flow	1	Efficacy	0
0	Prophylaxis	0	Angiotensin	0	Incidence	0	Duration	0	Patterns	0	Anger	0	Central	0	Future	0	Joy	0	Quality of life	0	Aggravate	0	Motion sickness	5	Steroid	0	Calcium channel blocker	0	faith	0	Shock	0	Population	0	boss	0	vacation	0	Neck muscle	0	Trigger point	0	NSAID	0	Agonist	0	CAT scan	0	Weight loss	0	Testing	0	Epilepsy	0	Peer support	0	stigma	0	Acute migraine	0	Cervical spine	0	Characteristic	0	Sensitivity to sound	0	Peripheral	0	Upset	0	Anti-inflammatory	0	Facial pain	0	Mechanism	0	Grandmother	0	Homeopathy	0	surgery	0	Accident	0	regime	0	Trauma	0	Mindfulness	0	Tricyclic	0	Remission	0	Histamine	0	Headache diary	0	Anti-seizure	0	Occipital nerve	0	Headache clinic	0	Caregiver	0	impairment	0	Glutamate	0	Type A	0	Sinus headache	0	Regimen	0	Lactation	0	Abnormalities	0	Blood flow	0	Efficacy	0
0	Prophylaxis	0	Angiotensin	0	Incidence	0	Duration	0	Patterns	0	Anger	0	Central	0	Future	0	Joy	0	Quality of life	0	Aggravate	0	Motion sickness	0	Steroid	0	Calcium channel blocker	0	faith	0	Shock	0	Population	0	boss	0	vacation	0	Neck muscle	0	Trigger point	0	NSAID	2	Agonist	0	CAT scan	0	Weight loss	0	Testing	0	Epilepsy	0	Peer support	0	stigma	0	Acute migraine	0	Cervical spine	0	Characteristic	0	Sensitivity to sound	0	Peripheral	0	Upset	0	Anti-inflammatory	0	Facial pain	0	Mechanism	0	Grandmother	0	Homeopathy	0	surgery	0	Accident	0	regime	0	Trauma	0	Mindfulness	0	Tricyclic	1	Remission	0	Histamine	0	Headache diary	0	Anti-seizure	0	Occipital nerve	0	Headache clinic	0	Caregiver	0	impairment	0	Glutamate	0	Type A	0	Sinus headache	0	Regimen	0	Lactation	2	Abnormalities	0	Blood flow	0	Efficacy	0
0	Prophylaxis	0	Angiotensin	0	Incidence	0	Duration	0	Patterns	0	Anger	0	Central	0	Future	0	Joy	0	Quality of life	0	Aggravate	0	Motion sickness	0	Steroid	0	Calcium channel blocker	0	faith	0	Shock	0	Population	0	boss	0	vacation	0	Neck muscle	0	Trigger point	0	NSAID	0	Agonist	0	CAT scan	2	Weight loss	0	Testing	0	Epilepsy	0	Peer support	0	stigma	0	Acute migraine	0	Cervical spine	0	Characteristic	0	Sensitivity to sound	0	Peripheral	0	Upset	0	Anti-inflammatory	0	Facial pain	0	Mechanism	0	Grandmother	0	Homeopathy	0	surgery	0	Accident	0	regime	0	Trauma	0	Mindfulness	0	Tricyclic	0	Remission	0	Histamine	0	Headache diary	0	Anti-seizure	0	Occipital nerve	0	Headache clinic	0	Caregiver	0	impairment	0	Glutamate	0	Type A	0	Sinus headache	0	Regimen	0	Lactation	0	Abnormalities	0	Blood flow	0	Efficacy	0
8		7		7		7		7		7		7		7		7		7		7		6		6		6		6		6		6		6		6		6		5		5		5		5		5		5		5		5		5		5		5		4		4		4		4		4		4		4		4		4		4		4		4		4		4		4		3		3		3		3		3		3		3		3		3		3		3		3		3		2		2		2		2

P199a	P199b	P200a	P200b	P201a	P201b	P202a	P202b	P203a	P203b	P204a	P204b	P205a	P205b	P206a	P206b	P207a	P207b	P208a	P208b	P209a	P209b	P210a	P210b	P211a	P211b	P212a	P212b	P213a	P213b	P214a	P214b	P215a	P215b	P216a	P216b	P217a	P217b	P218a	P218b	P219a	P219b	P220a	P220b	P221a	P221b	P222a	P222b	P223a	P223b	P224a	P224b	P225a	P225b	P226a	P226b	P227a	P227b	P228a	P228b	P229a	P229b	P230a	P230b	P231a	P231b	P232a	P232b	P233a	P233b	P234a	P234b	P235a	P235b	P236a	P236b	P237a	P237b	P238a	P238b	P239a	P239b	P240a	P240b	P241a	P241b	P242a	P242b	P243a	P243b	P244a	P244b	P245a	P245b	P246a	P246b	P247a	P247b	P248a	P248b	P249a	P249b	P250a	P250b	P251a	P251b	P252a	P252b	P253a	P253b	P254a	P254b
Advantage	0	Attitude	0	Antihistamine	0	Lifestyle change	0	Nerve block	0	Accepting	0	Lidocaine	0	Pain management	0	Menstrual migraine	0	Father	0	Spectrum	0	Grandfather	0	Botulinum Toxin	0	Double vision	0	Intracranial hypertension	0	church	0	Medication overuse	0	Empower	0	Suicide	0	Trigeminal autonomic cephalalgia	0	Secondary headache	0	Consultation	0	Progression	0	Beta-blocker	0	Melatonin	0	Episodic	0	Referred pain	0	Shoulder muscle	0	Detoxification	0	Abnormality	0	Beta-Blockers	0	5-HT	0	Ineffective	0	Eye symptom	0	Placebo effect	0	Foramen ovale	0	Angiotensin Converting Enzyme	0	Complications	0	Prophylactic treatment	0	Intervention	0	Subtype	0	Randomized controlled trial	0	Referral	0	Cranium	0	Temporal	0	Barbiturate	0	Septoplasty	0	nun	0	Eye pain	0	Angry	0	Emergency care	0	Food allergies	0	Motivation	0	spouse	0	Cadasil	0	5HT	0
Advantage	0	Attitude	0	Antihistamine	0	Lifestyle change	0	Nerve block	0	Accepting	0	Lidocaine	0	Pain management	0	Menstrual migraine	0	Father	0	Spectrum	0	Grandfather	0	Botulinum Toxin	0	Double vision	0	Intracranial hypertension	0	church	0	Medication overuse	0	Empower	0	Suicide	0	Trigeminal autonomic cephalalgia	0	Secondary headache	0	Consultation	0	Progression	0	Beta-blocker	0	Melatonin	0	Episodic	1	Referred pain	0	Shoulder muscle	0	Detoxification	0	Abnormality	0	Beta-Blockers	0	5-HT	0	Ineffective	0	Eye symptom	0	Placebo effect	0	Foramen ovale	0	Angiotensin Converting Enzyme	0	Complications	0	Prophylactic treatment	0	Intervention	0	Subtype	0	Randomized controlled trial	0	Referral	0	Cranium	0	Temporal	0	Barbiturate	0	Septoplasty	0	nun	0	Eye pain	0	Angry	0	Emergency care	0	Food allergies	0	Motivation	0	spouse	0	Cadasil	0	5HT	0
Advantage	0	Attitude	0	Antihistamine	0	Lifestyle change	0	Nerve block	0	Accepting	0	Lidocaine	0	Pain management	0	Menstrual migraine	0	Father	0	Spectrum	0	Grandfather	0	Botulinum Toxin	0	Double vision	0	Intracranial hypertension	0	church	0	Medication overuse	0	Empower	0	Suicide	0	Trigeminal autonomic cephalalgia	0	Secondary headache	0	Consultation	0	Progression	0	Beta-blocker	0	Melatonin	0	Episodic	0	Referred pain	0	Shoulder muscle	0	Detoxification	0	Abnormality	0	Beta-Blockers	0	5-HT	0	Ineffective	0	Eye symptom	0	Placebo effect	0	Foramen ovale	0	Angiotensin Converting Enzyme	0	Complications	0	Prophylactic treatment	0	Intervention	0	Subtype	0	Randomized controlled trial	0	Referral	0	Cranium	0	Temporal	0	Barbiturate	0	Septoplasty	0	nun	0	Eye pain	0	Angry	0	Emergency care	0	Food allergies	0	Motivation	0	spouse	0	Cadasil	0	5HT	0
Advantage	0	Attitude	0	Antihistamine	0	Lifestyle change	0	Nerve block	0	Accepting	0	Lidocaine	0	Pain management	0	Menstrual migraine	0	Father	0	Spectrum	0	Grandfather	0	Botulinum Toxin	0	Double vision	0	Intracranial hypertension	0	church	0	Medication overuse	0	Empower	0	Suicide	0	Trigeminal autonomic cephalalgia	0	Secondary headache	0	Consultation	0	Progression	0	Beta-blocker	0	Melatonin	1	Episodic	0	Referred pain	0	Shoulder muscle	0	Detoxification	0	Abnormality	0	Beta-Blockers	0	5-HT	0	Ineffective	0	Eye symptom	0	Placebo effect	0	Foramen ovale	0	Angiotensin Converting Enzyme	0	Complications	0	Prophylactic treatment	0	Intervention	0	Subtype	0	Randomized controlled trial	0	Referral	0	Cranium	0	Temporal	0	Barbiturate	0	Septoplasty	0	nun	0	Eye pain	0	Angry	0	Emergency care	0	Food allergies	0	Motivation	0	spouse	0	Cadasil	0	5HT	0
Advantage	0	Attitude	0	Antihistamine	0	Lifestyle change	0	Nerve block	0	Accepting	0	Lidocaine	0	Pain management	0	Menstrual migraine	0	Father	0	Spectrum	0	Grandfather	0	Botulinum Toxin	0	Double vision	0	Intracranial hypertension	0	church	0	Medication overuse	0	Empower	0	Suicide	0	Trigeminal autonomic cephalalgia	0	Secondary headache	0	Consultation	0	Progression	0	Beta-blocker	0	Melatonin	0	Episodic	0	Referred pain	0	Shoulder muscle	0	Detoxification	0	Abnormality	0	Beta-Blockers	0	5-HT	0	Ineffective	0	Eye symptom	0	Placebo effect	0	Foramen ovale	0	Angiotensin Converting Enzyme	0	Complications	0	Prophylactic treatment	0	Intervention	0	Subtype	0	Randomized controlled trial	0	Referral	0	Cranium	0	Temporal	0	Barbiturate	0	Septoplasty	0	nun	0	Eye pain	0	Angry	0	Emergency care	0	Food allergies	0	Motivation	0	spouse	0	Cadasil	0	5HT	0
Advantage	0	Attitude	0	Antihistamine	0	Lifestyle change	0	Nerve block	0	Accepting	0	Lidocaine	0	Pain management	0	Menstrual migraine	0	Father	0	Spectrum	0	Grandfather	0	Botulinum Toxin	0	Double vision	0	Intracranial hypertension	0	church	0	Medication overuse	0	Empower	0	Suicide	0	Trigeminal autonomic cephalalgia	0	Secondary headache	0	Consultation	0	Progression	0	Beta-blocker	0	Melatonin	0	Episodic	0	Referred pain	0	Shoulder muscle	0	Detoxification	0	Abnormality	0	Beta-Blockers	0	5-HT	0	Ineffective	0	Eye symptom	0	Placebo effect	0	Foramen ovale	0	Angiotensin Converting Enzyme	0	Complications	0	Prophylactic treatment	0	Intervention	0	Subtype	0	Randomized controlled trial	0	Referral	0	Cranium	0	Temporal	0	Barbiturate	0	Septoplasty	0	nun	0	Eye pain	0	Angry	0	Emergency care	0	Food allergies	0	Motivation	0	spouse	0	Cadasil	0	5HT	0
Advantage	0	Attitude	0	Antihistamine	0	Lifestyle change	0	Nerve block	0	Accepting	0	Lidocaine	0	Pain management	0	Menstrual migraine	0	Father	0	Spectrum	0	Grandfather	0	Botulinum Toxin	0	Double vision	0	Intracranial hypertension	0	church	0	Medication overuse	0	Empower	0	Suicide	0	Trigeminal autonomic cephalalgia	0	Secondary headache	0	Consultation	0	Progression	0	Beta-blocker	0	Melatonin	0	Episodic	0	Referred pain	0	Shoulder muscle	0	Detoxification	0	Abnormality	0	Beta-Blockers	0	5-HT	0	Ineffective	0	Eye symptom	0	Placebo effect	0	Foramen ovale	0	Angiotensin Converting Enzyme	0	Complications	0	Prophylactic treatment	0	Intervention	0	Subtype	0	Randomized controlled trial	0	Referral	0	Cranium	0	Temporal	0	Barbiturate	0	Septoplasty	0	nun	0	Eye pain	0	Angry	0	Emergency care	0	Food allergies	0	Motivation	0	spouse	0	Cadasil	0	5HT	0
Advantage	0	Attitude	0	Antihistamine	0	Lifestyle change	0	Nerve block	0	Accepting	0	Lidocaine	0	Pain management	0	Menstrual migraine	0	Father	0	Spectrum	0	Grandfather	0	Botulinum Toxin	0	Double vision	0	Intracranial hypertension	0	church	0	Medication overuse	0	Empower	0	Suicide	0	Trigeminal autonomic cephalalgia	0	Secondary headache	0	Consultation	0	Progression	0	Beta-blocker	0	Melatonin	0	Episodic	0	Referred pain	0	Shoulder muscle	0	Detoxification	0	Abnormality	0	Beta-Blockers	0	5-HT	0	Ineffective	0	Eye symptom	0	Placebo effect	0	Foramen ovale	0	Angiotensin Converting Enzyme	0	Complications	0	Prophylactic treatment	0	Intervention	0	Subtype	0	Randomized controlled trial	0	Referral	0	Cranium	0	Temporal	0	Barbiturate	0	Septoplasty	0	nun	0	Eye pain	0	Angry	0	Emergency care	0	Food allergies	0	Motivation	0	spouse	0	Cadasil	0	5HT	0
Advantage	0	Attitude	0	Antihistamine	0	Lifestyle change	0	Nerve block	0	Accepting	0	Lidocaine	0	Pain management	0	Menstrual migraine	0	Father	0	Spectrum	0	Grandfather	0	Botulinum Toxin	0	Double vision	0	Intracranial hypertension	0	church	0	Medication overuse	0	Empower	0	Suicide	0	Trigeminal autonomic cephalalgia	0	Secondary headache	0	Consultation	0	Progression	0	Beta-blocker	0	Melatonin	0	Episodic	0	Referred pain	0	Shoulder muscle	0	Detoxification	0	Abnormality	0	Beta-Blockers	0	5-HT	0	Ineffective	0	Eye symptom	0	Placebo effect	0	Foramen ovale	0	Angiotensin Converting Enzyme	0	Complications	0	Prophylactic treatment	0	Intervention	0	Subtype	0	Randomized controlled trial	0	Referral	0	Cranium	0	Temporal	0	Barbiturate	0	Septoplasty	0	nun	0	Eye pain	0	Angry	0	Emergency care	0	Food allergies	0	Motivation	0	spouse	0	Cadasil	0	5HT	0
Advantage	0	Attitude	0	Antihistamine	0	Lifestyle change	0	Nerve block	0	Accepting	0	Lidocaine	0	Pain management	0	Menstrual migraine	0	Father	0	Spectrum	0	Grandfather	0	Botulinum Toxin	0	Double vision	0	Intracranial hypertension	0	church	0	Medication overuse	0	Empower	0	Suicide	0	Trigeminal autonomic cephalalgia	0	Secondary headache	0	Consultation	0	Progression	0	Beta-blocker	0	Melatonin	0	Episodic	0	Referred pain	0	Shoulder muscle	0	Detoxification	0	Abnormality	0	Beta-Blockers	0	5-HT	0	Ineffective	0	Eye symptom	0	Placebo effect	0	Foramen ovale	0	Angiotensin Converting Enzyme	0	Complications	0	Prophylactic treatment	0	Intervention	0	Subtype	0	Randomized controlled trial	0	Referral	0	Cranium	0	Temporal	0	Barbiturate	0	Septoplasty	0	nun	0	Eye pain	0	Angry	0	Emergency care	0	Food allergies	0	Motivation	0	spouse	0	Cadasil	0	5HT	0
Advantage	0	Attitude	0	Antihistamine	0	Lifestyle change	0	Nerve block	0	Accepting	0	Lidocaine	0	Pain management	0	Menstrual migraine	0	Father	0	Spectrum	0	Grandfather	0	Botulinum Toxin	0	Double vision	0	Intracranial hypertension	0	church	0	Medication overuse	0	Empower	0	Suicide	0	Trigeminal autonomic cephalalgia	0	Secondary headache	0	Consultation	0	Progression	0	Beta-blocker	0	Melatonin	0	Episodic	0	Referred pain	0	Shoulder muscle	0	Detoxification	0	Abnormality	0	Beta-Blockers	0	5-HT	0	Ineffective	0	Eye symptom	0	Placebo effect	0	Foramen ovale	0	Angiotensin Converting Enzyme	0	Complications	0	Prophylactic treatment	0	Intervention	0	Subtype	0	Randomized controlled trial	0	Referral	0	Cranium	0	Temporal	0	Barbiturate	0	Septoplasty	0	nun	0	Eye pain	0	Angry	0	Emergency care	0	Food allergies	0	Motivation	0	spouse	0	Cadasil	0	5HT	0
Advantage	0	Attitude	0	Antihistamine	0	Lifestyle change	0	Nerve block	0	Accepting	0	Lidocaine	0	Pain management	0	Menstrual migraine	0	Father	0	Spectrum	0	Grandfather	0	Botulinum Toxin	0	Double vision	0	Intracranial hypertension	0	church	0	Medication overuse	0	Empower	0	Suicide	0	Trigeminal autonomic cephalalgia	0	Secondary headache	0	Consultation	0	Progression	0	Beta-blocker	0	Melatonin	0	Episodic	0	Referred pain	0	Shoulder muscle	0	Detoxification	0	Abnormality	0	Beta-Blockers	0	5-HT	0	Ineffective	0	Eye symptom	0	Placebo effect	0	Foramen ovale	0	Angiotensin Converting Enzyme	0	Complications	0	Prophylactic treatment	0	Intervention	0	Subtype	0	Randomized controlled trial	0	Referral	0	Cranium	0	Temporal	0	Barbiturate	0	Septoplasty	0	nun	0	Eye pain	0	Angry	0	Emergency care	0	Food allergies	0	Motivation	0	spouse	0	Cadasil	0	5HT	0
Advantage	0	Attitude	0	Antihistamine	0	Lifestyle change	0	Nerve block	0	Accepting	0	Lidocaine	0	Pain management	0	Menstrual migraine	0	Father	0	Spectrum	0	Grandfather	0	Botulinum Toxin	0	Double vision	0	Intracranial hypertension	0	church	0	Medication overuse	0	Empower	0	Suicide	0	Trigeminal autonomic cephalalgia	0	Secondary headache	0	Consultation	0	Progression	0	Beta-blocker	1	Melatonin	0	Episodic	0	Referred pain	0	Shoulder muscle	0	Detoxification	0	Abnormality	0	Beta-Blockers	0	5-HT	0	Ineffective	0	Eye symptom	0	Placebo effect	0	Foramen ovale	0	Angiotensin Converting Enzyme	0	Complications	0	Prophylactic treatment	0	Intervention	0	Subtype	0	Randomized controlled trial	0	Referral	0	Cranium	0	Temporal	0	Barbiturate	0	Septoplasty	0	nun	0	Eye pain	0	Angry	0	Emergency care	0	Food allergies	0	Motivation	0	spouse	0	Cadasil	0	5HT	0
Advantage	0	Attitude	0	Antihistamine	0	Lifestyle change	0	Nerve block	0	Accepting	0	Lidocaine	0	Pain management	0	Menstrual migraine	0	Father	0	Spectrum	0	Grandfather	0	Botulinum Toxin	0	Double vision	0	Intracranial hypertension	0	church	0	Medication overuse	0	Empower	0	Suicide	0	Trigeminal autonomic cephalalgia	0	Secondary headache	0	Consultation	0	Progression	0	Beta-blocker	0	Melatonin	0	Episodic	0	Referred pain	0	Shoulder muscle	0	Detoxification	0	Abnormality	0	Beta-Blockers	0	5-HT	0	Ineffective	0	Eye symptom	0	Placebo effect	0	Foramen ovale	0	Angiotensin Converting Enzyme	0	Complications	0	Prophylactic treatment	0	Intervention	0	Subtype	0	Randomized controlled trial	0	Referral	0	Cranium	0	Temporal	0	Barbiturate	0	Septoplasty	0	nun	0	Eye pain	0	Angry	0	Emergency care	0	Food allergies	0	Motivation	0	spouse	0	Cadasil	0	5HT	0
Advantage	0	Attitude	0	Antihistamine	0	Lifestyle change	0	Nerve block	0	Accepting	0	Lidocaine	0	Pain management	0	Menstrual migraine	0	Father	0	Spectrum	0	Grandfather	0	Botulinum Toxin	0	Double vision	0	Intracranial hypertension	0	church	0	Medication overuse	0	Empower	0	Suicide	0	Trigeminal autonomic cephalalgia	0	Secondary headache	0	Consultation	0	Progression	0	Beta-blocker	0	Melatonin	0	Episodic	0	Referred pain	0	Shoulder muscle	0	Detoxification	0	Abnormality	0	Beta-Blockers	0	5-HT	0	Ineffective	0	Eye symptom	0	Placebo effect	0	Foramen ovale	0	Angiotensin Converting Enzyme	0	Complications	0	Prophylactic treatment	0	Intervention	0	Subtype	0	Randomized controlled trial	0	Referral	0	Cranium	0	Temporal	0	Barbiturate	0	Septoplasty	0	nun	0	Eye pain	0	Angry	0	Emergency care	0	Food allergies	0	Motivation	0	spouse	0	Cadasil	0	5HT	0
Advantage	0	Attitude	0	Antihistamine	0	Lifestyle change	0	Nerve block	0	Accepting	0	Lidocaine	0	Pain management	0	Menstrual migraine	0	Father	0	Spectrum	0	Grandfather	0	Botulinum Toxin	0	Double vision	0	Intracranial hypertension	0	church	0	Medication overuse	0	Empower	0	Suicide	0	Trigeminal autonomic cephalalgia	0	Secondary headache	0	Consultation	1	Progression	1	Beta-blocker	0	Melatonin	0	Episodic	0	Referred pain	0	Shoulder muscle	0	Detoxification	0	Abnormality	0	Beta-Blockers	0	5-HT	0	Ineffective	0	Eye symptom	0	Placebo effect	0	Foramen ovale	0	Angiotensin Converting Enzyme	0	Complications	0	Prophylactic treatment	0	Intervention	0	Subtype	0	Randomized controlled trial	0	Referral	0	Cranium	0	Temporal	0	Barbiturate	0	Septoplasty	0	nun	0	Eye pain	0	Angry	0	Emergency care	0	Food allergies	0	Motivation	0	spouse	0	Cadasil	0	5HT	0
Advantage	0	Attitude	0	Antihistamine	0	Lifestyle change	0	Nerve block	0	Accepting	0	Lidocaine	0	Pain management	0	Menstrual migraine	0	Father	0	Spectrum	0	Grandfather	0	Botulinum Toxin	0	Double vision	0	Intracranial hypertension	0	church	0	Medication overuse	0	Empower	0	Suicide	0	Trigeminal autonomic cephalalgia	2	Secondary headache	2	Consultation	0	Progression	0	Beta-blocker	0	Melatonin	0	Episodic	0	Referred pain	0	Shoulder muscle	0	Detoxification	0	Abnormality	0	Beta-Blockers	0	5-HT	0	Ineffective	0	Eye symptom	0	Placebo effect	0	Foramen ovale	0	Angiotensin Converting Enzyme	0	Complications	0	Prophylactic treatment	0	Intervention	0	Subtype	0	Randomized controlled trial	0	Referral	0	Cranium	0	Temporal	0	Barbiturate	0	Septoplasty	0	nun	0	Eye pain	0	Angry	0	Emergency care	0	Food allergies	0	Motivation	0	spouse	0	Cadasil	0	5HT	0
Advantage	0	Attitude	0	Antihistamine	0	Lifestyle change	0	Nerve block	0	Accepting	0	Lidocaine	0	Pain management	0	Menstrual migraine	0	Father	0	Spectrum	0	Grandfather	0	Botulinum Toxin	0	Double vision	0	Intracranial hypertension	0	church	0	Medication overuse	0	Empower	0	Suicide	0	Trigeminal autonomic cephalalgia	0	Secondary headache	0	Consultation	0	Progression	0	Beta-blocker	0	Melatonin	0	Episodic	0	Referred pain	0	Shoulder muscle	0	Detoxification	0	Abnormality	0	Beta-Blockers	0	5-HT	0	Ineffective	0	Eye symptom	0	Placebo effect	0	Foramen ovale	0	Angiotensin Converting Enzyme	0	Complications	0	Prophylactic treatment	0	Intervention	0	Subtype	0	Randomized controlled trial	0	Referral	0	Cranium	0	Temporal	0	Barbiturate	0	Septoplasty	0	nun	0	Eye pain	0	Angry	0	Emergency care	0	Food allergies	0	Motivation	0	spouse	0	Cadasil	0	5HT	0
Advantage	0	Attitude	0	Antihistamine	0	Lifestyle change	0	Nerve block	0	Accepting	0	Lidocaine	0	Pain management	0	Menstrual migraine	0	Father	0	Spectrum	0	Grandfather	0	Botulinum Toxin	0	Double vision	0	Intracranial hypertension	0	church	0	Medication overuse	0	Empower	0	Suicide	0	Trigeminal autonomic cephalalgia	0	Secondary headache	0	Consultation	0	Progression	0	Beta-blocker	0	Melatonin	0	Episodic	0	Referred pain	0	Shoulder muscle	0	Detoxification	0	Abnormality	0	Beta-Blockers	0	5-HT	0	Ineffective	0	Eye symptom	0	Placebo effect	0	Foramen ovale	0	Angiotensin Converting Enzyme	0	Complications	0	Prophylactic treatment	0	Intervention	0	Subtype	0	Randomized controlled trial	0	Referral	0	Cranium	0	Temporal	0	Barbiturate	0	Septoplasty	0	nun	0	Eye pain	0	Angry	0	Emergency care	0	Food allergies	0	Motivation	0	spouse	0	Cadasil	0	5HT	0
Advantage	0	Attitude	0	Antihistamine	0	Lifestyle change	0	Nerve block	0	Accepting	0	Lidocaine	0	Pain management	0	Menstrual migraine	0	Father	0	Spectrum	0	Grandfather	0	Botulinum Toxin	0	Double vision	0	Intracranial hypertension	0	church	0	Medication overuse	0	Empower	0	Suicide	0	Trigeminal autonomic cephalalgia	0	Secondary headache	0	Consultation	0	Progression	0	Beta-blocker	0	Melatonin	0	Episodic	0	Referred pain	0	Shoulder muscle	0	Detoxification	0	Abnormality	0	Beta-Blockers	0	5-HT	0	Ineffective	0	Eye symptom	0	Placebo effect	0	Foramen ovale	0	Angiotensin Converting Enzyme	0	Complications	0	Prophylactic treatment	0	Intervention	0	Subtype	0	Randomized controlled trial	0	Referral	0	Cranium	0	Temporal	0	Barbiturate	0	Septoplasty	0	nun	0	Eye pain	0	Angry	0	Emergency care	0	Food allergies	0	Motivation	0	spouse	0	Cadasil	0	5HT	0
Advantage	0	Attitude	0	Antihistamine	0	Lifestyle change	0	Nerve block	0	Accepting	0	Lidocaine	0	Pain management	0	Menstrual migraine	0	Father	0	Spectrum	0	Grandfather	0	Botulinum Toxin	0	Double vision	0	Intracranial hypertension	0	church	0	Medication overuse	0	Empower	0	Suicide	0	Trigeminal autonomic cephalalgia	0	Secondary headache	0	Consultation	0	Progression	0	Beta-blocker	0	Melatonin	0	Episodic	0	Referred pain	0	Shoulder muscle	0	Detoxification	0	Abnormality	0	Beta-Blockers	0	5-HT	0	Ineffective	0	Eye symptom	0	Placebo effect	0	Foramen ovale	0	Angiotensin Converting Enzyme	0	Complications	0	Prophylactic treatment	0	Intervention	0	Subtype	0	Randomized controlled trial	0	Referral	0	Cranium	0	Temporal	0	Barbiturate	0	Septoplasty	0	nun	0	Eye pain	0	Angry	0	Emergency care	0	Food allergies	0	Motivation	0	spouse	0	Cadasil	0	5HT	0
Advantage	0	Attitude	0	Antihistamine	0	Lifestyle change	0	Nerve block	0	Accepting	0	Lidocaine	0	Pain management	0	Menstrual migraine	0	Father	0	Spectrum	0	Grandfather	0	Botulinum Toxin	0	Double vision	0	Intracranial hypertension	0	church	0	Medication overuse	0	Empower	0	Suicide	0	Trigeminal autonomic cephalalgia	0	Secondary headache	0	Consultation	1	Progression	0	Beta-blocker	0	Melatonin	0	Episodic	0	Referred pain	0	Shoulder muscle	0	Detoxification	0	Abnormality	0	Beta-Blockers	0	5-HT	0	Ineffective	0	Eye symptom	0	Placebo effect	0	Foramen ovale	0	Angiotensin Converting Enzyme	0	Complications	0	Prophylactic treatment	0	Intervention	0	Subtype	0	Randomized controlled trial	0	Referral	0	Cranium	0	Temporal	0	Barbiturate	0	Septoplasty	0	nun	0	Eye pain	0	Angry	0	Emergency care	0	Food allergies	0	Motivation	0	spouse	0	Cadasil	0	5HT	0
Advantage	0	Attitude	0	Antihistamine	0	Lifestyle change	0	Nerve block	0	Accepting	0	Lidocaine	0	Pain management	0	Menstrual migraine	0	Father	0	Spectrum	0	Grandfather	0	Botulinum Toxin	0	Double vision	0	Intracranial hypertension	0	church	0	Medication overuse	0	Empower	0	Suicide	0	Trigeminal autonomic cephalalgia	0	Secondary headache	0	Consultation	0	Progression	0	Beta-blocker	0	Melatonin	0	Episodic	0	Referred pain	0	Shoulder muscle	0	Detoxification	0	Abnormality	0	Beta-Blockers	0	5-HT	0	Ineffective	0	Eye symptom	0	Placebo effect	0	Foramen ovale	0	Angiotensin Converting Enzyme	0	Complications	0	Prophylactic treatment	0	Intervention	0	Subtype	0	Randomized controlled trial	0	Referral	0	Cranium	0	Temporal	0	Barbiturate	0	Septoplasty	0	nun	0	Eye pain	0	Angry	0	Emergency care	0	Food allergies	0	Motivation	0	spouse	0	Cadasil	0	5HT	0
Advantage	0	Attitude	0	Antihistamine	0	Lifestyle change	0	Nerve block	0	Accepting	0	Lidocaine	0	Pain management	0	Menstrual migraine	0	Father	0	Spectrum	0	Grandfather	0	Botulinum Toxin	0	Double vision	0	Intracranial hypertension	0	church	0	Medication overuse	0	Empower	0	Suicide	0	Trigeminal autonomic cephalalgia	0	Secondary headache	0	Consultation	0	Progression	0	Beta-blocker	0	Melatonin	0	Episodic	0	Referred pain	0	Shoulder muscle	0	Detoxification	0	Abnormality	0	Beta-Blockers	0	5-HT	0	Ineffective	0	Eye symptom	0	Placebo effect	0	Foramen ovale	0	Angiotensin Converting Enzyme	0	Complications	0	Prophylactic treatment	0	Intervention	0	Subtype	0	Randomized controlled trial	0	Referral	0	Cranium	0	Temporal	0	Barbiturate	0	Septoplasty	0	nun	0	Eye pain	0	Angry	0	Emergency care	0	Food allergies	0	Motivation	0	spouse	0	Cadasil	0	5HT	0
Advantage	0	Attitude	0	Antihistamine	0	Lifestyle change	0	Nerve block	0	Accepting	0	Lidocaine	0	Pain management	0	Menstrual migraine	0	Father	0	Spectrum	0	Grandfather	0	Botulinum Toxin	0	Double vision	0	Intracranial hypertension	0	church	0	Medication overuse	0	Empower	0	Suicide	0	Trigeminal autonomic cephalalgia	0	Secondary headache	0	Consultation	0	Progression	0	Beta-blocker	0	Melatonin	0	Episodic	1	Referred pain	0	Shoulder muscle	0	Detoxification	0	Abnormality	0	Beta-Blockers	0	5-HT	0	Ineffective	0	Eye symptom	0	Placebo effect	0	Foramen ovale	0	Angiotensin Converting Enzyme	0	Complications	0	Prophylactic treatment	0	Intervention	0	Subtype	0	Randomized controlled trial	0	Referral	0	Cranium	0	Temporal	0	Barbiturate	0	Septoplasty	0	nun	0	Eye pain	0	Angry	0	Emergency care	0	Food allergies	0	Motivation	0	spouse	0	Cadasil	0	5HT	0
Advantage	0	Attitude	0	Antihistamine	0	Lifestyle change	0	Nerve block	0	Accepting	0	Lidocaine	0	Pain management	0	Menstrual migraine	0	Father	0	Spectrum	0	Grandfather	0	Botulinum Toxin	0	Double vision	0	Intracranial hypertension	0	church	0	Medication overuse	0	Empower	0	Suicide	0	Trigeminal autonomic cephalalgia	0	Secondary headache	0	Consultation	0	Progression	0	Beta-blocker	0	Melatonin	0	Episodic	0	Referred pain	0	Shoulder muscle	0	Detoxification	0	Abnormality	0	Beta-Blockers	0	5-HT	0	Ineffective	0	Eye symptom	0	Placebo effect	0	Foramen ovale	0	Angiotensin Converting Enzyme	0	Complications	0	Prophylactic treatment	0	Intervention	0	Subtype	0	Randomized controlled trial	0	Referral	0	Cranium	0	Temporal	0	Barbiturate	0	Septoplasty	0	nun	0	Eye pain	0	Angry	0	Emergency care	0	Food allergies	0	Motivation	0	spouse	0	Cadasil	0	5HT	0
Advantage	0	Attitude	0	Antihistamine	0	Lifestyle change	0	Nerve block	0	Accepting	0	Lidocaine	0	Pain management	0	Menstrual migraine	0	Father	0	Spectrum	0	Grandfather	0	Botulinum Toxin	0	Double vision	0	Intracranial hypertension	0	church	0	Medication overuse	2	Empower	1	Suicide	2	Trigeminal autonomic cephalalgia	0	Secondary headache	0	Consultation	0	Progression	0	Beta-blocker	0	Melatonin	0	Episodic	0	Referred pain	0	Shoulder muscle	0	Detoxification	0	Abnormality	0	Beta-Blockers	0	5-HT	0	Ineffective	0	Eye symptom	0	Placebo effect	0	Foramen ovale	0	Angiotensin Converting Enzyme	0	Complications	0	Prophylactic treatment	0	Intervention	0	Subtype	0	Randomized controlled trial	0	Referral	0	Cranium	0	Temporal	0	Barbiturate	0	Septoplasty	0	nun	0	Eye pain	0	Angry	0	Emergency care	0	Food allergies	0	Motivation	0	spouse	0	Cadasil	0	5HT	0
Advantage	0	Attitude	0	Antihistamine	0	Lifestyle change	0	Nerve block	0	Accepting	0	Lidocaine	0	Pain management	0	Menstrual migraine	0	Father	0	Spectrum	0	Grandfather	0	Botulinum Toxin	0	Double vision	0	Intracranial hypertension	0	church	0	Medication overuse	0	Empower	0	Suicide	0	Trigeminal autonomic cephalalgia	0	Secondary headache	0	Consultation	0	Progression	0	Beta-blocker	0	Melatonin	0	Episodic	0	Referred pain	0	Shoulder muscle	0	Detoxification	0	Abnormality	0	Beta-Blockers	0	5-HT	0	Ineffective	0	Eye symptom	0	Placebo effect	0	Foramen ovale	0	Angiotensin Converting Enzyme	0	Complications	0	Prophylactic treatment	0	Intervention	0	Subtype	0	Randomized controlled trial	0	Referral	0	Cranium	0	Temporal	0	Barbiturate	0	Septoplasty	0	nun	0	Eye pain	0	Angry	0	Emergency care	0	Food allergies	0	Motivation	0	spouse	0	Cadasil	0	5HT	0
Advantage	0	Attitude	0	Antihistamine	0	Lifestyle change	0	Nerve block	0	Accepting	0	Lidocaine	0	Pain management	0	Menstrual migraine	0	Father	0	Spectrum	0	Grandfather	0	Botulinum Toxin	0	Double vision	1	Intracranial hypertension	1	church	1	Medication overuse	0	Empower	0	Suicide	0	Trigeminal autonomic cephalalgia	0	Secondary headache	0	Consultation	0	Progression	0	Beta-blocker	0	Melatonin	0	Episodic	0	Referred pain	0	Shoulder muscle	0	Detoxification	0	Abnormality	0	Beta-Blockers	0	5-HT	0	Ineffective	0	Eye symptom	0	Placebo effect	0	Foramen ovale	0	Angiotensin Converting Enzyme	0	Complications	0	Prophylactic treatment	0	Intervention	0	Subtype	0	Randomized controlled trial	0	Referral	0	Cranium	0	Temporal	0	Barbiturate	0	Septoplasty	0	nun	0	Eye pain	0	Angry	0	Emergency care	0	Food allergies	0	Motivation	0	spouse	0	Cadasil	0	5HT	0
Advantage	0	Attitude	0	Antihistamine	0	Lifestyle change	0	Nerve block	0	Accepting	0	Lidocaine	0	Pain management	0	Menstrual migraine	0	Father	0	Spectrum	0	Grandfather	0	Botulinum Toxin	0	Double vision	0	Intracranial hypertension	0	church	0	Medication overuse	0	Empower	0	Suicide	0	Trigeminal autonomic cephalalgia	0	Secondary headache	0	Consultation	0	Progression	0	Beta-blocker	0	Melatonin	0	Episodic	0	Referred pain	0	Shoulder muscle	0	Detoxification	0	Abnormality	0	Beta-Blockers	0	5-HT	0	Ineffective	0	Eye symptom	0	Placebo effect	0	Foramen ovale	0	Angiotensin Converting Enzyme	0	Complications	0	Prophylactic treatment	0	Intervention	0	Subtype	0	Randomized controlled trial	0	Referral	0	Cranium	0	Temporal	0	Barbiturate	0	Septoplasty	0	nun	0	Eye pain	0	Angry	0	Emergency care	0	Food allergies	0	Motivation	0	spouse	0	Cadasil	0	5HT	0
Advantage	0	Attitude	0	Antihistamine	0	Lifestyle change	0	Nerve block	0	Accepting	0	Lidocaine	0	Pain management	0	Menstrual migraine	0	Father	0	Spectrum	0	Grandfather	0	Botulinum Toxin	0	Double vision	0	Intracranial hypertension	0	church	0	Medication overuse	0	Empower	0	Suicide	0	Trigeminal autonomic cephalalgia	0	Secondary headache	0	Consultation	0	Progression	0	Beta-blocker	0	Melatonin	0	Episodic	0	Referred pain	0	Shoulder muscle	0	Detoxification	0	Abnormality	0	Beta-Blockers	0	5-HT	0	Ineffective	0	Eye symptom	0	Placebo effect	0	Foramen ovale	0	Angiotensin Converting Enzyme	0	Complications	0	Prophylactic treatment	0	Intervention	0	Subtype	0	Randomized controlled trial	0	Referral	0	Cranium	0	Temporal	0	Barbiturate	0	Septoplasty	0	nun	0	Eye pain	0	Angry	0	Emergency care	0	Food allergies	0	Motivation	0	spouse	0	Cadasil	0	5HT	0
Advantage	0	Attitude	0	Antihistamine	0	Lifestyle change	0	Nerve block	0	Accepting	0	Lidocaine	0	Pain management	0	Menstrual migraine	0	Father	0	Spectrum	0	Grandfather	0	Botulinum Toxin	1	Double vision	0	Intracranial hypertension	0	church	0	Medication overuse	0	Empower	0	Suicide	0	Trigeminal autonomic cephalalgia	0	Secondary headache	0	Consultation	0	Progression	0	Beta-blocker	0	Melatonin	0	Episodic	0	Referred pain	0	Shoulder muscle	0	Detoxification	0	Abnormality	0	Beta-Blockers	0	5-HT	0	Ineffective	0	Eye symptom	0	Placebo effect	0	Foramen ovale	0	Angiotensin Converting Enzyme	0	Complications	0	Prophylactic treatment	0	Intervention	0	Subtype	0	Randomized controlled trial	0	Referral	0	Cranium	0	Temporal	0	Barbiturate	0	Septoplasty	0	nun	0	Eye pain	0	Angry	0	Emergency care	0	Food allergies	0	Motivation	0	spouse	0	Cadasil	0	5HT	0
Advantage	0	Attitude	0	Antihistamine	0	Lifestyle change	0	Nerve block	0	Accepting	0	Lidocaine	0	Pain management	0	Menstrual migraine	0	Father	0	Spectrum	0	Grandfather	0	Botulinum Toxin	0	Double vision	0	Intracranial hypertension	0	church	0	Medication overuse	0	Empower	0	Suicide	0	Trigeminal autonomic cephalalgia	0	Secondary headache	0	Consultation	0	Progression	0	Beta-blocker	0	Melatonin	0	Episodic	0	Referred pain	0	Shoulder muscle	0	Detoxification	0	Abnormality	0	Beta-Blockers	0	5-HT	0	Ineffective	0	Eye symptom	0	Placebo effect	0	Foramen ovale	0	Angiotensin Converting Enzyme	0	Complications	0	Prophylactic treatment	0	Intervention	0	Subtype	0	Randomized controlled trial	0	Referral	0	Cranium	0	Temporal	0	Barbiturate	0	Septoplasty	0	nun	0	Eye pain	0	Angry	0	Emergency care	0	Food allergies	0	Motivation	0	spouse	0	Cadasil	0	5HT	0
Advantage	0	Attitude	0	Antihistamine	0	Lifestyle change	0	Nerve block	0	Accepting	0	Lidocaine	0	Pain management	0	Menstrual migraine	0	Father	0	Spectrum	0	Grandfather	0	Botulinum Toxin	0	Double vision	0	Intracranial hypertension	0	church	0	Medication overuse	0	Empower	0	Suicide	0	Trigeminal autonomic cephalalgia	0	Secondary headache	0	Consultation	0	Progression	0	Beta-blocker	0	Melatonin	0	Episodic	0	Referred pain	0	Shoulder muscle	0	Detoxification	0	Abnormality	0	Beta-Blockers	0	5-HT	0	Ineffective	0	Eye symptom	0	Placebo effect	0	Foramen ovale	0	Angiotensin Converting Enzyme	0	Complications	0	Prophylactic treatment	0	Intervention	0	Subtype	0	Randomized controlled trial	0	Referral	0	Cranium	0	Temporal	0	Barbiturate	0	Septoplasty	0	nun	0	Eye pain	0	Angry	0	Emergency care	0	Food allergies	0	Motivation	0	spouse	0	Cadasil	0	5HT	0
Advantage	0	Attitude	0	Antihistamine	0	Lifestyle change	0	Nerve block	0	Accepting	0	Lidocaine	0	Pain management	0	Menstrual migraine	0	Father	0	Spectrum	0	Grandfather	0	Botulinum Toxin	0	Double vision	0	Intracranial hypertension	0	church	0	Medication overuse	0	Empower	0	Suicide	0	Trigeminal autonomic cephalalgia	0	Secondary headache	0	Consultation	0	Progression	0	Beta-blocker	0	Melatonin	0	Episodic	0	Referred pain	0	Shoulder muscle	0	Detoxification	0	Abnormality	0	Beta-Blockers	0	5-HT	0	Ineffective	0	Eye symptom	0	Placebo effect	0	Foramen ovale	0	Angiotensin Converting Enzyme	0	Complications	0	Prophylactic treatment	0	Intervention	0	Subtype	0	Randomized controlled trial	0	Referral	0	Cranium	0	Temporal	0	Barbiturate	0	Septoplasty	0	nun	0	Eye pain	0	Angry	0	Emergency care	0	Food allergies	0	Motivation	0	spouse	0	Cadasil	0	5HT	0
Advantage	0	Attitude	0	Antihistamine	0	Lifestyle change	0	Nerve block	0	Accepting	0	Lidocaine	0	Pain management	0	Menstrual migraine	0	Father	1	Spectrum	1	Grandfather	1	Botulinum Toxin	0	Double vision	0	Intracranial hypertension	0	church	0	Medication overuse	0	Empower	0	Suicide	0	Trigeminal autonomic cephalalgia	0	Secondary headache	0	Consultation	0	Progression	0	Beta-blocker	0	Melatonin	0	Episodic	0	Referred pain	0	Shoulder muscle	0	Detoxification	0	Abnormality	0	Beta-Blockers	0	5-HT	0	Ineffective	0	Eye symptom	0	Placebo effect	0	Foramen ovale	0	Angiotensin Converting Enzyme	0	Complications	0	Prophylactic treatment	0	Intervention	0	Subtype	0	Randomized controlled trial	0	Referral	0	Cranium	0	Temporal	0	Barbiturate	0	Septoplasty	0	nun	0	Eye pain	0	Angry	0	Emergency care	0	Food allergies	0	Motivation	0	spouse	0	Cadasil	0	5HT	0
Advantage	0	Attitude	0	Antihistamine	0	Lifestyle change	0	Nerve block	0	Accepting	0	Lidocaine	0	Pain management	0	Menstrual migraine	0	Father	0	Spectrum	0	Grandfather	0	Botulinum Toxin	0	Double vision	0	Intracranial hypertension	0	church	0	Medication overuse	0	Empower	0	Suicide	0	Trigeminal autonomic cephalalgia	0	Secondary headache	0	Consultation	0	Progression	0	Beta-blocker	0	Melatonin	0	Episodic	0	Referred pain	0	Shoulder muscle	0	Detoxification	0	Abnormality	0	Beta-Blockers	0	5-HT	0	Ineffective	0	Eye symptom	0	Placebo effect	0	Foramen ovale	0	Angiotensin Converting Enzyme	0	Complications	0	Prophylactic treatment	0	Intervention	0	Subtype	0	Randomized controlled trial	0	Referral	0	Cranium	0	Temporal	0	Barbiturate	0	Septoplasty	0	nun	0	Eye pain	0	Angry	0	Emergency care	0	Food allergies	0	Motivation	0	spouse	0	Cadasil	0	5HT	0
Advantage	0	Attitude	0	Antihistamine	0	Lifestyle change	0	Nerve block	0	Accepting	0	Lidocaine	0	Pain management	0	Menstrual migraine	0	Father	0	Spectrum	0	Grandfather	0	Botulinum Toxin	0	Double vision	0	Intracranial hypertension	0	church	0	Medication overuse	0	Empower	0	Suicide	0	Trigeminal autonomic cephalalgia	0	Secondary headache	0	Consultation	0	Progression	0	Beta-blocker	0	Melatonin	0	Episodic	0	Referred pain	0	Shoulder muscle	0	Detoxification	0	Abnormality	0	Beta-Blockers	0	5-HT	0	Ineffective	0	Eye symptom	0	Placebo effect	0	Foramen ovale	0	Angiotensin Converting Enzyme	0	Complications	0	Prophylactic treatment	0	Intervention	0	Subtype	0	Randomized controlled trial	0	Referral	0	Cranium	0	Temporal	0	Barbiturate	0	Septoplasty	0	nun	0	Eye pain	0	Angry	0	Emergency care	0	Food allergies	0	Motivation	0	spouse	0	Cadasil	0	5HT	0
Advantage	0	Attitude	0	Antihistamine	0	Lifestyle change	2	Nerve block	2	Accepting	2	Lidocaine	1	Pain management	1	Menstrual migraine	1	Father	0	Spectrum	1	Grandfather	0	Botulinum Toxin	0	Double vision	0	Intracranial hypertension	0	church	0	Medication overuse	0	Empower	0	Suicide	0	Trigeminal autonomic cephalalgia	0	Secondary headache	0	Consultation	0	Progression	0	Beta-blocker	0	Melatonin	0	Episodic	0	Referred pain	0	Shoulder muscle	0	Detoxification	0	Abnormality	0	Beta-Blockers	0	5-HT	0	Ineffective	0	Eye symptom	0	Placebo effect	0	Foramen ovale	0	Angiotensin Converting Enzyme	0	Complications	0	Prophylactic treatment	0	Intervention	0	Subtype	0	Randomized controlled trial	0	Referral	0	Cranium	0	Temporal	1	Barbiturate	1	Septoplasty	1	nun	1	Eye pain	1	Angry	1	Emergency care	1	Food allergies	1	Motivation	1	spouse	1	Cadasil	1	5HT	1
Advantage	0	Attitude	0	Antihistamine	0	Lifestyle change	0	Nerve block	0	Accepting	0	Lidocaine	0	Pain management	0	Menstrual migraine	0	Father	1	Spectrum	0	Grandfather	1	Botulinum Toxin	0	Double vision	0	Intracranial hypertension	0	church	0	Medication overuse	0	Empower	0	Suicide	0	Trigeminal autonomic cephalalgia	0	Secondary headache	0	Consultation	0	Progression	0	Beta-blocker	0	Melatonin	0	Episodic	0	Referred pain	0	Shoulder muscle	0	Detoxification	0	Abnormality	0	Beta-Blockers	0	5-HT	0	Ineffective	0	Eye symptom	0	Placebo effect	0	Foramen ovale	0	Angiotensin Converting Enzyme	0	Complications	0	Prophylactic treatment	0	Intervention	0	Subtype	0	Randomized controlled trial	0	Referral	0	Cranium	1	Temporal	0	Barbiturate	0	Septoplasty	0	nun	0	Eye pain	0	Angry	0	Emergency care	0	Food allergies	0	Motivation	0	spouse	0	Cadasil	0	5HT	0
Advantage	0	Attitude	0	Antihistamine	1	Lifestyle change	0	Nerve block	0	Accepting	0	Lidocaine	0	Pain management	0	Menstrual migraine	0	Father	0	Spectrum	0	Grandfather	0	Botulinum Toxin	0	Double vision	0	Intracranial hypertension	0	church	0	Medication overuse	0	Empower	0	Suicide	0	Trigeminal autonomic cephalalgia	0	Secondary headache	0	Consultation	0	Progression	0	Beta-blocker	0	Melatonin	0	Episodic	0	Referred pain	0	Shoulder muscle	0	Detoxification	0	Abnormality	0	Beta-Blockers	0	5-HT	0	Ineffective	0	Eye symptom	0	Placebo effect	0	Foramen ovale	0	Angiotensin Converting Enzyme	0	Complications	0	Prophylactic treatment	0	Intervention	0	Subtype	0	Randomized controlled trial	0	Referral	0	Cranium	0	Temporal	0	Barbiturate	0	Septoplasty	0	nun	0	Eye pain	0	Angry	0	Emergency care	0	Food allergies	0	Motivation	0	spouse	0	Cadasil	0	5HT	0
Advantage	0	Attitude	0	Antihistamine	0	Lifestyle change	0	Nerve block	0	Accepting	0	Lidocaine	0	Pain management	1	Menstrual migraine	0	Father	0	Spectrum	0	Grandfather	0	Botulinum Toxin	0	Double vision	0	Intracranial hypertension	0	church	0	Medication overuse	0	Empower	0	Suicide	0	Trigeminal autonomic cephalalgia	0	Secondary headache	0	Consultation	0	Progression	0	Beta-blocker	0	Melatonin	0	Episodic	0	Referred pain	0	Shoulder muscle	0	Detoxification	0	Abnormality	0	Beta-Blockers	0	5-HT	0	Ineffective	0	Eye symptom	0	Placebo effect	0	Foramen ovale	0	Angiotensin Converting Enzyme	0	Complications	0	Prophylactic treatment	0	Intervention	0	Subtype	0	Randomized controlled trial	0	Referral	0	Cranium	0	Temporal	0	Barbiturate	0	Septoplasty	0	nun	0	Eye pain	0	Angry	0	Emergency care	0	Food allergies	0	Motivation	0	spouse	0	Cadasil	0	5HT	0
Advantage	0	Attitude	0	Antihistamine	0	Lifestyle change	0	Nerve block	0	Accepting	0	Lidocaine	0	Pain management	0	Menstrual migraine	0	Father	0	Spectrum	0	Grandfather	0	Botulinum Toxin	0	Double vision	0	Intracranial hypertension	1	church	0	Medication overuse	0	Empower	0	Suicide	0	Trigeminal autonomic cephalalgia	0	Secondary headache	0	Consultation	0	Progression	0	Beta-blocker	0	Melatonin	0	Episodic	0	Referred pain	0	Shoulder muscle	0	Detoxification	0	Abnormality	0	Beta-Blockers	0	5-HT	0	Ineffective	0	Eye symptom	0	Placebo effect	0	Foramen ovale	0	Angiotensin Converting Enzyme	0	Complications	0	Prophylactic treatment	0	Intervention	0	Subtype	0	Randomized controlled trial	0	Referral	0	Cranium	0	Temporal	0	Barbiturate	0	Septoplasty	0	nun	0	Eye pain	0	Angry	0	Emergency care	0	Food allergies	0	Motivation	0	spouse	0	Cadasil	0	5HT	0
Advantage	0	Attitude	0	Antihistamine	0	Lifestyle change	0	Nerve block	0	Accepting	0	Lidocaine	0	Pain management	0	Menstrual migraine	0	Father	0	Spectrum	0	Grandfather	0	Botulinum Toxin	0	Double vision	0	Intracranial hypertension	0	church	0	Medication overuse	0	Empower	0	Suicide	0	Trigeminal autonomic cephalalgia	0	Secondary headache	0	Consultation	0	Progression	0	Beta-blocker	0	Melatonin	0	Episodic	0	Referred pain	0	Shoulder muscle	0	Detoxification	0	Abnormality	0	Beta-Blockers	0	5-HT	0	Ineffective	0	Eye symptom	0	Placebo effect	0	Foramen ovale	0	Angiotensin Converting Enzyme	0	Complications	0	Prophylactic treatment	0	Intervention	0	Subtype	0	Randomized controlled trial	0	Referral	0	Cranium	0	Temporal	0	Barbiturate	0	Septoplasty	0	nun	0	Eye pain	0	Angry	0	Emergency care	0	Food allergies	0	Motivation	0	spouse	0	Cadasil	0	5HT	0
Advantage	0	Attitude	0	Antihistamine	0	Lifestyle change	0	Nerve block	0	Accepting	0	Lidocaine	0	Pain management	0	Menstrual migraine	0	Father	0	Spectrum	0	Grandfather	0	Botulinum Toxin	0	Double vision	0	Intracranial hypertension	0	church	0	Medication overuse	0	Empower	0	Suicide	0	Trigeminal autonomic cephalalgia	0	Secondary headache	0	Consultation	0	Progression	0	Beta-blocker	0	Melatonin	0	Episodic	0	Referred pain	0	Shoulder muscle	0	Detoxification	0	Abnormality	0	Beta-Blockers	0	5-HT	0	Ineffective	0	Eye symptom	0	Placebo effect	0	Foramen ovale	0	Angiotensin Converting Enzyme	0	Complications	0	Prophylactic treatment	0	Intervention	0	Subtype	0	Randomized controlled trial	0	Referral	0	Cranium	0	Temporal	0	Barbiturate	0	Septoplasty	0	nun	0	Eye pain	0	Angry	0	Emergency care	0	Food allergies	0	Motivation	0	spouse	0	Cadasil	0	5HT	0
Advantage	0	Attitude	0	Antihistamine	0	Lifestyle change	0	Nerve block	0	Accepting	0	Lidocaine	0	Pain management	0	Menstrual migraine	0	Father	0	Spectrum	0	Grandfather	0	Botulinum Toxin	0	Double vision	0	Intracranial hypertension	0	church	0	Medication overuse	0	Empower	0	Suicide	0	Trigeminal autonomic cephalalgia	0	Secondary headache	0	Consultation	0	Progression	0	Beta-blocker	0	Melatonin	0	Episodic	0	Referred pain	0	Shoulder muscle	0	Detoxification	0	Abnormality	0	Beta-Blockers	0	5-HT	0	Ineffective	0	Eye symptom	0	Placebo effect	0	Foramen ovale	0	Angiotensin Converting Enzyme	0	Complications	0	Prophylactic treatment	0	Intervention	0	Subtype	0	Randomized controlled trial	0	Referral	0	Cranium	0	Temporal	0	Barbiturate	0	Septoplasty	0	nun	0	Eye pain	0	Angry	0	Emergency care	0	Food allergies	0	Motivation	0	spouse	0	Cadasil	0	5HT	0
Advantage	0	Attitude	0	Antihistamine	0	Lifestyle change	0	Nerve block	0	Accepting	0	Lidocaine	0	Pain management	0	Menstrual migraine	0	Father	0	Spectrum	0	Grandfather	0	Botulinum Toxin	0	Double vision	0	Intracranial hypertension	0	church	0	Medication overuse	0	Empower	0	Suicide	0	Trigeminal autonomic cephalalgia	0	Secondary headache	0	Consultation	0	Progression	0	Beta-blocker	0	Melatonin	0	Episodic	0	Referred pain	0	Shoulder muscle	0	Detoxification	0	Abnormality	0	Beta-Blockers	0	5-HT	0	Ineffective	0	Eye symptom	0	Placebo effect	0	Foramen ovale	0	Angiotensin Converting Enzyme	0	Complications	0	Prophylactic treatment	0	Intervention	0	Subtype	0	Randomized controlled trial	0	Referral	1	Cranium	0	Temporal	0	Barbiturate	0	Septoplasty	0	nun	0	Eye pain	0	Angry	0	Emergency care	0	Food allergies	0	Motivation	0	spouse	0	Cadasil	0	5HT	0
Advantage	0	Attitude	0	Antihistamine	0	Lifestyle change	0	Nerve block	0	Accepting	0	Lidocaine	0	Pain management	0	Menstrual migraine	0	Father	0	Spectrum	0	Grandfather	0	Botulinum Toxin	0	Double vision	0	Intracranial hypertension	0	church	0	Medication overuse	0	Empower	0	Suicide	0	Trigeminal autonomic cephalalgia	0	Secondary headache	0	Consultation	0	Progression	0	Beta-blocker	0	Melatonin	0	Episodic	0	Referred pain	0	Shoulder muscle	0	Detoxification	0	Abnormality	0	Beta-Blockers	0	5-HT	0	Ineffective	0	Eye symptom	0	Placebo effect	0	Foramen ovale	0	Angiotensin Converting Enzyme	0	Complications	0	Prophylactic treatment	0	Intervention	0	Subtype	0	Randomized controlled trial	0	Referral	0	Cranium	0	Temporal	0	Barbiturate	0	Septoplasty	0	nun	0	Eye pain	0	Angry	0	Emergency care	0	Food allergies	0	Motivation	0	spouse	0	Cadasil	0	5HT	0
Advantage	0	Attitude	1	Antihistamine	0	Lifestyle change	0	Nerve block	0	Accepting	0	Lidocaine	0	Pain management	0	Menstrual migraine	0	Father	0	Spectrum	0	Grandfather	0	Botulinum Toxin	0	Double vision	0	Intracranial hypertension	0	church	0	Medication overuse	0	Empower	0	Suicide	0	Trigeminal autonomic cephalalgia	0	Secondary headache	0	Consultation	0	Progression	0	Beta-blocker	0	Melatonin	0	Episodic	0	Referred pain	0	Shoulder muscle	0	Detoxification	0	Abnormality	0	Beta-Blockers	0	5-HT	0	Ineffective	0	Eye symptom	0	Placebo effect	0	Foramen ovale	0	Angiotensin Converting Enzyme	0	Complications	0	Prophylactic treatment	0	Intervention	0	Subtype	0	Randomized controlled trial	0	Referral	0	Cranium	0	Temporal	0	Barbiturate	0	Septoplasty	0	nun	0	Eye pain	0	Angry	0	Emergency care	0	Food allergies	0	Motivation	0	spouse	0	Cadasil	0	5HT	0
Advantage	2	Attitude	0	Antihistamine	1	Lifestyle change	0	Nerve block	0	Accepting	0	Lidocaine	0	Pain management	0	Menstrual migraine	0	Father	0	Spectrum	0	Grandfather	0	Botulinum Toxin	1	Double vision	0	Intracranial hypertension	0	church	0	Medication overuse	0	Empower	0	Suicide	0	Trigeminal autonomic cephalalgia	0	Secondary headache	0	Consultation	0	Progression	1	Beta-blocker	0	Melatonin	1	Episodic	0	Referred pain	0	Shoulder muscle	0	Detoxification	0	Abnormality	0	Beta-Blockers	0	5-HT	0	Ineffective	0	Eye symptom	0	Placebo effect	0	Foramen ovale	1	Angiotensin Converting Enzyme	1	Complications	1	Prophylactic treatment	1	Intervention	1	Subtype	1	Randomized controlled trial	1	Referral	0	Cranium	0	Temporal	0	Barbiturate	0	Septoplasty	0	nun	0	Eye pain	0	Angry	0	Emergency care	0	Food allergies	0	Motivation	0	spouse	0	Cadasil	0	5HT	0
Advantage	0	Attitude	0	Antihistamine	0	Lifestyle change	0	Nerve block	0	Accepting	0	Lidocaine	0	Pain management	0	Menstrual migraine	0	Father	0	Spectrum	0	Grandfather	0	Botulinum Toxin	0	Double vision	0	Intracranial hypertension	0	church	0	Medication overuse	0	Empower	0	Suicide	0	Trigeminal autonomic cephalalgia	0	Secondary headache	0	Consultation	0	Progression	0	Beta-blocker	0	Melatonin	0	Episodic	0	Referred pain	0	Shoulder muscle	0	Detoxification	0	Abnormality	0	Beta-Blockers	0	5-HT	0	Ineffective	0	Eye symptom	0	Placebo effect	0	Foramen ovale	0	Angiotensin Converting Enzyme	0	Complications	0	Prophylactic treatment	0	Intervention	0	Subtype	0	Randomized controlled trial	0	Referral	0	Cranium	0	Temporal	0	Barbiturate	0	Septoplasty	0	nun	0	Eye pain	0	Angry	0	Emergency care	0	Food allergies	0	Motivation	0	spouse	0	Cadasil	0	5HT	0
Advantage	0	Attitude	0	Antihistamine	0	Lifestyle change	0	Nerve block	0	Accepting	0	Lidocaine	0	Pain management	0	Menstrual migraine	0	Father	0	Spectrum	0	Grandfather	0	Botulinum Toxin	0	Double vision	0	Intracranial hypertension	0	church	0	Medication overuse	0	Empower	0	Suicide	0	Trigeminal autonomic cephalalgia	0	Secondary headache	0	Consultation	0	Progression	0	Beta-blocker	0	Melatonin	0	Episodic	0	Referred pain	0	Shoulder muscle	0	Detoxification	0	Abnormality	0	Beta-Blockers	0	5-HT	0	Ineffective	0	Eye symptom	0	Placebo effect	1	Foramen ovale	0	Angiotensin Converting Enzyme	0	Complications	0	Prophylactic treatment	0	Intervention	0	Subtype	0	Randomized controlled trial	0	Referral	0	Cranium	0	Temporal	0	Barbiturate	0	Septoplasty	0	nun	0	Eye pain	0	Angry	0	Emergency care	0	Food allergies	0	Motivation	0	spouse	0	Cadasil	0	5HT	0
Advantage	0	Attitude	1	Antihistamine	0	Lifestyle change	0	Nerve block	0	Accepting	0	Lidocaine	0	Pain management	0	Menstrual migraine	0	Father	0	Spectrum	0	Grandfather	0	Botulinum Toxin	0	Double vision	0	Intracranial hypertension	0	church	0	Medication overuse	0	Empower	1	Suicide	0	Trigeminal autonomic cephalalgia	0	Secondary headache	0	Consultation	0	Progression	0	Beta-blocker	0	Melatonin	0	Episodic	0	Referred pain	0	Shoulder muscle	0	Detoxification	0	Abnormality	0	Beta-Blockers	0	5-HT	0	Ineffective	0	Eye symptom	0	Placebo effect	0	Foramen ovale	0	Angiotensin Converting Enzyme	0	Complications	0	Prophylactic treatment	0	Intervention	0	Subtype	0	Randomized controlled trial	0	Referral	0	Cranium	0	Temporal	0	Barbiturate	0	Septoplasty	0	nun	0	Eye pain	0	Angry	0	Emergency care	0	Food allergies	0	Motivation	0	spouse	0	Cadasil	0	5HT	0
Advantage	0	Attitude	0	Antihistamine	0	Lifestyle change	0	Nerve block	0	Accepting	0	Lidocaine	1	Pain management	0	Menstrual migraine	0	Father	0	Spectrum	0	Grandfather	0	Botulinum Toxin	0	Double vision	0	Intracranial hypertension	0	church	0	Medication overuse	0	Empower	0	Suicide	0	Trigeminal autonomic cephalalgia	0	Secondary headache	0	Consultation	0	Progression	0	Beta-blocker	0	Melatonin	0	Episodic	0	Referred pain	0	Shoulder muscle	0	Detoxification	0	Abnormality	0	Beta-Blockers	0	5-HT	0	Ineffective	0	Eye symptom	0	Placebo effect	0	Foramen ovale	0	Angiotensin Converting Enzyme	0	Complications	0	Prophylactic treatment	0	Intervention	0	Subtype	0	Randomized controlled trial	0	Referral	0	Cranium	0	Temporal	0	Barbiturate	0	Septoplasty	0	nun	0	Eye pain	0	Angry	0	Emergency care	0	Food allergies	0	Motivation	0	spouse	0	Cadasil	0	5HT	0
Advantage	0	Attitude	0	Antihistamine	0	Lifestyle change	0	Nerve block	0	Accepting	0	Lidocaine	0	Pain management	0	Menstrual migraine	0	Father	0	Spectrum	0	Grandfather	0	Botulinum Toxin	0	Double vision	0	Intracranial hypertension	0	church	0	Medication overuse	0	Empower	0	Suicide	0	Trigeminal autonomic cephalalgia	0	Secondary headache	0	Consultation	0	Progression	0	Beta-blocker	0	Melatonin	0	Episodic	0	Referred pain	0	Shoulder muscle	0	Detoxification	0	Abnormality	0	Beta-Blockers	0	5-HT	0	Ineffective	0	Eye symptom	0	Placebo effect	0	Foramen ovale	0	Angiotensin Converting Enzyme	0	Complications	0	Prophylactic treatment	0	Intervention	0	Subtype	0	Randomized controlled trial	0	Referral	0	Cranium	0	Temporal	0	Barbiturate	0	Septoplasty	0	nun	0	Eye pain	0	Angry	0	Emergency care	0	Food allergies	0	Motivation	0	spouse	0	Cadasil	0	5HT	0
Advantage	0	Attitude	0	Antihistamine	0	Lifestyle change	0	Nerve block	0	Accepting	0	Lidocaine	0	Pain management	0	Menstrual migraine	0	Father	0	Spectrum	0	Grandfather	0	Botulinum Toxin	0	Double vision	0	Intracranial hypertension	0	church	1	Medication overuse	0	Empower	0	Suicide	0	Trigeminal autonomic cephalalgia	0	Secondary headache	0	Consultation	0	Progression	0	Beta-blocker	0	Melatonin	0	Episodic	0	Referred pain	0	Shoulder muscle	0	Detoxification	0	Abnormality	0	Beta-Blockers	0	5-HT	1	Ineffective	1	Eye symptom	1	Placebo effect	0	Foramen ovale	0	Angiotensin Converting Enzyme	0	Complications	0	Prophylactic treatment	0	Intervention	0	Subtype	0	Randomized controlled trial	0	Referral	0	Cranium	0	Temporal	0	Barbiturate	0	Septoplasty	0	nun	0	Eye pain	0	Angry	0	Emergency care	0	Food allergies	0	Motivation	0	spouse	0	Cadasil	0	5HT	0
Advantage	0	Attitude	0	Antihistamine	0	Lifestyle change	0	Nerve block	0	Accepting	0	Lidocaine	0	Pain management	0	Menstrual migraine	0	Father	0	Spectrum	0	Grandfather	0	Botulinum Toxin	0	Double vision	0	Intracranial hypertension	0	church	0	Medication overuse	0	Empower	0	Suicide	0	Trigeminal autonomic cephalalgia	0	Secondary headache	0	Consultation	0	Progression	0	Beta-blocker	0	Melatonin	0	Episodic	0	Referred pain	0	Shoulder muscle	0	Detoxification	0	Abnormality	0	Beta-Blockers	0	5-HT	0	Ineffective	0	Eye symptom	0	Placebo effect	0	Foramen ovale	0	Angiotensin Converting Enzyme	0	Complications	0	Prophylactic treatment	0	Intervention	0	Subtype	0	Randomized controlled trial	0	Referral	0	Cranium	0	Temporal	0	Barbiturate	0	Septoplasty	0	nun	0	Eye pain	0	Angry	0	Emergency care	0	Food allergies	0	Motivation	0	spouse	0	Cadasil	0	5HT	0
Advantage	0	Attitude	0	Antihistamine	0	Lifestyle change	0	Nerve block	0	Accepting	0	Lidocaine	0	Pain management	0	Menstrual migraine	0	Father	0	Spectrum	0	Grandfather	0	Botulinum Toxin	0	Double vision	1	Intracranial hypertension	0	church	0	Medication overuse	0	Empower	0	Suicide	0	Trigeminal autonomic cephalalgia	0	Secondary headache	0	Consultation	0	Progression	0	Beta-blocker	1	Melatonin	0	Episodic	0	Referred pain	0	Shoulder muscle	0	Detoxification	0	Abnormality	0	Beta-Blockers	1	5-HT	0	Ineffective	0	Eye symptom	0	Placebo effect	0	Foramen ovale	0	Angiotensin Converting Enzyme	0	Complications	0	Prophylactic treatment	0	Intervention	0	Subtype	0	Randomized controlled trial	0	Referral	0	Cranium	0	Temporal	0	Barbiturate	0	Septoplasty	0	nun	0	Eye pain	0	Angry	0	Emergency care	0	Food allergies	0	Motivation	0	spouse	0	Cadasil	0	5HT	0
Advantage	0	Attitude	0	Antihistamine	0	Lifestyle change	0	Nerve block	0	Accepting	0	Lidocaine	0	Pain management	0	Menstrual migraine	0	Father	0	Spectrum	0	Grandfather	0	Botulinum Toxin	0	Double vision	0	Intracranial hypertension	0	church	0	Medication overuse	0	Empower	0	Suicide	0	Trigeminal autonomic cephalalgia	0	Secondary headache	0	Consultation	0	Progression	0	Beta-blocker	0	Melatonin	0	Episodic	0	Referred pain	0	Shoulder muscle	1	Detoxification	1	Abnormality	1	Beta-Blockers	0	5-HT	0	Ineffective	0	Eye symptom	0	Placebo effect	0	Foramen ovale	0	Angiotensin Converting Enzyme	0	Complications	0	Prophylactic treatment	0	Intervention	0	Subtype	0	Randomized controlled trial	0	Referral	0	Cranium	0	Temporal	0	Barbiturate	0	Septoplasty	0	nun	0	Eye pain	0	Angry	0	Emergency care	0	Food allergies	0	Motivation	0	spouse	0	Cadasil	0	5HT	0
Advantage	0	Attitude	0	Antihistamine	0	Lifestyle change	0	Nerve block	0	Accepting	0	Lidocaine	0	Pain management	0	Menstrual migraine	0	Father	0	Spectrum	0	Grandfather	0	Botulinum Toxin	0	Double vision	0	Intracranial hypertension	0	church	0	Medication overuse	0	Empower	0	Suicide	0	Trigeminal autonomic cephalalgia	0	Secondary headache	0	Consultation	0	Progression	0	Beta-blocker	0	Melatonin	0	Episodic	0	Referred pain	0	Shoulder muscle	0	Detoxification	0	Abnormality	0	Beta-Blockers	0	5-HT	0	Ineffective	0	Eye symptom	0	Placebo effect	0	Foramen ovale	0	Angiotensin Converting Enzyme	0	Complications	0	Prophylactic treatment	0	Intervention	0	Subtype	0	Randomized controlled trial	0	Referral	0	Cranium	0	Temporal	0	Barbiturate	0	Septoplasty	0	nun	0	Eye pain	0	Angry	0	Emergency care	0	Food allergies	0	Motivation	0	spouse	0	Cadasil	0	5HT	0
Advantage	0	Attitude	0	Antihistamine	0	Lifestyle change	0	Nerve block	0	Accepting	0	Lidocaine	0	Pain management	0	Menstrual migraine	1	Father	0	Spectrum	0	Grandfather	0	Botulinum Toxin	0	Double vision	0	Intracranial hypertension	0	church	0	Medication overuse	0	Empower	0	Suicide	0	Trigeminal autonomic cephalalgia	0	Secondary headache	0	Consultation	0	Progression	0	Beta-blocker	0	Melatonin	0	Episodic	0	Referred pain	0	Shoulder muscle	0	Detoxification	0	Abnormality	0	Beta-Blockers	0	5-HT	0	Ineffective	0	Eye symptom	0	Placebo effect	0	Foramen ovale	0	Angiotensin Converting Enzyme	0	Complications	0	Prophylactic treatment	0	Intervention	0	Subtype	0	Randomized controlled trial	0	Referral	0	Cranium	0	Temporal	0	Barbiturate	0	Septoplasty	0	nun	0	Eye pain	0	Angry	0	Emergency care	0	Food allergies	0	Motivation	0	spouse	0	Cadasil	0	5HT	0
Advantage	0	Attitude	0	Antihistamine	0	Lifestyle change	0	Nerve block	0	Accepting	0	Lidocaine	0	Pain management	0	Menstrual migraine	0	Father	0	Spectrum	0	Grandfather	0	Botulinum Toxin	0	Double vision	0	Intracranial hypertension	0	church	0	Medication overuse	0	Empower	0	Suicide	0	Trigeminal autonomic cephalalgia	0	Secondary headache	0	Consultation	0	Progression	0	Beta-blocker	0	Melatonin	0	Episodic	0	Referred pain	1	Shoulder muscle	0	Detoxification	0	Abnormality	0	Beta-Blockers	0	5-HT	0	Ineffective	0	Eye symptom	0	Placebo effect	0	Foramen ovale	0	Angiotensin Converting Enzyme	0	Complications	0	Prophylactic treatment	0	Intervention	0	Subtype	0	Randomized controlled trial	0	Referral	0	Cranium	0	Temporal	0	Barbiturate	0	Septoplasty	0	nun	0	Eye pain	0	Angry	0	Emergency care	0	Food allergies	0	Motivation	0	spouse	0	Cadasil	0	5HT	0
	2		2		2		2		2		2		2		2		2		2		2		2		2		2		2		2		2		2		2		2		2		2		2		2		2		2		1		1		1		1		1		1		1		1		1		1		1		1		1		1		1		1		1		1		1		1		1		1		1		1		1		1		1		1		1		1

[illegible]

May 2019 Migraine Webcrawl Data

Condition	URL Id	URL	Total number of words
Migraine	2293278	http://migrainetalk.forumotion.co.uk/	923
Migraine	2292659	http://www.migraine.org.uk/get-advice/	440
Migraine	2244789	http://www.topix.com/forum/health/migraine	1794
Migraine	2295373	https://cassiopaea.org/forum/?topic=17971.0	2196
Migraine	2285137	https://ergoweb.com/forums/topic/treatment-of-light-induced-migraine-headaches-in-the-office/	1236
Migraine	2296496	https://exchanges.webmd.com/migraines-and-headaches-exchange	6384
Migraine	2282154	https://forum.grasscity.com/threads/migraines.453628/	2514
Migraine	2283242	https://www.chronofhorse.com/forum/archive/index.php/t-318339.html	5948
Migraine	2294417	https://www.dinet.org/forums/topic/27868-anybody-else-have-an-intractable-migraine/	2140
Migraine	2291744	https://www.physicsforums.com/threads/the-four-stages-of-migraine.305792/	1946
			25521

URL Id	P0a	P0b	P1a	P1b	P2a	P2b	P3a	P3b	P4a	P4b	P5a	P5b	P6a	P6b	P7a	P7b	P8a	P8b	P9a	P9b	P10a	P10b	P11a	P11b
2293278	Migraine	15	Ache	0	Ill	0	Pain	0	Son	3	work	0	Doctor	0	Trigger	1	Symptom	0	Treatment	0	Chronic	3	suffer	4
2292659	Migraine	9	Ache	0	Ill	0	Pain	0	Son	1	work	0	Doctor	0	Trigger	0	Symptom	0	Treatment	1	Chronic	0	suffer	1
2244789	Migraine	24	Ache	5	Ill	4	Pain	3	Son	0	work	0	Doctor	2	Trigger	2	Symptom	1	Treatment	2	Chronic	4	suffer	1
2295373	Migraine	0	Ache	0	Ill	4	Pain	0	Son	3	work	2	Doctor	0	Trigger	2	Symptom	0	Treatment	0	Chronic	0	suffer	0
2285137	Migraine	11	Ache	6	Ill	4	Pain	0	Son	2	work	6	Doctor	0	Trigger	2	Symptom	2	Treatment	2	Chronic	0	suffer	1
2296496	Migraine	80	Ache	56	Ill	27	Pain	16	Son	10	work	6	Doctor	25	Trigger	2	Symptom	26	Treatment	16	Chronic	11	suffer	11
2282154	Migraine	47	Ache	14	Ill	9	Pain	8	Son	3	work	3	Doctor	3	Trigger	1	Symptom	3	Treatment	5	Chronic	4	suffer	0
2283242	Migraine	38	Ache	12	Ill	32	Pain	9	Son	11	work	12	Doctor	3	Trigger	22	Symptom	1	Treatment	0	Chronic	5	suffer	4
2294417	Migraine	21	Ache	11	Ill	6	Pain	16	Son	4	work	8	Doctor	6	Trigger	1	Symptom	0	Treatment	7	Chronic	2	suffer	1
2291744	Migraine	63	Ache	24	Ill	3	Pain	2	Son	7	work	4	Doctor	2	Trigger	8	Symptom	4	Treatment	0	Chronic	0	suffer	6
		308		128		89		54		44		41		41		41		37		33		29		29

URL Id	P12a	P12b	P13a	P13b	P14a	P14b	P15a	P15b	P16a	P16b	P17a	P17b	P18a	P18b	P19a	P19b	P20a	P20b	P21a	P21b	P22a	P22b	P23a
2293278	Gene	3	Nose	0	Medication	0	ACE	2	Stress	0	Chronic migraine	3	Good	0	good	0	Eye	0	drug	0	Medical	1	Bad
2292659	Gene	0	Nose	0	Medication	0	ACE	0	Stress	0	Chronic migraine	0	Good	0	good	0	Eye	0	drug	0	Medical	0	Bad
2244789	Gene	2	Nose	0	Medication	0	ACE	3	Stress	0	Chronic migraine	4	Good	1	good	1	Eye	1	drug	5	Medical	0	Bad
2295373	Gene	0	Nose	0	Medication	0	ACE	3	Stress	1	Chronic migraine	0	Good	2	good	2	Eye	0	drug	0	Medical	0	Bad
2285137	Gene	2	Nose	1	Medication	0	ACE	1	Stress	0	Chronic migraine	0	Good	0	good	0	Eye	10	drug	0	Medical	1	Bad
2296496	Gene	0	Nose	12	Medication	12	ACE	4	Stress	10	Chronic migraine	8	Good	5	good	5	Eye	2	drug	7	Medical	11	Bad
2282154	Gene	2	Nose	5	Medication	0	ACE	0	Stress	0	Chronic migraine	4	Good	1	good	1	Eye	0	drug	4	Medical	0	Bad
2283242	Gene	13	Nose	0	Medication	1	ACE	6	Stress	9	Chronic migraine	0	Good	7	good	7	Eye	3	drug	1	Medical	2	Bad
2294417	Gene	1	Nose	5	Medication	12	ACE	3	Stress	0	Chronic migraine	1	Good	1	good	1	Eye	0	drug	0	Medical	2	Bad
2291744	Gene	5	Nose	4	Medication	0	ACE	0	Stress	1	Chronic migraine	0	Good	2	good	2	Eye	2	drug	0	Medical	0	Bad
		28		27		25		22		21		20		19		19		18		17		17	

P23b	P24a	P24b	P25a	P25b	P26a	P26b	P27a	P27b	P28a	P28b	P29a	P29b	P30a	P30b	P31a	P31b	P32a	P32b	P33a	P33b	P34a	P34b	P35a	P35b	P36a	P36b	P37a	P37b	P38a	P38b	P39a	P39b	P40a	P40b	P41a	P41b	P42a	P42b	P43a	P43b	P44a	P44b	P45a	P45b	P46a	P46b	P47a	P47b	P48a	P48b	P49a	P49b	P50a	P50b	P51a	P51b	P52a	P52b	P53a	P53b	P54a
0	bad	0	Relief	0	Botox	0	best	0	past	0	Hormone	0	Aura	0	Friend	0	Current	1	Clinic	0	Diet	0	medicine	0	Thought	0	manage	0	Intractable	0	Cluster	0	Support group	0	Onset	0	Nausea	0	Parent	0	nasal	0	Nerve	0	Coping	0	Concussion	0	Daughter	0	Sick	0	Genetic	0	ill	0	Drugs	0	Cluster headache	0	Present
0	bad	0	Relief	0	Botox	0	best	0	past	0	Hormone	0	Aura	0	Friend	2	Current	0	Clinic	0	Diet	0	medicine	0	Thought	0	manage	1	Intractable	0	Cluster	0	Support group	0	Onset	0	Nausea	0	Parent	0	nasal	0	Nerve	0	Coping	0	Concussion	0	Daughter	0	Sick	0	Genetic	0	ill	0	Drugs	0	Cluster headache	0	Present
0	bad	0	Relief	0	Botox	2	best	0	past	0	Hormone	0	Aura	0	Friend	0	Current	0	Clinic	0	Diet	0	medicine	2	Thought	1	manage	1	Intractable	0	Cluster	0	Support group	0	Onset	0	Nausea	0	Parent	0	nasal	1	Nerve	1	Coping	0	Concussion	0	Daughter	0	Sick	0	Genetic	0	ill	0	Drugs	2	Cluster headache	0	Present
0	bad	0	Relief	0	Botox	0	best	0	past	0	Hormone	0	Aura	0	Friend	1	Current	1	Clinic	0	Diet	1	medicine	0	Thought	0	manage	0	Intractable	0	Cluster	0	Support group	0	Onset	0	Nausea	0	Parent	0	nasal	0	Nerve	0	Coping	0	Concussion	0	Daughter	0	Sick	0	Genetic	0	ill	1	Drugs	0	Cluster headache	0	Present
0	bad	0	Relief	0	Botox	0	best	0	past	0	Hormone	1	Aura	0	Friend	0	Current	1	Clinic	0	Diet	0	medicine	0	Thought	0	manage	2	Intractable	0	Cluster	0	Support group	0	Onset	0	Nausea	1	Parent	1	nasal	0	Nerve	0	Coping	0	Concussion	0	Daughter	0	Sick	1	Genetic	1	ill	0	Drugs	0	Cluster headache	0	Present
3	bad	3	Relief	8	Botox	5	best	4	past	4	Hormone	6	Aura	1	Friend	4	Current	0	Clinic	0	Diet	0	medicine	3	Thought	4	manage	2	Intractable	0	Cluster	1	Support group	6	Onset	1	Nausea	1	Parent	0	nasal	0	Nerve	0	Coping	3	Concussion	5	Daughter	2	Sick	0	Genetic	0	ill	0	Drugs	2	Cluster headache	1	Present
6	bad	6	Relief	3	Botox	0	best	3	past	0	Hormone	0	Aura	0	Friend	0	Current	0	Clinic	2	Diet	2	medicine	1	Thought	0	manage	0	Intractable	0	Cluster	4	Support group	0	Onset	1	Nausea	0	Parent	3	nasal	0	Nerve	0	Coping	0	Concussion	0	Daughter	0	Sick	0	Genetic	0	ill	3	Drugs	0	Cluster headache	2	Present
6	bad	6	Relief	1	Botox	2	best	4	past	7	Hormone	4	Aura	1	Friend	0	Current	5	Clinic	5	Diet	5	medicine	1	Thought	2	manage	1	Intractable	0	Cluster	0	Support group	0	Onset	2	Nausea	3	Parent	0	nasal	0	Nerve	1	Coping	2	Concussion	0	Daughter	0	Sick	4	Genetic	1	ill	1	Drugs	1	Cluster headache	0	Present
1	bad	1	Relief	1	Botox	3	best	0	past	0	Hormone	0	Aura	2	Friend	2	Current	0	Clinic	0	Diet	0	medicine	1	Thought	0	manage	0	Intractable	6	Cluster	1	Support group	0	Onset	0	Nausea	0	Parent	0	nasal	1	Nerve	4	Coping	0	Concussion	0	Daughter	3	Sick	0	Genetic	0	ill	0	Drugs	0	Cluster headache	1	Present
0	bad	0	Relief	0	Botox	0	best	0	past	0	Hormone	0	Aura	6	Friend	1	Current	1	Clinic	1	Diet	0	medicine	0	Thought	0	manage	0	Intractable	0	Cluster	0	Support group	0	Onset	2	Nausea	1	Parent	2	nasal	4	Nerve	0	Coping	0	Concussion	0	Daughter	0	Sick	0	Genetic	3	ill	0	Drugs	0	Cluster headache	0	Present
16		16		13		12		11		11		11		10		10		9		8		8		8		7		7		6		6		6		6		6		6		6		6		5		5		5		5		5		5		5		4	

P54b	P55a	P55b	P56a	P56b	P57a	P57b	P58a	P58b	P59a	P59b	P60a	P60b	P61a	P61b	P62a	P62b	P63a	P63b	P64a	P64b	P65a	P65b	P66a	P66b	P67a	P67b	P68a	P68b	P69a	P69b	P70a	P70b	P71a	P71b	P72a	P72b	P73a	P73b	P74a	P74b	P75a	P75b	P76a	P76b	P77a	P77b	P78a	P78b	P79a	P79b	P80a	P80b	P81a	P81b	P82a
0	Diagnosis	0	Tension	0	Surgery	0	Prescription	0	employ	0	Frequency	0	Wife	0	Mother	0	Migraine attack	0	Blocker	0	Facial pain	0	Suicide	0	Menopause	0	Therapy	0	Pregnancy	0	Sister	0	worst	0	Accident	0	History	0	Happy	0	Serotonin	0	Calcium channel	0	Injection	0	Calcium channel blocker	0	Stroke	0	vacation	0	exercise	0	Magnesium
0	Diagnosis	0	Tension	0	Surgery	0	Prescription	0	employ	0	Frequency	0	Wife	0	Mother	0	Migraine attack	0	Blocker	0	Facial pain	0	Suicide	0	Menopause	0	Therapy	0	Pregnancy	0	Sister	0	worst	0	Accident	0	History	0	Happy	0	Serotonin	0	Calcium channel	0	Injection	0	Calcium channel blocker	0	Stroke	0	vacation	0	exercise	0	Magnesium
0	Diagnosis	0	Tension	0	Surgery	0	Prescription	0	employ	0	Frequency	1	Wife	1	Mother	1	Migraine attack	0	Blocker	0	Facial pain	0	Suicide	0	Menopause	0	Therapy	0	Pregnancy	0	Sister	0	worst	0	Accident	0	History	0	Happy	0	Serotonin	1	Calcium channel	0	Injection	0	Calcium channel blocker	0	Stroke	0	vacation	0	exercise	0	Magnesium
0	Diagnosis	0	Tension	0	Surgery	0	Prescription	0	employ	0	Frequency	0	Wife	0	Mother	2	Migraine attack	0	Blocker	0	Facial pain	0	Suicide	0	Menopause	0	Therapy	0	Pregnancy	0	Sister	0	worst	0	Accident	1	History	3	Happy	2	Serotonin	0	Calcium channel	0	Injection	0	Calcium channel blocker	0	Stroke	0	vacation	0	exercise	0	Magnesium
0	Diagnosis	0	Tension	0	Surgery	0	Prescription	0	employ	4	Frequency	1	Wife	0	Mother	0	Migraine attack	0	Blocker	0	Facial pain	0	Suicide	0	Menopause	0	Therapy	0	Pregnancy	0	Sister	0	worst	0	Accident	0	History	0	Happy	0	Serotonin	0	Calcium channel	0	Injection	0	Calcium channel blocker	0	Stroke	0	vacation	0	exercise	0	Magnesium
2	Diagnosis	4	Tension	2	Surgery	4	Prescription	3	employ	0	Frequency	0	Wife	3	Mother	0	Migraine attack	0	Blocker	0	Facial pain	0	Suicide	0	Menopause	0	Therapy	0	Pregnancy	3	Sister	3	worst	1	Accident	1	History	0	Happy	1	Serotonin	0	Calcium channel	0	Injection	0	Calcium channel blocker	0	Stroke	0	vacation	0	exercise	0	Magnesium
0	Diagnosis	0	Tension	1	Surgery	0	Prescription	0	employ	0	Frequency	0	Wife	0	Mother	0	Migraine attack	0	Blocker	0	Facial pain	0	Suicide	0	Menopause	0	Therapy	1	Pregnancy	0	Sister	0	worst	2	Accident	0	History	0	Happy	0	Serotonin	2	Calcium channel	0	Injection	0	Calcium channel blocker	0	Stroke	0	vacation	0	exercise	0	Magnesium
1	Diagnosis	0	Tension	0	Surgery	0	Prescription	1	employ	0	Frequency	1	Wife	0	Mother	1	Migraine attack	0	Blocker	0	Facial pain	0	Suicide	3	Menopause	3	Therapy	0	Pregnancy	0	Sister	0	worst	0	Accident	1	History	0	Happy	0	Serotonin	0	Calcium channel	0	Injection	0	Calcium channel blocker	0	Stroke	2	vacation	2	exercise	2	Magnesium
1	Diagnosis	0	Tension	1	Surgery	0	Prescription	0	employ	0	Frequency	0	Wife	0	Mother	0	Migraine attack	0	Blocker	3	Facial pain	3	Suicide	0	Menopause	0	Therapy	1	Pregnancy	0	Sister	0	worst	0	Accident	0	History	0	Happy	0	Serotonin	0	Calcium channel	2	Injection	2	Calcium channel blocker	2	Stroke	0	vacation	0	exercise	0	Magnesium
0	Diagnosis	0	Tension	0	Surgery	0	Prescription	0	employ	0	Frequency	1	Wife	0	Mother	0	Migraine attack	3	Blocker	0	Facial pain	0	Suicide	0	Menopause	0	Therapy	1	Pregnancy	0	Sister	0	worst	0	Accident	0	History	0	Happy	0	Serotonin	0	Calcium channel	0	Injection	0	Calcium channel blocker	0	Stroke	0	vacation	0	exercise	0	Magnesium
4		4		4		4		4		4		4		4		4		3		3		3		3		3		3		3		3		3		3		3		3		3		2		2		2		2		2		2	

P82b	P83a	P83b	P84a	P84b	P85a	P85b	P86a	P86b	P87a	P87b	P88a	P88b	P89a	P89b	P90a	P90b	P91a	P91b	P92a	P92b	P93a	P93b	P94a	P94b	P95a	P95b	P96a	P96b	P97a	P97b	P98a	P98b	P99a	P99b	P100a	P100b	P101a	P101b	P102a	P102b	P103a	P103b	P104a	P104b	P105a	P105b	P106a	P106b	P107a	P107b	P108a	P108b	P109a	P109b	P110a
0	Exercise	0	Intracranial hypertension	0	nose	0	Nurse	0	travel	0	Emergency	0	Dopamine	0	Chronic pain	0	Cope	0	Child	0	Management	0	Abortive	0	Stages	0	Anatomy	0	minister	0	Education	0	Episodic	0	Double vision	0	faith	0	church	0	Father	0	Spectrum	0	ARB	0	Preemptive	0	Impact	0	Anger	0	Drive	0	Brother
0	Exercise	0	Intracranial hypertension	0	nose	0	Nurse	0	travel	0	Emergency	0	Dopamine	0	Chronic pain	0	Cope	0	Child	0	Management	0	Abortive	0	Stages	0	Anatomy	0	minister	0	Education	0	Episodic	0	Double vision	0	faith	0	church	0	Father	0	Spectrum	0	ARB	0	Preemptive	0	Impact	0	Anger	0	Drive	0	Brother
0	Exercise	0	Intracranial hypertension	0	nose	0	Nurse	0	travel	0	Emergency	0	Dopamine	0	Chronic pain	0	Cope	0	Child	0	Management	0	Abortive	1	Stages	0	Anatomy	0	minister	0	Education	0	Episodic	0	Double vision	0	faith	0	church	0	Father	0	Spectrum	0	ARB	0	Preemptive	0	Impact	0	Anger	0	Drive	0	Brother
0	Exercise	0	Intracranial hypertension	0	nose	0	Nurse	0	travel	0	Emergency	0	Dopamine	0	Chronic pain	0	Cope	0	Child	0	Management	0	Abortive	0	Stages	0	Anatomy	0	minister	0	Education	0	Episodic	0	Double vision	0	faith	0	church	0	Father	0	Spectrum	0	ARB	0	Preemptive	0	Impact	0	Anger	0	Drive	0	Brother
0	Exercise	0	Intracranial hypertension	0	nose	0	Nurse	0	travel	0	Emergency	0	Dopamine	0	Chronic pain	0	Cope	0	Child	0	Management	1	Abortive	0	Stages	0	Anatomy	0	minister	0	Education	0	Episodic	0	Double vision	0	faith	0	church	0	Father	0	Spectrum	0	ARB	0	Preemptive	0	Impact	0	Anger	0	Drive	0	Brother
0	Exercise	0	Intracranial hypertension	0	nose	0	Nurse	1	travel	1	Emergency	1	Dopamine	1	Chronic pain	1	Cope	1	Child	1	Management	1	Abortive	0	Stages	0	Anatomy	0	minister	0	Education	0	Episodic	0	Double vision	0	faith	0	church	0	Father	0	Spectrum	0	ARB	0	Preemptive	0	Impact	0	Anger	0	Drive	0	Brother
0	Exercise	0	Intracranial hypertension	1	nose	1	Nurse	0	travel	0	Emergency	1	Dopamine	0	Chronic pain	0	Cope	0	Child	0	Management	0	Abortive	0	Stages	0	Anatomy	0	minister	0	Education	0	Episodic	0	Double vision	0	faith	0	church	0	Father	0	Spectrum	0	ARB	0	Preemptive	0	Impact	0	Anger	0	Drive	0	Brother
1	Exercise	2	Intracranial hypertension	0	nose	0	Nurse	1	travel	0	Emergency	0	Dopamine	0	Chronic pain	1	Cope	1	Child	1	Management	0	Abortive	0	Stages	0	Anatomy	0	minister	0	Education	0	Episodic	0	Double vision	0	faith	0	church	0	Father	1	Spectrum	1	ARB	1	Preemptive	1	Impact	1	Anger	1	Drive	1	Brother
1	Exercise	0	Intracranial hypertension	1	nose	1	Nurse	0	travel	0	Emergency	0	Dopamine	1	Chronic pain	0	Cope	0	Child	0	Management	0	Abortive	1	Stages	0	Anatomy	0	minister	0	Education	0	Episodic	0	Double vision	1	faith	1	church	1	Father	0	Spectrum	0	ARB	0	Preemptive	0	Impact	0	Anger	0	Drive	0	Brother
0	Exercise	0	Intracranial hypertension	0	nose	0	Nurse	0	travel	1	Emergency	0	Dopamine	0	Chronic pain	0	Cope	0	Child	0	Management	0	Abortive	0	Stages	1	Anatomy	1	minister	1	Education	1	Episodic	1	Double vision	0	faith	0	church	0	Father	0	Spectrum	0	ARB	0	Preemptive	0	Impact	0	Anger	0	Drive	0	Brother

2 2 2 2 2 2 2 2 2 2 2 2 2 1 1 1 1 1 1 1 1 1 1 1 1 1 1 1

P110b	P111a	P111b	P112a	P112b	P113a	P113b	P114a	P114b	P115a	P115b	P116a	P116b	P117a	P117b	P118a	P118b	P119a	P119b	P120a	P120b	P121a	P121b	P122a	P122b	P123a	P123b	P124a	P124b	P125a	P125b	P126a	P126b	P127a	P127b	P128a	P128b	P129a	P129b	P130a	P130b	P131a	P131b	P132a	P132b	P133a	P133b	P134a	P134b
0	Grandfather	0	Adherence	0	husband	0	Anti-inflammatory	0	Future	0	Aggravate	0	CAT scan	0	Toxin	0	Depression	0	regime	0	Temporal	0	Agonist	0	Regimen	0	Botulinum Toxin	0	surgery	0	Patterns	0	Population	0	Assessment	0	Irk	0	Triptan	0	Serotonin receptor	0	pray	0	carer	1	Shock	1
0	Grandfather	0	Adherence	0	husband	0	Anti-inflammatory	0	Future	0	Aggravate	0	CAT scan	0	Toxin	0	Depression	0	regime	0	Temporal	0	Agonist	0	Regimen	0	Botulinum Toxin	0	surgery	0	Patterns	0	Population	0	Assessment	0	Irk	0	Triptan	0	Serotonin receptor	0	pray	0	carer	0	Shock	0
0	Grandfather	0	Adherence	0	husband	0	Anti-inflammatory	0	Future	0	Aggravate	0	CAT scan	0	Toxin	0	Depression	0	regime	0	Temporal	0	Agonist	0	Regimen	0	Botulinum Toxin	0	surgery	0	Patterns	0	Population	0	Assessment	0	Irk	0	Triptan	1	Serotonin receptor	1	pray	1	carer	0	Shock	0
0	Grandfather	0	Adherence	0	husband	0	Anti-inflammatory	0	Future	0	Aggravate	0	CAT scan	0	Toxin	0	Depression	0	regime	0	Temporal	0	Agonist	0	Regimen	0	Botulinum Toxin	0	surgery	0	Patterns	0	Population	0	Assessment	0	Irk	1	Triptan	0	Serotonin receptor	0	pray	0	carer	0	Shock	0
0	Grandfather	0	Adherence	0	husband	0	Anti-inflammatory	0	Future	0	Aggravate	0	CAT scan	0	Toxin	0	Depression	0	regime	0	Temporal	0	Agonist	0	Regimen	0	Botulinum Toxin	0	surgery	0	Patterns	1	Population	1	Assessment	1	Irk	0	Triptan	0	Serotonin receptor	0	pray	0	carer	0	Shock	0
0	Grandfather	0	Adherence	0	husband	0	Anti-inflammatory	0	Future	0	Aggravate	0	CAT scan	0	Toxin	1	Depression	1	regime	1	Temporal	1	Agonist	1	Regimen	1	Botulinum Toxin	1	surgery	1	Patterns	0	Population	0	Assessment	0	Irk	0	Triptan	0	Serotonin receptor	0	pray	0	carer	0	Shock	0
0	Grandfather	0	Adherence	0	husband	0	Anti-inflammatory	1	Future	1	Aggravate	1	CAT scan	1	Toxin	0	Depression	0	regime	0	Temporal	0	Agonist	0	Regimen	0	Botulinum Toxin	0	surgery	0	Patterns	0	Population	0	Assessment	0	Irk	0	Triptan	0	Serotonin receptor	0	pray	0	carer	0	Shock	0
1	Grandfather	1	Adherence	1	husband	1	Anti-inflammatory	0	Future	0	Aggravate	0	CAT scan	0	Toxin	0	Depression	0	regime	0	Temporal	0	Agonist	0	Regimen	0	Botulinum Toxin	0	surgery	0	Patterns	0	Population	0	Assessment	0	Irk	0	Triptan	0	Serotonin receptor	0	pray	0	carer	0	Shock	0
0	Grandfather	0	Adherence	0	husband	0	Anti-inflammatory	0	Future	0	Aggravate	0	CAT scan	0	Toxin	0	Depression	0	regime	0	Temporal	0	Agonist	0	Regimen	0	Botulinum Toxin	0	surgery	0	Patterns	0	Population	0	Assessment	0	Irk	0	Triptan	0	Serotonin receptor	0	pray	0	carer	0	Shock	0
0	Grandfather	0	Adherence	0	husband	0	Anti-inflammatory	0	Future	0	Aggravate	0	CAT scan	0	Toxin	0	Depression	0	regime	0	Temporal	0	Agonist	0	Regimen	0	Botulinum Toxin	0	surgery	0	Patterns	0	Population	0	Assessment	0	Irk	0	Triptan	0	Serotonin receptor	0	pray	0	carer	0	Shock	0

1 1 1 1 1 1 1 1 1 1 1 1 1 1 1 1 1 1 1 1 1 1 1 1 1

August 2019 Migraine Webcrawl Data

Condition	URL Id	URL	Total number of words	P0a	P0b	P1a	P1b	P2a	P2b	P3a	P3b	P4a	P4b	P5a	P5b
Migraine	1951845	http://curetogether.com/Migraine/discuss/	36387	Migraine	487	Ache	137	Ill	213	Pain	153	work	141	drug	45
Migraine	1765289	http://forums.phoenixrising.me/index.php?threads/questions-about-migraine-aura.34477/	4378	Migraine	64	Ache	11	Ill	18	Pain	9	work	9	drug	6
Migraine	2283240	http://forums.prohealth.com/forums/index.php?threads/migraine-from-hell-called-status-migrainosus.110648/	1390	Migraine	17	Ache	4	Ill	4	Pain	9	work	2	drug	0
Migraine	1951709	http://forums.whirlpool.net.au/archive/1924289	8822	Migraine	88	Ache	38	Ill	42	Pain	42	work	26	drug	5
Migraine	2174178	http://helpforheadaches.com/forum.htm	902	Migraine	15	Ache	12	Ill	3	Pain	0	work	3	drug	0
Migraine	2275843	http://migrainepage.forumotion.net/	189	Migraine	0	Ache	0	Ill	0	Pain	0	work	0	drug	0
Migraine	2275852	http://migrainepage.forumotion.net/f1-migraines	1230	Migraine	6	Ache	0	Ill	0	Pain	0	work	0	drug	0
Migraine	2293278	http://migrainetalk.forumotion.co.uk/	923	Migraine	15	Ache	0	Ill	0	Pain	0	work	0	drug	0
Migraine	2053758	http://vetsbenefits.net/topic/67841/Migraines?page=2	135	Migraine	0	Ache	0	Ill	0	Pain	0	work	0	drug	0
Migraine	2247837	http://www.experienceproject.com/groups/Get-Migraine-Headaches/102	43	Migraine	0	Ache	0	Ill	1	Pain	0	work	0	drug	0
Migraine	2242530	http://www.experienceproject.com/groups/Have-Vestibular-Migraine/117436	43	Migraine	0	Ache	0	Ill	1	Pain	0	work	0	drug	0
Migraine	2275938	http://www.fibromyalgiaforums.org/forum/general-discussions-about-fibromyalgia/26893-fibro-migraines.html	2540	Migraine	27	Ache	20	Ill	4	Pain	18	work	10	drug	0
Migraine	2277089	http://www.fightingfatigue.org/headache-migraine-websites-forums/	418	Migraine	5	Ache	4	Ill	0	Pain	2	work	0	drug	0
Migraine	1679505	http://www.healthcentral.com/migraine/	547	Migraine	19	Ache	5	Ill	1	Pain	1	work	1	drug	0
Migraine	1679511	http://www.healthcentral.com/migraine/c/123/177401/experience-spring-migraine	1259	Migraine	42	Ache	4	Ill	3	Pain	0	work	1	drug	0
Migraine	2250921	http://www.healthfulchat.org/migraine-chat-room.html	1063	Migraine	25	Ache	8	Ill	1	Pain	2	work	7	drug	0
Migraine	2029502	http://www.helpforheadaches.com/	389	Migraine	13	Ache	7	Ill	4	Pain	0	work	1	drug	0
Migraine	1679502	http://www.medhelp.org/forums/Migraines--Headaches/show/123	1337	Migraine	11	Ache	20	Ill	1	Pain	9	work	1	drug	0
Migraine	2277487	http://www.migraine.org.uk/	348	Migraine	9	Ache	1	Ill	0	Pain	0	work	0	drug	0
Migraine	2292659	http://www.migraine.org.uk/get-advice/	440	Migraine	9	Ache	0	Ill	0	Pain	0	work	0	drug	0
Migraine	2069158	http://www.migrainebuddy.com/migraine-stories/	526	Migraine	15	Ache	1	Ill	2	Pain	3	work	2	drug	0
Migraine	2063539	http://www.migrainebuddy.com/understanding-migraines-the-gender-divide/	2038	Migraine	117	Ache	3	Ill	5	Pain	14	work	7	drug	1
Migraine	2286545	http://www.migrainepage.com/	23	Migraine	0	Ache	0	Ill	0	Pain	0	work	0	drug	0
Migraine	2291250	http://www.migrainepage.com/help.html	287	Migraine	10	Ache	8	Ill	0	Pain	0	work	0	drug	0
Migraine	2291906	http://www.migraines.org/help/helpform.htm	340	Migraine	8	Ache	2	Ill	1	Pain	0	work	1	drug	0
Migraine	1679496	http://www.mvertigo.org/	363	Migraine	4	Ache	1	Ill	0	Pain	0	work	0	drug	0
Migraine	2041773	http://www.nomorepanic.co.uk/showthread.php?t=106093	1917	Migraine	16	Ache	0	Ill	5	Pain	5	work	3	drug	0
Migraine	2289385	http://www.relieve-migraine-headache.com/basilar-migraines.html	773	Migraine	20	Ache	5	Ill	2	Pain	0	work	0	drug	0
Migraine	2277529	http://www.thedailyheadache.com/	3540	Migraine	35	Ache	18	Ill	40	Pain	1	work	14	drug	5
Migraine	2277957	http://www.thedailymigraine.com/forum-moot/	121	Migraine	4	Ache	0	Ill	0	Pain	0	work	0	drug	0
Migraine	2290740	http://www.thedailymigraine.com/support-groups/	290	Migraine	11	Ache	0	Ill	2	Pain	6	work	0	drug	0
Migraine	2274213	http://www.themigrainerevolution.com/yahoo-group/	501	Migraine	16	Ache	0	Ill	2	Pain	0	work	0	drug	1
Migraine	2294811	http://www.tmwiki.org/forum/threads/my-migraines.2072/	2467	Migraine	30	Ache	9	Ill	8	Pain	6	work	7	drug	3
Migraine	2244789	http://www.topix.com/forum/health/migraine	1794	Migraine	24	Ache	5	Ill	4	Pain	3	work	0	drug	5
Migraine	1875338	http://www.usmle-forums.com/usmle-step-1-forum/217482-migraine.html	660	Migraine	4	Ache	1	Ill	0	Pain	0	work	0	drug	0
Migraine	2295373	https://cassiopaea.org/forum/?topic=17971.0	2196	Migraine	0	Ache	0	Ill	4	Pain	0	work	2	drug	0
Migraine	2289387	https://coloradopain.co/patient-forms/	383	Migraine	0	Ache	0	Ill	2	Pain	5	work	2	drug	0
Migraine	2276050	https://community.qvc.com/t5/Wellness/MIGRAINE-sufferers/td-p/1182015	3353	Migraine	34	Ache	3	Ill	6	Pain	6	work	8	drug	0
Migraine	1866146	https://curetogether.com/Migraine/discuss/	36747	Migraine	487	Ache	137	Ill	213	Pain	153	work	141	drug	45
Migraine	2268735	https://ehealthforum.com/health/alzheimers.html	715	Migraine	0	Ache	0	Ill	1	Pain	0	work	0	drug	1
Epilepsy	2291645	https://ehealthforum.com/health/epilepsy.html	652	Migraine	0	Ache	1	Ill	1	Pain	0	work	0	drug	1
Migraine	2278014	https://ehealthforum.com/health/headache-and-migraines-question-t504544.html	746	Migraine	1	Ache	8	Ill	1	Pain	0	work	1	drug	1
Migraine	2285137	https://ergoweb.com/forums/topic/treatment-of-light-induced-migraine-headaches-in-the-office/	1236	Migraine	11	Ache	6	Ill	4	Pain	0	work	6	drug	0
Migraine	2296496	https://exchanges.webmd.com/migraines-and-headaches-exchange	6384	Migraine	80	Ache	56	Ill	27	Pain	16	work	6	drug	7
Migraine	2286108	https://forum.bulletproof.com/discussion/1406/bulletproof-migraine-specific-diet	1772	Migraine	21	Ache	11	Ill	5	Pain	3	work	5	drug	0
Migraine	2290131	https://forum.dontpayfull.com/threads/migraine-medicine.25791/	3210	Migraine	36	Ache	29	Ill	17	Pain	16	work	13	drug	0
Migraine	2282154	https://forum.grasscity.com/threads/migraines.453628/	2514	Migraine	47	Ache	14	Ill	9	Pain	8	work	3	drug	4
Migraine	2273025	https://forums.phoenixrising.me/index.php?threads/questions-about-migraine-aura.34477/	4350	Migraine	64	Ache	11	Ill	18	Pain	9	work	9	drug	6
Migraine	2095418	https://forums.whirlpool.net.au/archive/1924289	8822	Migraine	88	Ache	38	Ill	42	Pain	42	work	26	drug	5
Migraine	2294008	https://hubpages.com/health/forum/23466/any-ocular-migraine-sufferers-here	3029	Migraine	28	Ache	5	Ill	6	Pain	9	work	7	drug	0
Migraine	2208143	https://livingwell.migrainedisease.com	350	Migraine	7	Ache	3	Ill	3	Pain	0	work	0	drug	0
Migraine	2274362	https://livingwell.migrainedisease.com/forum.htm	902	Migraine	15	Ache	12	Ill	3	Pain	0	work	3	drug	0
Migraine	2170918	https://livingwell.migrainedisease.com/www	389	Migraine	13	Ache	7	Ill	4	Pain	0	work	1	drug	0
Migraine	2287140	https://migraine.newlifeoutlook.com/community/	523	Migraine	9	Ache	1	Ill	0	Pain	0	work	0	drug	0
Migraine	2291171	https://migraine.newlifeoutlook.com/community/discussion/17/complex-migraine	681	Migraine	13	Ache	8	Ill	1	Pain	1	work	1	drug	0
Migraine	2269712	https://migrainebuddy.com/migraine-stories/	526	Migraine	15	Ache	1	Ill	2	Pain	3	work	2	drug	0
Migraine	2270662	https://migrainebuddy.com/migraine/2018/9/13/understanding-migraines-the-gender-divide	1826	Migraine	103	Ache	2	Ill	6	Pain	14	work	7	drug	1
Migraine	2100374	https://migrainebuddy.com/understanding-migraines-the-gender-divide/	2038	Migraine	117	Ache	3	Ill	5	Pain	14	work	7	drug	1
Migraine	2270732	https://migraineresearchfoundation.org/resources/resources-and-links/	1691	Migraine	37	Ache	34	Ill	11	Pain	4	work	2	drug	1
Migraine	2251382	https://migrainetalkblog.wordpress.com/	1208	Migraine	57	Ache	9	Ill	3	Pain	0	work	0	drug	0
Migraine	2283689	https://patient.info/forums/discuss/browse/migraine-1415	1131	Migraine	6	Ache	2	Ill	1	Pain	1	work	0	drug	0
Migraine	2268233	https://vetsbenefits.net/migraines-t67841.html	8897	Migraine	134	Ache	71	Ill	20	Pain	18	work	41	drug	1
Migraine	2283242	https://www.chronofhorse.com/forum/archive/index.php/t-318339.html	5948	Migraine	38	Ache	12	Ill	32	Pain	9	work	12	drug	1
Migraine	2296236	https://www.curezone.org/forums/am.asp?i=60104	1592	Migraine	23	Ache	6	Ill	6	Pain	2	work	10	drug	2
Migraine	2294557	https://www.curezone.org/forums/f3.asp?f=68	3380	Migraine	22	Ache	21	Ill	10	Pain	11	work	4	drug	2
Migraine	2294387	https://www.dailystrength.org/group/migraine-headaches	826	Migraine	14	Ache	3	Ill	0	Pain	1	work	0	drug	2
Migraine	2294417	https://www.dinet.org/forums/topic/27868-anybody-else-have-an-intractable-migraine/	2140	Migraine	21	Ache	11	Ill	6	Pain	16	work	8	drug	0
Migraine	2273389	https://www.drugabuse.gov/	560	Migraine	0	Ache	0	Ill	0	Pain	0	work	0	drug	6
Migraine	2273344	https://www.drugabuse.gov/related-topics/substance-use-suds-in-lgbt-populations	1468	Migraine	0	Ache	0	Ill	3	Pain	1	work	0	drug	11
Migraine	2247957	https://www.eumom.ie/forums/topic/bad-bad-migraine/	1330	Migraine	10	Ache	3	Ill	4	Pain	3	work	2	drug	1
Migraine	2272107	https://www.facebook.com/MigraineDotCom	375	Migraine	5	Ache	1	Ill	1	Pain	3	work	0	drug	0
Migraine	2284453	https://www.fodors.com/community/air-travel/long-flight-migraine.cfm	2591	Migraine	27	Ache	14	Ill	7	Pain	1	work	4	drug	0
Migraine	2276778	https://www.healingwell.com/community/?f=31	700	Migraine	3	Ache	0	Ill	0	Pain	0	work	0	drug	0
Migraine	2008810	https://www.healthcentral.com/migraine/	463	Migraine	15	Ache	0	Ill	5	Pain	0	work	2	drug	0
Migraine	2269583	https://www.healthcentral.com/migraine/c/123/177401/experience-spring-migraine	1089	Migraine	38	Ache	4	Ill	3	Pain	0	work	1	drug	0
Migraine	2282363	https://www.healthfulchat.org/migraine-chat-room.html	1063	Migraine	25	Ache	8	Ill	1	Pain	2	work	7	drug	0
Migraine	2275844	https://www.infantrisk.com/forum/forum/medications-and-breastfeeding-mothers/other-uncategorized-medications/152-migraine-medications	2082	Migraine	15	Ache	3	Ill	7	Pain	1	work	4	drug	18
Migraine	2290390	https://www.inspire.com/groups/headaches-and-migraines/discussion/migraines-each-day-unbearable/	5301	Migraine	92	Ache	44	Ill	17	Pain	9	work	14	drug	22
Migraine	2267892	https://www.medhelp.org/forums/Migraines--Headaches/show/123	1320	Migraine	9	Ache	17	Ill	0	Pain	6	work	2	drug	0
Migraine	2274356	https://www.medicinenet.com/drug_abuse/article.htm	7155	Migraine	0	Ache	10	Ill	19	Pain	2	work	4	drug	221
Migraine	2278343	https://www.medschat.com/Discuss/Migraine-Tension-Headache/	3875	Migraine	20	Ache	11	Ill	37	Pain	6	work	15	drug	13
Migraine	1945768	https://www.mvertigo.org/	391	Migraine	1	Ache	0	Ill	1	Pain	0	work	0	drug	0
Migraine	2267453	https://www.nomorepanic.co.uk/showthread.php?t=106093	1921	Migraine	16	Ache	0	Ill	5	Pain	5	work	3	drug	0
Migraine	2290389	https://www.patientslikeme.com/conditions/135-migraine	3640	Migraine	175	Ache	20	Ill	1	Pain	21	work	0	drug	1
Migraine	2282664	https://www.patientslikeme.com/patients	882	Migraine	0	Ache	0	Ill	0	Pain	0	work	0	drug	0
Migraine	2291744	https://www.physicsforums.com/threads/the-four-stages-of-migraine.305792/	1946	Migraine	63	Ache	24	Ill	3	Pain	2	work	4	drug	0
Migraine	2293898	https://www.tapatalk.com/groups/headachepainfreeforums/topic/766/Silent-Migraine	1566	Migraine	17	Ache	4	Ill	7	Pain	9	work	9	drug	2
Migraine	1679473	https://www.tapatalk.com/groups/vets/topic/67841/Migraines?page=2	13151	Migraine	134	Ache	71	Ill	30	Pain	18	work	41	drug	1
Migraine	2277773	https://www.topix.com/forum/health/migraine	1794	Migraine	24	Ache	5	Ill	4	Pain	3	work	0	drug	5
Migraine	2264114	https://www.usmle-forums.com/usmle-step-1-forum/217482-migraine.html	660	Migraine	4	Ache	1	Ill	0	Pain	0	work	0	drug	0
Migraine	2273781	https://www.webmd.com/anxiety-panic/default.htm	1074	Migraine	0	Ache	1	Ill	1	Pain	0	work	0	drug	2
			244977		3400		1090		992		736		675		[illegible]

P69a	P69b	P70a	P70b	P71a	P71b	P72a	P72b	P73a	P73b	P74a	P74b	P75a	P75b	P76a	P76b	P77a	P77b	P78a	P78b	P79a	P79b	P80a	P80b	P81a	P81b	P82a	P82b	P83a	P83b	P84a	P84b	P85a	P85b	P86a	P86b	P87a	P87b	P88a	P88b
Mother	6	Serotonin	0	Disability	0	nasal	10	Cluster headache	0	pray	11	husband	12	travel	2	employ	3	Management	2	Walk	6	Neck pain	14	Pregnancy	10	Support group	0	Chronic pain	0	Blocker	3	Menopause	6	Parent	2	Acetaminophen	4	Population	0

P89a	P89b	P90a	P90b	P91a	P91b	P92a	P92b	P93a	P93b	P94a	P94b	P95a	P95b	P96a	P96b	P97a	P97b	P98a	P98b	P99a	P99b	P100a	P100b	P101a	P101b	P102a	P102b	P103a	P103b	P104a	P104b	P105a	P105b	P106a	P106b	P107a	P107b	P108a	P108b
Abortive	6	Ergot	9	Daughter	2	ARB	8	Present	3	Depressed	8	Prophylactic	4	Sensitivity to light	7	Wife	6	Brother	4	Lifestyle	4	Inhibitor	0	Education	0	Visual disturbance	2	Photophobia	8	Surgery	2	Acute	1	Emergency	3	Allodynia	10	Genetic	6

P109a	P109b	P110a	P110b	P111a	P111b	P112a	P112b	P113a	P113b	P114a	P114b	P115a	P115b	P116a	P116b	P117a	P117b	P118a	P118b	P119a	P119b	P120a	P120b	P121a	P121b	P122a	P122b	P123a	P123b
Anger	0	Occipital	9	Sister	4	Procedure	6	Sore	6	procedure	6	Biofeedback	8	Opioid	0	Central	0	History	1	Butalbital	5	Peer	0	Drive	2	Antidepressants	2	nose	3

P124a	P124b	P125a	P125b	P126a	P126b	P127a	P127b	P128a	P128b	P129a	P129b	P130a	P130b	P131a	P131b	P132a	P132b	P133a	P133b	P134a	P134b	P135a	P135b	P136a	P136b	P137a	P137b	P138a	P138b
Vascular	2	stigma	4	Impact	1	Coping	1	Epilepsy	4	Sad	4	Joy	5	Duration	3	minister	3	Nurse	1	Steroid	4	Preventive	2	Riboflavin	2	Stages	0	Future	0

P139a	P139b	P140a	P140b	P141a	P141b	P142a	P142b	P143a	P143b	P144a	P144b	P145a	P145b	P146a	P146b	P147a	P147b	P148a	P148b	P149a	P149b	P150a	P150b	P151a	P151b	P152a	P152b	P153a	P153b	P154a	P154b	P155a	P155b	P156a	P156b	P157a	P157b	P158a	P158b	P159a	P159b	P160a	P160b	P161a	P161b	P162a	P162b	P163a	P163b	P164a	P164b	P165a	P165b	P166a	P166b	P167a	P167b	P168a	P168b	P169a	P169b	P170a	P170b	P171a	P171b	P172a	P172b	P173a	P173b	P174a	P174b	P175a	P175b	P176a	P176b	P177a	P177b	P178a	P178b	P179a	P179b	P180a	P180b	P181a	P181b	P182a	P182b	P183a	P183b	P184a	P184b	P185a	P185b	P186a	P186b	P187a	P187b	P188a	P188b	P189a	P189b	P190a	P190b	P191a	P191b	P192a	P192b	P193a	P193b	P194a	P194b	P195a	P195b	P196a	P196b	P197a	P197b	P198a	P198b	P199a
Trauma	0	Adverse effect	0	faith	5	Intractable	2	Sensitivity to sound	3	Aggravate	1	Hemiplegic migraine	1	Calcium channel	1	Risk factor	0	Toxin	2	Incidence	4	Trigger point	5	Shock	4	Hurts	3	Characteristic	0	Prophylaxis	0	Acute migraine	0	Quality of life	1	Testing	1	Weight loss	2	Agonist	1	Confuse	4	Patterns	2	Cervical spine	0	Suicide	0	Peer support	0	boss	0	regime	1	Grandmother	1	Headache diary	1	Complications	0	Intervention	0	Angiotensin	0	Episodic	0	impairment	0	Anti-inflammatory	2	Regimen	1	Glutamate	2	Neck muscle	3	Calcium channel blocker	1	CAT scan	0	Sinus headache	1	vacation	2	NSAID	1	Accident	1	Occipital nerve	3	Motion sickness	0	Concussion	0	Remission	0	Blood flow	0	Type A	0	Facial pain	0	Lifestyle change	2	Anti-seizure	2	Upset	1	surgery	1	Prognosis	0	Rehabilitation	0	Detoxification	0	Screening	0	Inpatient
Trauma	0	Adverse effect	4	faith	0	Intractable	0	Sensitivity to sound	0	Aggravate	0	Hemiplegic migraine	0	Calcium channel	0	Risk factor	0	Toxin	0	Incidence	0	Trigger point	0	Shock	0	Hurts	0	Characteristic	0	Prophylaxis	0	Acute migraine	0	Quality of life	0	Testing	0	Weight loss	0	Agonist	0	Confuse	0	Patterns	0	Cervical spine	0	Suicide	0	Peer support	0	boss	0	regime	0	Grandmother	3	Headache diary	0	Complications	0	Intervention	0	Angiotensin	0	Episodic	0	impairment	3	Anti-inflammatory	0	Regimen	0	Glutamate	1	Neck muscle	0	Calcium channel blocker	0	CAT scan	0	Sinus headache	0	vacation	0	NSAID	0	Accident	0	Occipital nerve	0	Motion sickness	0	Concussion	0	Remission	0	Blood flow	0	Type A	0	Facial pain	0	Lifestyle change	0	Anti-seizure	0	Upset	0	surgery	0	Prognosis	0	Rehabilitation	0	Detoxification	0	Screening	0	Inpatient
Trauma	1	Adverse effect	0	faith	0	Intractable	0	Sensitivity to sound	0	Aggravate	0	Hemiplegic migraine	0	Calcium channel	0	Risk factor	0	Toxin	0	Incidence	0	Trigger point	0	Shock	0	Hurts	0	Characteristic	0	Prophylaxis	0	Acute migraine	0	Quality of life	0	Testing	0	Weight loss	0	Agonist	0	Confuse	0	Patterns	0	Cervical spine	0	Suicide	0	Peer support	0	boss	0	regime	0	Grandmother	0	Headache diary	0	Complications	0	Intervention	0	Angiotensin	0	Episodic	0	impairment	0	Anti-inflammatory	0	Regimen	0	Glutamate	0	Neck muscle	1	Calcium channel blocker	0	CAT scan	0	Sinus headache	0	vacation	0	NSAID	1	Accident	0	Occipital nerve	0	Motion sickness	0	Concussion	0	Remission	0	Blood flow	0	Type A	0	Facial pain	0	Lifestyle change	0	Anti-seizure	0	Upset	0	surgery	0	Prognosis	0	Rehabilitation	0	Detoxification	0	Screening	0	Inpatient
Trauma	0	Adverse effect	0	faith	0	Intractable	0	Sensitivity to sound	0	Aggravate	1	Hemiplegic migraine	0	Calcium channel	0	Risk factor	0	Toxin	0	Incidence	0	Trigger point	0	Shock	0	Hurts	0	Characteristic	0	Prophylaxis	0	Acute migraine	0	Quality of life	0	Testing	2	Weight loss	0	Agonist	0	Confuse	0	Patterns	1	Cervical spine	0	Suicide	0	Peer support	0	boss	0	regime	0	Grandmother	0	Headache diary	2	Complications	0	Intervention	0	Angiotensin	0	Episodic	0	impairment	0	Anti-inflammatory	0	Regimen	0	Glutamate	0	Neck muscle	0	Calcium channel blocker	0	CAT scan	1	Sinus headache	1	vacation	0	NSAID	0	Accident	0	Occipital nerve	0	Motion sickness	0	Concussion	0	Remission	0	Blood flow	0	Type A	1	Facial pain	1	Lifestyle change	0	Anti-seizure	0	Upset	0	surgery	1	Prognosis	0	Rehabilitation	0	Detoxification	0	Screening	0	Inpatient
Trauma	0	Adverse effect	0	faith	0	Intractable	0	Sensitivity to sound	0	Aggravate	0	Hemiplegic migraine	0	Calcium channel	0	Risk factor	0	Toxin	0	Incidence	0	Trigger point	0	Shock	0	Hurts	0	Characteristic	0	Prophylaxis	0	Acute migraine	0	Quality of life	0	Testing	0	Weight loss	0	Agonist	0	Confuse	0	Patterns	0	Cervical spine	0	Suicide	0	Peer support	0	boss	2	regime	1	Grandmother	0	Headache diary	0	Complications	0	Intervention	0	Angiotensin	0	Episodic	0	impairment	0	Anti-inflammatory	0	Regimen	1	Glutamate	0	Neck muscle	0	Calcium channel blocker	0	CAT scan	0	Sinus headache	0	vacation	0	NSAID	0	Accident	0	Occipital nerve	0	Motion sickness	0	Concussion	0	Remission	0	Blood flow	0	Type A	0	Facial pain	0	Lifestyle change	0	Anti-seizure	0	Upset	0	surgery	0	Prognosis	0	Rehabilitation	0	Detoxification	0	Screening	0	Inpatient
Trauma	0	Adverse effect	0	faith	0	Intractable	0	Sensitivity to sound	0	Aggravate	0	Hemiplegic migraine	0	Calcium channel	0	Risk factor	0	Toxin	0	Incidence	0	Trigger point	0	Shock	0	Hurts	0	Characteristic	0	Prophylaxis	0	Acute migraine	0	Quality of life	0	Testing	0	Weight loss	0	Agonist	0	Confuse	0	Patterns	0	Cervical spine	0	Suicide	0	Peer support	0	boss	0	regime	0	Grandmother	0	Headache diary	0	Complications	0	Intervention	0	Angiotensin	0	Episodic	0	impairment	0	Anti-inflammatory	0	Regimen	0	Glutamate	0	Neck muscle	0	Calcium channel blocker	0	CAT scan	0	Sinus headache	0	vacation	0	NSAID	0	Accident	0	Occipital nerve	0	Motion sickness	0	Concussion	0	Remission	0	Blood flow	0	Type A	0	Facial pain	0	Lifestyle change	0	Anti-seizure	0	Upset	0	surgery	0	Prognosis	0	Rehabilitation	0	Detoxification	0	Screening	0	Inpatient
Trauma	0	Adverse effect	0	faith	0	Intractable	0	Sensitivity to sound	0	Aggravate	0	Hemiplegic migraine	0	Calcium channel	0	Risk factor	0	Toxin	0	Incidence	0	Trigger point	0	Shock	0	Hurts	0	Characteristic	0	Prophylaxis	0	Acute migraine	0	Quality of life	0	Testing	0	Weight loss	0	Agonist	0	Confuse	0	Patterns	0	Cervical spine	0	Suicide	0	Peer support	0	boss	0	regime	0	Grandmother	0	Headache diary	0	Complications	0	Intervention	0	Angiotensin	0	Episodic	0	impairment	0	Anti-inflammatory	0	Regimen	0	Glutamate	0	Neck muscle	0	Calcium channel blocker	0	CAT scan	0	Sinus headache	0	vacation	0	NSAID	0	Accident	0	Occipital nerve	0	Motion sickness	0	Concussion	0	Remission	0	Blood flow	0	Type A	0	Facial pain	0	Lifestyle change	0	Anti-seizure	0	Upset	0	surgery	0	Prognosis	0	Rehabilitation	0	Detoxification	0	Screening	0	Inpatient
Trauma	0	Adverse effect	0	faith	0	Intractable	0	Sensitivity to sound	0	Aggravate	0	Hemiplegic migraine	0	Calcium channel	0	Risk factor	0	Toxin	0	Incidence	0	Trigger point	0	Shock	1	Hurts	0	Characteristic	0	Prophylaxis	0	Acute migraine	0	Quality of life	0	Testing	0	Weight loss	0	Agonist	0	Confuse	0	Patterns	0	Cervical spine	0	Suicide	0	Peer support	0	boss	0	regime	0	Grandmother	0	Headache diary	0	Complications	0	Intervention	0	Angiotensin	0	Episodic	0	impairment	0	Anti-inflammatory	0	Regimen	0	Glutamate	0	Neck muscle	0	Calcium channel blocker	0	CAT scan	0	Sinus headache	0	vacation	0	NSAID	0	Accident	0	Occipital nerve	0	Motion sickness	0	Concussion	0	Remission	0	Blood flow	0	Type A	0	Facial pain	0	Lifestyle change	0	Anti-seizure	0	Upset	0	surgery	0	Prognosis	0	Rehabilitation	0	Detoxification	0	Screening	0	Inpatient
Trauma	0	Adverse effect	0	faith	0	Intractable	0	Sensitivity to sound	0	Aggravate	0	Hemiplegic migraine	0	Calcium channel	0	Risk factor	0	Toxin	0	Incidence	0	Trigger point	0	Shock	0	Hurts	0	Characteristic	0	Prophylaxis	0	Acute migraine	0	Quality of life	0	Testing	0	Weight loss	0	Agonist	0	Confuse	0	Patterns	0	Cervical spine	0	Suicide	0	Peer support	0	boss	0	regime	0	Grandmother	0	Headache diary	0	Complications	0	Intervention	0	Angiotensin	0	Episodic	0	impairment	0	Anti-inflammatory	0	Regimen	0	Glutamate	0	Neck muscle	0	Calcium channel blocker	0	CAT scan	0	Sinus headache	0	vacation	0	NSAID	0	Accident	0	Occipital nerve	0	Motion sickness	0	Concussion	0	Remission	0	Blood flow	0	Type A	0	Facial pain	0	Lifestyle change	0	Anti-seizure	0	Upset	0	surgery	0	Prognosis	0	Rehabilitation	0	Detoxification	0	Screening	0	Inpatient
Trauma	0	Adverse effect	0	faith	0	Intractable	0	Sensitivity to sound	0	Aggravate	0	Hemiplegic migraine	0	Calcium channel	0	Risk factor	0	Toxin	0	Incidence	0	Trigger point	0	Shock	0	Hurts	0	Characteristic	0	Prophylaxis	0	Acute migraine	0	Quality of life	0	Testing	0	Weight loss	0	Agonist	0	Confuse	0	Patterns	0	Cervical spine	0	Suicide	0	Peer support	0	boss	0	regime	0	Grandmother	0	Headache diary	0	Complications	0	Intervention	0	Angiotensin	0	Episodic	0	impairment	0	Anti-inflammatory	0	Regimen	0	Glutamate	0	Neck muscle	0	Calcium channel blocker	0	CAT scan	0	Sinus headache	0	vacation	0	NSAID	0	Accident	0	Occipital nerve	0	Motion sickness	0	Concussion	0	Remission	0	Blood flow	0	Type A	0	Facial pain	0	Lifestyle change	0	Anti-seizure	0	Upset	0	surgery	0	Prognosis	0	Rehabilitation	0	Detoxification	0	Screening	0	Inpatient
Trauma	0	Adverse effect	0	faith	0	Intractable	0	Sensitivity to sound	0	Aggravate	0	Hemiplegic migraine	0	Calcium channel	0	Risk factor	0	Toxin	0	Incidence	0	Trigger point	0	Shock	0	Hurts	0	Characteristic	0	Prophylaxis	0	Acute migraine	0	Quality of life	0	Testing	0	Weight loss	0	Agonist	0	Confuse	0	Patterns	0	Cervical spine	0	Suicide	0	Peer support	0	boss	0	regime	0	Grandmother	0	Headache diary	0	Complications	0	Intervention	0	Angiotensin	0	Episodic	0	impairment	0	Anti-inflammatory	0	Regimen	0	Glutamate	0	Neck muscle	0	Calcium channel blocker	0	CAT scan	0	Sinus headache	0	vacation	0	NSAID	0	Accident	0	Occipital nerve	0	Motion sickness	0	Concussion	0	Remission	0	Blood flow	0	Type A	0	Facial pain	0	Lifestyle change	0	Anti-seizure	0	Upset	0	surgery	0	Prognosis	0	Rehabilitation	0	Detoxification	0	Screening	0	Inpatient
Trauma	0	Adverse effect	0	faith	0	Intractable	0	Sensitivity to sound	0	Aggravate	0	Hemiplegic migraine	0	Calcium channel	0	Risk factor	0	Toxin	0	Incidence	0	Trigger point	0	Shock	0	Hurts	0	Characteristic	0	Prophylaxis	0	Acute migraine	0	Quality of life	0	Testing	0	Weight loss	0	Agonist	0	Confuse	0	Patterns	0	Cervical spine	0	Suicide	0	Peer support	0	boss	0	regime	0	Grandmother	0	Headache diary	0	Complications	0	Intervention	0	Angiotensin	0	Episodic	0	impairment	0	Anti-inflammatory	0	Regimen	0	Glutamate	0	Neck muscle	0	Calcium channel blocker	0	CAT scan	0	Sinus headache	0	vacation	0	NSAID	0	Accident	0	Occipital nerve	0	Motion sickness	0	Concussion	0	Remission	0	Blood flow	0	Type A	1	Facial pain	0	Lifestyle change	0	Anti-seizure	0	Upset	0	surgery	0	Prognosis	0	Rehabilitation	0	Detoxification	0	Screening	0	Inpatient
Trauma	0	Adverse effect	0	faith	0	Intractable	0	Sensitivity to sound	0	Aggravate	0	Hemiplegic migraine	0	Calcium channel	0	Risk factor	0	Toxin	0	Incidence	0	Trigger point	0	Shock	0	Hurts	0	Characteristic	0	Prophylaxis	0	Acute migraine	0	Quality of life	0	Testing	0	Weight loss	2	Agonist	0	Confuse	0	Patterns	0	Cervical spine	0	Suicide	0	Peer support	0	boss	0	regime	0	Grandmother	0	Headache diary	0	Complications	0	Intervention	0	Angiotensin	0	Episodic	0	impairment	0	Anti-inflammatory	0	Regimen	0	Glutamate	0	Neck muscle	0	Calcium channel blocker	0	CAT scan	0	Sinus headache	0	vacation	0	NSAID	0	Accident	0	Occipital nerve	0	Motion sickness	0	Concussion	0	Remission	0	Blood flow	0	Type A	0	Facial pain	0	Lifestyle change	0	Anti-seizure	0	Upset	0	surgery	0	Prognosis	0	Rehabilitation	0	Detoxification	0	Screening	0	Inpatient
Trauma	0	Adverse effect	0	faith	0	Intractable	0	Sensitivity to sound	0	Aggravate	0	Hemiplegic migraine	1	Calcium channel	0	Risk factor	0	Toxin	0	Incidence	0	Trigger point	0	Shock	0	Hurts	0	Characteristic	0	Prophylaxis	0	Acute migraine	1	Quality of life	0	Testing	0	Weight loss	0	Agonist	0	Confuse	0	Patterns	0	Cervical spine	0	Suicide	0	Peer support	0	boss	0	regime	0	Grandmother	0	Headache diary	0	Complications	0	Intervention	0	Angiotensin	0	Episodic	0	impairment	0	Anti-inflammatory	0	Regimen	0	Glutamate	0	Neck muscle	0	Calcium channel blocker	0	CAT scan	0	Sinus headache	0	vacation	0	NSAID	0	Accident	0	Occipital nerve	0	Motion sickness	0	Concussion	0	Remission	0	Blood flow	0	Type A	0	Facial pain	0	Lifestyle change	0	Anti-seizure	0	Upset	0	surgery	0	Prognosis	0	Rehabilitation	0	Detoxification	0	Screening	0	Inpatient
Trauma	0	Adverse effect	0	faith	0	Intractable	0	Sensitivity to sound	0	Aggravate	0	Hemiplegic migraine	0	Calcium channel	0	Risk factor	1	Toxin	0	Incidence	0	Trigger point	0	Shock	0	Hurts	0	Characteristic	0	Prophylaxis	0	Acute migraine	2	Quality of life	0	Testing	0	Weight loss	0	Agonist	0	Confuse	0	Patterns	0	Cervical spine	0	Suicide	0	Peer support	0	boss	0	regime	0	Grandmother	0	Headache diary	0	Complications	0	Intervention	0	Angiotensin	0	Episodic	0	impairment	0	Anti-inflammatory	0	Regimen	0	Glutamate	0	Neck muscle	0	Calcium channel blocker	0	CAT scan	0	Sinus headache	0	vacation	0	NSAID	0	Accident	0	Occipital nerve	0	Motion sickness	0	Concussion	0	Remission	0	Blood flow	0	Type A	0	Facial pain	0	Lifestyle change	0	Anti-seizure	0	Upset	0	surgery	0	Prognosis	0	Rehabilitation	0	Detoxification	0	Screening	0	Inpatient
Trauma	2	Adverse effect	0	faith	0	Intractable	0	Sensitivity to sound	0	Aggravate	0	Hemiplegic migraine	0	Calcium channel	0	Risk factor	0	Toxin	0	Incidence	0	Trigger point	0	Shock	0	Hurts	0	Characteristic	0	Prophylaxis	0	Acute migraine	0	Quality of life	0	Testing	0	Weight loss	0	Agonist	0	Confuse	0	Patterns	0	Cervical spine	0	Suicide	0	Peer support	4	boss	0	regime	0	Grandmother	0	Headache diary	0	Complications	0	Intervention	0	Angiotensin	0	Episodic	0	impairment	0	Anti-inflammatory	0	Regimen	0	Glutamate	0	Neck muscle	0	Calcium channel blocker	0	CAT scan	0	Sinus headache	0	vacation	0	NSAID	0	Accident	0	Occipital nerve	0	Motion sickness	0	Concussion	0	Remission	0	Blood flow	0	Type A	0	Facial pain	0	Lifestyle change	0	Anti-seizure	0	Upset	0	surgery	0	Prognosis	0	Rehabilitation	0	Detoxification	0	Screening	0	Inpatient
Trauma	0	Adverse effect	0	faith	0	Intractable	0	Sensitivity to sound	0	Aggravate	0	Hemiplegic migraine	0	Calcium channel	0	Risk factor	0	Toxin	0	Incidence	0	Trigger point	0	Shock	0	Hurts	0	Characteristic	0	Prophylaxis	0	Acute migraine	0	Quality of life	0	Testing	0	Weight loss	0	Agonist	0	Confuse	0	Patterns	0	Cervical spine	0	Suicide	0	Peer support	0	boss	0	regime	1	Grandmother	0	Headache diary	0	Complications	0	Intervention	0	Angiotensin	0	Episodic	0	impairment	0	Anti-inflammatory	0	Regimen	1	Glutamate	0	Neck muscle	0	Calcium channel blocker	0	CAT scan	0	Sinus headache	0	vacation	0	NSAID	0	Accident	0	Occipital nerve	0	Motion sickness	0	Concussion	0	Remission	0	Blood flow	0	Type A	0	Facial pain	0	Lifestyle change	0	Anti-seizure	0	Upset	0	surgery	0	Prognosis	0	Rehabilitation	0	Detoxification	0	Screening	0	Inpatient
Trauma	0	Adverse effect	0	faith	0	Intractable	0	Sensitivity to sound	0	Aggravate	0	Hemiplegic migraine	0	Calcium channel	0	Risk factor	0	Toxin	0	Incidence	0	Trigger point	0	Shock	0	Hurts	2	Characteristic	0	Prophylaxis	0	Acute migraine	2	Quality of life	0	Testing	0	Weight loss	1	Agonist	0	Confuse	0	Patterns	0	Cervical spine	0	Suicide	0	Peer support	0	boss	0	regime	0	Grandmother	0	Headache diary	0	Complications	0	Intervention	0	Angiotensin	0	Episodic	0	impairment	0	Anti-inflammatory	0	Regimen	0	Glutamate	0	Neck muscle	0	Calcium channel blocker	0	CAT scan	0	Sinus headache	2	vacation	0	NSAID	0	Accident	0	Occipital nerve	0	Motion sickness	0	Concussion	0	Remission	0	Blood flow	1	Type A	0	Facial pain	0	Lifestyle change	0	Anti-seizure	0	Upset	0	surgery	0	Prognosis	0	Rehabilitation	0	Detoxification	0	Screening	0	Inpatient
Trauma	0	Adverse effect	0	faith	0	Intractable	0	Sensitivity to sound	0	Aggravate	0	Hemiplegic migraine	0	Calcium channel	0	Risk factor	0	Toxin	0	Incidence	0	Trigger point	0	Shock	0	Hurts	0	Characteristic	0	Prophylaxis	0	Acute migraine	0	Quality of life	0	Testing	0	Weight loss	0	Agonist	0	Confuse	0	Patterns	0	Cervical spine	0	Suicide	0	Peer support	0	boss	0	regime	0	Grandmother	0	Headache diary	0	Complications	0	Intervention	0	Angiotensin	0	Episodic	0	impairment	0	Anti-inflammatory	0	Regimen	0	Glutamate	0	Neck muscle	0	Calcium channel blocker	0	CAT scan	0	Sinus headache	0	vacation	0	NSAID	0	Accident	0	Occipital nerve	0	Motion sickness	0	Concussion	0	Remission	0	Blood flow	0	Type A	0	Facial pain	0	Lifestyle change	0	Anti-seizure	0	Upset	0	surgery	0	Prognosis	0	Rehabilitation	0	Detoxification	0	Screening	0	Inpatient
Trauma	0	Adverse effect	0	faith	0	Intractable	0	Sensitivity to sound	0	Aggravate	0	Hemiplegic migraine	0	Calcium channel	0	Risk factor	0	Toxin	0	Incidence	0	Trigger point	0	Shock	0	Hurts	0	Characteristic	0	Prophylaxis	0	Acute migraine	0	Quality of life	0	Testing	0	Weight loss	0	Agonist	0	Confuse	0	Patterns	0	Cervical spine	0	Suicide	0	Peer support	0	boss	0	regime	0	Grandmother	0	Headache diary	0	Complications	0	Intervention	0	Angiotensin	0	Episodic	0	impairment	0	Anti-inflammatory	0	Regimen	0	Glutamate	0	Neck muscle	0	Calcium channel blocker	0	CAT scan	0	Sinus headache	0	vacation	0	NSAID	0	Accident	0	Occipital nerve	0	Motion sickness	0	Concussion	0	Remission	0	Blood flow	0	Type A	0	Facial pain	0	Lifestyle change	0	Anti-seizure	0	Upset	0	surgery	0	Prognosis	0	Rehabilitation	0	Detoxification	0	Screening	0	Inpatient
Trauma	0	Adverse effect	0	faith	0	Intractable	0	Sensitivity to sound	0	Aggravate	0	Hemiplegic migraine	0	Calcium channel	0	Risk factor	0	Toxin	0	Incidence	0	Trigger point	0	Shock	0	Hurts	0	Characteristic	0	Prophylaxis	0	Acute migraine	0	Quality of life	0	Testing	0	Weight loss	0	Agonist	0	Confuse	0	Patterns	0	Cervical spine	0	Suicide	0	Peer support	0	boss	0	regime	0	Grandmother	0	Headache diary	0	Complications	0	Intervention	0	Angiotensin	0	Episodic	1	impairment	0	Anti-inflammatory	0	Regimen	0	Glutamate	0	Neck muscle	0	Calcium channel blocker	0	CAT scan	0	Sinus headache	0	vacation	0	NSAID	0	Accident	0	Occipital nerve	0	Motion sickness	0	Concussion	0	Remission	0	Blood flow	0	Type A	0	Facial pain	0	Lifestyle change	0	Anti-seizure	0	Upset	0	surgery	0	Prognosis	0	Rehabilitation	0	Detoxification	0	Screening	0	Inpatient
Trauma	1	Adverse effect	0	faith	0	Intractable	0	Sensitivity to sound	0	Aggravate	0	Hemiplegic migraine	0	Calcium channel	0	Risk factor	0	Toxin	0	Incidence	0	Trigger point	0	Shock	0	Hurts	0	Characteristic	0	Prophylaxis	0	Acute migraine	0	Quality of life	1	Testing	0	Weight loss	0	Agonist	0	Confuse	0	Patterns	0	Cervical spine	0	Suicide	0	Peer support	0	boss	0	regime	0	Grandmother	0	Headache diary	1	Complications	0	Intervention	0	Angiotensin	0	Episodic	0	impairment	0	Anti-inflammatory	0	Regimen	0	Glutamate	0	Neck muscle	0	Calcium channel blocker	0	CAT scan	0	Sinus headache	0	vacation	0	NSAID	0	Accident	0	Occipital nerve	0	Motion sickness	0	Concussion	0	Remission	0	Blood flow	1	Type A	0	Facial pain	0	Lifestyle change	0	Anti-seizure	0	Upset	0	surgery	0	Prognosis	0	Rehabilitation	0	Detoxification	0	Screening	0	Inpatient
Trauma	0	Adverse effect	0	faith	0	Intractable	0	Sensitivity to sound	0	Aggravate	0	Hemiplegic migraine	0	Calcium channel	0	Risk factor	0	Toxin	0	Incidence	0	Trigger point	0	Shock	0	Hurts	0	Characteristic	0	Prophylaxis	0	Acute migraine	0	Quality of life	0	Testing	0	Weight loss	0	Agonist	0	Confuse	0	Patterns	0	Cervical spine	0	Suicide	0	Peer support	0	boss	0	regime	0	Grandmother	0	Headache diary	0	Complications	0	Intervention	0	Angiotensin	0	Episodic	0	impairment	0	Anti-inflammatory	0	Regimen	0	Glutamate	0	Neck muscle	0	Calcium channel blocker	0	CAT scan	0	Sinus headache	0	vacation	0	NSAID	0	Accident	0	Occipital nerve	0	Motion sickness	0	Concussion	0	Remission	0	Blood flow	0	Type A	0	Facial pain	0	Lifestyle change	0	Anti-seizure	0	Upset	0	surgery	0	Prognosis	0	Rehabilitation	0	Detoxification	0	Screening	0	Inpatient
Trauma	0	Adverse effect	0	faith	0	Intractable	0	Sensitivity to sound	0	Aggravate	0	Hemiplegic migraine	0	Calcium channel	0	Risk factor	0	Toxin	0	Incidence	0	Trigger point	0	Shock	0	Hurts	0	Characteristic	0	Prophylaxis	0	Acute migraine	0	Quality of life	0	Testing	0	Weight loss	0	Agonist	0	Confuse	0	Patterns	0	Cervical spine	0	Suicide	0	Peer support	0	boss	0	regime	0	Grandmother	0	Headache diary	0	Complications	0	Intervention	0	Angiotensin	0	Episodic	0	impairment	0	Anti-inflammatory	0	Regimen	0	Glutamate	0	Neck muscle	0	Calcium channel blocker	0	CAT scan	0	Sinus headache	0	vacation	0	NSAID	0	Accident	0	Occipital nerve	0	Motion sickness	0	Concussion	0	Remission	0	Blood flow	0	Type A	0	Facial pain	0	Lifestyle change	0	Anti-seizure	0	Upset	0	surgery	0	Prognosis	0	Rehabilitation	0	Detoxification	0	Screening	0	Inpatient
Trauma	0	Adverse effect	0	faith	0	Intractable	0	Sensitivity to sound	0	Aggravate	0	Hemiplegic migraine	0	Calcium channel	0	Risk factor	0	Toxin	0	Incidence	0	Trigger point	0	Shock	0	Hurts	0	Characteristic	0	Prophylaxis	0	Acute migraine	0	Quality of life	0	Testing	0	Weight loss	0	Agonist	0	Confuse	0	Patterns	0	Cervical spine	0	Suicide	0	Peer support	0	boss	0	regime	0	Grandmother	0	Headache diary	0	Complications	0	Intervention	0	Angiotensin	0	Episodic	0	impairment	0	Anti-inflammatory	0	Regimen	0	Glutamate	0	Neck muscle	0	Calcium channel blocker	0	CAT scan	0	Sinus headache	0	vacation	0	NSAID	0	Accident	0	Occipital nerve	0	Motion sickness	0	Concussion	0	Remission	0	Blood flow	0	Type A	0	Facial pain	0	Lifestyle change	0	Anti-seizure	0	Upset	0	surgery	0	Prognosis	0	Rehabilitation	0	Detoxification	0	Screening	0	Inpatient
Trauma	0	Adverse effect	0	faith	0	Intractable	0	Sensitivity to sound	0	Aggravate	0	Hemiplegic migraine	0	Calcium channel	0	Risk factor	0	Toxin	0	Incidence	0	Trigger point	0	Shock	0	Hurts	0	Characteristic	0	Prophylaxis	0	Acute migraine	0	Quality of life	0	Testing	0	Weight loss	0	Agonist	0	Confuse	0	Patterns	0	Cervical spine	0	Suicide	0	Peer support	0	boss	0	regime	0	Grandmother	0	Headache diary	0	Complications	0	Intervention	0	Angiotensin	0	Episodic	0	impairment	0	Anti-inflammatory	0	Regimen	0	Glutamate	0	Neck muscle	0	Calcium channel blocker	0	CAT scan	0	Sinus headache	0	vacation	0	NSAID	0	Accident	0	Occipital nerve	0	Motion sickness	0	Concussion	0	Remission	0	Blood flow	0	Type A	0	Facial pain	0	Lifestyle change	0	Anti-seizure	0	Upset	0	surgery	0	Prognosis	0	Rehabilitation	0	Detoxification	0	Screening	0	Inpatient
Trauma	0	Adverse effect	0	faith	0	Intractable	0	Sensitivity to sound	0	Aggravate	0	Hemiplegic migraine	0	Calcium channel	0	Risk factor	0	Toxin	0	Incidence	0	Trigger point	0	Shock	0	Hurts	0	Characteristic	0	Prophylaxis	0	Acute migraine	0	Quality of life	0	Testing	0	Weight loss	0	Agonist	0	Confuse	0	Patterns	0	Cervical spine	0	Suicide	0	Peer support	0	boss	0	regime	0	Grandmother	0	Headache diary	0	Complications	0	Intervention	0	Angiotensin	0	Episodic	0	impairment	0	Anti-inflammatory	0	Regimen	0	Glutamate	0	Neck muscle	0	Calcium channel blocker	0	CAT scan	0	Sinus headache	0	vacation	0	NSAID	0	Accident	0	Occipital nerve	0	Motion sickness	0	Concussion	0	Remission	0	Blood flow	0	Type A	0	Facial pain	0	Lifestyle change	0	Anti-seizure	0	Upset	0	surgery	0	Prognosis	0	Rehabilitation	0	Detoxification	0	Screening	0	Inpatient
Trauma	0	Adverse effect	0	faith	0	Intractable	0	Sensitivity to sound	0	Aggravate	0	Hemiplegic migraine	0	Calcium channel	0	Risk factor	0	Toxin	0	Incidence	0	Trigger point	0	Shock	0	Hurts	0	Characteristic	0	Prophylaxis	0	Acute migraine	0	Quality of life	0	Testing	0	Weight loss	0	Agonist	0	Confuse	0	Patterns	0	Cervical spine	0	Suicide	0	Peer support	0	boss	0	regime	0	Grandmother	0	Headache diary	0	Complications	0	Intervention	0	Angiotensin	0	Episodic	0	impairment	0	Anti-inflammatory	0	Regimen	0	Glutamate	0	Neck muscle	0	Calcium channel blocker	0	CAT scan	0	Sinus headache	0	vacation	0	NSAID	0	Accident	0	Occipital nerve	0	Motion sickness	0	Concussion	0	Remission	0	Blood flow	0	Type A	0	Facial pain	0	Lifestyle change	0	Anti-seizure	0	Upset	0	surgery	0	Prognosis	0	Rehabilitation	0	Detoxification	0	Screening	0	Inpatient
Trauma	0	Adverse effect	0	faith	0	Intractable	0	Sensitivity to sound	0	Aggravate	0	Hemiplegic migraine	0	Calcium channel	0	Risk factor	0	Toxin	0	Incidence	0	Trigger point	0	Shock	0	Hurts	0	Characteristic	0	Prophylaxis	0	Acute migraine	0	Quality of life	0	Testing	0	Weight loss	0	Agonist	0	Confuse	0	Patterns	0	Cervical spine	0	Suicide	0	Peer support	0	boss	0	regime	0	Grandmother	0	Headache diary	0	Complications	0	Intervention	0	Angiotensin	0	Episodic	3	impairment	0	Anti-inflammatory	0	Regimen	0	Glutamate	1	Neck muscle	0	Calcium channel blocker	0	CAT scan	0	Sinus headache	0	vacation	0	NSAID	0	Accident	0	Occipital nerve	0	Motion sickness	0	Concussion	0	Remission	0	Blood flow	0	Type A	0	Facial pain	0	Lifestyle change	0	Anti-seizure	0	Upset	0	surgery	0	Prognosis	0	Rehabilitation	0	Detoxification	0	Screening	0	Inpatient
Trauma	0	Adverse effect	0	faith	0	Intractable	0	Sensitivity to sound	0	Aggravate	0	Hemiplegic migraine	0	Calcium channel	0	Risk factor	0	Toxin	0	Incidence	0	Trigger point	0	Shock	0	Hurts	0	Characteristic	0	Prophylaxis	0	Acute migraine	0	Quality of life	0	Testing	0	Weight loss	0	Agonist	0	Confuse	0	Patterns	0	Cervical spine	0	Suicide	0	Peer support	0	boss	0	regime	0	Grandmother	0	Headache diary	0	Complications	0	Intervention	0	Angiotensin	0	Episodic	0	impairment	0	Anti-inflammatory	0	Regimen	0	Glutamate	0	Neck muscle	0	Calcium channel blocker	0	CAT scan	0	Sinus headache	0	vacation	0	NSAID	0	Accident	0	Occipital nerve	0	Motion sickness	0	Concussion	0	Remission	0	Blood flow	0	Type A	0	Facial pain	0	Lifestyle change	0	Anti-seizure	0	Upset	0	surgery	0	Prognosis	0	Rehabilitation	0	Detoxification	0	Screening	0	Inpatient
Trauma	0	Adverse effect	0	faith	0	Intractable	1	Sensitivity to sound	0	Aggravate	0	Hemiplegic migraine	3	Calcium channel	0	Risk factor	0	Toxin	0	Incidence	0	Trigger point	0	Shock	0	Hurts	0	Characteristic	0	Prophylaxis	0	Acute migraine	0	Quality of life	0	Testing	0	Weight loss	0	Agonist	0	Confuse	0	Patterns	0	Cervical spine	0	Suicide	0	Peer support	0	boss	0	regime	0	Grandmother	0	Headache diary	0	Complications	0	Intervention	0	Angiotensin	0	Episodic	0	impairment	0	Anti-inflammatory	0	Regimen	0	Glutamate	0	Neck muscle	0	Calcium channel blocker	0	CAT scan	0	Sinus headache	0	vacation	0	NSAID	0	Accident	0	Occipital nerve	0	Motion sickness	0	Concussion	0	Remission	0	Blood flow	0	Type A	0	Facial pain	0	Lifestyle change	0	Anti-seizure	0	Upset	0	surgery	0	Prognosis	0	Rehabilitation	0	Detoxification	0	Screening	0	Inpatient
Trauma	0	Adverse effect	0	faith	0	Intractable	0	Sensitivity to sound	0	Aggravate	0	Hemiplegic migraine	0	Calcium channel	0	Risk factor	0	Toxin	0	Incidence	0	Trigger point	0	Shock	0	Hurts	0	Characteristic	0	Prophylaxis	0	Acute migraine	0	Quality of life	0	Testing	0	Weight loss	0	Agonist	0	Confuse	0	Patterns	0	Cervical spine	0	Suicide	0	Peer support	0	boss	0	regime	1	Grandmother	0	Headache diary	0	Complications	0	Intervention	0	Angiotensin	0	Episodic	0	impairment	0	Anti-inflammatory	0	Regimen	0	Glutamate	0	Neck muscle	0	Calcium channel blocker	0	CAT scan	0	Sinus headache	0	vacation	0	NSAID	0	Accident	0	Occipital nerve	0	Motion sickness	0	Concussion	0	Remission	0	Blood flow	0	Type A	0	Facial pain	0	Lifestyle change	0	Anti-seizure	0	Upset	0	surgery	0	Prognosis	0	Rehabilitation	0	Detoxification	0	Screening	0	Inpatient
Trauma	0	Adverse effect	0	faith	0	Intractable	0	Sensitivity to sound	0	Aggravate	0	Hemiplegic migraine	0	Calcium channel	0	Risk factor	0	Toxin	0	Incidence	0	Trigger point	0	Shock	0	Hurts	0	Characteristic	0	Prophylaxis	0	Acute migraine	0	Quality of life	3	Testing	0	Weight loss	0	Agonist	0	Confuse	0	Patterns	0	Cervical spine	0	Suicide	0	Peer support	0	boss	0	regime	0	Grandmother	0	Headache diary	0	Complications	0	Intervention	0	Angiotensin	0	Episodic	0	impairment	0	Anti-inflammatory	0	Regimen	0	Glutamate	0	Neck muscle	0	Calcium channel blocker	0	CAT scan	0	Sinus headache	0	vacation	0	NSAID	0	Accident	0	Occipital nerve	0	Motion sickness	0	Concussion	0	Remission	1	Blood flow	0	Type A	0	Facial pain	0	Lifestyle change	0	Anti-seizure	0	Upset	0	surgery	0	Prognosis	0	Rehabilitation	0	Detoxification	0	Screening	0	Inpatient
Trauma	0	Adverse effect	0	faith	0	Intractable	0	Sensitivity to sound	0	Aggravate	0	Hemiplegic migraine	0	Calcium channel	0	Risk factor	0	Toxin	0	Incidence	0	Trigger point	0	Shock	0	Hurts	0	Characteristic	0	Prophylaxis	0	Acute migraine	0	Quality of life	0	Testing	0	Weight loss	0	Agonist	0	Confuse	0	Patterns	0	Cervical spine	0	Suicide	0	Peer support	0	boss	0	regime	0	Grandmother	0	Headache diary	0	Complications	0	Intervention	0	Angiotensin	0	Episodic	0	impairment	0	Anti-inflammatory	0	Regimen	0	Glutamate	0	Neck muscle	0	Calcium channel blocker	0	CAT scan	0	Sinus headache	0	vacation	0	NSAID	0	Accident	0	Occipital nerve	0	Motion sickness	0	Concussion	0	Remission	0	Blood flow	0	Type A	0	Facial pain	0	Lifestyle change	0	Anti-seizure	0	Upset	0	surgery	0	Prognosis	0	Rehabilitation	0	Detoxification	0	Screening	0	Inpatient
Trauma	0	Adverse effect	0	faith	0	Intractable	0	Sensitivity to sound	0	Aggravate	0	Hemiplegic migraine	0	Calcium channel	1	Risk factor	0	Toxin	0	Incidence	0	Trigger point	0	Shock	0	Hurts	0	Characteristic	0	Prophylaxis	2	Acute migraine	0	Quality of life	0	Testing	0	Weight loss	0	Agonist	2	Confuse	0	Patterns	0	Cervical spine	0	Suicide	0	Peer support	0	boss	0	regime	0	Grandmother	0	Headache diary	0	Complications	0	Intervention	0	Angiotensin	0	Episodic	0	impairment	0	Anti-inflammatory	0	Regimen	0	Glutamate	0	Neck muscle	0	Calcium channel blocker	0	CAT scan	0	Sinus headache	0	vacation	0	NSAID	0	Accident	0	Occipital nerve	0	Motion sickness	0	Concussion	0	Remission	0	Blood flow	0	Type A	0	Facial pain	0	Lifestyle change	0	Anti-seizure	0	Upset	0	surgery	0	Prognosis	0	Rehabilitation	0	Detoxification	0	Screening	0	Inpatient
Trauma	0	Adverse effect	0	faith	0	Intractable	0	Sensitivity to sound	0	Aggravate	0	Hemiplegic migraine	0	Calcium channel	0	Risk factor	0	Toxin	0	Incidence	0	Trigger point	0	Shock	0	Hurts	0	Characteristic	0	Prophylaxis	0	Acute migraine	0	Quality of life	0	Testing	0	Weight loss	0	Agonist	0	Confuse	0	Patterns	0	Cervical spine	0	Suicide	0	Peer support	0	boss	0	regime	0	Grandmother	0	Headache diary	0	Complications	0	Intervention	0	Angiotensin	0	Episodic	0	impairment	0	Anti-inflammatory	0	Regimen	0	Glutamate	0	Neck muscle	0	Calcium channel blocker	0	CAT scan	0	Sinus headache	0	vacation	0	NSAID	0	Accident	1	Occipital nerve	0	Motion sickness	0	Concussion	0	Remission	0	Blood flow	0	Type A	0	Facial pain	0	Lifestyle change	0	Anti-seizure	0	Upset	0	surgery	0	Prognosis	0	Rehabilitation	0	Detoxification	0	Screening	0	Inpatient
Trauma	0	Adverse effect	0	faith	0	Intractable	0	Sensitivity to sound	0	Aggravate	0	Hemiplegic migraine	0	Calcium channel	0	Risk factor	0	Toxin	0	Incidence	0	Trigger point	0	Shock	0	Hurts	0	Characteristic	0	Prophylaxis	0	Acute migraine	0	Quality of life	0	Testing	0	Weight loss	0	Agonist	0	Confuse	0	Patterns	0	Cervical spine	0	Suicide	0	Peer support	0	boss	0	regime	0	Grandmother	0	Headache diary	0	Complications	0	Intervention	0	Angiotensin	0	Episodic	0	impairment	0	Anti-inflammatory	0	Regimen	0	Glutamate	0	Neck muscle	0	Calcium channel blocker	0	CAT scan	0	Sinus headache	0	vacation	0	NSAID	0	Accident	0	Occipital nerve	0	Motion sickness	0	Concussion	0	Remission	0	Blood flow	0	Type A	0	Facial pain	0	Lifestyle change	0	Anti-seizure	0	Upset	0	surgery	0	Prognosis	0	Rehabilitation	0	Detoxification	0	Screening	0	Inpatient
Trauma	0	Adverse effect	0	faith	0	Intractable	0	Sensitivity to sound	0	Aggravate	0	Hemiplegic migraine	0	Calcium channel	0	Risk factor	0	Toxin	1	Incidence	0	Trigger point	0	Shock	0	Hurts	0	Characteristic	0	Prophylaxis	0	Acute migraine	0	Quality of life	0	Testing	0	Weight loss	0	Agonist	0	Confuse	0	Patterns	0	Cervical spine	0	Suicide	0	Peer support	0	boss	0	regime	0	Grandmother	0	Headache diary	0	Complications	0	Intervention	0	Angiotensin	0	Episodic	0	impairment	0	Anti-inflammatory	0	Regimen	0	Glutamate	0	Neck muscle	0	Calcium channel blocker	0	CAT scan	0	Sinus headache	0	vacation	0	NSAID	1	Accident	0	Occipital nerve	0	Motion sickness	0	Concussion	0	Remission	0	Blood flow	0	Type A	0	Facial pain	0	Lifestyle change	0	Anti-seizure	0	Upset	0	surgery	0	Prognosis	0	Rehabilitation	0	Detoxification	0	Screening	0	Inpatient
Trauma	0	Adverse effect	0	faith	5	Intractable	2	Sensitivity to sound	3	Aggravate	1	Hemiplegic migraine	1	Calcium channel	1	Risk factor	0	Toxin	2	Incidence	4	Trigger point	5	Shock	4	Hurts	3	Characteristic	0	Prophylaxis	0	Acute migraine	0	Quality of life	1	Testing	1	Weight loss	2	Agonist	1	Confuse	4	Patterns	2	Cervical spine	0	Suicide	0	Peer support	0	boss	0	regime	1	Grandmother	1	Headache diary	1	Complications	0	Intervention	0	Angiotensin	0	Episodic	0	impairment	0	Anti-inflammatory	2	Regimen	1	Glutamate	2	Neck muscle	3	Calcium channel blocker	1	CAT scan	0	Sinus headache	1	vacation	2	NSAID	1	Accident	1	Occipital nerve	3	Motion sickness	0	Concussion	0	Remission	0	Blood flow	0	Type A	0	Facial pain	0	Lifestyle change	2	Anti-seizure	2	Upset	1	surgery	1	Prognosis	0	Rehabilitation	0	Detoxification	0	Screening	0	Inpatient
Trauma	0	Adverse effect	0	faith	0	Intractable	0	Sensitivity to sound	0	Aggravate	0	Hemiplegic migraine	0	Calcium channel	0	Risk factor	0	Toxin	0	Incidence	0	Trigger point	0	Shock	0	Hurts	0	Characteristic	0	Prophylaxis	0	Acute migraine	0	Quality of life	0	Testing	0	Weight loss	0	Agonist	0	Confuse	0	Patterns	0	Cervical spine	0	Suicide	0	Peer support	0	boss	0	regime	0	Grandmother	0	Headache diary	0	Complications	0	Intervention	0	Angiotensin	0	Episodic	0	impairment	0	Anti-inflammatory	0	Regimen	0	Glutamate	0	Neck muscle	0	Calcium channel blocker	0	CAT scan	0	Sinus headache	0	vacation	0	NSAID	0	Accident	0	Occipital nerve	0	Motion sickness	0	Concussion	0	Remission	0	Blood flow	0	Type A	0	Facial pain	0	Lifestyle change	0	Anti-seizure	0	Upset	0	surgery	0	Prognosis	0	Rehabilitation	0	Detoxification	0	Screening	0	Inpatient
Trauma	0	Adverse effect	0	faith	0	Intractable	0	Sensitivity to sound	0	Aggravate	0	Hemiplegic migraine	0	Calcium channel	0	Risk factor	0	Toxin	0	Incidence	0	Trigger point	0	Shock	0	Hurts	0	Characteristic	0	Prophylaxis	0	Acute migraine	0	Quality of life	0	Testing	0	Weight loss	0	Agonist	0	Confuse	0	Patterns	0	Cervical spine	0	Suicide	0	Peer support	0	boss	0	regime	0	Grandmother	0	Headache diary	0	Complications	0	Intervention	0	Angiotensin	0	Episodic	0	impairment	0	Anti-inflammatory	0	Regimen	0	Glutamate	0	Neck muscle	0	Calcium channel blocker	0	CAT scan	0	Sinus headache	0	vacation	0	NSAID	0	Accident	0	Occipital nerve	0	Motion sickness	0	Concussion	0	Remission	0	Blood flow	0	Type A	0	Facial pain	0	Lifestyle change	0	Anti-seizure	0	Upset	0	surgery	0	Prognosis	0	Rehabilitation	0	Detoxification	0	Screening	0	Inpatient
Trauma	0	Adverse effect	0	faith	0	Intractable	0	Sensitivity to sound	0	Aggravate	0	Hemiplegic migraine	0	Calcium channel	0	Risk factor	0	Toxin	0	Incidence	0	Trigger point	0	Shock	0	Hurts	1	Characteristic	0	Prophylaxis	0	Acute migraine	0	Quality of life	0	Testing	0	Weight loss	0	Agonist	0	Confuse	0	Patterns	0	Cervical spine	0	Suicide	0	Peer support	0	boss	0	regime	0	Grandmother	0	Headache diary	0	Complications	0	Intervention	0	Angiotensin	0	Episodic	0	impairment	0	Anti-inflammatory	0	Regimen	0	Glutamate	0	Neck muscle	0	Calcium channel blocker	0	CAT scan	0	Sinus headache	0	vacation	0	NSAID	0	Accident	1	Occipital nerve	0	Motion sickness	0	Concussion	0	Remission	0	Blood flow	0	Type A	0	Facial pain	0	Lifestyle change	0	Anti-seizure	0	Upset	0	surgery	0	Prognosis	0	Rehabilitation	0	Detoxification	0	Screening	0	Inpatient
Trauma	0	Adverse effect	0	faith	0	Intractable	0	Sensitivity to sound	0	Aggravate	0	Hemiplegic migraine	0	Calcium channel	0	Risk factor	0	Toxin	0	Incidence	0	Trigger point	0	Shock	0	Hurts	0	Characteristic	0	Prophylaxis	0	Acute migraine	0	Quality of life	0	Testing	0	Weight loss	0	Agonist	0	Confuse	0	Patterns	1	Cervical spine	0	Suicide	0	Peer support	0	boss	0	regime	0	Grandmother	0	Headache diary	0	Complications	0	Intervention	0	Angiotensin	0	Episodic	0	impairment	0	Anti-inflammatory	0	Regimen	0	Glutamate	0	Neck muscle	0	Calcium channel blocker	0	CAT scan	0	Sinus headache	0	vacation	0	NSAID	0	Accident	0	Occipital nerve	0	Motion sickness	0	Concussion	0	Remission	0	Blood flow	0	Type A	0	Facial pain	0	Lifestyle change	0	Anti-seizure	0	Upset	0	surgery	0	Prognosis	0	Rehabilitation	0	Detoxification	0	Screening	0	Inpatient
Trauma	0	Adverse effect	0	faith	0	Intractable	0	Sensitivity to sound	0	Aggravate	0	Hemiplegic migraine	0	Calcium channel	0	Risk factor	0	Toxin	1	Incidence	0	Trigger point	0	Shock	0	Hurts	0	Characteristic	0	Prophylaxis	0	Acute migraine	0	Quality of life	0	Testing	0	Weight loss	0	Agonist	1	Confuse	0	Patterns	0	Cervical spine	0	Suicide	0	Peer support	0	boss	0	regime	1	Grandmother	0	Headache diary	0	Complications	0	Intervention	0	Angiotensin	0	Episodic	0	impairment	0	Anti-inflammatory	0	Regimen	1	Glutamate	0	Neck muscle	0	Calcium channel blocker	0	CAT scan	0	Sinus headache	0	vacation	0	NSAID	0	Accident	1	Occipital nerve	0	Motion sickness	0	Concussion	5	Remission	0	Blood flow	0	Type A	0	Facial pain	0	Lifestyle change	0	Anti-seizure	0	Upset	0	surgery	1	Prognosis	0	Rehabilitation	0	Detoxification	0	Screening	0	Inpatient
Trauma	0	Adverse effect	0	faith	0	Intractable	0	Sensitivity to sound	0	Aggravate	0	Hemiplegic migraine	0	Calcium channel	0	Risk factor	0	Toxin	2	Incidence	0	Trigger point	0	Shock	0	Hurts	0	Characteristic	0	Prophylaxis	0	Acute migraine	0	Quality of life	0	Testing	2	Weight loss	1	Agonist	0	Confuse	0	Patterns	0	Cervical spine	0	Suicide	0	Peer support	0	boss	0	regime	0	Grandmother	0	Headache diary	0	Complications	0	Intervention	0	Angiotensin	0	Episodic	0	impairment	0	Anti-inflammatory	0	Regimen	0	Glutamate	0	Neck muscle	0	Calcium channel blocker	0	CAT scan	0	Sinus headache	0	vacation	0	NSAID	0	Accident	0	Occipital nerve	0	Motion sickness	0	Concussion	0	Remission	0	Blood flow	0	Type A	0	Facial pain	0	Lifestyle change	0	Anti-seizure	0	Upset	0	surgery	0	Prognosis	0	Rehabilitation	0	Detoxification	0	Screening	0	Inpatient
Trauma	0	Adverse effect	0	faith	0	Intractable	0	Sensitivity to sound	0	Aggravate	0	Hemiplegic migraine	0	Calcium channel	0	Risk factor	0	Toxin	0	Incidence	0	Trigger point	0	Shock	0	Hurts	0	Characteristic	0	Prophylaxis	0	Acute migraine	0	Quality of life	0	Testing	0	Weight loss	0	Agonist	0	Confuse	0	Patterns	0	Cervical spine	0	Suicide	0	Peer support	0	boss	0	regime	0	Grandmother	0	Headache diary	0	Complications	0	Intervention	0	Angiotensin	0	Episodic	0	impairment	0	Anti-inflammatory	0	Regimen	0	Glutamate	0	Neck muscle	0	Calcium channel blocker	0	CAT scan	0	Sinus headache	0	vacation	0	NSAID	0	Accident	0	Occipital nerve	0	Motion sickness	0	Concussion	0	Remission	0	Blood flow	1	Type A	0	Facial pain	0	Lifestyle change	0	Anti-seizure	0	Upset	1	surgery	0	Prognosis	0	Rehabilitation	0	Detoxification	0	Screening	0	Inpatient
Trauma	0	Adverse effect	0	faith	0	Intractable	0	Sensitivity to sound	0	Aggravate	1	Hemiplegic migraine	0	Calcium channel	0	Risk factor	0	Toxin	0	Incidence	0	Trigger point	0	Shock	0	Hurts	0	Characteristic	0	Prophylaxis	0	Acute migraine	0	Quality of life	0	Testing	0	Weight loss	0	Agonist	0	Confuse	0	Patterns	0	Cervical spine	0	Suicide	0	Peer support	0	boss	0	regime	0	Grandmother	0	Headache diary	0	Complications	0	Intervention	0	Angiotensin	0	Episodic	0	impairment	0	Anti-inflammatory	1	Regimen	0	Glutamate	0	Neck muscle	0	Calcium channel blocker	0	CAT scan	1	Sinus headache	0	vacation	0	NSAID	0	Accident	0	Occipital nerve	0	Motion sickness	0	Concussion	0	Remission	0	Blood flow	0	Type A	0	Facial pain	0	Lifestyle change	0	Anti-seizure	0	Upset	0	surgery	0	Prognosis	0	Rehabilitation	0	Detoxification	0	Screening	0	Inpatient
Trauma	0	Adverse effect	4	faith	0	Intractable	0	Sensitivity to sound	0	Aggravate	0	Hemiplegic migraine	0	Calcium channel	0	Risk factor	0	Toxin	0	Incidence	0	Trigger point	0	Shock	0	Hurts	0	Characteristic	0	Prophylaxis	0	Acute migraine	0	Quality of life	0	Testing	0	Weight loss	0	Agonist	0	Confuse	0	Patterns	0	Cervical spine	0	Suicide	0	Peer support	0	boss	0	regime	0	Grandmother	3	Headache diary	0	Complications	0	Intervention	0	Angiotensin	0	Episodic	0	impairment	3	Anti-inflammatory	0	Regimen	0	Glutamate	1	Neck muscle	0	Calcium channel blocker	0	CAT scan	0	Sinus headache	0	vacation	0	NSAID	0	Accident	0	Occipital nerve	0	Motion sickness	0	Concussion	0	Remission	0	Blood flow	0	Type A	0	Facial pain	0	Lifestyle change	0	Anti-seizure	0	Upset	0	surgery	0	Prognosis	0	Rehabilitation	0	Detoxification	0	Screening	0	Inpatient
Trauma	0	Adverse effect	0	faith	0	Intractable	0	Sensitivity to sound	0	Aggravate	1	Hemiplegic migraine	0	Calcium channel	0	Risk factor	0	Toxin	0	Incidence	0	Trigger point	0	Shock	0	Hurts	0	Characteristic	0	Prophylaxis	0	Acute migraine	0	Quality of life	0	Testing	2	Weight loss	0	Agonist	0	Confuse	0	Patterns	1	Cervical spine	0	Suicide	0	Peer support	0	boss	0	regime	0	Grandmother	0	Headache diary	2	Complications	0	Intervention	0	Angiotensin	0	Episodic	0	impairment	0	Anti-inflammatory	0	Regimen	0	Glutamate	0	Neck muscle	0	Calcium channel blocker	0	CAT scan	1	Sinus headache	1	vacation	0	NSAID	0	Accident	0	Occipital nerve	0	Motion sickness	0	Concussion	0	Remission	0	Blood flow	0	Type A	1	Facial pain	1	Lifestyle change	0	Anti-seizure	0	Upset	0	surgery	1	Prognosis	0	Rehabilitation	0	Detoxification	0	Screening	0	Inpatient
Trauma	0	Adverse effect	0	faith	0	Intractable	0	Sensitivity to sound	0	Aggravate	0	Hemiplegic migraine	0	Calcium channel	0	Risk factor	0	Toxin	0	Incidence	2	Trigger point	0	Shock	0	Hurts	0	Characteristic	0	Prophylaxis	0	Acute migraine	0	Quality of life	0	Testing	0	Weight loss	0	Agonist	0	Confuse	1	Patterns	1	Cervical spine	0	Suicide	0	Peer support	0	boss	0	regime	0	Grandmother	0	Headache diary	0	Complications	0	Intervention	0	Angiotensin	0	Episodic	0	impairment	0	Anti-inflammatory	0	Regimen	0	Glutamate	0	Neck muscle	0	Calcium channel blocker	0	CAT scan	1	Sinus headache	0	vacation	0	NSAID	0	Accident	0	Occipital nerve	0	Motion sickness	0	Concussion	0	Remission	0	Blood flow	0	Type A	0	Facial pain	0	Lifestyle change	0	Anti-seizure	0	Upset	1	surgery	0	Prognosis	0	Rehabilitation	0	Detoxification	0	Screening	0	Inpatient
Trauma	0	Adverse effect	0	faith	0	Intractable	0	Sensitivity to sound	0	Aggravate	0	Hemiplegic migraine	0	Calcium channel	0	Risk factor	0	Toxin	0	Incidence	0	Trigger point	0	Shock	0	Hurts	0	Characteristic	0	Prophylaxis	0	Acute migraine	0	Quality of life	0	Testing	0	Weight loss	0	Agonist	0	Confuse	0	Patterns	0	Cervical spine	0	Suicide	0	Peer support	0	boss	0	regime	0	Grandmother	0	Headache diary	0	Complications	0	Intervention	0	Angiotensin	0	Episodic	0	impairment	0	Anti-inflammatory	0	Regimen	0	Glutamate	0	Neck muscle	0	Calcium channel blocker	0	CAT scan	0	Sinus headache	0	vacation	0	NSAID	0	Accident	0	Occipital nerve	0	Motion sickness	0	Concussion	0	Remission	0	Blood flow	0	Type A	0	Facial pain	0	Lifestyle change	0	Anti-seizure	0	Upset	0	surgery	0	Prognosis	0	Rehabilitation	0	Detoxification	0	Screening	0	Inpatient
Trauma	0	Adverse effect	0	faith	0	Intractable	0	Sensitivity to sound	0	Aggravate	0	Hemiplegic migraine	0	Calcium channel	0	Risk factor	0	Toxin	0	Incidence	0	Trigger point	0	Shock	0	Hurts	0	Characteristic	0	Prophylaxis	0	Acute migraine	0	Quality of life	0	Testing	0	Weight loss	0	Agonist	0	Confuse	0	Patterns	0	Cervical spine	0	Suicide	0	Peer support	0	boss	2	regime	1	Grandmother	0	Headache diary	0	Complications	0	Intervention	0	Angiotensin	0	Episodic	0	impairment	0	Anti-inflammatory	0	Regimen	1	Glutamate	0	Neck muscle	0	Calcium channel blocker	0	CAT scan	0	Sinus headache	0	vacation	0	NSAID	0	Accident	0	Occipital nerve	0	Motion sickness	0	Concussion	0	Remission	0	Blood flow	0	Type A	0	Facial pain	0	Lifestyle change	0	Anti-seizure	0	Upset	0	surgery	0	Prognosis	0	Rehabilitation	0	Detoxification	0	Screening	0	Inpatient
Trauma	0	Adverse effect	0	faith	0	Intractable	0	Sensitivity to sound	0	Aggravate	0	Hemiplegic migraine	0	Calcium channel	0	Risk factor	0	Toxin	0	Incidence	0	Trigger point	0	Shock	0	Hurts	0	Characteristic	0	Prophylaxis	0	Acute migraine	0	Quality of life	0	Testing	0	Weight loss	0	Agonist	0	Confuse	0	Patterns	0	Cervical spine	0	Suicide	0	Peer support	0	boss	0	regime	1	Grandmother	0	Headache diary	0	Complications	0	Intervention	0	Angiotensin	0	Episodic	0	impairment	0	Anti-inflammatory	0	Regimen	1	Glutamate	0	Neck muscle	0	Calcium channel blocker	0	CAT scan	0	Sinus headache	0	vacation	0	NSAID	0	Accident	0	Occipital nerve	0	Motion sickness	0	Concussion	0	Remission	0	Blood flow	0	Type A	0	Facial pain	0	Lifestyle change	0	Anti-seizure	0	Upset	0	surgery	0	Prognosis	0	Rehabilitation	0	Detoxification	0	Screening	0	Inpatient
Trauma	0	Adverse effect	0	faith	0	Intractable	0	Sensitivity to sound	0	Aggravate	0	Hemiplegic migraine	0	Calcium channel	0	Risk factor	0	Toxin	0	Incidence	0	Trigger point	0	Shock	0	Hurts	0	Characteristic	0	Prophylaxis	0	Acute migraine	0	Quality of life	0	Testing	0	Weight loss	0	Agonist	0	Confuse	0	Patterns	0	Cervical spine	0	Suicide	0	Peer support	0	boss	0	regime	0	Grandmother	0	Headache diary	0	Complications	0	Intervention	0	Angiotensin	0	Episodic	0	impairment	0	Anti-inflammatory	1	Regimen	0	Glutamate	0	Neck muscle	0	Calcium channel blocker	0	CAT scan	0	Sinus headache	0	vacation	0	NSAID	0	Accident	0	Occipital nerve	0	Motion sickness	0	Concussion	0	Remission	0	Blood flow	0	Type A	0	Facial pain	0	Lifestyle change	0	Anti-seizure	0	Upset	0	surgery	0	Prognosis	0	Rehabilitation	0	Detoxification	0	Screening	0	Inpatient
Trauma	0	Adverse effect	0	faith	0	Intractable	0	Sensitivity to sound	0	Aggravate	0	Hemiplegic migraine	0	Calcium channel	0	Risk factor	0	Toxin	0	Incidence	0	Trigger point	0	Shock	0	Hurts	0	Characteristic	0	Prophylaxis	0	Acute migraine	0	Quality of life	0	Testing	0	Weight loss	0	Agonist	0	Confuse	0	Patterns	0	Cervical spine	0	Suicide	0	Peer support	0	boss	0	regime	0	Grandmother	0	Headache diary	0	Complications	0	Intervention	0	Angiotensin	0	Episodic	0	impairment	0	Anti-inflammatory	0	Regimen	0	Glutamate	0	Neck muscle	0	Calcium channel blocker	0	CAT scan	0	Sinus headache	0	vacation	0	NSAID	0	Accident	0	Occipital nerve	0	Motion sickness	0	Concussion	0	Remission	0	Blood flow	0	Type A	0	Facial pain	0	Lifestyle change	0	Anti-seizure	0	Upset	0	surgery	0	Prognosis	0	Rehabilitation	0	Detoxification	0	Screening	0	Inpatient
Trauma	0	Adverse effect	0	faith	0	Intractable	0	Sensitivity to sound	0	Aggravate	0	Hemiplegic migraine	0	Calcium channel	0	Risk factor	0	Toxin	0	Incidence	0	Trigger point	0	Shock	0	Hurts	0	Characteristic	0	Prophylaxis	0	Acute migraine	0	Quality of life	0	Testing	0	Weight loss	0	Agonist	0	Confuse	0	Patterns	0	Cervical spine	0	Suicide	0	Peer support	0	boss	0	regime	0	Grandmother	0	Headache diary	0	Complications	0	Intervention	0	Angiotensin	0	Episodic	1	impairment	0	Anti-inflammatory	0	Regimen	0	Glutamate	0	Neck muscle	0	Calcium channel blocker	0	CAT scan	0	Sinus headache	0	vacation	0	NSAID	0	Accident	0	Occipital nerve	0	Motion sickness	0	Concussion	0	Remission	0	Blood flow	0	Type A	0	Facial pain	0	Lifestyle change	0	Anti-seizure	0	Upset	0	surgery	0	Prognosis	0	Rehabilitation	0	Detoxification	0	Screening	0	Inpatient
Trauma	1	Adverse effect	0	faith	0	Intractable	0	Sensitivity to sound	0	Aggravate	0	Hemiplegic migraine	0	Calcium channel	0	Risk factor	0	Toxin	0	Incidence	0	Trigger point	0	Shock	0	Hurts	0	Characteristic	0	Prophylaxis	0	Acute migraine	0	Quality of life	1	Testing	0	Weight loss	0	Agonist	0	Confuse	0	Patterns	0	Cervical spine	0	Suicide	0	Peer support	0	boss	0	regime	0	Grandmother	0	Headache diary	0	Complications	0	Intervention	0	Angiotensin	0	Episodic	0	impairment	0	Anti-inflammatory	0	Regimen	0	Glutamate	0	Neck muscle	0	Calcium channel blocker	0	CAT scan	0	Sinus headache	0	vacation	0	NSAID	0	Accident	0	Occipital nerve	0	Motion sickness	0	Concussion	0	Remission	0	Blood flow	1	Type A	0	Facial pain	0	Lifestyle change	0	Anti-seizure	0	Upset	0	surgery	0	Prognosis	0	Rehabilitation	0	Detoxification	0	Screening	0	Inpatient
Trauma	1	Adverse effect	0	faith	0	Intractable	0	Sensitivity to sound	0	Aggravate	0	Hemiplegic migraine	0	Calcium channel	0	Risk factor	0	Toxin	0	Incidence	0	Trigger point	0	Shock	0	Hurts	0	Characteristic	0	Prophylaxis	0	Acute migraine	0	Quality of life	1	Testing	0	Weight loss	0	Agonist	0	Confuse	0	Patterns	0	Cervical spine	0	Suicide	0	Peer support	0	boss	0	regime	0	Grandmother	0	Headache diary	1	Complications	0	Intervention	0	Angiotensin	0	Episodic	0	impairment	0	Anti-inflammatory	0	Regimen	0	Glutamate	0	Neck muscle	0	Calcium channel blocker	0	CAT scan	0	Sinus headache	0	vacation	0	NSAID	0	Accident	0	Occipital nerve	0	Motion sickness	0	Concussion	0	Remission	0	Blood flow	1	Type A	0	Facial pain	0	Lifestyle change	0	Anti-seizure	0	Upset	0	surgery	0	Prognosis	0	Rehabilitation	0	Detoxification	0	Screening	0	Inpatient
Trauma	0	Adverse effect	0	faith	0	Intractable	0	Sensitivity to sound	0	Aggravate	0	Hemiplegic migraine	0	Calcium channel	0	Risk factor	0	Toxin	0	Incidence	0	Trigger point	0	Shock	0	Hurts	0	Characteristic	0	Prophylaxis	0	Acute migraine	0	Quality of life	0	Testing	0	Weight loss	0	Agonist	0	Confuse	0	Patterns	0	Cervical spine	0	Suicide	2	Peer support	0	boss	0	regime	0	Grandmother	0	Headache diary	0	Complications	0	Intervention	0	Angiotensin	0	Episodic	0	impairment	0	Anti-inflammatory	0	Regimen	0	Glutamate	0	Neck muscle	0	Calcium channel blocker	0	CAT scan	0	Sinus headache	0	vacation	0	NSAID	0	Accident	0	Occipital nerve	0	Motion sickness	0	Concussion	0	Remission	0	Blood flow	0	Type A	0	Facial pain	0	Lifestyle change	0	Anti-seizure	0	Upset	0	surgery	0	Prognosis	0	Rehabilitation	0	Detoxification	0	Screening	0	Inpatient
Trauma	0	Adverse effect	0	faith	0	Intractable	0	Sensitivity to sound	0	Aggravate	0	Hemiplegic migraine	0	Calcium channel	0	Risk factor	0	Toxin	0	Incidence	0	Trigger point	0	Shock	0	Hurts	0	Characteristic	0	Prophylaxis	0	Acute migraine	0	Quality of life	0	Testing	0	Weight loss	0	Agonist	0	Confuse	0	Patterns	0	Cervical spine	0	Suicide	0	Peer support	0	boss	0	regime	0	Grandmother	0	Headache diary	0	Complications	0	Intervention	0	Angiotensin	0	Episodic	0	impairment	0	Anti-inflammatory	0	Regimen	0	Glutamate	0	Neck muscle	0	Calcium channel blocker	0	CAT scan	0	Sinus headache	0	vacation	0	NSAID	0	Accident	0	Occipital nerve	0	Motion sickness	0	Concussion	0	Remission	0	Blood flow	0	Type A	1	Facial pain	0	Lifestyle change	0	Anti-seizure	0	Upset	0	surgery	0	Prognosis	0	Rehabilitation	0	Detoxification	0	Screening	0	Inpatient
Trauma	0	Adverse effect	0	faith	0	Intractable	0	Sensitivity to sound	0	Aggravate	0	Hemiplegic migraine	0	Calcium channel	0	Risk factor	0	Toxin	0	Incidence	0	Trigger point	0	Shock	0	Hurts	0	Characteristic	0	Prophylaxis	0	Acute migraine	0	Quality of life	0	Testing	0	Weight loss	0	Agonist	0	Confuse	0	Patterns	0	Cervical spine	0	Suicide	0	Peer support	0	boss	0	regime	0	Grandmother	0	Headache diary	0	Complications	0	Intervention	0	Angiotensin	0	Episodic	0	impairment	0	Anti-inflammatory	0	Regimen	0	Glutamate	0	Neck muscle	0	Calcium channel blocker	0	CAT scan	0	Sinus headache	0	vacation	0	NSAID	0	Accident	0	Occipital nerve	0	Motion sickness	0	Concussion	0	Remission	0	Blood flow	0	Type A	0	Facial pain	0	Lifestyle change	0	Anti-seizure	0	Upset	0	surgery	0	Prognosis	0	Rehabilitation	0	Detoxification	0	Screening	0	Inpatient
Trauma	0	Adverse effect	0	faith	0	Intractable	0	Sensitivity to sound	0	Aggravate	3	Hemiplegic migraine	0	Calcium channel	0	Risk factor	0	Toxin	0	Incidence	0	Trigger point	0	Shock	0	Hurts	0	Characteristic	4	Prophylaxis	0	Acute migraine	0	Quality of life	0	Testing	0	Weight loss	0	Agonist	0	Confuse	0	Patterns	0	Cervical spine	4	Suicide	0	Peer support	0	boss	2	regime	0	Grandmother	0	Headache diary	0	Complications	0	Intervention	0	Angiotensin	0	Episodic	0	impairment	0	Anti-inflammatory	0	Regimen	0	Glutamate	0	Neck muscle	0	Calcium channel blocker	0	CAT scan	0	Sinus headache	0	vacation	0	NSAID	0	Accident	0	Occipital nerve	0	Motion sickness	0	Concussion	0	Remission	0	Blood flow	0	Type A	0	Facial pain	0	Lifestyle change	0	Anti-seizure	0	Upset	0	surgery	0	Prognosis	0	Rehabilitation	0	Detoxification	0	Screening	0	Inpatient
Trauma	0	Adverse effect	0	faith	0	Intractable	0	Sensitivity to sound	0	Aggravate	0	Hemiplegic migraine	0	Calcium channel	0	Risk factor	0	Toxin	0	Incidence	0	Trigger point	0	Shock	0	Hurts	0	Characteristic	0	Prophylaxis	0	Acute migraine	0	Quality of life	0	Testing	0	Weight loss	0	Agonist	0	Confuse	0	Patterns	0	Cervical spine	0	Suicide	3	Peer support	0	boss	0	regime	0	Grandmother	0	Headache diary	0	Complications	0	Intervention	0	Angiotensin	0	Episodic	0	impairment	0	Anti-inflammatory	0	Regimen	0	Glutamate	0	Neck muscle	0	Calcium channel blocker	0	CAT scan	0	Sinus headache	0	vacation	2	NSAID	0	Accident	1	Occipital nerve	0	Motion sickness	0	Concussion	0	Remission	0	Blood flow	0	Type A	0	Facial pain	0	Lifestyle change	0	Anti-seizure	0	Upset	0	surgery	0	Prognosis	0	Rehabilitation	0	Detoxification	0	Screening	0	Inpatient
Trauma	0	Adverse effect	0	faith	0	Intractable	0	Sensitivity to sound	0	Aggravate	0	Hemiplegic migraine	0	Calcium channel	0	Risk factor	0	Toxin	0	Incidence	0	Trigger point	0	Shock	0	Hurts	0	Characteristic	0	Prophylaxis	0	Acute migraine	0	Quality of life	0	Testing	0	Weight loss	0	Agonist	0	Confuse	0	Patterns	0	Cervical spine	0	Suicide	0	Peer support	0	boss	0	regime	0	Grandmother	0	Headache diary	0	Complications	0	Intervention	0	Angiotensin	0	Episodic	0	impairment	0	Anti-inflammatory	0	Regimen	0	Glutamate	0	Neck muscle	0	Calcium channel blocker	0	CAT scan	0	Sinus headache	0	vacation	0	NSAID	0	Accident	0	Occipital nerve	0	Motion sickness	0	Concussion	0	Remission	0	Blood flow	0	Type A	0	Facial pain	0	Lifestyle change	0	Anti-seizure	0	Upset	0	surgery	0	Prognosis	0	Rehabilitation	0	Detoxification	0	Screening	0	Inpatient
Trauma	0	Adverse effect	0	faith	0	Intractable	0	Sensitivity to sound	0	Aggravate	0	Hemiplegic migraine	0	Calcium channel	0	Risk factor	0	Toxin	1	Incidence	0	Trigger point	0	Shock	0	Hurts	0	Characteristic	0	Prophylaxis	0	Acute migraine	0	Quality of life	0	Testing	0	Weight loss	0	Agonist	0	Confuse	0	Patterns	0	Cervical spine	0	Suicide	0	Peer support	0	boss	0	regime	0	Grandmother	0	Headache diary	0	Complications	0	Intervention	0	Angiotensin	0	Episodic	0	impairment	0	Anti-inflammatory	0	Regimen	0	Glutamate	0	Neck muscle	0	Calcium channel blocker	0	CAT scan	0	Sinus headache	0	vacation	0	NSAID	0	Accident	0	Occipital nerve	0	Motion sickness	0	Concussion	0	Remission	0	Blood flow	0	Type A	0	Facial pain	0	Lifestyle change	0	Anti-seizure	0	Upset	0	surgery	0	Prognosis	0	Rehabilitation	0	Detoxification	0	Screening	0	Inpatient
Trauma	0	Adverse effect	0	faith	0	Intractable	0	Sensitivity to sound	1	Aggravate	0	Hemiplegic migraine	0	Calcium channel	0	Risk factor	0	Toxin	0	Incidence	0	Trigger point	0	Shock	1	Hurts	0	Characteristic	0	Prophylaxis	0	Acute migraine	0	Quality of life	0	Testing	0	Weight loss	0	Agonist	0	Confuse	0	Patterns	0	Cervical spine	0	Suicide	0	Peer support	0	boss	0	regime	0	Grandmother	0	Headache diary	0	Complications	0	Intervention	0	Angiotensin	0	Episodic	1	impairment	0	Anti-inflammatory	0	Regimen	0	Glutamate	0	Neck muscle	0	Calcium channel blocker	0	CAT scan	0	Sinus headache	0	vacation	0	NSAID	0	Accident	0	Occipital nerve	0	Motion sickness	0	Concussion	0	Remission	0	Blood flow	0	Type A	0	Facial pain	0	Lifestyle change	0	Anti-seizure	0	Upset	0	surgery	0	Prognosis	0	Rehabilitation	0	Detoxification	0	Screening	0	Inpatient
Trauma	0	Adverse effect	0	faith	1	Intractable	6	Sensitivity to sound	0	Aggravate	0	Hemiplegic migraine	0	Calcium channel	2	Risk factor	0	Toxin	0	Incidence	0	Trigger point	0	Shock	0	Hurts	0	Characteristic	0	Prophylaxis	0	Acute migraine	0	Quality of life	0	Testing	0	Weight loss	0	Agonist	0	Confuse	0	Patterns	0	Cervical spine	0	Suicide	0	Peer support	0	boss	0	regime	0	Grandmother	0	Headache diary	0	Complications	0	Intervention	0	Angiotensin	0	Episodic	0	impairment	0	Anti-inflammatory	0	Regimen	0	Glutamate	0	Neck muscle	0	Calcium channel blocker	2	CAT scan	0	Sinus headache	0	vacation	0	NSAID	0	Accident	0	Occipital nerve	0	Motion sickness	0	Concussion	0	Remission	0	Blood flow	0	Type A	0	Facial pain	3	Lifestyle change	0	Anti-seizure	0	Upset	0	surgery	0	Prognosis	0	Rehabilitation	0	Detoxification	0	Screening	0	Inpatient
Trauma	0	Adverse effect	0	faith	0	Intractable	0	Sensitivity to sound	0	Aggravate	0	Hemiplegic migraine	0	Calcium channel	0	Risk factor	1	Toxin	0	Incidence	0	Trigger point	0	Shock	0	Hurts	0	Characteristic	0	Prophylaxis	0	Acute migraine	0	Quality of life	0	Testing	1	Weight loss	0	Agonist	0	Confuse	0	Patterns	0	Cervical spine	0	Suicide	0	Peer support	0	boss	0	regime	0	Grandmother	0	Headache diary	0	Complications	0	Intervention	0	Angiotensin	0	Episodic	0	impairment	0	Anti-inflammatory	0	Regimen	0	Glutamate	0	Neck muscle	0	Calcium channel blocker	0	CAT scan	0	Sinus headache	0	vacation	0	NSAID	0	Accident	0	Occipital nerve	0	Motion sickness	0	Concussion	0	Remission	0	Blood flow	0	Type A	0	Facial pain	0	Lifestyle change	0	Anti-seizure	0	Upset	0	surgery	0	Prognosis	0	Rehabilitation	0	Detoxification	0	Screening	1	Inpatient
Trauma	0	Adverse effect	0	faith	0	Intractable	0	Sensitivity to sound	0	Aggravate	0	Hemiplegic migraine	0	Calcium channel	0	Risk factor	1	Toxin	0	Incidence	0	Trigger point	0	Shock	0	Hurts	0	Characteristic	1	Prophylaxis	0	Acute migraine	0	Quality of life	0	Testing	0	Weight loss	0	Agonist	0	Confuse	0	Patterns	0	Cervical spine	0	Suicide	0	Peer support	0	boss	0	regime	0	Grandmother	0	Headache diary	0	Complications	0	Intervention	0	Angiotensin	0	Episodic	0	impairment	0	Anti-inflammatory	0	Regimen	0	Glutamate	0	Neck muscle	0	Calcium channel blocker	0	CAT scan	0	Sinus headache	0	vacation	0	NSAID	0	Accident	0	Occipital nerve	0	Motion sickness	0	Concussion	0	Remission	0	Blood flow	0	Type A	0	Facial pain	0	Lifestyle change	0	Anti-seizure	0	Upset	0	surgery	0	Prognosis	0	Rehabilitation	0	Detoxification	0	Screening	0	Inpatient
Trauma	0	Adverse effect	0	faith	0	Intractable	0	Sensitivity to sound	0	Aggravate	0	Hemiplegic migraine	0	Calcium channel	0	Risk factor	0	Toxin	0	Incidence	0	Trigger point	0	Shock	0	Hurts	1	Characteristic	0	Prophylaxis	0	Acute migraine	0	Quality of life	0	Testing	0	Weight loss	0	Agonist	0	Confuse	0	Patterns	0	Cervical spine	0	Suicide	0	Peer support	0	boss	0	regime	0	Grandmother	0	Headache diary	0	Complications	0	Intervention	0	Angiotensin	0	Episodic	0	impairment	0	Anti-inflammatory	0	Regimen	0	Glutamate	0	Neck muscle	0	Calcium channel blocker	0	CAT scan	2	Sinus headache	0	vacation	0	NSAID	0	Accident	0	Occipital nerve	0	Motion sickness	0	Concussion	0	Remission	0	Blood flow	0	Type A	0	Facial pain	0	Lifestyle change	0	Anti-seizure	0	Upset	0	surgery	0	Prognosis	0	Rehabilitation	0	Detoxification	0	Screening	0	Inpatient
Trauma	0	Adverse effect	0	faith	0	Intractable	0	Sensitivity to sound	0	Aggravate	0	Hemiplegic migraine	0	Calcium channel	0	Risk factor	0	Toxin	0	Incidence	0	Trigger point	0	Shock	0	Hurts	0	Characteristic	0	Prophylaxis	0	Acute migraine	0	Quality of life	0	Testing	0	Weight loss	0	Agonist	0	Confuse	0	Patterns	0	Cervical spine	0	Suicide	0	Peer support	0	boss	0	regime	0	Grandmother	0	Headache diary	0	Complications	0	Intervention	0	Angiotensin	0	Episodic	0	impairment	0	Anti-inflammatory	0	Regimen	0	Glutamate	0	Neck muscle	0	Calcium channel blocker	0	CAT scan	0	Sinus headache	0	vacation	0	NSAID	0	Accident	0	Occipital nerve	0	Motion sickness	0	Concussion	0	Remission	0	Blood flow	0	Type A	0	Facial pain	0	Lifestyle change	0	Anti-seizure	0	Upset	0	surgery	0	Prognosis	0	Rehabilitation	0	Detoxification	0	Screening	0	Inpatient
Trauma	0	Adverse effect	0	faith	0	Intractable	0	Sensitivity to sound	0	Aggravate	0	Hemiplegic migraine	0	Calcium channel	0	Risk factor	0	Toxin	0	Incidence	0	Trigger point	0	Shock	0	Hurts	0	Characteristic	0	Prophylaxis	0	Acute migraine	0	Quality of life	0	Testing	0	Weight loss	0	Agonist	0	Confuse	0	Patterns	0	Cervical spine	0	Suicide	0	Peer support	0	boss	0	regime	0	Grandmother	0	Headache diary	0	Complications	0	Intervention	0	Angiotensin	0	Episodic	0	impairment	0	Anti-inflammatory	0	Regimen	0	Glutamate	0	Neck muscle	0	Calcium channel blocker	0	CAT scan	0	Sinus headache	0	vacation	0	NSAID	0	Accident	0	Occipital nerve	0	Motion sickness	5	Concussion	0	Remission	0	Blood flow	0	Type A	0	Facial pain	0	Lifestyle change	0	Anti-seizure	0	Upset	0	surgery	0	Prognosis	0	Rehabilitation	0	Detoxification	0	Screening	0	Inpatient
Trauma	0	Adverse effect	0	faith	0	Intractable	0	Sensitivity to sound	0	Aggravate	0	Hemiplegic migraine	0	Calcium channel	0	Risk factor	0	Toxin	0	Incidence	0	Trigger point	0	Shock	0	Hurts	0	Characteristic	0	Prophylaxis	0	Acute migraine	0	Quality of life	0	Testing	0	Weight loss	0	Agonist	0	Confuse	0	Patterns	0	Cervical spine	0	Suicide	0	Peer support	0	boss	0	regime	0	Grandmother	0	Headache diary	0	Complications	0	Intervention	0	Angiotensin	0	Episodic	0	impairment	0	Anti-inflammatory	0	Regimen	0	Glutamate	0	Neck muscle	0	Calcium channel blocker	0	CAT scan	0	Sinus headache	0	vacation	0	NSAID	0	Accident	0	Occipital nerve	0	Motion sickness	0	Concussion	0	Remission	0	Blood flow	0	Type A	0	Facial pain	0	Lifestyle change	0	Anti-seizure	0	Upset	0	surgery	0	Prognosis	0	Rehabilitation	0	Detoxification	0	Screening	0	Inpatient
Trauma	0	Adverse effect	0	faith	0	Intractable	0	Sensitivity to sound	0	Aggravate	0	Hemiplegic migraine	1	Calcium channel	0	Risk factor	0	Toxin	0	Incidence	0	Trigger point	0	Shock	0	Hurts	0	Characteristic	0	Prophylaxis	0	Acute migraine	0	Quality of life	0	Testing	0	Weight loss	0	Agonist	0	Confuse	0	Patterns	0	Cervical spine	0	Suicide	0	Peer support	0	boss	0	regime	0	Grandmother	0	Headache diary	0	Complications	0	Intervention	0	Angiotensin	0	Episodic	0	impairment	0	Anti-inflammatory	0	Regimen	0	Glutamate	0	Neck muscle	0	Calcium channel blocker	0	CAT scan	0	Sinus headache	0	vacation	0	NSAID	0	Accident	0	Occipital nerve	0	Motion sickness	0	Concussion	0	Remission	0	Blood flow	0	Type A	0	Facial pain	0	Lifestyle change	0	Anti-seizure	0	Upset	0	surgery	0	Prognosis	0	Rehabilitation	0	Detoxification	0	Screening	0	Inpatient
Trauma	0	Adverse effect	0	faith	0	Intractable	0	Sensitivity to sound	0	Aggravate	0	Hemiplegic migraine	0	Calcium channel	0	Risk factor	1	Toxin	0	Incidence	0	Trigger point	0	Shock	0	Hurts	0	Characteristic	0	Prophylaxis	0	Acute migraine	2	Quality of life	0	Testing	0	Weight loss	0	Agonist	0	Confuse	0	Patterns	0	Cervical spine	0	Suicide	0	Peer support	0	boss	0	regime	0	Grandmother	0	Headache diary	0	Complications	0	Intervention	0	Angiotensin	0	Episodic	0	impairment	0	Anti-inflammatory	0	Regimen	0	Glutamate	0	Neck muscle	0	Calcium channel blocker	0	CAT scan	0	Sinus headache	0	vacation	0	NSAID	0	Accident	0	Occipital nerve	0	Motion sickness	0	Concussion	0	Remission	0	Blood flow	0	Type A	0	Facial pain	0	Lifestyle change	0	Anti-seizure	0	Upset	0	surgery	0	Prognosis	0	Rehabilitation	0	Detoxification	0	Screening	0	Inpatient
Trauma	2	Adverse effect	0	faith	0	Intractable	0	Sensitivity to sound	0	Aggravate	0	Hemiplegic migraine	0	Calcium channel	0	Risk factor	0	Toxin	0	Incidence	0	Trigger point	0	Shock	0	Hurts	0	Characteristic	0	Prophylaxis	0	Acute migraine	0	Quality of life	0	Testing	0	Weight loss	0	Agonist	0	Confuse	0	Patterns	0	Cervical spine	0	Suicide	0	Peer support	4	boss	0	regime	0	Grandmother	0	Headache diary	0	Complications	0	Intervention	0	Angiotensin	0	Episodic	0	impairment	0	Anti-inflammatory	0	Regimen	0	Glutamate	0	Neck muscle	0	Calcium channel blocker	0	CAT scan	0	Sinus headache	0	vacation	0	NSAID	0	Accident	0	Occipital nerve	0	Motion sickness	0	Concussion	0	Remission	0	Blood flow	0	Type A	0	Facial pain	0	Lifestyle change	0	Anti-seizure	0	Upset	0	surgery	0	Prognosis	0	Rehabilitation	0	Detoxification	0	Screening	0	Inpatient
Trauma	0	Adverse effect	2	faith	0	Intractable	0	Sensitivity to sound	0	Aggravate	0	Hemiplegic migraine	0	Calcium channel	0	Risk factor	0	Toxin	0	Incidence	0	Trigger point	0	Shock	0	Hurts	0	Characteristic	0	Prophylaxis	0	Acute migraine	0	Quality of life	0	Testing	0	Weight loss	0	Agonist	0	Confuse	0	Patterns	0	Cervical spine	0	Suicide	0	Peer support	0	boss	0	regime	0	Grandmother	0	Headache diary	0	Complications	0	Intervention	0	Angiotensin	0	Episodic	0	impairment	0	Anti-inflammatory	0	Regimen	0	Glutamate	0	Neck muscle	0	Calcium channel blocker	0	CAT scan	0	Sinus headache	0	vacation	0	NSAID	2	Accident	0	Occipital nerve	0	Motion sickness	0	Concussion	0	Remission	0	Blood flow	0	Type A	0	Facial pain	0	Lifestyle change	0	Anti-seizure	0	Upset	0	surgery	0	Prognosis	0	Rehabilitation	0	Detoxification	0	Screening	0	Inpatient
Trauma	0	Adverse effect	0	faith	0	Intractable	0	Sensitivity to sound	0	Aggravate	0	Hemiplegic migraine	4	Calcium channel	4	Risk factor	0	Toxin	1	Incidence	0	Trigger point	0	Shock	0	Hurts	0	Characteristic	0	Prophylaxis	5	Acute migraine	0	Quality of life	0	Testing	0	Weight loss	0	Agonist	2	Confuse	0	Patterns	1	Cervical spine	0	Suicide	0	Peer support	0	boss	0	regime	0	Grandmother	0	Headache diary	0	Complications	1	Intervention	1	Angiotensin	7	Episodic	0	impairment	0	Anti-inflammatory	0	Regimen	0	Glutamate	0	Neck muscle	0	Calcium channel blocker	2	CAT scan	0	Sinus headache	0	vacation	0	NSAID	0	Accident	0	Occipital nerve	0	Motion sickness	0	Concussion	0	Remission	2	Blood flow	0	Type A	0	Facial pain	0	Lifestyle change	0	Anti-seizure	1	Upset	1	surgery	0	Prognosis	0	Rehabilitation	0	Detoxification	0	Screening	0	Inpatient
Trauma	0	Adverse effect	0	faith	0	Intractable	0	Sensitivity to sound	0	Aggravate	0	Hemiplegic migraine	0	Calcium channel	0	Risk factor	0	Toxin	0	Incidence	0	Trigger point	0	Shock	0	Hurts	0	Characteristic	0	Prophylaxis	0	Acute migraine	2	Quality of life	0	Testing	0	Weight loss	0	Agonist	0	Confuse	0	Patterns	0	Cervical spine	0	Suicide	0	Peer support	0	boss	0	regime	0	Grandmother	0	Headache diary	0	Complications	0	Intervention	0	Angiotensin	0	Episodic	0	impairment	0	Anti-inflammatory	0	Regimen	0	Glutamate	0	Neck muscle	0	Calcium channel blocker	0	CAT scan	0	Sinus headache	0	vacation	0	NSAID	0	Accident	0	Occipital nerve	0	Motion sickness	0	Concussion	0	Remission	0	Blood flow	0	Type A	0	Facial pain	0	Lifestyle change	0	Anti-seizure	0	Upset	0	surgery	0	Prognosis	0	Rehabilitation	0	Detoxification	0	Screening	0	Inpatient
Trauma	3	Adverse effect	0	faith	0	Intractable	0	Sensitivity to sound	0	Aggravate	0	Hemiplegic migraine	0	Calcium channel	0	Risk factor	6	Toxin	0	Incidence	0	Trigger point	0	Shock	0	Hurts	0	Characteristic	0	Prophylaxis	0	Acute migraine	0	Quality of life	0	Testing	0	Weight loss	1	Agonist	0	Confuse	0	Patterns	0	Cervical spine	0	Suicide	0	Peer support	0	boss	0	regime	0	Grandmother	0	Headache diary	0	Complications	6	Intervention	6	Angiotensin	0	Episodic	0	impairment	0	Anti-inflammatory	1	Regimen	0	Glutamate	0	Neck muscle	0	Calcium channel blocker	0	CAT scan	0	Sinus headache	0	vacation	0	NSAID	0	Accident	0	Occipital nerve	0	Motion sickness	0	Concussion	0	Remission	2	Blood flow	0	Type A	0	Facial pain	0	Lifestyle change	1	Anti-seizure	0	Upset	0	surgery	0	Prognosis	4	Rehabilitation	4	Detoxification	4	Screening	3	Inpatient
Trauma	0	Adverse effect	1	faith	0	Intractable	0	Sensitivity to sound	0	Aggravate	0	Hemiplegic migraine	0	Calcium channel	0	Risk factor	0	Toxin	0	Incidence	0	Trigger point	0	Shock	0	Hurts	0	Characteristic	0	Prophylaxis	0	Acute migraine	0	Quality of life	1	Testing	0	Weight loss	0	Agonist	0	Confuse	0	Patterns	0	Cervical spine	0	Suicide	3	Peer support	0	boss	0	regime	0	Grandmother	0	Headache diary	0	Complications	0	Intervention	0	Angiotensin	0	Episodic	0	impairment	0	Anti-inflammatory	0	Regimen	0	Glutamate	0	Neck muscle	0	Calcium channel blocker	0	CAT scan	0	Sinus headache	0	vacation	0	NSAID	0	Accident	0	Occipital nerve	0	Motion sickness	0	Concussion	0	Remission	0	Blood flow	0	Type A	1	Facial pain	0	Lifestyle change	0	Anti-seizure	0	Upset	0	surgery	0	Prognosis	0	Rehabilitation	0	Detoxification	0	Screening	0	Inpatient
Trauma	0	Adverse effect	0	faith	0	Intractable	0	Sensitivity to sound	0	Aggravate	0	Hemiplegic migraine	0	Calcium channel	0	Risk factor	0	Toxin	0	Incidence	0	Trigger point	0	Shock	0	Hurts	0	Characteristic	0	Prophylaxis	0	Acute migraine	0	Quality of life	0	Testing	0	Weight loss	0	Agonist	0	Confuse	0	Patterns	0	Cervical spine	0	Suicide	0	Peer support	0	boss	0	regime	0	Grandmother	0	Headache diary	0	Complications	0	Intervention	0	Angiotensin	0	Episodic	0	impairment	0	Anti-inflammatory	0	Regimen	0	Glutamate	0	Neck muscle	0	Calcium channel blocker	0	CAT scan	0	Sinus headache	0	vacation	0	NSAID	0	Accident	0	Occipital nerve	0	Motion sickness	0	Concussion	0	Remission	0	Blood flow	0	Type A	0	Facial pain	0	Lifestyle change	0	Anti-seizure	0	Upset	0	surgery	0	Prognosis	0	Rehabilitation	0	Detoxification	0	Screening	0	Inpatient
Trauma	0	Adverse effect	0	faith	0	Intractable	0	Sensitivity to sound	0	Aggravate	0	Hemiplegic migraine	0	Calcium channel	0	Risk factor	0	Toxin	0	Incidence	0	Trigger point	0	Shock	0	Hurts	0	Characteristic	0	Prophylaxis	0	Acute migraine	0	Quality of life	0	Testing	0	Weight loss	0	Agonist	0	Confuse	0	Patterns	0	Cervical spine	0	Suicide	0	Peer support	0	boss	0	regime	0	Grandmother	0	Headache diary	0	Complications	0	Intervention	0	Angiotensin	0	Episodic	0	impairment	0	Anti-inflammatory	0	Regimen	0	Glutamate	0	Neck muscle	0	Calcium channel blocker	0	CAT scan	0	Sinus headache	0	vacation	0	NSAID	0	Accident	0	Occipital nerve	0	Motion sickness	0	Concussion	0	Remission	0	Blood flow	0	Type A	0	Facial pain	0	Lifestyle change	0	Anti-seizure	0	Upset	0	surgery	0	Prognosis	0	Rehabilitation	0	Detoxification	0	Screening	0	Inpatient
Trauma	0	Adverse effect	0	faith	0	Intractable	0	Sensitivity to sound	4	Aggravate	0	Hemiplegic migraine	0	Calcium channel	0	Risk factor	0	Toxin	0	Incidence	0	Trigger point	0	Shock	0	Hurts	0	Characteristic	0	Prophylaxis	0	Acute migraine	0	Quality of life	0	Testing	0	Weight loss	0	Agonist	0	Confuse	0	Patterns	0	Cervical spine	0	Suicide	0	Peer support	0	boss	0	regime	0	Grandmother	0	Headache diary	0	Complications	0	Intervention	0	Angiotensin	0	Episodic	0	impairment	0	Anti-inflammatory	0	Regimen	0	Glutamate	0	Neck muscle	0	Calcium channel blocker	0	CAT scan	0	Sinus headache	0	vacation	0	NSAID	0	Accident	0	Occipital nerve	0	Motion sickness	0	Concussion	0	Remission	0	Blood flow	0	Type A	0	Facial pain	0	Lifestyle change	0	Anti-seizure	0	Upset	0	surgery	0	Prognosis	0	Rehabilitation	0	Detoxification	0	Screening	0	Inpatient
Trauma	0	Adverse effect	0	faith	0	Intractable	0	Sensitivity to sound	0	Aggravate	0	Hemiplegic migraine	0	Calcium channel	0	Risk factor	0	Toxin	0	Incidence	0	Trigger point	0	Shock	0	Hurts	0	Characteristic	0	Prophylaxis	0	Acute migraine	0	Quality of life	0	Testing	0	Weight loss	0	Agonist	0	Confuse	0	Patterns	0	Cervical spine	0	Suicide	0	Peer support	0	boss	0	regime	0	Grandmother	0	Headache diary	0	Complications	0	Intervention	0	Angiotensin	0	Episodic	0	impairment	1	Anti-inflammatory	0	Regimen	0	Glutamate	0	Neck muscle	0	Calcium channel blocker	0	CAT scan	0	Sinus headache	0	vacation	0	NSAID	0	Accident	0	Occipital nerve	0	Motion sickness	0	Concussion	0	Remission	0	Blood flow	0	Type A	0	Facial pain	0	Lifestyle change	0	Anti-seizure	0	Upset	0	surgery	0	Prognosis	0	Rehabilitation	0	Detoxification	0	Screening	0	Inpatient
Trauma	0	Adverse effect	0	faith	0	Intractable	0	Sensitivity to sound	0	Aggravate	0	Hemiplegic migraine	0	Calcium channel	0	Risk factor	0	Toxin	0	Incidence	0	Trigger point	0	Shock	0	Hurts	0	Characteristic	0	Prophylaxis	0	Acute migraine	0	Quality of life	0	Testing	0	Weight loss	0	Agonist	0	Confuse	0	Patterns	0	Cervical spine	0	Suicide	0	Peer support	0	boss	0	regime	0	Grandmother	0	Headache diary	0	Complications	0	Intervention	0	Angiotensin	0	Episodic	1	impairment	0	Anti-inflammatory	0	Regimen	0	Glutamate	0	Neck muscle	0	Calcium channel blocker	0	CAT scan	0	Sinus headache	0	vacation	0	NSAID	0	Accident	0	Occipital nerve	0	Motion sickness	0	Concussion	0	Remission	0	Blood flow	0	Type A	0	Facial pain	0	Lifestyle change	0	Anti-seizure	0	Upset	0	surgery	0	Prognosis	0	Rehabilitation	0	Detoxification	0	Screening	0	Inpatient
Trauma	0	Adverse effect	0	faith	0	Intractable	0	Sensitivity to sound	0	Aggravate	0	Hemiplegic migraine	0	Calcium channel	1	Risk factor	0	Toxin	0	Incidence	0	Trigger point	0	Shock	0	Hurts	0	Characteristic	0	Prophylaxis	0	Acute migraine	0	Quality of life	0	Testing	0	Weight loss	0	Agonist	0	Confuse	0	Patterns	0	Cervical spine	0	Suicide	0	Peer support	0	boss	0	regime	0	Grandmother	0	Headache diary	0	Complications	0	Intervention	0	Angiotensin	0	Episodic	0	impairment	0	Anti-inflammatory	0	Regimen	0	Glutamate	0	Neck muscle	0	Calcium channel blocker	1	CAT scan	0	Sinus headache	0	vacation	0	NSAID	0	Accident	0	Occipital nerve	0	Motion sickness	0	Concussion	0	Remission	0	Blood flow	0	Type A	0	Facial pain	0	Lifestyle change	0	Anti-seizure	0	Upset	0	surgery	0	Prognosis	0	Rehabilitation	0	Detoxification	0	Screening	0	Inpatient
Trauma	0	Adverse effect	0	faith	0	Intractable	0	Sensitivity to sound	0	Aggravate	3	Hemiplegic migraine	0	Calcium channel	0	Risk factor	0	Toxin	0	Incidence	0	Trigger point	0	Shock	0	Hurts	0	Characteristic	4	Prophylaxis	0	Acute migraine	0	Quality of life	0	Testing	0	Weight loss	0	Agonist	0	Confuse	0	Patterns	0	Cervical spine	4	Suicide	0	Peer support	0	boss	2	regime	0	Grandmother	0	Headache diary	0	Complications	0	Intervention	0	Angiotensin	0	Episodic	0	impairment	0	Anti-inflammatory	0	Regimen	0	Glutamate	0	Neck muscle	0	Calcium channel blocker	0	CAT scan	0	Sinus headache	0	vacation	0	NSAID	0	Accident	0	Occipital nerve	0	Motion sickness	0	Concussion	0	Remission	0	Blood flow	0	Type A	0	Facial pain	0	Lifestyle change	0	Anti-seizure	0	Upset	0	surgery	0	Prognosis	0	Rehabilitation	0	Detoxification	0	Screening	0	Inpatient
Trauma	0	Adverse effect	0	faith	0	Intractable	0	Sensitivity to sound	0	Aggravate	0	Hemiplegic migraine	0	Calcium channel	0	Risk factor	0	Toxin	0	Incidence	0	Trigger point	0	Shock	0	Hurts	0	Characteristic	0	Prophylaxis	0	Acute migraine	0	Quality of life	0	Testing	0	Weight loss	0	Agonist	0	Confuse	0	Patterns	0	Cervical spine	0	Suicide	0	Peer support	0	boss	0	regime	0	Grandmother	0	Headache diary	0	Complications	0	Intervention	0	Angiotensin	0	Episodic	0	impairment	0	Anti-inflammatory	0	Regimen	0	Glutamate	0	Neck muscle	0	Calcium channel blocker	0	CAT scan	0	Sinus headache	0	vacation	0	NSAID	0	Accident	0	Occipital nerve	0	Motion sickness	0	Concussion	0	Remission	0	Blood flow	0	Type A	0	Facial pain	0	Lifestyle change	0	Anti-seizure	0	Upset	0	surgery	0	Prognosis	0	Rehabilitation	0	Detoxification	0	Screening	0	Inpatient
Trauma	0	Adverse effect	0	faith	0	Intractable	0	Sensitivity to sound	0	Aggravate	0	Hemiplegic migraine	0	Calcium channel	1	Risk factor	0	Toxin	0	Incidence	0	Trigger point	0	Shock	0	Hurts	0	Characteristic	0	Prophylaxis	2	Acute migraine	0	Quality of life	0	Testing	0	Weight loss	0	Agonist	2	Confuse	0	Patterns	0	Cervical spine	0	Suicide	0	Peer support	0	boss	0	regime	0	Grandmother	0	Headache diary	0	Complications	0	Intervention	0	Angiotensin	0	Episodic	0	impairment	0	Anti-inflammatory	0	Regimen	0	Glutamate	0	Neck muscle	0	Calcium channel blocker	0	CAT scan	0	Sinus headache	0	vacation	0	NSAID	0	Accident	0	Occipital nerve	0	Motion sickness	0	Concussion	0	Remission	0	Blood flow	0	Type A	0	Facial pain	0	Lifestyle change	0	Anti-seizure	0	Upset	0	surgery	0	Prognosis	0	Rehabilitation	0	Detoxification	0	Screening	0	Inpatient
Trauma	0	Adverse effect	0	faith	0	Intractable	0	Sensitivity to sound	0	Aggravate	0	Hemiplegic migraine	0	Calcium channel	0	Risk factor	0	Toxin	0	Incidence	0	Trigger point	0	Shock	0	Hurts	0	Characteristic	0	Prophylaxis	0	Acute migraine	0	Quality of life	0	Testing	0	Weight loss	0	Agonist	0	Confuse	0	Patterns	0	Cervical spine	0	Suicide	0	Peer support	0	boss	0	regime	0	Grandmother	0	Headache diary	0	Complications	0	Intervention	0	Angiotensin	0	Episodic	0	impairment	0	Anti-inflammatory	0	Regimen	0	Glutamate	0	Neck muscle	0	Calcium channel blocker	0	CAT scan	0	Sinus headache	0	vacation	0	NSAID	0	Accident	0	Occipital nerve	0	Motion sickness	0	Concussion	0	Remission	0	Blood flow	0	Type A	0	Facial pain	0	Lifestyle change	0	Anti-seizure	0	Upset	0	surgery	0	Prognosis	0	Rehabilitation	0	Detoxification	0	Screening	0	Inpatient
	11		11		11		11		11		11		11		11		10		10		10		10		10		10		9		9		9		9		9		9		9		9		9		8		8		8		8		8		8		8		7		7		7		7		7		7		7		7		7		7		6		6		6		6		6		6		5		5		5		5		5		5		5		5		5		5		4		4		4		4	

P199b	P200a	P200b	P201a	P201b	P202a	P202b	P203a	P203b	P204a	P204b	P205a	P205b	P206a	P206b	P207a	P207b	P208a	P208b	P209a	P209b	P210a	P210b	P211a	P211b	P212a	P212b	P213a	P213b	P214a	P214b	P215a	P215b	P216a	P216b	P217a	P217b	P218a	P218b	P219a	P219b	P220a	P220b	P221a	P221b	P222a	P222b	P223a	P223b	P224a	P224b	P225a	P225b	P226a	P226b	P227a	P227b	P228a	P228b	P229a	P229b	P230a	P230b	P231a	P231b	P232a	P232b	P233a	P233b	P234a	P234b	P235a	P235b	P236a	P236b	P237a	P237b	P238a	P238b	P239a	P239b	P240a	P240b	P241a	P241b	P242a	P242b	P243a	P243b	P244a	P244b	P245a	P245b	P246a	P246b	P247a	P247b	P248a	P248b	P249a	P249b	P250a	P250b	P251a	P251b	P252a	P252b	P253a	P253b	P254a	P254b	P255a	P255b	P256a	P256b
0	Headache clinic	0	Assessment	0	Empower	0	Productivity	0	Caregiver	0	Nerve block	2	Accepting	2	Pain management	1	Mechanism	1	spouse	1	Co-morbid	0	Histamine	0	Tricyclic	0	Comorbid	0	Prevalence	0	Advantage	0	Peripheral	0	Dopamine	0	Physical examination	0	Referral	0	Attitude	0	Gene-related	0	Calcitonin	0	Beta-blocker	0	church	0	Temporal	1	Barbiturate	1	Lidocaine	1	Spectrum	1	Motivation	1	Menstrual migraine	1	Correlate	0	Outpatient	0	Injury	0	Lab test	0	Efficacy	0	Antihistamine	0	Lactation	0	Secondary headache	0	Trigeminal autonomic cephalalgia	0	Progression	0	Medication overuse	0	Inpatient treatment	0	Intracranial hypertension	0	Botulinum Toxin	0	Serotonin receptor	0	Mindfulness	0	Cranial nerve	0	Episodic migraine	0	Double vision	0	Vestibular migraine	0	surgical	0	Surgical treatment	0	5-HT	0	Ineffective	0	Eye symptom	0	Female hormone	0
0	Headache clinic	0	Assessment	0	Empower	0	Productivity	0	Caregiver	0	Nerve block	0	Accepting	0	Pain management	0	Mechanism	1	spouse	0	Co-morbid	0	Histamine	0	Tricyclic	0	Comorbid	0	Prevalence	0	Advantage	0	Peripheral	0	Dopamine	0	Physical examination	0	Referral	0	Attitude	0	Gene-related	0	Calcitonin	0	Beta-blocker	0	church	0	Temporal	0	Barbiturate	0	Lidocaine	0	Spectrum	0	Motivation	0	Menstrual migraine	0	Correlate	0	Outpatient	0	Injury	0	Lab test	0	Efficacy	0	Antihistamine	0	Lactation	0	Secondary headache	0	Trigeminal autonomic cephalalgia	0	Progression	0	Medication overuse	0	Inpatient treatment	0	Intracranial hypertension	0	Botulinum Toxin	0	Serotonin receptor	0	Mindfulness	0	Cranial nerve	0	Episodic migraine	0	Double vision	0	Vestibular migraine	0	surgical	0	Surgical treatment	0	5-HT	0	Ineffective	0	Eye symptom	0	Female hormone	1
0	Headache clinic	0	Assessment	0	Empower	0	Productivity	0	Caregiver	0	Nerve block	0	Accepting	0	Pain management	0	Mechanism	0	spouse	0	Co-morbid	0	Histamine	0	Tricyclic	0	Comorbid	0	Prevalence	0	Advantage	0	Peripheral	0	Dopamine	0	Physical examination	0	Referral	0	Attitude	0	Gene-related	0	Calcitonin	0	Beta-blocker	0	church	0	Temporal	0	Barbiturate	0	Lidocaine	0	Spectrum	0	Motivation	0	Menstrual migraine	0	Correlate	0	Outpatient	0	Injury	0	Lab test	0	Efficacy	0	Antihistamine	0	Lactation	0	Secondary headache	0	Trigeminal autonomic cephalalgia	0	Progression	0	Medication overuse	0	Inpatient treatment	0	Intracranial hypertension	0	Botulinum Toxin	0	Serotonin receptor	0	Mindfulness	0	Cranial nerve	0	Episodic migraine	0	Double vision	0	Vestibular migraine	0	surgical	0	Surgical treatment	0	5-HT	0	Ineffective	0	Eye symptom	0	Female hormone	0
0	Headache clinic	0	Assessment	0	Empower	0	Productivity	0	Caregiver	0	Nerve block	0	Accepting	0	Pain management	0	Mechanism	0	spouse	0	Co-morbid	0	Histamine	0	Tricyclic	0	Comorbid	0	Prevalence	0	Advantage	0	Peripheral	0	Dopamine	0	Physical examination	0	Referral	0	Attitude	0	Gene-related	0	Calcitonin	0	Beta-blocker	0	church	1	Temporal	0	Barbiturate	0	Lidocaine	0	Spectrum	0	Motivation	0	Menstrual migraine	0	Correlate	0	Outpatient	0	Injury	0	Lab test	0	Efficacy	0	Antihistamine	0	Lactation	0	Secondary headache	0	Trigeminal autonomic cephalalgia	0	Progression	0	Medication overuse	0	Inpatient treatment	0	Intracranial hypertension	0	Botulinum Toxin	0	Serotonin receptor	0	Mindfulness	0	Cranial nerve	0	Episodic migraine	0	Double vision	0	Vestibular migraine	0	surgical	0	Surgical treatment	0	5-HT	1	Ineffective	1	Eye symptom	1	Female hormone	0
0	Headache clinic	0	Assessment	0	Empower	0	Productivity	0	Caregiver	0	Nerve block	0	Accepting	0	Pain management	0	Mechanism	0	spouse	0	Co-morbid	0	Histamine	0	Tricyclic	0	Comorbid	0	Prevalence	0	Advantage	0	Peripheral	0	Dopamine	0	Physical examination	0	Referral	0	Attitude	0	Gene-related	0	Calcitonin	0	Beta-blocker	0	church	0	Temporal	0	Barbiturate	0	Lidocaine	0	Spectrum	0	Motivation	0	Menstrual migraine	0	Correlate	0	Outpatient	0	Injury	0	Lab test	0	Efficacy	0	Antihistamine	0	Lactation	0	Secondary headache	0	Trigeminal autonomic cephalalgia	0	Progression	0	Medication overuse	0	Inpatient treatment	0	Intracranial hypertension	0	Botulinum Toxin	0	Serotonin receptor	0	Mindfulness	0	Cranial nerve	0	Episodic migraine	0	Double vision	0	Vestibular migraine	0	surgical	0	Surgical treatment	0	5-HT	0	Ineffective	0	Eye symptom	0	Female hormone	0
0	Headache clinic	0	Assessment	0	Empower	0	Productivity	0	Caregiver	0	Nerve block	0	Accepting	0	Pain management	0	Mechanism	0	spouse	0	Co-morbid	0	Histamine	0	Tricyclic	0	Comorbid	0	Prevalence	0	Advantage	0	Peripheral	0	Dopamine	0	Physical examination	0	Referral	0	Attitude	0	Gene-related	0	Calcitonin	0	Beta-blocker	0	church	0	Temporal	0	Barbiturate	0	Lidocaine	0	Spectrum	0	Motivation	0	Menstrual migraine	0	Correlate	0	Outpatient	0	Injury	0	Lab test	0	Efficacy	0	Antihistamine	0	Lactation	0	Secondary headache	0	Trigeminal autonomic cephalalgia	0	Progression	0	Medication overuse	0	Inpatient treatment	0	Intracranial hypertension	0	Botulinum Toxin	0	Serotonin receptor	0	Mindfulness	0	Cranial nerve	0	Episodic migraine	0	Double vision	0	Vestibular migraine	0	surgical	0	Surgical treatment	0	5-HT	0	Ineffective	0	Eye symptom	0	Female hormone	0
0	Headache clinic	0	Assessment	0	Empower	0	Productivity	0	Caregiver	0	Nerve block	0	Accepting	0	Pain management	0	Mechanism	0	spouse	0	Co-morbid	0	Histamine	0	Tricyclic	0	Comorbid	0	Prevalence	0	Advantage	0	Peripheral	0	Dopamine	0	Physical examination	0	Referral	0	Attitude	0	Gene-related	0	Calcitonin	0	Beta-blocker	0	church	0	Temporal	0	Barbiturate	0	Lidocaine	0	Spectrum	0	Motivation	0	Menstrual migraine	0	Correlate	0	Outpatient	0	Injury	0	Lab test	0	Efficacy	0	Antihistamine	0	Lactation	0	Secondary headache	0	Trigeminal autonomic cephalalgia	0	Progression	0	Medication overuse	0	Inpatient treatment	0	Intracranial hypertension	0	Botulinum Toxin	0	Serotonin receptor	0	Mindfulness	0	Cranial nerve	0	Episodic migraine	0	Double vision	0	Vestibular migraine	0	surgical	0	Surgical treatment	0	5-HT	0	Ineffective	0	Eye symptom	0	Female hormone	0
0	Headache clinic	0	Assessment	0	Empower	0	Productivity	0	Caregiver	0	Nerve block	0	Accepting	0	Pain management	0	Mechanism	0	spouse	0	Co-morbid	0	Histamine	0	Tricyclic	0	Comorbid	0	Prevalence	0	Advantage	0	Peripheral	0	Dopamine	0	Physical examination	0	Referral	0	Attitude	0	Gene-related	0	Calcitonin	0	Beta-blocker	0	church	0	Temporal	0	Barbiturate	0	Lidocaine	0	Spectrum	0	Motivation	0	Menstrual migraine	0	Correlate	0	Outpatient	0	Injury	0	Lab test	0	Efficacy	0	Antihistamine	0	Lactation	0	Secondary headache	0	Trigeminal autonomic cephalalgia	0	Progression	0	Medication overuse	0	Inpatient treatment	0	Intracranial hypertension	0	Botulinum Toxin	0	Serotonin receptor	0	Mindfulness	0	Cranial nerve	0	Episodic migraine	0	Double vision	0	Vestibular migraine	0	surgical	0	Surgical treatment	0	5-HT	0	Ineffective	0	Eye symptom	0	Female hormone	0
0	Headache clinic	0	Assessment	0	Empower	0	Productivity	0	Caregiver	0	Nerve block	0	Accepting	0	Pain management	0	Mechanism	0	spouse	0	Co-morbid	0	Histamine	0	Tricyclic	0	Comorbid	0	Prevalence	0	Advantage	0	Peripheral	0	Dopamine	0	Physical examination	0	Referral	0	Attitude	0	Gene-related	0	Calcitonin	0	Beta-blocker	0	church	0	Temporal	0	Barbiturate	0	Lidocaine	0	Spectrum	0	Motivation	0	Menstrual migraine	0	Correlate	0	Outpatient	0	Injury	0	Lab test	0	Efficacy	0	Antihistamine	0	Lactation	0	Secondary headache	0	Trigeminal autonomic cephalalgia	0	Progression	0	Medication overuse	0	Inpatient treatment	0	Intracranial hypertension	0	Botulinum Toxin	0	Serotonin receptor	0	Mindfulness	0	Cranial nerve	0	Episodic migraine	0	Double vision	0	Vestibular migraine	0	surgical	0	Surgical treatment	0	5-HT	0	Ineffective	0	Eye symptom	0	Female hormone	0
0	Headache clinic	0	Assessment	0	Empower	0	Productivity	0	Caregiver	0	Nerve block	0	Accepting	0	Pain management	0	Mechanism	0	spouse	0	Co-morbid	0	Histamine	0	Tricyclic	0	Comorbid	0	Prevalence	0	Advantage	0	Peripheral	0	Dopamine	0	Physical examination	0	Referral	0	Attitude	0	Gene-related	0	Calcitonin	0	Beta-blocker	0	church	0	Temporal	0	Barbiturate	0	Lidocaine	0	Spectrum	0	Motivation	0	Menstrual migraine	0	Correlate	0	Outpatient	0	Injury	0	Lab test	0	Efficacy	0	Antihistamine	0	Lactation	0	Secondary headache	0	Trigeminal autonomic cephalalgia	0	Progression	0	Medication overuse	0	Inpatient treatment	0	Intracranial hypertension	0	Botulinum Toxin	0	Serotonin receptor	0	Mindfulness	0	Cranial nerve	0	Episodic migraine	0	Double vision	0	Vestibular migraine	0	surgical	0	Surgical treatment	0	5-HT	0	Ineffective	0	Eye symptom	0	Female hormone	0
0	Headache clinic	0	Assessment	0	Empower	0	Productivity	0	Caregiver	0	Nerve block	0	Accepting	0	Pain management	0	Mechanism	0	spouse	0	Co-morbid	0	Histamine	0	Tricyclic	0	Comorbid	0	Prevalence	0	Advantage	0	Peripheral	0	Dopamine	0	Physical examination	0	Referral	0	Attitude	0	Gene-related	0	Calcitonin	0	Beta-blocker	0	church	0	Temporal	0	Barbiturate	0	Lidocaine	0	Spectrum	0	Motivation	0	Menstrual migraine	0	Correlate	0	Outpatient	0	Injury	0	Lab test	0	Efficacy	0	Antihistamine	0	Lactation	0	Secondary headache	0	Trigeminal autonomic cephalalgia	0	Progression	0	Medication overuse	0	Inpatient treatment	0	Intracranial hypertension	0	Botulinum Toxin	0	Serotonin receptor	0	Mindfulness	0	Cranial nerve	0	Episodic migraine	0	Double vision	0	Vestibular migraine	0	surgical	0	Surgical treatment	0	5-HT	0	Ineffective	0	Eye symptom	0	Female hormone	0
0	Headache clinic	0	Assessment	0	Empower	0	Productivity	0	Caregiver	0	Nerve block	0	Accepting	0	Pain management	0	Mechanism	0	spouse	0	Co-morbid	0	Histamine	0	Tricyclic	0	Comorbid	0	Prevalence	0	Advantage	0	Peripheral	0	Dopamine	0	Physical examination	0	Referral	0	Attitude	0	Gene-related	0	Calcitonin	0	Beta-blocker	0	church	0	Temporal	0	Barbiturate	0	Lidocaine	1	Spectrum	0	Motivation	0	Menstrual migraine	0	Correlate	0	Outpatient	0	Injury	0	Lab test	0	Efficacy	0	Antihistamine	0	Lactation	0	Secondary headache	0	Trigeminal autonomic cephalalgia	0	Progression	0	Medication overuse	0	Inpatient treatment	0	Intracranial hypertension	0	Botulinum Toxin	0	Serotonin receptor	0	Mindfulness	0	Cranial nerve	0	Episodic migraine	0	Double vision	0	Vestibular migraine	0	surgical	0	Surgical treatment	0	5-HT	0	Ineffective	0	Eye symptom	0	Female hormone	0
0	Headache clinic	0	Assessment	0	Empower	0	Productivity	0	Caregiver	0	Nerve block	0	Accepting	0	Pain management	0	Mechanism	0	spouse	0	Co-morbid	0	Histamine	0	Tricyclic	0	Comorbid	0	Prevalence	0	Advantage	0	Peripheral	0	Dopamine	0	Physical examination	0	Referral	0	Attitude	0	Gene-related	0	Calcitonin	0	Beta-blocker	0	church	0	Temporal	0	Barbiturate	0	Lidocaine	0	Spectrum	0	Motivation	0	Menstrual migraine	0	Correlate	0	Outpatient	0	Injury	0	Lab test	0	Efficacy	0	Antihistamine	0	Lactation	0	Secondary headache	0	Trigeminal autonomic cephalalgia	0	Progression	0	Medication overuse	0	Inpatient treatment	0	Intracranial hypertension	0	Botulinum Toxin	0	Serotonin receptor	0	Mindfulness	0	Cranial nerve	0	Episodic migraine	0	Double vision	0	Vestibular migraine	0	surgical	0	Surgical treatment	0	5-HT	0	Ineffective	0	Eye symptom	0	Female hormone	0
0	Headache clinic	0	Assessment	0	Empower	0	Productivity	0	Caregiver	1	Nerve block	0	Accepting	0	Pain management	0	Mechanism	0	spouse	0	Co-morbid	0	Histamine	0	Tricyclic	0	Comorbid	0	Prevalence	0	Advantage	0	Peripheral	0	Dopamine	0	Physical examination	0	Referral	0	Attitude	0	Gene-related	0	Calcitonin	0	Beta-blocker	0	church	0	Temporal	0	Barbiturate	0	Lidocaine	0	Spectrum	0	Motivation	0	Menstrual migraine	0	Correlate	0	Outpatient	0	Injury	0	Lab test	0	Efficacy	0	Antihistamine	0	Lactation	0	Secondary headache	0	Trigeminal autonomic cephalalgia	0	Progression	0	Medication overuse	0	Inpatient treatment	0	Intracranial hypertension	0	Botulinum Toxin	0	Serotonin receptor	0	Mindfulness	0	Cranial nerve	0	Episodic migraine	0	Double vision	0	Vestibular migraine	0	surgical	0	Surgical treatment	0	5-HT	0	Ineffective	0	Eye symptom	0	Female hormone	0
0	Headache clinic	0	Assessment	0	Empower	0	Productivity	0	Caregiver	0	Nerve block	0	Accepting	0	Pain management	0	Mechanism	0	spouse	0	Co-morbid	0	Histamine	0	Tricyclic	0	Comorbid	0	Prevalence	0	Advantage	0	Peripheral	0	Dopamine	0	Physical examination	0	Referral	0	Attitude	0	Gene-related	0	Calcitonin	0	Beta-blocker	0	church	0	Temporal	0	Barbiturate	0	Lidocaine	0	Spectrum	0	Motivation	0	Menstrual migraine	0	Correlate	0	Outpatient	0	Injury	0	Lab test	0	Efficacy	0	Antihistamine	0	Lactation	0	Secondary headache	0	Trigeminal autonomic cephalalgia	0	Progression	0	Medication overuse	0	Inpatient treatment	0	Intracranial hypertension	0	Botulinum Toxin	0	Serotonin receptor	0	Mindfulness	0	Cranial nerve	0	Episodic migraine	0	Double vision	0	Vestibular migraine	0	surgical	1	Surgical treatment	1	5-HT	0	Ineffective	0	Eye symptom	0	Female hormone	0
0	Headache clinic	0	Assessment	0	Empower	0	Productivity	0	Caregiver	0	Nerve block	0	Accepting	0	Pain management	0	Mechanism	0	spouse	0	Co-morbid	0	Histamine	0	Tricyclic	0	Comorbid	0	Prevalence	0	Advantage	0	Peripheral	0	Dopamine	0	Physical examination	0	Referral	0	Attitude	0	Gene-related	0	Calcitonin	0	Beta-blocker	0	church	0	Temporal	0	Barbiturate	0	Lidocaine	0	Spectrum	0	Motivation	0	Menstrual migraine	0	Correlate	0	Outpatient	0	Injury	0	Lab test	0	Efficacy	0	Antihistamine	0	Lactation	0	Secondary headache	0	Trigeminal autonomic cephalalgia	0	Progression	0	Medication overuse	0	Inpatient treatment	0	Intracranial hypertension	0	Botulinum Toxin	0	Serotonin receptor	0	Mindfulness	0	Cranial nerve	0	Episodic migraine	0	Double vision	0	Vestibular migraine	0	surgical	0	Surgical treatment	0	5-HT	0	Ineffective	0	Eye symptom	0	Female hormone	0
0	Headache clinic	0	Assessment	0	Empower	0	Productivity	0	Caregiver	0	Nerve block	0	Accepting	0	Pain management	0	Mechanism	0	spouse	0	Co-morbid	0	Histamine	0	Tricyclic	0	Comorbid	0	Prevalence	0	Advantage	0	Peripheral	0	Dopamine	0	Physical examination	0	Referral	0	Attitude	0	Gene-related	0	Calcitonin	0	Beta-blocker	0	church	0	Temporal	0	Barbiturate	0	Lidocaine	0	Spectrum	0	Motivation	0	Menstrual migraine	0	Correlate	0	Outpatient	0	Injury	0	Lab test	0	Efficacy	0	Antihistamine	0	Lactation	0	Secondary headache	0	Trigeminal autonomic cephalalgia	0	Progression	0	Medication overuse	0	Inpatient treatment	0	Intracranial hypertension	0	Botulinum Toxin	0	Serotonin receptor	0	Mindfulness	0	Cranial nerve	0	Episodic migraine	0	Double vision	0	Vestibular migraine	0	surgical	0	Surgical treatment	0	5-HT	0	Ineffective	0	Eye symptom	0	Female hormone	0
0	Headache clinic	0	Assessment	0	Empower	0	Productivity	0	Caregiver	0	Nerve block	0	Accepting	0	Pain management	0	Mechanism	0	spouse	0	Co-morbid	0	Histamine	0	Tricyclic	0	Comorbid	0	Prevalence	0	Advantage	0	Peripheral	0	Dopamine	0	Physical examination	0	Referral	0	Attitude	0	Gene-related	0	Calcitonin	0	Beta-blocker	1	church	0	Temporal	0	Barbiturate	0	Lidocaine	0	Spectrum	0	Motivation	0	Menstrual migraine	0	Correlate	0	Outpatient	0	Injury	0	Lab test	0	Efficacy	0	Antihistamine	0	Lactation	0	Secondary headache	0	Trigeminal autonomic cephalalgia	0	Progression	0	Medication overuse	0	Inpatient treatment	0	Intracranial hypertension	0	Botulinum Toxin	0	Serotonin receptor	0	Mindfulness	0	Cranial nerve	0	Episodic migraine	0	Double vision	0	Vestibular migraine	0	surgical	0	Surgical treatment	0	5-HT	0	Ineffective	0	Eye symptom	0	Female hormone	0
0	Headache clinic	0	Assessment	0	Empower	0	Productivity	0	Caregiver	0	Nerve block	0	Accepting	0	Pain management	0	Mechanism	0	spouse	0	Co-morbid	0	Histamine	0	Tricyclic	0	Comorbid	0	Prevalence	0	Advantage	0	Peripheral	0	Dopamine	0	Physical examination	0	Referral	0	Attitude	0	Gene-related	0	Calcitonin	0	Beta-blocker	0	church	0	Temporal	0	Barbiturate	0	Lidocaine	0	Spectrum	0	Motivation	0	Menstrual migraine	0	Correlate	0	Outpatient	0	Injury	0	Lab test	0	Efficacy	0	Antihistamine	0	Lactation	0	Secondary headache	0	Trigeminal autonomic cephalalgia	0	Progression	0	Medication overuse	0	Inpatient treatment	0	Intracranial hypertension	0	Botulinum Toxin	0	Serotonin receptor	0	Mindfulness	0	Cranial nerve	0	Episodic migraine	0	Double vision	0	Vestibular migraine	0	surgical	0	Surgical treatment	0	5-HT	0	Ineffective	0	Eye symptom	0	Female hormone	0
0	Headache clinic	0	Assessment	0	Empower	0	Productivity	0	Caregiver	0	Nerve block	0	Accepting	0	Pain management	0	Mechanism	0	spouse	0	Co-morbid	0	Histamine	0	Tricyclic	0	Comorbid	0	Prevalence	0	Advantage	0	Peripheral	0	Dopamine	0	Physical examination	0	Referral	0	Attitude	0	Gene-related	0	Calcitonin	0	Beta-blocker	0	church	0	Temporal	0	Barbiturate	0	Lidocaine	0	Spectrum	0	Motivation	0	Menstrual migraine	0	Correlate	0	Outpatient	0	Injury	0	Lab test	0	Efficacy	0	Antihistamine	0	Lactation	0	Secondary headache	0	Trigeminal autonomic cephalalgia	0	Progression	0	Medication overuse	0	Inpatient treatment	0	Intracranial hypertension	0	Botulinum Toxin	0	Serotonin receptor	0	Mindfulness	0	Cranial nerve	0	Episodic migraine	0	Double vision	0	Vestibular migraine	0	surgical	0	Surgical treatment	0	5-HT	0	Ineffective	0	Eye symptom	0	Female hormone	0
0	Headache clinic	0	Assessment	0	Empower	0	Productivity	0	Caregiver	0	Nerve block	0	Accepting	0	Pain management	0	Mechanism	0	spouse	0	Co-morbid	0	Histamine	0	Tricyclic	0	Comorbid	0	Prevalence	0	Advantage	0	Peripheral	0	Dopamine	0	Physical examination	0	Referral	0	Attitude	0	Gene-related	0	Calcitonin	0	Beta-blocker	0	church	0	Temporal	0	Barbiturate	0	Lidocaine	0	Spectrum	0	Motivation	0	Menstrual migraine	0	Correlate	0	Outpatient	0	Injury	0	Lab test	0	Efficacy	0	Antihistamine	0	Lactation	0	Secondary headache	0	Trigeminal autonomic cephalalgia	0	Progression	0	Medication overuse	0	Inpatient treatment	0	Intracranial hypertension	0	Botulinum Toxin	0	Serotonin receptor	0	Mindfulness	0	Cranial nerve	0	Episodic migraine	0	Double vision	0	Vestibular migraine	0	surgical	0	Surgical treatment	0	5-HT	0	Ineffective	0	Eye symptom	0	Female hormone	0
0	Headache clinic	0	Assessment	0	Empower	0	Productivity	1	Caregiver	1	Nerve block	0	Accepting	0	Pain management	0	Mechanism	0	spouse	0	Co-morbid	0	Histamine	0	Tricyclic	0	Comorbid	0	Prevalence	0	Advantage	0	Peripheral	0	Dopamine	0	Physical examination	0	Referral	0	Attitude	0	Gene-related	1	Calcitonin	1	Beta-blocker	0	church	0	Temporal	0	Barbiturate	0	Lidocaine	0	Spectrum	0	Motivation	0	Menstrual migraine	0	Correlate	0	Outpatient	0	Injury	0	Lab test	0	Efficacy	0	Antihistamine	0	Lactation	0	Secondary headache	0	Trigeminal autonomic cephalalgia	0	Progression	0	Medication overuse	0	Inpatient treatment	0	Intracranial hypertension	0	Botulinum Toxin	0	Serotonin receptor	0	Mindfulness	0	Cranial nerve	0	Episodic migraine	0	Double vision	0	Vestibular migraine	0	surgical	0	Surgical treatment	0	5-HT	0	Ineffective	0	Eye symptom	0	Female hormone	0
0	Headache clinic	0	Assessment	0	Empower	0	Productivity	0	Caregiver	0	Nerve block	0	Accepting	0	Pain management	0	Mechanism	0	spouse	0	Co-morbid	0	Histamine	0	Tricyclic	0	Comorbid	0	Prevalence	0	Advantage	0	Peripheral	0	Dopamine	0	Physical examination	0	Referral	0	Attitude	0	Gene-related	0	Calcitonin	0	Beta-blocker	0	church	0	Temporal	0	Barbiturate	0	Lidocaine	0	Spectrum	0	Motivation	0	Menstrual migraine	0	Correlate	0	Outpatient	0	Injury	0	Lab test	0	Efficacy	0	Antihistamine	0	Lactation	0	Secondary headache	0	Trigeminal autonomic cephalalgia	0	Progression	0	Medication overuse	0	Inpatient treatment	0	Intracranial hypertension	0	Botulinum Toxin	0	Serotonin receptor	0	Mindfulness	0	Cranial nerve	0	Episodic migraine	0	Double vision	0	Vestibular migraine	0	surgical	0	Surgical treatment	0	5-HT	0	Ineffective	0	Eye symptom	0	Female hormone	0
0	Headache clinic	0	Assessment	0	Empower	0	Productivity	0	Caregiver	0	Nerve block	0	Accepting	0	Pain management	0	Mechanism	0	spouse	0	Co-morbid	0	Histamine	0	Tricyclic	0	Comorbid	0	Prevalence	0	Advantage	0	Peripheral	0	Dopamine	0	Physical examination	0	Referral	0	Attitude	0	Gene-related	0	Calcitonin	0	Beta-blocker	0	church	0	Temporal	0	Barbiturate	0	Lidocaine	0	Spectrum	0	Motivation	0	Menstrual migraine	0	Correlate	0	Outpatient	0	Injury	0	Lab test	0	Efficacy	0	Antihistamine	0	Lactation	0	Secondary headache	0	Trigeminal autonomic cephalalgia	0	Progression	0	Medication overuse	0	Inpatient treatment	0	Intracranial hypertension	0	Botulinum Toxin	0	Serotonin receptor	0	Mindfulness	0	Cranial nerve	0	Episodic migraine	0	Double vision	0	Vestibular migraine	0	surgical	0	Surgical treatment	0	5-HT	0	Ineffective	0	Eye symptom	0	Female hormone	0
0	Headache clinic	0	Assessment	0	Empower	0	Productivity	0	Caregiver	0	Nerve block	0	Accepting	0	Pain management	0	Mechanism	0	spouse	0	Co-morbid	0	Histamine	0	Tricyclic	0	Comorbid	0	Prevalence	0	Advantage	0	Peripheral	0	Dopamine	0	Physical examination	0	Referral	0	Attitude	0	Gene-related	0	Calcitonin	0	Beta-blocker	0	church	0	Temporal	0	Barbiturate	0	Lidocaine	0	Spectrum	0	Motivation	0	Menstrual migraine	0	Correlate	0	Outpatient	0	Injury	0	Lab test	0	Efficacy	0	Antihistamine	0	Lactation	0	Secondary headache	0	Trigeminal autonomic cephalalgia	0	Progression	0	Medication overuse	0	Inpatient treatment	0	Intracranial hypertension	0	Botulinum Toxin	0	Serotonin receptor	0	Mindfulness	0	Cranial nerve	0	Episodic migraine	0	Double vision	0	Vestibular migraine	0	surgical	0	Surgical treatment	0	5-HT	0	Ineffective	0	Eye symptom	0	Female hormone	0
0	Headache clinic	0	Assessment	0	Empower	0	Productivity	0	Caregiver	0	Nerve block	0	Accepting	0	Pain management	0	Mechanism	0	spouse	0	Co-morbid	0	Histamine	0	Tricyclic	0	Comorbid	0	Prevalence	0	Advantage	0	Peripheral	0	Dopamine	0	Physical examination	0	Referral	0	Attitude	0	Gene-related	0	Calcitonin	0	Beta-blocker	0	church	0	Temporal	0	Barbiturate	0	Lidocaine	0	Spectrum	0	Motivation	0	Menstrual migraine	0	Correlate	0	Outpatient	0	Injury	0	Lab test	0	Efficacy	0	Antihistamine	0	Lactation	0	Secondary headache	0	Trigeminal autonomic cephalalgia	0	Progression	0	Medication overuse	0	Inpatient treatment	0	Intracranial hypertension	0	Botulinum Toxin	0	Serotonin receptor	0	Mindfulness	0	Cranial nerve	0	Episodic migraine	0	Double vision	0	Vestibular migraine	2	surgical	0	Surgical treatment	0	5-HT	0	Ineffective	0	Eye symptom	0	Female hormone	0
0	Headache clinic	0	Assessment	0	Empower	0	Productivity	0	Caregiver	0	Nerve block	0	Accepting	0	Pain management	0	Mechanism	0	spouse	0	Co-morbid	0	Histamine	0	Tricyclic	0	Comorbid	0	Prevalence	0	Advantage	0	Peripheral	0	Dopamine	0	Physical examination	0	Referral	0	Attitude	0	Gene-related	0	Calcitonin	0	Beta-blocker	0	church	0	Temporal	0	Barbiturate	0	Lidocaine	0	Spectrum	0	Motivation	0	Menstrual migraine	0	Correlate	0	Outpatient	0	Injury	0	Lab test	0	Efficacy	0	Antihistamine	0	Lactation	0	Secondary headache	0	Trigeminal autonomic cephalalgia	0	Progression	0	Medication overuse	0	Inpatient treatment	0	Intracranial hypertension	0	Botulinum Toxin	0	Serotonin receptor	0	Mindfulness	0	Cranial nerve	0	Episodic migraine	0	Double vision	0	Vestibular migraine	0	surgical	0	Surgical treatment	0	5-HT	0	Ineffective	0	Eye symptom	0	Female hormone	0
0	Headache clinic	0	Assessment	0	Empower	0	Productivity	0	Caregiver	0	Nerve block	0	Accepting	0	Pain management	0	Mechanism	0	spouse	0	Co-morbid	0	Histamine	0	Tricyclic	0	Comorbid	0	Prevalence	0	Advantage	0	Peripheral	0	Dopamine	0	Physical examination	0	Referral	0	Attitude	0	Gene-related	0	Calcitonin	0	Beta-blocker	1	church	0	Temporal	0	Barbiturate	0	Lidocaine	0	Spectrum	0	Motivation	0	Menstrual migraine	0	Correlate	0	Outpatient	0	Injury	0	Lab test	0	Efficacy	0	Antihistamine	0	Lactation	0	Secondary headache	0	Trigeminal autonomic cephalalgia	0	Progression	0	Medication overuse	0	Inpatient treatment	0	Intracranial hypertension	0	Botulinum Toxin	0	Serotonin receptor	0	Mindfulness	0	Cranial nerve	0	Episodic migraine	0	Double vision	1	Vestibular migraine	0	surgical	0	Surgical treatment	0	5-HT	0	Ineffective	0	Eye symptom	0	Female hormone	0
0	Headache clinic	0	Assessment	0	Empower	0	Productivity	1	Caregiver	0	Nerve block	0	Accepting	0	Pain management	0	Mechanism	0	spouse	0	Co-morbid	0	Histamine	0	Tricyclic	0	Comorbid	0	Prevalence	0	Advantage	0	Peripheral	0	Dopamine	0	Physical examination	0	Referral	0	Attitude	0	Gene-related	0	Calcitonin	0	Beta-blocker	0	church	0	Temporal	0	Barbiturate	0	Lidocaine	0	Spectrum	0	Motivation	0	Menstrual migraine	0	Correlate	0	Outpatient	0	Injury	0	Lab test	0	Efficacy	0	Antihistamine	0	Lactation	0	Secondary headache	0	Trigeminal autonomic cephalalgia	0	Progression	0	Medication overuse	0	Inpatient treatment	0	Intracranial hypertension	0	Botulinum Toxin	0	Serotonin receptor	0	Mindfulness	0	Cranial nerve	2	Episodic migraine	2	Double vision	0	Vestibular migraine	0	surgical	0	Surgical treatment	0	5-HT	0	Ineffective	0	Eye symptom	0	Female hormone	0
0	Headache clinic	0	Assessment	0	Empower	0	Productivity	0	Caregiver	0	Nerve block	0	Accepting	0	Pain management	0	Mechanism	0	spouse	0	Co-morbid	0	Histamine	0	Tricyclic	0	Comorbid	0	Prevalence	0	Advantage	0	Peripheral	0	Dopamine	0	Physical examination	0	Referral	0	Attitude	0	Gene-related	0	Calcitonin	0	Beta-blocker	0	church	0	Temporal	0	Barbiturate	0	Lidocaine	0	Spectrum	0	Motivation	0	Menstrual migraine	0	Correlate	0	Outpatient	0	Injury	0	Lab test	0	Efficacy	0	Antihistamine	0	Lactation	0	Secondary headache	0	Trigeminal autonomic cephalalgia	0	Progression	0	Medication overuse	0	Inpatient treatment	0	Intracranial hypertension	0	Botulinum Toxin	0	Serotonin receptor	0	Mindfulness	0	Cranial nerve	0	Episodic migraine	0	Double vision	0	Vestibular migraine	0	surgical	0	Surgical treatment	0	5-HT	0	Ineffective	0	Eye symptom	0	Female hormone	0
0	Headache clinic	0	Assessment	0	Empower	0	Productivity	0	Caregiver	0	Nerve block	0	Accepting	0	Pain management	0	Mechanism	0	spouse	0	Co-morbid	0	Histamine	0	Tricyclic	0	Comorbid	0	Prevalence	0	Advantage	0	Peripheral	0	Dopamine	0	Physical examination	0	Referral	0	Attitude	0	Gene-related	0	Calcitonin	0	Beta-blocker	0	church	0	Temporal	0	Barbiturate	0	Lidocaine	0	Spectrum	0	Motivation	0	Menstrual migraine	0	Correlate	0	Outpatient	0	Injury	0	Lab test	0	Efficacy	0	Antihistamine	0	Lactation	0	Secondary headache	0	Trigeminal autonomic cephalalgia	0	Progression	0	Medication overuse	0	Inpatient treatment	0	Intracranial hypertension	0	Botulinum Toxin	0	Serotonin receptor	0	Mindfulness	0	Cranial nerve	0	Episodic migraine	0	Double vision	0	Vestibular migraine	0	surgical	0	Surgical treatment	0	5-HT	0	Ineffective	0	Eye symptom	0	Female hormone	0
0	Headache clinic	0	Assessment	0	Empower	1	Productivity	0	Caregiver	0	Nerve block	0	Accepting	0	Pain management	0	Mechanism	0	spouse	0	Co-morbid	0	Histamine	0	Tricyclic	0	Comorbid	0	Prevalence	0	Advantage	0	Peripheral	0	Dopamine	0	Physical examination	0	Referral	0	Attitude	1	Gene-related	0	Calcitonin	0	Beta-blocker	0	church	0	Temporal	0	Barbiturate	0	Lidocaine	0	Spectrum	0	Motivation	0	Menstrual migraine	0	Correlate	0	Outpatient	0	Injury	0	Lab test	0	Efficacy	0	Antihistamine	0	Lactation	0	Secondary headache	0	Trigeminal autonomic cephalalgia	0	Progression	0	Medication overuse	0	Inpatient treatment	0	Intracranial hypertension	0	Botulinum Toxin	0	Serotonin receptor	0	Mindfulness	0	Cranial nerve	0	Episodic migraine	0	Double vision	0	Vestibular migraine	0	surgical	0	Surgical treatment	0	5-HT	0	Ineffective	0	Eye symptom	0	Female hormone	0
0	Headache clinic	0	Assessment	0	Empower	0	Productivity	0	Caregiver	0	Nerve block	0	Accepting	0	Pain management	0	Mechanism	0	spouse	0	Co-morbid	0	Histamine	0	Tricyclic	0	Comorbid	0	Prevalence	0	Advantage	0	Peripheral	0	Dopamine	0	Physical examination	0	Referral	0	Attitude	0	Gene-related	0	Calcitonin	0	Beta-blocker	0	church	0	Temporal	0	Barbiturate	0	Lidocaine	0	Spectrum	0	Motivation	0	Menstrual migraine	0	Correlate	0	Outpatient	0	Injury	0	Lab test	0	Efficacy	0	Antihistamine	0	Lactation	0	Secondary headache	0	Trigeminal autonomic cephalalgia	0	Progression	0	Medication overuse	0	Inpatient treatment	0	Intracranial hypertension	0	Botulinum Toxin	0	Serotonin receptor	0	Mindfulness	1	Cranial nerve	0	Episodic migraine	0	Double vision	0	Vestibular migraine	0	surgical	0	Surgical treatment	0	5-HT	0	Ineffective	0	Eye symptom	0	Female hormone	0
0	Headache clinic	0	Assessment	0	Empower	0	Productivity	0	Caregiver	0	Nerve block	0	Accepting	0	Pain management	0	Mechanism	0	spouse	0	Co-morbid	0	Histamine	0	Tricyclic	0	Comorbid	0	Prevalence	0	Advantage	0	Peripheral	0	Dopamine	0	Physical examination	0	Referral	0	Attitude	0	Gene-related	0	Calcitonin	0	Beta-blocker	0	church	0	Temporal	0	Barbiturate	0	Lidocaine	0	Spectrum	0	Motivation	0	Menstrual migraine	0	Correlate	0	Outpatient	0	Injury	0	Lab test	0	Efficacy	0	Antihistamine	0	Lactation	0	Secondary headache	0	Trigeminal autonomic cephalalgia	0	Progression	0	Medication overuse	0	Inpatient treatment	0	Intracranial hypertension	0	Botulinum Toxin	0	Serotonin receptor	1	Mindfulness	0	Cranial nerve	0	Episodic migraine	0	Double vision	0	Vestibular migraine	0	surgical	0	Surgical treatment	0	5-HT	0	Ineffective	0	Eye symptom	0	Female hormone	0
0	Headache clinic	0	Assessment	0	Empower	0	Productivity	0	Caregiver	0	Nerve block	0	Accepting	0	Pain management	0	Mechanism	0	spouse	0	Co-morbid	0	Histamine	0	Tricyclic	0	Comorbid	0	Prevalence	0	Advantage	0	Peripheral	0	Dopamine	0	Physical examination	0	Referral	0	Attitude	0	Gene-related	0	Calcitonin	0	Beta-blocker	0	church	0	Temporal	0	Barbiturate	0	Lidocaine	0	Spectrum	0	Motivation	0	Menstrual migraine	0	Correlate	0	Outpatient	0	Injury	0	Lab test	0	Efficacy	0	Antihistamine	0	Lactation	0	Secondary headache	0	Trigeminal autonomic cephalalgia	0	Progression	0	Medication overuse	0	Inpatient treatment	0	Intracranial hypertension	0	Botulinum Toxin	0	Serotonin receptor	0	Mindfulness	0	Cranial nerve	0	Episodic migraine	0	Double vision	0	Vestibular migraine	0	surgical	0	Surgical treatment	0	5-HT	0	Ineffective	0	Eye symptom	0	Female hormone	0
0	Headache clinic	0	Assessment	0	Empower	0	Productivity	0	Caregiver	0	Nerve block	0	Accepting	0	Pain management	0	Mechanism	0	spouse	0	Co-morbid	0	Histamine	0	Tricyclic	0	Comorbid	0	Prevalence	0	Advantage	0	Peripheral	0	Dopamine	0	Physical examination	0	Referral	0	Attitude	0	Gene-related	0	Calcitonin	0	Beta-blocker	0	church	0	Temporal	0	Barbiturate	0	Lidocaine	0	Spectrum	0	Motivation	0	Menstrual migraine	0	Correlate	0	Outpatient	0	Injury	0	Lab test	0	Efficacy	0	Antihistamine	0	Lactation	0	Secondary headache	0	Trigeminal autonomic cephalalgia	0	Progression	0	Medication overuse	0	Inpatient treatment	0	Intracranial hypertension	0	Botulinum Toxin	0	Serotonin receptor	0	Mindfulness	0	Cranial nerve	0	Episodic migraine	0	Double vision	0	Vestibular migraine	0	surgical	0	Surgical treatment	0	5-HT	0	Ineffective	0	Eye symptom	0	Female hormone	0
0	Headache clinic	0	Assessment	0	Empower	0	Productivity	0	Caregiver	0	Nerve block	0	Accepting	0	Pain management	1	Mechanism	0	spouse	0	Co-morbid	0	Histamine	0	Tricyclic	0	Comorbid	0	Prevalence	0	Advantage	0	Peripheral	0	Dopamine	0	Physical examination	0	Referral	0	Attitude	0	Gene-related	0	Calcitonin	0	Beta-blocker	0	church	0	Temporal	0	Barbiturate	0	Lidocaine	0	Spectrum	0	Motivation	0	Menstrual migraine	0	Correlate	0	Outpatient	0	Injury	0	Lab test	0	Efficacy	0	Antihistamine	0	Lactation	0	Secondary headache	0	Trigeminal autonomic cephalalgia	0	Progression	0	Medication overuse	0	Inpatient treatment	0	Intracranial hypertension	0	Botulinum Toxin	0	Serotonin receptor	0	Mindfulness	0	Cranial nerve	0	Episodic migraine	0	Double vision	0	Vestibular migraine	0	surgical	0	Surgical treatment	0	5-HT	0	Ineffective	0	Eye symptom	0	Female hormone	0
0	Headache clinic	0	Assessment	0	Empower	0	Productivity	0	Caregiver	0	Nerve block	0	Accepting	0	Pain management	0	Mechanism	0	spouse	0	Co-morbid	0	Histamine	0	Tricyclic	0	Comorbid	0	Prevalence	0	Advantage	0	Peripheral	0	Dopamine	0	Physical examination	0	Referral	1	Attitude	0	Gene-related	0	Calcitonin	0	Beta-blocker	0	church	0	Temporal	0	Barbiturate	0	Lidocaine	0	Spectrum	0	Motivation	0	Menstrual migraine	0	Correlate	0	Outpatient	0	Injury	0	Lab test	0	Efficacy	0	Antihistamine	0	Lactation	0	Secondary headache	0	Trigeminal autonomic cephalalgia	0	Progression	0	Medication overuse	0	Inpatient treatment	0	Intracranial hypertension	0	Botulinum Toxin	0	Serotonin receptor	0	Mindfulness	0	Cranial nerve	0	Episodic migraine	0	Double vision	0	Vestibular migraine	0	surgical	0	Surgical treatment	0	5-HT	0	Ineffective	0	Eye symptom	0	Female hormone	0
0	Headache clinic	0	Assessment	0	Empower	0	Productivity	0	Caregiver	0	Nerve block	2	Accepting	2	Pain management	1	Mechanism	1	spouse	1	Co-morbid	0	Histamine	0	Tricyclic	0	Comorbid	0	Prevalence	0	Advantage	0	Peripheral	0	Dopamine	0	Physical examination	0	Referral	0	Attitude	0	Gene-related	0	Calcitonin	0	Beta-blocker	0	church	0	Temporal	1	Barbiturate	1	Lidocaine	1	Spectrum	1	Motivation	1	Menstrual migraine	1	Correlate	0	Outpatient	0	Injury	0	Lab test	0	Efficacy	0	Antihistamine	0	Lactation	0	Secondary headache	0	Trigeminal autonomic cephalalgia	0	Progression	0	Medication overuse	0	Inpatient treatment	0	Intracranial hypertension	0	Botulinum Toxin	0	Serotonin receptor	0	Mindfulness	0	Cranial nerve	0	Episodic migraine	0	Double vision	0	Vestibular migraine	0	surgical	0	Surgical treatment	0	5-HT	0	Ineffective	0	Eye symptom	0	Female hormone	0
0	Headache clinic	0	Assessment	0	Empower	0	Productivity	0	Caregiver	0	Nerve block	0	Accepting	0	Pain management	0	Mechanism	0	spouse	0	Co-morbid	0	Histamine	0	Tricyclic	0	Comorbid	0	Prevalence	0	Advantage	0	Peripheral	0	Dopamine	0	Physical examination	0	Referral	0	Attitude	0	Gene-related	0	Calcitonin	0	Beta-blocker	0	church	0	Temporal	0	Barbiturate	0	Lidocaine	0	Spectrum	0	Motivation	0	Menstrual migraine	0	Correlate	0	Outpatient	0	Injury	0	Lab test	0	Efficacy	0	Antihistamine	0	Lactation	0	Secondary headache	0	Trigeminal autonomic cephalalgia	0	Progression	0	Medication overuse	0	Inpatient treatment	0	Intracranial hypertension	0	Botulinum Toxin	0	Serotonin receptor	0	Mindfulness	0	Cranial nerve	0	Episodic migraine	0	Double vision	0	Vestibular migraine	0	surgical	0	Surgical treatment	0	5-HT	0	Ineffective	0	Eye symptom	0	Female hormone	0
0	Headache clinic	0	Assessment	0	Empower	0	Productivity	0	Caregiver	0	Nerve block	0	Accepting	0	Pain management	0	Mechanism	0	spouse	0	Co-morbid	0	Histamine	0	Tricyclic	0	Comorbid	0	Prevalence	0	Advantage	0	Peripheral	0	Dopamine	0	Physical examination	0	Referral	0	Attitude	0	Gene-related	0	Calcitonin	0	Beta-blocker	0	church	0	Temporal	0	Barbiturate	0	Lidocaine	0	Spectrum	0	Motivation	0	Menstrual migraine	0	Correlate	0	Outpatient	0	Injury	0	Lab test	0	Efficacy	0	Antihistamine	0	Lactation	0	Secondary headache	0	Trigeminal autonomic cephalalgia	0	Progression	0	Medication overuse	0	Inpatient treatment	0	Intracranial hypertension	0	Botulinum Toxin	0	Serotonin receptor	0	Mindfulness	0	Cranial nerve	0	Episodic migraine	0	Double vision	0	Vestibular migraine	0	surgical	0	Surgical treatment	0	5-HT	0	Ineffective	0	Eye symptom	0	Female hormone	0
0	Headache clinic	0	Assessment	0	Empower	0	Productivity	0	Caregiver	0	Nerve block	0	Accepting	0	Pain management	0	Mechanism	0	spouse	0	Co-morbid	0	Histamine	0	Tricyclic	0	Comorbid	0	Prevalence	0	Advantage	0	Peripheral	0	Dopamine	0	Physical examination	1	Referral	0	Attitude	0	Gene-related	0	Calcitonin	0	Beta-blocker	0	church	0	Temporal	0	Barbiturate	0	Lidocaine	0	Spectrum	0	Motivation	0	Menstrual migraine	0	Correlate	0	Outpatient	0	Injury	0	Lab test	0	Efficacy	0	Antihistamine	0	Lactation	0	Secondary headache	0	Trigeminal autonomic cephalalgia	0	Progression	0	Medication overuse	0	Inpatient treatment	0	Intracranial hypertension	0	Botulinum Toxin	0	Serotonin receptor	0	Mindfulness	0	Cranial nerve	0	Episodic migraine	0	Double vision	0	Vestibular migraine	0	surgical	0	Surgical treatment	0	5-HT	0	Ineffective	0	Eye symptom	0	Female hormone	0
0	Headache clinic	0	Assessment	1	Empower	0	Productivity	0	Caregiver	0	Nerve block	0	Accepting	0	Pain management	0	Mechanism	0	spouse	0	Co-morbid	0	Histamine	0	Tricyclic	0	Comorbid	0	Prevalence	0	Advantage	0	Peripheral	0	Dopamine	0	Physical examination	0	Referral	0	Attitude	0	Gene-related	0	Calcitonin	0	Beta-blocker	0	church	0	Temporal	0	Barbiturate	0	Lidocaine	0	Spectrum	0	Motivation	0	Menstrual migraine	0	Correlate	0	Outpatient	0	Injury	0	Lab test	0	Efficacy	0	Antihistamine	0	Lactation	0	Secondary headache	0	Trigeminal autonomic cephalalgia	0	Progression	0	Medication overuse	0	Inpatient treatment	0	Intracranial hypertension	0	Botulinum Toxin	0	Serotonin receptor	0	Mindfulness	0	Cranial nerve	0	Episodic migraine	0	Double vision	0	Vestibular migraine	0	surgical	0	Surgical treatment	0	5-HT	0	Ineffective	0	Eye symptom	0	Female hormone	0
0	Headache clinic	0	Assessment	0	Empower	0	Productivity	0	Caregiver	0	Nerve block	0	Accepting	0	Pain management	0	Mechanism	0	spouse	0	Co-morbid	0	Histamine	0	Tricyclic	0	Comorbid	0	Prevalence	0	Advantage	0	Peripheral	0	Dopamine	1	Physical examination	0	Referral	0	Attitude	0	Gene-related	0	Calcitonin	0	Beta-blocker	0	church	0	Temporal	1	Barbiturate	0	Lidocaine	0	Spectrum	0	Motivation	0	Menstrual migraine	0	Correlate	0	Outpatient	0	Injury	0	Lab test	0	Efficacy	0	Antihistamine	0	Lactation	0	Secondary headache	0	Trigeminal autonomic cephalalgia	0	Progression	0	Medication overuse	0	Inpatient treatment	0	Intracranial hypertension	0	Botulinum Toxin	1	Serotonin receptor	0	Mindfulness	0	Cranial nerve	0	Episodic migraine	0	Double vision	0	Vestibular migraine	0	surgical	0	Surgical treatment	0	5-HT	0	Ineffective	0	Eye symptom	0	Female hormone	0
0	Headache clinic	0	Assessment	0	Empower	0	Productivity	0	Caregiver	0	Nerve block	0	Accepting	0	Pain management	0	Mechanism	0	spouse	0	Co-morbid	0	Histamine	0	Tricyclic	0	Comorbid	0	Prevalence	0	Advantage	0	Peripheral	0	Dopamine	0	Physical examination	0	Referral	0	Attitude	0	Gene-related	0	Calcitonin	0	Beta-blocker	0	church	0	Temporal	0	Barbiturate	0	Lidocaine	0	Spectrum	0	Motivation	0	Menstrual migraine	0	Correlate	0	Outpatient	0	Injury	0	Lab test	0	Efficacy	0	Antihistamine	0	Lactation	0	Secondary headache	0	Trigeminal autonomic cephalalgia	0	Progression	0	Medication overuse	0	Inpatient treatment	0	Intracranial hypertension	0	Botulinum Toxin	0	Serotonin receptor	0	Mindfulness	0	Cranial nerve	0	Episodic migraine	0	Double vision	0	Vestibular migraine	0	surgical	0	Surgical treatment	0	5-HT	0	Ineffective	0	Eye symptom	0	Female hormone	0
0	Headache clinic	0	Assessment	0	Empower	0	Productivity	0	Caregiver	0	Nerve block	0	Accepting	0	Pain management	0	Mechanism	0	spouse	0	Co-morbid	0	Histamine	0	Tricyclic	0	Comorbid	0	Prevalence	0	Advantage	0	Peripheral	2	Dopamine	0	Physical examination	0	Referral	0	Attitude	0	Gene-related	0	Calcitonin	0	Beta-blocker	0	church	0	Temporal	0	Barbiturate	0	Lidocaine	0	Spectrum	0	Motivation	0	Menstrual migraine	0	Correlate	0	Outpatient	0	Injury	0	Lab test	0	Efficacy	0	Antihistamine	0	Lactation	0	Secondary headache	0	Trigeminal autonomic cephalalgia	0	Progression	0	Medication overuse	0	Inpatient treatment	0	Intracranial hypertension	0	Botulinum Toxin	0	Serotonin receptor	0	Mindfulness	0	Cranial nerve	0	Episodic migraine	0	Double vision	0	Vestibular migraine	0	surgical	0	Surgical treatment	0	5-HT	0	Ineffective	0	Eye symptom	0	Female hormone	0
0	Headache clinic	0	Assessment	0	Empower	0	Productivity	0	Caregiver	0	Nerve block	0	Accepting	0	Pain management	0	Mechanism	0	spouse	0	Co-morbid	0	Histamine	0	Tricyclic	0	Comorbid	0	Prevalence	0	Advantage	0	Peripheral	0	Dopamine	0	Physical examination	0	Referral	0	Attitude	0	Gene-related	0	Calcitonin	0	Beta-blocker	0	church	0	Temporal	0	Barbiturate	0	Lidocaine	0	Spectrum	0	Motivation	0	Menstrual migraine	0	Correlate	0	Outpatient	0	Injury	0	Lab test	0	Efficacy	0	Antihistamine	0	Lactation	0	Secondary headache	0	Trigeminal autonomic cephalalgia	0	Progression	0	Medication overuse	0	Inpatient treatment	0	Intracranial hypertension	1	Botulinum Toxin	0	Serotonin receptor	0	Mindfulness	0	Cranial nerve	0	Episodic migraine	0	Double vision	0	Vestibular migraine	0	surgical	0	Surgical treatment	0	5-HT	0	Ineffective	0	Eye symptom	0	Female hormone	0
0	Headache clinic	0	Assessment	0	Empower	0	Productivity	0	Caregiver	0	Nerve block	0	Accepting	0	Pain management	0	Mechanism	1	spouse	0	Co-morbid	0	Histamine	0	Tricyclic	0	Comorbid	0	Prevalence	0	Advantage	0	Peripheral	0	Dopamine	0	Physical examination	0	Referral	0	Attitude	0	Gene-related	0	Calcitonin	0	Beta-blocker	0	church	0	Temporal	0	Barbiturate	0	Lidocaine	0	Spectrum	0	Motivation	0	Menstrual migraine	0	Correlate	0	Outpatient	0	Injury	0	Lab test	0	Efficacy	0	Antihistamine	0	Lactation	0	Secondary headache	0	Trigeminal autonomic cephalalgia	0	Progression	0	Medication overuse	0	Inpatient treatment	0	Intracranial hypertension	0	Botulinum Toxin	0	Serotonin receptor	0	Mindfulness	0	Cranial nerve	0	Episodic migraine	0	Double vision	0	Vestibular migraine	0	surgical	0	Surgical treatment	0	5-HT	0	Ineffective	0	Eye symptom	0	Female hormone	1
0	Headache clinic	0	Assessment	0	Empower	0	Productivity	0	Caregiver	0	Nerve block	0	Accepting	0	Pain management	0	Mechanism	0	spouse	0	Co-morbid	0	Histamine	0	Tricyclic	0	Comorbid	0	Prevalence	0	Advantage	0	Peripheral	0	Dopamine	0	Physical examination	0	Referral	0	Attitude	0	Gene-related	0	Calcitonin	0	Beta-blocker	0	church	1	Temporal	0	Barbiturate	0	Lidocaine	0	Spectrum	0	Motivation	0	Menstrual migraine	0	Correlate	0	Outpatient	0	Injury	0	Lab test	0	Efficacy	0	Antihistamine	0	Lactation	0	Secondary headache	0	Trigeminal autonomic cephalalgia	0	Progression	0	Medication overuse	0	Inpatient treatment	0	Intracranial hypertension	0	Botulinum Toxin	0	Serotonin receptor	0	Mindfulness	0	Cranial nerve	0	Episodic migraine	0	Double vision	0	Vestibular migraine	0	surgical	0	Surgical treatment	0	5-HT	1	Ineffective	1	Eye symptom	1	Female hormone	0
0	Headache clinic	0	Assessment	0	Empower	0	Productivity	0	Caregiver	0	Nerve block	0	Accepting	0	Pain management	0	Mechanism	0	spouse	0	Co-morbid	0	Histamine	0	Tricyclic	0	Comorbid	0	Prevalence	0	Advantage	0	Peripheral	1	Dopamine	0	Physical examination	0	Referral	0	Attitude	0	Gene-related	0	Calcitonin	0	Beta-blocker	0	church	0	Temporal	0	Barbiturate	0	Lidocaine	0	Spectrum	0	Motivation	0	Menstrual migraine	0	Correlate	0	Outpatient	0	Injury	0	Lab test	0	Efficacy	0	Antihistamine	0	Lactation	0	Secondary headache	0	Trigeminal autonomic cephalalgia	0	Progression	0	Medication overuse	0	Inpatient treatment	0	Intracranial hypertension	0	Botulinum Toxin	0	Serotonin receptor	0	Mindfulness	0	Cranial nerve	0	Episodic migraine	0	Double vision	0	Vestibular migraine	0	surgical	0	Surgical treatment	0	5-HT	0	Ineffective	0	Eye symptom	0	Female hormone	0
0	Headache clinic	0	Assessment	0	Empower	0	Productivity	0	Caregiver	0	Nerve block	0	Accepting	0	Pain management	0	Mechanism	0	spouse	0	Co-morbid	0	Histamine	0	Tricyclic	0	Comorbid	0	Prevalence	0	Advantage	1	Peripheral	0	Dopamine	0	Physical examination	0	Referral	0	Attitude	0	Gene-related	0	Calcitonin	0	Beta-blocker	0	church	0	Temporal	0	Barbiturate	0	Lidocaine	0	Spectrum	0	Motivation	0	Menstrual migraine	0	Correlate	0	Outpatient	0	Injury	0	Lab test	0	Efficacy	0	Antihistamine	0	Lactation	0	Secondary headache	0	Trigeminal autonomic cephalalgia	0	Progression	0	Medication overuse	0	Inpatient treatment	0	Intracranial hypertension	0	Botulinum Toxin	0	Serotonin receptor	0	Mindfulness	0	Cranial nerve	0	Episodic migraine	0	Double vision	0	Vestibular migraine	0	surgical	0	Surgical treatment	0	5-HT	0	Ineffective	0	Eye symptom	0	Female hormone	0
0	Headache clinic	0	Assessment	0	Empower	0	Productivity	0	Caregiver	0	Nerve block	0	Accepting	0	Pain management	0	Mechanism	0	spouse	0	Co-morbid	0	Histamine	0	Tricyclic	0	Comorbid	0	Prevalence	0	Advantage	0	Peripheral	0	Dopamine	0	Physical examination	0	Referral	0	Attitude	0	Gene-related	0	Calcitonin	0	Beta-blocker	0	church	0	Temporal	0	Barbiturate	0	Lidocaine	0	Spectrum	0	Motivation	0	Menstrual migraine	0	Correlate	0	Outpatient	0	Injury	0	Lab test	0	Efficacy	0	Antihistamine	0	Lactation	0	Secondary headache	0	Trigeminal autonomic cephalalgia	0	Progression	0	Medication overuse	0	Inpatient treatment	0	Intracranial hypertension	0	Botulinum Toxin	0	Serotonin receptor	0	Mindfulness	0	Cranial nerve	0	Episodic migraine	0	Double vision	0	Vestibular migraine	0	surgical	0	Surgical treatment	0	5-HT	0	Ineffective	0	Eye symptom	0	Female hormone	0
0	Headache clinic	0	Assessment	0	Empower	0	Productivity	0	Caregiver	0	Nerve block	0	Accepting	0	Pain management	0	Mechanism	0	spouse	0	Co-morbid	0	Histamine	0	Tricyclic	0	Comorbid	0	Prevalence	0	Advantage	0	Peripheral	0	Dopamine	0	Physical examination	0	Referral	0	Attitude	0	Gene-related	0	Calcitonin	0	Beta-blocker	0	church	0	Temporal	0	Barbiturate	0	Lidocaine	0	Spectrum	0	Motivation	0	Menstrual migraine	0	Correlate	0	Outpatient	0	Injury	0	Lab test	0	Efficacy	0	Antihistamine	0	Lactation	0	Secondary headache	0	Trigeminal autonomic cephalalgia	0	Progression	0	Medication overuse	0	Inpatient treatment	0	Intracranial hypertension	0	Botulinum Toxin	0	Serotonin receptor	0	Mindfulness	0	Cranial nerve	0	Episodic migraine	0	Double vision	0	Vestibular migraine	0	surgical	0	Surgical treatment	0	5-HT	0	Ineffective	0	Eye symptom	0	Female hormone	0
1	Headache clinic	1	Assessment	0	Empower	0	Productivity	0	Caregiver	0	Nerve block	0	Accepting	0	Pain management	0	Mechanism	0	spouse	0	Co-morbid	0	Histamine	0	Tricyclic	0	Comorbid	0	Prevalence	0	Advantage	0	Peripheral	0	Dopamine	0	Physical examination	0	Referral	0	Attitude	0	Gene-related	0	Calcitonin	0	Beta-blocker	0	church	0	Temporal	0	Barbiturate	0	Lidocaine	0	Spectrum	0	Motivation	0	Menstrual migraine	0	Correlate	0	Outpatient	0	Injury	0	Lab test	0	Efficacy	0	Antihistamine	0	Lactation	0	Secondary headache	0	Trigeminal autonomic cephalalgia	0	Progression	0	Medication overuse	0	Inpatient treatment	1	Intracranial hypertension	0	Botulinum Toxin	0	Serotonin receptor	0	Mindfulness	0	Cranial nerve	0	Episodic migraine	0	Double vision	0	Vestibular migraine	0	surgical	0	Surgical treatment	0	5-HT	0	Ineffective	0	Eye symptom	0	Female hormone	0
0	Headache clinic	0	Assessment	0	Empower	0	Productivity	0	Caregiver	0	Nerve block	0	Accepting	0	Pain management	0	Mechanism	0	spouse	0	Co-morbid	0	Histamine	0	Tricyclic	0	Comorbid	0	Prevalence	0	Advantage	0	Peripheral	0	Dopamine	0	Physical examination	0	Referral	0	Attitude	0	Gene-related	0	Calcitonin	0	Beta-blocker	0	church	0	Temporal	0	Barbiturate	0	Lidocaine	0	Spectrum	0	Motivation	0	Menstrual migraine	0	Correlate	0	Outpatient	0	Injury	0	Lab test	0	Efficacy	0	Antihistamine	0	Lactation	0	Secondary headache	0	Trigeminal autonomic cephalalgia	0	Progression	0	Medication overuse	0	Inpatient treatment	0	Intracranial hypertension	0	Botulinum Toxin	0	Serotonin receptor	0	Mindfulness	0	Cranial nerve	0	Episodic migraine	0	Double vision	0	Vestibular migraine	0	surgical	0	Surgical treatment	0	5-HT	0	Ineffective	0	Eye symptom	0	Female hormone	0
0	Headache clinic	0	Assessment	0	Empower	0	Productivity	0	Caregiver	0	Nerve block	0	Accepting	0	Pain management	0	Mechanism	0	spouse	0	Co-morbid	0	Histamine	0	Tricyclic	0	Comorbid	0	Prevalence	0	Advantage	0	Peripheral	0	Dopamine	0	Physical examination	0	Referral	0	Attitude	0	Gene-related	0	Calcitonin	0	Beta-blocker	0	church	0	Temporal	0	Barbiturate	0	Lidocaine	0	Spectrum	0	Motivation	0	Menstrual migraine	0	Correlate	0	Outpatient	0	Injury	0	Lab test	0	Efficacy	0	Antihistamine	0	Lactation	0	Secondary headache	0	Trigeminal autonomic cephalalgia	0	Progression	0	Medication overuse	0	Inpatient treatment	0	Intracranial hypertension	0	Botulinum Toxin	0	Serotonin receptor	0	Mindfulness	0	Cranial nerve	0	Episodic migraine	0	Double vision	0	Vestibular migraine	0	surgical	0	Surgical treatment	0	5-HT	0	Ineffective	0	Eye symptom	0	Female hormone	0
0	Headache clinic	0	Assessment	0	Empower	0	Productivity	1	Caregiver	1	Nerve block	0	Accepting	0	Pain management	0	Mechanism	0	spouse	0	Co-morbid	0	Histamine	0	Tricyclic	0	Comorbid	0	Prevalence	0	Advantage	0	Peripheral	0	Dopamine	0	Physical examination	0	Referral	0	Attitude	0	Gene-related	1	Calcitonin	1	Beta-blocker	0	church	0	Temporal	0	Barbiturate	0	Lidocaine	0	Spectrum	0	Motivation	0	Menstrual migraine	0	Correlate	0	Outpatient	0	Injury	0	Lab test	0	Efficacy	0	Antihistamine	0	Lactation	0	Secondary headache	0	Trigeminal autonomic cephalalgia	0	Progression	0	Medication overuse	0	Inpatient treatment	0	Intracranial hypertension	0	Botulinum Toxin	0	Serotonin receptor	0	Mindfulness	0	Cranial nerve	0	Episodic migraine	0	Double vision	0	Vestibular migraine	0	surgical	0	Surgical treatment	0	5-HT	0	Ineffective	0	Eye symptom	0	Female hormone	0
0	Headache clinic	0	Assessment	0	Empower	0	Productivity	1	Caregiver	1	Nerve block	0	Accepting	0	Pain management	0	Mechanism	0	spouse	0	Co-morbid	0	Histamine	0	Tricyclic	0	Comorbid	0	Prevalence	0	Advantage	0	Peripheral	0	Dopamine	0	Physical examination	0	Referral	0	Attitude	0	Gene-related	1	Calcitonin	1	Beta-blocker	0	church	0	Temporal	0	Barbiturate	0	Lidocaine	0	Spectrum	0	Motivation	0	Menstrual migraine	0	Correlate	0	Outpatient	0	Injury	0	Lab test	0	Efficacy	0	Antihistamine	0	Lactation	0	Secondary headache	0	Trigeminal autonomic cephalalgia	0	Progression	0	Medication overuse	0	Inpatient treatment	0	Intracranial hypertension	0	Botulinum Toxin	0	Serotonin receptor	0	Mindfulness	0	Cranial nerve	0	Episodic migraine	0	Double vision	0	Vestibular migraine	0	surgical	0	Surgical treatment	0	5-HT	0	Ineffective	0	Eye symptom	0	Female hormone	0
0	Headache clinic	0	Assessment	0	Empower	1	Productivity	0	Caregiver	0	Nerve block	0	Accepting	0	Pain management	0	Mechanism	0	spouse	0	Co-morbid	0	Histamine	0	Tricyclic	0	Comorbid	0	Prevalence	0	Advantage	0	Peripheral	0	Dopamine	0	Physical examination	0	Referral	0	Attitude	0	Gene-related	0	Calcitonin	0	Beta-blocker	0	church	0	Temporal	0	Barbiturate	0	Lidocaine	0	Spectrum	0	Motivation	0	Menstrual migraine	0	Correlate	0	Outpatient	0	Injury	0	Lab test	0	Efficacy	0	Antihistamine	0	Lactation	0	Secondary headache	0	Trigeminal autonomic cephalalgia	0	Progression	0	Medication overuse	2	Inpatient treatment	0	Intracranial hypertension	0	Botulinum Toxin	0	Serotonin receptor	0	Mindfulness	1	Cranial nerve	0	Episodic migraine	0	Double vision	0	Vestibular migraine	0	surgical	0	Surgical treatment	0	5-HT	0	Ineffective	0	Eye symptom	0	Female hormone	0
0	Headache clinic	0	Assessment	0	Empower	0	Productivity	0	Caregiver	0	Nerve block	0	Accepting	0	Pain management	0	Mechanism	0	spouse	0	Co-morbid	0	Histamine	0	Tricyclic	0	Comorbid	0	Prevalence	0	Advantage	0	Peripheral	0	Dopamine	0	Physical examination	0	Referral	0	Attitude	0	Gene-related	0	Calcitonin	0	Beta-blocker	0	church	0	Temporal	0	Barbiturate	0	Lidocaine	0	Spectrum	0	Motivation	0	Menstrual migraine	0	Correlate	0	Outpatient	0	Injury	0	Lab test	0	Efficacy	0	Antihistamine	0	Lactation	0	Secondary headache	0	Trigeminal autonomic cephalalgia	0	Progression	0	Medication overuse	0	Inpatient treatment	0	Intracranial hypertension	0	Botulinum Toxin	0	Serotonin receptor	0	Mindfulness	0	Cranial nerve	0	Episodic migraine	0	Double vision	0	Vestibular migraine	0	surgical	0	Surgical treatment	0	5-HT	0	Ineffective	0	Eye symptom	0	Female hormone	0
0	Headache clinic	0	Assessment	0	Empower	0	Productivity	0	Caregiver	0	Nerve block	0	Accepting	0	Pain management	0	Mechanism	0	spouse	0	Co-morbid	0	Histamine	0	Tricyclic	0	Comorbid	0	Prevalence	0	Advantage	0	Peripheral	0	Dopamine	0	Physical examination	0	Referral	0	Attitude	0	Gene-related	0	Calcitonin	0	Beta-blocker	0	church	0	Temporal	0	Barbiturate	0	Lidocaine	0	Spectrum	0	Motivation	0	Menstrual migraine	0	Correlate	0	Outpatient	0	Injury	0	Lab test	0	Efficacy	0	Antihistamine	0	Lactation	0	Secondary headache	0	Trigeminal autonomic cephalalgia	0	Progression	0	Medication overuse	0	Inpatient treatment	0	Intracranial hypertension	0	Botulinum Toxin	0	Serotonin receptor	0	Mindfulness	0	Cranial nerve	0	Episodic migraine	0	Double vision	0	Vestibular migraine	0	surgical	0	Surgical treatment	0	5-HT	0	Ineffective	0	Eye symptom	0	Female hormone	0
0	Headache clinic	0	Assessment	0	Empower	0	Productivity	0	Caregiver	0	Nerve block	0	Accepting	0	Pain management	0	Mechanism	0	spouse	0	Co-morbid	0	Histamine	0	Tricyclic	0	Comorbid	0	Prevalence	0	Advantage	0	Peripheral	0	Dopamine	0	Physical examination	0	Referral	0	Attitude	1	Gene-related	0	Calcitonin	0	Beta-blocker	0	church	0	Temporal	0	Barbiturate	0	Lidocaine	0	Spectrum	0	Motivation	0	Menstrual migraine	0	Correlate	0	Outpatient	0	Injury	0	Lab test	0	Efficacy	0	Antihistamine	0	Lactation	0	Secondary headache	0	Trigeminal autonomic cephalalgia	0	Progression	0	Medication overuse	0	Inpatient treatment	0	Intracranial hypertension	0	Botulinum Toxin	0	Serotonin receptor	0	Mindfulness	0	Cranial nerve	0	Episodic migraine	0	Double vision	0	Vestibular migraine	0	surgical	0	Surgical treatment	0	5-HT	0	Ineffective	0	Eye symptom	0	Female hormone	0
0	Headache clinic	0	Assessment	0	Empower	0	Productivity	0	Caregiver	0	Nerve block	0	Accepting	0	Pain management	0	Mechanism	0	spouse	0	Co-morbid	0	Histamine	0	Tricyclic	0	Comorbid	0	Prevalence	0	Advantage	0	Peripheral	0	Dopamine	0	Physical examination	0	Referral	0	Attitude	0	Gene-related	0	Calcitonin	0	Beta-blocker	0	church	0	Temporal	0	Barbiturate	0	Lidocaine	0	Spectrum	1	Motivation	0	Menstrual migraine	0	Correlate	0	Outpatient	0	Injury	0	Lab test	0	Efficacy	0	Antihistamine	0	Lactation	0	Secondary headache	0	Trigeminal autonomic cephalalgia	0	Progression	0	Medication overuse	0	Inpatient treatment	0	Intracranial hypertension	0	Botulinum Toxin	0	Serotonin receptor	0	Mindfulness	0	Cranial nerve	0	Episodic migraine	0	Double vision	0	Vestibular migraine	0	surgical	0	Surgical treatment	0	5-HT	0	Ineffective	0	Eye symptom	0	Female hormone	0
0	Headache clinic	0	Assessment	0	Empower	0	Productivity	0	Caregiver	0	Nerve block	0	Accepting	0	Pain management	0	Mechanism	0	spouse	1	Co-morbid	0	Histamine	0	Tricyclic	0	Comorbid	0	Prevalence	0	Advantage	0	Peripheral	0	Dopamine	0	Physical examination	0	Referral	0	Attitude	0	Gene-related	0	Calcitonin	0	Beta-blocker	0	church	0	Temporal	0	Barbiturate	0	Lidocaine	0	Spectrum	0	Motivation	0	Menstrual migraine	0	Correlate	0	Outpatient	0	Injury	0	Lab test	0	Efficacy	0	Antihistamine	0	Lactation	0	Secondary headache	0	Trigeminal autonomic cephalalgia	0	Progression	1	Medication overuse	0	Inpatient treatment	0	Intracranial hypertension	0	Botulinum Toxin	0	Serotonin receptor	0	Mindfulness	0	Cranial nerve	0	Episodic migraine	0	Double vision	0	Vestibular migraine	0	surgical	0	Surgical treatment	0	5-HT	0	Ineffective	0	Eye symptom	0	Female hormone	0
0	Headache clinic	0	Assessment	0	Empower	0	Productivity	0	Caregiver	0	Nerve block	0	Accepting	0	Pain management	0	Mechanism	0	spouse	0	Co-morbid	0	Histamine	0	Tricyclic	0	Comorbid	0	Prevalence	0	Advantage	0	Peripheral	0	Dopamine	0	Physical examination	0	Referral	0	Attitude	0	Gene-related	0	Calcitonin	0	Beta-blocker	0	church	0	Temporal	0	Barbiturate	0	Lidocaine	0	Spectrum	0	Motivation	0	Menstrual migraine	0	Correlate	0	Outpatient	0	Injury	0	Lab test	0	Efficacy	0	Antihistamine	0	Lactation	0	Secondary headache	2	Trigeminal autonomic cephalalgia	2	Progression	0	Medication overuse	0	Inpatient treatment	0	Intracranial hypertension	0	Botulinum Toxin	0	Serotonin receptor	0	Mindfulness	0	Cranial nerve	0	Episodic migraine	0	Double vision	0	Vestibular migraine	0	surgical	0	Surgical treatment	0	5-HT	0	Ineffective	0	Eye symptom	0	Female hormone	0
0	Headache clinic	0	Assessment	0	Empower	0	Productivity	0	Caregiver	0	Nerve block	0	Accepting	0	Pain management	0	Mechanism	0	spouse	0	Co-morbid	0	Histamine	0	Tricyclic	0	Comorbid	0	Prevalence	0	Advantage	0	Peripheral	0	Dopamine	0	Physical examination	0	Referral	0	Attitude	0	Gene-related	0	Calcitonin	0	Beta-blocker	0	church	0	Temporal	0	Barbiturate	0	Lidocaine	0	Spectrum	0	Motivation	0	Menstrual migraine	0	Correlate	0	Outpatient	0	Injury	0	Lab test	0	Efficacy	0	Antihistamine	0	Lactation	0	Secondary headache	0	Trigeminal autonomic cephalalgia	0	Progression	0	Medication overuse	0	Inpatient treatment	0	Intracranial hypertension	0	Botulinum Toxin	0	Serotonin receptor	0	Mindfulness	0	Cranial nerve	0	Episodic migraine	0	Double vision	0	Vestibular migraine	0	surgical	0	Surgical treatment	0	5-HT	0	Ineffective	0	Eye symptom	0	Female hormone	0
0	Headache clinic	0	Assessment	0	Empower	0	Productivity	0	Caregiver	0	Nerve block	0	Accepting	0	Pain management	0	Mechanism	0	spouse	0	Co-morbid	0	Histamine	0	Tricyclic	0	Comorbid	0	Prevalence	0	Advantage	0	Peripheral	0	Dopamine	1	Physical examination	0	Referral	0	Attitude	0	Gene-related	0	Calcitonin	0	Beta-blocker	0	church	1	Temporal	0	Barbiturate	0	Lidocaine	0	Spectrum	0	Motivation	0	Menstrual migraine	0	Correlate	0	Outpatient	0	Injury	0	Lab test	0	Efficacy	0	Antihistamine	0	Lactation	0	Secondary headache	0	Trigeminal autonomic cephalalgia	0	Progression	0	Medication overuse	0	Inpatient treatment	0	Intracranial hypertension	1	Botulinum Toxin	0	Serotonin receptor	0	Mindfulness	0	Cranial nerve	0	Episodic migraine	0	Double vision	1	Vestibular migraine	0	surgical	0	Surgical treatment	0	5-HT	0	Ineffective	0	Eye symptom	0	Female hormone	0
0	Headache clinic	0	Assessment	1	Empower	0	Productivity	0	Caregiver	0	Nerve block	0	Accepting	0	Pain management	0	Mechanism	0	spouse	0	Co-morbid	0	Histamine	0	Tricyclic	0	Comorbid	0	Prevalence	0	Advantage	0	Peripheral	0	Dopamine	0	Physical examination	0	Referral	0	Attitude	0	Gene-related	0	Calcitonin	0	Beta-blocker	0	church	0	Temporal	0	Barbiturate	0	Lidocaine	0	Spectrum	0	Motivation	0	Menstrual migraine	0	Correlate	0	Outpatient	0	Injury	0	Lab test	0	Efficacy	0	Antihistamine	0	Lactation	0	Secondary headache	0	Trigeminal autonomic cephalalgia	0	Progression	0	Medication overuse	0	Inpatient treatment	0	Intracranial hypertension	0	Botulinum Toxin	0	Serotonin receptor	0	Mindfulness	0	Cranial nerve	0	Episodic migraine	0	Double vision	0	Vestibular migraine	0	surgical	0	Surgical treatment	0	5-HT	0	Ineffective	0	Eye symptom	0	Female hormone	0
0	Headache clinic	0	Assessment	0	Empower	0	Productivity	0	Caregiver	0	Nerve block	0	Accepting	0	Pain management	0	Mechanism	0	spouse	0	Co-morbid	0	Histamine	0	Tricyclic	0	Comorbid	2	Prevalence	1	Advantage	0	Peripheral	0	Dopamine	0	Physical examination	0	Referral	0	Attitude	0	Gene-related	0	Calcitonin	0	Beta-blocker	0	church	0	Temporal	0	Barbiturate	0	Lidocaine	0	Spectrum	0	Motivation	0	Menstrual migraine	0	Correlate	0	Outpatient	0	Injury	0	Lab test	0	Efficacy	0	Antihistamine	0	Lactation	0	Secondary headache	0	Trigeminal autonomic cephalalgia	0	Progression	0	Medication overuse	0	Inpatient treatment	0	Intracranial hypertension	0	Botulinum Toxin	0	Serotonin receptor	0	Mindfulness	0	Cranial nerve	0	Episodic migraine	0	Double vision	0	Vestibular migraine	0	surgical	0	Surgical treatment	0	5-HT	0	Ineffective	0	Eye symptom	0	Female hormone	0
0	Headache clinic	0	Assessment	0	Empower	0	Productivity	0	Caregiver	0	Nerve block	0	Accepting	0	Pain management	0	Mechanism	0	spouse	0	Co-morbid	0	Histamine	0	Tricyclic	0	Comorbid	0	Prevalence	0	Advantage	0	Peripheral	0	Dopamine	0	Physical examination	0	Referral	0	Attitude	0	Gene-related	0	Calcitonin	0	Beta-blocker	0	church	0	Temporal	0	Barbiturate	0	Lidocaine	0	Spectrum	0	Motivation	0	Menstrual migraine	0	Correlate	0	Outpatient	0	Injury	0	Lab test	0	Efficacy	0	Antihistamine	0	Lactation	0	Secondary headache	0	Trigeminal autonomic cephalalgia	0	Progression	0	Medication overuse	0	Inpatient treatment	0	Intracranial hypertension	0	Botulinum Toxin	0	Serotonin receptor	0	Mindfulness	0	Cranial nerve	0	Episodic migraine	0	Double vision	0	Vestibular migraine	0	surgical	0	Surgical treatment	0	5-HT	0	Ineffective	0	Eye symptom	0	Female hormone	0
0	Headache clinic	0	Assessment	0	Empower	0	Productivity	0	Caregiver	0	Nerve block	0	Accepting	0	Pain management	0	Mechanism	0	spouse	0	Co-morbid	0	Histamine	0	Tricyclic	0	Comorbid	0	Prevalence	0	Advantage	0	Peripheral	0	Dopamine	0	Physical examination	0	Referral	0	Attitude	0	Gene-related	0	Calcitonin	0	Beta-blocker	0	church	0	Temporal	0	Barbiturate	0	Lidocaine	0	Spectrum	0	Motivation	0	Menstrual migraine	0	Correlate	0	Outpatient	0	Injury	0	Lab test	0	Efficacy	0	Antihistamine	0	Lactation	0	Secondary headache	0	Trigeminal autonomic cephalalgia	0	Progression	0	Medication overuse	0	Inpatient treatment	0	Intracranial hypertension	0	Botulinum Toxin	0	Serotonin receptor	0	Mindfulness	0	Cranial nerve	0	Episodic migraine	0	Double vision	0	Vestibular migraine	0	surgical	0	Surgical treatment	0	5-HT	0	Ineffective	0	Eye symptom	0	Female hormone	0
0	Headache clinic	0	Assessment	0	Empower	0	Productivity	0	Caregiver	0	Nerve block	0	Accepting	0	Pain management	0	Mechanism	0	spouse	0	Co-morbid	0	Histamine	0	Tricyclic	0	Comorbid	0	Prevalence	0	Advantage	0	Peripheral	0	Dopamine	0	Physical examination	0	Referral	0	Attitude	0	Gene-related	0	Calcitonin	0	Beta-blocker	0	church	0	Temporal	0	Barbiturate	0	Lidocaine	0	Spectrum	0	Motivation	0	Menstrual migraine	0	Correlate	0	Outpatient	0	Injury	0	Lab test	0	Efficacy	0	Antihistamine	0	Lactation	0	Secondary headache	0	Trigeminal autonomic cephalalgia	0	Progression	0	Medication overuse	0	Inpatient treatment	0	Intracranial hypertension	0	Botulinum Toxin	0	Serotonin receptor	0	Mindfulness	0	Cranial nerve	0	Episodic migraine	0	Double vision	0	Vestibular migraine	0	surgical	0	Surgical treatment	0	5-HT	0	Ineffective	0	Eye symptom	0	Female hormone	0
0	Headache clinic	0	Assessment	0	Empower	0	Productivity	0	Caregiver	0	Nerve block	0	Accepting	0	Pain management	0	Mechanism	0	spouse	0	Co-morbid	0	Histamine	0	Tricyclic	0	Comorbid	0	Prevalence	0	Advantage	0	Peripheral	0	Dopamine	0	Physical examination	0	Referral	0	Attitude	0	Gene-related	0	Calcitonin	0	Beta-blocker	0	church	0	Temporal	0	Barbiturate	0	Lidocaine	0	Spectrum	0	Motivation	0	Menstrual migraine	0	Correlate	0	Outpatient	0	Injury	0	Lab test	0	Efficacy	0	Antihistamine	0	Lactation	0	Secondary headache	0	Trigeminal autonomic cephalalgia	0	Progression	0	Medication overuse	0	Inpatient treatment	0	Intracranial hypertension	0	Botulinum Toxin	0	Serotonin receptor	0	Mindfulness	0	Cranial nerve	0	Episodic migraine	0	Double vision	0	Vestibular migraine	0	surgical	0	Surgical treatment	0	5-HT	0	Ineffective	0	Eye symptom	0	Female hormone	0
0	Headache clinic	0	Assessment	0	Empower	2	Productivity	0	Caregiver	0	Nerve block	0	Accepting	0	Pain management	0	Mechanism	0	spouse	0	Co-morbid	0	Histamine	0	Tricyclic	0	Comorbid	0	Prevalence	0	Advantage	0	Peripheral	0	Dopamine	0	Physical examination	0	Referral	0	Attitude	0	Gene-related	0	Calcitonin	0	Beta-blocker	0	church	0	Temporal	0	Barbiturate	0	Lidocaine	0	Spectrum	0	Motivation	0	Menstrual migraine	0	Correlate	0	Outpatient	0	Injury	0	Lab test	0	Efficacy	0	Antihistamine	0	Lactation	0	Secondary headache	0	Trigeminal autonomic cephalalgia	0	Progression	0	Medication overuse	0	Inpatient treatment	0	Intracranial hypertension	0	Botulinum Toxin	0	Serotonin receptor	0	Mindfulness	0	Cranial nerve	0	Episodic migraine	0	Double vision	0	Vestibular migraine	0	surgical	0	Surgical treatment	0	5-HT	0	Ineffective	0	Eye symptom	0	Female hormone	0
0	Headache clinic	0	Assessment	0	Empower	0	Productivity	0	Caregiver	0	Nerve block	0	Accepting	0	Pain management	0	Mechanism	0	spouse	0	Co-morbid	0	Histamine	0	Tricyclic	0	Comorbid	0	Prevalence	0	Advantage	0	Peripheral	0	Dopamine	0	Physical examination	0	Referral	0	Attitude	0	Gene-related	0	Calcitonin	0	Beta-blocker	0	church	0	Temporal	0	Barbiturate	0	Lidocaine	0	Spectrum	0	Motivation	0	Menstrual migraine	0	Correlate	0	Outpatient	0	Injury	0	Lab test	0	Efficacy	0	Antihistamine	0	Lactation	0	Secondary headache	0	Trigeminal autonomic cephalalgia	0	Progression	0	Medication overuse	0	Inpatient treatment	0	Intracranial hypertension	0	Botulinum Toxin	0	Serotonin receptor	0	Mindfulness	0	Cranial nerve	0	Episodic migraine	0	Double vision	0	Vestibular migraine	0	surgical	1	Surgical treatment	1	5-HT	0	Ineffective	0	Eye symptom	0	Female hormone	0
0	Headache clinic	0	Assessment	0	Empower	0	Productivity	0	Caregiver	0	Nerve block	0	Accepting	0	Pain management	0	Mechanism	0	spouse	0	Co-morbid	0	Histamine	0	Tricyclic	0	Comorbid	0	Prevalence	0	Advantage	0	Peripheral	0	Dopamine	0	Physical examination	0	Referral	0	Attitude	0	Gene-related	0	Calcitonin	0	Beta-blocker	0	church	0	Temporal	0	Barbiturate	0	Lidocaine	0	Spectrum	0	Motivation	0	Menstrual migraine	0	Correlate	0	Outpatient	0	Injury	0	Lab test	0	Efficacy	0	Antihistamine	0	Lactation	0	Secondary headache	0	Trigeminal autonomic cephalalgia	0	Progression	0	Medication overuse	0	Inpatient treatment	0	Intracranial hypertension	0	Botulinum Toxin	0	Serotonin receptor	0	Mindfulness	0	Cranial nerve	0	Episodic migraine	0	Double vision	0	Vestibular migraine	0	surgical	0	Surgical treatment	0	5-HT	0	Ineffective	0	Eye symptom	0	Female hormone	0
0	Headache clinic	0	Assessment	0	Empower	0	Productivity	0	Caregiver	0	Nerve block	0	Accepting	0	Pain management	0	Mechanism	0	spouse	0	Co-morbid	0	Histamine	0	Tricyclic	1	Comorbid	0	Prevalence	0	Advantage	0	Peripheral	0	Dopamine	0	Physical examination	0	Referral	0	Attitude	0	Gene-related	0	Calcitonin	0	Beta-blocker	0	church	0	Temporal	0	Barbiturate	0	Lidocaine	0	Spectrum	0	Motivation	0	Menstrual migraine	1	Correlate	0	Outpatient	0	Injury	0	Lab test	0	Efficacy	0	Antihistamine	0	Lactation	2	Secondary headache	0	Trigeminal autonomic cephalalgia	0	Progression	0	Medication overuse	0	Inpatient treatment	0	Intracranial hypertension	0	Botulinum Toxin	0	Serotonin receptor	0	Mindfulness	0	Cranial nerve	0	Episodic migraine	0	Double vision	0	Vestibular migraine	0	surgical	0	Surgical treatment	0	5-HT	0	Ineffective	0	Eye symptom	0	Female hormone	0
0	Headache clinic	3	Assessment	0	Empower	0	Productivity	0	Caregiver	0	Nerve block	0	Accepting	0	Pain management	0	Mechanism	0	spouse	0	Co-morbid	0	Histamine	2	Tricyclic	2	Comorbid	0	Prevalence	0	Advantage	2	Peripheral	0	Dopamine	1	Physical examination	0	Referral	0	Attitude	0	Gene-related	0	Calcitonin	0	Beta-blocker	0	church	0	Temporal	0	Barbiturate	0	Lidocaine	0	Spectrum	0	Motivation	0	Menstrual migraine	0	Correlate	0	Outpatient	0	Injury	0	Lab test	0	Efficacy	2	Antihistamine	1	Lactation	0	Secondary headache	0	Trigeminal autonomic cephalalgia	0	Progression	1	Medication overuse	0	Inpatient treatment	0	Intracranial hypertension	0	Botulinum Toxin	1	Serotonin receptor	0	Mindfulness	0	Cranial nerve	0	Episodic migraine	0	Double vision	0	Vestibular migraine	0	surgical	0	Surgical treatment	0	5-HT	0	Ineffective	0	Eye symptom	0	Female hormone	0
0	Headache clinic	0	Assessment	0	Empower	0	Productivity	0	Caregiver	0	Nerve block	0	Accepting	0	Pain management	0	Mechanism	0	spouse	0	Co-morbid	0	Histamine	0	Tricyclic	0	Comorbid	0	Prevalence	0	Advantage	0	Peripheral	0	Dopamine	0	Physical examination	0	Referral	0	Attitude	0	Gene-related	0	Calcitonin	0	Beta-blocker	1	church	0	Temporal	0	Barbiturate	0	Lidocaine	0	Spectrum	0	Motivation	0	Menstrual migraine	0	Correlate	0	Outpatient	0	Injury	0	Lab test	0	Efficacy	0	Antihistamine	0	Lactation	0	Secondary headache	0	Trigeminal autonomic cephalalgia	0	Progression	0	Medication overuse	0	Inpatient treatment	0	Intracranial hypertension	0	Botulinum Toxin	0	Serotonin receptor	0	Mindfulness	0	Cranial nerve	0	Episodic migraine	0	Double vision	0	Vestibular migraine	0	surgical	0	Surgical treatment	0	5-HT	0	Ineffective	0	Eye symptom	0	Female hormone	0
3	Headache clinic	0	Assessment	2	Empower	0	Productivity	0	Caregiver	0	Nerve block	0	Accepting	0	Pain management	0	Mechanism	0	spouse	0	Co-morbid	3	Histamine	0	Tricyclic	0	Comorbid	1	Prevalence	2	Advantage	0	Peripheral	0	Dopamine	0	Physical examination	2	Referral	2	Attitude	0	Gene-related	0	Calcitonin	0	Beta-blocker	0	church	0	Temporal	0	Barbiturate	0	Lidocaine	0	Spectrum	0	Motivation	1	Menstrual migraine	0	Correlate	2	Outpatient	2	Injury	1	Lab test	2	Efficacy	0	Antihistamine	0	Lactation	0	Secondary headache	0	Trigeminal autonomic cephalalgia	0	Progression	0	Medication overuse	0	Inpatient treatment	1	Intracranial hypertension	0	Botulinum Toxin	0	Serotonin receptor	0	Mindfulness	0	Cranial nerve	0	Episodic migraine	0	Double vision	0	Vestibular migraine	0	surgical	0	Surgical treatment	0	5-HT	0	Ineffective	0	Eye symptom	0	Female hormone	0
0	Headache clinic	0	Assessment	0	Empower	0	Productivity	0	Caregiver	0	Nerve block	0	Accepting	0	Pain management	1	Mechanism	0	spouse	0	Co-morbid	0	Histamine	0	Tricyclic	0	Comorbid	0	Prevalence	0	Advantage	0	Peripheral	0	Dopamine	0	Physical examination	0	Referral	0	Attitude	0	Gene-related	0	Calcitonin	0	Beta-blocker	0	church	0	Temporal	0	Barbiturate	1	Lidocaine	0	Spectrum	0	Motivation	0	Menstrual migraine	0	Correlate	0	Outpatient	0	Injury	1	Lab test	0	Efficacy	0	Antihistamine	0	Lactation	0	Secondary headache	0	Trigeminal autonomic cephalalgia	0	Progression	0	Medication overuse	0	Inpatient treatment	0	Intracranial hypertension	0	Botulinum Toxin	0	Serotonin receptor	0	Mindfulness	0	Cranial nerve	0	Episodic migraine	0	Double vision	0	Vestibular migraine	0	surgical	0	Surgical treatment	0	5-HT	0	Ineffective	0	Eye symptom	0	Female hormone	0
0	Headache clinic	0	Assessment	0	Empower	0	Productivity	0	Caregiver	0	Nerve block	0	Accepting	0	Pain management	0	Mechanism	0	spouse	0	Co-morbid	0	Histamine	0	Tricyclic	0	Comorbid	0	Prevalence	0	Advantage	0	Peripheral	0	Dopamine	0	Physical examination	0	Referral	0	Attitude	0	Gene-related	0	Calcitonin	0	Beta-blocker	0	church	0	Temporal	0	Barbiturate	0	Lidocaine	0	Spectrum	0	Motivation	0	Menstrual migraine	0	Correlate	0	Outpatient	0	Injury	0	Lab test	0	Efficacy	0	Antihistamine	0	Lactation	0	Secondary headache	0	Trigeminal autonomic cephalalgia	0	Progression	0	Medication overuse	0	Inpatient treatment	0	Intracranial hypertension	0	Botulinum Toxin	0	Serotonin receptor	0	Mindfulness	0	Cranial nerve	0	Episodic migraine	0	Double vision	0	Vestibular migraine	0	surgical	0	Surgical treatment	0	5-HT	0	Ineffective	0	Eye symptom	0	Female hormone	0
0	Headache clinic	0	Assessment	0	Empower	0	Productivity	0	Caregiver	0	Nerve block	0	Accepting	0	Pain management	0	Mechanism	0	spouse	0	Co-morbid	0	Histamine	0	Tricyclic	0	Comorbid	0	Prevalence	0	Advantage	0	Peripheral	0	Dopamine	0	Physical examination	0	Referral	0	Attitude	0	Gene-related	0	Calcitonin	0	Beta-blocker	0	church	0	Temporal	0	Barbiturate	0	Lidocaine	0	Spectrum	0	Motivation	0	Menstrual migraine	0	Correlate	0	Outpatient	0	Injury	0	Lab test	0	Efficacy	0	Antihistamine	0	Lactation	0	Secondary headache	0	Trigeminal autonomic cephalalgia	0	Progression	0	Medication overuse	0	Inpatient treatment	0	Intracranial hypertension	0	Botulinum Toxin	0	Serotonin receptor	0	Mindfulness	0	Cranial nerve	0	Episodic migraine	0	Double vision	0	Vestibular migraine	0	surgical	0	Surgical treatment	0	5-HT	0	Ineffective	0	Eye symptom	0	Female hormone	0
0	Headache clinic	0	Assessment	0	Empower	0	Productivity	0	Caregiver	0	Nerve block	0	Accepting	0	Pain management	0	Mechanism	0	spouse	0	Co-morbid	0	Histamine	0	Tricyclic	0	Comorbid	0	Prevalence	0	Advantage	0	Peripheral	0	Dopamine	0	Physical examination	0	Referral	0	Attitude	0	Gene-related	0	Calcitonin	0	Beta-blocker	0	church	0	Temporal	0	Barbiturate	0	Lidocaine	0	Spectrum	0	Motivation	0	Menstrual migraine	0	Correlate	0	Outpatient	0	Injury	0	Lab test	0	Efficacy	0	Antihistamine	0	Lactation	0	Secondary headache	0	Trigeminal autonomic cephalalgia	0	Progression	0	Medication overuse	0	Inpatient treatment	0	Intracranial hypertension	0	Botulinum Toxin	0	Serotonin receptor	0	Mindfulness	0	Cranial nerve	0	Episodic migraine	0	Double vision	0	Vestibular migraine	0	surgical	0	Surgical treatment	0	5-HT	0	Ineffective	0	Eye symptom	0	Female hormone	0
0	Headache clinic	0	Assessment	0	Empower	0	Productivity	0	Caregiver	0	Nerve block	0	Accepting	0	Pain management	0	Mechanism	0	spouse	0	Co-morbid	0	Histamine	0	Tricyclic	0	Comorbid	0	Prevalence	0	Advantage	0	Peripheral	0	Dopamine	0	Physical examination	0	Referral	0	Attitude	0	Gene-related	0	Calcitonin	0	Beta-blocker	0	church	0	Temporal	0	Barbiturate	0	Lidocaine	0	Spectrum	0	Motivation	0	Menstrual migraine	0	Correlate	0	Outpatient	0	Injury	0	Lab test	0	Efficacy	0	Antihistamine	0	Lactation	0	Secondary headache	0	Trigeminal autonomic cephalalgia	0	Progression	0	Medication overuse	0	Inpatient treatment	0	Intracranial hypertension	0	Botulinum Toxin	0	Serotonin receptor	0	Mindfulness	0	Cranial nerve	0	Episodic migraine	0	Double vision	0	Vestibular migraine	0	surgical	0	Surgical treatment	0	5-HT	0	Ineffective	0	Eye symptom	0	Female hormone	0
0	Headache clinic	0	Assessment	0	Empower	0	Productivity	0	Caregiver	0	Nerve block	0	Accepting	0	Pain management	0	Mechanism	0	spouse	0	Co-morbid	0	Histamine	0	Tricyclic	0	Comorbid	0	Prevalence	0	Advantage	0	Peripheral	0	Dopamine	0	Physical examination	0	Referral	0	Attitude	0	Gene-related	0	Calcitonin	0	Beta-blocker	0	church	0	Temporal	0	Barbiturate	0	Lidocaine	0	Spectrum	0	Motivation	0	Menstrual migraine	0	Correlate	0	Outpatient	0	Injury	0	Lab test	0	Efficacy	0	Antihistamine	0	Lactation	0	Secondary headache	0	Trigeminal autonomic cephalalgia	0	Progression	0	Medication overuse	0	Inpatient treatment	0	Intracranial hypertension	0	Botulinum Toxin	0	Serotonin receptor	0	Mindfulness	0	Cranial nerve	0	Episodic migraine	0	Double vision	0	Vestibular migraine	0	surgical	0	Surgical treatment	0	5-HT	0	Ineffective	0	Eye symptom	0	Female hormone	0
0	Headache clinic	0	Assessment	0	Empower	0	Productivity	0	Caregiver	0	Nerve block	0	Accepting	0	Pain management	0	Mechanism	0	spouse	0	Co-morbid	0	Histamine	1	Tricyclic	0	Comorbid	0	Prevalence	0	Advantage	0	Peripheral	0	Dopamine	0	Physical examination	0	Referral	0	Attitude	0	Gene-related	0	Calcitonin	0	Beta-blocker	0	church	0	Temporal	0	Barbiturate	0	Lidocaine	0	Spectrum	0	Motivation	0	Menstrual migraine	0	Correlate	0	Outpatient	0	Injury	0	Lab test	0	Efficacy	0	Antihistamine	1	Lactation	0	Secondary headache	0	Trigeminal autonomic cephalalgia	0	Progression	0	Medication overuse	0	Inpatient treatment	0	Intracranial hypertension	0	Botulinum Toxin	0	Serotonin receptor	0	Mindfulness	0	Cranial nerve	0	Episodic migraine	0	Double vision	0	Vestibular migraine	0	surgical	0	Surgical treatment	0	5-HT	0	Ineffective	0	Eye symptom	0	Female hormone	0
0	Headache clinic	0	Assessment	0	Empower	0	Productivity	0	Caregiver	0	Nerve block	0	Accepting	0	Pain management	0	Mechanism	0	spouse	1	Co-morbid	0	Histamine	0	Tricyclic	0	Comorbid	0	Prevalence	0	Advantage	0	Peripheral	0	Dopamine	0	Physical examination	0	Referral	0	Attitude	1	Gene-related	0	Calcitonin	0	Beta-blocker	0	church	0	Temporal	0	Barbiturate	0	Lidocaine	0	Spectrum	0	Motivation	0	Menstrual migraine	0	Correlate	0	Outpatient	0	Injury	0	Lab test	0	Efficacy	0	Antihistamine	0	Lactation	0	Secondary headache	0	Trigeminal autonomic cephalalgia	0	Progression	0	Medication overuse	0	Inpatient treatment	0	Intracranial hypertension	0	Botulinum Toxin	0	Serotonin receptor	0	Mindfulness	0	Cranial nerve	0	Episodic migraine	0	Double vision	0	Vestibular migraine	0	surgical	0	Surgical treatment	0	5-HT	0	Ineffective	0	Eye symptom	0	Female hormone	0
0	Headache clinic	0	Assessment	0	Empower	0	Productivity	0	Caregiver	0	Nerve block	0	Accepting	0	Pain management	0	Mechanism	0	spouse	0	Co-morbid	0	Histamine	0	Tricyclic	0	Comorbid	0	Prevalence	0	Advantage	0	Peripheral	0	Dopamine	0	Physical examination	0	Referral	0	Attitude	0	Gene-related	0	Calcitonin	0	Beta-blocker	0	church	0	Temporal	0	Barbiturate	0	Lidocaine	0	Spectrum	0	Motivation	0	Menstrual migraine	0	Correlate	0	Outpatient	0	Injury	0	Lab test	0	Efficacy	0	Antihistamine	0	Lactation	0	Secondary headache	0	Trigeminal autonomic cephalalgia	0	Progression	0	Medication overuse	0	Inpatient treatment	0	Intracranial hypertension	0	Botulinum Toxin	0	Serotonin receptor	1	Mindfulness	0	Cranial nerve	0	Episodic migraine	0	Double vision	0	Vestibular migraine	0	surgical	0	Surgical treatment	0	5-HT	0	Ineffective	0	Eye symptom	0	Female hormone	0
0	Headache clinic	0	Assessment	0	Empower	0	Productivity	0	Caregiver	0	Nerve block	0	Accepting	0	Pain management	0	Mechanism	0	spouse	0	Co-morbid	0	Histamine	0	Tricyclic	0	Comorbid	0	Prevalence	0	Advantage	0	Peripheral	0	Dopamine	0	Physical examination	0	Referral	0	Attitude	0	Gene-related	0	Calcitonin	0	Beta-blocker	0	church	0	Temporal	0	Barbiturate	0	Lidocaine	0	Spectrum	0	Motivation	0	Menstrual migraine	0	Correlate	0	Outpatient	0	Injury	0	Lab test	0	Efficacy	0	Antihistamine	0	Lactation	0	Secondary headache	0	Trigeminal autonomic cephalalgia	0	Progression	0	Medication overuse	0	Inpatient treatment	0	Intracranial hypertension	0	Botulinum Toxin	0	Serotonin receptor	0	Mindfulness	0	Cranial nerve	0	Episodic migraine	0	Double vision	0	Vestibular migraine	0	surgical	0	Surgical treatment	0	5-HT	0	Ineffective	0	Eye symptom	0	Female hormone	0
0	Headache clinic	0	Assessment	0	Empower	0	Productivity	0	Caregiver	0	Nerve block	0	Accepting	0	Pain management	0	Mechanism	0	spouse	0	Co-morbid	0	Histamine	0	Tricyclic	0	Comorbid	0	Prevalence	0	Advantage	0	Peripheral	0	Dopamine	0	Physical examination	0	Referral	0	Attitude	0	Gene-related	0	Calcitonin	0	Beta-blocker	0	church	0	Temporal	0	Barbiturate	0	Lidocaine	0	Spectrum	0	Motivation	0	Menstrual migraine	0	Correlate	0	Outpatient	0	Injury	0	Lab test	0	Efficacy	0	Antihistamine	0	Lactation	0	Secondary headache	0	Trigeminal autonomic cephalalgia	0	Progression	0	Medication overuse	0	Inpatient treatment	0	Intracranial hypertension	0	Botulinum Toxin	0	Serotonin receptor	0	Mindfulness	0	Cranial nerve	0	Episodic migraine	0	Double vision	0	Vestibular migraine	0	surgical	0	Surgical treatment	0	5-HT	0	Ineffective	0	Eye symptom	0	Female hormone	0

P257a	P257b	P258a	P258b	P259a	P259b	P260a	P260b	P261a	P261b	P262a	P262b	P263a	P263b	P264a	P264b	P265a	P265b	P266a	P266b	P267a	P267b	P268a	P268b	P269a	P269b	P270a	P270b	P271a	P271b	P272a	P272b	P273a	P273b	P274a	P274b	P275a	P275b	P276a	P276b	P277a	P277b	P278a	P278b	P279a	P279b	P280a	P280b	P281a	P281b	P282a	P282b	P283a	P283b	P284a	P284b	P285a	P285b	P286a	P286b	P287a	P287b	P288a	P288b	P289a	P289b	P290a	P290b	P291a	P291b	P292a	P292b	P293a	P293b	P294a	P294b	P295a	P295b
Social worker	0	Folate	0	Septoplasty	1	nan	1	Eye pain	1	Angry	1	Emergency care	1	Food allergies	1	Cadasil	1	5HT	1	Anatomy	0	Counselor	0	Phenotype	0	Abnormalities	0	Comorbidity	0	Corticosteroid	0	Mood disorder	0	CT scan	0	Foramen ovale	0	Angiotensin Converting Enzyme	0	Prophylactic treatment	0	Subtype	0	Randomized controlled trial	0	Melatonin	0	Referred pain	0	Comorbidities	0	Behavioral therapy	0	Transmission	0	Father	0	Preemptive	0	Grandfather	0	Adherence	0	Clinician	0	Complicated migraine	0	Placebo effect	0	Consultation	0	Irk	0	Beta-Blockers	0	carer	0
Social worker	1	Folate	1	Septoplasty	0	nan	0	Eye pain	0	Angry	0	Emergency care	0	Food allergies	0	Cadasil	0	5HT	0	Anatomy	0	Counselor	0	Phenotype	0	Abnormalities	0	Comorbidity	0	Corticosteroid	0	Mood disorder	0	CT scan	0	Foramen ovale	0	Angiotensin Converting Enzyme	0	Prophylactic treatment	0	Subtype	0	Randomized controlled trial	0	Melatonin	0	Referred pain	0	Comorbidities	0	Behavioral therapy	0	Transmission	0	Father	0	Preemptive	0	Grandfather	0	Adherence	0	Clinician	0	Complicated migraine	0	Placebo effect	0	Consultation	0	Irk	0	Beta-Blockers	0	carer	0
Social worker	0	Folate	0	Septoplasty	0	nan	0	Eye pain	0	Angry	0	Emergency care	0	Food allergies	0	Cadasil	0	5HT	0	Anatomy	0	Counselor	0	Phenotype	0	Abnormalities	0	Comorbidity	0	Corticosteroid	0	Mood disorder	0	CT scan	0	Foramen ovale	0	Angiotensin Converting Enzyme	0	Prophylactic treatment	0	Subtype	0	Randomized controlled trial	0	Melatonin	0	Referred pain	0	Comorbidities	0	Behavioral therapy	0	Transmission	0	Father	0	Preemptive	0	Grandfather	0	Adherence	0	Clinician	0	Complicated migraine	0	Placebo effect	0	Consultation	0	Irk	0	Beta-Blockers	0	carer	0
Social worker	0	Folate	0	Septoplasty	0	nan	0	Eye pain	0	Angry	0	Emergency care	0	Food allergies	0	Cadasil	0	5HT	0	Anatomy	0	Counselor	0	Phenotype	0	Abnormalities	0	Comorbidity	0	Corticosteroid	0	Mood disorder	0	CT scan	0	Foramen ovale	0	Angiotensin Converting Enzyme	0	Prophylactic treatment	0	Subtype	0	Randomized controlled trial	0	Melatonin	0	Referred pain	0	Comorbidities	0	Behavioral therapy	0	Transmission	0	Father	0	Preemptive	0	Grandfather	0	Adherence	0	Clinician	0	Complicated migraine	0	Placebo effect	0	Consultation	0	Irk	0	Beta-Blockers	0	carer	0
Social worker	0	Folate	0	Septoplasty	0	nan	0	Eye pain	0	Angry	0	Emergency care	0	Food allergies	0	Cadasil	0	5HT	0	Anatomy	0	Counselor	0	Phenotype	0	Abnormalities	0	Comorbidity	0	Corticosteroid	0	Mood disorder	0	CT scan	0	Foramen ovale	0	Angiotensin Converting Enzyme	0	Prophylactic treatment	0	Subtype	0	Randomized controlled trial	0	Melatonin	0	Referred pain	0	Comorbidities	0	Behavioral therapy	0	Transmission	0	Father	0	Preemptive	0	Grandfather	0	Adherence	0	Clinician	0	Complicated migraine	0	Placebo effect	0	Consultation	0	Irk	0	Beta-Blockers	0	carer	0
Social worker	0	Folate	0	Septoplasty	0	nan	0	Eye pain	0	Angry	0	Emergency care	0	Food allergies	0	Cadasil	0	5HT	0	Anatomy	0	Counselor	0	Phenotype	0	Abnormalities	0	Comorbidity	0	Corticosteroid	0	Mood disorder	0	CT scan	0	Foramen ovale	0	Angiotensin Converting Enzyme	0	Prophylactic treatment	0	Subtype	0	Randomized controlled trial	0	Melatonin	0	Referred pain	0	Comorbidities	0	Behavioral therapy	0	Transmission	0	Father	0	Preemptive	0	Grandfather	0	Adherence	0	Clinician	0	Complicated migraine	0	Placebo effect	0	Consultation	0	Irk	0	Beta-Blockers	0	carer	0
Social worker	0	Folate	0	Septoplasty	0	nan	0	Eye pain	0	Angry	0	Emergency care	0	Food allergies	0	Cadasil	0	5HT	0	Anatomy	0	Counselor	0	Phenotype	0	Abnormalities	0	Comorbidity	0	Corticosteroid	0	Mood disorder	0	CT scan	0	Foramen ovale	0	Angiotensin Converting Enzyme	0	Prophylactic treatment	0	Subtype	0	Randomized controlled trial	0	Melatonin	0	Referred pain	0	Comorbidities	0	Behavioral therapy	0	Transmission	0	Father	0	Preemptive	0	Grandfather	0	Adherence	0	Clinician	0	Complicated migraine	0	Placebo effect	0	Consultation	0	Irk	0	Beta-Blockers	0	carer	0
Social worker	0	Folate	0	Septoplasty	0	nan	0	Eye pain	0	Angry	0	Emergency care	0	Food allergies	0	Cadasil	0	5HT	0	Anatomy	0	Counselor	0	Phenotype	0	Abnormalities	0	Comorbidity	0	Corticosteroid	0	Mood disorder	0	CT scan	0	Foramen ovale	0	Angiotensin Converting Enzyme	0	Prophylactic treatment	0	Subtype	0	Randomized controlled trial	0	Melatonin	0	Referred pain	0	Comorbidities	0	Behavioral therapy	0	Transmission	0	Father	0	Preemptive	0	Grandfather	0	Adherence	0	Clinician	0	Complicated migraine	0	Placebo effect	0	Consultation	0	Irk	0	Beta-Blockers	0	carer	1
Social worker	0	Folate	0	Septoplasty	0	nan	0	Eye pain	0	Angry	0	Emergency care	0	Food allergies	0	Cadasil	0	5HT	0	Anatomy	0	Counselor	0	Phenotype	0	Abnormalities	0	Comorbidity	0	Corticosteroid	0	Mood disorder	0	CT scan	0	Foramen ovale	0	Angiotensin Converting Enzyme	0	Prophylactic treatment	0	Subtype	0	Randomized controlled trial	0	Melatonin	0	Referred pain	0	Comorbidities	0	Behavioral therapy	0	Transmission	0	Father	0	Preemptive	0	Grandfather	0	Adherence	0	Clinician	0	Complicated migraine	0	Placebo effect	0	Consultation	0	Irk	0	Beta-Blockers	0	carer	0
Social worker	0	Folate	0	Septoplasty	0	nan	0	Eye pain	0	Angry	0	Emergency care	0	Food allergies	0	Cadasil	0	5HT	0	Anatomy	0	Counselor	0	Phenotype	0	Abnormalities	0	Comorbidity	0	Corticosteroid	0	Mood disorder	0	CT scan	0	Foramen ovale	0	Angiotensin Converting Enzyme	0	Prophylactic treatment	0	Subtype	0	Randomized controlled trial	0	Melatonin	0	Referred pain	0	Comorbidities	0	Behavioral therapy	0	Transmission	0	Father	0	Preemptive	0	Grandfather	0	Adherence	0	Clinician	0	Complicated migraine	0	Placebo effect	0	Consultation	0	Irk	0	Beta-Blockers	0	carer	0
Social worker	0	Folate	0	Septoplasty	0	nan	0	Eye pain	0	Angry	0	Emergency care	0	Food allergies	0	Cadasil	0	5HT	0	Anatomy	0	Counselor	0	Phenotype	0	Abnormalities	0	Comorbidity	0	Corticosteroid	0	Mood disorder	0	CT scan	0	Foramen ovale	0	Angiotensin Converting Enzyme	0	Prophylactic treatment	0	Subtype	0	Randomized controlled trial	0	Melatonin	0	Referred pain	0	Comorbidities	0	Behavioral therapy	0	Transmission	0	Father	0	Preemptive	0	Grandfather	0	Adherence	0	Clinician	0	Complicated migraine	0	Placebo effect	0	Consultation	0	Irk	0	Beta-Blockers	0	carer	0
Social worker	0	Folate	0	Septoplasty	0	nan	0	Eye pain	0	Angry	0	Emergency care	0	Food allergies	0	Cadasil	0	5HT	0	Anatomy	0	Counselor	0	Phenotype	0	Abnormalities	0	Comorbidity	0	Corticosteroid	0	Mood disorder	0	CT scan	0	Foramen ovale	0	Angiotensin Converting Enzyme	0	Prophylactic treatment	0	Subtype	0	Randomized controlled trial	0	Melatonin	0	Referred pain	0	Comorbidities	0	Behavioral therapy	0	Transmission	0	Father	0	Preemptive	0	Grandfather	0	Adherence	0	Clinician	0	Complicated migraine	0	Placebo effect	0	Consultation	0	Irk	0	Beta-Blockers	0	carer	0
Social worker	0	Folate	0	Septoplasty	0	nan	0	Eye pain	0	Angry	0	Emergency care	0	Food allergies	0	Cadasil	0	5HT	0	Anatomy	0	Counselor	0	Phenotype	0	Abnormalities	0	Comorbidity	0	Corticosteroid	0	Mood disorder	0	CT scan	0	Foramen ovale	0	Angiotensin Converting Enzyme	0	Prophylactic treatment	0	Subtype	0	Randomized controlled trial	0	Melatonin	0	Referred pain	0	Comorbidities	0	Behavioral therapy	0	Transmission	0	Father	0	Preemptive	0	Grandfather	0	Adherence	0	Clinician	0	Complicated migraine	0	Placebo effect	0	Consultation	0	Irk	0	Beta-Blockers	0	carer	0
Social worker	0	Folate	0	Septoplasty	0	nan	0	Eye pain	0	Angry	0	Emergency care	0	Food allergies	0	Cadasil	0	5HT	0	Anatomy	0	Counselor	0	Phenotype	0	Abnormalities	0	Comorbidity	0	Corticosteroid	0	Mood disorder	0	CT scan	0	Foramen ovale	0	Angiotensin Converting Enzyme	0	Prophylactic treatment	0	Subtype	0	Randomized controlled trial	0	Melatonin	0	Referred pain	0	Comorbidities	0	Behavioral therapy	0	Transmission	0	Father	0	Preemptive	0	Grandfather	0	Adherence	0	Clinician	0	Complicated migraine	0	Placebo effect	0	Consultation	0	Irk	0	Beta-Blockers	0	carer	0
Social worker	0	Folate	0	Septoplasty	0	nan	0	Eye pain	0	Angry	0	Emergency care	0	Food allergies	0	Cadasil	0	5HT	0	Anatomy	0	Counselor	0	Phenotype	0	Abnormalities	0	Comorbidity	0	Corticosteroid	0	Mood disorder	0	CT scan	0	Foramen ovale	0	Angiotensin Converting Enzyme	0	Prophylactic treatment	0	Subtype	0	Randomized controlled trial	0	Melatonin	0	Referred pain	0	Comorbidities	0	Behavioral therapy	0	Transmission	0	Father	0	Preemptive	0	Grandfather	0	Adherence	0	Clinician	0	Complicated migraine	0	Placebo effect	0	Consultation	0	Irk	0	Beta-Blockers	0	carer	0
Social worker	0	Folate	0	Septoplasty	0	nan	0	Eye pain	0	Angry	0	Emergency care	0	Food allergies	0	Cadasil	0	5HT	0	Anatomy	0	Counselor	0	Phenotype	0	Abnormalities	0	Comorbidity	0	Corticosteroid	0	Mood disorder	0	CT scan	0	Foramen ovale	0	Angiotensin Converting Enzyme	0	Prophylactic treatment	0	Subtype	0	Randomized controlled trial	0	Melatonin	0	Referred pain	0	Comorbidities	0	Behavioral therapy	0	Transmission	0	Father	0	Preemptive	0	Grandfather	0	Adherence	0	Clinician	0	Complicated migraine	0	Placebo effect	0	Consultation	0	Irk	0	Beta-Blockers	0	carer	0
Social worker	0	Folate	0	Septoplasty	0	nan	0	Eye pain	0	Angry	0	Emergency care	0	Food allergies	0	Cadasil	0	5HT	0	Anatomy	0	Counselor	0	Phenotype	0	Abnormalities	0	Comorbidity	0	Corticosteroid	0	Mood disorder	0	CT scan	0	Foramen ovale	0	Angiotensin Converting Enzyme	0	Prophylactic treatment	0	Subtype	0	Randomized controlled trial	0	Melatonin	0	Referred pain	0	Comorbidities	0	Behavioral therapy	0	Transmission	0	Father	0	Preemptive	0	Grandfather	0	Adherence	0	Clinician	0	Complicated migraine	0	Placebo effect	0	Consultation	0	Irk	0	Beta-Blockers	0	carer	0
Social worker	0	Folate	0	Septoplasty	0	nan	0	Eye pain	0	Angry	0	Emergency care	0	Food allergies	0	Cadasil	0	5HT	0	Anatomy	0	Counselor	0	Phenotype	0	Abnormalities	0	Comorbidity	0	Corticosteroid	0	Mood disorder	0	CT scan	0	Foramen ovale	0	Angiotensin Converting Enzyme	0	Prophylactic treatment	0	Subtype	0	Randomized controlled trial	0	Melatonin	0	Referred pain	0	Comorbidities	0	Behavioral therapy	0	Transmission	0	Father	0	Preemptive	0	Grandfather	0	Adherence	0	Clinician	0	Complicated migraine	0	Placebo effect	0	Consultation	0	Irk	0	Beta-Blockers	0	carer	0
Social worker	0	Folate	0	Septoplasty	0	nan	0	Eye pain	0	Angry	0	Emergency care	0	Food allergies	0	Cadasil	0	5HT	0	Anatomy	0	Counselor	0	Phenotype	0	Abnormalities	0	Comorbidity	0	Corticosteroid	0	Mood disorder	0	CT scan	0	Foramen ovale	0	Angiotensin Converting Enzyme	0	Prophylactic treatment	0	Subtype	0	Randomized controlled trial	0	Melatonin	0	Referred pain	0	Comorbidities	0	Behavioral therapy	0	Transmission	0	Father	0	Preemptive	0	Grandfather	0	Adherence	0	Clinician	0	Complicated migraine	0	Placebo effect	0	Consultation	0	Irk	0	Beta-Blockers	0	carer	0
Social worker	0	Folate	0	Septoplasty	0	nan	0	Eye pain	0	Angry	0	Emergency care	0	Food allergies	0	Cadasil	0	5HT	0	Anatomy	0	Counselor	0	Phenotype	0	Abnormalities	0	Comorbidity	0	Corticosteroid	0	Mood disorder	0	CT scan	0	Foramen ovale	0	Angiotensin Converting Enzyme	0	Prophylactic treatment	0	Subtype	0	Randomized controlled trial	0	Melatonin	0	Referred pain	0	Comorbidities	0	Behavioral therapy	0	Transmission	0	Father	0	Preemptive	0	Grandfather	0	Adherence	0	Clinician	0	Complicated migraine	0	Placebo effect	0	Consultation	0	Irk	0	Beta-Blockers	0	carer	0
Social worker	0	Folate	0	Septoplasty	0	nan	0	Eye pain	0	Angry	0	Emergency care	0	Food allergies	0	Cadasil	0	5HT	0	Anatomy	0	Counselor	0	Phenotype	0	Abnormalities	0	Comorbidity	0	Corticosteroid	0	Mood disorder	0	CT scan	0	Foramen ovale	0	Angiotensin Converting Enzyme	0	Prophylactic treatment	0	Subtype	0	Randomized controlled trial	0	Melatonin	0	Referred pain	0	Comorbidities	0	Behavioral therapy	0	Transmission	0	Father	0	Preemptive	0	Grandfather	0	Adherence	0	Clinician	0	Complicated migraine	0	Placebo effect	0	Consultation	0	Irk	0	Beta-Blockers	0	carer	0
Social worker	0	Folate	0	Septoplasty	0	nan	0	Eye pain	0	Angry	0	Emergency care	0	Food allergies	0	Cadasil	0	5HT	0	Anatomy	0	Counselor	0	Phenotype	0	Abnormalities	0	Comorbidity	0	Corticosteroid	0	Mood disorder	0	CT scan	0	Foramen ovale	0	Angiotensin Converting Enzyme	0	Prophylactic treatment	0	Subtype	0	Randomized controlled trial	0	Melatonin	0	Referred pain	0	Comorbidities	0	Behavioral therapy	0	Transmission	0	Father	0	Preemptive	0	Grandfather	0	Adherence	0	Clinician	0	Complicated migraine	0	Placebo effect	0	Consultation	0	Irk	0	Beta-Blockers	0	carer	0
Social worker	0	Folate	0	Septoplasty	0	nan	0	Eye pain	0	Angry	0	Emergency care	0	Food allergies	0	Cadasil	0	5HT	0	Anatomy	0	Counselor	0	Phenotype	0	Abnormalities	0	Comorbidity	0	Corticosteroid	0	Mood disorder	0	CT scan	0	Foramen ovale	0	Angiotensin Converting Enzyme	0	Prophylactic treatment	0	Subtype	0	Randomized controlled trial	0	Melatonin	0	Referred pain	0	Comorbidities	0	Behavioral therapy	0	Transmission	0	Father	0	Preemptive	0	Grandfather	0	Adherence	0	Clinician	0	Complicated migraine	0	Placebo effect	0	Consultation	0	Irk	0	Beta-Blockers	0	carer	0
Social worker	0	Folate	0	Septoplasty	0	nan	0	Eye pain	0	Angry	0	Emergency care	0	Food allergies	0	Cadasil	0	5HT	0	Anatomy	0	Counselor	0	Phenotype	0	Abnormalities	0	Comorbidity	0	Corticosteroid	0	Mood disorder	0	CT scan	0	Foramen ovale	0	Angiotensin Converting Enzyme	0	Prophylactic treatment	0	Subtype	0	Randomized controlled trial	0	Melatonin	0	Referred pain	0	Comorbidities	0	Behavioral therapy	0	Transmission	0	Father	0	Preemptive	0	Grandfather	0	Adherence	0	Clinician	0	Complicated migraine	0	Placebo effect	0	Consultation	0	Irk	0	Beta-Blockers	0	carer	0
Social worker	0	Folate	0	Septoplasty	0	nan	0	Eye pain	0	Angry	0	Emergency care	0	Food allergies	0	Cadasil	0	5HT	0	Anatomy	0	Counselor	0	Phenotype	0	Abnormalities	0	Comorbidity	0	Corticosteroid	0	Mood disorder	0	CT scan	0	Foramen ovale	0	Angiotensin Converting Enzyme	0	Prophylactic treatment	0	Subtype	0	Randomized controlled trial	0	Melatonin	0	Referred pain	0	Comorbidities	0	Behavioral therapy	0	Transmission	0	Father	0	Preemptive	0	Grandfather	0	Adherence	0	Clinician	0	Complicated migraine	0	Placebo effect	0	Consultation	0	Irk	0	Beta-Blockers	0	carer	0
Social worker	0	Folate	0	Septoplasty	0	nan	0	Eye pain	0	Angry	0	Emergency care	0	Food allergies	0	Cadasil	0	5HT	0	Anatomy	0	Counselor	0	Phenotype	0	Abnormalities	0	Comorbidity	0	Corticosteroid	0	Mood disorder	0	CT scan	0	Foramen ovale	0	Angiotensin Converting Enzyme	0	Prophylactic treatment	0	Subtype	0	Randomized controlled trial	0	Melatonin	0	Referred pain	0	Comorbidities	0	Behavioral therapy	0	Transmission	0	Father	0	Preemptive	0	Grandfather	0	Adherence	0	Clinician	0	Complicated migraine	0	Placebo effect	0	Consultation	0	Irk	0	Beta-Blockers	0	carer	0
Social worker	0	Folate	0	Septoplasty	0	nan	0	Eye pain	0	Angry	0	Emergency care	0	Food allergies	0	Cadasil	0	5HT	0	Anatomy	0	Counselor	0	Phenotype	0	Abnormalities	0	Comorbidity	0	Corticosteroid	0	Mood disorder	0	CT scan	0	Foramen ovale	0	Angiotensin Converting Enzyme	0	Prophylactic treatment	0	Subtype	0	Randomized controlled trial	0	Melatonin	0	Referred pain	0	Comorbidities	0	Behavioral therapy	0	Transmission	0	Father	0	Preemptive	0	Grandfather	0	Adherence	0	Clinician	0	Complicated migraine	0	Placebo effect	0	Consultation	0	Irk	0	Beta-Blockers	1	carer	0
Social worker	0	Folate	0	Septoplasty	0	nan	0	Eye pain	0	Angry	0	Emergency care	0	Food allergies	0	Cadasil	0	5HT	0	Anatomy	0	Counselor	0	Phenotype	0	Abnormalities	0	Comorbidity	0	Corticosteroid	0	Mood disorder	0	CT scan	0	Foramen ovale	0	Angiotensin Converting Enzyme	0	Prophylactic treatment	0	Subtype	0	Randomized controlled trial	0	Melatonin	0	Referred pain	0	Comorbidities	0	Behavioral therapy	0	Transmission	0	Father	0	Preemptive	0	Grandfather	0	Adherence	0	Clinician	0	Complicated migraine	0	Placebo effect	0	Consultation	0	Irk	0	Beta-Blockers	0	carer	0
Social worker	0	Folate	0	Septoplasty	0	nan	0	Eye pain	0	Angry	0	Emergency care	0	Food allergies	0	Cadasil	0	5HT	0	Anatomy	0	Counselor	0	Phenotype	0	Abnormalities	0	Comorbidity	0	Corticosteroid	0	Mood disorder	0	CT scan	0	Foramen ovale	0	Angiotensin Converting Enzyme	0	Prophylactic treatment	0	Subtype	0	Randomized controlled trial	0	Melatonin	0	Referred pain	0	Comorbidities	0	Behavioral therapy	0	Transmission	0	Father	0	Preemptive	0	Grandfather	0	Adherence	0	Clinician	0	Complicated migraine	0	Placebo effect	0	Consultation	0	Irk	0	Beta-Blockers	0	carer	0
Social worker	0	Folate	0	Septoplasty	0	nan	0	Eye pain	0	Angry	0	Emergency care	0	Food allergies	0	Cadasil	0	5HT	0	Anatomy	0	Counselor	0	Phenotype	0	Abnormalities	0	Comorbidity	0	Corticosteroid	0	Mood disorder	0	CT scan	0	Foramen ovale	0	Angiotensin Converting Enzyme	0	Prophylactic treatment	0	Subtype	0	Randomized controlled trial	0	Melatonin	0	Referred pain	0	Comorbidities	0	Behavioral therapy	0	Transmission	0	Father	0	Preemptive	0	Grandfather	0	Adherence	0	Clinician	0	Complicated migraine	0	Placebo effect	0	Consultation	0	Irk	0	Beta-Blockers	0	carer	0
Social worker	0	Folate	0	Septoplasty	0	nan	0	Eye pain	0	Angry	0	Emergency care	0	Food allergies	0	Cadasil	0	5HT	0	Anatomy	0	Counselor	0	Phenotype	0	Abnormalities	0	Comorbidity	0	Corticosteroid	0	Mood disorder	0	CT scan	0	Foramen ovale	0	Angiotensin Converting Enzyme	0	Prophylactic treatment	0	Subtype	0	Randomized controlled trial	0	Melatonin	0	Referred pain	0	Comorbidities	0	Behavioral therapy	0	Transmission	0	Father	0	Preemptive	0	Grandfather	0	Adherence	0	Clinician	0	Complicated migraine	0	Placebo effect	0	Consultation	0	Irk	0	Beta-Blockers	0	carer	0
Social worker	0	Folate	0	Septoplasty	0	nan	0	Eye pain	0	Angry	0	Emergency care	0	Food allergies	0	Cadasil	0	5HT	0	Anatomy	0	Counselor	0	Phenotype	0	Abnormalities	0	Comorbidity	0	Corticosteroid	0	Mood disorder	0	CT scan	0	Foramen ovale	0	Angiotensin Converting Enzyme	0	Prophylactic treatment	0	Subtype	0	Randomized controlled trial	0	Melatonin	0	Referred pain	0	Comorbidities	0	Behavioral therapy	0	Transmission	0	Father	0	Preemptive	0	Grandfather	0	Adherence	0	Clinician	0	Complicated migraine	0	Placebo effect	0	Consultation	0	Irk	0	Beta-Blockers	0	carer	0
Social worker	0	Folate	0	Septoplasty	0	nan	0	Eye pain	0	Angry	0	Emergency care	0	Food allergies	0	Cadasil	0	5HT	0	Anatomy	0	Counselor	0	Phenotype	0	Abnormalities	0	Comorbidity	0	Corticosteroid	0	Mood disorder	0	CT scan	0	Foramen ovale	0	Angiotensin Converting Enzyme	0	Prophylactic treatment	0	Subtype	0	Randomized controlled trial	0	Melatonin	0	Referred pain	0	Comorbidities	0	Behavioral therapy	0	Transmission	0	Father	0	Preemptive	0	Grandfather	0	Adherence	0	Clinician	0	Complicated migraine	0	Placebo effect	0	Consultation	0	Irk	0	Beta-Blockers	0	carer	0
Social worker	0	Folate	0	Septoplasty	0	nan	0	Eye pain	0	Angry	0	Emergency care	0	Food allergies	0	Cadasil	0	5HT	0	Anatomy	0	Counselor	0	Phenotype	0	Abnormalities	0	Comorbidity	0	Corticosteroid	0	Mood disorder	0	CT scan	0	Foramen ovale	0	Angiotensin Converting Enzyme	0	Prophylactic treatment	0	Subtype	0	Randomized controlled trial	0	Melatonin	0	Referred pain	0	Comorbidities	0	Behavioral therapy	0	Transmission	0	Father	0	Preemptive	0	Grandfather	0	Adherence	0	Clinician	0	Complicated migraine	0	Placebo effect	0	Consultation	0	Irk	0	Beta-Blockers	0	carer	0
Social worker	0	Folate	0	Septoplasty	0	nan	0	Eye pain	0	Angry	0	Emergency care	0	Food allergies	0	Cadasil	0	5HT	0	Anatomy	0	Counselor	0	Phenotype	0	Abnormalities	0	Comorbidity	0	Corticosteroid	0	Mood disorder	0	CT scan	0	Foramen ovale	0	Angiotensin Converting Enzyme	0	Prophylactic treatment	0	Subtype	0	Randomized controlled trial	0	Melatonin	0	Referred pain	0	Comorbidities	0	Behavioral therapy	0	Transmission	0	Father	0	Preemptive	0	Grandfather	0	Adherence	0	Clinician	0	Complicated migraine	0	Placebo effect	0	Consultation	0	Irk	1	Beta-Blockers	0	carer	0
Social worker	0	Folate	0	Septoplasty	0	nan	0	Eye pain	0	Angry	0	Emergency care	0	Food allergies	0	Cadasil	0	5HT	0	Anatomy	0	Counselor	0	Phenotype	0	Abnormalities	0	Comorbidity	0	Corticosteroid	0	Mood disorder	0	CT scan	0	Foramen ovale	0	Angiotensin Converting Enzyme	0	Prophylactic treatment	0	Subtype	0	Randomized controlled trial	0	Melatonin	0	Referred pain	0	Comorbidities	0	Behavioral therapy	0	Transmission	0	Father	0	Preemptive	0	Grandfather	0	Adherence	0	Clinician	0	Complicated migraine	0	Placebo effect	0	Consultation	0	Irk	0	Beta-Blockers	0	carer	0
Social worker	0	Folate	0	Septoplasty	0	nan	0	Eye pain	0	Angry	0	Emergency care	0	Food allergies	0	Cadasil	0	5HT	0	Anatomy	0	Counselor	0	Phenotype	0	Abnormalities	0	Comorbidity	0	Corticosteroid	0	Mood disorder	0	CT scan	0	Foramen ovale	0	Angiotensin Converting Enzyme	0	Prophylactic treatment	0	Subtype	0	Randomized controlled trial	0	Melatonin	0	Referred pain	0	Comorbidities	0	Behavioral therapy	0	Transmission	0	Father	0	Preemptive	0	Grandfather	0	Adherence	0	Clinician	0	Complicated migraine	0	Placebo effect	0	Consultation	0	Irk	0	Beta-Blockers	0	carer	0
Social worker	0	Folate	0	Septoplasty	1	nan	1	Eye pain	1	Angry	1	Emergency care	1	Food allergies	1	Cadasil	1	5HT	1	Anatomy	0	Counselor	0	Phenotype	0	Abnormalities	0	Comorbidity	0	Corticosteroid	0	Mood disorder	0	CT scan	0	Foramen ovale	0	Angiotensin Converting Enzyme	0	Prophylactic treatment	0	Subtype	0	Randomized controlled trial	0	Melatonin	0	Referred pain	0	Comorbidities	0	Behavioral therapy	0	Transmission	0	Father	0	Preemptive	0	Grandfather	0	Adherence	0	Clinician	0	Complicated migraine	0	Placebo effect	0	Consultation	0	Irk	0	Beta-Blockers	0	carer	0
Social worker	0	Folate	0	Septoplasty	0	nan	0	Eye pain	0	Angry	0	Emergency care	0	Food allergies	0	Cadasil	0	5HT	0	Anatomy	0	Counselor	0	Phenotype	0	Abnormalities	0	Comorbidity	0	Corticosteroid	0	Mood disorder	0	CT scan	0	Foramen ovale	0	Angiotensin Converting Enzyme	0	Prophylactic treatment	0	Subtype	0	Randomized controlled trial	0	Melatonin	0	Referred pain	0	Comorbidities	0	Behavioral therapy	0	Transmission	0	Father	0	Preemptive	0	Grandfather	0	Adherence	0	Clinician	0	Complicated migraine	0	Placebo effect	0	Consultation	0	Irk	0	Beta-Blockers	0	carer	0
Social worker	0	Folate	0	Septoplasty	0	nan	0	Eye pain	0	Angry	0	Emergency care	0	Food allergies	0	Cadasil	0	5HT	0	Anatomy	0	Counselor	0	Phenotype	0	Abnormalities	0	Comorbidity	0	Corticosteroid	0	Mood disorder	0	CT scan	0	Foramen ovale	0	Angiotensin Converting Enzyme	0	Prophylactic treatment	0	Subtype	0	Randomized controlled trial	0	Melatonin	0	Referred pain	0	Comorbidities	0	Behavioral therapy	0	Transmission	0	Father	0	Preemptive	0	Grandfather	0	Adherence	0	Clinician	0	Complicated migraine	0	Placebo effect	0	Consultation	0	Irk	0	Beta-Blockers	0	carer	0
Social worker	0	Folate	0	Septoplasty	0	nan	0	Eye pain	0	Angry	0	Emergency care	0	Food allergies	0	Cadasil	0	5HT	0	Anatomy	0	Counselor	0	Phenotype	0	Abnormalities	0	Comorbidity	0	Corticosteroid	0	Mood disorder	0	CT scan	0	Foramen ovale	0	Angiotensin Converting Enzyme	0	Prophylactic treatment	0	Subtype	0	Randomized controlled trial	0	Melatonin	0	Referred pain	0	Comorbidities	0	Behavioral therapy	0	Transmission	0	Father	0	Preemptive	0	Grandfather	0	Adherence	0	Clinician	0	Complicated migraine	0	Placebo effect	0	Consultation	1	Irk	0	Beta-Blockers	0	carer	0
Social worker	0	Folate	0	Septoplasty	0	nan	0	Eye pain	0	Angry	0	Emergency care	0	Food allergies	0	Cadasil	0	5HT	0	Anatomy	0	Counselor	0	Phenotype	0	Abnormalities	0	Comorbidity	0	Corticosteroid	0	Mood disorder	0	CT scan	0	Foramen ovale	0	Angiotensin Converting Enzyme	0	Prophylactic treatment	0	Subtype	0	Randomized controlled trial	0	Melatonin	0	Referred pain	0	Comorbidities	0	Behavioral therapy	0	Transmission	0	Father	0	Preemptive	0	Grandfather	0	Adherence	0	Clinician	0	Complicated migraine	0	Placebo effect	0	Consultation	0	Irk	0	Beta-Blockers	0	carer	0
Social worker	0	Folate	0	Septoplasty	0	nan	0	Eye pain	0	Angry	0	Emergency care	0	Food allergies	0	Cadasil	0	5HT	0	Anatomy	0	Counselor	0	Phenotype	0	Abnormalities	0	Comorbidity	0	Corticosteroid	0	Mood disorder	0	CT scan	0	Foramen ovale	0	Angiotensin Converting Enzyme	0	Prophylactic treatment	0	Subtype	0	Randomized controlled trial	0	Melatonin	0	Referred pain	0	Comorbidities	0	Behavioral therapy	0	Transmission	0	Father	0	Preemptive	0	Grandfather	0	Adherence	0	Clinician	0	Complicated migraine	0	Placebo effect	0	Consultation	0	Irk	0	Beta-Blockers	0	carer	0
Social worker	0	Folate	0	Septoplasty	0	nan	0	Eye pain	0	Angry	0	Emergency care	0	Food allergies	0	Cadasil	0	5HT	0	Anatomy	0	Counselor	0	Phenotype	0	Abnormalities	0	Comorbidity	0	Corticosteroid	0	Mood disorder	0	CT scan	0	Foramen ovale	0	Angiotensin Converting Enzyme	0	Prophylactic treatment	0	Subtype	0	Randomized controlled trial	0	Melatonin	0	Referred pain	0	Comorbidities	0	Behavioral therapy	0	Transmission	0	Father	0	Preemptive	0	Grandfather	0	Adherence	0	Clinician	0	Complicated migraine	0	Placebo effect	0	Consultation	0	Irk	0	Beta-Blockers	0	carer	0
Social worker	0	Folate	0	Septoplasty	0	nan	0	Eye pain	0	Angry	0	Emergency care	0	Food allergies	0	Cadasil	0	5HT	0	Anatomy	0	Counselor	0	Phenotype	0	Abnormalities	0	Comorbidity	0	Corticosteroid	0	Mood disorder	0	CT scan	0	Foramen ovale	0	Angiotensin Converting Enzyme	0	Prophylactic treatment	0	Subtype	0	Randomized controlled trial	0	Melatonin	0	Referred pain	0	Comorbidities	0	Behavioral therapy	0	Transmission	0	Father	0	Preemptive	0	Grandfather	0	Adherence	0	Clinician	0	Complicated migraine	0	Placebo effect	1	Consultation	0	Irk	0	Beta-Blockers	0	carer	0
Social worker	0	Folate	0	Septoplasty	0	nan	0	Eye pain	0	Angry	0	Emergency care	0	Food allergies	0	Cadasil	0	5HT	0	Anatomy	0	Counselor	0	Phenotype	0	Abnormalities	0	Comorbidity	0	Corticosteroid	0	Mood disorder	0	CT scan	0	Foramen ovale	0	Angiotensin Converting Enzyme	0	Prophylactic treatment	0	Subtype	0	Randomized controlled trial	0	Melatonin	0	Referred pain	0	Comorbidities	0	Behavioral therapy	0	Transmission	0	Father	0	Preemptive	0	Grandfather	0	Adherence	0	Clinician	0	Complicated migraine	0	Placebo effect	0	Consultation	0	Irk	0	Beta-Blockers	0	carer	0
Social worker	1	Folate	1	Septoplasty	0	nan	0	Eye pain	0	Angry	0	Emergency care	0	Food allergies	0	Cadasil	0	5HT	0	Anatomy	0	Counselor	0	Phenotype	0	Abnormalities	0	Comorbidity	0	Corticosteroid	0	Mood disorder	0	CT scan	0	Foramen ovale	0	Angiotensin Converting Enzyme	0	Prophylactic treatment	0	Subtype	0	Randomized controlled trial	0	Melatonin	0	Referred pain	0	Comorbidities	0	Behavioral therapy	0	Transmission	0	Father	0	Preemptive	0	Grandfather	0	Adherence	0	Clinician	0	Complicated migraine	0	Placebo effect	0	Consultation	0	Irk	0	Beta-Blockers	0	carer	0
Social worker	0	Folate	0	Septoplasty	0	nan	0	Eye pain	0	Angry	0	Emergency care	0	Food allergies	0	Cadasil	0	5HT	0	Anatomy	0	Counselor	0	Phenotype	0	Abnormalities	0	Comorbidity	0	Corticosteroid	0	Mood disorder	0	CT scan	0	Foramen ovale	0	Angiotensin Converting Enzyme	0	Prophylactic treatment	0	Subtype	0	Randomized controlled trial	0	Melatonin	0	Referred pain	0	Comorbidities	0	Behavioral therapy	0	Transmission	0	Father	0	Preemptive	0	Grandfather	0	Adherence	0	Clinician	0	Complicated migraine	0	Placebo effect	0	Consultation	0	Irk	0	Beta-Blockers	0	carer	0
Social worker	0	Folate	0	Septoplasty	0	nan	0	Eye pain	0	Angry	0	Emergency care	0	Food allergies	0	Cadasil	0	5HT	0	Anatomy	0	Counselor	0	Phenotype	0	Abnormalities	0	Comorbidity	0	Corticosteroid	0	Mood disorder	0	CT scan	0	Foramen ovale	0	Angiotensin Converting Enzyme	0	Prophylactic treatment	0	Subtype	0	Randomized controlled trial	0	Melatonin	0	Referred pain	0	Comorbidities	0	Behavioral therapy	0	Transmission	0	Father	0	Preemptive	0	Grandfather	0	Adherence	0	Clinician	0	Complicated migraine	0	Placebo effect	0	Consultation	0	Irk	0	Beta-Blockers	0	carer	0
Social worker	0	Folate	0	Septoplasty	0	nan	0	Eye pain	0	Angry	0	Emergency care	0	Food allergies	0	Cadasil	0	5HT	0	Anatomy	0	Counselor	0	Phenotype	0	Abnormalities	0	Comorbidity	0	Corticosteroid	0	Mood disorder	0	CT scan	0	Foramen ovale	0	Angiotensin Converting Enzyme	0	Prophylactic treatment	0	Subtype	0	Randomized controlled trial	0	Melatonin	0	Referred pain	0	Comorbidities	0	Behavioral therapy	0	Transmission	0	Father	0	Preemptive	0	Grandfather	0	Adherence	0	Clinician	0	Complicated migraine	0	Placebo effect	0	Consultation	0	Irk	0	Beta-Blockers	0	carer	0
Social worker	0	Folate	0	Septoplasty	0	nan	0	Eye pain	0	Angry	0	Emergency care	0	Food allergies	0	Cadasil	0	5HT	0	Anatomy	0	Counselor	0	Phenotype	0	Abnormalities	0	Comorbidity	0	Corticosteroid	0	Mood disorder	0	CT scan	0	Foramen ovale	0	Angiotensin Converting Enzyme	0	Prophylactic treatment	0	Subtype	0	Randomized controlled trial	0	Melatonin	0	Referred pain	0	Comorbidities	0	Behavioral therapy	0	Transmission	0	Father	0	Preemptive	0	Grandfather	0	Adherence	0	Clinician	0	Complicated migraine	0	Placebo effect	0	Consultation	0	Irk	0	Beta-Blockers	0	carer	0
Social worker	0	Folate	0	Septoplasty	0	nan	0	Eye pain	0	Angry	0	Emergency care	0	Food allergies	0	Cadasil	0	5HT	0	Anatomy	0	Counselor	0	Phenotype	0	Abnormalities	0	Comorbidity	0	Corticosteroid	0	Mood disorder	0	CT scan	0	Foramen ovale	0	Angiotensin Converting Enzyme	0	Prophylactic treatment	0	Subtype	0	Randomized controlled trial	0	Melatonin	0	Referred pain	0	Comorbidities	0	Behavioral therapy	0	Transmission	0	Father	0	Preemptive	0	Grandfather	0	Adherence	0	Clinician	0	Complicated migraine	0	Placebo effect	0	Consultation	0	Irk	0	Beta-Blockers	0	carer	0
Social worker	0	Folate	0	Septoplasty	0	nan	0	Eye pain	0	Angry	0	Emergency care	0	Food allergies	0	Cadasil	0	5HT	0	Anatomy	0	Counselor	0	Phenotype	0	Abnormalities	0	Comorbidity	0	Corticosteroid	0	Mood disorder	0	CT scan	0	Foramen ovale	0	Angiotensin Converting Enzyme	0	Prophylactic treatment	0	Subtype	0	Randomized controlled trial	0	Melatonin	0	Referred pain	0	Comorbidities	0	Behavioral therapy	0	Transmission	0	Father	0	Preemptive	0	Grandfather	0	Adherence	0	Clinician	0	Complicated migraine	0	Placebo effect	0	Consultation	0	Irk	0	Beta-Blockers	0	carer	0
Social worker	0	Folate	0	Septoplasty	0	nan	0	Eye pain	0	Angry	0	Emergency care	0	Food allergies	0	Cadasil	0	5HT	0	Anatomy	0	Counselor	0	Phenotype	0	Abnormalities	0	Comorbidity	0	Corticosteroid	0	Mood disorder	0	CT scan	0	Foramen ovale	0	Angiotensin Converting Enzyme	0	Prophylactic treatment	0	Subtype	0	Randomized controlled trial	0	Melatonin	0	Referred pain	0	Comorbidities	0	Behavioral therapy	0	Transmission	0	Father	0	Preemptive	0	Grandfather	0	Adherence	0	Clinician	0	Complicated migraine	1	Placebo effect	0	Consultation	0	Irk	0	Beta-Blockers	0	carer	0
Social worker	0	Folate	0	Septoplasty	0	nan	0	Eye pain	0	Angry	0	Emergency care	0	Food allergies	0	Cadasil	0	5HT	0	Anatomy	0	Counselor	0	Phenotype	0	Abnormalities	0	Comorbidity	0	Corticosteroid	0	Mood disorder	0	CT scan	0	Foramen ovale	0	Angiotensin Converting Enzyme	0	Prophylactic treatment	0	Subtype	0	Randomized controlled trial	0	Melatonin	0	Referred pain	0	Comorbidities	0	Behavioral therapy	0	Transmission	0	Father	0	Preemptive	0	Grandfather	0	Adherence	0	Clinician	0	Complicated migraine	0	Placebo effect	0	Consultation	0	Irk	0	Beta-Blockers	0	carer	0
Social worker	0	Folate	0	Septoplasty	0	nan	0	Eye pain	0	Angry	0	Emergency care	0	Food allergies	0	Cadasil	0	5HT	0	Anatomy	0	Counselor	0	Phenotype	0	Abnormalities	0	Comorbidity	0	Corticosteroid	0	Mood disorder	0	CT scan	0	Foramen ovale	0	Angiotensin Converting Enzyme	0	Prophylactic treatment	0	Subtype	0	Randomized controlled trial	0	Melatonin	0	Referred pain	0	Comorbidities	0	Behavioral therapy	0	Transmission	0	Father	0	Preemptive	0	Grandfather	0	Adherence	0	Clinician	0	Complicated migraine	0	Placebo effect	0	Consultation	0	Irk	0	Beta-Blockers	0	carer	0
Social worker	0	Folate	0	Septoplasty	0	nan	0	Eye pain	0	Angry	0	Emergency care	0	Food allergies	0	Cadasil	0	5HT	0	Anatomy	0	Counselor	0	Phenotype	0	Abnormalities	0	Comorbidity	0	Corticosteroid	0	Mood disorder	0	CT scan	0	Foramen ovale	0	Angiotensin Converting Enzyme	0	Prophylactic treatment	0	Subtype	0	Randomized controlled trial	0	Melatonin	0	Referred pain	0	Comorbidities	0	Behavioral therapy	0	Transmission	0	Father	0	Preemptive	0	Grandfather	0	Adherence	0	Clinician	0	Complicated migraine	0	Placebo effect	0	Consultation	0	Irk	0	Beta-Blockers	0	carer	0
Social worker	0	Folate	0	Septoplasty	0	nan	0	Eye pain	0	Angry	0	Emergency care	0	Food allergies	0	Cadasil	0	5HT	0	Anatomy	0	Counselor	0	Phenotype	0	Abnormalities	0	Comorbidity	0	Corticosteroid	0	Mood disorder	0	CT scan	0	Foramen ovale	0	Angiotensin Converting Enzyme	0	Prophylactic treatment	0	Subtype	0	Randomized controlled trial	0	Melatonin	0	Referred pain	0	Comorbidities	0	Behavioral therapy	0	Transmission	0	Father	0	Preemptive	0	Grandfather	0	Adherence	0	Clinician	1	Complicated migraine	0	Placebo effect	0	Consultation	0	Irk	0	Beta-Blockers	0	carer	0
Social worker	0	Folate	0	Septoplasty	0	nan	0	Eye pain	0	Angry	0	Emergency care	0	Food allergies	0	Cadasil	0	5HT	0	Anatomy	0	Counselor	0	Phenotype	0	Abnormalities	0	Comorbidity	0	Corticosteroid	0	Mood disorder	0	CT scan	0	Foramen ovale	0	Angiotensin Converting Enzyme	0	Prophylactic treatment	0	Subtype	0	Randomized controlled trial	0	Melatonin	0	Referred pain	0	Comorbidities	0	Behavioral therapy	0	Transmission	0	Father	0	Preemptive	0	Grandfather	0	Adherence	0	Clinician	0	Complicated migraine	0	Placebo effect	0	Consultation	0	Irk	0	Beta-Blockers	0	carer	0
Social worker	0	Folate	0	Septoplasty	0	nan	0	Eye pain	0	Angry	0	Emergency care	0	Food allergies	0	Cadasil	0	5HT	0	Anatomy	0	Counselor	0	Phenotype	0	Abnormalities	0	Comorbidity	0	Corticosteroid	0	Mood disorder	0	CT scan	0	Foramen ovale	0	Angiotensin Converting Enzyme	0	Prophylactic treatment	0	Subtype	0	Randomized controlled trial	0	Melatonin	0	Referred pain	0	Comorbidities	0	Behavioral therapy	0	Transmission	0	Father	0	Preemptive	0	Grandfather	0	Adherence	0	Clinician	0	Complicated migraine	0	Placebo effect	0	Consultation	0	Irk	0	Beta-Blockers	0	carer	0
Social worker	0	Folate	0	Septoplasty	0	nan	0	Eye pain	0	Angry	0	Emergency care	0	Food allergies	0	Cadasil	0	5HT	0	Anatomy	0	Counselor	0	Phenotype	0	Abnormalities	0	Comorbidity	0	Corticosteroid	0	Mood disorder	0	CT scan	0	Foramen ovale	0	Angiotensin Converting Enzyme	0	Prophylactic treatment	0	Subtype	0	Randomized controlled trial	0	Melatonin	0	Referred pain	0	Comorbidities	0	Behavioral therapy	0	Transmission	0	Father	0	Preemptive	0	Grandfather	0	Adherence	0	Clinician	0	Complicated migraine	0	Placebo effect	0	Consultation	0	Irk	0	Beta-Blockers	0	carer	0
Social worker	0	Folate	0	Septoplasty	0	nan	0	Eye pain	0	Angry	0	Emergency care	0	Food allergies	0	Cadasil	0	5HT	0	Anatomy	0	Counselor	0	Phenotype	0	Abnormalities	0	Comorbidity	0	Corticosteroid	0	Mood disorder	0	CT scan	0	Foramen ovale	0	Angiotensin Converting Enzyme	0	Prophylactic treatment	0	Subtype	0	Randomized controlled trial	0	Melatonin	0	Referred pain	0	Comorbidities	0	Behavioral therapy	0	Transmission	0	Father	1	Preemptive	1	Grandfather	1	Adherence	1	Clinician	0	Complicated migraine	0	Placebo effect	0	Consultation	0	Irk	0	Beta-Blockers	0	carer	0
Social worker	0	Folate	0	Septoplasty	0	nan	0	Eye pain	0	Angry	0	Emergency care	0	Food allergies	0	Cadasil	0	5HT	0	Anatomy	0	Counselor	0	Phenotype	0	Abnormalities	0	Comorbidity	0	Corticosteroid	0	Mood disorder	0	CT scan	0	Foramen ovale	0	Angiotensin Converting Enzyme	0	Prophylactic treatment	0	Subtype	0	Randomized controlled trial	0	Melatonin	0	Referred pain	0	Comorbidities	0	Behavioral therapy	0	Transmission	0	Father	0	Preemptive	0	Grandfather	0	Adherence	0	Clinician	0	Complicated migraine	0	Placebo effect	0	Consultation	0	Irk	0	Beta-Blockers	0	carer	0
Social worker	0	Folate	0	Septoplasty	0	nan	0	Eye pain	0	Angry	0	Emergency care	0	Food allergies	0	Cadasil	0	5HT	0	Anatomy	0	Counselor	0	Phenotype	0	Abnormalities	0	Comorbidity	0	Corticosteroid	0	Mood disorder	0	CT scan	0	Foramen ovale	0	Angiotensin Converting Enzyme	0	Prophylactic treatment	0	Subtype	0	Randomized controlled trial	0	Melatonin	0	Referred pain	0	Comorbidities	0	Behavioral therapy	0	Transmission	0	Father	0	Preemptive	0	Grandfather	0	Adherence	0	Clinician	0	Complicated migraine	0	Placebo effect	0	Consultation	0	Irk	0	Beta-Blockers	0	carer	0
Social worker	0	Folate	0	Septoplasty	0	nan	0	Eye pain	0	Angry	0	Emergency care	0	Food allergies	0	Cadasil	0	5HT	0	Anatomy	0	Counselor	0	Phenotype	0	Abnormalities	0	Comorbidity	0	Corticosteroid	0	Mood disorder	0	CT scan	0	Foramen ovale	0	Angiotensin Converting Enzyme	0	Prophylactic treatment	0	Subtype	0	Randomized controlled trial	0	Melatonin	0	Referred pain	0	Comorbidities	0	Behavioral therapy	0	Transmission	0	Father	0	Preemptive	0	Grandfather	0	Adherence	0	Clinician	0	Complicated migraine	0	Placebo effect	0	Consultation	0	Irk	0	Beta-Blockers	0	carer	0
Social worker	0	Folate	0	Septoplasty	0	nan	0	Eye pain	0	Angry	0	Emergency care	0	Food allergies	0	Cadasil	0	5HT	0	Anatomy	0	Counselor	0	Phenotype	0	Abnormalities	0	Comorbidity	0	Corticosteroid	0	Mood disorder	0	CT scan	0	Foramen ovale	0	Angiotensin Converting Enzyme	0	Prophylactic treatment	0	Subtype	0	Randomized controlled trial	0	Melatonin	0	Referred pain	0	Comorbidities	0	Behavioral therapy	0	Transmission	0	Father	0	Preemptive	0	Grandfather	0	Adherence	0	Clinician	0	Complicated migraine	0	Placebo effect	0	Consultation	0	Irk	0	Beta-Blockers	0	carer	0
Social worker	0	Folate	0	Septoplasty	0	nan	0	Eye pain	0	Angry	0	Emergency care	0	Food allergies	0	Cadasil	0	5HT	0	Anatomy	0	Counselor	0	Phenotype	0	Abnormalities	0	Comorbidity	0	Corticosteroid	0	Mood disorder	0	CT scan	0	Foramen ovale	0	Angiotensin Converting Enzyme	0	Prophylactic treatment	0	Subtype	0	Randomized controlled trial	0	Melatonin	0	Referred pain	0	Comorbidities	0	Behavioral therapy	0	Transmission	0	Father	0	Preemptive	0	Grandfather	0	Adherence	0	Clinician	0	Complicated migraine	0	Placebo effect	0	Consultation	0	Irk	0	Beta-Blockers	0	carer	0
Social worker	0	Folate	0	Septoplasty	0	nan	0	Eye pain	0	Angry	0	Emergency care	0	Food allergies	0	Cadasil	0	5HT	0	Anatomy	0	Counselor	0	Phenotype	0	Abnormalities	0	Comorbidity	0	Corticosteroid	0	Mood disorder	0	CT scan	0	Foramen ovale	0	Angiotensin Converting Enzyme	0	Prophylactic treatment	0	Subtype	0	Randomized controlled trial	0	Melatonin	0	Referred pain	0	Comorbidities	1	Behavioral therapy	1	Transmission	1	Father	0	Preemptive	0	Grandfather	0	Adherence	0	Clinician	0	Complicated migraine	0	Placebo effect	0	Consultation	0	Irk	0	Beta-Blockers	0	carer	0
Social worker	0	Folate	0	Septoplasty	0	nan	0	Eye pain	0	Angry	0	Emergency care	0	Food allergies	0	Cadasil	0	5HT	0	Anatomy	0	Counselor	0	Phenotype	0	Abnormalities	0	Comorbidity	0	Corticosteroid	0	Mood disorder	0	CT scan	0	Foramen ovale	0	Angiotensin Converting Enzyme	0	Prophylactic treatment	0	Subtype	0	Randomized controlled trial	0	Melatonin	0	Referred pain	1	Comorbidities	0	Behavioral therapy	0	Transmission	0	Father	0	Preemptive	0	Grandfather	0	Adherence	0	Clinician	0	Complicated migraine	0	Placebo effect	0	Consultation	0	Irk	0	Beta-Blockers	0	carer	0
Social worker	0	Folate	0	Septoplasty	0	nan	0	Eye pain	0	Angry	0	Emergency care	0	Food allergies	0	Cadasil	0	5HT	0	Anatomy	0	Counselor	0	Phenotype	0	Abnormalities	0	Comorbidity	0	Corticosteroid	0	Mood disorder	0	CT scan	0	Foramen ovale	0	Angiotensin Converting Enzyme	0	Prophylactic treatment	0	Subtype	0	Randomized controlled trial	0	Melatonin	0	Referred pain	0	Comorbidities	0	Behavioral therapy	0	Transmission	0	Father	0	Preemptive	0	Grandfather	0	Adherence	0	Clinician	0	Complicated migraine	0	Placebo effect	0	Consultation	0	Irk	0	Beta-Blockers	0	carer	0
Social worker	0	Folate	0	Septoplasty	0	nan	0	Eye pain	0	Angry	0	Emergency care	0	Food allergies	0	Cadasil	0	5HT	0	Anatomy	0	Counselor	0	Phenotype	0	Abnormalities	0	Comorbidity	0	Corticosteroid	0	Mood disorder	0	CT scan	0	Foramen ovale	0	Angiotensin Converting Enzyme	0	Prophylactic treatment	0	Subtype	0	Randomized controlled trial	0	Melatonin	0	Referred pain	0	Comorbidities	0	Behavioral therapy	0	Transmission	0	Father	0	Preemptive	0	Grandfather	0	Adherence	0	Clinician	0	Complicated migraine	0	Placebo effect	0	Consultation	0	Irk	0	Beta-Blockers	0	carer	0
Social worker	0	Folate	0	Septoplasty	0	nan	0	Eye pain	0	Angry	0	Emergency care	0	Food allergies	0	Cadasil	0	5HT	0	Anatomy	0	Counselor	0	Phenotype	0	Abnormalities	0	Comorbidity	0	Corticosteroid	0	Mood disorder	0	CT scan	0	Foramen ovale	0	Angiotensin Converting Enzyme	0	Prophylactic treatment	0	Subtype	0	Randomized controlled trial	0	Melatonin	0	Referred pain	0	Comorbidities	0	Behavioral therapy	0	Transmission	0	Father	0	Preemptive	0	Grandfather	0	Adherence	0	Clinician	0	Complicated migraine	0	Placebo effect	0	Consultation	0	Irk	0	Beta-Blockers	0	carer	0
Social worker	0	Folate	0	Septoplasty	0	nan	0	Eye pain	0	Angry	0	Emergency care	0	Food allergies	0	Cadasil	0	5HT	0	Anatomy	0	Counselor	0	Phenotype	0	Abnormalities	0	Comorbidity	0	Corticosteroid	0	Mood disorder	0	CT scan	0	Foramen ovale	0	Angiotensin Converting Enzyme	0	Prophylactic treatment	0	Subtype	0	Randomized controlled trial	0	Melatonin	0	Referred pain	0	Comorbidities	0	Behavioral therapy	0	Transmission	0	Father	0	Preemptive	0	Grandfather	0	Adherence	0	Clinician	0	Complicated migraine	0	Placebo effect	0	Consultation	0	Irk	0	Beta-Blockers	0	carer	0
Social worker	0	Folate	0	Septoplasty	0	nan	0	Eye pain	0	Angry	0	Emergency care	0	Food allergies	0	Cadasil	0	5HT	0	Anatomy	0	Counselor	0	Phenotype	0	Abnormalities	0	Comorbidity	0	Corticosteroid	0	Mood disorder	0	CT scan	0	Foramen ovale	0	Angiotensin Converting Enzyme	0	Prophylactic treatment	0	Subtype	0	Randomized controlled trial	0	Melatonin	0	Referred pain	0	Comorbidities	0	Behavioral therapy	0	Transmission	0	Father	0	Preemptive	0	Grandfather	0	Adherence	0	Clinician	0	Complicated migraine	0	Placebo effect	0	Consultation	0	Irk	0	Beta-Blockers	0	carer	0
Social worker	0	Folate	0	Septoplasty	0	nan	0	Eye pain	0	Angry	0	Emergency care	0	Food allergies	0	Cadasil	0	5HT	0	Anatomy	0	Counselor	0	Phenotype	0	Abnormalities	0	Comorbidity	0	Corticosteroid	0	Mood disorder	0	CT scan	0	Foramen ovale	0	Angiotensin Converting Enzyme	0	Prophylactic treatment	0	Subtype	0	Randomized controlled trial	0	Melatonin	0	Referred pain	0	Comorbidities	0	Behavioral therapy	0	Transmission	0	Father	0	Preemptive	0	Grandfather	0	Adherence	0	Clinician	0	Complicated migraine	0	Placebo effect	0	Consultation	0	Irk	0	Beta-Blockers	0	carer	0
Social worker	0	Folate	0	Septoplasty	0	nan	0	Eye pain	0	Angry	0	Emergency care	0	Food allergies	0	Cadasil	0	5HT	0	Anatomy	0	Counselor	0	Phenotype	0	Abnormalities	0	Comorbidity	0	Corticosteroid	0	Mood disorder	0	CT scan	0	Foramen ovale	1	Angiotensin Converting Enzyme	1	Prophylactic treatment	1	Subtype	1	Randomized controlled trial	1	Melatonin	1	Referred pain	0	Comorbidities	0	Behavioral therapy	0	Transmission	0	Father	0	Preemptive	0	Grandfather	0	Adherence	0	Clinician	0	Complicated migraine	0	Placebo effect	0	Consultation	0	Irk	0	Beta-Blockers	0	carer	0
Social worker	0	Folate	0	Septoplasty	0	nan	0	Eye pain	0	Angry	0	Emergency care	0	Food allergies	0	Cadasil	0	5HT	0	Anatomy	0	Counselor	0	Phenotype	0	Abnormalities	0	Comorbidity	0	Corticosteroid	0	Mood disorder	0	CT scan	1	Foramen ovale	0	Angiotensin Converting Enzyme	0	Prophylactic treatment	0	Subtype	0	Randomized controlled trial	0	Melatonin	0	Referred pain	0	Comorbidities	0	Behavioral therapy	0	Transmission	0	Father	0	Preemptive	0	Grandfather	0	Adherence	0	Clinician	0	Complicated migraine	0	Placebo effect	0	Consultation	0	Irk	0	Beta-Blockers	0	carer	0
Social worker	0	Folate	0	Septoplasty	0	nan	0	Eye pain	0	Angry	0	Emergency care	0	Food allergies	0	Cadasil	0	5HT	0	Anatomy	0	Counselor	1	Phenotype	1	Abnormalities	1	Comorbidity	1	Corticosteroid	1	Mood disorder	1	CT scan	0	Foramen ovale	0	Angiotensin Converting Enzyme	0	Prophylactic treatment	0	Subtype	0	Randomized controlled trial	0	Melatonin	0	Referred pain	0	Comorbidities	0	Behavioral therapy	0	Transmission	0	Father	0	Preemptive	0	Grandfather	0	Adherence	0	Clinician	0	Complicated migraine	0	Placebo effect	0	Consultation	0	Irk	0	Beta-Blockers	0	carer	0
Social worker	0	Folate	0	Septoplasty	0	nan	0	Eye pain	0	Angry	0	Emergency care	0	Food allergies	0	Cadasil	0	5HT	0	Anatomy	0	Counselor	0	Phenotype	0	Abnormalities	0	Comorbidity	0	Corticosteroid	0	Mood disorder	0	CT scan	0	Foramen ovale	0	Angiotensin Converting Enzyme	0	Prophylactic treatment	0	Subtype	0	Randomized controlled trial	0	Melatonin	0	Referred pain	0	Comorbidities	0	Behavioral therapy	0	Transmission	0	Father	0	Preemptive	0	Grandfather	0	Adherence	0	Clinician	0	Complicated migraine	0	Placebo effect	0	Consultation	0	Irk	0	Beta-Blockers	0	carer	0
Social worker	0	Folate	0	Septoplasty	0	nan	0	Eye pain	0	Angry	0	Emergency care	0	Food allergies	0	Cadasil	0	5HT	0	Anatomy	0	Counselor	0	Phenotype	0	Abnormalities	0	Comorbidity	0	Corticosteroid	0	Mood disorder	0	CT scan	0	Foramen ovale	0	Angiotensin Converting Enzyme	0	Prophylactic treatment	0	Subtype	0	Randomized controlled trial	0	Melatonin	0	Referred pain	0	Comorbidities	0	Behavioral therapy	0	Transmission	0	Father	0	Preemptive	0	Grandfather	0	Adherence	0	Clinician	0	Complicated migraine	0	Placebo effect	0	Consultation	0	Irk	0	Beta-Blockers	0	carer	0
Social worker	0	Folate	0	Septoplasty	0	nan	0	Eye pain	0	Angry	0	Emergency care	0	Food allergies	0	Cadasil	0	5HT	0	Anatomy	0	Counselor	0	Phenotype	0	Abnormalities	0	Comorbidity	0	Corticosteroid	0	Mood disorder	0	CT scan	0	Foramen ovale	0	Angiotensin Converting Enzyme	0	Prophylactic treatment	0	Subtype	0	Randomized controlled trial	0	Melatonin	0	Referred pain	0	Comorbidities	0	Behavioral therapy	0	Transmission	0	Father	0	Preemptive	0	Grandfather	0	Adherence	0	Clinician	0	Complicated migraine	0	Placebo effect	0	Consultation	0	Irk	0	Beta-Blockers	0	carer	0
Social worker	0	Folate	0	Septoplasty	0	nan	0	Eye pain	0	Angry	0	Emergency care	0	Food allergies	0	Cadasil	0	5HT	0	Anatomy	0	Counselor	0	Phenotype	0	Abnormalities	0	Comorbidity	0	Corticosteroid	0	Mood disorder	0	CT scan	0	Foramen ovale	0	Angiotensin Converting Enzyme	0	Prophylactic treatment	0	Subtype	0	Randomized controlled trial	0	Melatonin	0	Referred pain	0	Comorbidities	0	Behavioral therapy	0	Transmission	0	Father	0	Preemptive	0	Grandfather	0	Adherence	0	Clinician	0	Complicated migraine	0	Placebo effect	0	Consultation	0	Irk	0	Beta-Blockers	0	carer	0
Social worker	0	Folate	0	Septoplasty	0	nan	0	Eye pain	0	Angry	0	Emergency care	0	Food allergies	0	Cadasil	0	5HT	0	Anatomy	0	Counselor	0	Phenotype	0	Abnormalities	0	Comorbidity	0	Corticosteroid	0	Mood disorder	0	CT scan	0	Foramen ovale	0	Angiotensin Converting Enzyme	0	Prophylactic treatment	0	Subtype	0	Randomized controlled trial	0	Melatonin	0	Referred pain	0	Comorbidities	0	Behavioral therapy	0	Transmission	0	Father	0	Preemptive	0	Grandfather	0	Adherence	0	Clinician	0	Complicated migraine	0	Placebo effect	0	Consultation	0	Irk	0	Beta-Blockers	0	carer	0
Social worker	0	Folate	0	Septoplasty	0	nan	0	Eye pain	0	Angry	0	Emergency care	0	Food allergies	0	Cadasil	0	5HT	0	Anatomy	1	Counselor	0	Phenotype	0	Abnormalities	0	Comorbidity	0	Corticosteroid	0	Mood disorder	0	CT scan	0	Foramen ovale	0	Angiotensin Converting Enzyme	0	Prophylactic treatment	0	Subtype	0	Randomized controlled trial	0	Melatonin	0	Referred pain	0	Comorbidities	0	Behavioral therapy	0	Transmission	0	Father	0	Preemptive	0	Grandfather	0	Adherence	0	Clinician	0	Complicated migraine	0	Placebo effect	0	Consultation	0	Irk	0	Beta-Blockers	0	carer	0
Social worker	0	Folate	0	Septoplasty	0	nan	0	Eye pain	0	Angry	0	Emergency care	0	Food allergies	0	Cadasil	0	5HT	0	Anatomy	0	Counselor	0	Phenotype	0	Abnormalities	0	Comorbidity	0	Corticosteroid	0	Mood disorder	0	CT scan	0	Foramen ovale	0	Angiotensin Converting Enzyme	0	Prophylactic treatment	0	Subtype	0	Randomized controlled trial	0	Melatonin	0	Referred pain	0	Comorbidities	0	Behavioral therapy	0	Transmission	0	Father	0	Preemptive	0	Grandfather	0	Adherence	0	Clinician	0	Complicated migraine	0	Placebo effect	0	Consultation	0	Irk	0	Beta-Blockers	0	carer	0
Social worker	0	Folate	0	Septoplasty	0	nan	0	Eye pain	0	Angry	0	Emergency care	0	Food allergies	0	Cadasil	0	5HT	0	Anatomy	0	Counselor	0	Phenotype	0	Abnormalities	0	Comorbidity	0	Corticosteroid	0	Mood disorder	0	CT scan	0	Foramen ovale	0	Angiotensin Converting Enzyme	0	Prophylactic treatment	0	Subtype	0	Randomized controlled trial	0	Melatonin	0	Referred pain	0	Comorbidities	0	Behavioral therapy	0	Transmission	0	Father	0	Preemptive	0	Grandfather	0	Adherence	0	Clinician	0	Complicated migraine	0	Placebo effect	0	Consultation	0	Irk	0	Beta-Blockers	0	carer	0
Social worker	0	Folate	0	Septoplasty	0	nan	0	Eye pain	0	Angry	0	Emergency care	0	Food allergies	0	Cadasil	0	5HT	0	Anatomy	0	Counselor	0	Phenotype	0	Abnormalities	0	Comorbidity	0	Corticosteroid	0	Mood disorder	0	CT scan	0	Foramen ovale	0	Angiotensin Converting Enzyme	0	Prophylactic treatment	0	Subtype	0	Randomized controlled trial	0	Melatonin	0	Referred pain	0	Comorbidities	0	Behavioral therapy	0	Transmission	0	Father	0	Preemptive	0	Grandfather	0	Adherence	0	Clinician	0	Complicated migraine	0	Placebo effect	0	Consultation	0	Irk	0	Beta-Blockers	0	carer	0
Social worker	0	Folate	0	Septoplasty	0	nan	0	Eye pain	0	Angry	0	Emergency care	0	Food allergies	0	Cadasil	0	5HT	0	Anatomy	0	Counselor	0	Phenotype	0	Abnormalities	0	Comorbidity	0	Corticosteroid	0	Mood disorder	0	CT scan	0	Foramen ovale	0	Angiotensin Converting Enzyme	0	Prophylactic treatment	0	Subtype	0	Randomized controlled trial	0	Melatonin	0	Referred pain	0	Comorbidities	0	Behavioral therapy	0	Transmission	0	Father	0	Preemptive	0	Grandfather	0	Adherence	0	Clinician	0	Complicated migraine	0	Placebo effect	0	Consultation	0	Irk	0	Beta-Blockers	0	carer	0
Social worker	0	Folate	0	Septoplasty	0	nan	0	Eye pain	0	Angry	0	Emergency care	0	Food allergies	0	Cadasil	0	5HT	0	Anatomy	0	Counselor	0	Phenotype	0	Abnormalities	0	Comorbidity	0	Corticosteroid	0	Mood disorder	0	CT scan	0	Foramen ovale	0	Angiotensin Converting Enzyme	0	Prophylactic treatment	0	Subtype	0	Randomized controlled trial	0	Melatonin	0	Referred pain	0	Comorbidities	0	Behavioral therapy	0	Transmission	0	Father	0	Preemptive	0	Grandfather	0	Adherence	0	Clinician	0	Complicated migraine	0	Placebo effect	0	Consultation	0	Irk	0	Beta-Blockers	0	carer	0
	2		2		2		2		2		2		2		2		2		2		1		1		1		1		1		1		1		1		1		1		1		1		1		1		1		1		1		1		1		1		1		1		1		1		1		1		1		1		1

November 2019 Migraine Webcrawl Data

Condition	URL Id	URL	Total number of words	P0a	P0b	P1a	P1b	P2a	P2b	P3a	P3b	P4a	P4b
Migraine	2174178	http://helpforheadaches.com/forum.htm	902	Migraine	15	Ill	3	Pain	0	Ache	12	work	3
Migraine	2293278	http://migrainetalk.forumotion.co.uk/	923	Migraine	15	Ill	0	Pain	0	Ache	0	work	0
Migraine	1679505	http://www.healthcentral.com/migraine/	547	Migraine	19	Ill	1	Pain	1	Ache	5	work	1
Migraine	2286545	http://www.migrainepage.com/	23	Migraine	0	Ill	0	Pain	0	Ache	0	work	0
Migraine	1752114	https://curetogether.com/Migraine/discuss/	36747	Migraine	487	Ill	213	Pain	153	Ache	137	work	141
Migraine	2208143	https://livingwell.migrainedisease.com	350	Migraine	7	Ill	3	Pain	0	Ache	3	work	0
Migraine	2100374	https://migrainebuddy.com/understanding-migraines-the-gender-divide/	2038	Migraine	117	Ill	5	Pain	14	Ache	3	work	7
Migraine	2008810	https://www.healthcentral.com/migraine/	463	Migraine	15	Ill	5	Pain	0	Ache	0	work	2
Migraine	2275844	https://www.infantrisk.com/forum/forum/medications-and-breastfeeding-mothers/other-uncategorized-medications/152-migraine-medications	2082	Migraine	15	Ill	7	Pain	1	Ache	3	work	4
Migraine	2290389	https://www.patientslikeme.com/conditions/135-migraine	3640	Migraine	175	Ill	1	Pain	21	Ache	20	work	0
			47715		865		238		190		183		158

URL Id	P5a	P5b	P6a	P6b	P7a	P7b	P8a	P8b	P9a	P9b	P10a	P10b	P11a	P11b	P12a	P12b	P13a	P13b	P14a	P14b
2174178	Evaluation	0	Trigger	0	Doctor	2	Triptan	0	medicine	0	drug	0	Medication	0	ACE	2	Son	2	Treatment	2
2293278	Evaluation	0	Trigger	1	Doctor	0	Triptan	0	medicine	0	drug	0	Medication	0	ACE	2	Son	3	Treatment	0
1679505	Evaluation	0	Trigger	1	Doctor	1	Triptan	0	medicine	0	drug	0	Medication	1	ACE	0	Son	0	Treatment	2
2286545	Evaluation	0	Trigger	0	Doctor	0	Triptan	0	medicine	0	drug	0	Medication	0	ACE	0	Son	0	Treatment	0
1752114	Evaluation	0	Trigger	74	Doctor	79	Triptan	36	medicine	65	drug	45	Medication	40	ACE	43	Son	42	Treatment	37
2208143	Evaluation	0	Trigger	0	Doctor	0	Triptan	0	medicine	0	drug	0	Medication	0	ACE	1	Son	1	Treatment	0
2100374	Evaluation	0	Trigger	10	Doctor	4	Triptan	0	medicine	0	drug	1	Medication	8	ACE	4	Son	3	Treatment	9
2008810	Evaluation	0	Trigger	1	Doctor	0	Triptan	0	medicine	0	drug	0	Medication	0	ACE	1	Son	3	Treatment	1
2275844	Evaluation	0	Trigger	0	Doctor	2	Triptan	16	medicine	1	drug	18	Medication	12	ACE	0	Son	3	Treatment	1
2290389	Evaluation	90	Trigger	1	Doctor	0	Triptan	18	medicine	0	drug	1	Medication	1	ACE	9	Son	1	Treatment	4
		90		88		88		70		66		65		62		62		58		56

URL Id	P15a	P15b	P16a	P16b	P17a	P17b	P18a	P18b	P19a	P19b	P20a	P20b	P21a	P21b	P22a	P22b	P23a	P23b	P24a	P24b
2174178	Good	0	good	0	Eye	0	Symptom	1	suffer	1	Aura	1	Relief	0	Herbal medicine	0	Nose	0	Nausea	0
2293278	Good	0	good	0	Eye	0	Symptom	0	suffer	4	Aura	0	Relief	0	Herbal medicine	0	Nose	0	Nausea	0
1679505	Good	0	good	0	Eye	0	Symptom	1	suffer	0	Aura	0	Relief	1	Herbal medicine	0	Nose	0	Nausea	0
2286545	Good	0	good	0	Eye	0	Symptom	0	suffer	0	Aura	0	Relief	0	Herbal medicine	0	Nose	0	Nausea	0
1752114	Good	53	good	53	Eye	54	Symptom	38	suffer	28	Aura	45	Relief	40	Herbal medicine	37	Nose	33	Nausea	26
2208143	Good	0	good	0	Eye	0	Symptom	0	suffer	0	Aura	0	Relief	0	Herbal medicine	0	Nose	0	Nausea	0
2100374	Good	0	good	0	Eye	0	Symptom	6	suffer	12	Aura	0	Relief	1	Herbal medicine	0	Nose	1	Nausea	2
2008810	Good	0	good	0	Eye	0	Symptom	1	suffer	1	Aura	0	Relief	0	Herbal medicine	0	Nose	0	Nausea	0
2275844	Good	1	good	1	Eye	0	Symptom	3	suffer	3	Aura	0	Relief	1	Herbal medicine	0	Nose	0	Nausea	1
2290389	Good	1	good	1	Eye	1	Symptom	4	suffer	0	Aura	2	Relief	0	Herbal medicine	0	Nose	0	Nausea	5
		55		55		55		54		49		48		43		37		34		34

URL Id	P25a	P25b	P26a	P26b	P27a	P27b	P28a	P28b	P29a	P29b	P30a	P30b	P31a	P31b	P32a	P32b	P33a	P33b
2174178	Diet	0	Gene	0	Sick	0	Hospital	0	Happy	0	Thought	0	Friend	2	Chronic	0	best	1
2293278	Diet	0	Gene	3	Sick	0	Hospital	0	Happy	0	Thought	0	Friend	0	Chronic	3	best	0
1679505	Diet	0	Gene	0	Sick	1	Hospital	1	Happy	0	Thought	0	Friend	0	Chronic	0	best	0
2286545	Diet	0	Gene	0	Sick	0	Hospital	0	Happy	0	Thought	0	Friend	0	Chronic	0	best	0
1752114	Diet	25	Gene	23	Sick	28	Hospital	28	Happy	26	Thought	25	Friend	22	Chronic	10	best	19
2208143	Diet	0	Gene	0	Sick	0	Hospital	0	Happy	0	Thought	0	Friend	1	Chronic	0	best	0
2100374	Diet	0	Gene	3	Sick	0	Hospital	1	Happy	0	Thought	0	Friend	0	Chronic	5	best	1
2008810	Diet	6	Gene	0	Sick	0	Hospital	0	Happy	0	Thought	0	Friend	0	Chronic	4	best	1
2275844	Diet	0	Gene	2	Sick	1	Hospital	0	Happy	0	Thought	0	Friend	0	Chronic	1	best	1
2290389	Diet	1	Gene	1	Sick	0	Hospital	0	Happy	0	Thought	1	Friend	0	Chronic	1	best	1
		32		32		30		30		26		26		25		24		24

URL Id	P34a	P34b	P35a	P35b	P36a	P36b	P37a	P37b	P38a	P38b	P39a	P39b	P40a	P40b	P41a	P41b	P42a
2174178	Injection	0	Botox	0	Stress	0	Bad	0	bad	0	worst	0	Exercise	0	exercise	0	Sinus
2293278	Injection	0	Botox	0	Stress	0	Bad	0	bad	0	worst	0	Exercise	0	exercise	0	Sinus
1679505	Injection	0	Botox	0	Stress	0	Bad	0	bad	0	worst	0	Exercise	0	exercise	0	Sinus
2286545	Injection	0	Botox	0	Stress	0	Bad	0	bad	0	worst	0	Exercise	0	exercise	0	Sinus
1752114	Injection	22	Botox	22	Stress	14	Bad	21	bad	21	worst	20	Exercise	18	exercise	18	Sinus
2208143	Injection	0	Botox	0	Stress	0	Bad	0	bad	0	worst	0	Exercise	0	exercise	0	Sinus
2100374	Injection	0	Botox	0	Stress	2	Bad	0	bad	0	worst	0	Exercise	2	exercise	2	Sinus
2008810	Injection	0	Botox	0	Stress	0	Bad	0	bad	0	worst	0	Exercise	0	exercise	0	Sinus
2275844	Injection	0	Botox	0	Stress	0	Bad	0	bad	0	worst	0	Exercise	0	exercise	0	Sinus
2290389	Injection	0	Botox	0	Stress	5	Bad	0	bad	0	worst	0	Exercise	0	exercise	0	Sinus
		22		22		21		21		21		20		20		20	

P42b	P43a	P43b	P44a	P44b	P45a	P45b	P46a	P46b	P47a	P47b	P48a	P48b	P49a	P49b	P50a	P50b	P51a	P51b
0	Therapy	0	sickness	0	Acupuncture	0	Cope	0	Drugs	0	Current	0	Medical	2	ill	0	Hormone	0
0	Therapy	0	sickness	0	Acupuncture	0	Cope	0	Drugs	0	Current	1	Medical	1	ill	0	Hormone	0
0	Therapy	0	sickness	0	Acupuncture	0	Cope	0	Drugs	0	Current	0	Medical	0	ill	0	Hormone	0
0	Therapy	0	sickness	0	Acupuncture	0	Cope	0	Drugs	0	Current	0	Medical	0	ill	0	Hormone	0
19	Therapy	18	sickness	18	Acupuncture	17	Cope	17	Drugs	15	Current	11	Medical	9	ill	9	Hormone	14
0	Therapy	0	sickness	0	Acupuncture	0	Cope	0	Drugs	0	Current	2	Medical	1	ill	0	Hormone	0
0	Therapy	0	sickness	0	Acupuncture	0	Cope	0	Drugs	1	Current	0	Medical	0	ill	3	Hormone	1
0	Therapy	0	sickness	0	Acupuncture	0	Cope	0	Drugs	0	Current	0	Medical	1	ill	3	Hormone	0
0	Therapy	1	sickness	0	Acupuncture	0	Cope	0	Drugs	1	Current	1	Medical	0	ill	0	Hormone	0
0	Therapy	0	sickness	0	Acupuncture	0	Cope	0	Drugs	0	Current	1	Medical	2	ill	0	Hormone	0
19		19		18		17		17		17		16		16		15		15

P52a	P52b	P53a	P53b	P54a	P54b	P55a	P55b	P56a	P56b	P57a	P57b	P58a	P58b	P59a	P59b	P60a	P60b
Neck pain	0	Depressed	0	Stroke	0	Ergot	0	Pregnancy	0	Photophobia	0	Acetaminophen	0	Tension	0	husband	0
Neck pain	0	Depressed	0	Stroke	0	Ergot	0	Pregnancy	0	Photophobia	0	Acetaminophen	0	Tension	0	husband	0
Neck pain	0	Depressed	0	Stroke	0	Ergot	0	Pregnancy	0	Photophobia	0	Acetaminophen	0	Tension	0	husband	0
Neck pain	0	Depressed	0	Stroke	0	Ergot	0	Pregnancy	0	Photophobia	0	Acetaminophen	0	Tension	0	husband	0
Neck pain	14	Depressed	8	Stroke	14	Ergot	9	Pregnancy	10	Photophobia	8	Acetaminophen	4	Tension	11	husband	12
Neck pain	0	Depressed	0	Stroke	0	Ergot	0	Pregnancy	0	Photophobia	0	Acetaminophen	0	Tension	0	husband	0
Neck pain	0	Depressed	1	Stroke	0	Ergot	0	Pregnancy	0	Photophobia	0	Acetaminophen	0	Tension	0	husband	0
Neck pain	0	Depressed	0	Stroke	0	Ergot	0	Pregnancy	0	Photophobia	0	Acetaminophen	0	Tension	0	husband	0
Neck pain	0	Depressed	0	Stroke	0	Ergot	4	Pregnancy	3	Photophobia	0	Acetaminophen	0	Tension	1	husband	0
Neck pain	0	Depressed	5	Stroke	0	Ergot	0	Pregnancy	0	Photophobia	5	Acetaminophen	9	Tension	1	husband	0
	14		14		14		13		13		13		13		13		12

P61a	P61b	P62a	P62b	P63a	P63b	P64a	P64b	P65a	P65b	P66a	P66b	P67a	P67b	P68a	P68b	P69a	P69b	P70a	P70b	P71a	P71b	P72a	P72b
Frequency	0	Magnesium	0	pray	0	Nerve	0	Prescription	0	Allodynia	0	Onset	0	Illness	0	nasal	0	Migraine attack	0	Chronic migraine	0	Clinic	1
Frequency	0	Magnesium	0	pray	0	Nerve	0	Prescription	0	Allodynia	0	Onset	0	Illness	0	nasal	0	Migraine attack	0	Chronic migraine	3	Clinic	0
Frequency	0	Magnesium	0	pray	0	Nerve	0	Prescription	0	Allodynia	0	Onset	0	Illness	0	nasal	0	Migraine attack	6	Chronic migraine	0	Clinic	0
Frequency	0	Magnesium	0	pray	0	Nerve	0	Prescription	0	Allodynia	0	Onset	0	Illness	0	nasal	0	Migraine attack	0	Chronic migraine	0	Clinic	0
Frequency	12	Magnesium	11	pray	11	Nerve	10	Prescription	9	Allodynia	10	Onset	9	Illness	4	nasal	10	Migraine attack	4	Chronic migraine	2	Clinic	4
Frequency	0	Magnesium	0	pray	0	Nerve	0	Prescription	0	Allodynia	0	Onset	0	Illness	0	nasal	0	Migraine attack	0	Chronic migraine	0	Clinic	0
Frequency	0	Magnesium	0	pray	0	Nerve	1	Prescription	2	Allodynia	0	Onset	0	Illness	3	nasal	0	Migraine attack	0	Chronic migraine	3	Clinic	2
Frequency	0	Magnesium	0	pray	0	Nerve	0	Prescription	0	Allodynia	0	Onset	0	Illness	3	nasal	0	Migraine attack	0	Chronic migraine	2	Clinic	0
Frequency	0	Magnesium	0	pray	0	Nerve	0	Prescription	0	Allodynia	0	Onset	1	Illness	0	nasal	0	Migraine attack	0	Chronic migraine	0	Clinic	0
Frequency	0	Magnesium	0	pray	0	Nerve	0	Prescription	0	Allodynia	0	Onset	0	Illness	0	nasal	0	Migraine attack	0	Chronic migraine	0	Clinic	3
	12		11		11		11		11		10		10		10		10		10		10		10

P73a	P73b	P74a	P74b	P75a	P75b	P76a	P76b	P77a	P77b	P78a	P78b	P79a	P79b	P80a	P80b	P81a	P81b	P82a	P82b	P83a	P83b	P84a	P84b	P85a	P85b	P86a	P86b	P87a	P87b	P88a	P88b	P89a
Abortive	0	Mother	0	ARB	0	Occipital	0	Lifestyle	0	Biofeedback	0	past	0	Sensitivity to light	0	Depression	0	Walk	0	Sensitivity to sound	0	Diagnosis	0	Wife	0	Procedure	0	Sore	0	procedure	0	Genetic
Abortive	0	Mother	0	ARB	0	Occipital	0	Lifestyle	0	Biofeedback	0	past	0	Sensitivity to light	0	Depression	0	Walk	0	Sensitivity to sound	0	Diagnosis	0	Wife	0	Procedure	0	Sore	0	procedure	0	Genetic
Abortive	0	Mother	0	ARB	0	Occipital	0	Lifestyle	0	Biofeedback	0	past	0	Sensitivity to light	0	Depression	0	Walk	0	Sensitivity to sound	0	Diagnosis	0	Wife	1	Procedure	0	Sore	0	procedure	0	Genetic
Abortive	0	Mother	0	ARB	0	Occipital	0	Lifestyle	0	Biofeedback	0	past	0	Sensitivity to light	0	Depression	0	Walk	0	Sensitivity to sound	0	Diagnosis	0	Wife	0	Procedure	0	Sore	0	procedure	0	Genetic
Abortive	9	Mother	6	ARB	8	Occipital	9	Lifestyle	4	Biofeedback	8	past	8	Sensitivity to light	7	Depression	1	Walk	6	Sensitivity to sound	3	Diagnosis	5	Wife	6	Procedure	6	Sore	6	procedure	6	Genetic
Abortive	0	Mother	0	ARB	0	Occipital	0	Lifestyle	0	Biofeedback	0	past	0	Sensitivity to light	0	Depression	0	Walk	0	Sensitivity to sound	0	Diagnosis	0	Wife	0	Procedure	0	Sore	0	procedure	0	Genetic
Abortive	0	Mother	0	ARB	0	Occipital	0	Lifestyle	4	Biofeedback	0	past	0	Sensitivity to light	1	Depression	4	Walk	1	Sensitivity to sound	0	Diagnosis	2	Wife	0	Procedure	0	Sore	0	procedure	0	Genetic
Abortive	0	Mother	0	ARB	0	Occipital	0	Lifestyle	0	Biofeedback	0	past	0	Sensitivity to light	0	Depression	0	Walk	0	Sensitivity to sound	0	Diagnosis	0	Wife	0	Procedure	0	Sore	0	procedure	0	Genetic
Abortive	0	Mother	3	ARB	0	Occipital	0	Lifestyle	0	Biofeedback	0	past	0	Sensitivity to light	0	Depression	2	Walk	0	Sensitivity to sound	0	Diagnosis	0	Wife	0	Procedure	0	Sore	0	procedure	0	Genetic
Abortive	0	Mother	0	ARB	1	Occipital	0	Lifestyle	0	Biofeedback	0	past	0	Sensitivity to light	0	Depression	0	Walk	0	Sensitivity to sound	4	Diagnosis	0	Wife	0	Procedure	0	Sore	0	procedure	0	Genetic
	9		9		9		9		8		8		8		8		7		7		7		7		7		6		6		6	

P89b	P90a	P90b	P91a	P91b	P92a	P92b	P93a	P93b	P94a	P94b	P95a	P95b	P96a	P96b	P97a	P97b	P98a	P98b	P99a	P99b	P100a	P100b
0	Menopause	0	manage	0	faith	0	Joy	0	Butalbital	0	Trigger point	0	Blocker	0	Child	0	Shock	0	Central	1	Sister	0
0	Menopause	0	manage	0	faith	0	Joy	0	Butalbital	0	Trigger point	0	Blocker	0	Child	0	Shock	1	Central	0	Sister	0
0	Menopause	0	manage	1	faith	0	Joy	0	Butalbital	0	Trigger point	0	Blocker	0	Child	1	Shock	0	Central	1	Sister	0
0	Menopause	0	manage	0	faith	0	Joy	0	Butalbital	0	Trigger point	0	Blocker	0	Child	0	Shock	0	Central	0	Sister	0
6	Menopause	6	manage	3	faith	5	Joy	5	Butalbital	5	Trigger point	5	Blocker	3	Child	2	Shock	4	Central	0	Sister	4
0	Menopause	0	manage	0	faith	0	Joy	0	Butalbital	0	Trigger point	0	Blocker	0	Child	0	Shock	0	Central	0	Sister	0
0	Menopause	0	manage	1	faith	0	Joy	0	Butalbital	0	Trigger point	0	Blocker	0	Child	2	Shock	0	Central	0	Sister	0
0	Menopause	0	manage	0	faith	0	Joy	0	Butalbital	0	Trigger point	0	Blocker	0	Child	0	Shock	0	Central	3	Sister	0
0	Menopause	0	manage	0	faith	0	Joy	0	Butalbital	0	Trigger point	0	Blocker	2	Child	0	Shock	0	Central	0	Sister	0
0	Menopause	0	manage	1	faith	0	Joy	0	Butalbital	0	Trigger point	0	Blocker	0	Child	0	Shock	0	Central	0	Sister	0
6		6		6		5		5		5		5		5		5		5		5		4

P101a	P101b	P102a	P102b	P103a	P103b	P104a	P104b	P105a	P105b	P106a	P106b	P107a	P107b	P108a	P108b	P109a	P109b	P110a	P110b	P111a	P111b
Epilepsy	0	stigma	0	Sad	0	Brother	0	Incidence	0	travel	0	Prophylactic	0	Steroid	0	Confuse	0	Emergency	0	Drive	1
Epilepsy	0	stigma	0	Sad	0	Brother	0	Incidence	0	travel	0	Prophylactic	0	Steroid	0	Confuse	0	Emergency	0	Drive	0
Epilepsy	0	stigma	0	Sad	0	Brother	0	Incidence	0	travel	0	Prophylactic	0	Steroid	0	Confuse	0	Emergency	1	Drive	0
Epilepsy	0	stigma	0	Sad	0	Brother	0	Incidence	0	travel	0	Prophylactic	0	Steroid	0	Confuse	0	Emergency	0	Drive	0
Epilepsy	4	stigma	4	Sad	4	Brother	4	Incidence	4	travel	2	Prophylactic	4	Steroid	4	Confuse	4	Emergency	3	Drive	2
Epilepsy	0	stigma	0	Sad	0	Brother	0	Incidence	0	travel	0	Prophylactic	0	Steroid	0	Confuse	0	Emergency	0	Drive	0
Epilepsy	0	stigma	0	Sad	0	Brother	0	Incidence	0	travel	1	Prophylactic	0	Steroid	0	Confuse	0	Emergency	0	Drive	0
Epilepsy	0	stigma	0	Sad	0	Brother	0	Incidence	0	travel	1	Prophylactic	0	Steroid	0	Confuse	0	Emergency	0	Drive	1
Epilepsy	0	stigma	0	Sad	0	Brother	0	Incidence	0	travel	0	Prophylactic	0	Steroid	0	Confuse	0	Emergency	0	Drive	0
Epilepsy	0	stigma	0	Sad	0	Brother	0	Incidence	0	travel	0	Prophylactic	0	Steroid	0	Confuse	0	Emergency	0	Drive	0
	4		4		4		4		4		4		4		4		4		4		4

P112a	P112b	P113a	P113b	P114a	P114b	P115a	P115b	P116a	P116b	P117a	P117b	P118a	P118b	P119a	P119b	P120a	P120b	P121a	P121b
Present	0	Duration	0	minister	0	Neck muscle	0	Nurse	0	nose	0	NSAID	0	Occipital nerve	0	Hurts	0	employ	0
Present	0	Duration	0	minister	0	Neck muscle	0	Nurse	0	nose	0	NSAID	0	Occipital nerve	0	Hurts	0	employ	0
Present	0	Duration	0	minister	0	Neck muscle	0	Nurse	0	nose	0	NSAID	0	Occipital nerve	0	Hurts	0	employ	0
Present	0	Duration	0	minister	0	Neck muscle	0	Nurse	0	nose	0	NSAID	0	Occipital nerve	0	Hurts	0	employ	0
Present	3	Duration	3	minister	3	Neck muscle	3	Nurse	1	nose	3	NSAID	1	Occipital nerve	3	Hurts	3	employ	3
Present	0	Duration	0	minister	0	Neck muscle	0	Nurse	0	nose	0	NSAID	0	Occipital nerve	0	Hurts	0	employ	0
Present	0	Duration	0	minister	0	Neck muscle	0	Nurse	0	nose	0	NSAID	0	Occipital nerve	0	Hurts	0	employ	0
Present	0	Duration	0	minister	0	Neck muscle	0	Nurse	0	nose	0	NSAID	0	Occipital nerve	0	Hurts	0	employ	0
Present	0	Duration	0	minister	0	Neck muscle	0	Nurse	2	nose	0	NSAID	2	Occipital nerve	0	Hurts	0	employ	0
Present	0	Duration	0	minister	0	Neck muscle	0	Nurse	0	nose	0	NSAID	0	Occipital nerve	0	Hurts	0	employ	0
	3		3		3		3		3		3		3		3		3		3

P122a	P122b	P123a	P123b	P124a	P124b	P125a	P125b	P126a	P126b	P127a	P127b	P128a	P128b	P129a	P129b	P130a	P130b	P131a	P131b	P132a	P132b
Cluster headache	0	Management	0	Cluster	0	Impact	0	Hemiplegic migraine	0	Preventive	1	Adverse effect	0	Lactation	0	Empower	0	Support group	0	Toxin	0
Cluster headache	0	Management	0	Cluster	0	Impact	0	Hemiplegic migraine	0	Preventive	0	Adverse effect	0	Lactation	0	Empower	0	Support group	0	Toxin	0
Cluster headache	3	Management	1	Cluster	3	Impact	1	Hemiplegic migraine	1	Preventive	0	Adverse effect	0	Lactation	0	Empower	0	Support group	0	Toxin	0
Cluster headache	0	Management	0	Cluster	0	Impact	0	Hemiplegic migraine	0	Preventive	0	Adverse effect	0	Lactation	0	Empower	0	Support group	0	Toxin	0
Cluster headache	0	Management	2	Cluster	0	Impact	1	Hemiplegic migraine	1	Preventive	2	Adverse effect	0	Lactation	0	Empower	0	Support group	0	Toxin	2
Cluster headache	0	Management	0	Cluster	0	Impact	0	Hemiplegic migraine	0	Preventive	0	Adverse effect	0	Lactation	0	Empower	0	Support group	0	Toxin	0
Cluster headache	0	Management	0	Cluster	0	Impact	1	Hemiplegic migraine	0	Preventive	0	Adverse effect	0	Lactation	0	Empower	0	Support group	2	Toxin	0
Cluster headache	0	Management	0	Cluster	0	Impact	0	Hemiplegic migraine	1	Preventive	0	Adverse effect	0	Lactation	0	Empower	2	Support group	0	Toxin	0
Cluster headache	0	Management	0	Cluster	0	Impact	0	Hemiplegic migraine	0	Preventive	0	Adverse effect	2	Lactation	2	Empower	0	Support group	0	Toxin	0
Cluster headache	0	Management	0	Cluster	0	Impact	0	Hemiplegic migraine	0	Preventive	0	Adverse effect	0	Lactation	0	Empower	0	Support group	0	Toxin	0
	3		3		3		3		3		3		2		2		2		2		2

P133a	P133b	P134a	P134b	P135a	P135b	P136a	P136b	P137a	P137b	P138a	P138b	P139a	P139b	P140a	P140b	P141a	P141b	P142a	P142b
Lifestyle change	0	Parent	0	Intractable	0	Anti-inflammatory	0	Quality of life	0	Glutamate	0	Daughter	0	Nerve block	0	Visual disturbance	0	Headache diary	0
Lifestyle change	0	Parent	0	Intractable	0	Anti-inflammatory	0	Quality of life	0	Glutamate	0	Daughter	0	Nerve block	0	Visual disturbance	0	Headache diary	0
Lifestyle change	0	Parent	0	Intractable	0	Anti-inflammatory	0	Quality of life	0	Glutamate	0	Daughter	0	Nerve block	0	Visual disturbance	0	Headache diary	0
Lifestyle change	0	Parent	0	Intractable	0	Anti-inflammatory	0	Quality of life	0	Glutamate	0	Daughter	0	Nerve block	0	Visual disturbance	0	Headache diary	0
Lifestyle change	2	Parent	2	Intractable	2	Anti-inflammatory	2	Quality of life	1	Glutamate	2	Daughter	2	Nerve block	2	Visual disturbance	2	Headache diary	1
Lifestyle change	0	Parent	0	Intractable	0	Anti-inflammatory	0	Quality of life	0	Glutamate	0	Daughter	0	Nerve block	0	Visual disturbance	0	Headache diary	0
Lifestyle change	0	Parent	0	Intractable	0	Anti-inflammatory	0	Quality of life	1	Glutamate	0	Daughter	0	Nerve block	0	Visual disturbance	0	Headache diary	1
Lifestyle change	0	Parent	0	Intractable	0	Anti-inflammatory	0	Quality of life	0	Glutamate	0	Daughter	0	Nerve block	0	Visual disturbance	0	Headache diary	0
Lifestyle change	0	Parent	0	Intractable	0	Anti-inflammatory	0	Quality of life	0	Glutamate	0	Daughter	0	Nerve block	0	Visual disturbance	0	Headache diary	0
Lifestyle change	0	Parent	0	Intractable	0	Anti-inflammatory	0	Quality of life	0	Glutamate	0	Daughter	0	Nerve block	0	Visual disturbance	0	Headache diary	0
	2		2		2		2		2		2		2		2		2		2

P143a	P143b	P144a	P144b	P145a	P145b	P146a	P146b	P147a	P147b	P148a	P148b	P149a	P149b	P150a	P150b	P151a	P151b	P152a	P152b
Accepting	0	Weight loss	0	Coping	0	vacation	0	Antidepressants	0	Anti-seizure	0	Vascular	0	Patterns	0	Surgery	0	History	0
Accepting	0	Weight loss	0	Coping	0	vacation	0	Antidepressants	0	Anti-seizure	0	Vascular	0	Patterns	0	Surgery	0	History	0
Accepting	0	Weight loss	0	Coping	0	vacation	0	Antidepressants	0	Anti-seizure	0	Vascular	0	Patterns	0	Surgery	0	History	0
Accepting	0	Weight loss	0	Coping	0	vacation	0	Antidepressants	0	Anti-seizure	0	Vascular	0	Patterns	0	Surgery	0	History	0
Accepting	2	Weight loss	2	Coping	1	vacation	2	Antidepressants	2	Anti-seizure	2	Vascular	2	Patterns	2	Surgery	2	History	1
Accepting	0	Weight loss	0	Coping	0	vacation	0	Antidepressants	0	Anti-seizure	0	Vascular	0	Patterns	0	Surgery	0	History	0
Accepting	0	Weight loss	0	Coping	1	vacation	0	Antidepressants	0	Anti-seizure	0	Vascular	0	Patterns	0	Surgery	0	History	0
Accepting	0	Weight loss	0	Coping	0	vacation	0	Antidepressants	0	Anti-seizure	0	Vascular	0	Patterns	0	Surgery	0	History	0
Accepting	0	Weight loss	0	Coping	0	vacation	0	Antidepressants	0	Anti-seizure	0	Vascular	0	Patterns	0	Surgery	0	History	0
Accepting	0	Weight loss	0	Coping	0	vacation	0	Antidepressants	0	Anti-seizure	0	Vascular	0	Patterns	0	Surgery	0	History	1
	2		2		2		2		2		2		2		2		2		2

P153a	P153b	P154a	P154b	P155a	P155b	P156a	P156b	P157a	P157b	P158a	P158b	P159a	P159b	P160a	P160b	P161a	P161b	P162a	P162b
Riboflavin	0	Menstrual migraine	0	Acute	0	Caregiver	0	Regimen	1	regime	1	boss	2	Inhibitor	0	Tricyclic	0	Stages	0
Riboflavin	0	Menstrual migraine	0	Acute	0	Caregiver	0	Regimen	0	regime	0	boss	0	Inhibitor	0	Tricyclic	0	Stages	0
Riboflavin	0	Menstrual migraine	0	Acute	1	Caregiver	1	Regimen	0	regime	0	boss	0	Inhibitor	0	Tricyclic	0	Stages	0
Riboflavin	0	Menstrual migraine	0	Acute	0	Caregiver	0	Regimen	0	regime	0	boss	0	Inhibitor	0	Tricyclic	0	Stages	0
Riboflavin	2	Menstrual migraine	1	Acute	1	Caregiver	0	Regimen	1	regime	1	boss	0	Inhibitor	0	Tricyclic	0	Stages	0
Riboflavin	0	Menstrual migraine	0	Acute	0	Caregiver	0	Regimen	0	regime	0	boss	0	Inhibitor	0	Tricyclic	0	Stages	0
Riboflavin	0	Menstrual migraine	0	Acute	0	Caregiver	1	Regimen	0	regime	0	boss	0	Inhibitor	0	Tricyclic	0	Stages	1
Riboflavin	0	Menstrual migraine	0	Acute	0	Caregiver	0	Regimen	0	regime	0	boss	0	Inhibitor	0	Tricyclic	0	Stages	0
Riboflavin	0	Menstrual migraine	1	Acute	0	Caregiver	0	Regimen	0	regime	0	boss	0	Inhibitor	1	Tricyclic	1	Stages	0
Riboflavin	0	Menstrual migraine	0	Acute	0	Caregiver	0	Regimen	0	regime	0	boss	0	Inhibitor	0	Tricyclic	0	Stages	0
	2		2		2		2		2		2		2		1		1		1

P163a	P163b	P164a	P164b	P165a	P165b	P166a	P166b	P167a	P167b	P168a	P168b	P169a	P169b	P170a	P170b	P171a	P171b	P172a	P172b
Blood flow	0	Serotonin	0	Gene-related	0	Chronic pain	0	Calcitonin	0	Trauma	0	Productivity	0	Advantage	0	Temporal	0	Grandmother	0
Blood flow	0	Serotonin	0	Gene-related	0	Chronic pain	0	Calcitonin	0	Trauma	0	Productivity	0	Advantage	0	Temporal	0	Grandmother	0
Blood flow	0	Serotonin	0	Gene-related	0	Chronic pain	0	Calcitonin	0	Trauma	0	Productivity	0	Advantage	0	Temporal	0	Grandmother	0
Blood flow	0	Serotonin	0	Gene-related	0	Chronic pain	0	Calcitonin	0	Trauma	0	Productivity	0	Advantage	0	Temporal	0	Grandmother	0
Blood flow	0	Serotonin	0	Gene-related	0	Chronic pain	0	Calcitonin	0	Trauma	0	Productivity	0	Advantage	0	Temporal	1	Grandmother	1
Blood flow	0	Serotonin	0	Gene-related	0	Chronic pain	0	Calcitonin	0	Trauma	0	Productivity	0	Advantage	1	Temporal	0	Grandmother	0
Blood flow	1	Serotonin	1	Gene-related	1	Chronic pain	1	Calcitonin	1	Trauma	1	Productivity	1	Advantage	0	Temporal	0	Grandmother	0
Blood flow	0	Serotonin	0	Gene-related	0	Chronic pain	0	Calcitonin	0	Trauma	0	Productivity	0	Advantage	0	Temporal	0	Grandmother	0
Blood flow	0	Serotonin	0	Gene-related	0	Chronic pain	0	Calcitonin	0	Trauma	0	Productivity	0	Advantage	0	Temporal	0	Grandmother	0
Blood flow	0	Serotonin	0	Gene-related	0	Chronic pain	0	Calcitonin	0	Trauma	0	Productivity	0	Advantage	0	Temporal	0	Grandmother	0
	1		1		1		1		1		1		1		1		1		1

P173a	P173b	P174a	P174b	P175a	P175b	P176a	P176b	P177a	P177b	P178a	P178b	P179a	P179b	P180a	P180b	P181a	P181b
Barbiturate	0	Testing	0	Aggravate	0	Sinus headache	0	Septoplasty	0	nun	0	Lidocaine	0	Pain management	0	Eye pain	0
Barbiturate	0	Testing	0	Aggravate	0	Sinus headache	0	Septoplasty	0	nun	0	Lidocaine	0	Pain management	0	Eye pain	0
Barbiturate	0	Testing	0	Aggravate	0	Sinus headache	0	Septoplasty	0	nun	0	Lidocaine	0	Pain management	0	Eye pain	0
Barbiturate	0	Testing	0	Aggravate	0	Sinus headache	0	Septoplasty	0	nun	0	Lidocaine	0	Pain management	0	Eye pain	0
Barbiturate	1	Testing	1	Aggravate	1	Sinus headache	1	Septoplasty	1	nun	1	Lidocaine	1	Pain management	1	Eye pain	1
Barbiturate	0	Testing	0	Aggravate	0	Sinus headache	0	Septoplasty	0	nun	0	Lidocaine	0	Pain management	0	Eye pain	0
Barbiturate	0	Testing	0	Aggravate	0	Sinus headache	0	Septoplasty	0	nun	0	Lidocaine	0	Pain management	0	Eye pain	0
Barbiturate	0	Testing	0	Aggravate	0	Sinus headache	0	Septoplasty	0	nun	0	Lidocaine	0	Pain management	0	Eye pain	0
Barbiturate	0	Testing	0	Aggravate	0	Sinus headache	0	Septoplasty	0	nun	0	Lidocaine	0	Pain management	0	Eye pain	0
Barbiturate	0	Testing	0	Aggravate	0	Sinus headache	0	Septoplasty	0	nun	0	Lidocaine	0	Pain management	0	Eye pain	0
	1		1		1		1		1		1		1		1		1

P182a	P182b	P183a	P183b	P184a	P184b	P185a	P185b	P186a	P186b	P187a	P187b	P188a	P188b	P189a	P189b	P190a	P190b
Spectrum	0	Mechanism	0	Angry	0	Agonist	0	Accident	0	Calcium channel	0	Calcium channel blocker	0	Upset	0	Emergency care	0
Spectrum	0	Mechanism	0	Angry	0	Agonist	0	Accident	0	Calcium channel	0	Calcium channel blocker	0	Upset	0	Emergency care	0
Spectrum	0	Mechanism	0	Angry	0	Agonist	0	Accident	0	Calcium channel	0	Calcium channel blocker	0	Upset	0	Emergency care	0
Spectrum	0	Mechanism	0	Angry	0	Agonist	0	Accident	0	Calcium channel	0	Calcium channel blocker	0	Upset	0	Emergency care	0
Spectrum	1	Mechanism	1	Angry	1	Agonist	1	Accident	1	Calcium channel	1	Calcium channel blocker	1	Upset	1	Emergency care	1
Spectrum	0	Mechanism	0	Angry	0	Agonist	0	Accident	0	Calcium channel	0	Calcium channel blocker	0	Upset	0	Emergency care	0
Spectrum	0	Mechanism	0	Angry	0	Agonist	0	Accident	0	Calcium channel	0	Calcium channel blocker	0	Upset	0	Emergency care	0
Spectrum	0	Mechanism	0	Angry	0	Agonist	0	Accident	0	Calcium channel	0	Calcium channel blocker	0	Upset	0	Emergency care	0
Spectrum	0	Mechanism	0	Angry	0	Agonist	0	Accident	0	Calcium channel	0	Calcium channel blocker	0	Upset	0	Emergency care	0
Spectrum	0	Mechanism	0	Angry	0	Agonist	0	Accident	0	Calcium channel	0	Calcium channel blocker	0	Upset	0	Emergency care	0
	1		1		1		1		1		1		1		1		1

P191a	P191b	P192a	P192b	P193a	P193b	P194a	P194b	P195a	P195b	P196a	P196b	P197a	P197b	P198a	P198b	P199a	P199b
Food allergies	0	Motivation	0	spouse	0	Cadasil	0	surgery	0	5HT	0	Acute migraine	0	carer	0	Education	1
Food allergies	0	Motivation	0	spouse	0	Cadasil	0	surgery	0	5HT	0	Acute migraine	0	carer	1	Education	0
Food allergies	0	Motivation	0	spouse	0	Cadasil	0	surgery	0	5HT	0	Acute migraine	1	carer	0	Education	0
Food allergies	0	Motivation	0	spouse	0	Cadasil	0	surgery	0	5HT	0	Acute migraine	0	carer	0	Education	0
Food allergies	1	Motivation	1	spouse	1	Cadasil	1	surgery	1	5HT	1	Acute migraine	0	carer	0	Education	0
Food allergies	0	Motivation	0	spouse	0	Cadasil	0	surgery	0	5HT	0	Acute migraine	0	carer	0	Education	0
Food allergies	0	Motivation	0	spouse	0	Cadasil	0	surgery	0	5HT	0	Acute migraine	0	carer	0	Education	0
Food allergies	0	Motivation	0	spouse	0	Cadasil	0	surgery	0	5HT	0	Acute migraine	0	carer	0	Education	0
Food allergies	0	Motivation	0	spouse	0	Cadasil	0	surgery	0	5HT	0	Acute migraine	0	carer	0	Education	0
Food allergies	0	Motivation	0	spouse	0	Cadasil	0	surgery	0	5HT	0	Acute migraine	0	carer	0	Education	0
	1		1		1		1		1		1		1		1		1

March 2021 Migraine Webcrawl Data

Condition	URL Id	URL	Total number of words	P0a	P0b	P1a	P1b	P2a	P2b	P3a	P3b	P4a	P4b	P5a	P5b
Migraine	1502221	https://migraineresearchfoundation.org/resources/resources-and-links/	1694	Migraine	37	Ache	34	Ill	11	Pain	4	work	2	Doctor	5
Migraine	1502222	http://www.thedailymigraine.com/forum-moot/	126	Migraine	4	Ache	0	Ill	0	Pain	0	work	0	Doctor	0
Migraine	1502223	https://www.dailystrength.org/group/migraine-headaches	835	Migraine	11	Ache	5	Ill	0	Pain	1	work	1	Doctor	1
Migraine	1502227	http://migrainetalk.forumotion.co.uk/	924	Migraine	15	Ache	0	Ill	0	Pain	0	work	0	Doctor	0
Migraine	1502228	http://www.thedailymigraine.com/support-groups/	295	Migraine	11	Ache	0	Ill	2	Pain	6	work	0	Doctor	0
Migraine	1502231	https://ergoweb.com/forums/topic/treatment-of-light-induced-migraine-headaches-in-the-office/	1236	Migraine	11	Ache	6	Ill	4	Pain	0	work	6	Doctor	0
Migraine	1502232	http://www.migrainebuddy.com/migraine-stories/	639	Migraine	23	Ache	0	Ill	4	Pain	0	work	1	Doctor	1
Migraine	1502238	http://forums.prohealth.com/forums/index.php?threads/migraine-from-hell-called-status-migrainosus.110648/	1390	Migraine	17	Ache	4	Ill	4	Pain	9	work	2	Doctor	5
Migraine	1502237	https://www.chronofhorse.com/forum/archive/index.php/t-318339.html	5506	Migraine	38	Ache	12	Ill	29	Pain	9	work	12	Doctor	3
Migraine	1502241	http://www.themigrainerevolution.com/yahoo-group/	501	Migraine	16	Ache	0	Ill	2	Pain	0	work	0	Doctor	0
Migraine	1502243	https://hubpages.com/health/forum/27466/any-ocular-migraine-sufferers-here	2165	Migraine	27	Ache	5	Ill	5	Pain	9	work	4	Doctor	1
Migraine	1502245	https://www.facebook.com/MigraineDotCom	998	Migraine	21	Ache	0	Ill	5	Pain	2	work	1	Doctor	2
Migraine	1502246	https://www.physicsforums.com/threads/the-four-stages-of-migraine.305792/	2099	Migraine	63	Ache	24	Ill	2	Pain	2	work	2	Doctor	2
Migraine	1502248	https://patient.info/forums/discuss/browse/migraine-1415	3176	Migraine	47	Ache	31	Ill	10	Pain	11	work	3	Doctor	9
Migraine	1502251	https://www.medschat.com/Discuss/Migraine-Tension-Headache/	3348	Migraine	21	Ache	19	Ill	22	Pain	4	work	8	Doctor	4
Migraine	1502253	https://www.cumom.ie/forums/topic/bad-bad-migraine/	1330	Migraine	10	Ache	3	Ill	4	Pain	3	work	2	Doctor	3
Migraine	1502254	http://www.healthcentral.com/migraine/c/123/177401/experience-spring-migraine	1259	Migraine	42	Ache	4	Ill	3	Pain	0	work	1	Doctor	1
Migraine	1502256	http://forums.phoenixrising.me/index.php?threads/questions-about-migraine-aura.34477/	4369	Migraine	64	Ache	11	Ill	19	Pain	9	work	9	Doctor	2
Migraine	1502257	http://www.experienceproject.com/groups/Have-Vestibular-Migraine/117436	43	Migraine	0	Ache	0	Ill	1	Pain	0	work	0	Doctor	0
Migraine	1502259	https://migraine.newlifeoutlook.com/community/	518	Migraine	9	Ache	1	Ill	0	Pain	0	work	0	Doctor	1
Migraine	1502263	https://www.dinet.org/forums/topic/27868-anybody-else-have-an-intractable-migraine/	2069	Migraine	21	Ache	11	Ill	6	Pain	16	work	8	Doctor	6
Migraine	1502268	http://www.healthcentral.com/migraine/	538	Migraine	21	Ache	1	Ill	1	Pain	0	work	1	Doctor	2
Migraine	1502270	https://forum.dontpayfull.com/threads/migraine-medicine.25791/	3248	Migraine	36	Ache	29	Ill	17	Pain	16	work	13	Doctor	6
Migraine	1502274	http://www.healthfulchat.org/migraine-chat-room.html	1063	Migraine	25	Ache	8	Ill	1	Pain	2	work	7	Doctor	0
Migraine	1502275	http://www.fibromyalgiaforums.org/forum/general-discussions-about-fibromyalgia/26893-fibro-migraines.html	2552	Migraine	27	Ache	20	Ill	4	Pain	18	work	10	Doctor	3
Migraine	1502276	http://www.migraine.org.uk/get-advice/	440	Migraine	9	Ache	0	Ill	0	Pain	0	work	0	Doctor	0
Migraine	1502278	http://www.fightingfatigue.org/headache-migraine-websites-forums/	431	Migraine	5	Ache	4	Ill	1	Pain	0	work	0	Doctor	0
Migraine	1502284	https://www.tapatalk.com/groups/vets/topic/67841/Migraines?page=2	13151	Migraine	134	Ache	71	Ill	30	Pain	18	work	41	Doctor	12
Migraine	1502287	https://www.fodors.com/community/air-travel/long-flight-migraine.cfm	2581	Migraine	27	Ache	14	Ill	7	Pain	1	work	4	Doctor	9
Migraine	1502291	https://www.curezone.org/forums/f3.asp?f=68	5642	Migraine	29	Ache	42	Ill	14	Pain	7	work	3	Doctor	1
Migraine	1502292	http://www.migrainepage.com/	23	Migraine	0	Ache	0	Ill	0	Pain	0	work	0	Doctor	0
Migraine	1502294	https://cassiopaea.org/forum/?topic=17971.0	2138	Migraine	0	Ache	0	Ill	4	Pain	0	work	1	Doctor	0
Migraine	1502296	http://www.topix.com/forum/health/migraine	1755	Migraine	25	Ache	3	Ill	3	Pain	4	work	2	Doctor	2
Migraine	1502297	https://www.healingwell.com/community/?f=31	734	Migraine	9	Ache	5	Ill	1	Pain	3	work	0	Doctor	0
Migraine	1502301	http://curetogether.com/Migraine/discuss/	36387	Migraine	487	Ache	137	Ill	213	Pain	153	work	141	Doctor	70
Migraine	1502305	http://www.migrainepage.com/help.html	287	Migraine	10	Ache	8	Ill	0	Pain	0	work	0	Doctor	0
Migraine	1502318	http://www.migrainebuddy.com/understanding-migraines-the-gender-divide/	1999	Migraine	114	Ache	3	Ill	6	Pain	14	work	7	Doctor	3
Migraine	1502320	https://www.curezone.org/forums/am.asp?i=60104	1529	Migraine	23	Ache	6	Ill	3	Pain	2	work	7	Doctor	0
Migraine	1502321	https://www.inspire.com/groups/headaches-and-migraines/discussion/migraines-each-day-unbeareable/	5326	Migraine	86	Ache	40	Ill	16	Pain	9	work	14	Doctor	5
Migraine	1502322	http://www.relieve-migraine-headache.com/basilar-migraines.html	773	Migraine	20	Ache	5	Ill	2	Pain	0	work	0	Doctor	5
Migraine	1502325	http://migrainepage.forumotion.net/	197	Migraine	0	Ache	0	Ill	0	Pain	0	work	0	Doctor	0
Migraine	1502327	https://www.tapatalk.com/groups/headachepainfreeforums/topic/766/Silent-Migraine	1643	Migraine	18	Ache	4	Ill	7	Pain	9	work	9	Doctor	4
Migraine	1502330	https://community.qvc.com/t5/Wellness/MIGRAINE-sufferers/td-p/1182015	3297	Migraine	34	Ache	3	Ill	6	Pain	6	work	8	Doctor	2
Migraine	1502331	https://forum.grasscity.com/threads/migraines.453628/	2427	Migraine	46	Ache	14	Ill	9	Pain	8	work	3	Doctor	3
Migraine	1502332	http://www.usmle-forums.com/usmle-step-1-forum/217482-migraine.html	693	Migraine	4	Ache	1	Ill	0	Pain	0	work	0	Doctor	0
Migraine	1502333	https://coloradopain.co/patient-forms/	377	Migraine	0	Ache	0	Ill	2	Pain	5	work	2	Doctor	1
Migraine	1502334	https://www.infantrisk.com/forum/forum/medications-and-breastfeeding-mothers/other-uncategorized-medications/152-migraine-medications	2082	Migraine	15	Ache	3	Ill	7	Pain	1	work	4	Doctor	2
Migraine	1502336	https://ehealthforum.com/health/headache-and-migraines-question-t504544.html	737	Migraine	1	Ache	8	Ill	1	Pain	0	work	1	Doctor	6
Migraine	1502338	http://helpforheadaches.com/forum.htm	902	Migraine	15	Ache	12	Ill	3	Pain	0	work	3	Doctor	2
Migraine	1502341	http://www.helpforheadaches.com/	389	Migraine	13	Ache	7	Ill	4	Pain	0	work	1	Doctor	1
Migraine	1502346	https://forum.bulletproof.com/discussion/1406/bulletproof-migraine-specific-diet	1772	Migraine	21	Ache	11	Ill	5	Pain	3	work	5	Doctor	4
Migraine	1502348	http://www.medhelp.org/forums/Migraines--Headaches/show/123	1345	Migraine	15	Ache	21	Ill	0	Pain	13	work	0	Doctor	4
Migraine	1502349	http://www.mvertigo.org/	323	Migraine	2	Ache	0	Ill	0	Pain	0	work	0	Doctor	0
Migraine	1502355	http://www.nomorepanic.co.uk/showthread.php?t=106093	1917	Migraine	16	Ache	0	Ill	5	Pain	5	work	3	Doctor	1
Migraine	1502358	http://forums.whirlpool.net.au/archive/1924289	8824	Migraine	88	Ache	38	Ill	42	Pain	42	work	26	Doctor	13
Migraine	1502359	http://www.migraines.org/help/helpform.htm	340	Migraine	8	Ache	2	Ill	1	Pain	0	work	1	Doctor	0
Migraine	1502362	http://www.tmswiki.org/forum/threads/my-migraines.2072/	2467	Migraine	30	Ache	9	Ill	8	Pain	6	work	7	Doctor	1
Migraine	1502369	https://migraine.newlifeoutlook.com/community/discussion/17/complex-migraine	680	Migraine	13	Ache	8	Ill	1	Pain	1	work	1	Doctor	0
Migraine	1502370	http://migrainepage.forumotion.net/f1-migraines	1251	Migraine	7	Ache	0	Ill	0	Pain	0	work	1	Doctor	0
Migraine	1502371	https://migrainetalkblog.wordpress.com/	1179	Migraine	57	Ache	9	Ill	3	Pain	0	work	0	Doctor	0
Migraine	1502372	https://www.patientslikeme.com/conditions/135-migraine	3564	Migraine	164	Ache	15	Ill	1	Pain	26	work	0	Doctor	0
Migraine	1502374	http://www.thedailyheadache.com/	2758	Migraine	32	Ache	18	Ill	42	Pain	4	work	16	Doctor	4
Migraine	1502375	http://www.experienceproject.com/groups/Get-Migraine-Headaches/102	43	Migraine	0	Ache	0	Ill	1	Pain	0	work	0	Doctor	0
Migraine	1502380	http://www.migraine.org.uk/	322	Migraine	7	Ache	1	Ill	0	Pain	0	work	0	Doctor	0
			154646		2201		750		604		461		404		212

URL Id	P6a	P6b	P7a	P7b	P8a	P8b	P9a	P9b	P10a	P10b	P11a	P11b	P12a	P12b	P13a	P13b	P14a	P14b	P15a	P15b	P16a	P16b	P17a	P17b	P18a	P18b	P19a	P19b	P20a	P20b	P21a	P21b	P22a	P22b	P23a	P23b
1502221	Trigger	0	suffer	12	Symptom	0	Treatment	10	ACE	4	Son	1	Good	0	good	0	Aura	0	Medication	4	Chronic	7	drug	1	Eye	0	medicine	3	Triptan	0	Bad	0	bad	0	Evaluation	0
1502222	Trigger	0	suffer	0	Symptom	0	Treatment	0	ACE	0	Son	0	Good	0	good	0	Aura	0	Medication	0	Chronic	1	drug	0	Eye	0	medicine	0	Triptan	0	Bad	0	bad	0	Evaluation	0
1502223	Trigger	1	suffer	1	Symptom	1	Treatment	1	ACE	0	Son	0	Good	1	good	1	Aura	0	Medication	0	Chronic	0	drug	0	Eye	0	medicine	0	Triptan	0	Bad	0	bad	0	Evaluation	0
1502227	Trigger	1	suffer	4	Symptom	0	Treatment	0	ACE	2	Son	3	Good	0	good	0	Aura	0	Medication	0	Chronic	3	drug	0	Eye	0	medicine	0	Triptan	0	Bad	0	bad	0	Evaluation	0
1502228	Trigger	0	suffer	0	Symptom	0	Treatment	0	ACE	2	Son	0	Good	0	good	0	Aura	0	Medication	0	Chronic	6	drug	0	Eye	0	medicine	0	Triptan	0	Bad	0	bad	0	Evaluation	0
1502231	Trigger	2	suffer	1	Symptom	2	Treatment	2	ACE	1	Son	2	Good	0	good	0	Aura	0	Medication	0	Chronic	0	drug	0	Eye	10	medicine	0	Triptan	0	Bad	0	bad	0	Evaluation	0
1502232	Trigger	2	suffer	2	Symptom	1	Treatment	1	ACE	1	Son	1	Good	0	good	0	Aura	0	Medication	2	Chronic	0	drug	0	Eye	0	medicine	0	Triptan	0	Bad	0	bad	0	Evaluation	0
1502238	Trigger	0	suffer	2	Symptom	2	Treatment	2	ACE	3	Son	1	Good	1	good	1	Aura	1	Medication	1	Chronic	0	drug	0	Eye	0	medicine	1	Triptan	0	Bad	3	bad	3	Evaluation	0
1502237	Trigger	22	suffer	4	Symptom	1	Treatment	0	ACE	6	Son	9	Good	7	good	7	Aura	1	Medication	1	Chronic	5	drug	1	Eye	3	medicine	1	Triptan	0	Bad	6	bad	6	Evaluation	0
1502241	Trigger	0	suffer	1	Symptom	0	Treatment	0	ACE	0	Son	0	Good	0	good	0	Aura	0	Medication	2	Chronic	0	drug	1	Eye	0	medicine	0	Triptan	0	Bad	0	bad	0	Evaluation	0
1502243	Trigger	5	suffer	11	Symptom	2	Treatment	0	ACE	0	Son	0	Good	0	good	0	Aura	3	Medication	1	Chronic	0	drug	0	Eye	7	medicine	0	Triptan	0	Bad	4	bad	4	Evaluation	0
1502245	Trigger	4	suffer	1	Symptom	2	Treatment	1	ACE	3	Son	0	Good	0	good	0	Aura	0	Medication	2	Chronic	0	drug	1	Eye	0	medicine	0	Triptan	0	Bad	1	bad	1	Evaluation	0
1502246	Trigger	8	suffer	6	Symptom	4	Treatment	0	ACE	0	Son	8	Good	2	good	2	Aura	6	Medication	0	Chronic	0	drug	0	Eye	2	medicine	0	Triptan	0	Bad	0	bad	0	Evaluation	0
1502248	Trigger	0	suffer	10	Symptom	13	Treatment	3	ACE	6	Son	1	Good	2	good	2	Aura	8	Medication	0	Chronic	6	drug	1	Eye	12	medicine	0	Triptan	8	Bad	4	bad	4	Evaluation	0
1502251	Trigger	0	suffer	2	Symptom	3	Treatment	4	ACE	4	Son	11	Good	3	good	3	Aura	2	Medication	9	Chronic	0	drug	9	Eye	1	medicine	6	Triptan	1	Bad	0	bad	0	Evaluation	0
1502253	Trigger	4	suffer	8	Symptom	0	Treatment	2	ACE	2	Son	0	Good	2	good	2	Aura	0	Medication	0	Chronic	0	drug	1	Eye	1	medicine	0	Triptan	1	Bad	6	bad	6	Evaluation	0
1502254	Trigger	0	suffer	0	Symptom	0	Treatment	13	ACE	0	Son	3	Good	1	good	1	Aura	3	Medication	2	Chronic	0	drug	0	Eye	0	medicine	0	Triptan	6	Bad	0	bad	0	Evaluation	0
1502256	Trigger	9	suffer	3	Symptom	10	Treatment	0	ACE	1	Son	5	Good	7	good	7	Aura	24	Medication	4	Chronic	2	drug	6	Eye	5	medicine	2	Triptan	1	Bad	6	bad	6	Evaluation	0
1502257	Trigger	0	suffer	0	Symptom	0	Treatment	0	ACE	0	Son	0	Good	0	good	0	Aura	0	Medication	0	Chronic	0	drug	0	Eye	0	medicine	0	Triptan	0	Bad	0	bad	0	Evaluation	0
1502259	Trigger	1	suffer	1	Symptom	0	Treatment	2	ACE	0	Son	2	Good	0	good	0	Aura	0	Medication	0	Chronic	0	drug	0	Eye	0	medicine	0	Triptan	0	Bad	0	bad	0	Evaluation	0
1502263	Trigger	1	suffer	1	Symptom	0	Treatment	7	ACE	3	Son	4	Good	1	good	1	Aura	2	Medication	12	Chronic	2	drug	0	Eye	0	medicine	1	Triptan	0	Bad	1	bad	1	Evaluation	0
1502268	Trigger	1	suffer	0	Symptom	2	Treatment	2	ACE	0	Son	0	Good	0	good	0	Aura	0	Medication	4	Chronic	0	drug	3	Eye	0	medicine	0	Triptan	0	Bad	0	bad	0	Evaluation	0
1502270	Trigger	5	suffer	3	Symptom	0	Treatment	0	ACE	6	Son	3	Good	6	good	6	Aura	0	Medication	1	Chronic	0	drug	0	Eye	0	medicine	20	Triptan	0	Bad	5	bad	5	Evaluation	0
1502274	Trigger	0	suffer	3	Symptom	1	Treatment	1	ACE	3	Son	1	Good	1	good	1	Aura	0	Medication	1	Chronic	0	drug	0	Eye	0	medicine	0	Triptan	0	Bad	0	bad	0	Evaluation	0
1502275	Trigger	1	suffer	2	Symptom	5	Treatment	0	ACE	0	Son	2	Good	2	good	2	Aura	1	Medication	1	Chronic	37	drug	0	Eye	4	medicine	0	Triptan	1	Bad	1	bad	1	Evaluation	0
1502276	Trigger	0	suffer	1	Symptom	0	Treatment	1	ACE	0	Son	0	Good	0	good	0	Aura	0	Medication	0	Chronic	0	drug	0	Eye	0	medicine	0	Triptan	0	Bad	0	bad	0	Evaluation	0
1502278	Trigger	0	suffer	0	Symptom	4	Treatment	0	ACE	0	Son	1	Good	0	good	0	Aura	0	Medication	0	Chronic	3	drug	0	Eye	0	medicine	0	Triptan	0	Bad	0	bad	0	Evaluation	0
1502284	Trigger	2	suffer	3	Symptom	3	Treatment	3	ACE	13	Son	5	Good	7	good	7	Aura	0	Medication	8	Chronic	5	drug	1	Eye	2	medicine	2	Triptan	0	Bad	6	bad	6	Evaluation	6
1502287	Trigger	7	suffer	3	Symptom	5	Treatment	1	ACE	3	Son	4	Good	1	good	1	Aura	0	Medication	2	Chronic	0	drug	0	Eye	0	medicine	0	Triptan	0	Bad	5	bad	5	Evaluation	0
1502291	Trigger	0	suffer	4	Symptom	7	Treatment	2	ACE	2	Son	5	Good	8	good	8	Aura	0	Medication	0	Chronic	5	drug	2	Eye	1	medicine	0	Triptan	0	Bad	2	bad	2	Evaluation	0
1502292	Trigger	0	suffer	0	Symptom	0	Treatment	0	ACE	0	Son	0	Good	0	good	0	Aura	0	Medication	0	Chronic	0	drug	0	Eye	0	medicine	0	Triptan	0	Bad	0	bad	0	Evaluation	0
1502294	Trigger	1	suffer	0	Symptom	0	Treatment	0	ACE	3	Son	2	Good	1	good	1	Aura	0	Medication	0	Chronic	0	drug	0	Eye	0	medicine	0	Triptan	0	Bad	0	bad	0	Evaluation	0
1502296	Trigger	2	suffer	1	Symptom	1	Treatment	3	ACE	1	Son	0	Good	1	good	1	Aura	0	Medication	0	Chronic	4	drug	4	Eye	1	medicine	2	Triptan	1	Bad	0	bad	0	Evaluation	0
1502297	Trigger	0	suffer	1	Symptom	0	Treatment	0	ACE	0	Son	0	Good	0	good	0	Aura	0	Medication	0	Chronic	3	drug	0	Eye	1	medicine	0	Triptan	0	Bad	0	bad	0	Evaluation	0
1502301	Trigger	74	suffer	28	Symptom	38	Treatment	37	ACE	43	Son	42	Good	53	good	53	Aura	45	Medication	40	Chronic	10	drug	45	Eye	54	medicine	56	Triptan	36	Bad	21	bad	21	Evaluation	0
1502305	Trigger	0	suffer	8	Symptom	0	Treatment	4	ACE	0	Son	0	Good	0	good	0	Aura	0	Medication	0	Chronic	0	drug	0	Eye	0	medicine	0	Triptan	0	Bad	0	bad	0	Evaluation	0
1502318	Trigger	9	suffer	12	Symptom	6	Treatment	9	ACE	4	Son	3	Good	0	good	0	Aura	0	Medication	8	Chronic	5	drug	1	Eye	0	medicine	0	Triptan	0	Bad	0	bad	0	Evaluation	0
1502320	Trigger	1	suffer	1	Symptom	2	Treatment	0	ACE	1	Son	1	Good	0	good	0	Aura	3	Medication	0	Chronic	0	drug	1	Eye	1	medicine	0	Triptan	0	Bad	1	bad	1	Evaluation	0
1502321	Trigger	5	suffer	3	Symptom	6	Treatment	10	ACE	15	Son	7	Good	12	good	12	Aura	5	Medication	5	Chronic	5	drug	22	Eye	0	medicine	4	Triptan	2	Bad	0	bad	0	Evaluation	1
1502322	Trigger	0	suffer	0	Symptom	7	Treatment	4	ACE	2	Son	2	Good	0	good	0	Aura	0	Medication	1	Chronic	0	drug	0	Eye	1	medicine	0	Triptan	1	Bad	1	bad	1	Evaluation	0
1502325	Trigger	0	suffer	0	Symptom	0	Treatment	0	ACE	0	Son	1	Good	0	good	0	Aura	0	Medication	0	Chronic	0	drug	0	Eye	0	medicine	0	Triptan	0	Bad	0	bad	0	Evaluation	0
1502327	Trigger	0	suffer	4	Symptom	13	Treatment	5	ACE	3	Son	1	Good	4	good	4	Aura	0	Medication	2	Chronic	0	drug	2	Eye	0	medicine	0	Triptan	0	Bad	2	bad	2	Evaluation	0
1502330	Trigger	0	suffer	1	Symptom	0	Treatment	1	ACE	0	Son	0	Good	2	good	2	Aura	0	Medication	3	Chronic	1	drug	0	Eye	0	medicine	1	Triptan	0	Bad	1	bad	1	Evaluation	0
1502331	Trigger	1	suffer	0	Symptom	3	Treatment	5	ACE	0	Son	3	Good	1	good	1	Aura	0	Medication	0	Chronic	4	drug	3	Eye	0	medicine	1	Triptan	0	Bad	6	bad	6	Evaluation	0
1502332	Trigger	0	suffer	0	Symptom	0	Treatment	1	ACE	0	Son	0	Good	0	good	0	Aura	0	Medication	0	Chronic	0	drug	0	Eye	0	medicine	0	Triptan	1	Bad	0	bad	0	Evaluation	0
1502333	Trigger	0	suffer	1	Symptom	1	Treatment	1	ACE	0	Son	0	Good	0	good	0	Aura	0	Medication	0	Chronic	0	drug	0	Eye	0	medicine	2	Triptan	0	Bad	0	bad	0	Evaluation	0
1502334	Trigger	0	suffer	3	Symptom	3	Treatment	1	ACE	0	Son	3	Good	1	good	1	Aura	0	Medication	12	Chronic	1	drug	18	Eye	0	medicine	1	Triptan	16	Bad	0	bad	0	Evaluation	0
1502336	Trigger	0	suffer	1	Symptom	2	Treatment	3	ACE	3	Son	1	Good	0	good	0	Aura	0	Medication	0	Chronic	0	drug	1	Eye	0	medicine	0	Triptan	0	Bad	0	bad	0	Evaluation	0
1502338	Trigger	0	suffer	1	Symptom	1	Treatment	2	ACE	2	Son	2	Good	0	good	0	Aura	1	Medication	0	Chronic	0	drug	0	Eye	0	medicine	0	Triptan	0	Bad	0	bad	0	Evaluation	0
1502341	Trigger	0	suffer	0	Symptom	1	Treatment	3	ACE	1	Son	1	Good	0	good	0	Aura	0	Medication	0	Chronic	0	drug	0	Eye	0	medicine	0	Triptan	1	Bad	0	bad	0	Evaluation	0
1502346	Trigger	2	suffer	6	Symptom	0	Treatment	4	ACE	2	Son	3	Good	2	good	2	Aura	0	Medication	2	Chronic	1	drug	0	Eye	0	medicine	0	Triptan	0	Bad	0	bad	0	Evaluation	0
1502348	Trigger	0	suffer	2	Symptom	2	Treatment	3	ACE	1	Son	1	Good	0	good	0	Aura	0	Medication	0	Chronic	2	drug	0	Eye	4	medicine	1	Triptan	0	Bad	2	bad	2	Evaluation	0
1502349	Trigger	0	suffer	0	Symptom	0	Treatment	0	ACE	0	Son	1	Good	0	good	0	Aura	0	Medication	0	Chronic	0	drug	0	Eye	0	medicine	0	Triptan	0	Bad	1	bad	1	Evaluation	0
1502355	Trigger	0	suffer	0	Symptom	1	Treatment	2	ACE	2	Son	3	Good	2	good	2	Aura	8	Medication	1	Chronic	0	drug	0	Eye	2	medicine	0	Triptan	0	Bad	2	bad	2	Evaluation	0
1502358	Trigger	14	suffer	18	Symptom	15	Treatment	6	ACE	6	Son	3	Good	12	good	12	Aura	18	Medication	5	Chronic	1	drug	5	Eye	15	medicine	0	Triptan	7	Bad	10	bad	10	Evaluation	0
1502359	Trigger	0	suffer	1	Symptom	0	Treatment	1	ACE	1	Son	0	Good	0	good	0	Aura	0	Medication	0	Chronic	0	drug	0	Eye	0	medicine	0	Triptan	0	Bad	0	bad	0	Evaluation	0
1502362	Trigger	0	suffer	2	Symptom	7	Treatment	1	ACE	1	Son	0	Good	6	good	6	Aura	0	Medication	1	Chronic	3	drug	3	Eye	0	medicine	0	Triptan	0	Bad	0	bad	0	Evaluation	0
1502369	Trigger	0	suffer	1	Symptom	0	Treatment	0	ACE	0	Son	0	Good	0	good	0	Aura	0	Medication	0	Chronic	0	drug	0	Eye	2	medicine	0	Triptan	0	Bad	0	bad	0	Evaluation	0
1502370	Trigger	0	suffer	0	Symptom	0	Treatment	0	ACE	0	Son	1	Good	0	good	0	Aura	1	Medication	0	Chronic	0	drug	0	Eye	1	medicine	0	Triptan	0	Bad	0	bad	0	Evaluation	0
1502371	Trigger	4	suffer	4	Symptom	3	Treatment	1	ACE	1	Son	0	Good	0	good	0	Aura	9	Medication	1	Chronic	6	drug	0	Eye	0	medicine	0	Triptan	0	Bad	0	bad	0	Evaluation	0
1502372	Trigger	1	suffer	0	Symptom	4	Treatment	4	ACE	9	Son	1	Good	1	good	1	Aura	1	Medication	1	Chronic	1	drug	0	Eye	0	medicine	0	Triptan	18	Bad	0	bad	0	Evaluation	90
1502374	Trigger	1	suffer	1	Symptom	1	Treatment	8	ACE	2	Son	2	Good	2	good	2	Aura	1	Medication	0	Chronic	9	drug	0	Eye	0	medicine	0	Triptan	0	Bad	1	bad	1	Evaluation	0
1502375	Trigger	0	suffer	0	Symptom	0	Treatment	0	ACE	0	Son	0	Good	0	good	0	Aura	0	Medication	0	Chronic	0	drug	0	Eye	0	medicine	0	Triptan	0	Bad	0	bad	0	Evaluation	0
1502380	Trigger	0	suffer	0	Symptom	0	Treatment	0	ACE	0	Son	0	Good	0	good	0	Aura	0	Medication	0	Chronic	0	drug	0	Eye	0	medicine	0	Triptan	0	Bad	0	bad	0	Evaluation	0
		191		189		182		175		169		158		152		152		145		139		138		133		129		104		101		98		98		97

URL Id	P24a	P24b	P25a	P25b	P26a	P26b	P27a	P27b	P28a	P28b	P29a	P29b	P30a	P30b	P31a	P31b	P32a	P32b	P33a	P33b	P34a	P34b	P35a	P35b	P36a	P36b	P37a	P37b	P38a	P38b	P39a	P39b	P40a	P40b	P41a	P41b	P42a	P42b	P43a	P43b
1502221	Gene	0	Relief	1	Medical	2	Nose	0	Thought	2	Nausea	0	Diet	0	best	3	Current	0	Stress	0	Sick	0	ill	3	Hospital	0	Friend	1	Botox	0	Drugs	1	Tension	0	Chronic migraine	3	Clinic	3	Nerve	0
1502222	Gene	0	Relief	0	Medical	0	Nose	0	Thought	0	Nausea	0	Diet	0	best	0	Current	0	Stress	0	Sick	0	ill	0	Hospital	0	Friend	0	Botox	0	Drugs	0	Tension	0	Chronic migraine	1	Clinic	0	Nerve	0
1502223	Gene	0	Relief	0	Medical	1	Nose	2	Thought	0	Nausea	1	Diet	0	best	0	Current	0	Stress	0	Sick	1	ill	0	Hospital	0	Friend	0	Botox	0	Drugs	0	Tension	0	Chronic migraine	0	Clinic	0	Nerve	0
1502227	Gene	3	Relief	0	Medical	1	Nose	0	Thought	0	Nausea	0	Diet	0	best	0	Current	1	Stress	0	Sick	0	ill	0	Hospital	0	Friend	0	Botox	0	Drugs	0	Tension	0	Chronic migraine	3	Clinic	0	Nerve	0
1502228	Gene	0	Relief	0	Medical	0	Nose	0	Thought	0	Nausea	0	Diet	0	best	0	Current	0	Stress	0	Sick	0	ill	1	Hospital	0	Friend	0	Botox	0	Drugs	0	Tension	0	Chronic migraine	1	Clinic	0	Nerve	0
1502231	Gene	2	Relief	0	Medical	1	Nose	1	Thought	0	Nausea	1	Diet	0	best	0	Current	1	Stress	0	Sick	1	ill	0	Hospital	0	Friend	0	Botox	0	Drugs	0	Tension	0	Chronic migraine	0	Clinic	0	Nerve	0
1502232	Gene	0	Relief	0	Medical	0	Nose	0	Thought	0	Nausea	0	Diet	0	best	0	Current	0	Stress	0	Sick	0	ill	0	Hospital	0	Friend	0	Botox	0	Drugs	0	Tension	0	Chronic migraine	0	Clinic	0	Nerve	0
1502238	Gene	0	Relief	3	Medical	0	Nose	2	Thought	0	Nausea	0	Diet	0	best	0	Current	1	Stress	1	Sick	0	ill	0	Hospital	3	Friend	0	Botox	0	Drugs	0	Tension	0	Chronic migraine	0	Clinic	1	Nerve	0
1502237	Gene	12	Relief	1	Medical	2	Nose	0	Thought	2	Nausea	3	Diet	5	best	4	Current	5	Stress	9	Sick	4	ill	1	Hospital	0	Friend	0	Botox	2	Drugs	1	Tension	0	Chronic migraine	0	Clinic	5	Nerve	1
1502241	Gene	1	Relief	0	Medical	0	Nose	0	Thought	0	Nausea	0	Diet	0	best	0	Current	0	Stress	0	Sick	0	ill	0	Hospital	0	Friend	0	Botox	0	Drugs	0	Tension	0	Chronic migraine	0	Clinic	0	Nerve	0
1502243	Gene	0	Relief	0	Medical	0	Nose	0	Thought	4	Nausea	1	Diet	1	best	3	Current	0	Stress	1	Sick	1	ill	0	Hospital	0	Friend	0	Botox	0	Drugs	0	Tension	0	Chronic migraine	0	Clinic	0	Nerve	0
1502245	Gene	0	Relief	0	Medical	0	Nose	0	Thought	0	Nausea	0	Diet	0	best	0	Current	0	Stress	0	Sick	0	ill	0	Hospital	0	Friend	0	Botox	0	Drugs	0	Tension	0	Chronic migraine	0	Clinic	1	Nerve	0
1502246	Gene	4	Relief	0	Medical	0	Nose	4	Thought	0	Nausea	1	Diet	0	best	0	Current	1	Stress	1	Sick	0	ill	0	Hospital	0	Friend	1	Botox	0	Drugs	0	Tension	0	Chronic migraine	0	Clinic	1	Nerve	0
1502248	Gene	0	Relief	2	Medical	6	Nose	6	Thought	1	Nausea	1	Diet	0	best	0	Current	1	Stress	2	Sick	0	ill	1	Hospital	0	Friend	0	Botox	1	Drugs	0	Tension	0	Chronic migraine	2	Clinic	1	Nerve	0
1502251	Gene	3	Relief	2	Medical	2	Nose	2	Thought	0	Nausea	8	Diet	0	best	1	Current	0	Stress	1	Sick	1	ill	0	Hospital	2	Friend	0	Botox	0	Drugs	0	Tension	5	Chronic migraine	0	Clinic	0	Nerve	0
1502253	Gene	2	Relief	1	Medical	1	Nose	0	Thought	2	Nausea	0	Diet	3	best	0	Current	0	Stress	5	Sick	0	ill	1	Hospital	1	Friend	0	Botox	0	Drugs	0	Tension	0	Chronic migraine	0	Clinic	2	Nerve	0
1502254	Gene	0	Relief	0	Medical	0	Nose	0	Thought	0	Nausea	0	Diet	0	best	0	Current	1	Stress	0	Sick	0	ill	0	Hospital	1	Friend	0	Botox	4	Drugs	0	Tension	1	Chronic migraine	0	Clinic	1	Nerve	0
1502256	Gene	0	Relief	0	Medical	2	Nose	2	Thought	8	Nausea	4	Diet	2	best	0	Current	2	Stress	1	Sick	0	ill	5	Hospital	4	Friend	4	Botox	0	Drugs	2	Tension	0	Chronic migraine	0	Clinic	0	Nerve	1
1502257	Gene	0	Relief	0	Medical	0	Nose	0	Thought	0	Nausea	0	Diet	0	best	0	Current	0	Stress	0	Sick	0	ill	0	Hospital	0	Friend	0	Botox	0	Drugs	0	Tension	0	Chronic migraine	0	Clinic	0	Nerve	0
1502259	Gene	0	Relief	0	Medical	1	Nose	1	Thought	0	Nausea	0	Diet	1	best	0	Current	0	Stress	0	Sick	0	ill	0	Hospital	1	Friend	0	Botox	1	Drugs	0	Tension	0	Chronic migraine	0	Clinic	1	Nerve	0
1502263	Gene	1	Relief	1	Medical	2	Nose	5	Thought	0	Nausea	0	Diet	0	best	0	Current	0	Stress	0	Sick	0	ill	0	Hospital	0	Friend	0	Botox	3	Drugs	0	Tension	1	Chronic migraine	1	Clinic	0	Nerve	4
1502268	Gene	1	Relief	1	Medical	1	Nose	0	Thought	0	Nausea	0	Diet	0	best	0	Current	0	Stress	0	Sick	1	ill	0	Hospital	1	Friend	0	Botox	0	Drugs	2	Tension	0	Chronic migraine	0	Clinic	0	Nerve	0
1502270	Gene	3	Relief	2	Medical	0	Nose	0	Thought	0	Nausea	0	Diet	0	best	5	Current	2	Stress	1	Sick	4	ill	0	Hospital	2	Friend	2	Botox	0	Drugs	0	Tension	11	Chronic migraine	0	Clinic	0	Nerve	0
1502274	Gene	0	Relief	0	Medical	2	Nose	0	Thought	0	Nausea	1	Diet	0	best	0	Current	0	Stress	2	Sick	0	ill	0	Hospital	0	Friend	0	Botox	0	Drugs	0	Tension	0	Chronic migraine	0	Clinic	0	Nerve	0
1502275	Gene	0	Relief	2	Medical	2	Nose	2	Thought	0	Nausea	1	Diet	1	best	2	Current	2	Stress	1	Sick	0	ill	0	Hospital	0	Friend	0	Botox	0	Drugs	0	Tension	1	Chronic migraine	4	Clinic	0	Nerve	1
1502276	Gene	0	Relief	0	Medical	0	Nose	0	Thought	0	Nausea	0	Diet	0	best	0	Current	0	Stress	0	Sick	0	ill	0	Hospital	0	Friend	2	Botox	0	Drugs	0	Tension	0	Chronic migraine	0	Clinic	0	Nerve	0
1502278	Gene	0	Relief	0	Medical	0	Nose	0	Thought	0	Nausea	0	Diet	0	best	0	Current	0	Stress	0	Sick	0	ill	1	Hospital	0	Friend	0	Botox	0	Drugs	0	Tension	0	Chronic migraine	0	Clinic	0	Nerve	0
1502284	Gene	2	Relief	0	Medical	17	Nose	4	Thought	1	Nausea	0	Diet	2	best	3	Current	7	Stress	0	Sick	3	ill	15	Hospital	5	Friend	4	Botox	0	Drugs	1	Tension	0	Chronic migraine	2	Clinic	1	Nerve	5
1502287	Gene	0	Relief	0	Medical	0	Nose	2	Thought	1	Nausea	2	Diet	0	best	0	Current	0	Stress	0	Sick	9	ill	0	Hospital	0	Friend	1	Botox	0	Drugs	0	Tension	0	Chronic migraine	0	Clinic	0	Nerve	0
1502291	Gene	2	Relief	4	Medical	1	Nose	0	Thought	1	Nausea	0	Diet	6	best	3	Current	0	Stress	0	Sick	0	ill	0	Hospital	0	Friend	3	Botox	0	Drugs	0	Tension	3	Chronic migraine	2	Clinic	1	Nerve	0
1502292	Gene	0	Relief	0	Medical	0	Nose	0	Thought	0	Nausea	0	Diet	0	best	0	Current	0	Stress	0	Sick	0	ill	0	Hospital	0	Friend	0	Botox	0	Drugs	0	Tension	0	Chronic migraine	0	Clinic	0	Nerve	0
1502294	Gene	2	Relief	0	Medical	0	Nose	0	Thought	0	Nausea	0	Diet	1	best	0	Current	0	Stress	1	Sick	0	ill	1	Hospital	0	Friend	1	Botox	0	Drugs	0	Tension	0	Chronic migraine	0	Clinic	0	Nerve	0
1502296	Gene	1	Relief	0	Medical	0	Nose	0	Thought	1	Nausea	0	Diet	0	best	0	Current	0	Stress	0	Sick	0	ill	0	Hospital	0	Friend	0	Botox	2	Drugs	0	Tension	0	Chronic migraine	4	Clinic	0	Nerve	0
1502297	Gene	0	Relief	0	Medical	0	Nose	0	Thought	0	Nausea	0	Diet	0	best	1	Current	2	Stress	0	Sick	0	ill	0	Hospital	0	Friend	0	Botox	0	Drugs	0	Tension	0	Chronic migraine	0	Clinic	0	Nerve	0
1502301	Gene	23	Relief	40	Medical	9	Nose	30	Thought	25	Nausea	26	Diet	25	best	19	Current	11	Stress	14	Sick	28	ill	9	Hospital	28	Friend	19	Botox	22	Drugs	15	Tension	11	Chronic migraine	2	Clinic	4	Nerve	10
1502305	Gene	1	Relief	1	Medical	0	Nose	0	Thought	0	Nausea	0	Diet	0	best	0	Current	1	Stress	0	Sick	0	ill	0	Hospital	0	Friend	0	Botox	0	Drugs	0	Tension	0	Chronic migraine	0	Clinic	0	Nerve	0
1502318	Gene	3	Relief	1	Medical	0	Nose	1	Thought	0	Nausea	2	Diet	0	best	1	Current	0	Stress	2	Sick	0	ill	3	Hospital	1	Friend	0	Botox	0	Drugs	1	Tension	0	Chronic migraine	3	Clinic	2	Nerve	1
1502320	Gene	0	Relief	1	Medical	0	Nose	0	Thought	1	Nausea	3	Diet	1	best	4	Current	0	Stress	0	Sick	0	ill	0	Hospital	0	Friend	2	Botox	0	Drugs	0	Tension	0	Chronic migraine	0	Clinic	0	Nerve	0
1502321	Gene	3	Relief	4	Medical	3	Nose	1	Thought	5	Nausea	3	Diet	1	best	1	Current	3	Stress	0	Sick	0	ill	3	Hospital	1	Friend	1	Botox	6	Drugs	18	Tension	3	Chronic migraine	2	Clinic	7	Nerve	0
1502322	Gene	2	Relief	0	Medical	0	Nose	0	Thought	1	Nausea	1	Diet	0	best	0	Current	0	Stress	0	Sick	0	ill	0	Hospital	0	Friend	0	Botox	0	Drugs	0	Tension	0	Chronic migraine	0	Clinic	0	Nerve	0
1502325	Gene	0	Relief	0	Medical	0	Nose	0	Thought	0	Nausea	0	Diet	0	best	0	Current	1	Stress	0	Sick	0	ill	0	Hospital	0	Friend	0	Botox	0	Drugs	0	Tension	0	Chronic migraine	0	Clinic	0	Nerve	0
1502327	Gene	0	Relief	1	Medical	0	Nose	1	Thought	0	Nausea	2	Diet	1	best	2	Current	0	Stress	0	Sick	0	ill	0	Hospital	0	Friend	1	Botox	0	Drugs	0	Tension	0	Chronic migraine	0	Clinic	0	Nerve	0
1502330	Gene	1	Relief	2	Medical	0	Nose	0	Thought	2	Nausea	1	Diet	1	best	1	Current	2	Stress	0	Sick	0	ill	0	Hospital	0	Friend	1	Botox	4	Drugs	0	Tension	0	Chronic migraine	1	Clinic	3	Nerve	0
1502331	Gene	0	Relief	3	Medical	0	Nose	4	Thought	0	Nausea	0	Diet	1	best	3	Current	0	Stress	0	Sick	0	ill	3	Hospital	0	Friend	0	Botox	0	Drugs	0	Tension	1	Chronic migraine	4	Clinic	2	Nerve	0
1502332	Gene	0	Relief	0	Medical	2	Nose	0	Thought	0	Nausea	0	Diet	0	best	0	Current	0	Stress	0	Sick	0	ill	0	Hospital	0	Friend	0	Botox	0	Drugs	0	Tension	0	Chronic migraine	0	Clinic	0	Nerve	0
1502333	Gene	0	Relief	0	Medical	0	Nose	0	Thought	0	Nausea	0	Diet	0	best	0	Current	0	Stress	0	Sick	0	ill	0	Hospital	0	Friend	0	Botox	0	Drugs	0	Tension	0	Chronic migraine	0	Clinic	0	Nerve	0
1502334	Gene	2	Relief	1	Medical	0	Nose	0	Thought	0	Nausea	1	Diet	0	best	1	Current	1	Stress	0	Sick	1	ill	0	Hospital	0	Friend	0	Botox	0	Drugs	1	Tension	1	Chronic migraine	0	Clinic	0	Nerve	0
1502336	Gene	0	Relief	0	Medical	8	Nose	3	Thought	0	Nausea	0	Diet	0	best	0	Current	0	Stress	0	Sick	0	ill	0	Hospital	0	Friend	0	Botox	0	Drugs	0	Tension	0	Chronic migraine	0	Clinic	0	Nerve	0
1502338	Gene	0	Relief	0	Medical	2	Nose	0	Thought	0	Nausea	0	Diet	0	best	1	Current	0	Stress	0	Sick	0	ill	0	Hospital	0	Friend	2	Botox	0	Drugs	0	Tension	0	Chronic migraine	0	Clinic	1	Nerve	0
1502341	Gene	2	Relief	0	Medical	1	Nose	0	Thought	0	Nausea	0	Diet	0	best	0	Current	0	Stress	0	Sick	0	ill	0	Hospital	0	Friend	1	Botox	0	Drugs	0	Tension	0	Chronic migraine	0	Clinic	0	Nerve	0
1502346	Gene	0	Relief	2	Medical	1	Nose	0	Thought	0	Nausea	0	Diet	12	best	2	Current	0	Stress	2	Sick	0	ill	0	Hospital	2	Friend	0	Botox	0	Drugs	0	Tension	0	Chronic migraine	0	Clinic	0	Nerve	0
1502348	Gene	3	Relief	1	Medical	5	Nose	0	Thought	0	Nausea	0	Diet	0	best	0	Current	1	Stress	0	Sick	0	ill	0	Hospital	0	Friend	0	Botox	0	Drugs	0	Tension	1	Chronic migraine	1	Clinic	0	Nerve	1
1502349	Gene	0	Relief	1	Medical	0	Nose	0	Thought	0	Nausea	0	Diet	0	best	1	Current	0	Stress	0	Sick	0	ill	0	Hospital	0	Friend	0	Botox	0	Drugs	0	Tension	0	Chronic migraine	0	Clinic	0	Nerve	0
1502355	Gene	2	Relief	0	Medical	0	Nose	0	Thought	2	Nausea	1	Diet	0	best	0	Current	1	Stress	0	Sick	1	ill	0	Hospital	0	Friend	0	Botox	0	Drugs	0	Tension	0	Chronic migraine	0	Clinic	0	Nerve	0
1502358	Gene	8	Relief	6	Medical	0	Nose	5	Thought	7	Nausea	7	Diet	3	best	3	Current	0	Stress	6	Sick	3	ill	3	Hospital	2	Friend	2	Botox	1	Drugs	3	Tension	2	Chronic migraine	0	Clinic	2	Nerve	0
1502359	Gene	0	Relief	0	Medical	2	Nose	0	Thought	0	Nausea	0	Diet	0	best	0	Current	0	Stress	0	Sick	0	ill	0	Hospital	0	Friend	0	Botox	0	Drugs	0	Tension	0	Chronic migraine	0	Clinic	0	Nerve	0
1502362	Gene	2	Relief	2	Medical	1	Nose	2	Thought	6	Nausea	1	Diet	0	best	0	Current	0	Stress	0	Sick	0	ill	2	Hospital	0	Friend	1	Botox	0	Drugs	1	Tension	3	Chronic migraine	2	Clinic	0	Nerve	0
1502369	Gene	0	Relief	0	Medical	0	Nose	1	Thought	4	Nausea	2	Diet	0	best	1	Current	0	Stress	0	Sick	0	ill	0	Hospital	0	Friend	0	Botox	0	Drugs	0	Tension	0	Chronic migraine	0	Clinic	0	Nerve	0
1502370	Gene	0	Relief	0	Medical	0	Nose	0	Thought	0	Nausea	0	Diet	0	best	0	Current	1	Stress	0	Sick	0	ill	0	Hospital	0	Friend	0	Botox	0	Drugs	0	Tension	0	Chronic migraine	0	Clinic	0	Nerve	1
1502371	Gene	1	Relief	0	Medical	2	Nose	0	Thought	1	Nausea	0	Diet	0	best	0	Current	0	Stress	0	Sick	0	ill	1	Hospital	0	Friend	0	Botox	3	Drugs	0	Tension	0	Chronic migraine	4	Clinic	1	Nerve	0
1502372	Gene	1	Relief	0	Medical	2	Nose	0	Thought	1	Nausea	5	Diet	1	best	1	Current	1	Stress	5	Sick	0	ill	0	Hospital	0	Friend	0	Botox	0	Drugs	0	Tension	1	Chronic migraine	0	Clinic	3	Nerve	0
1502374	Gene	1	Relief	0	Medical	2	Nose	0	Thought	3	Nausea	0	Diet	0	best	0	Current	6	Stress	1	Sick	1	ill	4	Hospital	0	Friend	1	Botox	0	Drugs	0	Tension	0	Chronic migraine	3	Clinic	0	Nerve	17
1502375	Gene	0	Relief	0	Medical	0	Nose	0	Thought	0	Nausea	0	Diet	0	best	0	Current	0	Stress	0	Sick	0	ill	0	Hospital	0	Friend	0	Botox	0	Drugs	0	Tension	0	Chronic migraine	0	Clinic	0	Nerve	0
1502380	Gene	0	Relief	0	Medical	0	Nose	0	Thought	0	Nausea	0	Diet	0	best	0	Current	0	Stress	0	Sick	0	ill	0	Hospital	0	Friend	0	Botox	0	Drugs	0	Tension	0	Chronic migraine	0	Clinic	0	Nerve	0
		93		86		83		81		81		79		68		67		61		59		59		58		54		50		49		47		45		45		43		42

URL Id	P44a	P44b	P45a	P45b	P46a	P46b	P47a	P47b	P48a	P48b	P49a	P49b	P50a	P50b	P51a	P51b	P52a	P52b	P53a	P53b	P54a	P54b	P55a	P55b	P56a	P56b	P57a	P57b	P58a	P58b	P59a	P59b	P60a	P60b	P61a	P61b	P62a
1502221	Prescription	0	Magnesium	0	Sinus	0	past	0	manage	3	Happy	0	Migraine attack	0	Acupuncture	0	Frequency	0	Stroke	1	Illness	3	Therapy	0	Cluster	1	Hormone	0	exercise	0	Exercise	0	worst	0	Injection	0	Cope
1502222	Prescription	0	Magnesium	0	Sinus	0	past	1	manage	0	Happy	0	Migraine attack	0	Acupuncture	0	Frequency	0	Stroke	0	Illness	0	Therapy	0	Cluster	0	Hormone	0	exercise	0	Exercise	0	worst	0	Injection	0	Cope
1502223	Prescription	0	Magnesium	0	Sinus	0	past	1	manage	0	Happy	0	Migraine attack	0	Acupuncture	0	Frequency	0	Stroke	0	Illness	0	Therapy	0	Cluster	0	Hormone	0	exercise	0	Exercise	0	worst	0	Injection	0	Cope
1502227	Prescription	0	Magnesium	0	Sinus	0	past	0	manage	0	Happy	0	Migraine attack	0	Acupuncture	0	Frequency	0	Stroke	0	Illness	0	Therapy	0	Cluster	0	Hormone	0	exercise	0	Exercise	0	worst	0	Injection	0	Cope
1502228	Prescription	0	Magnesium	0	Sinus	0	past	0	manage	0	Happy	0	Migraine attack	0	Acupuncture	0	Frequency	0	Stroke	0	Illness	1	Therapy	0	Cluster	0	Hormone	0	exercise	0	Exercise	0	worst	0	Injection	0	Cope
1502231	Prescription	0	Magnesium	0	Sinus	0	past	0	manage	2	Happy	0	Migraine attack	0	Acupuncture	0	Frequency	1	Stroke	0	Illness	0	Therapy	0	Cluster	0	Hormone	1	exercise	0	Exercise	0	worst	0	Injection	0	Cope
1502232	Prescription	0	Magnesium	0	Sinus	0	past	0	manage	1	Happy	0	Migraine attack	0	Acupuncture	2	Frequency	0	Stroke	0	Illness	0	Therapy	0	Cluster	0	Hormone	0	exercise	0	Exercise	0	worst	0	Injection	0	Cope
1502238	Prescription	0	Magnesium	0	Sinus	0	past	0	manage	0	Happy	0	Migraine attack	1	Acupuncture	0	Frequency	0	Stroke	0	Illness	0	Therapy	0	Cluster	0	Hormone	1	exercise	0	Exercise	0	worst	0	Injection	0	Cope
1502237	Prescription	1	Magnesium	1	Sinus	0	past	6	manage	1	Happy	0	Migraine attack	0	Acupuncture	0	Frequency	1	Stroke	2	Illness	0	Therapy	0	Cluster	0	Hormone	4	exercise	2	Exercise	2	worst	0	Injection	0	Cope
1502241	Prescription	0	Magnesium	0	Sinus	0	past	0	manage	0	Happy	0	Migraine attack	0	Acupuncture	0	Frequency	0	Stroke	0	Illness	0	Therapy	0	Cluster	0	Hormone	0	exercise	0	Exercise	0	worst	0	Injection	0	Cope
1502243	Prescription	1	Magnesium	0	Sinus	0	past	3	manage	0	Happy	0	Migraine attack	2	Acupuncture	0	Frequency	0	Stroke	2	Illness	0	Therapy	0	Cluster	0	Hormone	0	exercise	1	Exercise	1	worst	1	Injection	0	Cope
1502245	Prescription	0	Magnesium	1	Sinus	0	past	0	manage	0	Happy	0	Migraine attack	2	Acupuncture	0	Frequency	1	Stroke	0	Illness	0	Therapy	0	Cluster	0	Hormone	0	exercise	1	Exercise	1	worst	0	Injection	0	Cope
1502246	Prescription	0	Magnesium	0	Sinus	0	past	0	manage	0	Happy	0	Migraine attack	3	Acupuncture	0	Frequency	1	Stroke	0	Illness	0	Therapy	1	Cluster	0	Hormone	0	exercise	0	Exercise	0	worst	0	Injection	0	Cope
1502248	Prescription	0	Magnesium	0	Sinus	0	past	5	manage	2	Happy	0	Migraine attack	2	Acupuncture	0	Frequency	0	Stroke	2	Illness	0	Therapy	0	Cluster	0	Hormone	0	exercise	0	Exercise	0	worst	1	Injection	1	Cope
1502251	Prescription	1	Magnesium	1	Sinus	0	past	2	manage	2	Happy	0	Migraine attack	0	Acupuncture	0	Frequency	0	Stroke	0	Illness	0	Therapy	0	Cluster	0	Hormone	0	exercise	0	Exercise	0	worst	1	Injection	0	Cope
1502253	Prescription	0	Magnesium	0	Sinus	0	past	0	manage	0	Happy	0	Migraine attack	0	Acupuncture	0	Frequency	0	Stroke	0	Illness	0	Therapy	2	Cluster	0	Hormone	3	exercise	0	Exercise	0	worst	2	Injection	0	Cope
1502254	Prescription	0	Magnesium	0	Sinus	0	past	0	manage	1	Happy	0	Migraine attack	1	Acupuncture	0	Frequency	0	Stroke	2	Illness	0	Therapy	0	Cluster	0	Hormone	0	exercise	0	Exercise	0	worst	0	Injection	0	Cope
1502256	Prescription	3	Magnesium	1	Sinus	0	past	3	manage	1	Happy	0	Migraine attack	2	Acupuncture	0	Frequency	2	Stroke	3	Illness	4	Therapy	0	Cluster	0	Hormone	3	exercise	0	Exercise	0	worst	0	Injection	0	Cope
1502257	Prescription	0	Magnesium	0	Sinus	0	past	0	manage	0	Happy	0	Migraine attack	0	Acupuncture	0	Frequency	0	Stroke	0	Illness	0	Therapy	0	Cluster	0	Hormone	0	exercise	0	Exercise	0	worst	0	Injection	0	Cope
1502259	Prescription	0	Magnesium	0	Sinus	0	past	0	manage	1	Happy	0	Migraine attack	0	Acupuncture	0	Frequency	0	Stroke	0	Illness	0	Therapy	0	Cluster	0	Hormone	0	exercise	0	Exercise	0	worst	0	Injection	0	Cope
1502263	Prescription	0	Magnesium	1	Sinus	0	past	0	manage	0	Happy	0	Migraine attack	0	Acupuncture	0	Frequency	0	Stroke	0	Illness	0	Therapy	1	Cluster	1	Hormone	0	exercise	0	Exercise	0	worst	0	Injection	2	Cope
1502268	Prescription	0	Magnesium	0	Sinus	0	past	0	manage	1	Happy	0	Migraine attack	6	Acupuncture	0	Frequency	0	Stroke	0	Illness	0	Therapy	0	Cluster	0	Hormone	0	exercise	0	Exercise	0	worst	0	Injection	0	Cope
1502270	Prescription	5	Magnesium	0	Sinus	0	past	0	manage	0	Happy	0	Migraine attack	1	Acupuncture	0	Frequency	0	Stroke	0	Illness	0	Therapy	0	Cluster	0	Hormone	3	exercise	0	Exercise	0	worst	0	Injection	0	Cope
1502274	Prescription	0	Magnesium	0	Sinus	0	past	0	manage	0	Happy	0	Migraine attack	0	Acupuncture	0	Frequency	0	Stroke	0	Illness	0	Therapy	0	Cluster	0	Hormone	0	exercise	0	Exercise	0	worst	0	Injection	0	Cope
1502275	Prescription	0	Magnesium	0	Sinus	0	past	1	manage	0	Happy	1	Migraine attack	0	Acupuncture	0	Frequency	1	Stroke	0	Illness	0	Therapy	0	Cluster	1	Hormone	0	exercise	0	Exercise	0	worst	0	Injection	0	Cope
1502276	Prescription	0	Magnesium	0	Sinus	0	past	0	manage	1	Happy	0	Migraine attack	0	Acupuncture	0	Frequency	0	Stroke	0	Illness	0	Therapy	0	Cluster	0	Hormone	0	exercise	0	Exercise	0	worst	0	Injection	0	Cope
1502278	Prescription	0	Magnesium	0	Sinus	0	past	0	manage	0	Happy	0	Migraine attack	0	Acupuncture	0	Frequency	0	Stroke	0	Illness	1	Therapy	0	Cluster	0	Hormone	0	exercise	0	Exercise	0	worst	0	Injection	0	Cope
1502284	Prescription	8	Magnesium	0	Sinus	2	past	2	manage	1	Happy	0	Migraine attack	2	Acupuncture	0	Frequency	10	Stroke	0	Illness	13	Therapy	0	Cluster	5	Hormone	0	exercise	1	Exercise	1	worst	0	Injection	0	Cope
1502287	Prescription	2	Magnesium	0	Sinus	2	past	1	manage	0	Happy	0	Migraine attack	0	Acupuncture	0	Frequency	0	Stroke	0	Illness	0	Therapy	0	Cluster	0	Hormone	0	exercise	0	Exercise	0	worst	2	Injection	0	Cope
1502291	Prescription	0	Magnesium	2	Sinus	6	past	1	manage	0	Happy	2	Migraine attack	0	Acupuncture	3	Frequency	0	Stroke	0	Illness	0	Therapy	1	Cluster	2	Hormone	0	exercise	1	Exercise	1	worst	0	Injection	0	Cope
1502292	Prescription	0	Magnesium	0	Sinus	0	past	0	manage	0	Happy	0	Migraine attack	0	Acupuncture	0	Frequency	0	Stroke	0	Illness	0	Therapy	0	Cluster	0	Hormone	0	exercise	0	Exercise	0	worst	0	Injection	0	Cope
1502294	Prescription	0	Magnesium	0	Sinus	0	past	0	manage	0	Happy	1	Migraine attack	0	Acupuncture	0	Frequency	0	Stroke	0	Illness	0	Therapy	0	Cluster	0	Hormone	0	exercise	0	Exercise	0	worst	0	Injection	0	Cope
1502296	Prescription	0	Magnesium	0	Sinus	0	past	0	manage	1	Happy	0	Migraine attack	0	Acupuncture	0	Frequency	1	Stroke	0	Illness	0	Therapy	0	Cluster	0	Hormone	0	exercise	0	Exercise	0	worst	0	Injection	0	Cope
1502297	Prescription	1	Magnesium	0	Sinus	2	past	0	manage	0	Happy	0	Migraine attack	0	Acupuncture	0	Frequency	0	Stroke	0	Illness	0	Therapy	0	Cluster	0	Hormone	0	exercise	0	Exercise	0	worst	0	Injection	0	Cope
1502301	Prescription	9	Magnesium	11	Sinus	19	past	8	manage	3	Happy	26	Migraine attack	4	Acupuncture	17	Frequency	12	Stroke	14	Illness	4	Therapy	18	Cluster	0	Hormone	14	exercise	18	Exercise	18	worst	20	Injection	22	Cope
1502305	Prescription	0	Magnesium	0	Sinus	0	past	0	manage	0	Happy	0	Migraine attack	0	Acupuncture	0	Frequency	0	Stroke	0	Illness	0	Therapy	0	Cluster	1	Hormone	0	exercise	0	Exercise	0	worst	0	Injection	0	Cope
1502318	Prescription	2	Magnesium	0	Sinus	0	past	0	manage	0	Happy	0	Migraine attack	0	Acupuncture	0	Frequency	0	Stroke	0	Illness	3	Therapy	0	Cluster	0	Hormone	1	exercise	2	Exercise	2	worst	0	Injection	0	Cope
1502320	Prescription	0	Magnesium	0	Sinus	0	past	0	manage	0	Happy	0	Migraine attack	0	Acupuncture	0	Frequency	0	Stroke	0	Illness	0	Therapy	0	Cluster	0	Hormone	0	exercise	1	Exercise	1	worst	0	Injection	0	Cope
1502321	Prescription	5	Magnesium	9	Sinus	0	past	0	manage	2	Happy	0	Migraine attack	1	Acupuncture	10	Frequency	5	Stroke	3	Illness	0	Therapy	5	Cluster	0	Hormone	0	exercise	1	Exercise	1	worst	0	Injection	2	Cope
1502322	Prescription	0	Magnesium	0	Sinus	0	past	0	manage	0	Happy	0	Migraine attack	0	Acupuncture	0	Frequency	0	Stroke	1	Illness	0	Therapy	0	Cluster	0	Hormone	0	exercise	0	Exercise	0	worst	0	Injection	0	Cope
1502325	Prescription	0	Magnesium	0	Sinus	0	past	0	manage	0	Happy	0	Migraine attack	0	Acupuncture	0	Frequency	0	Stroke	0	Illness	0	Therapy	0	Cluster	0	Hormone	0	exercise	0	Exercise	0	worst	0	Injection	0	Cope
1502327	Prescription	1	Magnesium	7	Sinus	0	past	0	manage	0	Happy	0	Migraine attack	0	Acupuncture	1	Frequency	1	Stroke	0	Illness	0	Therapy	0	Cluster	0	Hormone	0	exercise	0	Exercise	0	worst	0	Injection	0	Cope
1502330	Prescription	0	Magnesium	1	Sinus	0	past	0	manage	2	Happy	1	Migraine attack	0	Acupuncture	2	Frequency	0	Stroke	0	Illness	0	Therapy	2	Cluster	0	Hormone	1	exercise	0	Exercise	0	worst	0	Injection	0	Cope
1502331	Prescription	0	Magnesium	0	Sinus	0	past	0	manage	0	Happy	0	Migraine attack	0	Acupuncture	0	Frequency	0	Stroke	0	Illness	0	Therapy	1	Cluster	4	Hormone	0	exercise	0	Exercise	0	worst	2	Injection	0	Cope
1502332	Prescription	0	Magnesium	0	Sinus	0	past	0	manage	0	Happy	0	Migraine attack	0	Acupuncture	0	Frequency	0	Stroke	0	Illness	0	Therapy	0	Cluster	0	Hormone	0	exercise	0	Exercise	0	worst	0	Injection	0	Cope
1502333	Prescription	0	Magnesium	0	Sinus	0	past	0	manage	1	Happy	0	Migraine attack	0	Acupuncture	0	Frequency	0	Stroke	0	Illness	0	Therapy	0	Cluster	0	Hormone	0	exercise	0	Exercise	0	worst	0	Injection	0	Cope
1502334	Prescription	0	Magnesium	0	Sinus	0	past	0	manage	0	Happy	0	Migraine attack	0	Acupuncture	0	Frequency	0	Stroke	0	Illness	0	Therapy	1	Cluster	0	Hormone	0	exercise	0	Exercise	0	worst	0	Injection	0	Cope
1502336	Prescription	0	Magnesium	0	Sinus	0	past	0	manage	0	Happy	0	Migraine attack	0	Acupuncture	0	Frequency	0	Stroke	0	Illness	0	Therapy	1	Cluster	0	Hormone	0	exercise	0	Exercise	0	worst	0	Injection	0	Cope
1502338	Prescription	0	Magnesium	0	Sinus	0	past	0	manage	0	Happy	0	Migraine attack	0	Acupuncture	0	Frequency	0	Stroke	0	Illness	0	Therapy	0	Cluster	0	Hormone	0	exercise	0	Exercise	0	worst	0	Injection	0	Cope
1502341	Prescription	0	Magnesium	0	Sinus	0	past	0	manage	1	Happy	0	Migraine attack	0	Acupuncture	0	Frequency	0	Stroke	0	Illness	0	Therapy	0	Cluster	1	Hormone	0	exercise	0	Exercise	0	worst	0	Injection	0	Cope
1502346	Prescription	0	Magnesium	1	Sinus	0	past	0	manage	0	Happy	4	Migraine attack	0	Acupuncture	0	Frequency	0	Stroke	0	Illness	0	Therapy	0	Cluster	0	Hormone	0	exercise	2	Exercise	2	worst	0	Injection	0	Cope
1502348	Prescription	0	Magnesium	0	Sinus	2	past	4	manage	0	Happy	0	Migraine attack	0	Acupuncture	0	Frequency	0	Stroke	0	Illness	0	Therapy	0	Cluster	2	Hormone	0	exercise	0	Exercise	0	worst	1	Injection	0	Cope
1502349	Prescription	0	Magnesium	0	Sinus	0	past	0	manage	0	Happy	0	Migraine attack	0	Acupuncture	0	Frequency	0	Stroke	0	Illness	0	Therapy	0	Cluster	0	Hormone	0	exercise	0	Exercise	0	worst	0	Injection	0	Cope
1502355	Prescription	0	Magnesium	0	Sinus	0	past	1	manage	2	Happy	1	Migraine attack	0	Acupuncture	0	Frequency	0	Stroke	1	Illness	0	Therapy	1	Cluster	3	Hormone	1	exercise	0	Exercise	0	worst	1	Injection	0	Cope
1502358	Prescription	1	Magnesium	2	Sinus	7	past	1	manage	0	Happy	0	Migraine attack	0	Acupuncture	2	Frequency	0	Stroke	4	Illness	2	Therapy	0	Cluster	1	Hormone	2	exercise	2	Exercise	2	worst	1	Injection	1	Cope
1502359	Prescription	0	Magnesium	0	Sinus	0	past	0	manage	0	Happy	0	Migraine attack	0	Acupuncture	0	Frequency	0	Stroke	0	Illness	0	Therapy	0	Cluster	0	Hormone	0	exercise	0	Exercise	0	worst	0	Injection	0	Cope
1502362	Prescription	0	Magnesium	0	Sinus	0	past	0	manage	7	Happy	1	Migraine attack	0	Acupuncture	0	Frequency	0	Stroke	0	Illness	0	Therapy	1	Cluster	0	Hormone	0	exercise	0	Exercise	0	worst	0	Injection	1	Cope
1502369	Prescription	0	Magnesium	3	Sinus	0	past	0	manage	0	Happy	0	Migraine attack	0	Acupuncture	0	Frequency	0	Stroke	1	Illness	0	Therapy	0	Cluster	0	Hormone	0	exercise	0	Exercise	0	worst	0	Injection	0	Cope
1502370	Prescription	0	Magnesium	0	Sinus	0	past	0	manage	0	Happy	0	Migraine attack	0	Acupuncture	0	Frequency	0	Stroke	0	Illness	0	Therapy	0	Cluster	0	Hormone	0	exercise	0	Exercise	0	worst	0	Injection	0	Cope
1502371	Prescription	0	Magnesium	0	Sinus	0	past	0	manage	2	Happy	0	Migraine attack	9	Acupuncture	0	Frequency	0	Stroke	0	Illness	1	Therapy	0	Cluster	0	Hormone	0	exercise	0	Exercise	0	worst	0	Injection	0	Cope
1502372	Prescription	0	Magnesium	0	Sinus	0	past	0	manage	1	Happy	0	Migraine attack	0	Acupuncture	0	Frequency	0	Stroke	0	Illness	0	Therapy	0	Cluster	0	Hormone	0	exercise	0	Exercise	0	worst	0	Injection	0	Cope
1502374	Prescription	2	Magnesium	0	Sinus	0	past	0	manage	0	Happy	1	Migraine attack	2	Acupuncture	0	Frequency	1	Stroke	0	Illness	4	Therapy	0	Cluster	13	Hormone	0	exercise	1	Exercise	1	worst	0	Injection	0	Cope
1502375	Prescription	0	Magnesium	0	Sinus	0	past	0	manage	0	Happy	0	Migraine attack	0	Acupuncture	0	Frequency	0	Stroke	0	Illness	0	Therapy	0	Cluster	0	Hormone	0	exercise	0	Exercise	0	worst	0	Injection	0	Cope
1502380	Prescription	0	Magnesium	0	Sinus	0	past	0	manage	0	Happy	0	Migraine attack	0	Acupuncture	0	Frequency	0	Stroke	0	Illness	0	Therapy	0	Cluster	0	Hormone	0	exercise	0	Exercise	0	worst	0	Injection	0	Cope
		42		41		40		40		39		38		38		37		37		37		36		35		35		34		33		33		32		29	

P62b	P63a	P63b	P64a	P64b	P65a	P65b	P66a	P66b	P67a	P67b	P68a	P68b	P69a	P69b	P70a	P70b	P71a	P71b
0	Herbal medicine	0	Onset	0	Cluster headache	1	Mother	1	sickness	0	Chronic pain	1	Serotonin	0	Blocker	0	husband	0
0	Herbal medicine	0	Onset	0	Cluster headache	0	Mother	0	sickness	0	Chronic pain	0	Serotonin	0	Blocker	0	husband	0
0	Herbal medicine	0	Onset	0	Cluster headache	0	Mother	1	sickness	0	Chronic pain	0	Serotonin	0	Blocker	0	husband	0
0	Herbal medicine	0	Onset	0	Cluster headache	0	Mother	0	sickness	0	Chronic pain	0	Serotonin	0	Blocker	0	husband	0
0	Herbal medicine	0	Onset	0	Cluster headache	0	Mother	0	sickness	0	Chronic pain	5	Serotonin	0	Blocker	0	husband	0
0	Herbal medicine	0	Onset	0	Cluster headache	0	Mother	0	sickness	0	Chronic pain	0	Serotonin	0	Blocker	0	husband	0
0	Herbal medicine	0	Onset	0	Cluster headache	0	Mother	0	sickness	0	Chronic pain	0	Serotonin	0	Blocker	0	husband	0
0	Herbal medicine	0	Onset	0	Cluster headache	0	Mother	0	sickness	0	Chronic pain	0	Serotonin	0	Blocker	0	husband	0
1	Herbal medicine	0	Onset	2	Cluster headache	0	Mother	1	sickness	0	Chronic pain	1	Serotonin	0	Blocker	0	husband	1
0	Herbal medicine	0	Onset	0	Cluster headache	0	Mother	0	sickness	0	Chronic pain	0	Serotonin	0	Blocker	0	husband	0
0	Herbal medicine	0	Onset	1	Cluster headache	0	Mother	1	sickness	0	Chronic pain	0	Serotonin	0	Blocker	0	husband	1
0	Herbal medicine	0	Onset	0	Cluster headache	0	Mother	0	sickness	0	Chronic pain	0	Serotonin	0	Blocker	0	husband	1
0	Herbal medicine	0	Onset	2	Cluster headache	0	Mother	0	sickness	0	Chronic pain	0	Serotonin	0	Blocker	0	husband	0
0	Herbal medicine	0	Onset	0	Cluster headache	0	Mother	0	sickness	0	Chronic pain	0	Serotonin	0	Blocker	0	husband	1
0	Herbal medicine	0	Onset	1	Cluster headache	0	Mother	2	sickness	0	Chronic pain	0	Serotonin	0	Blocker	0	husband	0
0	Herbal medicine	0	Onset	0	Cluster headache	0	Mother	0	sickness	0	Chronic pain	0	Serotonin	0	Blocker	2	husband	0
0	Herbal medicine	0	Onset	0	Cluster headache	0	Mother	0	sickness	0	Chronic pain	0	Serotonin	0	Blocker	0	husband	0
6	Herbal medicine	0	Onset	0	Cluster headache	0	Mother	3	sickness	0	Chronic pain	0	Serotonin	0	Blocker	0	husband	0
0	Herbal medicine	0	Onset	0	Cluster headache	0	Mother	0	sickness	0	Chronic pain	0	Serotonin	0	Blocker	0	husband	0
0	Herbal medicine	0	Onset	0	Cluster headache	0	Mother	0	sickness	0	Chronic pain	0	Serotonin	0	Blocker	0	husband	0
0	Herbal medicine	0	Onset	0	Cluster headache	1	Mother	0	sickness	0	Chronic pain	0	Serotonin	0	Blocker	3	husband	0
0	Herbal medicine	0	Onset	0	Cluster headache	0	Mother	0	sickness	0	Chronic pain	0	Serotonin	0	Blocker	0	husband	0
0	Herbal medicine	0	Onset	0	Cluster headache	0	Mother	0	sickness	0	Chronic pain	0	Serotonin	0	Blocker	0	husband	0
0	Herbal medicine	0	Onset	0	Cluster headache	0	Mother	0	sickness	0	Chronic pain	0	Serotonin	0	Blocker	0	husband	0
0	Herbal medicine	0	Onset	1	Cluster headache	1	Mother	0	sickness	0	Chronic pain	15	Serotonin	0	Blocker	0	husband	0
0	Herbal medicine	0	Onset	0	Cluster headache	0	Mother	0	sickness	0	Chronic pain	0	Serotonin	0	Blocker	0	husband	0
0	Herbal medicine	0	Onset	0	Cluster headache	0	Mother	0	sickness	0	Chronic pain	0	Serotonin	0	Blocker	0	husband	0
0	Herbal medicine	0	Onset	3	Cluster headache	3	Mother	0	sickness	0	Chronic pain	0	Serotonin	0	Blocker	0	husband	0
0	Herbal medicine	0	Onset	1	Cluster headache	0	Mother	1	sickness	6	Chronic pain	0	Serotonin	0	Blocker	0	husband	4
0	Herbal medicine	0	Onset	0	Cluster headache	2	Mother	0	sickness	0	Chronic pain	0	Serotonin	0	Blocker	0	husband	0
0	Herbal medicine	0	Onset	0	Cluster headache	0	Mother	0	sickness	0	Chronic pain	0	Serotonin	0	Blocker	0	husband	0
0	Herbal medicine	0	Onset	0	Cluster headache	0	Mother	2	sickness	0	Chronic pain	0	Serotonin	0	Blocker	0	husband	0
0	Herbal medicine	0	Onset	0	Cluster headache	0	Mother	0	sickness	0	Chronic pain	0	Serotonin	1	Blocker	0	husband	0
0	Herbal medicine	0	Onset	0	Cluster headache	0	Mother	0	sickness	0	Chronic pain	0	Serotonin	0	Blocker	0	husband	0
17	Herbal medicine	28	Onset	9	Cluster headache	0	Mother	6	sickness	18	Chronic pain	0	Serotonin	0	Blocker	3	husband	12
1	Herbal medicine	0	Onset	0	Cluster headache	1	Mother	0	sickness	0	Chronic pain	0	Serotonin	0	Blocker	0	husband	0
0	Herbal medicine	0	Onset	0	Cluster headache	0	Mother	0	sickness	0	Chronic pain	1	Serotonin	1	Blocker	0	husband	0
0	Herbal medicine	0	Onset	0	Cluster headache	0	Mother	0	sickness	0	Chronic pain	0	Serotonin	0	Blocker	0	husband	0
1	Herbal medicine	0	Onset	1	Cluster headache	0	Mother	1	sickness	0	Chronic pain	0	Serotonin	7	Blocker	7	husband	0
0	Herbal medicine	0	Onset	0	Cluster headache	0	Mother	0	sickness	0	Chronic pain	0	Serotonin	0	Blocker	1	husband	0
0	Herbal medicine	0	Onset	0	Cluster headache	0	Mother	0	sickness	0	Chronic pain	0	Serotonin	0	Blocker	0	husband	0
0	Herbal medicine	0	Onset	0	Cluster headache	0	Mother	0	sickness	0	Chronic pain	0	Serotonin	0	Blocker	1	husband	0
0	Herbal medicine	0	Onset	0	Cluster headache	0	Mother	0	sickness	0	Chronic pain	0	Serotonin	0	Blocker	0	husband	0
0	Herbal medicine	0	Onset	1	Cluster headache	2	Mother	0	sickness	0	Chronic pain	0	Serotonin	2	Blocker	0	husband	0
0	Herbal medicine	0	Onset	0	Cluster headache	0	Mother	0	sickness	0	Chronic pain	0	Serotonin	3	Blocker	1	husband	0
0	Herbal medicine	0	Onset	0	Cluster headache	0	Mother	0	sickness	0	Chronic pain	0	Serotonin	0	Blocker	0	husband	0
0	Herbal medicine	0	Onset	1	Cluster headache	0	Mother	3	sickness	0	Chronic pain	0	Serotonin	0	Blocker	2	husband	0
0	Herbal medicine	0	Onset	0	Cluster headache	0	Mother	0	sickness	0	Chronic pain	0	Serotonin	0	Blocker	0	husband	0
0	Herbal medicine	0	Onset	0	Cluster headache	0	Mother	0	sickness	0	Chronic pain	0	Serotonin	0	Blocker	0	husband	0
0	Herbal medicine	0	Onset	0	Cluster headache	1	Mother	0	sickness	0	Chronic pain	0	Serotonin	0	Blocker	0	husband	0
0	Herbal medicine	0	Onset	0	Cluster headache	0	Mother	0	sickness	0	Chronic pain	0	Serotonin	0	Blocker	0	husband	0
0	Herbal medicine	0	Onset	0	Cluster headache	1	Mother	0	sickness	0	Chronic pain	0	Serotonin	0	Blocker	2	husband	0
0	Herbal medicine	0	Onset	0	Cluster headache	0	Mother	0	sickness	0	Chronic pain	0	Serotonin	0	Blocker	0	husband	0
2	Herbal medicine	0	Onset	0	Cluster headache	0	Mother	1	sickness	1	Chronic pain	0	Serotonin	0	Blocker	0	husband	0
1	Herbal medicine	0	Onset	3	Cluster headache	1	Mother	2	sickness	1	Chronic pain	0	Serotonin	8	Blocker	0	husband	1
0	Herbal medicine	0	Onset	0	Cluster headache	0	Mother	0	sickness	0	Chronic pain	0	Serotonin	0	Blocker	0	husband	0
0	Herbal medicine	0	Onset	0	Cluster headache	0	Mother	1	sickness	0	Chronic pain	1	Serotonin	0	Blocker	0	husband	0
0	Herbal medicine	0	Onset	0	Cluster headache	0	Mother	1	sickness	0	Chronic pain	0	Serotonin	0	Blocker	0	husband	1
0	Herbal medicine	0	Onset	0	Cluster headache	0	Mother	0	sickness	0	Chronic pain	0	Serotonin	0	Blocker	0	husband	0
0	Herbal medicine	0	Onset	1	Cluster headache	0	Mother	0	sickness	0	Chronic pain	0	Serotonin	0	Blocker	0	husband	0
0	Herbal medicine	0	Onset	0	Cluster headache	0	Mother	0	sickness	0	Chronic pain	0	Serotonin	0	Blocker	0	husband	0
0	Herbal medicine	0	Onset	0	Cluster headache	13	Mother	0	sickness	0	Chronic pain	1	Serotonin	0	Blocker	0	husband	0
0	Herbal medicine	0	Onset	0	Cluster headache	0	Mother	0	sickness	0	Chronic pain	0	Serotonin	0	Blocker	0	husband	0
0	Herbal medicine	0	Onset	0	Cluster headache	0	Mother	0	sickness	0	Chronic pain	0	Serotonin	0	Blocker	0	husband	0
29		28		27		27		27		26		25		22		22		22

P72a	P72b	P73a	P73b	P74a	P74b	P75a	P75b	P76a	P76b	P77a	P77b	P78a	P78b	P79a	P79b	P80a	P80b	P81a	P81b
Management	2	Daughter	0	Walk	0	Child	3	nasal	0	Depression	2	Prophylactic	0	Pregnancy	0	pray	0	Acetaminophen	1
Management	0	Daughter	0	Walk	0	Child	0	nasal	0	Depression	0	Prophylactic	0	Pregnancy	0	pray	0	Acetaminophen	0
Management	0	Daughter	0	Walk	0	Child	0	nasal	0	Depression	0	Prophylactic	0	Pregnancy	0	pray	0	Acetaminophen	0
Management	0	Daughter	0	Walk	0	Child	0	nasal	0	Depression	0	Prophylactic	0	Pregnancy	0	pray	0	Acetaminophen	0
Management	0	Daughter	0	Walk	0	Child	0	nasal	0	Depression	2	Prophylactic	0	Pregnancy	0	pray	0	Acetaminophen	0
Management	1	Daughter	0	Walk	0	Child	0	nasal	0	Depression	0	Prophylactic	0	Pregnancy	0	pray	0	Acetaminophen	0
Management	0	Daughter	0	Walk	0	Child	0	nasal	0	Depression	0	Prophylactic	0	Pregnancy	0	pray	0	Acetaminophen	0
Management	0	Daughter	3	Walk	2	Child	0	nasal	0	Depression	1	Prophylactic	0	Pregnancy	0	pray	2	Acetaminophen	0
Management	0	Daughter	0	Walk	0	Child	1	nasal	0	Depression	0	Prophylactic	0	Pregnancy	0	pray	0	Acetaminophen	0
Management	0	Daughter	0	Walk	0	Child	0	nasal	0	Depression	0	Prophylactic	0	Pregnancy	0	pray	0	Acetaminophen	0
Management	0	Daughter	4	Walk	0	Child	0	nasal	0	Depression	0	Prophylactic	0	Pregnancy	0	pray	0	Acetaminophen	0
Management	0	Daughter	0	Walk	0	Child	0	nasal	0	Depression	0	Prophylactic	0	Pregnancy	0	pray	0	Acetaminophen	0
Management	0	Daughter	0	Walk	0	Child	1	nasal	4	Depression	0	Prophylactic	0	Pregnancy	0	pray	0	Acetaminophen	0
Management	0	Daughter	1	Walk	1	Child	0	nasal	0	Depression	0	Prophylactic	0	Pregnancy	0	pray	0	Acetaminophen	0
Management	2	Daughter	3	Walk	0	Child	0	nasal	0	Depression	0	Prophylactic	0	Pregnancy	3	pray	0	Acetaminophen	2
Management	0	Daughter	0	Walk	2	Child	0	nasal	0	Depression	0	Prophylactic	0	Pregnancy	1	pray	0	Acetaminophen	0
Management	1	Daughter	0	Walk	0	Child	0	nasal	0	Depression	0	Prophylactic	0	Pregnancy	0	pray	0	Acetaminophen	0
Management	0	Daughter	0	Walk	0	Child	1	nasal	0	Depression	0	Prophylactic	1	Pregnancy	0	pray	0	Acetaminophen	0
Management	0	Daughter	0	Walk	0	Child	0	nasal	0	Depression	0	Prophylactic	0	Pregnancy	0	pray	0	Acetaminophen	0
Management	0	Daughter	0	Walk	0	Child	0	nasal	0	Depression	0	Prophylactic	0	Pregnancy	0	pray	0	Acetaminophen	0
Management	0	Daughter	3	Walk	0	Child	0	nasal	1	Depression	0	Prophylactic	0	Pregnancy	0	pray	0	Acetaminophen	0
Management	1	Daughter	0	Walk	0	Child	1	nasal	0	Depression	0	Prophylactic	0	Pregnancy	0	pray	0	Acetaminophen	0
Management	0	Daughter	0	Walk	0	Child	0	nasal	0	Depression	0	Prophylactic	0	Pregnancy	0	pray	0	Acetaminophen	3
Management	0	Daughter	0	Walk	0	Child	0	nasal	0	Depression	0	Prophylactic	0	Pregnancy	0	pray	0	Acetaminophen	0
Management	0	Daughter	0	Walk	1	Child	0	nasal	0	Depression	0	Prophylactic	0	Pregnancy	0	pray	0	Acetaminophen	0
Management	0	Daughter	0	Walk	0	Child	0	nasal	0	Depression	0	Prophylactic	0	Pregnancy	0	pray	0	Acetaminophen	0
Management	0	Daughter	0	Walk	0	Child	0	nasal	0	Depression	0	Prophylactic	0	Pregnancy	0	pray	0	Acetaminophen	0
Management	0	Daughter	0	Walk	0	Child	0	nasal	0	Depression	0	Prophylactic	0	Pregnancy	0	pray	0	Acetaminophen	0
Management	0	Daughter	2	Walk	0	Child	1	nasal	0	Depression	0	Prophylactic	1	Pregnancy	0	pray	1	Acetaminophen	0
Management	0	Daughter	0	Walk	1	Child	1	nasal	0	Depression	1	Prophylactic	0	Pregnancy	0	pray	0	Acetaminophen	0
Management	0	Daughter	0	Walk	0	Child	0	nasal	0	Depression	0	Prophylactic	0	Pregnancy	0	pray	0	Acetaminophen	0
Management	0	Daughter	0	Walk	0	Child	0	nasal	0	Depression	0	Prophylactic	0	Pregnancy	0	pray	0	Acetaminophen	0
Management	0	Daughter	0	Walk	1	Child	2	nasal	1	Depression	0	Prophylactic	0	Pregnancy	0	pray	1	Acetaminophen	0
Management	0	Daughter	0	Walk	0	Child	0	nasal	0	Depression	0	Prophylactic	0	Pregnancy	0	pray	0	Acetaminophen	0
Management	2	Daughter	2	Walk	6	Child	2	nasal	10	Depression	1	Prophylactic	4	Pregnancy	10	pray	11	Acetaminophen	4
Management	0	Daughter	0	Walk	0	Child	0	nasal	0	Depression	0	Prophylactic	0	Pregnancy	0	pray	0	Acetaminophen	0
Management	0	Daughter	0	Walk	1	Child	2	nasal	0	Depression	4	Prophylactic	0	Pregnancy	0	pray	0	Acetaminophen	0
Management	0	Daughter	0	Walk	1	Child	0	nasal	0	Depression	0	Prophylactic	0	Pregnancy	0	pray	0	Acetaminophen	0
Management	1	Daughter	2	Walk	0	Child	2	nasal	1	Depression	2	Prophylactic	13	Pregnancy	2	pray	0	Acetaminophen	0
Management	0	Daughter	0	Walk	0	Child	0	nasal	0	Depression	0	Prophylactic	0	Pregnancy	0	pray	0	Acetaminophen	0
Management	0	Daughter	0	Walk	0	Child	0	nasal	0	Depression	0	Prophylactic	0	Pregnancy	0	pray	0	Acetaminophen	0
Management	0	Daughter	0	Walk	0	Child	0	nasal	0	Depression	0	Prophylactic	0	Pregnancy	0	pray	0	Acetaminophen	0
Management	1	Daughter	0	Walk	0	Child	0	nasal	1	Depression	0	Prophylactic	0	Pregnancy	0	pray	1	Acetaminophen	0
Management	0	Daughter	0	Walk	0	Child	0	nasal	0	Depression	0	Prophylactic	0	Pregnancy	0	pray	0	Acetaminophen	0
Management	0	Daughter	0	Walk	0	Child	0	nasal	0	Depression	0	Prophylactic	0	Pregnancy	0	pray	0	Acetaminophen	0
Management	1	Daughter	0	Walk	0	Child	0	nasal	0	Depression	0	Prophylactic	0	Pregnancy	0	pray	0	Acetaminophen	0
Management	0	Daughter	0	Walk	0	Child	0	nasal	0	Depression	2	Prophylactic	0	Pregnancy	3	pray	0	Acetaminophen	0
Management	0	Daughter	0	Walk	0	Child	0	nasal	0	Depression	0	Prophylactic	0	Pregnancy	0	pray	0	Acetaminophen	0
Management	0	Daughter	0	Walk	0	Child	0	nasal	0	Depression	0	Prophylactic	0	Pregnancy	0	pray	0	Acetaminophen	0
Management	1	Daughter	0	Walk	0	Child	0	nasal	0	Depression	0	Prophylactic	0	Pregnancy	0	pray	0	Acetaminophen	0
Management	0	Daughter	0	Walk	0	Child	0	nasal	0	Depression	0	Prophylactic	0	Pregnancy	0	pray	0	Acetaminophen	0
Management	0	Daughter	0	Walk	1	Child	1	nasal	0	Depression	0	Prophylactic	0	Pregnancy	0	pray	0	Acetaminophen	0
Management	0	Daughter	0	Walk	0	Child	0	nasal	0	Depression	0	Prophylactic	0	Pregnancy	0	pray	0	Acetaminophen	0
Management	0	Daughter	1	Walk	0	Child	0	nasal	0	Depression	0	Prophylactic	0	Pregnancy	0	pray	0	Acetaminophen	0
Management	0	Daughter	0	Walk	2	Child	3	nasal	2	Depression	4	Prophylactic	0	Pregnancy	0	pray	2	Acetaminophen	0
Management	0	Daughter	0	Walk	0	Child	0	nasal	0	Depression	0	Prophylactic	0	Pregnancy	0	pray	0	Acetaminophen	0
Management	7	Daughter	0	Walk	1	Child	0	nasal	0	Depression	0	Prophylactic	0	Pregnancy	0	pray	0	Acetaminophen	0
Management	0	Daughter	0	Walk	0	Child	0	nasal	0	Depression	0	Prophylactic	0	Pregnancy	0	pray	1	Acetaminophen	0
Management	0	Daughter	0	Walk	0	Child	0	nasal	0	Depression	0	Prophylactic	0	Pregnancy	0	pray	0	Acetaminophen	0
Management	2	Daughter	0	Walk	0	Child	0	nasal	0	Depression	0	Prophylactic	0	Pregnancy	0	pray	0	Acetaminophen	0
Management	0	Daughter	0	Walk	0	Child	0	nasal	0	Depression	0	Prophylactic	0	Pregnancy	0	pray	0	Acetaminophen	9
Management	0	Daughter	0	Walk	1	Child	0	nasal	0	Depression	1	Prophylactic	0	Pregnancy	0	pray	0	Acetaminophen	0
Management	0	Daughter	0	Walk	0	Child	0	nasal	0	Depression	0	Prophylactic	0	Pregnancy	0	pray	0	Acetaminophen	0
Management	0	Daughter	0	Walk	0	Child	0	nasal	0	Depression	0	Prophylactic	0	Pregnancy	0	pray	0	Acetaminophen	0
	22		21		21		21		20		20		19		19		19		19

P82a	P82b	P83a	P83b	P84a	P84b	P85a	P85b	P86a	P86b	P87a	P87b	P88a	P88b	P89a	P89b	P90a	P90b	P91a	P91b	P92a	P92b
Diagnosis	2	Ergot	0	travel	0	Menopause	0	employ	0	Inhibitor	0	Disability	0	Neck pain	0	Present	0	Brother	0	Genetic	0
Diagnosis	0	Ergot	0	travel	0	Menopause	0	employ	0	Inhibitor	0	Disability	0	Neck pain	0	Present	0	Brother	0	Genetic	0
Diagnosis	1	Ergot	0	travel	0	Menopause	0	employ	0	Inhibitor	0	Disability	0	Neck pain	0	Present	0	Brother	0	Genetic	0
Diagnosis	0	Ergot	0	travel	0	Menopause	0	employ	0	Inhibitor	0	Disability	0	Neck pain	0	Present	0	Brother	0	Genetic	0
Diagnosis	0	Ergot	0	travel	0	Menopause	0	employ	0	Inhibitor	0	Disability	0	Neck pain	0	Present	0	Brother	0	Genetic	0
Diagnosis	0	Ergot	0	travel	0	Menopause	0	employ	4	Inhibitor	0	Disability	0	Neck pain	0	Present	0	Brother	0	Genetic	1
Diagnosis	0	Ergot	0	travel	0	Menopause	0	employ	0	Inhibitor	0	Disability	0	Neck pain	0	Present	0	Brother	0	Genetic	0
Diagnosis	0	Ergot	0	travel	0	Menopause	3	employ	0	Inhibitor	0	Disability	0	Neck pain	0	Present	0	Brother	0	Genetic	0
Diagnosis	0	Ergot	0	travel	0	Menopause	3	employ	0	Inhibitor	0	Disability	0	Neck pain	0	Present	1	Brother	1	Genetic	1
Diagnosis	0	Ergot	0	travel	0	Menopause	0	employ	0	Inhibitor	0	Disability	0	Neck pain	0	Present	0	Brother	0	Genetic	1
Diagnosis	1	Ergot	0	travel	0	Menopause	0	employ	0	Inhibitor	0	Disability	0	Neck pain	0	Present	0	Brother	0	Genetic	0
Diagnosis	0	Ergot	0	travel	0	Menopause	0	employ	0	Inhibitor	0	Disability	0	Neck pain	0	Present	0	Brother	0	Genetic	0
Diagnosis	0	Ergot	0	travel	1	Menopause	0	employ	0	Inhibitor	0	Disability	0	Neck pain	0	Present	0	Brother	0	Genetic	3
Diagnosis	1	Ergot	1	travel	0	Menopause	0	employ	0	Inhibitor	0	Disability	0	Neck pain	0	Present	0	Brother	0	Genetic	0
Diagnosis	0	Ergot	0	travel	0	Menopause	0	employ	0	Inhibitor	0	Disability	0	Neck pain	0	Present	0	Brother	0	Genetic	0
Diagnosis	1	Ergot	0	travel	0	Menopause	0	employ	0	Inhibitor	0	Disability	0	Neck pain	0	Present	0	Brother	0	Genetic	0
Diagnosis	0	Ergot	0	travel	1	Menopause	0	employ	0	Inhibitor	0	Disability	0	Neck pain	0	Present	0	Brother	0	Genetic	0
Diagnosis	1	Ergot	0	travel	0	Menopause	4	employ	0	Inhibitor	0	Disability	0	Neck pain	0	Present	2	Brother	1	Genetic	0
Diagnosis	0	Ergot	0	travel	0	Menopause	0	employ	0	Inhibitor	0	Disability	0	Neck pain	0	Present	0	Brother	0	Genetic	0
Diagnosis	0	Ergot	0	travel	0	Menopause	0	employ	1	Inhibitor	0	Disability	0	Neck pain	0	Present	0	Brother	0	Genetic	0
Diagnosis	0	Ergot	0	travel	0	Menopause	0	employ	0	Inhibitor	0	Disability	0	Neck pain	0	Present	1	Brother	0	Genetic	0
Diagnosis	0	Ergot	0	travel	0	Menopause	0	employ	0	Inhibitor	0	Disability	0	Neck pain	0	Present	0	Brother	0	Genetic	0
Diagnosis	0	Ergot	0	travel	0	Menopause	0	employ	0	Inhibitor	0	Disability	0	Neck pain	0	Present	0	Brother	1	Genetic	0
Diagnosis	0	Ergot	0	travel	0	Menopause	0	employ	0	Inhibitor	0	Disability	2	Neck pain	0	Present	0	Brother	0	Genetic	0
Diagnosis	0	Ergot	0	travel	0	Menopause	0	employ	0	Inhibitor	0	Disability	0	Neck pain	0	Present	0	Brother	0	Genetic	0
Diagnosis	0	Ergot	0	travel	0	Menopause	0	employ	0	Inhibitor	0	Disability	0	Neck pain	0	Present	0	Brother	0	Genetic	0
Diagnosis	0	Ergot	0	travel	0	Menopause	0	employ	0	Inhibitor	0	Disability	0	Neck pain	0	Present	0	Brother	0	Genetic	0
Diagnosis	0	Ergot	0	travel	0	Menopause	0	employ	10	Inhibitor	0	Disability	15	Neck pain	0	Present	0	Brother	2	Genetic	0
Diagnosis	0	Ergot	0	travel	8	Menopause	0	employ	0	Inhibitor	0	Disability	0	Neck pain	0	Present	0	Brother	0	Genetic	0
Diagnosis	0	Ergot	0	travel	1	Menopause	0	employ	0	Inhibitor	0	Disability	0	Neck pain	0	Present	0	Brother	0	Genetic	0
Diagnosis	0	Ergot	0	travel	0	Menopause	0	employ	0	Inhibitor	0	Disability	0	Neck pain	0	Present	0	Brother	0	Genetic	0
Diagnosis	0	Ergot	0	travel	0	Menopause	0	employ	0	Inhibitor	0	Disability	0	Neck pain	0	Present	0	Brother	0	Genetic	2
Diagnosis	0	Ergot	0	travel	0	Menopause	0	employ	0	Inhibitor	0	Disability	0	Neck pain	0	Present	0	Brother	0	Genetic	0
Diagnosis	0	Ergot	0	travel	0	Menopause	0	employ	0	Inhibitor	0	Disability	0	Neck pain	0	Present	0	Brother	0	Genetic	0
Diagnosis	5	Ergot	9	travel	2	Menopause	6	employ	3	Inhibitor	0	Disability	0	Neck pain	14	Present	3	Brother	4	Genetic	6
Diagnosis	0	Ergot	0	travel	0	Menopause	0	employ	0	Inhibitor	0	Disability	0	Neck pain	0	Present	0	Brother	0	Genetic	0
Diagnosis	2	Ergot	0	travel	0	Menopause	0	employ	0	Inhibitor	0	Disability	0	Neck pain	0	Present	0	Brother	0	Genetic	0
Diagnosis	0	Ergot	0	travel	0	Menopause	0	employ	0	Inhibitor	0	Disability	0	Neck pain	0	Present	0	Brother	0	Genetic	0
Diagnosis	0	Ergot	3	travel	1	Menopause	0	employ	0	Inhibitor	12	Disability	0	Neck pain	0	Present	2	Brother	0	Genetic	0
Diagnosis	0	Ergot	1	travel	0	Menopause	0	employ	0	Inhibitor	0	Disability	0	Neck pain	0	Present	0	Brother	0	Genetic	0
Diagnosis	0	Ergot	0	travel	0	Menopause	0	employ	0	Inhibitor	0	Disability	0	Neck pain	0	Present	0	Brother	0	Genetic	0
Diagnosis	0	Ergot	0	travel	1	Menopause	0	employ	0	Inhibitor	0	Disability	0	Neck pain	0	Present	0	Brother	3	Genetic	0
Diagnosis	0	Ergot	0	travel	0	Menopause	2	employ	0	Inhibitor	0	Disability	0	Neck pain	1	Present	1	Brother	0	Genetic	0
Diagnosis	0	Ergot	0	travel	0	Menopause	0	employ	0	Inhibitor	0	Disability	0	Neck pain	0	Present	0	Brother	0	Genetic	0
Diagnosis	0	Ergot	0	travel	0	Menopause	0	employ	0	Inhibitor	0	Disability	0	Neck pain	0	Present	0	Brother	0	Genetic	0
Diagnosis	0	Ergot	0	travel	0	Menopause	0	employ	0	Inhibitor	0	Disability	0	Neck pain	0	Present	0	Brother	0	Genetic	0
Diagnosis	0	Ergot	4	travel	0	Menopause	0	employ	0	Inhibitor	1	Disability	0	Neck pain	0	Present	0	Brother	0	Genetic	0
Diagnosis	2	Ergot	0	travel	0	Menopause	0	employ	0	Inhibitor	0	Disability	0	Neck pain	0	Present	0	Brother	0	Genetic	0
Diagnosis	0	Ergot	0	travel	0	Menopause	0	employ	0	Inhibitor	0	Disability	0	Neck pain	0	Present	0	Brother	0	Genetic	0
Diagnosis	0	Ergot	0	travel	0	Menopause	0	employ	0	Inhibitor	0	Disability	0	Neck pain	0	Present	1	Brother	0	Genetic	0
Diagnosis	0	Ergot	0	travel	0	Menopause	0	employ	0	Inhibitor	0	Disability	0	Neck pain	0	Present	1	Brother	4	Genetic	0
Diagnosis	2	Ergot	0	travel	0	Menopause	0	employ	0	Inhibitor	0	Disability	0	Neck pain	1	Present	1	Brother	0	Genetic	0
Diagnosis	0	Ergot	0	travel	0	Menopause	0	employ	0	Inhibitor	0	Disability	0	Neck pain	0	Present	0	Brother	0	Genetic	0
Diagnosis	0	Ergot	0	travel	0	Menopause	0	employ	0	Inhibitor	0	Disability	0	Neck pain	0	Present	0	Brother	0	Genetic	0
Diagnosis	0	Ergot	0	travel	1	Menopause	0	employ	0	Inhibitor	0	Disability	0	Neck pain	0	Present	1	Brother	0	Genetic	0
Diagnosis	1	Ergot	0	travel	0	Menopause	0	employ	0	Inhibitor	0	Disability	0	Neck pain	0	Present	0	Brother	0	Genetic	0
Diagnosis	0	Ergot	0	travel	0	Menopause	0	employ	0	Inhibitor	0	Disability	0	Neck pain	0	Present	0	Brother	0	Genetic	0
Diagnosis	0	Ergot	0	travel	0	Menopause	0	employ	0	Inhibitor	0	Disability	0	Neck pain	0	Present	0	Brother	0	Genetic	0
Diagnosis	0	Ergot	0	travel	0	Menopause	0	employ	0	Inhibitor	0	Disability	0	Neck pain	0	Present	0	Brother	0	Genetic	0
Diagnosis	0	Ergot	0	travel	0	Menopause	0	employ	0	Inhibitor	0	Disability	0	Neck pain	0	Present	0	Brother	0	Genetic	1
Diagnosis	0	Ergot	0	travel	0	Menopause	0	employ	0	Inhibitor	0	Disability	0	Neck pain	0	Present	0	Brother	0	Genetic	0
Diagnosis	0	Ergot	0	travel	2	Menopause	0	employ	0	Inhibitor	4	Disability	0	Neck pain	0	Present	2	Brother	0	Genetic	0
Diagnosis	0	Ergot	0	travel	0	Menopause	0	employ	0	Inhibitor	0	Disability	0	Neck pain	0	Present	0	Brother	0	Genetic	0
Diagnosis	0	Ergot	0	travel	0	Menopause	0	employ	0	Inhibitor	0	Disability	0	Neck pain	0	Present	0	Brother	0	Genetic	0
	19		18		18		18		18		17		17		16		16		16		15

P93a	P93b	P94a	P94b	P95a	P95b	P96a	P96b	P97a	P97b	P98a	P98b	P99a	P99b	P100a	P100b	P101a	P101b
Education	5	Parent	1	Depressed	0	Visual disturbance	0	ARB	0	Photophobia	0	Support group	0	Abortive	0	Sister	0
Education	0	Parent	0	Depressed	0	Visual disturbance	0	ARB	0	Photophobia	0	Support group	0	Abortive	0	Sister	0
Education	0	Parent	0	Depressed	0	Visual disturbance	0	ARB	0	Photophobia	1	Support group	1	Abortive	0	Sister	0
Education	0	Parent	0	Depressed	0	Visual disturbance	0	ARB	0	Photophobia	0	Support group	0	Abortive	0	Sister	0
Education	0	Parent	0	Depressed	0	Visual disturbance	0	ARB	0	Photophobia	0	Support group	5	Abortive	0	Sister	0
Education	0	Parent	1	Depressed	0	Visual disturbance	0	ARB	0	Photophobia	0	Support group	0	Abortive	0	Sister	0
Education	0	Parent	0	Depressed	0	Visual disturbance	0	ARB	0	Photophobia	0	Support group	0	Abortive	0	Sister	0
Education	0	Parent	0	Depressed	0	Visual disturbance	0	ARB	0	Photophobia	0	Support group	0	Abortive	0	Sister	0
Education	0	Parent	0	Depressed	0	Visual disturbance	0	ARB	1	Photophobia	0	Support group	0	Abortive	0	Sister	0
Education	0	Parent	0	Depressed	0	Visual disturbance	0	ARB	0	Photophobia	0	Support group	0	Abortive	0	Sister	0
Education	0	Parent	1	Depressed	0	Visual disturbance	0	ARB	0	Photophobia	0	Support group	0	Abortive	0	Sister	1
Education	0	Parent	0	Depressed	0	Visual disturbance	0	ARB	0	Photophobia	0	Support group	0	Abortive	0	Sister	0
Education	1	Parent	2	Depressed	0	Visual disturbance	0	ARB	0	Photophobia	0	Support group	0	Abortive	0	Sister	0
Education	0	Parent	0	Depressed	0	Visual disturbance	0	ARB	0	Photophobia	0	Support group	0	Abortive	0	Sister	0
Education	0	Parent	0	Depressed	0	Visual disturbance	0	ARB	0	Photophobia	0	Support group	0	Abortive	0	Sister	5
Education	0	Parent	0	Depressed	0	Visual disturbance	0	ARB	0	Photophobia	0	Support group	0	Abortive	0	Sister	0
Education	0	Parent	0	Depressed	0	Visual disturbance	0	ARB	0	Photophobia	0	Support group	0	Abortive	2	Sister	0
Education	0	Parent	3	Depressed	0	Visual disturbance	4	ARB	1	Photophobia	0	Support group	0	Abortive	0	Sister	1
Education	0	Parent	0	Depressed	0	Visual disturbance	0	ARB	0	Photophobia	0	Support group	0	Abortive	0	Sister	0
Education	0	Parent	0	Depressed	0	Visual disturbance	0	ARB	0	Photophobia	0	Support group	0	Abortive	0	Sister	0
Education	0	Parent	0	Depressed	0	Visual disturbance	0	ARB	0	Photophobia	0	Support group	0	Abortive	1	Sister	0
Education	0	Parent	0	Depressed	0	Visual disturbance	0	ARB	0	Photophobia	0	Support group	0	Abortive	0	Sister	0
Education	0	Parent	0	Depressed	0	Visual disturbance	0	ARB	0	Photophobia	0	Support group	0	Abortive	0	Sister	0
Education	0	Parent	0	Depressed	0	Visual disturbance	0	ARB	0	Photophobia	0	Support group	1	Abortive	0	Sister	0
Education	0	Parent	0	Depressed	0	Visual disturbance	0	ARB	0	Photophobia	0	Support group	0	Abortive	0	Sister	0
Education	0	Parent	0	Depressed	0	Visual disturbance	0	ARB	0	Photophobia	0	Support group	0	Abortive	0	Sister	0
Education	0	Parent	0	Depressed	0	Visual disturbance	0	ARB	0	Photophobia	0	Support group	1	Abortive	0	Sister	0
Education	1	Parent	0	Depressed	0	Visual disturbance	0	ARB	0	Photophobia	0	Support group	0	Abortive	1	Sister	0
Education	0	Parent	0	Depressed	0	Visual disturbance	0	ARB	0	Photophobia	0	Support group	0	Abortive	0	Sister	0
Education	0	Parent	0	Depressed	0	Visual disturbance	0	ARB	0	Photophobia	0	Support group	0	Abortive	0	Sister	0
Education	0	Parent	0	Depressed	0	Visual disturbance	0	ARB	0	Photophobia	0	Support group	0	Abortive	0	Sister	0
Education	0	Parent	0	Depressed	0	Visual disturbance	0	ARB	0	Photophobia	0	Support group	0	Abortive	0	Sister	0
Education	0	Parent	0	Depressed	0	Visual disturbance	0	ARB	0	Photophobia	0	Support group	0	Abortive	1	Sister	0
Education	0	Parent	0	Depressed	0	Visual disturbance	1	ARB	0	Photophobia	0	Support group	0	Abortive	0	Sister	0
Education	0	Parent	2	Depressed	8	Visual disturbance	2	ARB	8	Photophobia	8	Support group	0	Abortive	6	Sister	4
Education	1	Parent	0	Depressed	0	Visual disturbance	0	ARB	0	Photophobia	0	Support group	3	Abortive	0	Sister	0
Education	0	Parent	0	Depressed	1	Visual disturbance	0	ARB	0	Photophobia	0	Support group	2	Abortive	0	Sister	0
Education	0	Parent	0	Depressed	0	Visual disturbance	0	ARB	0	Photophobia	0	Support group	0	Abortive	0	Sister	0
Education	1	Parent	0	Depressed	0	Visual disturbance	0	ARB	3	Photophobia	0	Support group	1	Abortive	2	Sister	0
Education	0	Parent	0	Depressed	0	Visual disturbance	0	ARB	0	Photophobia	0	Support group	0	Abortive	0	Sister	0
Education	0	Parent	0	Depressed	0	Visual disturbance	0	ARB	0	Photophobia	0	Support group	0	Abortive	0	Sister	0
Education	0	Parent	0	Depressed	0	Visual disturbance	2	ARB	0	Photophobia	0	Support group	0	Abortive	0	Sister	0
Education	0	Parent	0	Depressed	0	Visual disturbance	0	ARB	0	Photophobia	0	Support group	0	Abortive	0	Sister	0
Education	0	Parent	3	Depressed	0	Visual disturbance	0	ARB	0	Photophobia	0	Support group	0	Abortive	0	Sister	0
Education	0	Parent	0	Depressed	0	Visual disturbance	0	ARB	0	Photophobia	0	Support group	0	Abortive	0	Sister	0
Education	0	Parent	0	Depressed	0	Visual disturbance	0	ARB	0	Photophobia	0	Support group	0	Abortive	0	Sister	0
Education	0	Parent	0	Depressed	0	Visual disturbance	0	ARB	0	Photophobia	0	Support group	0	Abortive	0	Sister	0
Education	0	Parent	0	Depressed	0	Visual disturbance	0	ARB	0	Photophobia	0	Support group	0	Abortive	0	Sister	0
Education	1	Parent	0	Depressed	0	Visual disturbance	0	ARB	0	Photophobia	0	Support group	0	Abortive	0	Sister	0
Education	1	Parent	0	Depressed	0	Visual disturbance	0	ARB	0	Photophobia	0	Support group	0	Abortive	0	Sister	0
Education	0	Parent	0	Depressed	0	Visual disturbance	0	ARB	0	Photophobia	0	Support group	0	Abortive	0	Sister	0
Education	1	Parent	0	Depressed	0	Visual disturbance	1	ARB	0	Photophobia	0	Support group	0	Abortive	0	Sister	0
Education	0	Parent	0	Depressed	0	Visual disturbance	0	ARB	0	Photophobia	0	Support group	0	Abortive	0	Sister	0
Education	0	Parent	0	Depressed	0	Visual disturbance	1	ARB	0	Photophobia	0	Support group	0	Abortive	0	Sister	0
Education	0	Parent	1	Depressed	0	Visual disturbance	2	ARB	0	Photophobia	0	Support group	0	Abortive	0	Sister	2
Education	1	Parent	0	Depressed	0	Visual disturbance	0	ARB	0	Photophobia	0	Support group	0	Abortive	0	Sister	0
Education	0	Parent	0	Depressed	0	Visual disturbance	0	ARB	0	Photophobia	0	Support group	0	Abortive	0	Sister	0
Education	0	Parent	1	Depressed	0	Visual disturbance	0	ARB	0	Photophobia	0	Support group	0	Abortive	0	Sister	0
Education	0	Parent	0	Depressed	0	Visual disturbance	0	ARB	0	Photophobia	0	Support group	0	Abortive	0	Sister	0
Education	1	Parent	0	Depressed	0	Visual disturbance	1	ARB	0	Photophobia	0	Support group	0	Abortive	0	Sister	0
Education	0	Parent	0	Depressed	5	Visual disturbance	0	ARB	1	Photophobia	5	Support group	0	Abortive	0	Sister	0
Education	1	Parent	0	Depressed	0	Visual disturbance	0	ARB	0	Photophobia	0	Support group	0	Abortive	0	Sister	0
Education	0	Parent	0	Depressed	0	Visual disturbance	0	ARB	0	Photophobia	0	Support group	0	Abortive	0	Sister	0
Education	0	Parent	0	Depressed	0	Visual disturbance	0	ARB	0	Photophobia	0	Support group	0	Abortive	0	Sister	0
	15		15		14		14		14		14		14		13		13

P102a	P102b	P103a	P103b	P104a	P104b	P105a	P105b	P106a	P106b	P107a	P107b	P108a	P108b	P109a	P109b	P110a	P110b	P111a	P111b
Sensitivity to light	0	Wife	1	Emergency	0	Stages	0	Surgery	0	Lifestyle	0	Acute	0	Sore	0	Hemiplegic migraine	0	Allodynia	0
Sensitivity to light	0	Wife	0	Emergency	0	Stages	0	Surgery	0	Lifestyle	0	Acute	0	Sore	0	Hemiplegic migraine	0	Allodynia	0
Sensitivity to light	1	Wife	1	Emergency	0	Stages	0	Surgery	1	Lifestyle	0	Acute	0	Sore	0	Hemiplegic migraine	0	Allodynia	0
Sensitivity to light	0	Wife	0	Emergency	0	Stages	0	Surgery	0	Lifestyle	0	Acute	0	Sore	0	Hemiplegic migraine	0	Allodynia	0
Sensitivity to light	0	Wife	0	Emergency	0	Stages	0	Surgery	0	Lifestyle	0	Acute	0	Sore	0	Hemiplegic migraine	3	Allodynia	0
Sensitivity to light	0	Wife	0	Emergency	0	Stages	0	Surgery	0	Lifestyle	0	Acute	0	Sore	0	Hemiplegic migraine	0	Allodynia	0
Sensitivity to light	0	Wife	0	Emergency	0	Stages	1	Surgery	0	Lifestyle	0	Acute	0	Sore	0	Hemiplegic migraine	0	Allodynia	0
Sensitivity to light	0	Wife	0	Emergency	0	Stages	0	Surgery	0	Lifestyle	0	Acute	0	Sore	0	Hemiplegic migraine	0	Allodynia	0
Sensitivity to light	0	Wife	0	Emergency	0	Stages	0	Surgery	0	Lifestyle	0	Acute	0	Sore	0	Hemiplegic migraine	0	Allodynia	0
Sensitivity to light	0	Wife	0	Emergency	0	Stages	0	Surgery	0	Lifestyle	0	Acute	0	Sore	0	Hemiplegic migraine	0	Allodynia	0
Sensitivity to light	2	Wife	0	Emergency	0	Stages	0	Surgery	0	Lifestyle	0	Acute	0	Sore	0	Hemiplegic migraine	0	Allodynia	0
Sensitivity to light	0	Wife	0	Emergency	0	Stages	0	Surgery	0	Lifestyle	0	Acute	0	Sore	0	Hemiplegic migraine	1	Allodynia	0
Sensitivity to light	0	Wife	0	Emergency	0	Stages	1	Surgery	0	Lifestyle	0	Acute	0	Sore	0	Hemiplegic migraine	0	Allodynia	0
Sensitivity to light	0	Wife	1	Emergency	1	Stages	0	Surgery	0	Lifestyle	0	Acute	0	Sore	1	Hemiplegic migraine	0	Allodynia	0
Sensitivity to light	0	Wife	0	Emergency	1	Stages	0	Surgery	0	Lifestyle	0	Acute	0	Sore	0	Hemiplegic migraine	0	Allodynia	0
Sensitivity to light	0	Wife	0	Emergency	0	Stages	0	Surgery	0	Lifestyle	0	Acute	0	Sore	0	Hemiplegic migraine	0	Allodynia	0
Sensitivity to light	0	Wife	0	Emergency	0	Stages	0	Surgery	0	Lifestyle	0	Acute	7	Sore	0	Hemiplegic migraine	0	Allodynia	0
Sensitivity to light	0	Wife	0	Emergency	2	Stages	0	Surgery	0	Lifestyle	0	Acute	0	Sore	0	Hemiplegic migraine	0	Allodynia	0
Sensitivity to light	0	Wife	0	Emergency	0	Stages	0	Surgery	0	Lifestyle	0	Acute	0	Sore	0	Hemiplegic migraine	0	Allodynia	0
Sensitivity to light	0	Wife	0	Emergency	1	Stages	0	Surgery	0	Lifestyle	0	Acute	0	Sore	0	Hemiplegic migraine	0	Allodynia	0
Sensitivity to light	0	Wife	0	Emergency	0	Stages	0	Surgery	0	Lifestyle	0	Acute	0	Sore	0	Hemiplegic migraine	0	Allodynia	0
Sensitivity to light	0	Wife	0	Emergency	1	Stages	0	Surgery	0	Lifestyle	1	Acute	1	Sore	0	Hemiplegic migraine	2	Allodynia	0
Sensitivity to light	0	Wife	0	Emergency	0	Stages	0	Surgery	0	Lifestyle	0	Acute	0	Sore	0	Hemiplegic migraine	0	Allodynia	0
Sensitivity to light	1	Wife	0	Emergency	0	Stages	0	Surgery	0	Lifestyle	0	Acute	0	Sore	0	Hemiplegic migraine	0	Allodynia	0
Sensitivity to light	0	Wife	0	Emergency	0	Stages	0	Surgery	0	Lifestyle	0	Acute	0	Sore	1	Hemiplegic migraine	0	Allodynia	0
Sensitivity to light	0	Wife	0	Emergency	0	Stages	0	Surgery	0	Lifestyle	0	Acute	0	Sore	0	Hemiplegic migraine	0	Allodynia	0
Sensitivity to light	0	Wife	0	Emergency	0	Stages	0	Surgery	0	Lifestyle	0	Acute	0	Sore	0	Hemiplegic migraine	0	Allodynia	0
Sensitivity to light	0	Wife	0	Emergency	0	Stages	0	Surgery	0	Lifestyle	0	Acute	0	Sore	0	Hemiplegic migraine	0	Allodynia	0
Sensitivity to light	0	Wife	0	Emergency	0	Stages	0	Surgery	0	Lifestyle	0	Acute	0	Sore	0	Hemiplegic migraine	0	Allodynia	0
Sensitivity to light	0	Wife	1	Emergency	0	Stages	0	Surgery	1	Lifestyle	0	Acute	0	Sore	1	Hemiplegic migraine	0	Allodynia	0
Sensitivity to light	0	Wife	0	Emergency	0	Stages	0	Surgery	0	Lifestyle	0	Acute	0	Sore	0	Hemiplegic migraine	0	Allodynia	0
Sensitivity to light	0	Wife	0	Emergency	0	Stages	0	Surgery	0	Lifestyle	0	Acute	0	Sore	0	Hemiplegic migraine	0	Allodynia	0
Sensitivity to light	0	Wife	1	Emergency	0	Stages	0	Surgery	0	Lifestyle	0	Acute	0	Sore	0	Hemiplegic migraine	0	Allodynia	0
Sensitivity to light	0	Wife	0	Emergency	0	Stages	0	Surgery	0	Lifestyle	0	Acute	0	Sore	0	Hemiplegic migraine	0	Allodynia	0
Sensitivity to light	7	Wife	6	Emergency	3	Stages	0	Surgery	2	Lifestyle	4	Acute	1	Sore	6	Hemiplegic migraine	1	Allodynia	10
Sensitivity to light	0	Wife	0	Emergency	0	Stages	0	Surgery	0	Lifestyle	0	Acute	0	Sore	0	Hemiplegic migraine	0	Allodynia	0
Sensitivity to light	1	Wife	0	Emergency	0	Stages	1	Surgery	0	Lifestyle	4	Acute	0	Sore	0	Hemiplegic migraine	0	Allodynia	0
Sensitivity to light	0	Wife	0	Emergency	0	Stages	0	Surgery	0	Lifestyle	0	Acute	0	Sore	0	Hemiplegic migraine	0	Allodynia	0
Sensitivity to light	0	Wife	0	Emergency	0	Stages	0	Surgery	1	Lifestyle	0	Acute	0	Sore	0	Hemiplegic migraine	4	Allodynia	0
Sensitivity to light	0	Wife	0	Emergency	0	Stages	0	Surgery	0	Lifestyle	0	Acute	0	Sore	0	Hemiplegic migraine	0	Allodynia	0
Sensitivity to light	0	Wife	0	Emergency	0	Stages	0	Surgery	0	Lifestyle	0	Acute	0	Sore	0	Hemiplegic migraine	0	Allodynia	0
Sensitivity to light	0	Wife	0	Emergency	0	Stages	0	Surgery	0	Lifestyle	0	Acute	0	Sore	0	Hemiplegic migraine	0	Allodynia	0
Sensitivity to light	0	Wife	0	Emergency	0	Stages	0	Surgery	0	Lifestyle	0	Acute	0	Sore	0	Hemiplegic migraine	0	Allodynia	0
Sensitivity to light	0	Wife	0	Emergency	1	Stages	0	Surgery	0	Lifestyle	0	Acute	0	Sore	0	Hemiplegic migraine	0	Allodynia	0
Sensitivity to light	0	Wife	0	Emergency	0	Stages	0	Surgery	0	Lifestyle	0	Acute	0	Sore	0	Hemiplegic migraine	0	Allodynia	0
Sensitivity to light	0	Wife	0	Emergency	0	Stages	0	Surgery	0	Lifestyle	0	Acute	0	Sore	0	Hemiplegic migraine	0	Allodynia	0
Sensitivity to light	0	Wife	0	Emergency	0	Stages	0	Surgery	0	Lifestyle	0	Acute	0	Sore	0	Hemiplegic migraine	0	Allodynia	0
Sensitivity to light	0	Wife	0	Emergency	0	Stages	0	Surgery	0	Lifestyle	0	Acute	0	Sore	0	Hemiplegic migraine	0	Allodynia	0
Sensitivity to light	0	Wife	0	Emergency	0	Stages	0	Surgery	0	Lifestyle	0	Acute	0	Sore	0	Hemiplegic migraine	0	Allodynia	0
Sensitivity to light	0	Wife	0	Emergency	0	Stages	0	Surgery	0	Lifestyle	0	Acute	0	Sore	0	Hemiplegic migraine	0	Allodynia	0
Sensitivity to light	0	Wife	0	Emergency	0	Stages	0	Surgery	0	Lifestyle	2	Acute	0	Sore	0	Hemiplegic migraine	0	Allodynia	0
Sensitivity to light	0	Wife	0	Emergency	2	Stages	0	Surgery	0	Lifestyle	0	Acute	2	Sore	0	Hemiplegic migraine	0	Allodynia	0
Sensitivity to light	0	Wife	0	Emergency	0	Stages	0	Surgery	0	Lifestyle	0	Acute	0	Sore	0	Hemiplegic migraine	0	Allodynia	0
Sensitivity to light	0	Wife	0	Emergency	0	Stages	0	Surgery	0	Lifestyle	0	Acute	0	Sore	0	Hemiplegic migraine	0	Allodynia	0
Sensitivity to light	1	Wife	2	Emergency	0	Stages	0	Surgery	5	Lifestyle	0	Acute	0	Sore	2	Hemiplegic migraine	0	Allodynia	0
Sensitivity to light	0	Wife	0	Emergency	0	Stages	0	Surgery	0	Lifestyle	0	Acute	0	Sore	0	Hemiplegic migraine	0	Allodynia	0
Sensitivity to light	0	Wife	0	Emergency	0	Stages	0	Surgery	1	Lifestyle	0	Acute	0	Sore	0	Hemiplegic migraine	0	Allodynia	0
Sensitivity to light	0	Wife	0	Emergency	0	Stages	0	Surgery	0	Lifestyle	0	Acute	0	Sore	0	Hemiplegic migraine	0	Allodynia	0
Sensitivity to light	0	Wife	0	Emergency	0	Stages	0	Surgery	1	Lifestyle	0	Acute	0	Sore	0	Hemiplegic migraine	0	Allodynia	0
Sensitivity to light	0	Wife	0	Emergency	0	Stages	9	Surgery	0	Lifestyle	0	Acute	0	Sore	0	Hemiplegic migraine	0	Allodynia	0
Sensitivity to light	0	Wife	0	Emergency	0	Stages	0	Surgery	0	Lifestyle	0	Acute	0	Sore	0	Hemiplegic migraine	0	Allodynia	0
Sensitivity to light	0	Wife	0	Emergency	0	Stages	0	Surgery	0	Lifestyle	0	Acute	0	Sore	0	Hemiplegic migraine	0	Allodynia	0
Sensitivity to light	0	Wife	0	Emergency	0	Stages	0	Surgery	0	Lifestyle	0	Acute	0	Sore	0	Hemiplegic migraine	0	Allodynia	0
Sensitivity to light	0	Wife	0	Emergency	0	Stages	0	Surgery	0	Lifestyle	0	Acute	0	Sore	0	Hemiplegic migraine	0	Allodynia	0
	13		13		12		12		12		11		11		11		11		10

P112a	P112b	P113a	P113b	P114a	P114b	P115a	P115b	P116a	P116b	P117a	P117b	P118a	P118b	P119a	P119b	P120a	P120b	P121a	P121b	P122a	P122b	P123a	P123b	P124a
Antidepressants	0	Occipital	0	procedure	0	Procedure	0	Coping	0	Calcium channel	0	Riboflavin	0	Central	0	Future	0	Nurse	0	nose	0	Intractable	0	History
Antidepressants	0	Occipital	0	procedure	0	Procedure	0	Coping	0	Calcium channel	0	Riboflavin	0	Central	0	Future	0	Nurse	0	nose	0	Intractable	0	History
Antidepressants	0	Occipital	0	procedure	0	Procedure	0	Coping	0	Calcium channel	0	Riboflavin	0	Central	0	Future	0	Nurse	0	nose	0	Intractable	0	History
Antidepressants	0	Occipital	0	procedure	0	Procedure	0	Coping	0	Calcium channel	0	Riboflavin	0	Central	0	Future	0	Nurse	0	nose	0	Intractable	0	History
Antidepressants	0	Occipital	0	procedure	0	Procedure	0	Coping	0	Calcium channel	0	Riboflavin	0	Central	0	Future	0	Nurse	0	nose	0	Intractable	1	History
Antidepressants	0	Occipital	0	procedure	0	Procedure	0	Coping	0	Calcium channel	0	Riboflavin	0	Central	0	Future	0	Nurse	0	nose	0	Intractable	0	History
Antidepressants	0	Occipital	0	procedure	0	Procedure	0	Coping	0	Calcium channel	0	Riboflavin	0	Central	0	Future	0	Nurse	0	nose	0	Intractable	0	History
Antidepressants	0	Occipital	0	procedure	0	Procedure	0	Coping	1	Calcium channel	0	Riboflavin	0	Central	0	Future	0	Nurse	1	nose	1	Intractable	0	History
Antidepressants	0	Occipital	0	procedure	0	Procedure	0	Coping	2	Calcium channel	0	Riboflavin	0	Central	0	Future	0	Nurse	1	nose	0	Intractable	0	History
Antidepressants	0	Occipital	0	procedure	0	Procedure	0	Coping	1	Calcium channel	0	Riboflavin	0	Central	0	Future	0	Nurse	0	nose	0	Intractable	0	History
Antidepressants	0	Occipital	0	procedure	0	Procedure	0	Coping	0	Calcium channel	0	Riboflavin	0	Central	0	Future	0	Nurse	2	nose	0	Intractable	0	History
Antidepressants	0	Occipital	0	procedure	0	Procedure	0	Coping	1	Calcium channel	0	Riboflavin	0	Central	0	Future	0	Nurse	0	nose	0	Intractable	0	History
Antidepressants	0	Occipital	0	procedure	0	Procedure	0	Coping	0	Calcium channel	0	Riboflavin	0	Central	0	Future	0	Nurse	0	nose	0	Intractable	0	History
Antidepressants	0	Occipital	0	procedure	0	Procedure	0	Coping	0	Calcium channel	0	Riboflavin	0	Central	0	Future	1	Nurse	0	nose	1	Intractable	0	History
Antidepressants	0	Occipital	0	procedure	0	Procedure	0	Coping	0	Calcium channel	0	Riboflavin	0	Central	0	Future	0	Nurse	0	nose	0	Intractable	0	History
Antidepressants	0	Occipital	0	procedure	0	Procedure	0	Coping	0	Calcium channel	0	Riboflavin	0	Central	0	Future	0	Nurse	0	nose	0	Intractable	0	History
Antidepressants	0	Occipital	0	procedure	0	Procedure	0	Coping	0	Calcium channel	0	Riboflavin	0	Central	2	Future	0	Nurse	0	nose	0	Intractable	0	History
Antidepressants	0	Occipital	0	procedure	0	Procedure	0	Coping	0	Calcium channel	0	Riboflavin	1	Central	0	Future	0	Nurse	0	nose	0	Intractable	0	History
Antidepressants	0	Occipital	0	procedure	0	Procedure	0	Coping	0	Calcium channel	0	Riboflavin	0	Central	0	Future	0	Nurse	0	nose	0	Intractable	0	History
Antidepressants	0	Occipital	0	procedure	0	Procedure	0	Coping	0	Calcium channel	0	Riboflavin	0	Central	0	Future	0	Nurse	0	nose	0	Intractable	0	History
Antidepressants	0	Occipital	0	procedure	0	Procedure	0	Coping	0	Calcium channel	2	Riboflavin	0	Central	0	Future	0	Nurse	0	nose	1	Intractable	6	History
Antidepressants	0	Occipital	0	procedure	0	Procedure	0	Coping	0	Calcium channel	0	Riboflavin	0	Central	1	Future	0	Nurse	0	nose	0	Intractable	0	History
Antidepressants	0	Occipital	0	procedure	0	Procedure	0	Coping	0	Calcium channel	0	Riboflavin	0	Central	0	Future	0	Nurse	0	nose	0	Intractable	0	History
Antidepressants	0	Occipital	0	procedure	0	Procedure	0	Coping	0	Calcium channel	0	Riboflavin	0	Central	0	Future	0	Nurse	0	nose	0	Intractable	0	History
Antidepressants	0	Occipital	0	procedure	0	Procedure	0	Coping	0	Calcium channel	0	Riboflavin	0	Central	0	Future	0	Nurse	0	nose	0	Intractable	0	History
Antidepressants	0	Occipital	0	procedure	0	Procedure	0	Coping	0	Calcium channel	0	Riboflavin	0	Central	0	Future	0	Nurse	0	nose	0	Intractable	0	History
Antidepressants	0	Occipital	0	procedure	1	Procedure	1	Coping	0	Calcium channel	0	Riboflavin	0	Central	0	Future	0	Nurse	1	nose	2	Intractable	0	History
Antidepressants	0	Occipital	0	procedure	0	Procedure	0	Coping	0	Calcium channel	0	Riboflavin	0	Central	0	Future	0	Nurse	0	nose	0	Intractable	0	History
Antidepressants	0	Occipital	0	procedure	1	Procedure	1	Coping	0	Calcium channel	0	Riboflavin	0	Central	0	Future	0	Nurse	1	nose	0	Intractable	0	History
Antidepressants	0	Occipital	0	procedure	0	Procedure	0	Coping	0	Calcium channel	0	Riboflavin	0	Central	0	Future	0	Nurse	0	nose	0	Intractable	0	History
Antidepressants	0	Occipital	0	procedure	0	Procedure	0	Coping	0	Calcium channel	0	Riboflavin	0	Central	0	Future	0	Nurse	0	nose	0	Intractable	0	History
Antidepressants	0	Occipital	0	procedure	0	Procedure	0	Coping	0	Calcium channel	0	Riboflavin	0	Central	0	Future	0	Nurse	0	nose	0	Intractable	0	History
Antidepressants	0	Occipital	0	procedure	0	Procedure	0	Coping	0	Calcium channel	0	Riboflavin	0	Central	0	Future	0	Nurse	0	nose	0	Intractable	0	History
Antidepressants	0	Occipital	0	procedure	0	Procedure	0	Coping	0	Calcium channel	0	Riboflavin	0	Central	0	Future	0	Nurse	0	nose	0	Intractable	0	History
Antidepressants	2	Occipital	9	procedure	6	Procedure	6	Coping	1	Calcium channel	1	Riboflavin	2	Central	0	Future	0	Nurse	1	nose	3	Intractable	2	History
Antidepressants	0	Occipital	0	procedure	0	Procedure	0	Coping	1	Calcium channel	0	Riboflavin	0	Central	0	Future	0	Nurse	0	nose	0	Intractable	0	History
Antidepressants	0	Occipital	0	procedure	0	Procedure	0	Coping	1	Calcium channel	0	Riboflavin	0	Central	0	Future	0	Nurse	0	nose	0	Intractable	0	History
Antidepressants	0	Occipital	0	procedure	0	Procedure	0	Coping	0	Calcium channel	0	Riboflavin	0	Central	0	Future	0	Nurse	0	nose	0	Intractable	0	History
Antidepressants	5	Occipital	0	procedure	0	Procedure	0	Coping	0	Calcium channel	4	Riboflavin	4	Central	1	Future	0	Nurse	0	nose	0	Intractable	0	History
Antidepressants	0	Occipital	0	procedure	0	Procedure	0	Coping	0	Calcium channel	0	Riboflavin	0	Central	0	Future	0	Nurse	0	nose	0	Intractable	0	History
Antidepressants	0	Occipital	0	procedure	0	Procedure	0	Coping	0	Calcium channel	0	Riboflavin	0	Central	0	Future	0	Nurse	0	nose	0	Intractable	0	History
Antidepressants	0	Occipital	0	procedure	0	Procedure	0	Coping	0	Calcium channel	1	Riboflavin	1	Central	0	Future	0	Nurse	0	nose	0	Intractable	0	History
Antidepressants	0	Occipital	0	procedure	0	Procedure	0	Coping	0	Calcium channel	0	Riboflavin	1	Central	0	Future	0	Nurse	0	nose	0	Intractable	0	History
Antidepressants	0	Occipital	0	procedure	0	Procedure	0	Coping	0	Calcium channel	0	Riboflavin	0	Central	0	Future	1	Nurse	0	nose	1	Intractable	0	History
Antidepressants	0	Occipital	0	procedure	0	Procedure	0	Coping	0	Calcium channel	1	Riboflavin	0	Central	0	Future	0	Nurse	0	nose	0	Intractable	0	History
Antidepressants	0	Occipital	0	procedure	0	Procedure	0	Coping	0	Calcium channel	0	Riboflavin	0	Central	0	Future	0	Nurse	0	nose	0	Intractable	0	History
Antidepressants	0	Occipital	0	procedure	0	Procedure	0	Coping	0	Calcium channel	0	Riboflavin	0	Central	0	Future	0	Nurse	2	nose	0	Intractable	0	History
Antidepressants	0	Occipital	0	procedure	0	Procedure	0	Coping	0	Calcium channel	0	Riboflavin	0	Central	0	Future	0	Nurse	0	nose	0	Intractable	0	History
Antidepressants	0	Occipital	0	procedure	0	Procedure	0	Coping	0	Calcium channel	0	Riboflavin	0	Central	1	Future	0	Nurse	0	nose	0	Intractable	0	History
Antidepressants	0	Occipital	0	procedure	0	Procedure	0	Coping	0	Calcium channel	0	Riboflavin	0	Central	1	Future	0	Nurse	0	nose	0	Intractable	0	History
Antidepressants	0	Occipital	0	procedure	0	Procedure	0	Coping	0	Calcium channel	0	Riboflavin	0	Central	0	Future	2	Nurse	0	nose	0	Intractable	0	History
Antidepressants	0	Occipital	0	procedure	1	Procedure	1	Coping	0	Calcium channel	0	Riboflavin	0	Central	0	Future	1	Nurse	0	nose	0	Intractable	0	History
Antidepressants	0	Occipital	0	procedure	0	Procedure	0	Coping	0	Calcium channel	0	Riboflavin	0	Central	0	Future	0	Nurse	0	nose	0	Intractable	0	History
Antidepressants	0	Occipital	0	procedure	0	Procedure	0	Coping	0	Calcium channel	0	Riboflavin	0	Central	0	Future	0	Nurse	0	nose	0	Intractable	0	History
Antidepressants	3	Occipital	0	procedure	0	Procedure	0	Coping	0	Calcium channel	0	Riboflavin	0	Central	1	Future	0	Nurse	0	nose	0	Intractable	0	History
Antidepressants	0	Occipital	0	procedure	0	Procedure	0	Coping	0	Calcium channel	0	Riboflavin	0	Central	1	Future	0	Nurse	0	nose	0	Intractable	0	History
Antidepressants	0	Occipital	0	procedure	0	Procedure	0	Coping	0	Calcium channel	0	Riboflavin	0	Central	0	Future	1	Nurse	0	nose	0	Intractable	0	History
Antidepressants	0	Occipital	0	procedure	0	Procedure	0	Coping	0	Calcium channel	0	Riboflavin	0	Central	0	Future	0	Nurse	0	nose	0	Intractable	0	History
Antidepressants	0	Occipital	0	procedure	1	Procedure	1	Coping	0	Calcium channel	0	Riboflavin	0	Central	0	Future	0	Nurse	0	nose	0	Intractable	0	History
Antidepressants	0	Occipital	0	procedure	0	Procedure	0	Coping	0	Calcium channel	0	Riboflavin	0	Central	0	Future	0	Nurse	0	nose	0	Intractable	0	History
Antidepressants	0	Occipital	0	procedure	0	Procedure	0	Coping	0	Calcium channel	0	Riboflavin	0	Central	0	Future	0	Nurse	0	nose	0	Intractable	0	History
Antidepressants	0	Occipital	1	procedure	0	Procedure	0	Coping	2	Calcium channel	0	Riboflavin	0	Central	1	Future	3	Nurse	0	nose	0	Intractable	0	History
Antidepressants	0	Occipital	0	procedure	0	Procedure	0	Coping	0	Calcium channel	0	Riboflavin	0	Central	0	Future	0	Nurse	0	nose	0	Intractable	0	History
Antidepressants	0	Occipital	0	procedure	0	Procedure	0	Coping	0	Calcium channel	0	Riboflavin	0	Central	0	Future	0	Nurse	0	nose	0	Intractable	0	History
	10		10		10		10		10		9		9		9		9		9		9		9	

P124b	P125a	P125b	P126a	P126b	P127a	P127b	P128a	P128b	P129a	P129b	P130a	P130b	P131a	P131b	P132a	P132b	P133a	P133b	P134a	P134b	P135a	P135b	P136a	P136b	P137a	P137b	P138a	P138b	P139a	P139b	P140a	P140b	P141a	P141b	P142a	P142b	P143a	P143b	P144a	P144b	P145a	P145b	P146a	P146b	P147a	P147b	P148a	P148b	P149a	P149b	P150a	P150b	P151a	P151b	P152a	P152b	P153a	P153b	P154a	P154b	P155a	P155b	P156a	P156b	P157a	P157b	P158a	P158b	P159a	P159b	P160a	P160b	P161a	P161b	P162a	P162b	P163a	P163b	P164a	P164b	P165a	P165b	P166a	P166b	P167a	P167b	P168a	P168b	P169a	P169b	P170a	P170b	P171a	P171b	P172a	P172b	P173a	P173b	P174a	P174b	P175a	P175b	P176a	P176b	P177a	P177b	P178a	P178b	P179a	P179b	P180a	P180b
1	Biofeedback	0	Preventive	0	Joy	0	Vascular	0	Butalbital	0	Impact	0	Drive	0	Peer	1	Prophylaxis	0	Angiotensin	0	Toxin	0	Duration	0	Sad	0	minister	0	Hurts	0	Incidence	0	Testing	0	stigma	0	Aggravate	0	Calcium channel blocker	0	faith	0	Adverse effect	0	Confuse	0	Anger	0	Patterns	0	Quality of life	0	Suicide	2	Trigger point	0	Agonist	0	Motion sickness	0	Acute migraine	0	Steroid	0	Anti-inflammatory	0	CAT scan	0	NSAID	0	Shock	0	Epilepsy	0	Headache diary	0	Sinus headache	0	Weight loss	0	Cervical spine	0	boss	0	Characteristic	0	Peer support	0	Facial pain	0	Grandmother	0	Glutamate	0	Vestibular migraine	0	Upset	0	regime	0	vacation	0	Accident	0	Trauma	0	Neck muscle	0	Population	0	Sensitivity to sound	0
0	Biofeedback	0	Preventive	0	Joy	0	Vascular	0	Butalbital	0	Impact	0	Drive	0	Peer	0	Prophylaxis	0	Angiotensin	0	Toxin	0	Duration	0	Sad	0	minister	0	Hurts	0	Incidence	0	Testing	0	stigma	0	Aggravate	0	Calcium channel blocker	0	faith	0	Adverse effect	0	Confuse	0	Anger	0	Patterns	0	Quality of life	0	Suicide	0	Trigger point	0	Agonist	0	Motion sickness	0	Acute migraine	0	Steroid	0	Anti-inflammatory	0	CAT scan	0	NSAID	0	Shock	0	Epilepsy	0	Headache diary	0	Sinus headache	0	Weight loss	0	Cervical spine	0	boss	0	Characteristic	0	Peer support	0	Facial pain	0	Grandmother	0	Glutamate	0	Vestibular migraine	0	Upset	0	regime	0	vacation	0	Accident	0	Trauma	0	Neck muscle	0	Population	0	Sensitivity to sound	0
0	Biofeedback	0	Preventive	0	Joy	0	Vascular	0	Butalbital	0	Impact	0	Drive	0	Peer	0	Prophylaxis	0	Angiotensin	0	Toxin	0	Duration	0	Sad	0	minister	0	Hurts	0	Incidence	0	Testing	1	stigma	0	Aggravate	0	Calcium channel blocker	0	faith	0	Adverse effect	0	Confuse	0	Anger	0	Patterns	0	Quality of life	1	Suicide	0	Trigger point	0	Agonist	0	Motion sickness	0	Acute migraine	0	Steroid	0	Anti-inflammatory	0	CAT scan	0	NSAID	0	Shock	0	Epilepsy	0	Headache diary	0	Sinus headache	0	Weight loss	0	Cervical spine	0	boss	0	Characteristic	0	Peer support	0	Facial pain	0	Grandmother	0	Glutamate	0	Vestibular migraine	0	Upset	0	regime	0	vacation	0	Accident	0	Trauma	0	Neck muscle	0	Population	0	Sensitivity to sound	1
0	Biofeedback	0	Preventive	0	Joy	0	Vascular	0	Butalbital	0	Impact	0	Drive	0	Peer	0	Prophylaxis	0	Angiotensin	0	Toxin	0	Duration	0	Sad	0	minister	0	Hurts	0	Incidence	0	Testing	0	stigma	0	Aggravate	0	Calcium channel blocker	0	faith	0	Adverse effect	0	Confuse	0	Anger	0	Patterns	0	Quality of life	0	Suicide	0	Trigger point	0	Agonist	0	Motion sickness	0	Acute migraine	0	Steroid	0	Anti-inflammatory	0	CAT scan	0	NSAID	0	Shock	1	Epilepsy	0	Headache diary	0	Sinus headache	0	Weight loss	0	Cervical spine	0	boss	0	Characteristic	0	Peer support	0	Facial pain	0	Grandmother	0	Glutamate	0	Vestibular migraine	0	Upset	0	regime	0	vacation	0	Accident	0	Trauma	0	Neck muscle	0	Population	0	Sensitivity to sound	0
0	Biofeedback	0	Preventive	0	Joy	0	Vascular	0	Butalbital	0	Impact	0	Drive	0	Peer	0	Prophylaxis	0	Angiotensin	0	Toxin	0	Duration	0	Sad	0	minister	0	Hurts	0	Incidence	0	Testing	0	stigma	0	Aggravate	0	Calcium channel blocker	0	faith	0	Adverse effect	0	Confuse	0	Anger	0	Patterns	0	Quality of life	0	Suicide	0	Trigger point	0	Agonist	0	Motion sickness	0	Acute migraine	0	Steroid	0	Anti-inflammatory	0	CAT scan	0	NSAID	0	Shock	0	Epilepsy	0	Headache diary	0	Sinus headache	0	Weight loss	0	Cervical spine	0	boss	0	Characteristic	0	Peer support	0	Facial pain	0	Grandmother	0	Glutamate	0	Vestibular migraine	0	Upset	0	regime	0	vacation	0	Accident	0	Trauma	0	Neck muscle	0	Population	0	Sensitivity to sound	0
0	Biofeedback	0	Preventive	0	Joy	0	Vascular	0	Butalbital	0	Impact	0	Drive	0	Peer	0	Prophylaxis	0	Angiotensin	0	Toxin	0	Duration	0	Sad	0	minister	0	Hurts	0	Incidence	0	Testing	0	stigma	0	Aggravate	0	Calcium channel blocker	0	faith	0	Adverse effect	0	Confuse	0	Anger	0	Patterns	1	Quality of life	0	Suicide	0	Trigger point	0	Agonist	0	Motion sickness	0	Acute migraine	0	Steroid	0	Anti-inflammatory	0	CAT scan	0	NSAID	0	Shock	0	Epilepsy	0	Headache diary	0	Sinus headache	0	Weight loss	0	Cervical spine	0	boss	0	Characteristic	0	Peer support	0	Facial pain	0	Grandmother	0	Glutamate	0	Vestibular migraine	0	Upset	0	regime	0	vacation	0	Accident	0	Trauma	0	Neck muscle	0	Population	1	Sensitivity to sound	0
0	Biofeedback	0	Preventive	0	Joy	0	Vascular	0	Butalbital	0	Impact	0	Drive	0	Peer	0	Prophylaxis	0	Angiotensin	0	Toxin	0	Duration	0	Sad	0	minister	0	Hurts	0	Incidence	0	Testing	0	stigma	0	Aggravate	0	Calcium channel blocker	0	faith	0	Adverse effect	0	Confuse	0	Anger	0	Patterns	0	Quality of life	0	Suicide	0	Trigger point	0	Agonist	0	Motion sickness	0	Acute migraine	0	Steroid	0	Anti-inflammatory	0	CAT scan	0	NSAID	0	Shock	0	Epilepsy	0	Headache diary	0	Sinus headache	0	Weight loss	0	Cervical spine	0	boss	0	Characteristic	0	Peer support	0	Facial pain	0	Grandmother	0	Glutamate	0	Vestibular migraine	0	Upset	0	regime	0	vacation	0	Accident	0	Trauma	0	Neck muscle	0	Population	0	Sensitivity to sound	0
0	Biofeedback	0	Preventive	0	Joy	0	Vascular	0	Butalbital	0	Impact	0	Drive	0	Peer	0	Prophylaxis	0	Angiotensin	0	Toxin	0	Duration	0	Sad	0	minister	0	Hurts	0	Incidence	0	Testing	0	stigma	0	Aggravate	0	Calcium channel blocker	0	faith	0	Adverse effect	0	Confuse	0	Anger	0	Patterns	0	Quality of life	0	Suicide	0	Trigger point	0	Agonist	0	Motion sickness	0	Acute migraine	0	Steroid	0	Anti-inflammatory	0	CAT scan	0	NSAID	1	Shock	0	Epilepsy	0	Headache diary	0	Sinus headache	0	Weight loss	0	Cervical spine	0	boss	0	Characteristic	0	Peer support	0	Facial pain	0	Grandmother	0	Glutamate	0	Vestibular migraine	0	Upset	0	regime	0	vacation	0	Accident	0	Trauma	1	Neck muscle	1	Population	0	Sensitivity to sound	0
0	Biofeedback	0	Preventive	0	Joy	0	Vascular	0	Butalbital	0	Impact	1	Drive	1	Peer	0	Prophylaxis	0	Angiotensin	0	Toxin	0	Duration	0	Sad	0	minister	0	Hurts	0	Incidence	0	Testing	0	stigma	0	Aggravate	0	Calcium channel blocker	0	faith	0	Adverse effect	0	Confuse	0	Anger	1	Patterns	0	Quality of life	0	Suicide	3	Trigger point	0	Agonist	0	Motion sickness	0	Acute migraine	0	Steroid	0	Anti-inflammatory	0	CAT scan	0	NSAID	0	Shock	0	Epilepsy	0	Headache diary	0	Sinus headache	0	Weight loss	0	Cervical spine	0	boss	0	Characteristic	0	Peer support	0	Facial pain	0	Grandmother	0	Glutamate	0	Vestibular migraine	0	Upset	0	regime	0	vacation	2	Accident	1	Trauma	0	Neck muscle	0	Population	0	Sensitivity to sound	0
0	Biofeedback	0	Preventive	0	Joy	0	Vascular	0	Butalbital	0	Impact	0	Drive	0	Peer	0	Prophylaxis	0	Angiotensin	0	Toxin	0	Duration	0	Sad	0	minister	0	Hurts	0	Incidence	0	Testing	0	stigma	0	Aggravate	0	Calcium channel blocker	0	faith	0	Adverse effect	0	Confuse	0	Anger	0	Patterns	0	Quality of life	0	Suicide	0	Trigger point	0	Agonist	0	Motion sickness	0	Acute migraine	0	Steroid	0	Anti-inflammatory	0	CAT scan	0	NSAID	0	Shock	0	Epilepsy	0	Headache diary	0	Sinus headache	0	Weight loss	0	Cervical spine	0	boss	0	Characteristic	0	Peer support	0	Facial pain	0	Grandmother	0	Glutamate	0	Vestibular migraine	0	Upset	0	regime	1	vacation	0	Accident	0	Trauma	0	Neck muscle	0	Population	0	Sensitivity to sound	0
0	Biofeedback	0	Preventive	0	Joy	0	Vascular	0	Butalbital	0	Impact	0	Drive	1	Peer	0	Prophylaxis	0	Angiotensin	0	Toxin	0	Duration	0	Sad	0	minister	0	Hurts	0	Incidence	2	Testing	0	stigma	0	Aggravate	0	Calcium channel blocker	0	faith	0	Adverse effect	0	Confuse	1	Anger	0	Patterns	1	Quality of life	0	Suicide	0	Trigger point	0	Agonist	0	Motion sickness	0	Acute migraine	0	Steroid	0	Anti-inflammatory	0	CAT scan	1	NSAID	0	Shock	0	Epilepsy	0	Headache diary	0	Sinus headache	0	Weight loss	0	Cervical spine	0	boss	0	Characteristic	0	Peer support	0	Facial pain	0	Grandmother	0	Glutamate	0	Vestibular migraine	0	Upset	1	regime	0	vacation	0	Accident	0	Trauma	0	Neck muscle	0	Population	0	Sensitivity to sound	0
0	Biofeedback	0	Preventive	0	Joy	0	Vascular	0	Butalbital	0	Impact	1	Drive	0	Peer	0	Prophylaxis	0	Angiotensin	0	Toxin	0	Duration	0	Sad	0	minister	0	Hurts	1	Incidence	0	Testing	0	stigma	0	Aggravate	0	Calcium channel blocker	0	faith	0	Adverse effect	0	Confuse	0	Anger	0	Patterns	0	Quality of life	0	Suicide	0	Trigger point	0	Agonist	0	Motion sickness	0	Acute migraine	0	Steroid	0	Anti-inflammatory	0	CAT scan	0	NSAID	0	Shock	0	Epilepsy	0	Headache diary	0	Sinus headache	0	Weight loss	0	Cervical spine	0	boss	0	Characteristic	0	Peer support	0	Facial pain	0	Grandmother	0	Glutamate	0	Vestibular migraine	0	Upset	0	regime	0	vacation	0	Accident	0	Trauma	0	Neck muscle	0	Population	0	Sensitivity to sound	0
0	Biofeedback	0	Preventive	0	Joy	0	Vascular	0	Butalbital	0	Impact	0	Drive	0	Peer	0	Prophylaxis	0	Angiotensin	0	Toxin	0	Duration	0	Sad	0	minister	1	Hurts	0	Incidence	0	Testing	0	stigma	0	Aggravate	0	Calcium channel blocker	0	faith	0	Adverse effect	0	Confuse	0	Anger	0	Patterns	0	Quality of life	0	Suicide	0	Trigger point	0	Agonist	0	Motion sickness	0	Acute migraine	0	Steroid	0	Anti-inflammatory	0	CAT scan	0	NSAID	0	Shock	0	Epilepsy	0	Headache diary	0	Sinus headache	0	Weight loss	0	Cervical spine	0	boss	0	Characteristic	0	Peer support	0	Facial pain	0	Grandmother	0	Glutamate	0	Vestibular migraine	0	Upset	0	regime	0	vacation	0	Accident	0	Trauma	0	Neck muscle	0	Population	0	Sensitivity to sound	0
0	Biofeedback	0	Preventive	0	Joy	0	Vascular	0	Butalbital	0	Impact	0	Drive	0	Peer	0	Prophylaxis	0	Angiotensin	0	Toxin	0	Duration	0	Sad	0	minister	0	Hurts	1	Incidence	1	Testing	0	stigma	0	Aggravate	0	Calcium channel blocker	0	faith	0	Adverse effect	0	Confuse	1	Anger	0	Patterns	0	Quality of life	0	Suicide	0	Trigger point	0	Agonist	0	Motion sickness	0	Acute migraine	0	Steroid	0	Anti-inflammatory	0	CAT scan	0	NSAID	0	Shock	0	Epilepsy	0	Headache diary	0	Sinus headache	0	Weight loss	0	Cervical spine	0	boss	0	Characteristic	0	Peer support	0	Facial pain	0	Grandmother	0	Glutamate	0	Vestibular migraine	2	Upset	0	regime	0	vacation	0	Accident	0	Trauma	0	Neck muscle	0	Population	0	Sensitivity to sound	0
0	Biofeedback	0	Preventive	0	Joy	0	Vascular	0	Butalbital	3	Impact	0	Drive	0	Peer	0	Prophylaxis	0	Angiotensin	0	Toxin	0	Duration	0	Sad	0	minister	0	Hurts	0	Incidence	0	Testing	0	stigma	0	Aggravate	0	Calcium channel blocker	0	faith	0	Adverse effect	0	Confuse	0	Anger	2	Patterns	0	Quality of life	0	Suicide	1	Trigger point	0	Agonist	0	Motion sickness	0	Acute migraine	0	Steroid	1	Anti-inflammatory	1	CAT scan	0	NSAID	0	Shock	0	Epilepsy	0	Headache diary	0	Sinus headache	0	Weight loss	0	Cervical spine	0	boss	0	Characteristic	0	Peer support	0	Facial pain	0	Grandmother	0	Glutamate	0	Vestibular migraine	0	Upset	0	regime	0	vacation	0	Accident	0	Trauma	0	Neck muscle	0	Population	0	Sensitivity to sound	0
0	Biofeedback	0	Preventive	0	Joy	0	Vascular	0	Butalbital	0	Impact	0	Drive	0	Peer	0	Prophylaxis	0	Angiotensin	0	Toxin	0	Duration	0	Sad	0	minister	0	Hurts	1	Incidence	0	Testing	0	stigma	0	Aggravate	0	Calcium channel blocker	0	faith	0	Adverse effect	0	Confuse	0	Anger	0	Patterns	0	Quality of life	0	Suicide	0	Trigger point	0	Agonist	0	Motion sickness	0	Acute migraine	0	Steroid	0	Anti-inflammatory	0	CAT scan	2	NSAID	0	Shock	0	Epilepsy	0	Headache diary	0	Sinus headache	0	Weight loss	0	Cervical spine	0	boss	0	Characteristic	0	Peer support	0	Facial pain	0	Grandmother	0	Glutamate	0	Vestibular migraine	0	Upset	0	regime	0	vacation	0	Accident	0	Trauma	0	Neck muscle	0	Population	0	Sensitivity to sound	0
0	Biofeedback	0	Preventive	0	Joy	0	Vascular	0	Butalbital	0	Impact	0	Drive	0	Peer	0	Prophylaxis	0	Angiotensin	0	Toxin	0	Duration	0	Sad	0	minister	0	Hurts	0	Incidence	0	Testing	0	stigma	0	Aggravate	0	Calcium channel blocker	0	faith	0	Adverse effect	0	Confuse	0	Anger	1	Patterns	0	Quality of life	0	Suicide	0	Trigger point	0	Agonist	0	Motion sickness	0	Acute migraine	2	Steroid	0	Anti-inflammatory	0	CAT scan	0	NSAID	0	Shock	0	Epilepsy	0	Headache diary	0	Sinus headache	0	Weight loss	0	Cervical spine	0	boss	0	Characteristic	0	Peer support	0	Facial pain	0	Grandmother	0	Glutamate	0	Vestibular migraine	0	Upset	0	regime	0	vacation	0	Accident	0	Trauma	0	Neck muscle	0	Population	0	Sensitivity to sound	0
1	Biofeedback	0	Preventive	0	Joy	0	Vascular	1	Butalbital	0	Impact	0	Drive	0	Peer	0	Prophylaxis	0	Angiotensin	0	Toxin	0	Duration	0	Sad	1	minister	0	Hurts	0	Incidence	0	Testing	0	stigma	0	Aggravate	0	Calcium channel blocker	0	faith	0	Adverse effect	4	Confuse	0	Anger	0	Patterns	0	Quality of life	0	Suicide	0	Trigger point	0	Agonist	0	Motion sickness	0	Acute migraine	0	Steroid	0	Anti-inflammatory	0	CAT scan	0	NSAID	0	Shock	0	Epilepsy	0	Headache diary	0	Sinus headache	0	Weight loss	0	Cervical spine	0	boss	0	Characteristic	0	Peer support	0	Facial pain	0	Grandmother	3	Glutamate	1	Vestibular migraine	0	Upset	0	regime	0	vacation	0	Accident	0	Trauma	0	Neck muscle	0	Population	0	Sensitivity to sound	0
0	Biofeedback	0	Preventive	0	Joy	0	Vascular	0	Butalbital	0	Impact	0	Drive	0	Peer	0	Prophylaxis	0	Angiotensin	0	Toxin	0	Duration	0	Sad	0	minister	0	Hurts	0	Incidence	0	Testing	0	stigma	0	Aggravate	0	Calcium channel blocker	0	faith	0	Adverse effect	0	Confuse	0	Anger	0	Patterns	0	Quality of life	0	Suicide	0	Trigger point	0	Agonist	0	Motion sickness	0	Acute migraine	0	Steroid	0	Anti-inflammatory	0	CAT scan	0	NSAID	0	Shock	0	Epilepsy	0	Headache diary	0	Sinus headache	0	Weight loss	0	Cervical spine	0	boss	0	Characteristic	0	Peer support	0	Facial pain	0	Grandmother	0	Glutamate	0	Vestibular migraine	0	Upset	0	regime	0	vacation	0	Accident	0	Trauma	0	Neck muscle	0	Population	0	Sensitivity to sound	0
0	Biofeedback	0	Preventive	0	Joy	0	Vascular	0	Butalbital	0	Impact	0	Drive	0	Peer	0	Prophylaxis	0	Angiotensin	0	Toxin	0	Duration	0	Sad	0	minister	0	Hurts	0	Incidence	0	Testing	0	stigma	0	Aggravate	0	Calcium channel blocker	0	faith	0	Adverse effect	0	Confuse	0	Anger	0	Patterns	0	Quality of life	0	Suicide	0	Trigger point	0	Agonist	0	Motion sickness	0	Acute migraine	0	Steroid	0	Anti-inflammatory	1	CAT scan	0	NSAID	0	Shock	0	Epilepsy	0	Headache diary	0	Sinus headache	0	Weight loss	0	Cervical spine	0	boss	0	Characteristic	0	Peer support	0	Facial pain	0	Grandmother	0	Glutamate	0	Vestibular migraine	0	Upset	0	regime	0	vacation	0	Accident	0	Trauma	0	Neck muscle	0	Population	0	Sensitivity to sound	0
0	Biofeedback	0	Preventive	0	Joy	0	Vascular	0	Butalbital	0	Impact	0	Drive	0	Peer	0	Prophylaxis	0	Angiotensin	0	Toxin	0	Duration	0	Sad	0	minister	0	Hurts	0	Incidence	0	Testing	0	stigma	0	Aggravate	0	Calcium channel blocker	2	faith	1	Adverse effect	0	Confuse	0	Anger	0	Patterns	0	Quality of life	0	Suicide	0	Trigger point	0	Agonist	0	Motion sickness	0	Acute migraine	0	Steroid	0	Anti-inflammatory	0	CAT scan	0	NSAID	0	Shock	0	Epilepsy	0	Headache diary	0	Sinus headache	0	Weight loss	0	Cervical spine	0	boss	0	Characteristic	0	Peer support	0	Facial pain	3	Grandmother	0	Glutamate	0	Vestibular migraine	0	Upset	0	regime	0	vacation	0	Accident	0	Trauma	0	Neck muscle	0	Population	0	Sensitivity to sound	0
0	Biofeedback	0	Preventive	0	Joy	0	Vascular	0	Butalbital	0	Impact	1	Drive	0	Peer	0	Prophylaxis	0	Angiotensin	0	Toxin	0	Duration	0	Sad	0	minister	0	Hurts	0	Incidence	0	Testing	0	stigma	0	Aggravate	0	Calcium channel blocker	0	faith	0	Adverse effect	0	Confuse	0	Anger	0	Patterns	0	Quality of life	0	Suicide	0	Trigger point	0	Agonist	0	Motion sickness	0	Acute migraine	1	Steroid	0	Anti-inflammatory	0	CAT scan	0	NSAID	0	Shock	0	Epilepsy	0	Headache diary	0	Sinus headache	0	Weight loss	0	Cervical spine	0	boss	0	Characteristic	0	Peer support	0	Facial pain	0	Grandmother	0	Glutamate	0	Vestibular migraine	0	Upset	0	regime	0	vacation	0	Accident	0	Trauma	0	Neck muscle	0	Population	0	Sensitivity to sound	0
0	Biofeedback	0	Preventive	0	Joy	0	Vascular	0	Butalbital	0	Impact	0	Drive	0	Peer	0	Prophylaxis	0	Angiotensin	0	Toxin	0	Duration	0	Sad	0	minister	0	Hurts	0	Incidence	0	Testing	0	stigma	0	Aggravate	0	Calcium channel blocker	0	faith	0	Adverse effect	0	Confuse	0	Anger	0	Patterns	0	Quality of life	0	Suicide	0	Trigger point	0	Agonist	0	Motion sickness	0	Acute migraine	0	Steroid	0	Anti-inflammatory	0	CAT scan	0	NSAID	0	Shock	0	Epilepsy	0	Headache diary	0	Sinus headache	0	Weight loss	0	Cervical spine	0	boss	0	Characteristic	0	Peer support	0	Facial pain	0	Grandmother	0	Glutamate	0	Vestibular migraine	0	Upset	1	regime	0	vacation	0	Accident	0	Trauma	0	Neck muscle	0	Population	0	Sensitivity to sound	0
0	Biofeedback	0	Preventive	0	Joy	1	Vascular	0	Butalbital	0	Impact	0	Drive	0	Peer	6	Prophylaxis	0	Angiotensin	0	Toxin	0	Duration	0	Sad	0	minister	0	Hurts	0	Incidence	0	Testing	0	stigma	0	Aggravate	0	Calcium channel blocker	0	faith	0	Adverse effect	0	Confuse	0	Anger	0	Patterns	0	Quality of life	0	Suicide	0	Trigger point	0	Agonist	0	Motion sickness	0	Acute migraine	0	Steroid	0	Anti-inflammatory	0	CAT scan	0	NSAID	0	Shock	0	Epilepsy	0	Headache diary	0	Sinus headache	0	Weight loss	0	Cervical spine	0	boss	0	Characteristic	0	Peer support	4	Facial pain	0	Grandmother	0	Glutamate	0	Vestibular migraine	0	Upset	0	regime	0	vacation	0	Accident	0	Trauma	2	Neck muscle	0	Population	2	Sensitivity to sound	0
0	Biofeedback	0	Preventive	0	Joy	0	Vascular	0	Butalbital	0	Impact	0	Drive	0	Peer	0	Prophylaxis	0	Angiotensin	0	Toxin	0	Duration	0	Sad	0	minister	0	Hurts	0	Incidence	0	Testing	0	stigma	0	Aggravate	0	Calcium channel blocker	0	faith	0	Adverse effect	0	Confuse	0	Anger	0	Patterns	0	Quality of life	0	Suicide	0	Trigger point	0	Agonist	0	Motion sickness	0	Acute migraine	0	Steroid	0	Anti-inflammatory	0	CAT scan	0	NSAID	0	Shock	0	Epilepsy	0	Headache diary	0	Sinus headache	0	Weight loss	0	Cervical spine	0	boss	0	Characteristic	0	Peer support	0	Facial pain	0	Grandmother	0	Glutamate	0	Vestibular migraine	0	Upset	0	regime	0	vacation	0	Accident	0	Trauma	0	Neck muscle	0	Population	0	Sensitivity to sound	0
0	Biofeedback	0	Preventive	0	Joy	0	Vascular	0	Butalbital	0	Impact	0	Drive	0	Peer	0	Prophylaxis	0	Angiotensin	0	Toxin	0	Duration	0	Sad	0	minister	0	Hurts	0	Incidence	0	Testing	0	stigma	0	Aggravate	0	Calcium channel blocker	0	faith	0	Adverse effect	0	Confuse	0	Anger	0	Patterns	0	Quality of life	0	Suicide	0	Trigger point	0	Agonist	0	Motion sickness	0	Acute migraine	0	Steroid	0	Anti-inflammatory	0	CAT scan	0	NSAID	0	Shock	0	Epilepsy	0	Headache diary	0	Sinus headache	0	Weight loss	0	Cervical spine	0	boss	0	Characteristic	0	Peer support	0	Facial pain	0	Grandmother	0	Glutamate	0	Vestibular migraine	0	Upset	0	regime	0	vacation	0	Accident	0	Trauma	0	Neck muscle	0	Population	0	Sensitivity to sound	0
0	Biofeedback	0	Preventive	0	Joy	0	Vascular	0	Butalbital	0	Impact	0	Drive	0	Peer	0	Prophylaxis	0	Angiotensin	0	Toxin	0	Duration	0	Sad	0	minister	0	Hurts	0	Incidence	0	Testing	0	stigma	0	Aggravate	0	Calcium channel blocker	0	faith	0	Adverse effect	0	Confuse	0	Anger	0	Patterns	0	Quality of life	0	Suicide	0	Trigger point	0	Agonist	0	Motion sickness	0	Acute migraine	0	Steroid	0	Anti-inflammatory	0	CAT scan	0	NSAID	0	Shock	0	Epilepsy	0	Headache diary	0	Sinus headache	0	Weight loss	0	Cervical spine	0	boss	0	Characteristic	0	Peer support	0	Facial pain	0	Grandmother	0	Glutamate	0	Vestibular migraine	0	Upset	0	regime	0	vacation	0	Accident	0	Trauma	0	Neck muscle	0	Population	0	Sensitivity to sound	0
2	Biofeedback	0	Preventive	1	Joy	0	Vascular	0	Butalbital	0	Impact	0	Drive	0	Peer	0	Prophylaxis	0	Angiotensin	0	Toxin	0	Duration	3	Sad	0	minister	0	Hurts	0	Incidence	0	Testing	0	stigma	0	Aggravate	3	Calcium channel blocker	0	faith	0	Adverse effect	0	Confuse	0	Anger	0	Patterns	0	Quality of life	0	Suicide	0	Trigger point	0	Agonist	0	Motion sickness	0	Acute migraine	0	Steroid	0	Anti-inflammatory	0	CAT scan	0	NSAID	0	Shock	0	Epilepsy	0	Headache diary	0	Sinus headache	0	Weight loss	0	Cervical spine	4	boss	2	Characteristic	4	Peer support	0	Facial pain	0	Grandmother	0	Glutamate	0	Vestibular migraine	0	Upset	0	regime	0	vacation	0	Accident	0	Trauma	0	Neck muscle	0	Population	0	Sensitivity to sound	0
1	Biofeedback	0	Preventive	1	Joy	0	Vascular	1	Butalbital	0	Impact	0	Drive	0	Peer	0	Prophylaxis	0	Angiotensin	0	Toxin	0	Duration	0	Sad	0	minister	0	Hurts	0	Incidence	0	Testing	0	stigma	0	Aggravate	0	Calcium channel blocker	0	faith	0	Adverse effect	0	Confuse	0	Anger	0	Patterns	0	Quality of life	0	Suicide	0	Trigger point	0	Agonist	0	Motion sickness	5	Acute migraine	0	Steroid	0	Anti-inflammatory	0	CAT scan	0	NSAID	0	Shock	0	Epilepsy	0	Headache diary	0	Sinus headache	0	Weight loss	0	Cervical spine	0	boss	0	Characteristic	0	Peer support	0	Facial pain	0	Grandmother	0	Glutamate	0	Vestibular migraine	0	Upset	0	regime	0	vacation	0	Accident	0	Trauma	0	Neck muscle	0	Population	0	Sensitivity to sound	0
1	Biofeedback	0	Preventive	0	Joy	0	Vascular	0	Butalbital	0	Impact	0	Drive	0	Peer	0	Prophylaxis	0	Angiotensin	0	Toxin	0	Duration	0	Sad	0	minister	0	Hurts	0	Incidence	0	Testing	1	stigma	0	Aggravate	0	Calcium channel blocker	0	faith	0	Adverse effect	0	Confuse	0	Anger	0	Patterns	0	Quality of life	0	Suicide	0	Trigger point	0	Agonist	0	Motion sickness	0	Acute migraine	0	Steroid	0	Anti-inflammatory	0	CAT scan	0	NSAID	0	Shock	0	Epilepsy	0	Headache diary	0	Sinus headache	0	Weight loss	0	Cervical spine	0	boss	0	Characteristic	0	Peer support	0	Facial pain	0	Grandmother	0	Glutamate	0	Vestibular migraine	0	Upset	0	regime	0	vacation	0	Accident	0	Trauma	0	Neck muscle	0	Population	0	Sensitivity to sound	0
0	Biofeedback	0	Preventive	0	Joy	0	Vascular	0	Butalbital	0	Impact	0	Drive	0	Peer	0	Prophylaxis	0	Angiotensin	0	Toxin	0	Duration	0	Sad	0	minister	0	Hurts	0	Incidence	0	Testing	0	stigma	0	Aggravate	0	Calcium channel blocker	0	faith	0	Adverse effect	0	Confuse	0	Anger	0	Patterns	0	Quality of life	0	Suicide	0	Trigger point	0	Agonist	0	Motion sickness	0	Acute migraine	0	Steroid	0	Anti-inflammatory	0	CAT scan	0	NSAID	0	Shock	0	Epilepsy	0	Headache diary	0	Sinus headache	0	Weight loss	0	Cervical spine	0	boss	0	Characteristic	0	Peer support	0	Facial pain	0	Grandmother	0	Glutamate	0	Vestibular migraine	0	Upset	0	regime	0	vacation	0	Accident	0	Trauma	0	Neck muscle	0	Population	0	Sensitivity to sound	0
1	Biofeedback	0	Preventive	0	Joy	0	Vascular	0	Butalbital	0	Impact	0	Drive	0	Peer	0	Prophylaxis	0	Angiotensin	0	Toxin	1	Duration	0	Sad	0	minister	0	Hurts	0	Incidence	0	Testing	0	stigma	0	Aggravate	0	Calcium channel blocker	0	faith	0	Adverse effect	0	Confuse	0	Anger	0	Patterns	0	Quality of life	0	Suicide	0	Trigger point	0	Agonist	0	Motion sickness	0	Acute migraine	0	Steroid	0	Anti-inflammatory	0	CAT scan	0	NSAID	0	Shock	0	Epilepsy	0	Headache diary	0	Sinus headache	0	Weight loss	0	Cervical spine	0	boss	0	Characteristic	0	Peer support	0	Facial pain	0	Grandmother	0	Glutamate	0	Vestibular migraine	0	Upset	0	regime	0	vacation	0	Accident	1	Trauma	0	Neck muscle	0	Population	0	Sensitivity to sound	0
0	Biofeedback	0	Preventive	0	Joy	0	Vascular	0	Butalbital	0	Impact	0	Drive	0	Peer	0	Prophylaxis	0	Angiotensin	0	Toxin	0	Duration	0	Sad	0	minister	0	Hurts	0	Incidence	0	Testing	0	stigma	0	Aggravate	0	Calcium channel blocker	0	faith	0	Adverse effect	0	Confuse	0	Anger	0	Patterns	0	Quality of life	0	Suicide	0	Trigger point	0	Agonist	0	Motion sickness	0	Acute migraine	0	Steroid	0	Anti-inflammatory	0	CAT scan	0	NSAID	0	Shock	0	Epilepsy	0	Headache diary	0	Sinus headache	0	Weight loss	0	Cervical spine	0	boss	0	Characteristic	0	Peer support	0	Facial pain	0	Grandmother	0	Glutamate	0	Vestibular migraine	0	Upset	0	regime	0	vacation	0	Accident	0	Trauma	0	Neck muscle	0	Population	0	Sensitivity to sound	0
0	Biofeedback	0	Preventive	0	Joy	0	Vascular	0	Butalbital	0	Impact	0	Drive	0	Peer	0	Prophylaxis	0	Angiotensin	0	Toxin	0	Duration	0	Sad	0	minister	0	Hurts	0	Incidence	0	Testing	0	stigma	0	Aggravate	0	Calcium channel blocker	0	faith	0	Adverse effect	0	Confuse	0	Anger	0	Patterns	0	Quality of life	0	Suicide	0	Trigger point	0	Agonist	0	Motion sickness	0	Acute migraine	0	Steroid	0	Anti-inflammatory	0	CAT scan	0	NSAID	0	Shock	0	Epilepsy	0	Headache diary	0	Sinus headache	0	Weight loss	0	Cervical spine	0	boss	0	Characteristic	0	Peer support	0	Facial pain	0	Grandmother	0	Glutamate	0	Vestibular migraine	0	Upset	0	regime	0	vacation	0	Accident	0	Trauma	0	Neck muscle	0	Population	0	Sensitivity to sound	0
1	Biofeedback	8	Preventive	2	Joy	5	Vascular	2	Butalbital	5	Impact	1	Drive	2	Peer	0	Prophylaxis	0	Angiotensin	0	Toxin	2	Duration	3	Sad	4	minister	3	Hurts	3	Incidence	4	Testing	1	stigma	4	Aggravate	1	Calcium channel blocker	1	faith	5	Adverse effect	0	Confuse	4	Anger	0	Patterns	2	Quality of life	1	Suicide	0	Trigger point	5	Agonist	1	Motion sickness	0	Acute migraine	0	Steroid	4	Anti-inflammatory	2	CAT scan	0	NSAID	1	Shock	4	Epilepsy	4	Headache diary	1	Sinus headache	1	Weight loss	2	Cervical spine	0	boss	0	Characteristic	0	Peer support	0	Facial pain	0	Grandmother	1	Glutamate	2	Vestibular migraine	0	Upset	1	regime	1	vacation	2	Accident	1	Trauma	0	Neck muscle	3	Population	0	Sensitivity to sound	3
0	Biofeedback	0	Preventive	0	Joy	0	Vascular	0	Butalbital	0	Impact	0	Drive	0	Peer	0	Prophylaxis	0	Angiotensin	0	Toxin	0	Duration	0	Sad	0	minister	0	Hurts	0	Incidence	0	Testing	0	stigma	0	Aggravate	0	Calcium channel blocker	0	faith	0	Adverse effect	0	Confuse	0	Anger	0	Patterns	0	Quality of life	0	Suicide	0	Trigger point	0	Agonist	0	Motion sickness	0	Acute migraine	0	Steroid	0	Anti-inflammatory	0	CAT scan	0	NSAID	0	Shock	0	Epilepsy	0	Headache diary	0	Sinus headache	0	Weight loss	0	Cervical spine	0	boss	0	Characteristic	0	Peer support	0	Facial pain	0	Grandmother	0	Glutamate	0	Vestibular migraine	0	Upset	0	regime	0	vacation	0	Accident	0	Trauma	0	Neck muscle	0	Population	0	Sensitivity to sound	0
0	Biofeedback	0	Preventive	0	Joy	0	Vascular	0	Butalbital	0	Impact	1	Drive	0	Peer	0	Prophylaxis	0	Angiotensin	0	Toxin	0	Duration	0	Sad	0	minister	0	Hurts	0	Incidence	0	Testing	0	stigma	0	Aggravate	0	Calcium channel blocker	0	faith	0	Adverse effect	0	Confuse	0	Anger	0	Patterns	0	Quality of life	1	Suicide	0	Trigger point	0	Agonist	0	Motion sickness	0	Acute migraine	0	Steroid	0	Anti-inflammatory	0	CAT scan	0	NSAID	0	Shock	0	Epilepsy	0	Headache diary	1	Sinus headache	0	Weight loss	0	Cervical spine	0	boss	0	Characteristic	0	Peer support	0	Facial pain	0	Grandmother	0	Glutamate	0	Vestibular migraine	0	Upset	0	regime	0	vacation	0	Accident	0	Trauma	1	Neck muscle	0	Population	0	Sensitivity to sound	0
0	Biofeedback	0	Preventive	0	Joy	0	Vascular	0	Butalbital	0	Impact	0	Drive	0	Peer	0	Prophylaxis	0	Angiotensin	0	Toxin	0	Duration	0	Sad	0	minister	0	Hurts	0	Incidence	0	Testing	0	stigma	0	Aggravate	0	Calcium channel blocker	0	faith	0	Adverse effect	0	Confuse	0	Anger	0	Patterns	0	Quality of life	0	Suicide	0	Trigger point	0	Agonist	0	Motion sickness	0	Acute migraine	0	Steroid	0	Anti-inflammatory	0	CAT scan	0	NSAID	0	Shock	0	Epilepsy	0	Headache diary	0	Sinus headache	0	Weight loss	0	Cervical spine	0	boss	0	Characteristic	0	Peer support	0	Facial pain	0	Grandmother	0	Glutamate	0	Vestibular migraine	0	Upset	0	regime	0	vacation	0	Accident	0	Trauma	0	Neck muscle	0	Population	0	Sensitivity to sound	0
0	Biofeedback	0	Preventive	3	Joy	0	Vascular	1	Butalbital	0	Impact	0	Drive	0	Peer	0	Prophylaxis	5	Angiotensin	7	Toxin	1	Duration	0	Sad	0	minister	0	Hurts	0	Incidence	0	Testing	0	stigma	0	Aggravate	0	Calcium channel blocker	2	faith	0	Adverse effect	0	Confuse	0	Anger	2	Patterns	1	Quality of life	0	Suicide	0	Trigger point	0	Agonist	2	Motion sickness	0	Acute migraine	0	Steroid	0	Anti-inflammatory	0	CAT scan	0	NSAID	0	Shock	0	Epilepsy	0	Headache diary	0	Sinus headache	0	Weight loss	0	Cervical spine	0	boss	0	Characteristic	0	Peer support	0	Facial pain	0	Grandmother	0	Glutamate	0	Vestibular migraine	0	Upset	1	regime	0	vacation	0	Accident	0	Trauma	0	Neck muscle	0	Population	1	Sensitivity to sound	0
0	Biofeedback	0	Preventive	0	Joy	0	Vascular	0	Butalbital	0	Impact	1	Drive	0	Peer	0	Prophylaxis	0	Angiotensin	0	Toxin	0	Duration	0	Sad	0	minister	0	Hurts	0	Incidence	0	Testing	0	stigma	0	Aggravate	0	Calcium channel blocker	0	faith	0	Adverse effect	0	Confuse	0	Anger	0	Patterns	0	Quality of life	0	Suicide	0	Trigger point	0	Agonist	0	Motion sickness	0	Acute migraine	0	Steroid	0	Anti-inflammatory	0	CAT scan	0	NSAID	0	Shock	0	Epilepsy	0	Headache diary	0	Sinus headache	0	Weight loss	0	Cervical spine	0	boss	0	Characteristic	0	Peer support	0	Facial pain	0	Grandmother	0	Glutamate	0	Vestibular migraine	0	Upset	0	regime	0	vacation	0	Accident	0	Trauma	0	Neck muscle	0	Population	0	Sensitivity to sound	0
0	Biofeedback	0	Preventive	0	Joy	0	Vascular	0	Butalbital	0	Impact	0	Drive	0	Peer	0	Prophylaxis	0	Angiotensin	0	Toxin	0	Duration	0	Sad	0	minister	0	Hurts	0	Incidence	0	Testing	0	stigma	0	Aggravate	0	Calcium channel blocker	0	faith	0	Adverse effect	0	Confuse	0	Anger	0	Patterns	0	Quality of life	0	Suicide	0	Trigger point	0	Agonist	0	Motion sickness	0	Acute migraine	0	Steroid	0	Anti-inflammatory	0	CAT scan	0	NSAID	0	Shock	0	Epilepsy	0	Headache diary	0	Sinus headache	0	Weight loss	0	Cervical spine	0	boss	0	Characteristic	0	Peer support	0	Facial pain	0	Grandmother	0	Glutamate	0	Vestibular migraine	0	Upset	0	regime	0	vacation	0	Accident	0	Trauma	0	Neck muscle	0	Population	0	Sensitivity to sound	0
0	Biofeedback	0	Preventive	0	Joy	0	Vascular	0	Butalbital	0	Impact	0	Drive	0	Peer	0	Prophylaxis	0	Angiotensin	0	Toxin	0	Duration	0	Sad	0	minister	0	Hurts	0	Incidence	0	Testing	0	stigma	0	Aggravate	0	Calcium channel blocker	1	faith	0	Adverse effect	0	Confuse	0	Anger	0	Patterns	0	Quality of life	0	Suicide	0	Trigger point	0	Agonist	0	Motion sickness	0	Acute migraine	0	Steroid	0	Anti-inflammatory	0	CAT scan	0	NSAID	0	Shock	0	Epilepsy	0	Headache diary	0	Sinus headache	0	Weight loss	0	Cervical spine	0	boss	0	Characteristic	0	Peer support	0	Facial pain	0	Grandmother	0	Glutamate	0	Vestibular migraine	0	Upset	0	regime	0	vacation	0	Accident	0	Trauma	0	Neck muscle	0	Population	0	Sensitivity to sound	0
0	Biofeedback	0	Preventive	0	Joy	0	Vascular	0	Butalbital	0	Impact	0	Drive	0	Peer	0	Prophylaxis	0	Angiotensin	0	Toxin	1	Duration	0	Sad	0	minister	0	Hurts	0	Incidence	0	Testing	0	stigma	0	Aggravate	0	Calcium channel blocker	0	faith	0	Adverse effect	0	Confuse	0	Anger	0	Patterns	0	Quality of life	0	Suicide	0	Trigger point	0	Agonist	0	Motion sickness	0	Acute migraine	0	Steroid	0	Anti-inflammatory	0	CAT scan	0	NSAID	1	Shock	0	Epilepsy	0	Headache diary	0	Sinus headache	0	Weight loss	0	Cervical spine	0	boss	0	Characteristic	0	Peer support	0	Facial pain	0	Grandmother	0	Glutamate	0	Vestibular migraine	0	Upset	0	regime	0	vacation	0	Accident	0	Trauma	0	Neck muscle	0	Population	0	Sensitivity to sound	0
0	Biofeedback	0	Preventive	0	Joy	0	Vascular	0	Butalbital	0	Impact	0	Drive	0	Peer	0	Prophylaxis	0	Angiotensin	0	Toxin	0	Duration	0	Sad	0	minister	0	Hurts	0	Incidence	0	Testing	0	stigma	0	Aggravate	1	Calcium channel blocker	0	faith	0	Adverse effect	0	Confuse	0	Anger	0	Patterns	0	Quality of life	0	Suicide	0	Trigger point	0	Agonist	0	Motion sickness	0	Acute migraine	0	Steroid	0	Anti-inflammatory	1	CAT scan	1	NSAID	0	Shock	0	Epilepsy	0	Headache diary	0	Sinus headache	0	Weight loss	0	Cervical spine	0	boss	0	Characteristic	0	Peer support	0	Facial pain	0	Grandmother	0	Glutamate	0	Vestibular migraine	0	Upset	0	regime	0	vacation	0	Accident	0	Trauma	0	Neck muscle	0	Population	0	Sensitivity to sound	0
0	Biofeedback	0	Preventive	0	Joy	0	Vascular	0	Butalbital	0	Impact	0	Drive	0	Peer	0	Prophylaxis	2	Angiotensin	0	Toxin	0	Duration	0	Sad	0	minister	0	Hurts	0	Incidence	0	Testing	0	stigma	0	Aggravate	0	Calcium channel blocker	0	faith	0	Adverse effect	0	Confuse	0	Anger	0	Patterns	0	Quality of life	0	Suicide	0	Trigger point	0	Agonist	2	Motion sickness	0	Acute migraine	0	Steroid	0	Anti-inflammatory	0	CAT scan	0	NSAID	0	Shock	0	Epilepsy	0	Headache diary	0	Sinus headache	0	Weight loss	0	Cervical spine	0	boss	0	Characteristic	0	Peer support	0	Facial pain	0	Grandmother	0	Glutamate	0	Vestibular migraine	0	Upset	0	regime	0	vacation	0	Accident	0	Trauma	0	Neck muscle	0	Population	0	Sensitivity to sound	0
0	Biofeedback	0	Preventive	0	Joy	0	Vascular	0	Butalbital	0	Impact	0	Drive	0	Peer	0	Prophylaxis	0	Angiotensin	0	Toxin	0	Duration	0	Sad	0	minister	0	Hurts	0	Incidence	0	Testing	0	stigma	0	Aggravate	0	Calcium channel blocker	0	faith	0	Adverse effect	0	Confuse	0	Anger	0	Patterns	0	Quality of life	0	Suicide	0	Trigger point	0	Agonist	0	Motion sickness	0	Acute migraine	0	Steroid	0	Anti-inflammatory	0	CAT scan	0	NSAID	0	Shock	0	Epilepsy	0	Headache diary	0	Sinus headache	0	Weight loss	0	Cervical spine	0	boss	0	Characteristic	0	Peer support	0	Facial pain	0	Grandmother	0	Glutamate	0	Vestibular migraine	0	Upset	0	regime	0	vacation	0	Accident	0	Trauma	0	Neck muscle	0	Population	0	Sensitivity to sound	0
0	Biofeedback	0	Preventive	0	Joy	0	Vascular	0	Butalbital	0	Impact	0	Drive	0	Peer	0	Prophylaxis	0	Angiotensin	0	Toxin	0	Duration	0	Sad	0	minister	0	Hurts	0	Incidence	0	Testing	0	stigma	0	Aggravate	0	Calcium channel blocker	0	faith	0	Adverse effect	2	Confuse	0	Anger	0	Patterns	0	Quality of life	0	Suicide	0	Trigger point	0	Agonist	0	Motion sickness	0	Acute migraine	0	Steroid	0	Anti-inflammatory	0	CAT scan	0	NSAID	2	Shock	0	Epilepsy	0	Headache diary	0	Sinus headache	0	Weight loss	0	Cervical spine	0	boss	0	Characteristic	0	Peer support	0	Facial pain	0	Grandmother	0	Glutamate	0	Vestibular migraine	0	Upset	0	regime	0	vacation	0	Accident	0	Trauma	0	Neck muscle	0	Population	0	Sensitivity to sound	0
0	Biofeedback	0	Preventive	0	Joy	0	Vascular	0	Butalbital	0	Impact	0	Drive	0	Peer	0	Prophylaxis	0	Angiotensin	0	Toxin	0	Duration	0	Sad	0	minister	0	Hurts	1	Incidence	0	Testing	0	stigma	0	Aggravate	0	Calcium channel blocker	0	faith	0	Adverse effect	0	Confuse	0	Anger	0	Patterns	0	Quality of life	0	Suicide	0	Trigger point	0	Agonist	0	Motion sickness	0	Acute migraine	0	Steroid	0	Anti-inflammatory	0	CAT scan	0	NSAID	0	Shock	0	Epilepsy	0	Headache diary	0	Sinus headache	0	Weight loss	0	Cervical spine	0	boss	0	Characteristic	0	Peer support	0	Facial pain	0	Grandmother	0	Glutamate	0	Vestibular migraine	0	Upset	0	regime	0	vacation	0	Accident	1	Trauma	0	Neck muscle	0	Population	0	Sensitivity to sound	0
0	Biofeedback	0	Preventive	1	Joy	0	Vascular	0	Butalbital	0	Impact	0	Drive	1	Peer	0	Prophylaxis	0	Angiotensin	0	Toxin	0	Duration	0	Sad	0	minister	0	Hurts	0	Incidence	0	Testing	0	stigma	0	Aggravate	0	Calcium channel blocker	0	faith	0	Adverse effect	0	Confuse	0	Anger	0	Patterns	0	Quality of life	0	Suicide	0	Trigger point	0	Agonist	0	Motion sickness	0	Acute migraine	0	Steroid	0	Anti-inflammatory	0	CAT scan	0	NSAID	0	Shock	0	Epilepsy	0	Headache diary	0	Sinus headache	0	Weight loss	0	Cervical spine	0	boss	2	Characteristic	0	Peer support	0	Facial pain	0	Grandmother	0	Glutamate	0	Vestibular migraine	0	Upset	0	regime	1	vacation	0	Accident	0	Trauma	0	Neck muscle	0	Population	0	Sensitivity to sound	0
0	Biofeedback	0	Preventive	0	Joy	0	Vascular	0	Butalbital	0	Impact	1	Drive	0	Peer	0	Prophylaxis	0	Angiotensin	0	Toxin	0	Duration	0	Sad	0	minister	0	Hurts	0	Incidence	0	Testing	0	stigma	1	Aggravate	0	Calcium channel blocker	0	faith	0	Adverse effect	0	Confuse	0	Anger	0	Patterns	0	Quality of life	0	Suicide	0	Trigger point	0	Agonist	0	Motion sickness	0	Acute migraine	0	Steroid	0	Anti-inflammatory	0	CAT scan	0	NSAID	0	Shock	0	Epilepsy	0	Headache diary	0	Sinus headache	0	Weight loss	0	Cervical spine	0	boss	0	Characteristic	0	Peer support	0	Facial pain	0	Grandmother	0	Glutamate	0	Vestibular migraine	0	Upset	0	regime	1	vacation	0	Accident	0	Trauma	0	Neck muscle	0	Population	0	Sensitivity to sound	0
0	Biofeedback	0	Preventive	0	Joy	0	Vascular	0	Butalbital	0	Impact	0	Drive	0	Peer	0	Prophylaxis	0	Angiotensin	0	Toxin	2	Duration	0	Sad	0	minister	0	Hurts	0	Incidence	0	Testing	2	stigma	0	Aggravate	0	Calcium channel blocker	0	faith	0	Adverse effect	0	Confuse	0	Anger	0	Patterns	0	Quality of life	0	Suicide	0	Trigger point	0	Agonist	0	Motion sickness	0	Acute migraine	0	Steroid	0	Anti-inflammatory	0	CAT scan	0	NSAID	0	Shock	0	Epilepsy	0	Headache diary	0	Sinus headache	0	Weight loss	1	Cervical spine	0	boss	0	Characteristic	0	Peer support	0	Facial pain	0	Grandmother	0	Glutamate	0	Vestibular migraine	0	Upset	0	regime	0	vacation	0	Accident	0	Trauma	0	Neck muscle	0	Population	0	Sensitivity to sound	0
0	Biofeedback	0	Preventive	0	Joy	0	Vascular	0	Butalbital	0	Impact	0	Drive	0	Peer	0	Prophylaxis	0	Angiotensin	0	Toxin	0	Duration	0	Sad	0	minister	0	Hurts	0	Incidence	0	Testing	0	stigma	0	Aggravate	0	Calcium channel blocker	0	faith	0	Adverse effect	0	Confuse	0	Anger	0	Patterns	0	Quality of life	0	Suicide	0	Trigger point	0	Agonist	0	Motion sickness	0	Acute migraine	2	Steroid	0	Anti-inflammatory	0	CAT scan	0	NSAID	0	Shock	0	Epilepsy	0	Headache diary	0	Sinus headache	2	Weight loss	1	Cervical spine	0	boss	0	Characteristic	0	Peer support	0	Facial pain	0	Grandmother	0	Glutamate	0	Vestibular migraine	0	Upset	0	regime	0	vacation	0	Accident	0	Trauma	0	Neck muscle	0	Population	0	Sensitivity to sound	0
0	Biofeedback	0	Preventive	0	Joy	0	Vascular	0	Butalbital	0	Impact	0	Drive	0	Peer	0	Prophylaxis	0	Angiotensin	0	Toxin	0	Duration	0	Sad	0	minister	0	Hurts	0	Incidence	0	Testing	0	stigma	0	Aggravate	0	Calcium channel blocker	0	faith	0	Adverse effect	0	Confuse	0	Anger	0	Patterns	0	Quality of life	0	Suicide	0	Trigger point	0	Agonist	0	Motion sickness	0	Acute migraine	0	Steroid	0	Anti-inflammatory	0	CAT scan	0	NSAID	0	Shock	0	Epilepsy	0	Headache diary	0	Sinus headache	0	Weight loss	0	Cervical spine	0	boss	0	Characteristic	0	Peer support	0	Facial pain	0	Grandmother	0	Glutamate	0	Vestibular migraine	2	Upset	0	regime	0	vacation	0	Accident	0	Trauma	0	Neck muscle	0	Population	0	Sensitivity to sound	0
0	Biofeedback	0	Preventive	0	Joy	0	Vascular	0	Butalbital	0	Impact	0	Drive	0	Peer	0	Prophylaxis	0	Angiotensin	0	Toxin	0	Duration	0	Sad	0	minister	0	Hurts	0	Incidence	0	Testing	0	stigma	0	Aggravate	0	Calcium channel blocker	0	faith	0	Adverse effect	0	Confuse	0	Anger	0	Patterns	0	Quality of life	0	Suicide	0	Trigger point	0	Agonist	0	Motion sickness	0	Acute migraine	0	Steroid	0	Anti-inflammatory	0	CAT scan	0	NSAID	0	Shock	0	Epilepsy	0	Headache diary	0	Sinus headache	0	Weight loss	0	Cervical spine	0	boss	0	Characteristic	0	Peer support	0	Facial pain	0	Grandmother	0	Glutamate	0	Vestibular migraine	0	Upset	0	regime	0	vacation	0	Accident	0	Trauma	0	Neck muscle	0	Population	0	Sensitivity to sound	0
0	Biofeedback	0	Preventive	0	Joy	0	Vascular	3	Butalbital	0	Impact	0	Drive	3	Peer	0	Prophylaxis	0	Angiotensin	0	Toxin	0	Duration	0	Sad	0	minister	2	Hurts	0	Incidence	0	Testing	2	stigma	0	Aggravate	1	Calcium channel blocker	0	faith	0	Adverse effect	0	Confuse	0	Anger	0	Patterns	1	Quality of life	0	Suicide	0	Trigger point	0	Agonist	0	Motion sickness	0	Acute migraine	0	Steroid	0	Anti-inflammatory	0	CAT scan	1	NSAID	0	Shock	0	Epilepsy	0	Headache diary	2	Sinus headache	1	Weight loss	0	Cervical spine	0	boss	0	Characteristic	0	Peer support	0	Facial pain	1	Grandmother	0	Glutamate	0	Vestibular migraine	0	Upset	0	regime	0	vacation	0	Accident	0	Trauma	0	Neck muscle	0	Population	0	Sensitivity to sound	0
0	Biofeedback	0	Preventive	0	Joy	0	Vascular	0	Butalbital	0	Impact	0	Drive	0	Peer	0	Prophylaxis	0	Angiotensin	0	Toxin	0	Duration	0	Sad	0	minister	0	Hurts	0	Incidence	0	Testing	0	stigma	0	Aggravate	0	Calcium channel blocker	0	faith	0	Adverse effect	0	Confuse	0	Anger	0	Patterns	0	Quality of life	0	Suicide	0	Trigger point	0	Agonist	0	Motion sickness	0	Acute migraine	0	Steroid	0	Anti-inflammatory	0	CAT scan	0	NSAID	0	Shock	0	Epilepsy	0	Headache diary	0	Sinus headache	0	Weight loss	0	Cervical spine	0	boss	0	Characteristic	0	Peer support	0	Facial pain	0	Grandmother	0	Glutamate	0	Vestibular migraine	0	Upset	0	regime	0	vacation	0	Accident	0	Trauma	0	Neck muscle	0	Population	0	Sensitivity to sound	0
0	Biofeedback	0	Preventive	0	Joy	0	Vascular	0	Butalbital	0	Impact	1	Drive	0	Peer	0	Prophylaxis	0	Angiotensin	0	Toxin	0	Duration	0	Sad	0	minister	1	Hurts	0	Incidence	0	Testing	0	stigma	0	Aggravate	0	Calcium channel blocker	0	faith	0	Adverse effect	0	Confuse	0	Anger	0	Patterns	0	Quality of life	3	Suicide	0	Trigger point	0	Agonist	0	Motion sickness	0	Acute migraine	0	Steroid	0	Anti-inflammatory	0	CAT scan	0	NSAID	0	Shock	0	Epilepsy	0	Headache diary	0	Sinus headache	0	Weight loss	0	Cervical spine	0	boss	0	Characteristic	0	Peer support	0	Facial pain	0	Grandmother	0	Glutamate	0	Vestibular migraine	0	Upset	0	regime	0	vacation	0	Accident	0	Trauma	0	Neck muscle	0	Population	0	Sensitivity to sound	0
0	Biofeedback	0	Preventive	0	Joy	0	Vascular	0	Butalbital	0	Impact	0	Drive	0	Peer	1	Prophylaxis	0	Angiotensin	0	Toxin	0	Duration	1	Sad	0	minister	0	Hurts	0	Incidence	0	Testing	0	stigma	0	Aggravate	0	Calcium channel blocker	0	faith	0	Adverse effect	0	Confuse	0	Anger	0	Patterns	0	Quality of life	0	Suicide	0	Trigger point	0	Agonist	0	Motion sickness	0	Acute migraine	0	Steroid	0	Anti-inflammatory	0	CAT scan	0	NSAID	0	Shock	0	Epilepsy	0	Headache diary	0	Sinus headache	0	Weight loss	0	Cervical spine	0	boss	0	Characteristic	0	Peer support	0	Facial pain	0	Grandmother	0	Glutamate	0	Vestibular migraine	0	Upset	0	regime	0	vacation	0	Accident	0	Trauma	0	Neck muscle	0	Population	0	Sensitivity to sound	0
0	Biofeedback	0	Preventive	0	Joy	0	Vascular	0	Butalbital	0	Impact	0	Drive	0	Peer	0	Prophylaxis	0	Angiotensin	0	Toxin	0	Duration	0	Sad	0	minister	0	Hurts	0	Incidence	0	Testing	0	stigma	0	Aggravate	0	Calcium channel blocker	0	faith	0	Adverse effect	0	Confuse	0	Anger	0	Patterns	0	Quality of life	0	Suicide	0	Trigger point	0	Agonist	0	Motion sickness	0	Acute migraine	0	Steroid	0	Anti-inflammatory	0	CAT scan	0	NSAID	0	Shock	0	Epilepsy	0	Headache diary	0	Sinus headache	0	Weight loss	0	Cervical spine	0	boss	0	Characteristic	0	Peer support	0	Facial pain	0	Grandmother	0	Glutamate	0	Vestibular migraine	0	Upset	0	regime	0	vacation	0	Accident	0	Trauma	0	Neck muscle	0	Population	0	Sensitivity to sound	0
0	Biofeedback	0	Preventive	0	Joy	0	Vascular	0	Butalbital	0	Impact	0	Drive	0	Peer	0	Prophylaxis	0	Angiotensin	0	Toxin	0	Duration	0	Sad	2	minister	0	Hurts	0	Incidence	0	Testing	0	stigma	0	Aggravate	0	Calcium channel blocker	0	faith	0	Adverse effect	0	Confuse	0	Anger	0	Patterns	0	Quality of life	0	Suicide	0	Trigger point	0	Agonist	0	Motion sickness	0	Acute migraine	0	Steroid	0	Anti-inflammatory	0	CAT scan	0	NSAID	0	Shock	0	Epilepsy	0	Headache diary	0	Sinus headache	0	Weight loss	0	Cervical spine	0	boss	0	Characteristic	0	Peer support	0	Facial pain	0	Grandmother	0	Glutamate	0	Vestibular migraine	0	Upset	0	regime	0	vacation	0	Accident	0	Trauma	0	Neck muscle	0	Population	0	Sensitivity to sound	0
1	Biofeedback	0	Preventive	0	Joy	0	Vascular	0	Butalbital	0	Impact	0	Drive	0	Peer	0	Prophylaxis	0	Angiotensin	0	Toxin	0	Duration	0	Sad	0	minister	0	Hurts	0	Incidence	0	Testing	0	stigma	0	Aggravate	0	Calcium channel blocker	0	faith	0	Adverse effect	0	Confuse	0	Anger	0	Patterns	0	Quality of life	0	Suicide	0	Trigger point	0	Agonist	0	Motion sickness	0	Acute migraine	0	Steroid	0	Anti-inflammatory	0	CAT scan	0	NSAID	0	Shock	0	Epilepsy	0	Headache diary	0	Sinus headache	0	Weight loss	0	Cervical spine	0	boss	0	Characteristic	0	Peer support	0	Facial pain	0	Grandmother	0	Glutamate	0	Vestibular migraine	0	Upset	0	regime	0	vacation	0	Accident	0	Trauma	0	Neck muscle	0	Population	0	Sensitivity to sound	0
0	Biofeedback	0	Preventive	0	Joy	2	Vascular	0	Butalbital	0	Impact	0	Drive	0	Peer	0	Prophylaxis	0	Angiotensin	0	Toxin	0	Duration	0	Sad	0	minister	0	Hurts	0	Incidence	0	Testing	0	stigma	1	Aggravate	0	Calcium channel blocker	0	faith	0	Adverse effect	0	Confuse	0	Anger	0	Patterns	0	Quality of life	0	Suicide	0	Trigger point	0	Agonist	0	Motion sickness	0	Acute migraine	0	Steroid	0	Anti-inflammatory	0	CAT scan	0	NSAID	0	Shock	0	Epilepsy	0	Headache diary	0	Sinus headache	0	Weight loss	0	Cervical spine	0	boss	0	Characteristic	0	Peer support	0	Facial pain	0	Grandmother	0	Glutamate	1	Vestibular migraine	0	Upset	0	regime	0	vacation	0	Accident	0	Trauma	0	Neck muscle	0	Population	0	Sensitivity to sound	0
0	Biofeedback	0	Preventive	0	Joy	0	Vascular	0	Butalbital	0	Impact	0	Drive	0	Peer	0	Prophylaxis	0	Angiotensin	0	Toxin	0	Duration	0	Sad	0	minister	0	Hurts	0	Incidence	0	Testing	0	stigma	0	Aggravate	0	Calcium channel blocker	0	faith	0	Adverse effect	0	Confuse	0	Anger	0	Patterns	0	Quality of life	0	Suicide	0	Trigger point	0	Agonist	0	Motion sickness	0	Acute migraine	0	Steroid	0	Anti-inflammatory	0	CAT scan	0	NSAID	0	Shock	0	Epilepsy	0	Headache diary	0	Sinus headache	0	Weight loss	0	Cervical spine	0	boss	0	Characteristic	0	Peer support	0	Facial pain	0	Grandmother	0	Glutamate	0	Vestibular migraine	0	Upset	0	regime	0	vacation	0	Accident	0	Trauma	0	Neck muscle	0	Population	0	Sensitivity to sound	0
0	Biofeedback	0	Preventive	0	Joy	0	Vascular	0	Butalbital	0	Impact	0	Drive	0	Peer	0	Prophylaxis	0	Angiotensin	0	Toxin	0	Duration	0	Sad	0	minister	0	Hurts	0	Incidence	0	Testing	0	stigma	0	Aggravate	0	Calcium channel blocker	0	faith	0	Adverse effect	0	Confuse	0	Anger	0	Patterns	0	Quality of life	0	Suicide	0	Trigger point	0	Agonist	0	Motion sickness	0	Acute migraine	0	Steroid	0	Anti-inflammatory	0	CAT scan	0	NSAID	0	Shock	0	Epilepsy	0	Headache diary	0	Sinus headache	0	Weight loss	0	Cervical spine	0	boss	0	Characteristic	0	Peer support	0	Facial pain	0	Grandmother	0	Glutamate	0	Vestibular migraine	0	Upset	0	regime	0	vacation	0	Accident	0	Trauma	0	Neck muscle	0	Population	0	Sensitivity to sound	0
9		8		8		8		8		8		8		8		8		7		7		7		7		7		7		7		7		7		6		6		6		6		6		6		6		6		6		6		5		5		5		5		5		5		5		5		5		4		4		4		4		4		4		4		4		4		4		4		4		4		4		4		4		4		4		4

P181a	P181b	P182a	P182b	P183a	P183b	P184a	P184b	P185a	P185b	P186a	P186b	P187a	P187b	P188a	P188b	P189a	P189b	P190a	P190b	P191a	P191b	P192a	P192b	P193a	P193b	P194a	P194b	P195a	P195b	P196a	P196b	P197a	P197b	P198a	P198b	P199a	P199b	P200a	P200b	P201a	P201b	P202a	P202b	P203a	P203b	P204a	P204b	P205a	P205b	P206a	P206b	P207a	P207b	P208a	P208b	P209a	P209b	P210a	P210b	P211a	P211b	P212a	P212b	P213a	P213b	P214a	P214b	P215a	P215b	P216a	P216b	P217a	P217b	P218a	P218b	P219a	P219b	P220a	P220b	P221a	P221b	P222a	P222b	P223a	P223b	P224a	P224b	P225a	P225b	P226a	P226b	P227a	P227b	P228a	P228b	P229a	P229b	P230a	P230b	P231a	P231b	P232a
surgery	0	Medication overuse	2	Mindfulness	1	Tricyclic	0	Histamine	0	Remission	0	Regimen	0	Anti-seizure	0	Occipital nerve	0	Menstrual migraine	0	Type A	0	Lifestyle change	0	Headache clinic	0	impairment	0	Pain management	0	Contraception	0	Peripheral	0	Episodic	0	Cranial nerve	0	Lactation	0	Beta-blocker	0	Efficacy	0	Antihistamine	0	Advantage	0	Progression	0	Productivity	0	Nerve block	0	Accepting	0	Trigeminal autonomic cephalalgia	0	Detoxification	0	Secondary headache	0	spouse	0	Lidocaine	0	Blood flow	0	Double vision	0	Intracranial hypertension	0	Dopamine	0	church	0	Mechanism	0	Complications	0	Attitude	0	Spectrum	0	Consultation	0	Empower	1	Frustrate	0	Complicated migraine	0	5-HT	0	Ineffective	0	Eye symptom	0	Brain lesion	0	Physical examination	0	Referral
surgery	0	Medication overuse	0	Mindfulness	0	Tricyclic	0	Histamine	0	Remission	0	Regimen	0	Anti-seizure	0	Occipital nerve	0	Menstrual migraine	0	Type A	0	Lifestyle change	0	Headache clinic	0	impairment	0	Pain management	0	Contraception	0	Peripheral	0	Episodic	0	Cranial nerve	0	Lactation	0	Beta-blocker	0	Efficacy	0	Antihistamine	0	Advantage	0	Progression	0	Productivity	0	Nerve block	0	Accepting	0	Trigeminal autonomic cephalalgia	0	Detoxification	0	Secondary headache	0	spouse	0	Lidocaine	0	Blood flow	0	Double vision	0	Intracranial hypertension	0	Dopamine	0	church	0	Mechanism	0	Complications	0	Attitude	0	Spectrum	0	Consultation	0	Empower	0	Frustrate	0	Complicated migraine	0	5-HT	0	Ineffective	0	Eye symptom	0	Brain lesion	0	Physical examination	0	Referral
surgery	1	Medication overuse	0	Mindfulness	0	Tricyclic	0	Histamine	0	Remission	0	Regimen	0	Anti-seizure	0	Occipital nerve	0	Menstrual migraine	0	Type A	0	Lifestyle change	0	Headache clinic	0	impairment	0	Pain management	0	Contraception	0	Peripheral	0	Episodic	1	Cranial nerve	0	Lactation	0	Beta-blocker	0	Efficacy	0	Antihistamine	0	Advantage	0	Progression	0	Productivity	0	Nerve block	0	Accepting	0	Trigeminal autonomic cephalalgia	0	Detoxification	0	Secondary headache	0	spouse	0	Lidocaine	0	Blood flow	0	Double vision	0	Intracranial hypertension	0	Dopamine	0	church	0	Mechanism	0	Complications	0	Attitude	0	Spectrum	0	Consultation	0	Empower	0	Frustrate	0	Complicated migraine	0	5-HT	0	Ineffective	0	Eye symptom	0	Brain lesion	0	Physical examination	0	Referral
surgery	0	Medication overuse	0	Mindfulness	0	Tricyclic	0	Histamine	0	Remission	0	Regimen	0	Anti-seizure	0	Occipital nerve	0	Menstrual migraine	0	Type A	0	Lifestyle change	0	Headache clinic	0	impairment	0	Pain management	0	Contraception	0	Peripheral	0	Episodic	0	Cranial nerve	0	Lactation	0	Beta-blocker	0	Efficacy	0	Antihistamine	0	Advantage	0	Progression	0	Productivity	0	Nerve block	0	Accepting	0	Trigeminal autonomic cephalalgia	0	Detoxification	0	Secondary headache	0	spouse	0	Lidocaine	0	Blood flow	0	Double vision	0	Intracranial hypertension	0	Dopamine	0	church	0	Mechanism	0	Complications	0	Attitude	0	Spectrum	0	Consultation	0	Empower	0	Frustrate	0	Complicated migraine	0	5-HT	0	Ineffective	0	Eye symptom	0	Brain lesion	0	Physical examination	0	Referral
surgery	0	Medication overuse	0	Mindfulness	0	Tricyclic	0	Histamine	0	Remission	0	Regimen	0	Anti-seizure	0	Occipital nerve	0	Menstrual migraine	0	Type A	0	Lifestyle change	0	Headache clinic	0	impairment	0	Pain management	0	Contraception	0	Peripheral	0	Episodic	0	Cranial nerve	0	Lactation	0	Beta-blocker	0	Efficacy	0	Antihistamine	0	Advantage	0	Progression	0	Productivity	0	Nerve block	0	Accepting	0	Trigeminal autonomic cephalalgia	0	Detoxification	0	Secondary headache	0	spouse	0	Lidocaine	0	Blood flow	0	Double vision	0	Intracranial hypertension	0	Dopamine	0	church	0	Mechanism	0	Complications	0	Attitude	0	Spectrum	0	Consultation	0	Empower	0	Frustrate	0	Complicated migraine	0	5-HT	0	Ineffective	0	Eye symptom	0	Brain lesion	0	Physical examination	0	Referral
surgery	0	Medication overuse	0	Mindfulness	0	Tricyclic	0	Histamine	0	Remission	0	Regimen	0	Anti-seizure	0	Occipital nerve	0	Menstrual migraine	0	Type A	0	Lifestyle change	0	Headache clinic	0	impairment	0	Pain management	0	Contraception	0	Peripheral	0	Episodic	0	Cranial nerve	0	Lactation	0	Beta-blocker	0	Efficacy	0	Antihistamine	0	Advantage	0	Progression	0	Productivity	0	Nerve block	0	Accepting	0	Trigeminal autonomic cephalalgia	0	Detoxification	0	Secondary headache	0	spouse	0	Lidocaine	0	Blood flow	0	Double vision	0	Intracranial hypertension	0	Dopamine	0	church	0	Mechanism	0	Complications	0	Attitude	0	Spectrum	0	Consultation	0	Empower	0	Frustrate	0	Complicated migraine	0	5-HT	0	Ineffective	0	Eye symptom	0	Brain lesion	0	Physical examination	0	Referral
surgery	0	Medication overuse	0	Mindfulness	0	Tricyclic	0	Histamine	0	Remission	0	Regimen	0	Anti-seizure	0	Occipital nerve	0	Menstrual migraine	0	Type A	0	Lifestyle change	0	Headache clinic	0	impairment	0	Pain management	0	Contraception	0	Peripheral	0	Episodic	0	Cranial nerve	0	Lactation	0	Beta-blocker	0	Efficacy	0	Antihistamine	0	Advantage	0	Progression	0	Productivity	0	Nerve block	0	Accepting	0	Trigeminal autonomic cephalalgia	0	Detoxification	0	Secondary headache	0	spouse	0	Lidocaine	0	Blood flow	0	Double vision	0	Intracranial hypertension	0	Dopamine	0	church	0	Mechanism	0	Complications	0	Attitude	0	Spectrum	0	Consultation	1	Empower	0	Frustrate	0	Complicated migraine	0	5-HT	0	Ineffective	0	Eye symptom	0	Brain lesion	0	Physical examination	0	Referral
surgery	0	Medication overuse	0	Mindfulness	0	Tricyclic	0	Histamine	0	Remission	0	Regimen	0	Anti-seizure	0	Occipital nerve	0	Menstrual migraine	0	Type A	0	Lifestyle change	0	Headache clinic	0	impairment	0	Pain management	0	Contraception	0	Peripheral	0	Episodic	0	Cranial nerve	0	Lactation	0	Beta-blocker	0	Efficacy	0	Antihistamine	0	Advantage	0	Progression	0	Productivity	0	Nerve block	0	Accepting	0	Trigeminal autonomic cephalalgia	0	Detoxification	0	Secondary headache	0	spouse	0	Lidocaine	0	Blood flow	0	Double vision	0	Intracranial hypertension	0	Dopamine	0	church	0	Mechanism	0	Complications	0	Attitude	0	Spectrum	0	Consultation	0	Empower	0	Frustrate	0	Complicated migraine	0	5-HT	0	Ineffective	0	Eye symptom	0	Brain lesion	0	Physical examination	0	Referral
surgery	0	Medication overuse	0	Mindfulness	0	Tricyclic	0	Histamine	0	Remission	0	Regimen	0	Anti-seizure	0	Occipital nerve	0	Menstrual migraine	0	Type A	0	Lifestyle change	0	Headache clinic	0	impairment	0	Pain management	0	Contraception	0	Peripheral	0	Episodic	0	Cranial nerve	0	Lactation	0	Beta-blocker	0	Efficacy	0	Antihistamine	0	Advantage	0	Progression	0	Productivity	0	Nerve block	0	Accepting	0	Trigeminal autonomic cephalalgia	0	Detoxification	0	Secondary headache	0	spouse	0	Lidocaine	0	Blood flow	0	Double vision	0	Intracranial hypertension	0	Dopamine	0	church	0	Mechanism	0	Complications	0	Attitude	0	Spectrum	1	Consultation	0	Empower	0	Frustrate	0	Complicated migraine	0	5-HT	0	Ineffective	0	Eye symptom	0	Brain lesion	0	Physical examination	0	Referral
surgery	0	Medication overuse	0	Mindfulness	0	Tricyclic	0	Histamine	0	Remission	0	Regimen	0	Anti-seizure	0	Occipital nerve	0	Menstrual migraine	0	Type A	0	Lifestyle change	0	Headache clinic	0	impairment	0	Pain management	0	Contraception	0	Peripheral	0	Episodic	0	Cranial nerve	0	Lactation	0	Beta-blocker	0	Efficacy	0	Antihistamine	0	Advantage	0	Progression	0	Productivity	0	Nerve block	0	Accepting	0	Trigeminal autonomic cephalalgia	0	Detoxification	0	Secondary headache	0	spouse	0	Lidocaine	0	Blood flow	0	Double vision	0	Intracranial hypertension	0	Dopamine	0	church	0	Mechanism	0	Complications	0	Attitude	1	Spectrum	0	Consultation	0	Empower	1	Frustrate	0	Complicated migraine	0	5-HT	0	Ineffective	0	Eye symptom	0	Brain lesion	0	Physical examination	0	Referral
surgery	0	Medication overuse	0	Mindfulness	0	Tricyclic	0	Histamine	0	Remission	0	Regimen	0	Anti-seizure	0	Occipital nerve	0	Menstrual migraine	0	Type A	0	Lifestyle change	0	Headache clinic	0	impairment	0	Pain management	0	Contraception	0	Peripheral	1	Episodic	0	Cranial nerve	0	Lactation	0	Beta-blocker	0	Efficacy	0	Antihistamine	0	Advantage	0	Progression	0	Productivity	0	Nerve block	0	Accepting	0	Trigeminal autonomic cephalalgia	0	Detoxification	0	Secondary headache	0	spouse	0	Lidocaine	0	Blood flow	0	Double vision	0	Intracranial hypertension	0	Dopamine	0	church	0	Mechanism	0	Complications	0	Attitude	0	Spectrum	0	Consultation	0	Empower	0	Frustrate	0	Complicated migraine	0	5-HT	0	Ineffective	0	Eye symptom	0	Brain lesion	0	Physical examination	0	Referral
surgery	0	Medication overuse	0	Mindfulness	0	Tricyclic	0	Histamine	0	Remission	0	Regimen	0	Anti-seizure	0	Occipital nerve	0	Menstrual migraine	0	Type A	0	Lifestyle change	0	Headache clinic	0	impairment	0	Pain management	0	Contraception	0	Peripheral	0	Episodic	0	Cranial nerve	0	Lactation	0	Beta-blocker	0	Efficacy	0	Antihistamine	0	Advantage	0	Progression	0	Productivity	0	Nerve block	0	Accepting	0	Trigeminal autonomic cephalalgia	0	Detoxification	0	Secondary headache	0	spouse	0	Lidocaine	0	Blood flow	0	Double vision	0	Intracranial hypertension	0	Dopamine	0	church	0	Mechanism	0	Complications	0	Attitude	0	Spectrum	0	Consultation	0	Empower	0	Frustrate	0	Complicated migraine	0	5-HT	0	Ineffective	0	Eye symptom	0	Brain lesion	0	Physical examination	0	Referral
surgery	0	Medication overuse	0	Mindfulness	0	Tricyclic	0	Histamine	0	Remission	0	Regimen	0	Anti-seizure	0	Occipital nerve	0	Menstrual migraine	0	Type A	0	Lifestyle change	0	Headache clinic	0	impairment	0	Pain management	0	Contraception	0	Peripheral	0	Episodic	1	Cranial nerve	0	Lactation	0	Beta-blocker	0	Efficacy	0	Antihistamine	0	Advantage	0	Progression	0	Productivity	0	Nerve block	0	Accepting	0	Trigeminal autonomic cephalalgia	0	Detoxification	0	Secondary headache	0	spouse	0	Lidocaine	0	Blood flow	0	Double vision	0	Intracranial hypertension	0	Dopamine	0	church	0	Mechanism	0	Complications	0	Attitude	0	Spectrum	0	Consultation	0	Empower	0	Frustrate	0	Complicated migraine	0	5-HT	0	Ineffective	0	Eye symptom	0	Brain lesion	0	Physical examination	0	Referral
surgery	0	Medication overuse	0	Mindfulness	0	Tricyclic	0	Histamine	0	Remission	0	Regimen	0	Anti-seizure	0	Occipital nerve	0	Menstrual migraine	0	Type A	0	Lifestyle change	0	Headache clinic	0	impairment	0	Pain management	0	Contraception	3	Peripheral	0	Episodic	0	Cranial nerve	0	Lactation	0	Beta-blocker	0	Efficacy	0	Antihistamine	0	Advantage	0	Progression	0	Productivity	0	Nerve block	0	Accepting	0	Trigeminal autonomic cephalalgia	0	Detoxification	0	Secondary headache	0	spouse	0	Lidocaine	0	Blood flow	0	Double vision	0	Intracranial hypertension	0	Dopamine	0	church	0	Mechanism	0	Complications	0	Attitude	0	Spectrum	0	Consultation	0	Empower	0	Frustrate	0	Complicated migraine	0	5-HT	0	Ineffective	0	Eye symptom	0	Brain lesion	0	Physical examination	0	Referral
surgery	0	Medication overuse	0	Mindfulness	0	Tricyclic	0	Histamine	0	Remission	0	Regimen	0	Anti-seizure	0	Occipital nerve	0	Menstrual migraine	0	Type A	0	Lifestyle change	0	Headache clinic	0	impairment	0	Pain management	1	Contraception	0	Peripheral	0	Episodic	0	Cranial nerve	0	Lactation	0	Beta-blocker	0	Efficacy	0	Antihistamine	0	Advantage	0	Progression	0	Productivity	0	Nerve block	0	Accepting	0	Trigeminal autonomic cephalalgia	0	Detoxification	0	Secondary headache	0	spouse	0	Lidocaine	0	Blood flow	0	Double vision	0	Intracranial hypertension	0	Dopamine	0	church	0	Mechanism	0	Complications	1	Attitude	0	Spectrum	0	Consultation	0	Empower	0	Frustrate	0	Complicated migraine	0	5-HT	0	Ineffective	0	Eye symptom	0	Brain lesion	0	Physical examination	0	Referral
surgery	0	Medication overuse	0	Mindfulness	0	Tricyclic	0	Histamine	0	Remission	0	Regimen	0	Anti-seizure	0	Occipital nerve	0	Menstrual migraine	0	Type A	0	Lifestyle change	0	Headache clinic	0	impairment	0	Pain management	0	Contraception	0	Peripheral	0	Episodic	0	Cranial nerve	0	Lactation	0	Beta-blocker	0	Efficacy	0	Antihistamine	0	Advantage	0	Progression	0	Productivity	0	Nerve block	0	Accepting	0	Trigeminal autonomic cephalalgia	0	Detoxification	0	Secondary headache	0	spouse	0	Lidocaine	0	Blood flow	0	Double vision	0	Intracranial hypertension	0	Dopamine	0	church	0	Mechanism	0	Complications	0	Attitude	0	Spectrum	0	Consultation	0	Empower	0	Frustrate	0	Complicated migraine	0	5-HT	0	Ineffective	0	Eye symptom	0	Brain lesion	0	Physical examination	0	Referral
surgery	0	Medication overuse	0	Mindfulness	0	Tricyclic	0	Histamine	0	Remission	0	Regimen	0	Anti-seizure	0	Occipital nerve	0	Menstrual migraine	0	Type A	0	Lifestyle change	0	Headache clinic	0	impairment	0	Pain management	0	Contraception	0	Peripheral	0	Episodic	0	Cranial nerve	0	Lactation	0	Beta-blocker	0	Efficacy	0	Antihistamine	0	Advantage	0	Progression	0	Productivity	0	Nerve block	0	Accepting	0	Trigeminal autonomic cephalalgia	0	Detoxification	0	Secondary headache	0	spouse	0	Lidocaine	0	Blood flow	0	Double vision	0	Intracranial hypertension	0	Dopamine	0	church	0	Mechanism	0	Complications	0	Attitude	0	Spectrum	0	Consultation	0	Empower	0	Frustrate	0	Complicated migraine	0	5-HT	0	Ineffective	0	Eye symptom	0	Brain lesion	0	Physical examination	0	Referral
surgery	0	Medication overuse	0	Mindfulness	0	Tricyclic	0	Histamine	0	Remission	0	Regimen	0	Anti-seizure	0	Occipital nerve	0	Menstrual migraine	0	Type A	0	Lifestyle change	0	Headache clinic	0	impairment	3	Pain management	0	Contraception	0	Peripheral	0	Episodic	0	Cranial nerve	0	Lactation	0	Beta-blocker	0	Efficacy	0	Antihistamine	0	Advantage	0	Progression	0	Productivity	0	Nerve block	0	Accepting	0	Trigeminal autonomic cephalalgia	0	Detoxification	0	Secondary headache	0	spouse	0	Lidocaine	0	Blood flow	0	Double vision	0	Intracranial hypertension	0	Dopamine	0	church	0	Mechanism	1	Complications	0	Attitude	0	Spectrum	0	Consultation	0	Empower	0	Frustrate	0	Complicated migraine	0	5-HT	0	Ineffective	0	Eye symptom	0	Brain lesion	0	Physical examination	0	Referral
surgery	0	Medication overuse	0	Mindfulness	0	Tricyclic	0	Histamine	0	Remission	0	Regimen	0	Anti-seizure	0	Occipital nerve	0	Menstrual migraine	0	Type A	0	Lifestyle change	0	Headache clinic	0	impairment	0	Pain management	0	Contraception	0	Peripheral	0	Episodic	0	Cranial nerve	0	Lactation	0	Beta-blocker	0	Efficacy	0	Antihistamine	0	Advantage	0	Progression	0	Productivity	0	Nerve block	0	Accepting	0	Trigeminal autonomic cephalalgia	0	Detoxification	0	Secondary headache	0	spouse	0	Lidocaine	0	Blood flow	0	Double vision	0	Intracranial hypertension	0	Dopamine	0	church	0	Mechanism	0	Complications	0	Attitude	0	Spectrum	0	Consultation	0	Empower	0	Frustrate	0	Complicated migraine	0	5-HT	0	Ineffective	0	Eye symptom	0	Brain lesion	0	Physical examination	0	Referral
surgery	0	Medication overuse	0	Mindfulness	0	Tricyclic	0	Histamine	0	Remission	0	Regimen	0	Anti-seizure	0	Occipital nerve	0	Menstrual migraine	0	Type A	0	Lifestyle change	0	Headache clinic	1	impairment	0	Pain management	0	Contraception	0	Peripheral	0	Episodic	0	Cranial nerve	0	Lactation	0	Beta-blocker	0	Efficacy	0	Antihistamine	0	Advantage	0	Progression	0	Productivity	0	Nerve block	0	Accepting	0	Trigeminal autonomic cephalalgia	0	Detoxification	0	Secondary headache	0	spouse	0	Lidocaine	0	Blood flow	0	Double vision	0	Intracranial hypertension	0	Dopamine	0	church	0	Mechanism	0	Complications	0	Attitude	0	Spectrum	0	Consultation	0	Empower	0	Frustrate	0	Complicated migraine	0	5-HT	0	Ineffective	0	Eye symptom	0	Brain lesion	0	Physical examination	0	Referral
surgery	0	Medication overuse	0	Mindfulness	0	Tricyclic	0	Histamine	0	Remission	0	Regimen	0	Anti-seizure	0	Occipital nerve	0	Menstrual migraine	0	Type A	0	Lifestyle change	0	Headache clinic	0	impairment	0	Pain management	0	Contraception	0	Peripheral	0	Episodic	0	Cranial nerve	0	Lactation	0	Beta-blocker	0	Efficacy	0	Antihistamine	0	Advantage	0	Progression	0	Productivity	0	Nerve block	0	Accepting	0	Trigeminal autonomic cephalalgia	0	Detoxification	0	Secondary headache	0	spouse	0	Lidocaine	0	Blood flow	0	Double vision	1	Intracranial hypertension	1	Dopamine	1	church	1	Mechanism	0	Complications	0	Attitude	0	Spectrum	0	Consultation	0	Empower	0	Frustrate	0	Complicated migraine	0	5-HT	0	Ineffective	0	Eye symptom	0	Brain lesion	0	Physical examination	0	Referral
surgery	0	Medication overuse	2	Mindfulness	0	Tricyclic	0	Histamine	0	Remission	0	Regimen	0	Anti-seizure	0	Occipital nerve	0	Menstrual migraine	0	Type A	0	Lifestyle change	1	Headache clinic	0	impairment	0	Pain management	0	Contraception	0	Peripheral	0	Episodic	0	Cranial nerve	0	Lactation	0	Beta-blocker	0	Efficacy	0	Antihistamine	0	Advantage	0	Progression	0	Productivity	0	Nerve block	0	Accepting	0	Trigeminal autonomic cephalalgia	0	Detoxification	0	Secondary headache	0	spouse	0	Lidocaine	0	Blood flow	0	Double vision	0	Intracranial hypertension	0	Dopamine	0	church	0	Mechanism	0	Complications	0	Attitude	0	Spectrum	0	Consultation	0	Empower	0	Frustrate	0	Complicated migraine	0	5-HT	0	Ineffective	0	Eye symptom	0	Brain lesion	0	Physical examination	0	Referral
surgery	0	Medication overuse	0	Mindfulness	0	Tricyclic	0	Histamine	0	Remission	0	Regimen	0	Anti-seizure	0	Occipital nerve	0	Menstrual migraine	0	Type A	0	Lifestyle change	0	Headache clinic	0	impairment	0	Pain management	0	Contraception	0	Peripheral	2	Episodic	0	Cranial nerve	0	Lactation	0	Beta-blocker	0	Efficacy	0	Antihistamine	0	Advantage	0	Progression	0	Productivity	0	Nerve block	0	Accepting	0	Trigeminal autonomic cephalalgia	0	Detoxification	0	Secondary headache	0	spouse	0	Lidocaine	0	Blood flow	1	Double vision	0	Intracranial hypertension	0	Dopamine	0	church	0	Mechanism	0	Complications	0	Attitude	0	Spectrum	0	Consultation	0	Empower	0	Frustrate	0	Complicated migraine	0	5-HT	0	Ineffective	0	Eye symptom	0	Brain lesion	0	Physical examination	0	Referral
surgery	0	Medication overuse	0	Mindfulness	0	Tricyclic	0	Histamine	0	Remission	0	Regimen	0	Anti-seizure	0	Occipital nerve	0	Menstrual migraine	0	Type A	0	Lifestyle change	0	Headache clinic	0	impairment	0	Pain management	0	Contraception	0	Peripheral	0	Episodic	0	Cranial nerve	0	Lactation	0	Beta-blocker	0	Efficacy	0	Antihistamine	0	Advantage	0	Progression	0	Productivity	0	Nerve block	0	Accepting	0	Trigeminal autonomic cephalalgia	0	Detoxification	0	Secondary headache	0	spouse	0	Lidocaine	0	Blood flow	0	Double vision	0	Intracranial hypertension	0	Dopamine	0	church	0	Mechanism	0	Complications	0	Attitude	0	Spectrum	0	Consultation	0	Empower	0	Frustrate	0	Complicated migraine	0	5-HT	0	Ineffective	0	Eye symptom	0	Brain lesion	0	Physical examination	0	Referral
surgery	0	Medication overuse	0	Mindfulness	0	Tricyclic	0	Histamine	0	Remission	0	Regimen	0	Anti-seizure	0	Occipital nerve	0	Menstrual migraine	0	Type A	1	Lifestyle change	0	Headache clinic	0	impairment	0	Pain management	0	Contraception	0	Peripheral	0	Episodic	0	Cranial nerve	0	Lactation	0	Beta-blocker	0	Efficacy	0	Antihistamine	0	Advantage	0	Progression	0	Productivity	0	Nerve block	0	Accepting	0	Trigeminal autonomic cephalalgia	0	Detoxification	0	Secondary headache	0	spouse	0	Lidocaine	1	Blood flow	0	Double vision	0	Intracranial hypertension	0	Dopamine	0	church	0	Mechanism	0	Complications	0	Attitude	0	Spectrum	0	Consultation	0	Empower	0	Frustrate	0	Complicated migraine	0	5-HT	0	Ineffective	0	Eye symptom	0	Brain lesion	0	Physical examination	0	Referral
surgery	0	Medication overuse	0	Mindfulness	0	Tricyclic	0	Histamine	0	Remission	0	Regimen	0	Anti-seizure	0	Occipital nerve	0	Menstrual migraine	0	Type A	0	Lifestyle change	0	Headache clinic	0	impairment	0	Pain management	0	Contraception	0	Peripheral	0	Episodic	0	Cranial nerve	0	Lactation	0	Beta-blocker	0	Efficacy	0	Antihistamine	0	Advantage	0	Progression	0	Productivity	0	Nerve block	0	Accepting	0	Trigeminal autonomic cephalalgia	0	Detoxification	0	Secondary headache	0	spouse	0	Lidocaine	0	Blood flow	0	Double vision	0	Intracranial hypertension	0	Dopamine	0	church	0	Mechanism	0	Complications	0	Attitude	0	Spectrum	0	Consultation	0	Empower	0	Frustrate	0	Complicated migraine	0	5-HT	0	Ineffective	0	Eye symptom	0	Brain lesion	0	Physical examination	0	Referral
surgery	0	Medication overuse	0	Mindfulness	0	Tricyclic	0	Histamine	0	Remission	0	Regimen	0	Anti-seizure	0	Occipital nerve	0	Menstrual migraine	0	Type A	0	Lifestyle change	0	Headache clinic	0	impairment	0	Pain management	0	Contraception	0	Peripheral	0	Episodic	0	Cranial nerve	0	Lactation	0	Beta-blocker	0	Efficacy	0	Antihistamine	0	Advantage	0	Progression	0	Productivity	0	Nerve block	0	Accepting	0	Trigeminal autonomic cephalalgia	0	Detoxification	0	Secondary headache	0	spouse	0	Lidocaine	0	Blood flow	0	Double vision	0	Intracranial hypertension	0	Dopamine	0	church	0	Mechanism	0	Complications	0	Attitude	0	Spectrum	0	Consultation	0	Empower	0	Frustrate	0	Complicated migraine	0	5-HT	0	Ineffective	0	Eye symptom	0	Brain lesion	0	Physical examination	0	Referral
surgery	0	Medication overuse	0	Mindfulness	0	Tricyclic	0	Histamine	0	Remission	0	Regimen	0	Anti-seizure	0	Occipital nerve	0	Menstrual migraine	0	Type A	0	Lifestyle change	0	Headache clinic	0	impairment	0	Pain management	0	Contraception	0	Peripheral	0	Episodic	0	Cranial nerve	0	Lactation	0	Beta-blocker	0	Efficacy	0	Antihistamine	0	Advantage	0	Progression	0	Productivity	0	Nerve block	0	Accepting	0	Trigeminal autonomic cephalalgia	0	Detoxification	0	Secondary headache	0	spouse	1	Lidocaine	0	Blood flow	0	Double vision	0	Intracranial hypertension	0	Dopamine	0	church	0	Mechanism	0	Complications	0	Attitude	1	Spectrum	0	Consultation	0	Empower	0	Frustrate	0	Complicated migraine	0	5-HT	0	Ineffective	0	Eye symptom	0	Brain lesion	0	Physical examination	0	Referral
surgery	0	Medication overuse	0	Mindfulness	0	Tricyclic	0	Histamine	0	Remission	0	Regimen	0	Anti-seizure	0	Occipital nerve	0	Menstrual migraine	0	Type A	0	Lifestyle change	0	Headache clinic	0	impairment	0	Pain management	0	Contraception	0	Peripheral	0	Episodic	0	Cranial nerve	0	Lactation	0	Beta-blocker	0	Efficacy	0	Antihistamine	0	Advantage	0	Progression	0	Productivity	0	Nerve block	0	Accepting	0	Trigeminal autonomic cephalalgia	0	Detoxification	0	Secondary headache	0	spouse	0	Lidocaine	0	Blood flow	0	Double vision	0	Intracranial hypertension	0	Dopamine	0	church	0	Mechanism	0	Complications	0	Attitude	0	Spectrum	0	Consultation	0	Empower	0	Frustrate	0	Complicated migraine	0	5-HT	0	Ineffective	0	Eye symptom	0	Brain lesion	0	Physical examination	0	Referral
surgery	1	Medication overuse	0	Mindfulness	0	Tricyclic	0	Histamine	0	Remission	0	Regimen	0	Anti-seizure	0	Occipital nerve	0	Menstrual migraine	0	Type A	0	Lifestyle change	0	Headache clinic	0	impairment	0	Pain management	0	Contraception	0	Peripheral	0	Episodic	0	Cranial nerve	0	Lactation	0	Beta-blocker	0	Efficacy	0	Antihistamine	0	Advantage	0	Progression	0	Productivity	0	Nerve block	0	Accepting	0	Trigeminal autonomic cephalalgia	2	Detoxification	1	Secondary headache	2	spouse	0	Lidocaine	0	Blood flow	0	Double vision	0	Intracranial hypertension	0	Dopamine	0	church	0	Mechanism	0	Complications	0	Attitude	0	Spectrum	0	Consultation	0	Empower	0	Frustrate	0	Complicated migraine	0	5-HT	0	Ineffective	0	Eye symptom	0	Brain lesion	0	Physical examination	0	Referral
surgery	0	Medication overuse	0	Mindfulness	0	Tricyclic	0	Histamine	0	Remission	0	Regimen	0	Anti-seizure	0	Occipital nerve	0	Menstrual migraine	0	Type A	0	Lifestyle change	0	Headache clinic	0	impairment	0	Pain management	0	Contraception	0	Peripheral	0	Episodic	0	Cranial nerve	0	Lactation	0	Beta-blocker	0	Efficacy	0	Antihistamine	0	Advantage	0	Progression	0	Productivity	0	Nerve block	0	Accepting	0	Trigeminal autonomic cephalalgia	0	Detoxification	0	Secondary headache	0	spouse	0	Lidocaine	0	Blood flow	0	Double vision	0	Intracranial hypertension	0	Dopamine	0	church	0	Mechanism	0	Complications	0	Attitude	0	Spectrum	0	Consultation	0	Empower	0	Frustrate	0	Complicated migraine	0	5-HT	0	Ineffective	0	Eye symptom	0	Brain lesion	0	Physical examination	0	Referral
surgery	0	Medication overuse	0	Mindfulness	0	Tricyclic	0	Histamine	0	Remission	0	Regimen	0	Anti-seizure	0	Occipital nerve	0	Menstrual migraine	0	Type A	0	Lifestyle change	0	Headache clinic	0	impairment	0	Pain management	0	Contraception	0	Peripheral	0	Episodic	0	Cranial nerve	0	Lactation	0	Beta-blocker	0	Efficacy	0	Antihistamine	0	Advantage	0	Progression	0	Productivity	0	Nerve block	0	Accepting	0	Trigeminal autonomic cephalalgia	0	Detoxification	0	Secondary headache	0	spouse	0	Lidocaine	0	Blood flow	0	Double vision	0	Intracranial hypertension	0	Dopamine	0	church	0	Mechanism	0	Complications	0	Attitude	0	Spectrum	0	Consultation	0	Empower	0	Frustrate	0	Complicated migraine	0	5-HT	0	Ineffective	0	Eye symptom	0	Brain lesion	0	Physical examination	0	Referral
surgery	0	Medication overuse	0	Mindfulness	0	Tricyclic	0	Histamine	0	Remission	0	Regimen	0	Anti-seizure	0	Occipital nerve	0	Menstrual migraine	0	Type A	0	Lifestyle change	0	Headache clinic	0	impairment	0	Pain management	0	Contraception	0	Peripheral	0	Episodic	0	Cranial nerve	0	Lactation	0	Beta-blocker	0	Efficacy	0	Antihistamine	0	Advantage	0	Progression	0	Productivity	0	Nerve block	0	Accepting	0	Trigeminal autonomic cephalalgia	0	Detoxification	0	Secondary headache	0	spouse	0	Lidocaine	0	Blood flow	0	Double vision	0	Intracranial hypertension	0	Dopamine	0	church	0	Mechanism	0	Complications	0	Attitude	0	Spectrum	0	Consultation	0	Empower	0	Frustrate	0	Complicated migraine	0	5-HT	0	Ineffective	0	Eye symptom	0	Brain lesion	0	Physical examination	0	Referral
surgery	0	Medication overuse	0	Mindfulness	0	Tricyclic	0	Histamine	0	Remission	0	Regimen	0	Anti-seizure	0	Occipital nerve	0	Menstrual migraine	1	Type A	0	Lifestyle change	0	Headache clinic	0	impairment	0	Pain management	0	Contraception	0	Peripheral	0	Episodic	0	Cranial nerve	0	Lactation	0	Beta-blocker	0	Efficacy	0	Antihistamine	0	Advantage	0	Progression	0	Productivity	0	Nerve block	0	Accepting	0	Trigeminal autonomic cephalalgia	0	Detoxification	0	Secondary headache	0	spouse	0	Lidocaine	0	Blood flow	0	Double vision	0	Intracranial hypertension	0	Dopamine	0	church	0	Mechanism	0	Complications	0	Attitude	0	Spectrum	0	Consultation	0	Empower	0	Frustrate	0	Complicated migraine	0	5-HT	0	Ineffective	0	Eye symptom	0	Brain lesion	0	Physical examination	0	Referral
surgery	1	Medication overuse	0	Mindfulness	0	Tricyclic	0	Histamine	0	Remission	0	Regimen	1	Anti-seizure	2	Occipital nerve	3	Menstrual migraine	1	Type A	0	Lifestyle change	2	Headache clinic	0	impairment	0	Pain management	1	Contraception	0	Peripheral	0	Episodic	0	Cranial nerve	0	Lactation	0	Beta-blocker	0	Efficacy	0	Antihistamine	0	Advantage	0	Progression	0	Productivity	0	Nerve block	2	Accepting	2	Trigeminal autonomic cephalalgia	0	Detoxification	0	Secondary headache	0	spouse	1	Lidocaine	1	Blood flow	0	Double vision	0	Intracranial hypertension	0	Dopamine	0	church	0	Mechanism	1	Complications	0	Attitude	0	Spectrum	1	Consultation	0	Empower	0	Frustrate	0	Complicated migraine	0	5-HT	0	Ineffective	0	Eye symptom	0	Brain lesion	0	Physical examination	0	Referral
surgery	0	Medication overuse	0	Mindfulness	0	Tricyclic	0	Histamine	0	Remission	0	Regimen	0	Anti-seizure	0	Occipital nerve	0	Menstrual migraine	0	Type A	0	Lifestyle change	0	Headache clinic	0	impairment	0	Pain management	0	Contraception	0	Peripheral	0	Episodic	0	Cranial nerve	0	Lactation	0	Beta-blocker	0	Efficacy	0	Antihistamine	0	Advantage	0	Progression	0	Productivity	0	Nerve block	0	Accepting	0	Trigeminal autonomic cephalalgia	0	Detoxification	0	Secondary headache	0	spouse	0	Lidocaine	0	Blood flow	0	Double vision	0	Intracranial hypertension	0	Dopamine	0	church	0	Mechanism	0	Complications	0	Attitude	0	Spectrum	0	Consultation	0	Empower	0	Frustrate	0	Complicated migraine	0	5-HT	0	Ineffective	0	Eye symptom	0	Brain lesion	0	Physical examination	0	Referral
surgery	0	Medication overuse	0	Mindfulness	0	Tricyclic	0	Histamine	0	Remission	0	Regimen	0	Anti-seizure	0	Occipital nerve	0	Menstrual migraine	0	Type A	0	Lifestyle change	0	Headache clinic	0	impairment	0	Pain management	0	Contraception	0	Peripheral	0	Episodic	0	Cranial nerve	0	Lactation	0	Beta-blocker	0	Efficacy	0	Antihistamine	0	Advantage	0	Progression	0	Productivity	1	Nerve block	0	Accepting	0	Trigeminal autonomic cephalalgia	0	Detoxification	0	Secondary headache	0	spouse	0	Lidocaine	0	Blood flow	1	Double vision	0	Intracranial hypertension	0	Dopamine	0	church	0	Mechanism	0	Complications	0	Attitude	0	Spectrum	0	Consultation	0	Empower	0	Frustrate	0	Complicated migraine	0	5-HT	0	Ineffective	0	Eye symptom	0	Brain lesion	0	Physical examination	0	Referral
surgery	0	Medication overuse	0	Mindfulness	0	Tricyclic	0	Histamine	0	Remission	0	Regimen	0	Anti-seizure	0	Occipital nerve	0	Menstrual migraine	0	Type A	0	Lifestyle change	0	Headache clinic	0	impairment	0	Pain management	0	Contraception	0	Peripheral	0	Episodic	0	Cranial nerve	0	Lactation	0	Beta-blocker	0	Efficacy	0	Antihistamine	0	Advantage	0	Progression	1	Productivity	0	Nerve block	0	Accepting	0	Trigeminal autonomic cephalalgia	0	Detoxification	1	Secondary headache	0	spouse	0	Lidocaine	0	Blood flow	0	Double vision	0	Intracranial hypertension	0	Dopamine	0	church	0	Mechanism	0	Complications	0	Attitude	0	Spectrum	0	Consultation	0	Empower	0	Frustrate	0	Complicated migraine	0	5-HT	0	Ineffective	0	Eye symptom	0	Brain lesion	0	Physical examination	0	Referral
surgery	0	Medication overuse	0	Mindfulness	0	Tricyclic	2	Histamine	2	Remission	2	Regimen	0	Anti-seizure	1	Occipital nerve	0	Menstrual migraine	0	Type A	0	Lifestyle change	0	Headache clinic	2	impairment	0	Pain management	0	Contraception	0	Peripheral	0	Episodic	0	Cranial nerve	0	Lactation	0	Beta-blocker	0	Efficacy	2	Antihistamine	1	Advantage	2	Progression	1	Productivity	0	Nerve block	0	Accepting	0	Trigeminal autonomic cephalalgia	0	Detoxification	0	Secondary headache	0	spouse	0	Lidocaine	0	Blood flow	0	Double vision	0	Intracranial hypertension	0	Dopamine	1	church	0	Mechanism	0	Complications	1	Attitude	0	Spectrum	0	Consultation	0	Empower	0	Frustrate	0	Complicated migraine	0	5-HT	0	Ineffective	0	Eye symptom	0	Brain lesion	0	Physical examination	0	Referral
surgery	0	Medication overuse	0	Mindfulness	0	Tricyclic	0	Histamine	0	Remission	0	Regimen	0	Anti-seizure	0	Occipital nerve	0	Menstrual migraine	0	Type A	0	Lifestyle change	0	Headache clinic	0	impairment	0	Pain management	0	Contraception	0	Peripheral	0	Episodic	0	Cranial nerve	0	Lactation	0	Beta-blocker	1	Efficacy	0	Antihistamine	0	Advantage	0	Progression	0	Productivity	0	Nerve block	0	Accepting	0	Trigeminal autonomic cephalalgia	0	Detoxification	0	Secondary headache	0	spouse	0	Lidocaine	0	Blood flow	0	Double vision	1	Intracranial hypertension	0	Dopamine	0	church	0	Mechanism	0	Complications	0	Attitude	0	Spectrum	0	Consultation	0	Empower	0	Frustrate	0	Complicated migraine	0	5-HT	0	Ineffective	0	Eye symptom	0	Brain lesion	0	Physical examination	0	Referral
surgery	0	Medication overuse	0	Mindfulness	0	Tricyclic	0	Histamine	0	Remission	0	Regimen	0	Anti-seizure	0	Occipital nerve	0	Menstrual migraine	0	Type A	0	Lifestyle change	0	Headache clinic	0	impairment	0	Pain management	0	Contraception	0	Peripheral	0	Episodic	0	Cranial nerve	0	Lactation	0	Beta-blocker	0	Efficacy	0	Antihistamine	0	Advantage	0	Progression	0	Productivity	0	Nerve block	0	Accepting	0	Trigeminal autonomic cephalalgia	0	Detoxification	0	Secondary headache	0	spouse	0	Lidocaine	0	Blood flow	0	Double vision	0	Intracranial hypertension	0	Dopamine	0	church	0	Mechanism	0	Complications	0	Attitude	0	Spectrum	0	Consultation	0	Empower	0	Frustrate	0	Complicated migraine	0	5-HT	0	Ineffective	0	Eye symptom	0	Brain lesion	0	Physical examination	0	Referral
surgery	0	Medication overuse	0	Mindfulness	0	Tricyclic	0	Histamine	1	Remission	0	Regimen	0	Anti-seizure	0	Occipital nerve	0	Menstrual migraine	0	Type A	0	Lifestyle change	0	Headache clinic	0	impairment	0	Pain management	0	Contraception	0	Peripheral	0	Episodic	0	Cranial nerve	0	Lactation	0	Beta-blocker	0	Efficacy	0	Antihistamine	1	Advantage	0	Progression	0	Productivity	0	Nerve block	0	Accepting	0	Trigeminal autonomic cephalalgia	0	Detoxification	0	Secondary headache	0	spouse	0	Lidocaine	0	Blood flow	0	Double vision	0	Intracranial hypertension	0	Dopamine	0	church	0	Mechanism	0	Complications	0	Attitude	0	Spectrum	0	Consultation	0	Empower	0	Frustrate	0	Complicated migraine	0	5-HT	0	Ineffective	0	Eye symptom	0	Brain lesion	0	Physical examination	0	Referral
surgery	0	Medication overuse	0	Mindfulness	0	Tricyclic	0	Histamine	0	Remission	0	Regimen	0	Anti-seizure	0	Occipital nerve	0	Menstrual migraine	0	Type A	0	Lifestyle change	0	Headache clinic	0	impairment	0	Pain management	0	Contraception	0	Peripheral	0	Episodic	0	Cranial nerve	0	Lactation	0	Beta-blocker	0	Efficacy	0	Antihistamine	0	Advantage	0	Progression	0	Productivity	0	Nerve block	0	Accepting	0	Trigeminal autonomic cephalalgia	0	Detoxification	0	Secondary headache	0	spouse	0	Lidocaine	0	Blood flow	0	Double vision	0	Intracranial hypertension	0	Dopamine	0	church	0	Mechanism	0	Complications	0	Attitude	0	Spectrum	0	Consultation	0	Empower	0	Frustrate	0	Complicated migraine	0	5-HT	0	Ineffective	0	Eye symptom	0	Brain lesion	0	Physical examination	0	Referral
surgery	0	Medication overuse	0	Mindfulness	0	Tricyclic	0	Histamine	0	Remission	0	Regimen	0	Anti-seizure	0	Occipital nerve	0	Menstrual migraine	0	Type A	0	Lifestyle change	0	Headache clinic	0	impairment	0	Pain management	0	Contraception	0	Peripheral	0	Episodic	0	Cranial nerve	0	Lactation	0	Beta-blocker	0	Efficacy	0	Antihistamine	0	Advantage	0	Progression	0	Productivity	0	Nerve block	0	Accepting	0	Trigeminal autonomic cephalalgia	0	Detoxification	0	Secondary headache	0	spouse	0	Lidocaine	0	Blood flow	0	Double vision	0	Intracranial hypertension	1	Dopamine	0	church	0	Mechanism	0	Complications	0	Attitude	0	Spectrum	0	Consultation	0	Empower	0	Frustrate	0	Complicated migraine	0	5-HT	0	Ineffective	0	Eye symptom	0	Brain lesion	0	Physical examination	0	Referral
surgery	0	Medication overuse	0	Mindfulness	0	Tricyclic	0	Histamine	0	Remission	0	Regimen	0	Anti-seizure	0	Occipital nerve	0	Menstrual migraine	0	Type A	0	Lifestyle change	0	Headache clinic	0	impairment	0	Pain management	0	Contraception	0	Peripheral	0	Episodic	0	Cranial nerve	0	Lactation	0	Beta-blocker	0	Efficacy	0	Antihistamine	0	Advantage	0	Progression	0	Productivity	0	Nerve block	0	Accepting	0	Trigeminal autonomic cephalalgia	0	Detoxification	0	Secondary headache	0	spouse	0	Lidocaine	0	Blood flow	0	Double vision	0	Intracranial hypertension	0	Dopamine	0	church	0	Mechanism	0	Complications	0	Attitude	0	Spectrum	0	Consultation	0	Empower	0	Frustrate	0	Complicated migraine	0	5-HT	0	Ineffective	0	Eye symptom	0	Brain lesion	0	Physical examination	0	Referral
surgery	0	Medication overuse	0	Mindfulness	0	Tricyclic	0	Histamine	0	Remission	0	Regimen	0	Anti-seizure	0	Occipital nerve	0	Menstrual migraine	0	Type A	0	Lifestyle change	0	Headache clinic	0	impairment	0	Pain management	1	Contraception	0	Peripheral	0	Episodic	0	Cranial nerve	0	Lactation	0	Beta-blocker	0	Efficacy	0	Antihistamine	0	Advantage	0	Progression	0	Productivity	0	Nerve block	0	Accepting	0	Trigeminal autonomic cephalalgia	0	Detoxification	0	Secondary headache	0	spouse	0	Lidocaine	0	Blood flow	0	Double vision	0	Intracranial hypertension	0	Dopamine	0	church	0	Mechanism	0	Complications	0	Attitude	0	Spectrum	0	Consultation	0	Empower	0	Frustrate	0	Complicated migraine	0	5-HT	0	Ineffective	0	Eye symptom	0	Brain lesion	0	Physical examination	0	Referral
surgery	0	Medication overuse	0	Mindfulness	0	Tricyclic	1	Histamine	0	Remission	0	Regimen	0	Anti-seizure	0	Occipital nerve	0	Menstrual migraine	1	Type A	0	Lifestyle change	0	Headache clinic	0	impairment	0	Pain management	0	Contraception	0	Peripheral	0	Episodic	0	Cranial nerve	0	Lactation	2	Beta-blocker	0	Efficacy	0	Antihistamine	0	Advantage	0	Progression	0	Productivity	0	Nerve block	0	Accepting	0	Trigeminal autonomic cephalalgia	0	Detoxification	0	Secondary headache	0	spouse	0	Lidocaine	0	Blood flow	0	Double vision	0	Intracranial hypertension	0	Dopamine	0	church	0	Mechanism	0	Complications	0	Attitude	0	Spectrum	0	Consultation	0	Empower	0	Frustrate	0	Complicated migraine	0	5-HT	0	Ineffective	0	Eye symptom	0	Brain lesion	0	Physical examination	0	Referral
surgery	0	Medication overuse	0	Mindfulness	0	Tricyclic	0	Histamine	0	Remission	0	Regimen	0	Anti-seizure	0	Occipital nerve	0	Menstrual migraine	0	Type A	0	Lifestyle change	0	Headache clinic	0	impairment	0	Pain management	0	Contraception	0	Peripheral	0	Episodic	0	Cranial nerve	0	Lactation	0	Beta-blocker	0	Efficacy	0	Antihistamine	0	Advantage	0	Progression	0	Productivity	0	Nerve block	0	Accepting	0	Trigeminal autonomic cephalalgia	0	Detoxification	0	Secondary headache	0	spouse	0	Lidocaine	0	Blood flow	0	Double vision	0	Intracranial hypertension	0	Dopamine	0	church	0	Mechanism	0	Complications	0	Attitude	0	Spectrum	0	Consultation	1	Empower	0	Frustrate	0	Complicated migraine	0	5-HT	0	Ineffective	0	Eye symptom	0	Brain lesion	0	Physical examination	1	Referral
surgery	0	Medication overuse	0	Mindfulness	0	Tricyclic	0	Histamine	0	Remission	0	Regimen	1	Anti-seizure	0	Occipital nerve	0	Menstrual migraine	0	Type A	0	Lifestyle change	0	Headache clinic	0	impairment	0	Pain management	0	Contraception	0	Peripheral	0	Episodic	0	Cranial nerve	0	Lactation	0	Beta-blocker	0	Efficacy	0	Antihistamine	0	Advantage	0	Progression	0	Productivity	0	Nerve block	0	Accepting	0	Trigeminal autonomic cephalalgia	0	Detoxification	0	Secondary headache	0	spouse	0	Lidocaine	0	Blood flow	0	Double vision	0	Intracranial hypertension	0	Dopamine	0	church	0	Mechanism	0	Complications	0	Attitude	0	Spectrum	0	Consultation	0	Empower	0	Frustrate	0	Complicated migraine	0	5-HT	0	Ineffective	0	Eye symptom	0	Brain lesion	0	Physical examination	0	Referral
surgery	0	Medication overuse	0	Mindfulness	0	Tricyclic	0	Histamine	0	Remission	0	Regimen	1	Anti-seizure	0	Occipital nerve	0	Menstrual migraine	0	Type A	0	Lifestyle change	0	Headache clinic	0	impairment	0	Pain management	0	Contraception	0	Peripheral	0	Episodic	0	Cranial nerve	0	Lactation	0	Beta-blocker	0	Efficacy	0	Antihistamine	0	Advantage	0	Progression	0	Productivity	0	Nerve block	0	Accepting	0	Trigeminal autonomic cephalalgia	0	Detoxification	0	Secondary headache	0	spouse	0	Lidocaine	0	Blood flow	0	Double vision	0	Intracranial hypertension	0	Dopamine	0	church	0	Mechanism	0	Complications	0	Attitude	0	Spectrum	0	Consultation	0	Empower	0	Frustrate	0	Complicated migraine	0	5-HT	0	Ineffective	0	Eye symptom	0	Brain lesion	0	Physical examination	0	Referral
surgery	0	Medication overuse	0	Mindfulness	0	Tricyclic	0	Histamine	0	Remission	0	Regimen	0	Anti-seizure	0	Occipital nerve	0	Menstrual migraine	0	Type A	0	Lifestyle change	0	Headache clinic	0	impairment	0	Pain management	0	Contraception	0	Peripheral	0	Episodic	0	Cranial nerve	0	Lactation	0	Beta-blocker	0	Efficacy	0	Antihistamine	0	Advantage	0	Progression	0	Productivity	0	Nerve block	0	Accepting	0	Trigeminal autonomic cephalalgia	0	Detoxification	0	Secondary headache	0	spouse	0	Lidocaine	0	Blood flow	0	Double vision	0	Intracranial hypertension	0	Dopamine	0	church	0	Mechanism	0	Complications	0	Attitude	0	Spectrum	0	Consultation	0	Empower	0	Frustrate	0	Complicated migraine	0	5-HT	0	Ineffective	0	Eye symptom	0	Brain lesion	0	Physical examination	0	Referral
surgery	0	Medication overuse	0	Mindfulness	0	Tricyclic	0	Histamine	0	Remission	0	Regimen	0	Anti-seizure	0	Occipital nerve	0	Menstrual migraine	0	Type A	0	Lifestyle change	0	Headache clinic	0	impairment	0	Pain management	0	Contraception	0	Peripheral	0	Episodic	0	Cranial nerve	0	Lactation	0	Beta-blocker	1	Efficacy	0	Antihistamine	0	Advantage	0	Progression	0	Productivity	0	Nerve block	0	Accepting	0	Trigeminal autonomic cephalalgia	0	Detoxification	0	Secondary headache	0	spouse	0	Lidocaine	0	Blood flow	0	Double vision	0	Intracranial hypertension	0	Dopamine	0	church	0	Mechanism	0	Complications	0	Attitude	0	Spectrum	0	Consultation	0	Empower	0	Frustrate	0	Complicated migraine	0	5-HT	0	Ineffective	0	Eye symptom	0	Brain lesion	1	Physical examination	0	Referral
surgery	0	Medication overuse	0	Mindfulness	0	Tricyclic	0	Histamine	0	Remission	0	Regimen	0	Anti-seizure	0	Occipital nerve	0	Menstrual migraine	0	Type A	0	Lifestyle change	0	Headache clinic	0	impairment	0	Pain management	0	Contraception	0	Peripheral	0	Episodic	0	Cranial nerve	0	Lactation	0	Beta-blocker	0	Efficacy	0	Antihistamine	0	Advantage	0	Progression	0	Productivity	0	Nerve block	0	Accepting	0	Trigeminal autonomic cephalalgia	0	Detoxification	0	Secondary headache	0	spouse	0	Lidocaine	0	Blood flow	0	Double vision	0	Intracranial hypertension	0	Dopamine	0	church	0	Mechanism	0	Complications	0	Attitude	0	Spectrum	0	Consultation	0	Empower	0	Frustrate	0	Complicated migraine	0	5-HT	0	Ineffective	0	Eye symptom	0	Brain lesion	0	Physical examination	0	Referral
surgery	0	Medication overuse	0	Mindfulness	0	Tricyclic	0	Histamine	0	Remission	0	Regimen	0	Anti-seizure	0	Occipital nerve	0	Menstrual migraine	0	Type A	0	Lifestyle change	0	Headache clinic	0	impairment	0	Pain management	0	Contraception	0	Peripheral	0	Episodic	0	Cranial nerve	0	Lactation	0	Beta-blocker	0	Efficacy	0	Antihistamine	0	Advantage	0	Progression	0	Productivity	0	Nerve block	0	Accepting	0	Trigeminal autonomic cephalalgia	0	Detoxification	0	Secondary headache	0	spouse	0	Lidocaine	0	Blood flow	0	Double vision	0	Intracranial hypertension	0	Dopamine	0	church	0	Mechanism	0	Complications	0	Attitude	0	Spectrum	0	Consultation	0	Empower	0	Frustrate	0	Complicated migraine	0	5-HT	0	Ineffective	0	Eye symptom	0	Brain lesion	0	Physical examination	0	Referral
surgery	1	Medication overuse	0	Mindfulness	0	Tricyclic	0	Histamine	0	Remission	0	Regimen	0	Anti-seizure	0	Occipital nerve	0	Menstrual migraine	0	Type A	1	Lifestyle change	0	Headache clinic	0	impairment	0	Pain management	0	Contraception	0	Peripheral	0	Episodic	0	Cranial nerve	0	Lactation	0	Beta-blocker	0	Efficacy	0	Antihistamine	0	Advantage	0	Progression	0	Productivity	0	Nerve block	0	Accepting	0	Trigeminal autonomic cephalalgia	0	Detoxification	0	Secondary headache	0	spouse	0	Lidocaine	0	Blood flow	0	Double vision	0	Intracranial hypertension	0	Dopamine	0	church	1	Mechanism	0	Complications	0	Attitude	0	Spectrum	0	Consultation	0	Empower	0	Frustrate	0	Complicated migraine	0	5-HT	1	Ineffective	1	Eye symptom	1	Brain lesion	0	Physical examination	0	Referral
surgery	0	Medication overuse	0	Mindfulness	0	Tricyclic	0	Histamine	0	Remission	0	Regimen	0	Anti-seizure	0	Occipital nerve	0	Menstrual migraine	0	Type A	0	Lifestyle change	0	Headache clinic	0	impairment	0	Pain management	0	Contraception	0	Peripheral	0	Episodic	0	Cranial nerve	0	Lactation	0	Beta-blocker	0	Efficacy	0	Antihistamine	0	Advantage	0	Progression	0	Productivity	0	Nerve block	0	Accepting	0	Trigeminal autonomic cephalalgia	0	Detoxification	0	Secondary headache	0	spouse	0	Lidocaine	0	Blood flow	0	Double vision	0	Intracranial hypertension	0	Dopamine	0	church	0	Mechanism	0	Complications	0	Attitude	0	Spectrum	0	Consultation	0	Empower	0	Frustrate	0	Complicated migraine	0	5-HT	0	Ineffective	0	Eye symptom	0	Brain lesion	0	Physical examination	0	Referral
surgery	0	Medication overuse	0	Mindfulness	1	Tricyclic	0	Histamine	0	Remission	1	Regimen	0	Anti-seizure	0	Occipital nerve	0	Menstrual migraine	0	Type A	0	Lifestyle change	0	Headache clinic	0	impairment	0	Pain management	0	Contraception	0	Peripheral	0	Episodic	0	Cranial nerve	0	Lactation	0	Beta-blocker	0	Efficacy	0	Antihistamine	0	Advantage	0	Progression	0	Productivity	0	Nerve block	0	Accepting	0	Trigeminal autonomic cephalalgia	0	Detoxification	0	Secondary headache	0	spouse	0	Lidocaine	0	Blood flow	0	Double vision	0	Intracranial hypertension	0	Dopamine	0	church	0	Mechanism	0	Complications	0	Attitude	0	Spectrum	0	Consultation	0	Empower	0	Frustrate	0	Complicated migraine	0	5-HT	0	Ineffective	0	Eye symptom	0	Brain lesion	0	Physical examination	0	Referral
surgery	0	Medication overuse	0	Mindfulness	0	Tricyclic	0	Histamine	0	Remission	0	Regimen	0	Anti-seizure	0	Occipital nerve	0	Menstrual migraine	0	Type A	0	Lifestyle change	0	Headache clinic	0	impairment	0	Pain management	0	Contraception	0	Peripheral	0	Episodic	0	Cranial nerve	0	Lactation	0	Beta-blocker	0	Efficacy	0	Antihistamine	0	Advantage	0	Progression	0	Productivity	0	Nerve block	0	Accepting	0	Trigeminal autonomic cephalalgia	0	Detoxification	0	Secondary headache	0	spouse	0	Lidocaine	0	Blood flow	0	Double vision	0	Intracranial hypertension	0	Dopamine	0	church	0	Mechanism	0	Complications	0	Attitude	0	Spectrum	0	Consultation	0	Empower	0	Frustrate	0	Complicated migraine	1	5-HT	0	Ineffective	0	Eye symptom	0	Brain lesion	0	Physical examination	0	Referral
surgery	0	Medication overuse	0	Mindfulness	0	Tricyclic	0	Histamine	0	Remission	0	Regimen	0	Anti-seizure	0	Occipital nerve	0	Menstrual migraine	0	Type A	0	Lifestyle change	0	Headache clinic	0	impairment	0	Pain management	0	Contraception	0	Peripheral	0	Episodic	0	Cranial nerve	0	Lactation	0	Beta-blocker	0	Efficacy	0	Antihistamine	0	Advantage	0	Progression	0	Productivity	0	Nerve block	0	Accepting	0	Trigeminal autonomic cephalalgia	0	Detoxification	0	Secondary headache	0	spouse	0	Lidocaine	0	Blood flow	0	Double vision	0	Intracranial hypertension	0	Dopamine	0	church	0	Mechanism	0	Complications	0	Attitude	0	Spectrum	0	Consultation	0	Empower	0	Frustrate	0	Complicated migraine	0	5-HT	0	Ineffective	0	Eye symptom	0	Brain lesion	0	Physical examination	0	Referral
surgery	0	Medication overuse	0	Mindfulness	0	Tricyclic	0	Histamine	0	Remission	0	Regimen	0	Anti-seizure	0	Occipital nerve	0	Menstrual migraine	0	Type A	1	Lifestyle change	0	Headache clinic	0	impairment	0	Pain management	0	Contraception	0	Peripheral	0	Episodic	0	Cranial nerve	0	Lactation	0	Beta-blocker	0	Efficacy	0	Antihistamine	0	Advantage	0	Progression	0	Productivity	0	Nerve block	0	Accepting	0	Trigeminal autonomic cephalalgia	0	Detoxification	0	Secondary headache	0	spouse	0	Lidocaine	0	Blood flow	0	Double vision	0	Intracranial hypertension	0	Dopamine	0	church	0	Mechanism	0	Complications	0	Attitude	0	Spectrum	0	Consultation	0	Empower	0	Frustrate	0	Complicated migraine	0	5-HT	0	Ineffective	0	Eye symptom	0	Brain lesion	0	Physical examination	0	Referral
surgery	0	Medication overuse	0	Mindfulness	0	Tricyclic	0	Histamine	0	Remission	0	Regimen	0	Anti-seizure	0	Occipital nerve	0	Menstrual migraine	0	Type A	0	Lifestyle change	0	Headache clinic	0	impairment	0	Pain management	0	Contraception	0	Peripheral	0	Episodic	0	Cranial nerve	0	Lactation	0	Beta-blocker	0	Efficacy	0	Antihistamine	0	Advantage	0	Progression	0	Productivity	0	Nerve block	0	Accepting	0	Trigeminal autonomic cephalalgia	0	Detoxification	0	Secondary headache	0	spouse	0	Lidocaine	0	Blood flow	0	Double vision	0	Intracranial hypertension	0	Dopamine	0	church	0	Mechanism	0	Complications	0	Attitude	0	Spectrum	0	Consultation	0	Empower	0	Frustrate	0	Complicated migraine	0	5-HT	0	Ineffective	0	Eye symptom	0	Brain lesion	0	Physical examination	0	Referral
surgery	0	Medication overuse	0	Mindfulness	2	Tricyclic	0	Histamine	0	Remission	0	Regimen	0	Anti-seizure	0	Occipital nerve	0	Menstrual migraine	0	Type A	0	Lifestyle change	0	Headache clinic	0	impairment	0	Pain management	0	Contraception	0	Peripheral	0	Episodic	1	Cranial nerve	2	Lactation	0	Beta-blocker	0	Efficacy	0	Antihistamine	0	Advantage	0	Progression	0	Productivity	1	Nerve block	0	Accepting	0	Trigeminal autonomic cephalalgia	0	Detoxification	0	Secondary headache	0	spouse	0	Lidocaine	0	Blood flow	0	Double vision	0	Intracranial hypertension	0	Dopamine	0	church	0	Mechanism	0	Complications	0	Attitude	0	Spectrum	0	Consultation	0	Empower	0	Frustrate	1	Complicated migraine	0	5-HT	0	Ineffective	0	Eye symptom	0	Brain lesion	0	Physical examination	0	Referral
surgery	0	Medication overuse	0	Mindfulness	0	Tricyclic	0	Histamine	0	Remission	0	Regimen	0	Anti-seizure	0	Occipital nerve	0	Menstrual migraine	0	Type A	0	Lifestyle change	0	Headache clinic	0	impairment	0	Pain management	0	Contraception	0	Peripheral	0	Episodic	0	Cranial nerve	0	Lactation	0	Beta-blocker	0	Efficacy	0	Antihistamine	0	Advantage	0	Progression	0	Productivity	0	Nerve block	0	Accepting	0	Trigeminal autonomic cephalalgia	0	Detoxification	0	Secondary headache	0	spouse	0	Lidocaine	0	Blood flow	0	Double vision	0	Intracranial hypertension	0	Dopamine	0	church	0	Mechanism	0	Complications	0	Attitude	0	Spectrum	0	Consultation	0	Empower	0	Frustrate	0	Complicated migraine	0	5-HT	0	Ineffective	0	Eye symptom	0	Brain lesion	0	Physical examination	0	Referral
surgery	0	Medication overuse	0	Mindfulness	0	Tricyclic	0	Histamine	0	Remission	0	Regimen	0	Anti-seizure	0	Occipital nerve	0	Menstrual migraine	0	Type A	0	Lifestyle change	0	Headache clinic	0	impairment	0	Pain management	0	Contraception	0	Peripheral	0	Episodic	0	Cranial nerve	0	Lactation	0	Beta-blocker	0	Efficacy	0	Antihistamine	0	Advantage	0	Progression	0	Productivity	0	Nerve block	0	Accepting	0	Trigeminal autonomic cephalalgia	0	Detoxification	0	Secondary headache	0	spouse	0	Lidocaine	0	Blood flow	0	Double vision	0	Intracranial hypertension	0	Dopamine	0	church	0	Mechanism	0	Complications	0	Attitude	0	Spectrum	0	Consultation	0	Empower	0	Frustrate	0	Complicated migraine	0	5-HT	0	Ineffective	0	Eye symptom	0	Brain lesion	0	Physical examination	0	Referral
	4		4		4		3		3		3		3		3		3		3		3		3		3		3		3		3		3		3		2		2		2		2		2		2		2		2		2		2		2		2		2		2		2		2		2		2		2		2		2		2		2		2		2		1		1		1		1		1		1		1	

P232b	P233a	P233b	P234a	P234b	P235a	P235b	P236a	P236b	P237a	P237b	P238a	P238b	P239a	P239b	P240a	P240b	P241a	P241b	P242a	P242b	P243a	P243b	P244a	P244b	P245a	P245b	P246a	P246b	P247a	P247b	P248a	P248b	P249a	P249b	P250a	P250b	P251a	P251b	P252a	P252b	P253a	P253b	P254a	P254b	P255a	P255b	P256a	P256b	P257a	P257b	P258a	P258b	P259a	P259b	P260a	P260b	P261a	P261b	P262a	P262b	P263a	P263b	P264a	P264b	P265a	P265b	P266a	P266b	P267a	P267b	P268a	P268b	P269a	P269b	P270a	P270b	P271a	P271b	P272a	P272b	P273a	P273b	P274a	P274b	P275a	P275b	P276a	P276b	P277a	P277b	P278a	P278b	P279a	P279b
0	Beta-Blockers	0	Foramen ovale	0	Angiotensin Converting Enzyme	0	Prophylactic treatment	0	Intervention	0	Subtype	0	Randomized controlled trial	0	Botulinum Toxin	0	Melatonin	0	Gene-related	0	Calcitonin	0	Caregiver	0	Temporal	0	Barbiturate	0	Septoplasty	0	max	0	Eye pain	0	Angry	0	Emergency care	0	Food allergies	0	Motivation	0	Catheter	0	SST	0	Serotonin receptor	0	Ink	0	Homeopathy	0	Placebo effect	0	Inpatient	0	Inpatient treatment	0	Female hormones	0	Social worker	0	Future	0	Risk factor	0	surgical	0	Surgical treatment	0	Referred pain	0	Nonsteroidal anti-inflammatory	0	Concussion	0	Mood disorder	0	Anatomy	0	Father	0	Preemptive	0	Grandfather	0	Adherence	0	Assessment	0	carers	0	Clinician	0
0	Beta-Blockers	0	Foramen ovale	0	Angiotensin Converting Enzyme	0	Prophylactic treatment	0	Intervention	0	Subtype	0	Randomized controlled trial	0	Botulinum Toxin	0	Melatonin	0	Gene-related	0	Calcitonin	0	Caregiver	0	Temporal	0	Barbiturate	0	Septoplasty	0	max	0	Eye pain	0	Angry	0	Emergency care	0	Food allergies	0	Motivation	0	Catheter	0	SST	0	Serotonin receptor	0	Ink	0	Homeopathy	0	Placebo effect	0	Inpatient	0	Inpatient treatment	0	Female hormones	0	Social worker	0	Future	0	Risk factor	0	surgical	0	Surgical treatment	0	Referred pain	0	Nonsteroidal anti-inflammatory	0	Concussion	0	Mood disorder	0	Anatomy	0	Father	0	Preemptive	0	Grandfather	0	Adherence	0	Assessment	0	carers	0	Clinician	0
0	Beta-Blockers	0	Foramen ovale	0	Angiotensin Converting Enzyme	0	Prophylactic treatment	0	Intervention	0	Subtype	0	Randomized controlled trial	0	Botulinum Toxin	0	Melatonin	0	Gene-related	0	Calcitonin	0	Caregiver	0	Temporal	0	Barbiturate	0	Septoplasty	0	max	0	Eye pain	0	Angry	0	Emergency care	0	Food allergies	0	Motivation	0	Catheter	0	SST	0	Serotonin receptor	0	Ink	0	Homeopathy	0	Placebo effect	0	Inpatient	0	Inpatient treatment	0	Female hormones	0	Social worker	0	Future	0	Risk factor	0	surgical	0	Surgical treatment	0	Referred pain	0	Nonsteroidal anti-inflammatory	0	Concussion	0	Mood disorder	0	Anatomy	0	Father	0	Preemptive	0	Grandfather	0	Adherence	0	Assessment	0	carers	0	Clinician	0
0	Beta-Blockers	0	Foramen ovale	0	Angiotensin Converting Enzyme	0	Prophylactic treatment	0	Intervention	0	Subtype	0	Randomized controlled trial	0	Botulinum Toxin	0	Melatonin	0	Gene-related	0	Calcitonin	0	Caregiver	0	Temporal	0	Barbiturate	0	Septoplasty	0	max	0	Eye pain	0	Angry	0	Emergency care	0	Food allergies	0	Motivation	0	Catheter	0	SST	0	Serotonin receptor	0	Ink	0	Homeopathy	0	Placebo effect	0	Inpatient	0	Inpatient treatment	0	Female hormones	0	Social worker	0	Future	0	Risk factor	0	surgical	0	Surgical treatment	0	Referred pain	0	Nonsteroidal anti-inflammatory	0	Concussion	0	Mood disorder	0	Anatomy	0	Father	0	Preemptive	0	Grandfather	0	Adherence	0	Assessment	0	carers	0	Clinician	0
0	Beta-Blockers	0	Foramen ovale	0	Angiotensin Converting Enzyme	0	Prophylactic treatment	0	Intervention	0	Subtype	0	Randomized controlled trial	0	Botulinum Toxin	0	Melatonin	0	Gene-related	0	Calcitonin	0	Caregiver	0	Temporal	0	Barbiturate	0	Septoplasty	0	max	0	Eye pain	0	Angry	0	Emergency care	0	Food allergies	0	Motivation	0	Catheter	0	SST	0	Serotonin receptor	0	Ink	0	Homeopathy	0	Placebo effect	0	Inpatient	0	Inpatient treatment	0	Female hormones	0	Social worker	0	Future	0	Risk factor	0	surgical	0	Surgical treatment	0	Referred pain	0	Nonsteroidal anti-inflammatory	0	Concussion	0	Mood disorder	0	Anatomy	0	Father	0	Preemptive	0	Grandfather	0	Adherence	0	Assessment	0	carers	0	Clinician	0
0	Beta-Blockers	0	Foramen ovale	0	Angiotensin Converting Enzyme	0	Prophylactic treatment	0	Intervention	0	Subtype	0	Randomized controlled trial	0	Botulinum Toxin	0	Melatonin	0	Gene-related	0	Calcitonin	0	Caregiver	0	Temporal	0	Barbiturate	0	Septoplasty	0	max	0	Eye pain	0	Angry	0	Emergency care	0	Food allergies	0	Motivation	0	Catheter	0	SST	0	Serotonin receptor	0	Ink	0	Homeopathy	0	Placebo effect	0	Inpatient	0	Inpatient treatment	0	Female hormones	0	Social worker	0	Future	0	Risk factor	0	surgical	0	Surgical treatment	0	Referred pain	0	Nonsteroidal anti-inflammatory	0	Concussion	0	Mood disorder	0	Anatomy	0	Father	0	Preemptive	0	Grandfather	0	Adherence	0	Assessment	0	carers	0	Clinician	0
0	Beta-Blockers	0	Foramen ovale	0	Angiotensin Converting Enzyme	0	Prophylactic treatment	0	Intervention	0	Subtype	0	Randomized controlled trial	0	Botulinum Toxin	0	Melatonin	0	Gene-related	0	Calcitonin	0	Caregiver	0	Temporal	0	Barbiturate	0	Septoplasty	0	max	0	Eye pain	0	Angry	0	Emergency care	0	Food allergies	0	Motivation	0	Catheter	0	SST	0	Serotonin receptor	0	Ink	0	Homeopathy	0	Placebo effect	0	Inpatient	0	Inpatient treatment	0	Female hormones	0	Social worker	0	Future	0	Risk factor	0	surgical	0	Surgical treatment	0	Referred pain	0	Nonsteroidal anti-inflammatory	0	Concussion	0	Mood disorder	0	Anatomy	0	Father	0	Preemptive	0	Grandfather	0	Adherence	0	Assessment	0	carers	0	Clinician	0
0	Beta-Blockers	0	Foramen ovale	0	Angiotensin Converting Enzyme	0	Prophylactic treatment	0	Intervention	0	Subtype	0	Randomized controlled trial	0	Botulinum Toxin	0	Melatonin	0	Gene-related	0	Calcitonin	0	Caregiver	0	Temporal	0	Barbiturate	0	Septoplasty	0	max	0	Eye pain	0	Angry	0	Emergency care	0	Food allergies	0	Motivation	0	Catheter	0	SST	0	Serotonin receptor	0	Ink	0	Homeopathy	0	Placebo effect	0	Inpatient	0	Inpatient treatment	0	Female hormones	0	Social worker	0	Future	0	Risk factor	0	surgical	0	Surgical treatment	0	Referred pain	0	Nonsteroidal anti-inflammatory	0	Concussion	0	Mood disorder	0	Anatomy	0	Father	0	Preemptive	0	Grandfather	0	Adherence	0	Assessment	0	carers	0	Clinician	0
0	Beta-Blockers	0	Foramen ovale	0	Angiotensin Converting Enzyme	0	Prophylactic treatment	0	Intervention	0	Subtype	0	Randomized controlled trial	0	Botulinum Toxin	0	Melatonin	0	Gene-related	0	Calcitonin	0	Caregiver	0	Temporal	0	Barbiturate	0	Septoplasty	0	max	0	Eye pain	0	Angry	0	Emergency care	0	Food allergies	0	Motivation	0	Catheter	0	SST	0	Serotonin receptor	0	Ink	0	Homeopathy	0	Placebo effect	0	Inpatient	0	Inpatient treatment	0	Female hormones	0	Social worker	0	Future	0	Risk factor	0	surgical	0	Surgical treatment	0	Referred pain	0	Nonsteroidal anti-inflammatory	0	Concussion	0	Mood disorder	0	Anatomy	0	Father	0	Preemptive	0	Grandfather	0	Adherence	0	Assessment	0	carers	0	Clinician	0
0	Beta-Blockers	0	Foramen ovale	0	Angiotensin Converting Enzyme	0	Prophylactic treatment	0	Intervention	0	Subtype	0	Randomized controlled trial	0	Botulinum Toxin	0	Melatonin	0	Gene-related	0	Calcitonin	0	Caregiver	0	Temporal	0	Barbiturate	0	Septoplasty	0	max	0	Eye pain	0	Angry	0	Emergency care	0	Food allergies	0	Motivation	0	Catheter	0	SST	0	Serotonin receptor	0	Ink	0	Homeopathy	0	Placebo effect	0	Inpatient	0	Inpatient treatment	0	Female hormones	0	Social worker	0	Future	0	Risk factor	0	surgical	0	Surgical treatment	0	Referred pain	0	Nonsteroidal anti-inflammatory	0	Concussion	0	Mood disorder	0	Anatomy	0	Father	0	Preemptive	0	Grandfather	0	Adherence	0	Assessment	0	carers	0	Clinician	0

May 2021 Migraine Webcrawl Data

Condition	URL Id	URL	Total number of words	P0a	P0b	P1a	P1b	P2a	P2b	P3a	P3b	P4a	P4b	P5a	P5b	P6a	P6b
Migraine	1951845	http://curetogether.com/Migraine/discuss/	36387	Migraine	487	Ache	137	Ill	213	Pain	153	work	141	drug	45	Doctor	70
Migraine	1765289	http://forums.phoenixrising.me/index.php?threads/questions-about-migraine-aura.34477/	4378	Migraine	64	Ache	11	Ill	18	Pain	9	work	9	drug	6	Doctor	2
Migraine	2283240	http://forums.prohealth.com/forums/index.php?threads/migraine-from-hell-called-status-migrainosus.110648/	1390	Migraine	17	Ache	4	Ill	4	Pain	9	work	2	drug	0	Doctor	5
Migraine	1951709	http://forums.whirlpool.net.au/archive/1924289	8822	Migraine	88	Ache	38	Ill	42	Pain	42	work	26	drug	5	Doctor	13
Migraine	2174178	http://helpforheadaches.com/forum.htm	902	Migraine	15	Ache	12	Ill	3	Pain	0	work	3	drug	0	Doctor	2
Migraine	2275843	http://migrainepage.forumotion.net/	189	Migraine	0	Ache	0	Ill	0	Pain	0	work	0	drug	0	Doctor	0
Migraine	2275852	http://migrainepage.forumotion.net/f1-migraines	1230	Migraine	6	Ache	0	Ill	0	Pain	0	work	0	drug	0	Doctor	0
Migraine	2293278	http://migrainetalk.forumotion.co.uk/	923	Migraine	15	Ache	0	Ill	0	Pain	0	work	0	drug	0	Doctor	0
Migraine	2053758	http://vetsbenefits.net/topic/67841/Migraines?page=2	135	Migraine	0	Ache	0	Ill	0	Pain	0	work	0	drug	0	Doctor	0
Migraine	2247837	http://www.experienceproject.com/groups/Get-Migraine-Headaches/102	43	Migraine	0	Ache	0	Ill	1	Pain	0	work	0	drug	0	Doctor	0
Migraine	2242530	http://www.experienceproject.com/groups/Have-Vestibular-Migraine/117436	43	Migraine	0	Ache	0	Ill	1	Pain	0	work	0	drug	0	Doctor	0
Migraine	2275938	http://www.fibromyalgiaforums.org/forum/general-discussions-about-fibromyalgia/26893-fibro-migraines.html	2540	Migraine	27	Ache	20	Ill	4	Pain	18	work	10	drug	0	Doctor	3
Migraine	2277089	http://www.fightingfatigue.org/headache-migraine-websites-forums/	418	Migraine	5	Ache	4	Ill	0	Pain	2	work	0	drug	0	Doctor	0
Migraine	1679505	http://www.healthcentral.com/migraine/	547	Migraine	19	Ache	5	Ill	1	Pain	1	work	1	drug	0	Doctor	1
Migraine	1679511	http://www.healthcentral.com/migraine/c/123/177401/experience-spring-migraine	1259	Migraine	42	Ache	4	Ill	3	Pain	0	work	1	drug	0	Doctor	1
Migraine	2250921	http://www.healthfulchat.org/migraine-chat-room.html	1063	Migraine	25	Ache	8	Ill	1	Pain	2	work	7	drug	0	Doctor	0
Migraine	2029502	http://www.helpforheadaches.com/	389	Migraine	13	Ache	7	Ill	4	Pain	0	work	1	drug	0	Doctor	1
Migraine	1679502	http://www.medhelp.org/forums/Migraines--Headaches/show/123	1337	Migraine	11	Ache	20	Ill	1	Pain	9	work	1	drug	0	Doctor	4
Migraine	2277487	http://www.migraine.org.uk/	348	Migraine	9	Ache	1	Ill	0	Pain	0	work	0	drug	0	Doctor	0
Migraine	2292659	http://www.migraine.org.uk/get-advice/	440	Migraine	9	Ache	0	Ill	0	Pain	0	work	0	drug	0	Doctor	0
Migraine	2069158	http://www.migrainebuddy.com/migraine-stories/	526	Migraine	15	Ache	1	Ill	2	Pain	3	work	2	drug	0	Doctor	0
Migraine	2063539	http://www.migrainebuddy.com/understanding-migraines-the-gender-divide/	2038	Migraine	117	Ache	3	Ill	5	Pain	14	work	7	drug	1	Doctor	4
Migraine	2286545	http://www.migrainepage.com/	23	Migraine	0	Ache	0	Ill	0	Pain	0	work	0	drug	0	Doctor	0
Migraine	2291250	http://www.migrainepage.com/help.html	287	Migraine	10	Ache	8	Ill	0	Pain	0	work	0	drug	0	Doctor	0
Migraine	2291906	http://www.migraines.org/help/helpform.htm	340	Migraine	8	Ache	2	Ill	1	Pain	0	work	1	drug	0	Doctor	0
Migraine	1679496	http://www.mvertigo.org/	363	Migraine	4	Ache	1	Ill	0	Pain	0	work	0	drug	0	Doctor	0
Migraine	2041773	http://www.nomorepanic.co.uk/showthread.php?t=106093	1917	Migraine	16	Ache	0	Ill	5	Pain	5	work	3	drug	0	Doctor	1
Migraine	2289385	http://www.relieve-migraine-headache.com/basilar-migraines.html	773	Migraine	20	Ache	5	Ill	2	Pain	0	work	0	drug	0	Doctor	5
Migraine	2277529	http://www.thedailyheadache.com/	3540	Migraine	35	Ache	18	Ill	40	Pain	1	work	14	drug	5	Doctor	7
Migraine	2277957	http://www.thedailymigraine.com/forum-moot/	121	Migraine	4	Ache	0	Ill	0	Pain	0	work	0	drug	0	Doctor	0
Migraine	2290740	http://www.thedailymigraine.com/support-groups/	290	Migraine	11	Ache	0	Ill	2	Pain	6	work	0	drug	0	Doctor	0
Migraine	2274213	http://www.themigrainerevolution.com/yahoo-group/	501	Migraine	16	Ache	0	Ill	2	Pain	0	work	0	drug	1	Doctor	0
Migraine	2294811	http://www.tmswiki.org/forum/threads/my-migraines.2072/	2467	Migraine	30	Ache	9	Ill	8	Pain	6	work	7	drug	3	Doctor	1
Migraine	2244789	http://www.topix.com/forum/health/migraine	1794	Migraine	24	Ache	5	Ill	4	Pain	3	work	0	drug	5	Doctor	2
Migraine	1875338	http://www.usmle-forums.com/usmle-step-1-forum/217482-migraine.html	660	Migraine	4	Ache	1	Ill	0	Pain	0	work	0	drug	0	Doctor	0
Migraine	2295373	https://cassiopaea.org/forum/?topic=17971.0	2196	Migraine	0	Ache	0	Ill	4	Pain	0	work	2	drug	0	Doctor	0
Migraine	2289387	https://coloradopain.co/patient-forms/	383	Migraine	0	Ache	0	Ill	2	Pain	5	work	2	drug	0	Doctor	1
Migraine	2276050	https://community.qvc.com/t5/Wellness/MIGRAINE-sufferers/td-p/1182015	3353	Migraine	34	Ache	3	Ill	6	Pain	6	work	8	drug	0	Doctor	2
Migraine	1752114	https://curetogether.com/Migraine/discuss/	36747	Migraine	487	Ache	137	Ill	213	Pain	153	work	141	drug	45	Doctor	79
Migraine	2268735	https://ehealthforum.com/health/alzheimers.html	715	Migraine	0	Ache	0	Ill	1	Pain	0	work	0	drug	1	Doctor	3
Migraine	2291645	https://ehealthforum.com/health/epilepsy.html	652	Migraine	0	Ache	1	Ill	1	Pain	0	work	0	drug	1	Doctor	3
Migraine	2278014	https://ehealthforum.com/health/headache-and-migraines-question-t504544.html	746	Migraine	1	Ache	8	Ill	1	Pain	0	work	1	drug	1	Doctor	6
Migraine	2285137	https://ergoweb.com/forums/topic/treatment-of-light-induced-migraine-headaches-in-the-office/	1236	Migraine	11	Ache	6	Ill	4	Pain	0	work	6	drug	0	Doctor	0
Migraine	2296496	https://exchanges.webmd.com/migraines-and-headaches-exchange	6384	Migraine	80	Ache	56	Ill	27	Pain	16	work	6	drug	7	Doctor	25
Migraine	2286108	https://forum.bulletproof.com/discussion/1406/bulletproof-migraine-specific-diet	1772	Migraine	21	Ache	11	Ill	5	Pain	3	work	5	drug	0	Doctor	4
Migraine	2290131	https://forum.dontpayfull.com/threads/migraine-medicine.25791/	3210	Migraine	36	Ache	29	Ill	17	Pain	16	work	13	drug	0	Doctor	6
Migraine	2282154	https://forum.grasscity.com/threads/migraines.453628/	2514	Migraine	47	Ache	14	Ill	9	Pain	8	work	3	drug	4	Doctor	3
Migraine	2273025	https://forums.phoenixrising.me/index.php?threads/questions-about-migraine-aura.34477/	4350	Migraine	64	Ache	11	Ill	18	Pain	9	work	9	drug	6	Doctor	2
Migraine	2095418	https://forums.whirlpool.net.au/archive/1924289	8822	Migraine	88	Ache	38	Ill	42	Pain	42	work	26	drug	5	Doctor	13
Migraine	2294008	https://hubpages.com/health/forum/27466/any-ocular-migraine-sufferers-here	3029	Migraine	28	Ache	5	Ill	6	Pain	9	work	7	drug	0	Doctor	2
Migraine	2208143	https://livingwell.migrainedisease.com	350	Migraine	7	Ache	3	Ill	3	Pain	0	work	0	drug	0	Doctor	0
Migraine	2274362	https://livingwell.migrainedisease.com/forum.htm	902	Migraine	15	Ache	12	Ill	3	Pain	0	work	3	drug	0	Doctor	2
Migraine	2170918	https://livingwell.migrainedisease.com/www	389	Migraine	13	Ache	7	Ill	4	Pain	0	work	1	drug	0	Doctor	1
Migraine	2287140	https://migraine.newlifeoutlook.com/community/	523	Migraine	9	Ache	1	Ill	0	Pain	0	work	0	drug	0	Doctor	1
Migraine	2291171	https://migraine.newlifeoutlook.com/community/discussion/17/complex-migraine	681	Migraine	13	Ache	8	Ill	1	Pain	1	work	1	drug	0	Doctor	0
Migraine	2269712	https://migrainebuddy.com/migraine-stories/	526	Migraine	15	Ache	1	Ill	2	Pain	3	work	2	drug	0	Doctor	0
Migraine	2270662	https://migrainebuddy.com/migraine/2018/9/13/understanding-migraines-the-gender-divide	1826	Migraine	103	Ache	2	Ill	6	Pain	14	work	7	drug	1	Doctor	4
Migraine	2100374	https://migrainebuddy.com/understanding-migraines-the-gender-divide/	2038	Migraine	117	Ache	3	Ill	5	Pain	14	work	7	drug	1	Doctor	4
Migraine	2270732	https://migraineresearchfoundation.org/resources/resources-and-links/	1691	Migraine	37	Ache	34	Ill	11	Pain	4	work	2	drug	1	Doctor	5
Migraine	2251382	https://migrainetalkblog.wordpress.com/	1208	Migraine	57	Ache	9	Ill	3	Pain	0	work	0	drug	0	Doctor	0
Migraine	2283689	https://patient.info/forums/discuss/browse/migraine-1415	1131	Migraine	6	Ache	2	Ill	1	Pain	1	work	0	drug	0	Doctor	2
Migraine	2268233	https://vetsbenefits.net/migraines-t67841.html	8897	Migraine	134	Ache	71	Ill	20	Pain	18	work	41	drug	1	Doctor	12
Migraine	2283242	https://www.chronofhorse.com/forum/archive/index.php/t-318339.html	5948	Migraine	38	Ache	12	Ill	32	Pain	9	work	12	drug	1	Doctor	3
Migraine	2296236	https://www.curezone.org/forums/am.asp?i=60104	1592	Migraine	23	Ache	6	Ill	6	Pain	2	work	10	drug	2	Doctor	0
Migraine	2294557	https://www.curezone.org/forums/f3.asp?f=68	3380	Migraine	22	Ache	21	Ill	10	Pain	11	work	4	drug	2	Doctor	1
Migraine	2294387	https://www.dailystrength.org/group/migraine-headaches	826	Migraine	14	Ache	3	Ill	0	Pain	1	work	0	drug	2	Doctor	0
Migraine	2294417	https://www.dinet.org/forums/topic/27868-anybody-else-have-an-intractable-migraine/	2140	Migraine	21	Ache	11	Ill	6	Pain	16	work	8	drug	0	Doctor	6
Migraine	2273344	https://www.drugabuse.gov/related-topics/substance-use-suds-in-lgbt-populations	1468	Migraine	0	Ache	0	Ill	3	Pain	1	work	0	drug	11	Doctor	0
Migraine	2247957	https://www.eumom.ie/forums/topic/bad-bad-migraine/	1330	Migraine	10	Ache	3	Ill	4	Pain	3	work	2	drug	1	Doctor	3
Migraine	2272107	https://www.facebook.com/MigraineDotCom	375	Migraine	5	Ache	1	Ill	1	Pain	3	work	0	drug	0	Doctor	0
Migraine	2284453	https://www.fodors.com/community/air-travel/long-flight-migraine.cfm	2591	Migraine	27	Ache	14	Ill	7	Pain	1	work	4	drug	0	Doctor	9
Migraine	2276778	https://www.healingwell.com/community/?f=31	700	Migraine	3	Ache	0	Ill	0	Pain	0	work	0	drug	0	Doctor	0
Migraine	2008810	https://www.healthcentral.com/migraine/	463	Migraine	15	Ache	0	Ill	5	Pain	0	work	2	drug	0	Doctor	0
Migraine	2269583	https://www.healthcentral.com/migraine/c/123/177401/experience-spring-migraine	1089	Migraine	38	Ache	4	Ill	3	Pain	0	work	1	drug	0	Doctor	1
Migraine	2282363	https://www.healthfulchat.org/migraine-chat-room.html	1063	Migraine	25	Ache	8	Ill	1	Pain	2	work	7	drug	0	Doctor	0
Migraine	2275844	https://www.infantrisk.com/forum/forum/medications-and-breastfeeding-mothers/other-uncategorized-medications/152-migraine-medications	2082	Migraine	15	Ache	3	Ill	7	Pain	1	work	4	drug	18	Doctor	2
Migraine	2290390	https://www.inspire.com/groups/headaches-and-migraines/discussion/migraines-each-day-unbareable/	5301	Migraine	92	Ache	44	Ill	17	Pain	9	work	14	drug	22	Doctor	5
Migraine	2267892	https://www.medhelp.org/forums/Migraines--Headaches/show/123	1320	Migraine	9	Ache	17	Ill	0	Pain	6	work	2	drug	0	Doctor	5
Migraine	2274356	https://www.medicinenet.com/drug_abuse/article.htm	7155	Migraine	0	Ache	10	Ill	19	Pain	2	work	4	drug	221	Doctor	1
Migraine	2278343	https://www.medschat.com/Discuss/Migraine-Tension-Headache/	3875	Migraine	20	Ache	11	Ill	37	Pain	6	work	15	drug	13	Doctor	6
Migraine	1945768	https://www.mvertigo.org/	391	Migraine	1	Ache	0	Ill	1	Pain	0	work	0	drug	0	Doctor	0
Migraine	2267453	https://www.nomorepanic.co.uk/showthread.php?t=106093	1921	Migraine	16	Ache	0	Ill	5	Pain	5	work	3	drug	0	Doctor	1
Migraine	2290389	https://www.patientslikeme.com/conditions/135-migraine	3640	Migraine	175	Ache	20	Ill	1	Pain	21	work	0	drug	1	Doctor	0
Migraine	2282664	https://www.patientslikeme.com/patients	882	Migraine	0	Ache	0	Ill	0	Pain	0	work	0	drug	0	Doctor	0
Migraine	2291744	https://www.physicsforums.com/threads/the-four-stages-of-migraine.305792/	1946	Migraine	63	Ache	24	Ill	3	Pain	2	work	4	drug	0	Doctor	2
Migraine	2293898	https://www.tapatalk.com/groups/headachepainfreeforums/topic/766/Silent-Migraine	1566	Migraine	17	Ache	4	Ill	7	Pain	9	work	9	drug	2	Doctor	4
Migraine	1679473	https://www.tapatalk.com/groups/vets/topic/67841/Migraines?page=2	13151	Migraine	134	Ache	71	Ill	30	Pain	18	work	41	drug	1	Doctor	12
Migraine	2277773	https://www.topix.com/forum/health/migraine	1794	Migraine	24	Ache	5	Ill	4	Pain	3	work	0	drug	5	Doctor	2
Migraine	2264114	https://www.usmle-forums.com/usmle-step-1-forum/217482-migraine.html	660	Migraine	4	Ache	1	Ill	0	Pain	0	work	0	drug	0	Doctor	0
Migraine	2273781	https://www.webmd.com/anxiety-panic/default.htm	1074	Migraine	0	Ache	1	Ill	1	Pain	0	work	0	drug	2	Doctor	0
			244417		3400		1090		992		736		675		449		365

P69a	P69b	P70a	P70b	P71a	P71b	P72a	P72b	P73a	P73b	P74a	P74b	P75a	P75b	P76a	P76b	P77a	P77b	P78a	P78b	P79a	P79b	P80a	P80b
Mother	6	Serotonin	0	Disability	0	nasal	10	Cluster headache	0	pray	11	husband	12	travel	2	employ	3	Management	2	Walk	6	Neck pain	14
Mother	3	Serotonin	0	Disability	0	nasal	0	Cluster headache	0	pray	0	husband	0	travel	0	employ	0	Management	0	Walk	0	Neck pain	0
Mother	0	Serotonin	0	Disability	0	nasal	0	Cluster headache	0	pray	2	husband	0	travel	0	employ	0	Management	0	Walk	2	Neck pain	0
Mother	2	Serotonin	8	Disability	0	nasal	2	Cluster headache	1	pray	2	husband	1	travel	1	employ	0	Management	0	Walk	2	Neck pain	0
Mother	0	Serotonin	0	Disability	0	nasal	0	Cluster headache	0	pray	0	husband	0	travel	0	employ	0	Management	0	Walk	0	Neck pain	0
Mother	0	Serotonin	0	Disability	0	nasal	0	Cluster headache	0	pray	0	husband	0	travel	0	employ	0	Management	0	Walk	0	Neck pain	0
Mother	0	Serotonin	0	Disability	1	nasal	0	Cluster headache	0	pray	0	husband	0	travel	0	employ	0	Management	0	Walk	0	Neck pain	0
Mother	0	Serotonin	0	Disability	0	nasal	0	Cluster headache	0	pray	0	husband	0	travel	0	employ	0	Management	0	Walk	0	Neck pain	0
Mother	0	Serotonin	0	Disability	0	nasal	0	Cluster headache	0	pray	0	husband	0	travel	0	employ	0	Management	0	Walk	0	Neck pain	0
Mother	0	Serotonin	0	Disability	0	nasal	0	Cluster headache	0	pray	0	husband	0	travel	0	employ	0	Management	0	Walk	0	Neck pain	0
Mother	0	Serotonin	0	Disability	0	nasal	0	Cluster headache	0	pray	0	husband	0	travel	0	employ	0	Management	0	Walk	0	Neck pain	0
Mother	0	Serotonin	0	Disability	0	nasal	0	Cluster headache	1	pray	0	husband	0	travel	0	employ	0	Management	0	Walk	1	Neck pain	0
Mother	0	Serotonin	0	Disability	0	nasal	0	Cluster headache	0	pray	0	husband	0	travel	0	employ	0	Management	0	Walk	0	Neck pain	0
Mother	0	Serotonin	0	Disability	0	nasal	0	Cluster headache	3	pray	0	husband	0	travel	0	employ	0	Management	1	Walk	0	Neck pain	0
Mother	0	Serotonin	0	Disability	0	nasal	0	Cluster headache	0	pray	0	husband	0	travel	1	employ	0	Management	1	Walk	0	Neck pain	0
Mother	0	Serotonin	0	Disability	2	nasal	0	Cluster headache	0	pray	0	husband	0	travel	0	employ	0	Management	0	Walk	0	Neck pain	0
Mother	0	Serotonin	0	Disability	0	nasal	0	Cluster headache	1	pray	0	husband	0	travel	0	employ	0	Management	1	Walk	0	Neck pain	0
Mother	0	Serotonin	0	Disability	0	nasal	0	Cluster headache	1	pray	0	husband	0	travel	0	employ	0	Management	0	Walk	1	Neck pain	0
Mother	0	Serotonin	0	Disability	0	nasal	0	Cluster headache	0	pray	0	husband	0	travel	0	employ	0	Management	0	Walk	0	Neck pain	0
Mother	0	Serotonin	0	Disability	0	nasal	0	Cluster headache	0	pray	0	husband	0	travel	0	employ	0	Management	0	Walk	0	Neck pain	0
Mother	0	Serotonin	0	Disability	0	nasal	0	Cluster headache	0	pray	0	husband	0	travel	3	employ	0	Management	1	Walk	0	Neck pain	0
Mother	0	Serotonin	1	Disability	0	nasal	0	Cluster headache	0	pray	0	husband	0	travel	1	employ	0	Management	0	Walk	1	Neck pain	0
Mother	0	Serotonin	0	Disability	0	nasal	0	Cluster headache	0	pray	0	husband	0	travel	0	employ	0	Management	0	Walk	0	Neck pain	0
Mother	0	Serotonin	0	Disability	0	nasal	0	Cluster headache	1	pray	0	husband	0	travel	0	employ	0	Management	0	Walk	0	Neck pain	0
Mother	0	Serotonin	0	Disability	0	nasal	0	Cluster headache	0	pray	0	husband	0	travel	0	employ	0	Management	0	Walk	0	Neck pain	0
Mother	0	Serotonin	0	Disability	0	nasal	0	Cluster headache	0	pray	0	husband	0	travel	1	employ	0	Management	0	Walk	0	Neck pain	0
Mother	1	Serotonin	0	Disability	0	nasal	0	Cluster headache	0	pray	0	husband	0	travel	0	employ	0	Management	0	Walk	0	Neck pain	0
Mother	0	Serotonin	0	Disability	0	nasal	0	Cluster headache	0	pray	0	husband	0	travel	0	employ	0	Management	0	Walk	0	Neck pain	0
Mother	0	Serotonin	0	Disability	0	nasal	0	Cluster headache	10	pray	0	husband	0	travel	1	employ	0	Management	0	Walk	0	Neck pain	0
Mother	0	Serotonin	0	Disability	0	nasal	0	Cluster headache	0	pray	0	husband	0	travel	0	employ	0	Management	0	Walk	0	Neck pain	0
Mother	0	Serotonin	0	Disability	0	nasal	0	Cluster headache	0	pray	0	husband	0	travel	0	employ	0	Management	0	Walk	0	Neck pain	0
Mother	0	Serotonin	0	Disability	0	nasal	0	Cluster headache	0	pray	0	husband	0	travel	0	employ	0	Management	0	Walk	0	Neck pain	0
Mother	1	Serotonin	0	Disability	0	nasal	0	Cluster headache	0	pray	0	husband	0	travel	0	employ	0	Management	7	Walk	1	Neck pain	0
[illegible]																							

P139a	P139b	P140a	P140b	P141a	P141b	P142a	P142b	P143a	P143b	P144a	P144b	P145a	P145b	P146a	P146b	P147a	P147b	P148a	P148b	P149a	P149b	P150a	P150b	P151a	P151b	P152a	P152b	P153a	P153b	P154a	P154b
Adverse effect	0	faith	5	Intractable	2	Sensitivity to sound	3	Aggravate	1	Hemiplegic migraine	1	Calcium channel	1	Toxin	2	Incidence	4	Trigger point	5	Shock	4	Hurts	3	Characteristic	0	Prophylaxis	0	Future	0	Risk factor	0
Adverse effect	4	faith	0	Intractable	0	Sensitivity to sound	0	Aggravate	0	Hemiplegic migraine	0	Calcium channel	0	Toxin	0	Incidence	0	Trigger point	0	Shock	0	Hurts	0	Characteristic	0	Prophylaxis	0	Future	0	Risk factor	0
Adverse effect	0	faith	0	Intractable	0	Sensitivity to sound	0	Aggravate	0	Hemiplegic migraine	0	Calcium channel	0	Toxin	0	Incidence	0	Trigger point	0	Shock	0	Hurts	0	Characteristic	0	Prophylaxis	0	Future	0	Risk factor	0
Adverse effect	0	faith	0	Intractable	0	Sensitivity to sound	0	Aggravate	1	Hemiplegic migraine	0	Calcium channel	0	Toxin	0	Incidence	0	Trigger point	0	Shock	0	Hurts	0	Characteristic	0	Prophylaxis	0	Future	0	Risk factor	0
Adverse effect	0	faith	0	Intractable	0	Sensitivity to sound	0	Aggravate	0	Hemiplegic migraine	0	Calcium channel	0	Toxin	0	Incidence	0	Trigger point	0	Shock	0	Hurts	0	Characteristic	0	Prophylaxis	0	Future	0	Risk factor	0
Adverse effect	0	faith	0	Intractable	0	Sensitivity to sound	0	Aggravate	0	Hemiplegic migraine	0	Calcium channel	0	Toxin	0	Incidence	0	Trigger point	0	Shock	0	Hurts	0	Characteristic	0	Prophylaxis	0	Future	0	Risk factor	0
Adverse effect	0	faith	0	Intractable	0	Sensitivity to sound	0	Aggravate	0	Hemiplegic migraine	0	Calcium channel	0	Toxin	0	Incidence	0	Trigger point	0	Shock	0	Hurts	0	Characteristic	0	Prophylaxis	0	Future	0	Risk factor	0
Adverse effect	0	faith	0	Intractable	0	Sensitivity to sound	0	Aggravate	0	Hemiplegic migraine	0	Calcium channel	0	Toxin	0	Incidence	0	Trigger point	0	Shock	1	Hurts	0	Characteristic	0	Prophylaxis	0	Future	0	Risk factor	0
Adverse effect	0	faith	0	Intractable	0	Sensitivity to sound	0	Aggravate	0	Hemiplegic migraine	0	Calcium channel	0	Toxin	0	Incidence	0	Trigger point	0	Shock	0	Hurts	0	Characteristic	0	Prophylaxis	0	Future	0	Risk factor	0
Adverse effect	0	faith	0	Intractable	0	Sensitivity to sound	0	Aggravate	0	Hemiplegic migraine	0	Calcium channel	0	Toxin	0	Incidence	0	Trigger point	0	Shock	0	Hurts	0	Characteristic	0	Prophylaxis	0	Future	0	Risk factor	0
Adverse effect	0	faith	0	Intractable	0	Sensitivity to sound	0	Aggravate	0	Hemiplegic migraine	0	Calcium channel	0	Toxin	0	Incidence	0	Trigger point	0	Shock	0	Hurts	0	Characteristic	0	Prophylaxis	0	Future	0	Risk factor	0
Adverse effect	0	faith	0	Intractable	0	Sensitivity to sound	0	Aggravate	0	Hemiplegic migraine	0	Calcium channel	0	Toxin	0	Incidence	0	Trigger point	0	Shock	0	Hurts	0	Characteristic	0	Prophylaxis	0	Future	0	Risk factor	0
Adverse effect	0	faith	0	Intractable	0	Sensitivity to sound	0	Aggravate	0	Hemiplegic migraine	0	Calcium channel	0	Toxin	0	Incidence	0	Trigger point	0	Shock	0	Hurts	0	Characteristic	0	Prophylaxis	0	Future	0	Risk factor	0
Adverse effect	0	faith	0	Intractable	0	Sensitivity to sound	0	Aggravate	0	Hemiplegic migraine	1	Calcium channel	0	Toxin	0	Incidence	0	Trigger point	0	Shock	0	Hurts	0	Characteristic	0	Prophylaxis	0	Future	0	Risk factor	0
Adverse effect	0	faith	0	Intractable	0	Sensitivity to sound	0	Aggravate	0	Hemiplegic migraine	0	Calcium channel	0	Toxin	0	Incidence	0	Trigger point	0	Shock	0	Hurts	0	Characteristic	0	Prophylaxis	0	Future	0	Risk factor	1
Adverse effect	0	faith	0	Intractable	0	Sensitivity to sound	0	Aggravate	0	Hemiplegic migraine	0	Calcium channel	0	Toxin	0	Incidence	0	Trigger point	0	Shock	0	Hurts	0	Characteristic	0	Prophylaxis	0	Future	0	Risk factor	0
Adverse effect	0	faith	0	Intractable	0	Sensitivity to sound	0	Aggravate	0	Hemiplegic migraine	0	Calcium channel	0	Toxin	0	Incidence	0	Trigger point	0	Shock	0	Hurts	0	Characteristic	0	Prophylaxis	0	Future	0	Risk factor	0
Adverse effect	0	faith	0	Intractable	0	Sensitivity to sound	0	Aggravate	0	Hemiplegic migraine	0	Calcium channel	0	Toxin	0	Incidence	0	Trigger point	0	Shock	0	Hurts	2	Characteristic	0	Prophylaxis	0	Future	1	Risk factor	0
Adverse effect	0	faith	0	Intractable	0	Sensitivity to sound	0	Aggravate	0	Hemiplegic migraine	0	Calcium channel	0	Toxin	0	Incidence	0	Trigger point	0	Shock	0	Hurts	0	Characteristic	0	Prophylaxis	0	Future	0	Risk factor	0
Adverse effect	0	faith	0	Intractable	0	Sensitivity to sound	0	Aggravate	0	Hemiplegic migraine	0	Calcium channel	0	Toxin	0	Incidence	0	Trigger point	0	Shock	0	Hurts	0	Characteristic	0	Prophylaxis	0	Future	0	Risk factor	0
Adverse effect	0	faith	0	Intractable	0	Sensitivity to sound	0	Aggravate	0	Hemiplegic migraine	0	Calcium channel	0	Toxin	0	Incidence	0	Trigger point	0	Shock	0	Hurts	0	Characteristic	0	Prophylaxis	0	Future	0	Risk factor	0
Adverse effect	0	faith	0	Intractable	0	Sensitivity to sound	0	Aggravate	0	Hemiplegic migraine	0	Calcium channel	0	Toxin	0	Incidence	0	Trigger point	0	Shock	0	Hurts	0	Characteristic	0	Prophylaxis	0	Future	0	Risk factor	0
Adverse effect	0	faith	0	Intractable	0	Sensitivity to sound	0	Aggravate	0	Hemiplegic migraine	0	Calcium channel	0	Toxin	0	Incidence	0	Trigger point	0	Shock	0	Hurts	0	Characteristic	0	Prophylaxis	0	Future	0	Risk factor	0
Adverse effect	0	faith	0	Intractable	0	Sensitivity to sound	0	Aggravate	0	Hemiplegic migraine	0	Calcium channel	0	Toxin	0	Incidence	0	Trigger point	0	Shock	0	Hurts	0	Characteristic	0	Prophylaxis	0	Future	0	Risk factor	0
Adverse effect	0	faith	0	Intractable	0	Sensitivity to sound	0	Aggravate	0	Hemiplegic migraine	0	Calcium channel	0	Toxin	0	Incidence	0	Trigger point	0	Shock	0	Hurts	0	Characteristic	0	Prophylaxis	0	Future	0	Risk factor	0
Adverse effect	0	faith	0	Intractable	0	Sensitivity to sound	0	Aggravate	0	Hemiplegic migraine	0	Calcium channel	0	Toxin	0	Incidence	0	Trigger point	0	Shock	0	Hurts	0	Characteristic	0	Prophylaxis	0	Future	0	Risk factor	0
Adverse effect	0	faith	0	Intractable	0	Sensitivity to sound	0	Aggravate	0	Hemiplegic migraine	0	Calcium channel	0	Toxin	0	Incidence	0	Trigger point	0	Shock	0	Hurts	0	Characteristic	0	Prophylaxis	0	Future	0	Risk factor	0
Adverse effect	0	faith	0	Intractable	0	Sensitivity to sound	0	Aggravate	0	Hemiplegic migraine	0	Calcium channel	0	Toxin	0	Incidence	0	Trigger point	0	Shock	0	Hurts	0	Characteristic	0	Prophylaxis	0	Future	0	Risk factor	0
Adverse effect	0	faith	0	Intractable	0	Sensitivity to sound	0	Aggravate	0	Hemiplegic migraine	0	Calcium channel	0	Toxin	0	Incidence	0	Trigger point	0	Shock	0	Hurts	0	Characteristic	0	Prophylaxis	0	Future	0	Risk factor	0
Adverse effect	0	faith	0	Intractable	0	Sensitivity to sound	0	Aggravate	0	Hemiplegic migraine	0	Calcium channel	0	Toxin	0	Incidence	0	Trigger point	0	Shock	0	Hurts	0	Characteristic	0	Prophylaxis	0	Future	0	Risk factor	0
Adverse effect	0	faith	0	Intractable	0	Sensitivity to sound	0	Aggravate	0	Hemiplegic migraine	0	Calcium channel	0	Toxin	0	Incidence	0	Trigger point	0	Shock	0	Hurts	0	Characteristic	0	Prophylaxis	0	Future	3	Risk factor	0
Adverse effect	0	faith	0	Intractable	0	Sensitivity to sound	0	Aggravate	0	Hemiplegic migraine	0	Calcium channel	0	Toxin	0	Incidence	0	Trigger point	0	Shock	0	Hurts	0	Characteristic	0	Prophylaxis	0	Future	0	Risk factor	0
Adverse effect	0	faith	0	Intractable	1	Sensitivity to sound	0	Aggravate	0	Hemiplegic migraine	3	Calcium channel	0	Toxin	0	Incidence	0	Trigger point	0	Shock	0	Hurts	0	Characteristic	0	Prophylaxis	0	Future	0	Risk factor	0
Adverse effect	0	faith	0	Intractable	0	Sensitivity to sound	0	Aggravate	0	Hemiplegic migraine	0	Calcium channel	0	Toxin	0	Incidence	0	Trigger point	0	Shock	0	Hurts	0	Characteristic	0	Prophylaxis	0	Future	0	Risk factor	0
Adverse effect	0	faith	0	Intractable	0	Sensitivity to sound	0	Aggravate	0	Hemiplegic migraine	0	Calcium channel	0	Toxin	0	Incidence	0	Trigger point	0	Shock	0	Hurts	0	Characteristic	0	Prophylaxis	0	Future	1	Risk factor	0
Adverse effect	0	faith	0	Intractable	0	Sensitivity to sound	0	Aggravate	0	Hemiplegic migraine	0	Calcium channel	0	Toxin	0	Incidence	0	Trigger point	0	Shock	0	Hurts	0	Characteristic	0	Prophylaxis	0	Future	0	Risk factor	0
Adverse effect	0	faith	0	Intractable	0	Sensitivity to sound	0	Aggravate	0	Hemiplegic migraine	0	Calcium channel	1	Toxin	0	Incidence	0	Trigger point	0	Shock	0	Hurts	0	Characteristic	0	Prophylaxis	2	Future	0	Risk factor	0
Adverse effect	0	faith	0	Intractable	0	Sensitivity to sound	0	Aggravate	0	Hemiplegic migraine	0	Calcium channel	0	Toxin	0	Incidence	0	Trigger point	0	Shock	0	Hurts	0	Characteristic	0	Prophylaxis	0	Future	0	Risk factor	0
Adverse effect	0	faith	0	Intractable	0	Sensitivity to sound	0	Aggravate	0	Hemiplegic migraine	0	Calcium channel	0	Toxin	0	Incidence	0	Trigger point	0	Shock	0	Hurts	0	Characteristic	0	Prophylaxis	0	Future	0	Risk factor	0
Adverse effect	0	faith	0	Intractable	0	Sensitivity to sound	0	Aggravate	0	Hemiplegic migraine	0	Calcium channel	0	Toxin	1	Incidence	0	Trigger point	0	Shock	0	Hurts	0	Characteristic	0	Prophylaxis	0	Future	0	Risk factor	0
Adverse effect	0	faith	5	Intractable	2	Sensitivity to sound	3	Aggravate	1	Hemiplegic migraine	1	Calcium channel	1	Toxin	2	Incidence	4	Trigger point	5	Shock	4	Hurts	3	Characteristic	0	Prophylaxis	0	Future	0	Risk factor	0
Adverse effect	0	faith	0	Intractable	0	Sensitivity to sound	0	Aggravate	0	Hemiplegic migraine	0	Calcium channel	0	Toxin	0	Incidence	0	Trigger point	0	Shock	0	Hurts	0	Characteristic	0	Prophylaxis	0	Future	0	Risk factor	0
Adverse effect	0	faith	0	Intractable	0	Sensitivity to sound	0	Aggravate	0	Hemiplegic migraine	0	Calcium channel	0	Toxin	0	Incidence	0	Trigger point	0	Shock	0	Hurts	0	Characteristic	0	Prophylaxis	0	Future	0	Risk factor	0
Adverse effect	0	faith	0	Intractable	0	Sensitivity to sound	0	Aggravate	0	Hemiplegic migraine	0	Calcium channel	0	Toxin	0	Incidence	0	Trigger point	0	Shock	0	Hurts	1	Characteristic	0	Prophylaxis	0	Future	0	Risk factor	0
Adverse effect	0	faith	0	Intractable	0	Sensitivity to sound	0	Aggravate	0	Hemiplegic migraine	0	Calcium channel	0	Toxin	0	Incidence	0	Trigger point	0	Shock	0	Hurts	0	Characteristic	0	Prophylaxis	0	Future	0	Risk factor	0
Adverse effect	0	faith	0	Intractable	0	Sensitivity to sound	0	Aggravate	0	Hemiplegic migraine	0	Calcium channel	0	Toxin	1	Incidence	0	Trigger point	0	Shock	0	Hurts	0	Characteristic	0	Prophylaxis	0	Future	0	Risk factor	0
Adverse effect	0	faith	0	Intractable	0	Sensitivity to sound	0	Aggravate	0	Hemiplegic migraine	0	Calcium channel	0	Toxin	2	Incidence	0	Trigger point	0	Shock	0	Hurts	0	Characteristic	0	Prophylaxis	0	Future	2	Risk factor	0
Adverse effect	0	faith	0	Intractable	0	Sensitivity to sound	0	Aggravate	0	Hemiplegic migraine	0	Calcium channel	0	Toxin	0	Incidence	0	Trigger point	0	Shock	0	Hurts	0	Characteristic	0	Prophylaxis	0	Future	0	Risk factor	0
Adverse effect	0	faith	0	Intractable	0	Sensitivity to sound	0	Aggravate	1	Hemiplegic migraine	0	Calcium channel	0	Toxin	0	Incidence	0	Trigger point	0	Shock	0	Hurts	0	Characteristic	0	Prophylaxis	0	Future	1	Risk factor	0
Adverse effect	4	faith	0	Intractable	0	Sensitivity to sound	0	Aggravate	0	Hemiplegic migraine	0	Calcium channel	0	Toxin	0	Incidence	0	Trigger point	0	Shock	0	Hurts	0	Characteristic	0	Prophylaxis	0	Future	0	Risk factor	0
Adverse effect	0	faith	0	Intractable	0	Sensitivity to sound	0	Aggravate	1	Hemiplegic migraine	0	Calcium channel	0	Toxin	0	Incidence	0	Trigger point	0	Shock	0	Hurts	0	Characteristic	0	Prophylaxis	0	Future	0	Risk factor	0
Adverse effect	0	faith	0	Intractable	0	Sensitivity to sound	0	Aggravate	0	Hemiplegic migraine	0	Calcium channel	0	Toxin	0	Incidence	2	Trigger point	0	Shock	0	Hurts	0	Characteristic	0	Prophylaxis	0	Future	0	Risk factor	0
Adverse effect	0	faith	0	Intractable	0	Sensitivity to sound	0	Aggravate	0	Hemiplegic migraine	0	Calcium channel	0	Toxin	0	Incidence	0	Trigger point	0	Shock	0	Hurts	0	Characteristic	0	Prophylaxis	0	Future	0	Risk factor	0
Adverse effect	0	faith	0	Intractable	0	Sensitivity to sound	0	Aggravate	0	Hemiplegic migraine	0	Calcium channel	0	Toxin	0	Incidence	0	Trigger point	0	Shock	0	Hurts	0	Characteristic	0	Prophylaxis	0	Future	0	Risk factor	0
Adverse effect	0	faith	0	Intractable	0	Sensitivity to sound	0	Aggravate	0	Hemiplegic migraine	0	Calcium channel	0	Toxin	0	Incidence	0	Trigger point	0	Shock	0	Hurts	0	Characteristic	0	Prophylaxis	0	Future	0	Risk factor	0
Adverse effect	0	faith	0	Intractable	0	Sensitivity to sound	0	Aggravate	0	Hemiplegic migraine	0	Calcium channel	0	Toxin	0	Incidence	0	Trigger point	0	Shock	0	Hurts	0	Characteristic	0	Prophylaxis	0	Future	0	Risk factor	0
Adverse effect	0	faith	0	Intractable	0	Sensitivity to sound	0	Aggravate	0	Hemiplegic migraine	0	Calcium channel	0	Toxin	0	Incidence	0	Trigger point	0	Shock	0	Hurts	0	Characteristic	0	Prophylaxis	0	Future	0	Risk factor	0
Adverse effect	0	faith	0	Intractable	0	Sensitivity to sound	0	Aggravate	0	Hemiplegic migraine	0	Calcium channel	0	Toxin	0	Incidence	0	Trigger point	0	Shock	0	Hurts	0	Characteristic	0	Prophylaxis	0	Future	0	Risk factor	0
Adverse effect	0	faith	0	Intractable	0	Sensitivity to sound	0	Aggravate	0	Hemiplegic migraine	0	Calcium channel	0	Toxin	0	Incidence	0	Trigger point	0	Shock	0	Hurts	0	Characteristic	0	Prophylaxis	0	Future	0	Risk factor	0
Adverse effect	0	faith	0	Intractable	0	Sensitivity to sound	0	Aggravate	0	Hemiplegic migraine	0	Calcium channel	0	Toxin	0	Incidence	0	Trigger point	0	Shock	0	Hurts	0	Characteristic	0	Prophylaxis	0	Future	0	Risk factor	0
Adverse effect	0	faith	0	Intractable	0	Sensitivity to sound	0	Aggravate	0	Hemiplegic migraine	0	Calcium channel	0	Toxin	0	Incidence	0	Trigger point	0	Shock	0	Hurts	0	Characteristic	0	Prophylaxis	0	Future	0	Risk factor	0
Adverse effect	0	faith	0	Intractable	0	Sensitivity to sound	0	Aggravate	0	Hemiplegic migraine	0	Calcium channel	0	Toxin	0	Incidence	0	Trigger point	0	Shock	0	Hurts	0	Characteristic	0	Prophylaxis	0	Future	0	Risk factor	0
Adverse effect	0	faith	0	Intractable	0	Sensitivity to sound	0	Aggravate	3	Hemiplegic migraine	0	Calcium channel	0	Toxin	0	Incidence	0	Trigger point	0	Shock	0	Hurts	0	Characteristic	4	Prophylaxis	0	Future	0	Risk factor	0
Adverse effect	0	faith	0	Intractable	0	Sensitivity to sound	0	Aggravate	0	Hemiplegic migraine	0	Calcium channel	0	Toxin	0	Incidence	0	Trigger point	0	Shock	0	Hurts	0	Characteristic	0	Prophylaxis	0	Future	0	Risk factor	0
Adverse effect	0	faith	0	Intractable	0	Sensitivity to sound	0	Aggravate	0	Hemiplegic migraine	0	Calcium channel	0	Toxin	0	Incidence	0	Trigger point	0	Shock	0	Hurts	0	Characteristic	0	Prophylaxis	0	Future	0	Risk factor	0
Adverse effect	0	faith	0	Intractable	0	Sensitivity to sound	0	Aggravate	0	Hemiplegic migraine	0	Calcium channel	0	Toxin	0	Incidence	0	Trigger point	0	Shock	0	Hurts	0	Characteristic	0	Prophylaxis	0	Future	0	Risk factor	0
Adverse effect	0	faith	0	Intractable	0	Sensitivity to sound	0	Aggravate	0	Hemiplegic migraine	0	Calcium channel	0	Toxin	1	Incidence	0	Trigger point	0	Shock	0	Hurts	0	Characteristic	0	Prophylaxis	0	Future	0	Risk factor	0

[illegible]

P199b	P200a	P200b	P201a	P201b	P202a	P202b	P203a	P203b	P204a	P204b	P205a	P205b	P206a	P206b	P207a	P207b	P208a	P208b	P209a	P209b	P210a	P210b	P211a	P211b	P212a	P212b	P213a	P213b	P214a	P214b	P215a	P215b	P216a	P216b	P217a	P217b	P218a	P218b	P219a	P219b	P220a	P220b	P221a	P221b	P222a	P222b	P223a	P223b	P224a	P224b	P225a	P225b	P226a	P226b	P227a	P227b	P228a	P228b	P229a	P229b	P230a	P230b	P231a	P231b	P232a	P232b	P233a	P233b	P234a	P234b	P235a	P235b	P236a	P236b	P237a	P237b	P238a	P238b	P239a	P239b	P240a	P240b	P241a	P241b	P242a	P242b	P243a	P243b	P244a	P244b	P245a	P245b	P246a	P246b	P247a	P247b	P248a	P248b	P249a	P249b	P250a	P250b	P251a	P251b	P252a	P252b	P253a	P253b	P254a	P254b	P255a	P255b	P256a	P256b
0	Empower	0	Productivity	0	Caregiver	0	Nerve block	2	Accepting	2	Pain management	1	Mechanism	1	spouse	1	Screening	0	Co-morbid	0	Histamine	0	Tricyclic	0	Comorbid	0	Prevalence	0	Advantage	0	Peripheral	0	Dopamine	0	Assessment	0	Physical examination	0	Referral	0	Attitude	0	Gene-related	0	Calcitonin	0	Beta-blocker	0	church	0	Temporal	1	Barbiturate	1	Lidocaine	1	Spectrum	1	Motivation	1	Menstrual migraine	1	Correlate	0	Outpatient	0	Injury	0	Lab test	0	Efficacy	0	Antihistamine	0	Lactation	0	Secondary headache	0	Trigeminal autonomic cephalalgia	0	Progression	0	Medication overuse	0	Inpatient treatment	0	Intracranial hypertension	0	Botulinum Toxin	0	Serotonin receptor	0	Mindfulness	0	Cranial nerve	0	Episodic migraine	0	Double vision	0	Vestibular migraine	0	surgical	0	Surgical treatment	0	5-HT	0	Ineffective	0	Eye symptom	0	Female hormone	0
0	Empower	0	Productivity	0	Caregiver	0	Nerve block	0	Accepting	0	Pain management	0	Mechanism	1	spouse	0	Screening	0	Co-morbid	0	Histamine	0	Tricyclic	0	Comorbid	0	Prevalence	0	Advantage	0	Peripheral	0	Dopamine	0	Assessment	0	Physical examination	0	Referral	0	Attitude	0	Gene-related	0	Calcitonin	0	Beta-blocker	0	church	0	Temporal	0	Barbiturate	0	Lidocaine	0	Spectrum	0	Motivation	0	Menstrual migraine	0	Correlate	0	Outpatient	0	Injury	0	Lab test	0	Efficacy	0	Antihistamine	0	Lactation	0	Secondary headache	0	Trigeminal autonomic cephalalgia	0	Progression	0	Medication overuse	0	Inpatient treatment	0	Intracranial hypertension	0	Botulinum Toxin	0	Serotonin receptor	0	Mindfulness	0	Cranial nerve	0	Episodic migraine	0	Double vision	0	Vestibular migraine	0	surgical	0	Surgical treatment	0	5-HT	0	Ineffective	0	Eye symptom	0	Female hormone	1
0	Empower	0	Productivity	0	Caregiver	0	Nerve block	0	Accepting	0	Pain management	0	Mechanism	0	spouse	0	Screening	0	Co-morbid	0	Histamine	0	Tricyclic	0	Comorbid	0	Prevalence	0	Advantage	0	Peripheral	0	Dopamine	0	Assessment	0	Physical examination	0	Referral	0	Attitude	0	Gene-related	0	Calcitonin	0	Beta-blocker	0	church	0	Temporal	0	Barbiturate	0	Lidocaine	0	Spectrum	0	Motivation	0	Menstrual migraine	0	Correlate	0	Outpatient	0	Injury	0	Lab test	0	Efficacy	0	Antihistamine	0	Lactation	0	Secondary headache	0	Trigeminal autonomic cephalalgia	0	Progression	0	Medication overuse	0	Inpatient treatment	0	Intracranial hypertension	0	Botulinum Toxin	0	Serotonin receptor	0	Mindfulness	0	Cranial nerve	0	Episodic migraine	0	Double vision	0	Vestibular migraine	0	surgical	0	Surgical treatment	0	5-HT	0	Ineffective	0	Eye symptom	0	Female hormone	0
0	Empower	0	Productivity	0	Caregiver	0	Nerve block	0	Accepting	0	Pain management	0	Mechanism	0	spouse	0	Screening	0	Co-morbid	0	Histamine	0	Tricyclic	0	Comorbid	0	Prevalence	0	Advantage	0	Peripheral	0	Dopamine	0	Assessment	0	Physical examination	0	Referral	0	Attitude	0	Gene-related	0	Calcitonin	0	Beta-blocker	0	church	1	Temporal	0	Barbiturate	0	Lidocaine	0	Spectrum	0	Motivation	0	Menstrual migraine	0	Correlate	0	Outpatient	0	Injury	0	Lab test	0	Efficacy	0	Antihistamine	0	Lactation	0	Secondary headache	0	Trigeminal autonomic cephalalgia	0	Progression	0	Medication overuse	0	Inpatient treatment	0	Intracranial hypertension	0	Botulinum Toxin	0	Serotonin receptor	0	Mindfulness	0	Cranial nerve	0	Episodic migraine	0	Double vision	0	Vestibular migraine	0	surgical	0	Surgical treatment	0	5-HT	1	Ineffective	1	Eye symptom	1	Female hormone	0
0	Empower	0	Productivity	0	Caregiver	0	Nerve block	0	Accepting	0	Pain management	0	Mechanism	0	spouse	0	Screening	0	Co-morbid	0	Histamine	0	Tricyclic	0	Comorbid	0	Prevalence	0	Advantage	0	Peripheral	0	Dopamine	0	Assessment	0	Physical examination	0	Referral	0	Attitude	0	Gene-related	0	Calcitonin	0	Beta-blocker	0	church	0	Temporal	0	Barbiturate	0	Lidocaine	0	Spectrum	0	Motivation	0	Menstrual migraine	0	Correlate	0	Outpatient	0	Injury	0	Lab test	0	Efficacy	0	Antihistamine	0	Lactation	0	Secondary headache	0	Trigeminal autonomic cephalalgia	0	Progression	0	Medication overuse	0	Inpatient treatment	0	Intracranial hypertension	0	Botulinum Toxin	0	Serotonin receptor	0	Mindfulness	0	Cranial nerve	0	Episodic migraine	0	Double vision	0	Vestibular migraine	0	surgical	0	Surgical treatment	0	5-HT	0	Ineffective	0	Eye symptom	0	Female hormone	0
0	Empower	0	Productivity	0	Caregiver	0	Nerve block	0	Accepting	0	Pain management	0	Mechanism	0	spouse	0	Screening	0	Co-morbid	0	Histamine	0	Tricyclic	0	Comorbid	0	Prevalence	0	Advantage	0	Peripheral	0	Dopamine	0	Assessment	0	Physical examination	0	Referral	0	Attitude	0	Gene-related	0	Calcitonin	0	Beta-blocker	0	church	0	Temporal	0	Barbiturate	0	Lidocaine	0	Spectrum	0	Motivation	0	Menstrual migraine	0	Correlate	0	Outpatient	0	Injury	0	Lab test	0	Efficacy	0	Antihistamine	0	Lactation	0	Secondary headache	0	Trigeminal autonomic cephalalgia	0	Progression	0	Medication overuse	0	Inpatient treatment	0	Intracranial hypertension	0	Botulinum Toxin	0	Serotonin receptor	0	Mindfulness	0	Cranial nerve	0	Episodic migraine	0	Double vision	0	Vestibular migraine	0	surgical	0	Surgical treatment	0	5-HT	0	Ineffective	0	Eye symptom	0	Female hormone	0
0	Empower	0	Productivity	0	Caregiver	0	Nerve block	0	Accepting	0	Pain management	0	Mechanism	0	spouse	0	Screening	0	Co-morbid	0	Histamine	0	Tricyclic	0	Comorbid	0	Prevalence	0	Advantage	0	Peripheral	0	Dopamine	0	Assessment	0	Physical examination	0	Referral	0	Attitude	0	Gene-related	0	Calcitonin	0	Beta-blocker	0	church	0	Temporal	0	Barbiturate	0	Lidocaine	0	Spectrum	0	Motivation	0	Menstrual migraine	0	Correlate	0	Outpatient	0	Injury	0	Lab test	0	Efficacy	0	Antihistamine	0	Lactation	0	Secondary headache	0	Trigeminal autonomic cephalalgia	0	Progression	0	Medication overuse	0	Inpatient treatment	0	Intracranial hypertension	0	Botulinum Toxin	0	Serotonin receptor	0	Mindfulness	0	Cranial nerve	0	Episodic migraine	0	Double vision	0	Vestibular migraine	0	surgical	0	Surgical treatment	0	5-HT	0	Ineffective	0	Eye symptom	0	Female hormone	0
0	Empower	0	Productivity	0	Caregiver	0	Nerve block	0	Accepting	0	Pain management	0	Mechanism	0	spouse	0	Screening	0	Co-morbid	0	Histamine	0	Tricyclic	0	Comorbid	0	Prevalence	0	Advantage	0	Peripheral	0	Dopamine	0	Assessment	0	Physical examination	0	Referral	0	Attitude	0	Gene-related	0	Calcitonin	0	Beta-blocker	0	church	0	Temporal	0	Barbiturate	0	Lidocaine	0	Spectrum	0	Motivation	0	Menstrual migraine	0	Correlate	0	Outpatient	0	Injury	0	Lab test	0	Efficacy	0	Antihistamine	0	Lactation	0	Secondary headache	0	Trigeminal autonomic cephalalgia	0	Progression	0	Medication overuse	0	Inpatient treatment	0	Intracranial hypertension	0	Botulinum Toxin	0	Serotonin receptor	0	Mindfulness	0	Cranial nerve	0	Episodic migraine	0	Double vision	0	Vestibular migraine	0	surgical	0	Surgical treatment	0	5-HT	0	Ineffective	0	Eye symptom	0	Female hormone	0
0	Empower	0	Productivity	0	Caregiver	0	Nerve block	0	Accepting	0	Pain management	0	Mechanism	0	spouse	0	Screening	0	Co-morbid	0	Histamine	0	Tricyclic	0	Comorbid	0	Prevalence	0	Advantage	0	Peripheral	0	Dopamine	0	Assessment	0	Physical examination	0	Referral	0	Attitude	0	Gene-related	0	Calcitonin	0	Beta-blocker	0	church	0	Temporal	0	Barbiturate	0	Lidocaine	0	Spectrum	0	Motivation	0	Menstrual migraine	0	Correlate	0	Outpatient	0	Injury	0	Lab test	0	Efficacy	0	Antihistamine	0	Lactation	0	Secondary headache	0	Trigeminal autonomic cephalalgia	0	Progression	0	Medication overuse	0	Inpatient treatment	0	Intracranial hypertension	0	Botulinum Toxin	0	Serotonin receptor	0	Mindfulness	0	Cranial nerve	0	Episodic migraine	0	Double vision	0	Vestibular migraine	0	surgical	0	Surgical treatment	0	5-HT	0	Ineffective	0	Eye symptom	0	Female hormone	0
0	Empower	0	Productivity	0	Caregiver	0	Nerve block	0	Accepting	0	Pain management	0	Mechanism	0	spouse	0	Screening	0	Co-morbid	0	Histamine	0	Tricyclic	0	Comorbid	0	Prevalence	0	Advantage	0	Peripheral	0	Dopamine	0	Assessment	0	Physical examination	0	Referral	0	Attitude	0	Gene-related	0	Calcitonin	0	Beta-blocker	0	church	0	Temporal	0	Barbiturate	0	Lidocaine	0	Spectrum	0	Motivation	0	Menstrual migraine	0	Correlate	0	Outpatient	0	Injury	0	Lab test	0	Efficacy	0	Antihistamine	0	Lactation	0	Secondary headache	0	Trigeminal autonomic cephalalgia	0	Progression	0	Medication overuse	0	Inpatient treatment	0	Intracranial hypertension	0	Botulinum Toxin	0	Serotonin receptor	0	Mindfulness	0	Cranial nerve	0	Episodic migraine	0	Double vision	0	Vestibular migraine	0	surgical	0	Surgical treatment	0	5-HT	0	Ineffective	0	Eye symptom	0	Female hormone	0
0	Empower	0	Productivity	0	Caregiver	0	Nerve block	0	Accepting	0	Pain management	0	Mechanism	0	spouse	0	Screening	0	Co-morbid	0	Histamine	0	Tricyclic	0	Comorbid	0	Prevalence	0	Advantage	0	Peripheral	0	Dopamine	0	Assessment	0	Physical examination	0	Referral	0	Attitude	0	Gene-related	0	Calcitonin	0	Beta-blocker	0	church	0	Temporal	0	Barbiturate	0	Lidocaine	0	Spectrum	0	Motivation	0	Menstrual migraine	0	Correlate	0	Outpatient	0	Injury	0	Lab test	0	Efficacy	0	Antihistamine	0	Lactation	0	Secondary headache	0	Trigeminal autonomic cephalalgia	0	Progression	0	Medication overuse	0	Inpatient treatment	0	Intracranial hypertension	0	Botulinum Toxin	0	Serotonin receptor	0	Mindfulness	0	Cranial nerve	0	Episodic migraine	0	Double vision	0	Vestibular migraine	0	surgical	0	Surgical treatment	0	5-HT	0	Ineffective	0	Eye symptom	0	Female hormone	0
0	Empower	0	Productivity	0	Caregiver	0	Nerve block	0	Accepting	0	Pain management	0	Mechanism	0	spouse	0	Screening	0	Co-morbid	0	Histamine	0	Tricyclic	0	Comorbid	0	Prevalence	0	Advantage	0	Peripheral	0	Dopamine	0	Assessment	0	Physical examination	0	Referral	0	Attitude	0	Gene-related	0	Calcitonin	0	Beta-blocker	0	church	0	Temporal	0	Barbiturate	0	Lidocaine	1	Spectrum	0	Motivation	0	Menstrual migraine	0	Correlate	0	Outpatient	0	Injury	0	Lab test	0	Efficacy	0	Antihistamine	0	Lactation	0	Secondary headache	0	Trigeminal autonomic cephalalgia	0	Progression	0	Medication overuse	0	Inpatient treatment	0	Intracranial hypertension	0	Botulinum Toxin	0	Serotonin receptor	0	Mindfulness	0	Cranial nerve	0	Episodic migraine	0	Double vision	0	Vestibular migraine	0	surgical	0	Surgical treatment	0	5-HT	0	Ineffective	0	Eye symptom	0	Female hormone	0
0	Empower	0	Productivity	0	Caregiver	0	Nerve block	0	Accepting	0	Pain management	0	Mechanism	0	spouse	0	Screening	0	Co-morbid	0	Histamine	0	Tricyclic	0	Comorbid	0	Prevalence	0	Advantage	0	Peripheral	0	Dopamine	0	Assessment	0	Physical examination	0	Referral	0	Attitude	0	Gene-related	0	Calcitonin	0	Beta-blocker	0	church	0	Temporal	0	Barbiturate	0	Lidocaine	0	Spectrum	0	Motivation	0	Menstrual migraine	0	Correlate	0	Outpatient	0	Injury	0	Lab test	0	Efficacy	0	Antihistamine	0	Lactation	0	Secondary headache	0	Trigeminal autonomic cephalalgia	0	Progression	0	Medication overuse	0	Inpatient treatment	0	Intracranial hypertension	0	Botulinum Toxin	0	Serotonin receptor	0	Mindfulness	0	Cranial nerve	0	Episodic migraine	0	Double vision	0	Vestibular migraine	0	surgical	0	Surgical treatment	0	5-HT	0	Ineffective	0	Eye symptom	0	Female hormone	0
0	Empower	0	Productivity	0	Caregiver	1	Nerve block	0	Accepting	0	Pain management	0	Mechanism	0	spouse	0	Screening	0	Co-morbid	0	Histamine	0	Tricyclic	0	Comorbid	0	Prevalence	0	Advantage	0	Peripheral	0	Dopamine	0	Assessment	0	Physical examination	0	Referral	0	Attitude	0	Gene-related	0	Calcitonin	0	Beta-blocker	0	church	0	Temporal	0	Barbiturate	0	Lidocaine	0	Spectrum	0	Motivation	0	Menstrual migraine	0	Correlate	0	Outpatient	0	Injury	0	Lab test	0	Efficacy	0	Antihistamine	0	Lactation	0	Secondary headache	0	Trigeminal autonomic cephalalgia	0	Progression	0	Medication overuse	0	Inpatient treatment	0	Intracranial hypertension	0	Botulinum Toxin	0	Serotonin receptor	0	Mindfulness	0	Cranial nerve	0	Episodic migraine	0	Double vision	0	Vestibular migraine	0	surgical	0	Surgical treatment	0	5-HT	0	Ineffective	0	Eye symptom	0	Female hormone	0
0	Empower	0	Productivity	0	Caregiver	0	Nerve block	0	Accepting	0	Pain management	0	Mechanism	0	spouse	0	Screening	0	Co-morbid	0	Histamine	0	Tricyclic	0	Comorbid	0	Prevalence	0	Advantage	0	Peripheral	0	Dopamine	0	Assessment	0	Physical examination	0	Referral	0	Attitude	0	Gene-related	0	Calcitonin	0	Beta-blocker	0	church	0	Temporal	0	Barbiturate	0	Lidocaine	0	Spectrum	0	Motivation	0	Menstrual migraine	0	Correlate	0	Outpatient	0	Injury	0	Lab test	0	Efficacy	0	Antihistamine	0	Lactation	0	Secondary headache	0	Trigeminal autonomic cephalalgia	0	Progression	0	Medication overuse	0	Inpatient treatment	0	Intracranial hypertension	0	Botulinum Toxin	0	Serotonin receptor	0	Mindfulness	0	Cranial nerve	0	Episodic migraine	0	Double vision	0	Vestibular migraine	0	surgical	1	Surgical treatment	1	5-HT	0	Ineffective	0	Eye symptom	0	Female hormone	0
0	Empower	0	Productivity	0	Caregiver	0	Nerve block	0	Accepting	0	Pain management	0	Mechanism	0	spouse	0	Screening	0	Co-morbid	0	Histamine	0	Tricyclic	0	Comorbid	0	Prevalence	0	Advantage	0	Peripheral	0	Dopamine	0	Assessment	0	Physical examination	0	Referral	0	Attitude	0	Gene-related	0	Calcitonin	0	Beta-blocker	0	church	0	Temporal	0	Barbiturate	0	Lidocaine	0	Spectrum	0	Motivation	0	Menstrual migraine	0	Correlate	0	Outpatient	0	Injury	0	Lab test	0	Efficacy	0	Antihistamine	0	Lactation	0	Secondary headache	0	Trigeminal autonomic cephalalgia	0	Progression	0	Medication overuse	0	Inpatient treatment	0	Intracranial hypertension	0	Botulinum Toxin	0	Serotonin receptor	0	Mindfulness	0	Cranial nerve	0	Episodic migraine	0	Double vision	0	Vestibular migraine	0	surgical	0	Surgical treatment	0	5-HT	0	Ineffective	0	Eye symptom	0	Female hormone	0
0	Empower	0	Productivity	0	Caregiver	0	Nerve block	0	Accepting	0	Pain management	0	Mechanism	0	spouse	0	Screening	0	Co-morbid	0	Histamine	0	Tricyclic	0	Comorbid	0	Prevalence	0	Advantage	0	Peripheral	0	Dopamine	0	Assessment	0	Physical examination	0	Referral	0	Attitude	0	Gene-related	0	Calcitonin	0	Beta-blocker	0	church	0	Temporal	0	Barbiturate	0	Lidocaine	0	Spectrum	0	Motivation	0	Menstrual migraine	0	Correlate	0	Outpatient	0	Injury	0	Lab test	0	Efficacy	0	Antihistamine	0	Lactation	0	Secondary headache	0	Trigeminal autonomic cephalalgia	0	Progression	0	Medication overuse	0	Inpatient treatment	0	Intracranial hypertension	0	Botulinum Toxin	0	Serotonin receptor	0	Mindfulness	0	Cranial nerve	0	Episodic migraine	0	Double vision	0	Vestibular migraine	0	surgical	0	Surgical treatment	0	5-HT	0	Ineffective	0	Eye symptom	0	Female hormone	0
0	Empower	0	Productivity	0	Caregiver	0	Nerve block	0	Accepting	0	Pain management	0	Mechanism	0	spouse	0	Screening	0	Co-morbid	0	Histamine	0	Tricyclic	0	Comorbid	0	Prevalence	0	Advantage	0	Peripheral	0	Dopamine	0	Assessment	0	Physical examination	0	Referral	0	Attitude	0	Gene-related	0	Calcitonin	0	Beta-blocker	1	church	0	Temporal	0	Barbiturate	0	Lidocaine	0	Spectrum	0	Motivation	0	Menstrual migraine	0	Correlate	0	Outpatient	0	Injury	0	Lab test	0	Efficacy	0	Antihistamine	0	Lactation	0	Secondary headache	0	Trigeminal autonomic cephalalgia	0	Progression	0	Medication overuse	0	Inpatient treatment	0	Intracranial hypertension	0	Botulinum Toxin	0	Serotonin receptor	0	Mindfulness	0	Cranial nerve	0	Episodic migraine	0	Double vision	0	Vestibular migraine	0	surgical	0	Surgical treatment	0	5-HT	0	Ineffective	0	Eye symptom	0	Female hormone	0
0	Empower	0	Productivity	0	Caregiver	0	Nerve block	0	Accepting	0	Pain management	0	Mechanism	0	spouse	0	Screening	0	Co-morbid	0	Histamine	0	Tricyclic	0	Comorbid	0	Prevalence	0	Advantage	0	Peripheral	0	Dopamine	0	Assessment	0	Physical examination	0	Referral	0	Attitude	0	Gene-related	0	Calcitonin	0	Beta-blocker	0	church	0	Temporal	0	Barbiturate	0	Lidocaine	0	Spectrum	0	Motivation	0	Menstrual migraine	0	Correlate	0	Outpatient	0	Injury	0	Lab test	0	Efficacy	0	Antihistamine	0	Lactation	0	Secondary headache	0	Trigeminal autonomic cephalalgia	0	Progression	0	Medication overuse	0	Inpatient treatment	0	Intracranial hypertension	0	Botulinum Toxin	0	Serotonin receptor	0	Mindfulness	0	Cranial nerve	0	Episodic migraine	0	Double vision	0	Vestibular migraine	0	surgical	0	Surgical treatment	0	5-HT	0	Ineffective	0	Eye symptom	0	Female hormone	0
0	Empower	0	Productivity	0	Caregiver	0	Nerve block	0	Accepting	0	Pain management	0	Mechanism	0	spouse	0	Screening	0	Co-morbid	0	Histamine	0	Tricyclic	0	Comorbid	0	Prevalence	0	Advantage	0	Peripheral	0	Dopamine	0	Assessment	0	Physical examination	0	Referral	0	Attitude	0	Gene-related	0	Calcitonin	0	Beta-blocker	0	church	0	Temporal	0	Barbiturate	0	Lidocaine	0	Spectrum	0	Motivation	0	Menstrual migraine	0	Correlate	0	Outpatient	0	Injury	0	Lab test	0	Efficacy	0	Antihistamine	0	Lactation	0	Secondary headache	0	Trigeminal autonomic cephalalgia	0	Progression	0	Medication overuse	0	Inpatient treatment	0	Intracranial hypertension	0	Botulinum Toxin	0	Serotonin receptor	0	Mindfulness	0	Cranial nerve	0	Episodic migraine	0	Double vision	0	Vestibular migraine	0	surgical	0	Surgical treatment	0	5-HT	0	Ineffective	0	Eye symptom	0	Female hormone	0
0	Empower	0	Productivity	0	Caregiver	0	Nerve block	0	Accepting	0	Pain management	0	Mechanism	0	spouse	0	Screening	0	Co-morbid	0	Histamine	0	Tricyclic	0	Comorbid	0	Prevalence	0	Advantage	0	Peripheral	0	Dopamine	0	Assessment	0	Physical examination	0	Referral	0	Attitude	0	Gene-related	0	Calcitonin	0	Beta-blocker	0	church	0	Temporal	0	Barbiturate	0	Lidocaine	0	Spectrum	0	Motivation	0	Menstrual migraine	0	Correlate	0	Outpatient	0	Injury	0	Lab test	0	Efficacy	0	Antihistamine	0	Lactation	0	Secondary headache	0	Trigeminal autonomic cephalalgia	0	Progression	0	Medication overuse	0	Inpatient treatment	0	Intracranial hypertension	0	Botulinum Toxin	0	Serotonin receptor	0	Mindfulness	0	Cranial nerve	0	Episodic migraine	0	Double vision	0	Vestibular migraine	0	surgical	0	Surgical treatment	0	5-HT	0	Ineffective	0	Eye symptom	0	Female hormone	0
0	Empower	0	Productivity	1	Caregiver	1	Nerve block	0	Accepting	0	Pain management	0	Mechanism	0	spouse	0	Screening	0	Co-morbid	0	Histamine	0	Tricyclic	0	Comorbid	0	Prevalence	0	Advantage	0	Peripheral	0	Dopamine	0	Assessment	0	Physical examination	0	Referral	0	Attitude	0	Gene-related	1	Calcitonin	1	Beta-blocker	0	church	0	Temporal	0	Barbiturate	0	Lidocaine	0	Spectrum	0	Motivation	0	Menstrual migraine	0	Correlate	0	Outpatient	0	Injury	0	Lab test	0	Efficacy	0	Antihistamine	0	Lactation	0	Secondary headache	0	Trigeminal autonomic cephalalgia	0	Progression	0	Medication overuse	0	Inpatient treatment	0	Intracranial hypertension	0	Botulinum Toxin	0	Serotonin receptor	0	Mindfulness	0	Cranial nerve	0	Episodic migraine	0	Double vision	0	Vestibular migraine	0	surgical	0	Surgical treatment	0	5-HT	0	Ineffective	0	Eye symptom	0	Female hormone	0
0	Empower	0	Productivity	0	Caregiver	0	Nerve block	0	Accepting	0	Pain management	0	Mechanism	0	spouse	0	Screening	0	Co-morbid	0	Histamine	0	Tricyclic	0	Comorbid	0	Prevalence	0	Advantage	0	Peripheral	0	Dopamine	0	Assessment	0	Physical examination	0	Referral	0	Attitude	0	Gene-related	0	Calcitonin	0	Beta-blocker	0	church	0	Temporal	0	Barbiturate	0	Lidocaine	0	Spectrum	0	Motivation	0	Menstrual migraine	0	Correlate	0	Outpatient	0	Injury	0	Lab test	0	Efficacy	0	Antihistamine	0	Lactation	0	Secondary headache	0	Trigeminal autonomic cephalalgia	0	Progression	0	Medication overuse	0	Inpatient treatment	0	Intracranial hypertension	0	Botulinum Toxin	0	Serotonin receptor	0	Mindfulness	0	Cranial nerve	0	Episodic migraine	0	Double vision	0	Vestibular migraine	0	surgical	0	Surgical treatment	0	5-HT	0	Ineffective	0	Eye symptom	0	Female hormone	0
0	Empower	0	Productivity	0	Caregiver	0	Nerve block	0	Accepting	0	Pain management	0	Mechanism	0	spouse	0	Screening	0	Co-morbid	0	Histamine	0	Tricyclic	0	Comorbid	0	Prevalence	0	Advantage	0	Peripheral	0	Dopamine	0	Assessment	0	Physical examination	0	Referral	0	Attitude	0	Gene-related	0	Calcitonin	0	Beta-blocker	0	church	0	Temporal	0	Barbiturate	0	Lidocaine	0	Spectrum	0	Motivation	0	Menstrual migraine	0	Correlate	0	Outpatient	0	Injury	0	Lab test	0	Efficacy	0	Antihistamine	0	Lactation	0	Secondary headache	0	Trigeminal autonomic cephalalgia	0	Progression	0	Medication overuse	0	Inpatient treatment	0	Intracranial hypertension	0	Botulinum Toxin	0	Serotonin receptor	0	Mindfulness	0	Cranial nerve	0	Episodic migraine	0	Double vision	0	Vestibular migraine	0	surgical	0	Surgical treatment	0	5-HT	0	Ineffective	0	Eye symptom	0	Female hormone	0
0	Empower	0	Productivity	0	Caregiver	0	Nerve block	0	Accepting	0	Pain management	0	Mechanism	0	spouse	0	Screening	0	Co-morbid	0	Histamine	0	Tricyclic	0	Comorbid	0	Prevalence	0	Advantage	0	Peripheral	0	Dopamine	0	Assessment	0	Physical examination	0	Referral	0	Attitude	0	Gene-related	0	Calcitonin	0	Beta-blocker	0	church	0	Temporal	0	Barbiturate	0	Lidocaine	0	Spectrum	0	Motivation	0	Menstrual migraine	0	Correlate	0	Outpatient	0	Injury	0	Lab test	0	Efficacy	0	Antihistamine	0	Lactation	0	Secondary headache	0	Trigeminal autonomic cephalalgia	0	Progression	0	Medication overuse	0	Inpatient treatment	0	Intracranial hypertension	0	Botulinum Toxin	0	Serotonin receptor	0	Mindfulness	0	Cranial nerve	0	Episodic migraine	0	Double vision	0	Vestibular migraine	0	surgical	0	Surgical treatment	0	5-HT	0	Ineffective	0	Eye symptom	0	Female hormone	0
0	Empower	0	Productivity	0	Caregiver	0	Nerve block	0	Accepting	0	Pain management	0	Mechanism	0	spouse	0	Screening	0	Co-morbid	0	Histamine	0	Tricyclic	0	Comorbid	0	Prevalence	0	Advantage	0	Peripheral	0	Dopamine	0	Assessment	0	Physical examination	0	Referral	0	Attitude	0	Gene-related	0	Calcitonin	0	Beta-blocker	0	church	0	Temporal	0	Barbiturate	0	Lidocaine	0	Spectrum	0	Motivation	0	Menstrual migraine	0	Correlate	0	Outpatient	0	Injury	0	Lab test	0	Efficacy	0	Antihistamine	0	Lactation	0	Secondary headache	0	Trigeminal autonomic cephalalgia	0	Progression	0	Medication overuse	0	Inpatient treatment	0	Intracranial hypertension	0	Botulinum Toxin	0	Serotonin receptor	0	Mindfulness	0	Cranial nerve	0	Episodic migraine	0	Double vision	0	Vestibular migraine	2	surgical	0	Surgical treatment	0	5-HT	0	Ineffective	0	Eye symptom	0	Female hormone	0
0	Empower	0	Productivity	0	Caregiver	0	Nerve block	0	Accepting	0	Pain management	0	Mechanism	0	spouse	0	Screening	0	Co-morbid	0	Histamine	0	Tricyclic	0	Comorbid	0	Prevalence	0	Advantage	0	Peripheral	0	Dopamine	0	Assessment	0	Physical examination	0	Referral	0	Attitude	0	Gene-related	0	Calcitonin	0	Beta-blocker	0	church	0	Temporal	0	Barbiturate	0	Lidocaine	0	Spectrum	0	Motivation	0	Menstrual migraine	0	Correlate	0	Outpatient	0	Injury	0	Lab test	0	Efficacy	0	Antihistamine	0	Lactation	0	Secondary headache	0	Trigeminal autonomic cephalalgia	0	Progression	0	Medication overuse	0	Inpatient treatment	0	Intracranial hypertension	0	Botulinum Toxin	0	Serotonin receptor	0	Mindfulness	0	Cranial nerve	0	Episodic migraine	0	Double vision	0	Vestibular migraine	0	surgical	0	Surgical treatment	0	5-HT	0	Ineffective	0	Eye symptom	0	Female hormone	0
0	Empower	0	Productivity	0	Caregiver	0	Nerve block	0	Accepting	0	Pain management	0	Mechanism	0	spouse	0	Screening	0	Co-morbid	0	Histamine	0	Tricyclic	0	Comorbid	0	Prevalence	0	Advantage	0	Peripheral	0	Dopamine	0	Assessment	0	Physical examination	0	Referral	0	Attitude	0	Gene-related	0	Calcitonin	0	Beta-blocker	1	church	0	Temporal	0	Barbiturate	0	Lidocaine	0	Spectrum	0	Motivation	0	Menstrual migraine	0	Correlate	0	Outpatient	0	Injury	0	Lab test	0	Efficacy	0	Antihistamine	0	Lactation	0	Secondary headache	0	Trigeminal autonomic cephalalgia	0	Progression	0	Medication overuse	0	Inpatient treatment	0	Intracranial hypertension	0	Botulinum Toxin	0	Serotonin receptor	0	Mindfulness	0	Cranial nerve	0	Episodic migraine	0	Double vision	1	Vestibular migraine	0	surgical	0	Surgical treatment	0	5-HT	0	Ineffective	0	Eye symptom	0	Female hormone	0
0	Empower	0	Productivity	1	Caregiver	0	Nerve block	0	Accepting	0	Pain management	0	Mechanism	0	spouse	0	Screening	0	Co-morbid	0	Histamine	0	Tricyclic	0	Comorbid	0	Prevalence	0	Advantage	0	Peripheral	0	Dopamine	0	Assessment	0	Physical examination	0	Referral	0	Attitude	0	Gene-related	0	Calcitonin	0	Beta-blocker	0	church	0	Temporal	0	Barbiturate	0	Lidocaine	0	Spectrum	0	Motivation	0	Menstrual migraine	0	Correlate	0	Outpatient	0	Injury	0	Lab test	0	Efficacy	0	Antihistamine	0	Lactation	0	Secondary headache	0	Trigeminal autonomic cephalalgia	0	Progression	0	Medication overuse	0	Inpatient treatment	0	Intracranial hypertension	0	Botulinum Toxin	0	Serotonin receptor	0	Mindfulness	0	Cranial nerve	2	Episodic migraine	2	Double vision	0	Vestibular migraine	0	surgical	0	Surgical treatment	0	5-HT	0	Ineffective	0	Eye symptom	0	Female hormone	0
0	Empower	0	Productivity	0	Caregiver	0	Nerve block	0	Accepting	0	Pain management	0	Mechanism	0	spouse	0	Screening	0	Co-morbid	0	Histamine	0	Tricyclic	0	Comorbid	0	Prevalence	0	Advantage	0	Peripheral	0	Dopamine	0	Assessment	0	Physical examination	0	Referral	0	Attitude	0	Gene-related	0	Calcitonin	0	Beta-blocker	0	church	0	Temporal	0	Barbiturate	0	Lidocaine	0	Spectrum	0	Motivation	0	Menstrual migraine	0	Correlate	0	Outpatient	0	Injury	0	Lab test	0	Efficacy	0	Antihistamine	0	Lactation	0	Secondary headache	0	Trigeminal autonomic cephalalgia	0	Progression	0	Medication overuse	0	Inpatient treatment	0	Intracranial hypertension	0	Botulinum Toxin	0	Serotonin receptor	0	Mindfulness	0	Cranial nerve	0	Episodic migraine	0	Double vision	0	Vestibular migraine	0	surgical	0	Surgical treatment	0	5-HT	0	Ineffective	0	Eye symptom	0	Female hormone	0
0	Empower	0	Productivity	0	Caregiver	0	Nerve block	0	Accepting	0	Pain management	0	Mechanism	0	spouse	0	Screening	0	Co-morbid	0	Histamine	0	Tricyclic	0	Comorbid	0	Prevalence	0	Advantage	0	Peripheral	0	Dopamine	0	Assessment	0	Physical examination	0	Referral	0	Attitude	0	Gene-related	0	Calcitonin	0	Beta-blocker	0	church	0	Temporal	0	Barbiturate	0	Lidocaine	0	Spectrum	0	Motivation	0	Menstrual migraine	0	Correlate	0	Outpatient	0	Injury	0	Lab test	0	Efficacy	0	Antihistamine	0	Lactation	0	Secondary headache	0	Trigeminal autonomic cephalalgia	0	Progression	0	Medication overuse	0	Inpatient treatment	0	Intracranial hypertension	0	Botulinum Toxin	0	Serotonin receptor	0	Mindfulness	0	Cranial nerve	0	Episodic migraine	0	Double vision	0	Vestibular migraine	0	surgical	0	Surgical treatment	0	5-HT	0	Ineffective	0	Eye symptom	0	Female hormone	0
0	Empower	1	Productivity	0	Caregiver	0	Nerve block	0	Accepting	0	Pain management	0	Mechanism	0	spouse	0	Screening	0	Co-morbid	0	Histamine	0	Tricyclic	0	Comorbid	0	Prevalence	0	Advantage	0	Peripheral	0	Dopamine	0	Assessment	0	Physical examination	0	Referral	0	Attitude	1	Gene-related	0	Calcitonin	0	Beta-blocker	0	church	0	Temporal	0	Barbiturate	0	Lidocaine	0	Spectrum	0	Motivation	0	Menstrual migraine	0	Correlate	0	Outpatient	0	Injury	0	Lab test	0	Efficacy	0	Antihistamine	0	Lactation	0	Secondary headache	0	Trigeminal autonomic cephalalgia	0	Progression	0	Medication overuse	0	Inpatient treatment	0	Intracranial hypertension	0	Botulinum Toxin	0	Serotonin receptor	0	Mindfulness	0	Cranial nerve	0	Episodic migraine	0	Double vision	0	Vestibular migraine	0	surgical	0	Surgical treatment	0	5-HT	0	Ineffective	0	Eye symptom	0	Female hormone	0
0	Empower	0	Productivity	0	Caregiver	0	Nerve block	0	Accepting	0	Pain management	0	Mechanism	0	spouse	0	Screening	0	Co-morbid	0	Histamine	0	Tricyclic	0	Comorbid	0	Prevalence	0	Advantage	0	Peripheral	0	Dopamine	0	Assessment	0	Physical examination	0	Referral	0	Attitude	0	Gene-related	0	Calcitonin	0	Beta-blocker	0	church	0	Temporal	0	Barbiturate	0	Lidocaine	0	Spectrum	0	Motivation	0	Menstrual migraine	0	Correlate	0	Outpatient	0	Injury	0	Lab test	0	Efficacy	0	Antihistamine	0	Lactation	0	Secondary headache	0	Trigeminal autonomic cephalalgia	0	Progression	0	Medication overuse	0	Inpatient treatment	0	Intracranial hypertension	0	Botulinum Toxin	0	Serotonin receptor	0	Mindfulness	1	Cranial nerve	0	Episodic migraine	0	Double vision	0	Vestibular migraine	0	surgical	0	Surgical treatment	0	5-HT	0	Ineffective	0	Eye symptom	0	Female hormone	0
0	Empower	0	Productivity	0	Caregiver	0	Nerve block	0	Accepting	0	Pain management	0	Mechanism	0	spouse	0	Screening	0	Co-morbid	0	Histamine	0	Tricyclic	0	Comorbid	0	Prevalence	0	Advantage	0	Peripheral	0	Dopamine	0	Assessment	0	Physical examination	0	Referral	0	Attitude	0	Gene-related	0	Calcitonin	0	Beta-blocker	0	church	0	Temporal	0	Barbiturate	0	Lidocaine	0	Spectrum	0	Motivation	0	Menstrual migraine	0	Correlate	0	Outpatient	0	Injury	0	Lab test	0	Efficacy	0	Antihistamine	0	Lactation	0	Secondary headache	0	Trigeminal autonomic cephalalgia	0	Progression	0	Medication overuse	0	Inpatient treatment	0	Intracranial hypertension	0	Botulinum Toxin	0	Serotonin receptor	1	Mindfulness	0	Cranial nerve	0	Episodic migraine	0	Double vision	0	Vestibular migraine	0	surgical	0	Surgical treatment	0	5-HT	0	Ineffective	0	Eye symptom	0	Female hormone	0
0	Empower	0	Productivity	0	Caregiver	0	Nerve block	0	Accepting	0	Pain management	0	Mechanism	0	spouse	0	Screening	0	Co-morbid	0	Histamine	0	Tricyclic	0	Comorbid	0	Prevalence	0	Advantage	0	Peripheral	0	Dopamine	0	Assessment	0	Physical examination	0	Referral	0	Attitude	0	Gene-related	0	Calcitonin	0	Beta-blocker	0	church	0	Temporal	0	Barbiturate	0	Lidocaine	0	Spectrum	0	Motivation	0	Menstrual migraine	0	Correlate	0	Outpatient	0	Injury	0	Lab test	0	Efficacy	0	Antihistamine	0	Lactation	0	Secondary headache	0	Trigeminal autonomic cephalalgia	0	Progression	0	Medication overuse	0	Inpatient treatment	0	Intracranial hypertension	0	Botulinum Toxin	0	Serotonin receptor	0	Mindfulness	0	Cranial nerve	0	Episodic migraine	0	Double vision	0	Vestibular migraine	0	surgical	0	Surgical treatment	0	5-HT	0	Ineffective	0	Eye symptom	0	Female hormone	0
0	Empower	0	Productivity	0	Caregiver	0	Nerve block	0	Accepting	0	Pain management	0	Mechanism	0	spouse	0	Screening	0	Co-morbid	0	Histamine	0	Tricyclic	0	Comorbid	0	Prevalence	0	Advantage	0	Peripheral	0	Dopamine	0	Assessment	0	Physical examination	0	Referral	0	Attitude	0	Gene-related	0	Calcitonin	0	Beta-blocker	0	church	0	Temporal	0	Barbiturate	0	Lidocaine	0	Spectrum	0	Motivation	0	Menstrual migraine	0	Correlate	0	Outpatient	0	Injury	0	Lab test	0	Efficacy	0	Antihistamine	0	Lactation	0	Secondary headache	0	Trigeminal autonomic cephalalgia	0	Progression	0	Medication overuse	0	Inpatient treatment	0	Intracranial hypertension	0	Botulinum Toxin	0	Serotonin receptor	0	Mindfulness	0	Cranial nerve	0	Episodic migraine	0	Double vision	0	Vestibular migraine	0	surgical	0	Surgical treatment	0	5-HT	0	Ineffective	0	Eye symptom	0	Female hormone	0
0	Empower	0	Productivity	0	Caregiver	0	Nerve block	0	Accepting	0	Pain management	1	Mechanism	0	spouse	0	Screening	0	Co-morbid	0	Histamine	0	Tricyclic	0	Comorbid	0	Prevalence	0	Advantage	0	Peripheral	0	Dopamine	0	Assessment	0	Physical examination	0	Referral	0	Attitude	0	Gene-related	0	Calcitonin	0	Beta-blocker	0	church	0	Temporal	0	Barbiturate	0	Lidocaine	0	Spectrum	0	Motivation	0	Menstrual migraine	0	Correlate	0	Outpatient	0	Injury	0	Lab test	0	Efficacy	0	Antihistamine	0	Lactation	0	Secondary headache	0	Trigeminal autonomic cephalalgia	0	Progression	0	Medication overuse	0	Inpatient treatment	0	Intracranial hypertension	0	Botulinum Toxin	0	Serotonin receptor	0	Mindfulness	0	Cranial nerve	0	Episodic migraine	0	Double vision	0	Vestibular migraine	0	surgical	0	Surgical treatment	0	5-HT	0	Ineffective	0	Eye symptom	0	Female hormone	0
0	Empower	0	Productivity	0	Caregiver	0	Nerve block	0	Accepting	0	Pain management	0	Mechanism	0	spouse	0	Screening	0	Co-morbid	0	Histamine	0	Tricyclic	0	Comorbid	0	Prevalence	0	Advantage	0	Peripheral	0	Dopamine	0	Assessment	0	Physical examination	0	Referral	1	Attitude	0	Gene-related	0	Calcitonin	0	Beta-blocker	0	church	0	Temporal	0	Barbiturate	0	Lidocaine	0	Spectrum	0	Motivation	0	Menstrual migraine	0	Correlate	0	Outpatient	0	Injury	0	Lab test	0	Efficacy	0	Antihistamine	0	Lactation	0	Secondary headache	0	Trigeminal autonomic cephalalgia	0	Progression	0	Medication overuse	0	Inpatient treatment	0	Intracranial hypertension	0	Botulinum Toxin	0	Serotonin receptor	0	Mindfulness	0	Cranial nerve	0	Episodic migraine	0	Double vision	0	Vestibular migraine	0	surgical	0	Surgical treatment	0	5-HT	0	Ineffective	0	Eye symptom	0	Female hormone	0
0	Empower	0	Productivity	0	Caregiver	0	Nerve block	2	Accepting	2	Pain management	1	Mechanism	1	spouse	1	Screening	0	Co-morbid	0	Histamine	0	Tricyclic	0	Comorbid	0	Prevalence	0	Advantage	0	Peripheral	0	Dopamine	0	Assessment	0	Physical examination	0	Referral	0	Attitude	0	Gene-related	0	Calcitonin	0	Beta-blocker	0	church	0	Temporal	1	Barbiturate	1	Lidocaine	1	Spectrum	1	Motivation	1	Menstrual migraine	1	Correlate	0	Outpatient	0	Injury	0	Lab test	0	Efficacy	0	Antihistamine	0	Lactation	0	Secondary headache	0	Trigeminal autonomic cephalalgia	0	Progression	0	Medication overuse	0	Inpatient treatment	0	Intracranial hypertension	0	Botulinum Toxin	0	Serotonin receptor	0	Mindfulness	0	Cranial nerve	0	Episodic migraine	0	Double vision	0	Vestibular migraine	0	surgical	0	Surgical treatment	0	5-HT	0	Ineffective	0	Eye symptom	0	Female hormone	0
0	Empower	0	Productivity	0	Caregiver	0	Nerve block	0	Accepting	0	Pain management	0	Mechanism	0	spouse	0	Screening	0	Co-morbid	0	Histamine	0	Tricyclic	0	Comorbid	0	Prevalence	0	Advantage	0	Peripheral	0	Dopamine	0	Assessment	0	Physical examination	0	Referral	0	Attitude	0	Gene-related	0	Calcitonin	0	Beta-blocker	0	church	0	Temporal	0	Barbiturate	0	Lidocaine	0	Spectrum	0	Motivation	0	Menstrual migraine	0	Correlate	0	Outpatient	0	Injury	0	Lab test	0	Efficacy	0	Antihistamine	0	Lactation	0	Secondary headache	0	Trigeminal autonomic cephalalgia	0	Progression	0	Medication overuse	0	Inpatient treatment	0	Intracranial hypertension	0	Botulinum Toxin	0	Serotonin receptor	0	Mindfulness	0	Cranial nerve	0	Episodic migraine	0	Double vision	0	Vestibular migraine	0	surgical	0	Surgical treatment	0	5-HT	0	Ineffective	0	Eye symptom	0	Female hormone	0
0	Empower	0	Productivity	0	Caregiver	0	Nerve block	0	Accepting	0	Pain management	0	Mechanism	0	spouse	0	Screening	0	Co-morbid	0	Histamine	0	Tricyclic	0	Comorbid	0	Prevalence	0	Advantage	0	Peripheral	0	Dopamine	0	Assessment	0	Physical examination	0	Referral	0	Attitude	0	Gene-related	0	Calcitonin	0	Beta-blocker	0	church	0	Temporal	0	Barbiturate	0	Lidocaine	0	Spectrum	0	Motivation	0	Menstrual migraine	0	Correlate	0	Outpatient	0	Injury	0	Lab test	0	Efficacy	0	Antihistamine	0	Lactation	0	Secondary headache	0	Trigeminal autonomic cephalalgia	0	Progression	0	Medication overuse	0	Inpatient treatment	0	Intracranial hypertension	0	Botulinum Toxin	0	Serotonin receptor	0	Mindfulness	0	Cranial nerve	0	Episodic migraine	0	Double vision	0	Vestibular migraine	0	surgical	0	Surgical treatment	0	5-HT	0	Ineffective	0	Eye symptom	0	Female hormone	0
0	Empower	0	Productivity	0	Caregiver	0	Nerve block	0	Accepting	0	Pain management	0	Mechanism	0	spouse	0	Screening	0	Co-morbid	0	Histamine	0	Tricyclic	0	Comorbid	0	Prevalence	0	Advantage	0	Peripheral	0	Dopamine	0	Assessment	0	Physical examination	1	Referral	0	Attitude	0	Gene-related	0	Calcitonin	0	Beta-blocker	0	church	0	Temporal	0	Barbiturate	0	Lidocaine	0	Spectrum	0	Motivation	0	Menstrual migraine	0	Correlate	0	Outpatient	0	Injury	0	Lab test	0	Efficacy	0	Antihistamine	0	Lactation	0	Secondary headache	0	Trigeminal autonomic cephalalgia	0	Progression	0	Medication overuse	0	Inpatient treatment	0	Intracranial hypertension	0	Botulinum Toxin	0	Serotonin receptor	0	Mindfulness	0	Cranial nerve	0	Episodic migraine	0	Double vision	0	Vestibular migraine	0	surgical	0	Surgical treatment	0	5-HT	0	Ineffective	0	Eye symptom	0	Female hormone	0
0	Empower	0	Productivity	0	Caregiver	0	Nerve block	0	Accepting	0	Pain management	0	Mechanism	0	spouse	0	Screening	0	Co-morbid	0	Histamine	0	Tricyclic	0	Comorbid	0	Prevalence	0	Advantage	0	Peripheral	0	Dopamine	0	Assessment	1	Physical examination	0	Referral	0	Attitude	0	Gene-related	0	Calcitonin	0	Beta-blocker	0	church	0	Temporal	0	Barbiturate	0	Lidocaine	0	Spectrum	0	Motivation	0	Menstrual migraine	0	Correlate	0	Outpatient	0	Injury	0	Lab test	0	Efficacy	0	Antihistamine	0	Lactation	0	Secondary headache	0	Trigeminal autonomic cephalalgia	0	Progression	0	Medication overuse	0	Inpatient treatment	0	Intracranial hypertension	0	Botulinum Toxin	0	Serotonin receptor	0	Mindfulness	0	Cranial nerve	0	Episodic migraine	0	Double vision	0	Vestibular migraine	0	surgical	0	Surgical treatment	0	5-HT	0	Ineffective	0	Eye symptom	0	Female hormone	0
0	Empower	0	Productivity	0	Caregiver	0	Nerve block	0	Accepting	0	Pain management	0	Mechanism	0	spouse	0	Screening	0	Co-morbid	0	Histamine	0	Tricyclic	0	Comorbid	0	Prevalence	0	Advantage	0	Peripheral	0	Dopamine	1	Assessment	0	Physical examination	0	Referral	0	Attitude	0	Gene-related	0	Calcitonin	0	Beta-blocker	0	church	0	Temporal	1	Barbiturate	0	Lidocaine	0	Spectrum	0	Motivation	0	Menstrual migraine	0	Correlate	0	Outpatient	0	Injury	0	Lab test	0	Efficacy	0	Antihistamine	0	Lactation	0	Secondary headache	0	Trigeminal autonomic cephalalgia	0	Progression	0	Medication overuse	0	Inpatient treatment	0	Intracranial hypertension	0	Botulinum Toxin	1	Serotonin receptor	0	Mindfulness	0	Cranial nerve	0	Episodic migraine	0	Double vision	0	Vestibular migraine	0	surgical	0	Surgical treatment	0	5-HT	0	Ineffective	0	Eye symptom	0	Female hormone	0
0	Empower	0	Productivity	0	Caregiver	0	Nerve block	0	Accepting	0	Pain management	0	Mechanism	0	spouse	0	Screening	0	Co-morbid	0	Histamine	0	Tricyclic	0	Comorbid	0	Prevalence	0	Advantage	0	Peripheral	0	Dopamine	0	Assessment	0	Physical examination	0	Referral	0	Attitude	0	Gene-related	0	Calcitonin	0	Beta-blocker	0	church	0	Temporal	0	Barbiturate	0	Lidocaine	0	Spectrum	0	Motivation	0	Menstrual migraine	0	Correlate	0	Outpatient	0	Injury	0	Lab test	0	Efficacy	0	Antihistamine	0	Lactation	0	Secondary headache	0	Trigeminal autonomic cephalalgia	0	Progression	0	Medication overuse	0	Inpatient treatment	0	Intracranial hypertension	0	Botulinum Toxin	0	Serotonin receptor	0	Mindfulness	0	Cranial nerve	0	Episodic migraine	0	Double vision	0	Vestibular migraine	0	surgical	0	Surgical treatment	0	5-HT	0	Ineffective	0	Eye symptom	0	Female hormone	0
0	Empower	0	Productivity	0	Caregiver	0	Nerve block	0	Accepting	0	Pain management	0	Mechanism	0	spouse	0	Screening	0	Co-morbid	0	Histamine	0	Tricyclic	0	Comorbid	0	Prevalence	0	Advantage	0	Peripheral	2	Dopamine	0	Assessment	0	Physical examination	0	Referral	0	Attitude	0	Gene-related	0	Calcitonin	0	Beta-blocker	0	church	0	Temporal	0	Barbiturate	0	Lidocaine	0	Spectrum	0	Motivation	0	Menstrual migraine	0	Correlate	0	Outpatient	0	Injury	0	Lab test	0	Efficacy	0	Antihistamine	0	Lactation	0	Secondary headache	0	Trigeminal autonomic cephalalgia	0	Progression	0	Medication overuse	0	Inpatient treatment	0	Intracranial hypertension	0	Botulinum Toxin	0	Serotonin receptor	0	Mindfulness	0	Cranial nerve	0	Episodic migraine	0	Double vision	0	Vestibular migraine	0	surgical	0	Surgical treatment	0	5-HT	0	Ineffective	0	Eye symptom	0	Female hormone	0
0	Empower	0	Productivity	0	Caregiver	0	Nerve block	0	Accepting	0	Pain management	0	Mechanism	0	spouse	0	Screening	0	Co-morbid	0	Histamine	0	Tricyclic	0	Comorbid	0	Prevalence	0	Advantage	0	Peripheral	0	Dopamine	0	Assessment	0	Physical examination	0	Referral	0	Attitude	0	Gene-related	0	Calcitonin	0	Beta-blocker	0	church	0	Temporal	0	Barbiturate	0	Lidocaine	0	Spectrum	0	Motivation	0	Menstrual migraine	0	Correlate	0	Outpatient	0	Injury	0	Lab test	0	Efficacy	0	Antihistamine	0	Lactation	0	Secondary headache	0	Trigeminal autonomic cephalalgia	0	Progression	0	Medication overuse	0	Inpatient treatment	0	Intracranial hypertension	1	Botulinum Toxin	0	Serotonin receptor	0	Mindfulness	0	Cranial nerve	0	Episodic migraine	0	Double vision	0	Vestibular migraine	0	surgical	0	Surgical treatment	0	5-HT	0	Ineffective	0	Eye symptom	0	Female hormone	0
0	Empower	0	Productivity	0	Caregiver	0	Nerve block	0	Accepting	0	Pain management	0	Mechanism	1	spouse	0	Screening	0	Co-morbid	0	Histamine	0	Tricyclic	0	Comorbid	0	Prevalence	0	Advantage	0	Peripheral	0	Dopamine	0	Assessment	0	Physical examination	0	Referral	0	Attitude	0	Gene-related	0	Calcitonin	0	Beta-blocker	0	church	0	Temporal	0	Barbiturate	0	Lidocaine	0	Spectrum	0	Motivation	0	Menstrual migraine	0	Correlate	0	Outpatient	0	Injury	0	Lab test	0	Efficacy	0	Antihistamine	0	Lactation	0	Secondary headache	0	Trigeminal autonomic cephalalgia	0	Progression	0	Medication overuse	0	Inpatient treatment	0	Intracranial hypertension	0	Botulinum Toxin	0	Serotonin receptor	0	Mindfulness	0	Cranial nerve	0	Episodic migraine	0	Double vision	0	Vestibular migraine	0	surgical	0	Surgical treatment	0	5-HT	0	Ineffective	0	Eye symptom	0	Female hormone	1
0	Empower	0	Productivity	0	Caregiver	0	Nerve block	0	Accepting	0	Pain management	0	Mechanism	0	spouse	0	Screening	0	Co-morbid	0	Histamine	0	Tricyclic	0	Comorbid	0	Prevalence	0	Advantage	0	Peripheral	0	Dopamine	0	Assessment	0	Physical examination	0	Referral	0	Attitude	0	Gene-related	0	Calcitonin	0	Beta-blocker	0	church	1	Temporal	0	Barbiturate	0	Lidocaine	0	Spectrum	0	Motivation	0	Menstrual migraine	0	Correlate	0	Outpatient	0	Injury	0	Lab test	0	Efficacy	0	Antihistamine	0	Lactation	0	Secondary headache	0	Trigeminal autonomic cephalalgia	0	Progression	0	Medication overuse	0	Inpatient treatment	0	Intracranial hypertension	0	Botulinum Toxin	0	Serotonin receptor	0	Mindfulness	0	Cranial nerve	0	Episodic migraine	0	Double vision	0	Vestibular migraine	0	surgical	0	Surgical treatment	0	5-HT	1	Ineffective	1	Eye symptom	1	Female hormone	0
0	Empower	0	Productivity	0	Caregiver	0	Nerve block	0	Accepting	0	Pain management	0	Mechanism	0	spouse	0	Screening	0	Co-morbid	0	Histamine	0	Tricyclic	0	Comorbid	0	Prevalence	0	Advantage	0	Peripheral	1	Dopamine	0	Assessment	0	Physical examination	0	Referral	0	Attitude	0	Gene-related	0	Calcitonin	0	Beta-blocker	0	church	0	Temporal	0	Barbiturate	0	Lidocaine	0	Spectrum	0	Motivation	0	Menstrual migraine	0	Correlate	0	Outpatient	0	Injury	0	Lab test	0	Efficacy	0	Antihistamine	0	Lactation	0	Secondary headache	0	Trigeminal autonomic cephalalgia	0	Progression	0	Medication overuse	0	Inpatient treatment	0	Intracranial hypertension	0	Botulinum Toxin	0	Serotonin receptor	0	Mindfulness	0	Cranial nerve	0	Episodic migraine	0	Double vision	0	Vestibular migraine	0	surgical	0	Surgical treatment	0	5-HT	0	Ineffective	0	Eye symptom	0	Female hormone	0
0	Empower	0	Productivity	0	Caregiver	0	Nerve block	0	Accepting	0	Pain management	0	Mechanism	0	spouse	0	Screening	0	Co-morbid	0	Histamine	0	Tricyclic	0	Comorbid	0	Prevalence	0	Advantage	1	Peripheral	0	Dopamine	0	Assessment	0	Physical examination	0	Referral	0	Attitude	0	Gene-related	0	Calcitonin	0	Beta-blocker	0	church	0	Temporal	0	Barbiturate	0	Lidocaine	0	Spectrum	0	Motivation	0	Menstrual migraine	0	Correlate	0	Outpatient	0	Injury	0	Lab test	0	Efficacy	0	Antihistamine	0	Lactation	0	Secondary headache	0	Trigeminal autonomic cephalalgia	0	Progression	0	Medication overuse	0	Inpatient treatment	0	Intracranial hypertension	0	Botulinum Toxin	0	Serotonin receptor	0	Mindfulness	0	Cranial nerve	0	Episodic migraine	0	Double vision	0	Vestibular migraine	0	surgical	0	Surgical treatment	0	5-HT	0	Ineffective	0	Eye symptom	0	Female hormone	0
0	Empower	0	Productivity	0	Caregiver	0	Nerve block	0	Accepting	0	Pain management	0	Mechanism	0	spouse	0	Screening	0	Co-morbid	0	Histamine	0	Tricyclic	0	Comorbid	0	Prevalence	0	Advantage	0	Peripheral	0	Dopamine	0	Assessment	0	Physical examination	0	Referral	0	Attitude	0	Gene-related	0	Calcitonin	0	Beta-blocker	0	church	0	Temporal	0	Barbiturate	0	Lidocaine	0	Spectrum	0	Motivation	0	Menstrual migraine	0	Correlate	0	Outpatient	0	Injury	0	Lab test	0	Efficacy	0	Antihistamine	0	Lactation	0	Secondary headache	0	Trigeminal autonomic cephalalgia	0	Progression	0	Medication overuse	0	Inpatient treatment	0	Intracranial hypertension	0	Botulinum Toxin	0	Serotonin receptor	0	Mindfulness	0	Cranial nerve	0	Episodic migraine	0	Double vision	0	Vestibular migraine	0	surgical	0	Surgical treatment	0	5-HT	0	Ineffective	0	Eye symptom	0	Female hormone	0
0	Empower	0	Productivity	0	Caregiver	0	Nerve block	0	Accepting	0	Pain management	0	Mechanism	0	spouse	0	Screening	0	Co-morbid	0	Histamine	0	Tricyclic	0	Comorbid	0	Prevalence	0	Advantage	0	Peripheral	0	Dopamine	0	Assessment	0	Physical examination	0	Referral	0	Attitude	0	Gene-related	0	Calcitonin	0	Beta-blocker	0	church	0	Temporal	0	Barbiturate	0	Lidocaine	0	Spectrum	0	Motivation	0	Menstrual migraine	0	Correlate	0	Outpatient	0	Injury	0	Lab test	0	Efficacy	0	Antihistamine	0	Lactation	0	Secondary headache	0	Trigeminal autonomic cephalalgia	0	Progression	0	Medication overuse	0	Inpatient treatment	0	Intracranial hypertension	0	Botulinum Toxin	0	Serotonin receptor	0	Mindfulness	0	Cranial nerve	0	Episodic migraine	0	Double vision	0	Vestibular migraine	0	surgical	0	Surgical treatment	0	5-HT	0	Ineffective	0	Eye symptom	0	Female hormone	0
1	Empower	0	Productivity	0	Caregiver	0	Nerve block	0	Accepting	0	Pain management	0	Mechanism	0	spouse	0	Screening	0	Co-morbid	0	Histamine	0	Tricyclic	0	Comorbid	0	Prevalence	0	Advantage	0	Peripheral	0	Dopamine	0	Assessment	0	Physical examination	0	Referral	0	Attitude	0	Gene-related	0	Calcitonin	0	Beta-blocker	0	church	0	Temporal	0	Barbiturate	0	Lidocaine	0	Spectrum	0	Motivation	0	Menstrual migraine	0	Correlate	0	Outpatient	0	Injury	0	Lab test	0	Efficacy	0	Antihistamine	0	Lactation	0	Secondary headache	0	Trigeminal autonomic cephalalgia	0	Progression	0	Medication overuse	0	Inpatient treatment	1	Intracranial hypertension	0	Botulinum Toxin	0	Serotonin receptor	0	Mindfulness	0	Cranial nerve	0	Episodic migraine	0	Double vision	0	Vestibular migraine	0	surgical	0	Surgical treatment	0	5-HT	0	Ineffective	0	Eye symptom	0	Female hormone	0
0	Empower	0	Productivity	0	Caregiver	0	Nerve block	0	Accepting	0	Pain management	0	Mechanism	0	spouse	0	Screening	0	Co-morbid	0	Histamine	0	Tricyclic	0	Comorbid	0	Prevalence	0	Advantage	0	Peripheral	0	Dopamine	0	Assessment	0	Physical examination	0	Referral	0	Attitude	0	Gene-related	0	Calcitonin	0	Beta-blocker	0	church	0	Temporal	0	Barbiturate	0	Lidocaine	0	Spectrum	0	Motivation	0	Menstrual migraine	0	Correlate	0	Outpatient	0	Injury	0	Lab test	0	Efficacy	0	Antihistamine	0	Lactation	0	Secondary headache	0	Trigeminal autonomic cephalalgia	0	Progression	0	Medication overuse	0	Inpatient treatment	0	Intracranial hypertension	0	Botulinum Toxin	0	Serotonin receptor	0	Mindfulness	0	Cranial nerve	0	Episodic migraine	0	Double vision	0	Vestibular migraine	0	surgical	0	Surgical treatment	0	5-HT	0	Ineffective	0	Eye symptom	0	Female hormone	0
0	Empower	0	Productivity	0	Caregiver	0	Nerve block	0	Accepting	0	Pain management	0	Mechanism	0	spouse	0	Screening	0	Co-morbid	0	Histamine	0	Tricyclic	0	Comorbid	0	Prevalence	0	Advantage	0	Peripheral	0	Dopamine	0	Assessment	0	Physical examination	0	Referral	0	Attitude	0	Gene-related	0	Calcitonin	0	Beta-blocker	0	church	0	Temporal	0	Barbiturate	0	Lidocaine	0	Spectrum	0	Motivation	0	Menstrual migraine	0	Correlate	0	Outpatient	0	Injury	0	Lab test	0	Efficacy	0	Antihistamine	0	Lactation	0	Secondary headache	0	Trigeminal autonomic cephalalgia	0	Progression	0	Medication overuse	0	Inpatient treatment	0	Intracranial hypertension	0	Botulinum Toxin	0	Serotonin receptor	0	Mindfulness	0	Cranial nerve	0	Episodic migraine	0	Double vision	0	Vestibular migraine	0	surgical	0	Surgical treatment	0	5-HT	0	Ineffective	0	Eye symptom	0	Female hormone	0
0	Empower	0	Productivity	1	Caregiver	1	Nerve block	0	Accepting	0	Pain management	0	Mechanism	0	spouse	0	Screening	0	Co-morbid	0	Histamine	0	Tricyclic	0	Comorbid	0	Prevalence	0	Advantage	0	Peripheral	0	Dopamine	0	Assessment	0	Physical examination	0	Referral	0	Attitude	0	Gene-related	1	Calcitonin	1	Beta-blocker	0	church	0	Temporal	0	Barbiturate	0	Lidocaine	0	Spectrum	0	Motivation	0	Menstrual migraine	0	Correlate	0	Outpatient	0	Injury	0	Lab test	0	Efficacy	0	Antihistamine	0	Lactation	0	Secondary headache	0	Trigeminal autonomic cephalalgia	0	Progression	0	Medication overuse	0	Inpatient treatment	0	Intracranial hypertension	0	Botulinum Toxin	0	Serotonin receptor	0	Mindfulness	0	Cranial nerve	0	Episodic migraine	0	Double vision	0	Vestibular migraine	0	surgical	0	Surgical treatment	0	5-HT	0	Ineffective	0	Eye symptom	0	Female hormone	0
0	Empower	0	Productivity	1	Caregiver	1	Nerve block	0	Accepting	0	Pain management	0	Mechanism	0	spouse	0	Screening	0	Co-morbid	0	Histamine	0	Tricyclic	0	Comorbid	0	Prevalence	0	Advantage	0	Peripheral	0	Dopamine	0	Assessment	0	Physical examination	0	Referral	0	Attitude	0	Gene-related	1	Calcitonin	1	Beta-blocker	0	church	0	Temporal	0	Barbiturate	0	Lidocaine	0	Spectrum	0	Motivation	0	Menstrual migraine	0	Correlate	0	Outpatient	0	Injury	0	Lab test	0	Efficacy	0	Antihistamine	0	Lactation	0	Secondary headache	0	Trigeminal autonomic cephalalgia	0	Progression	0	Medication overuse	0	Inpatient treatment	0	Intracranial hypertension	0	Botulinum Toxin	0	Serotonin receptor	0	Mindfulness	0	Cranial nerve	0	Episodic migraine	0	Double vision	0	Vestibular migraine	0	surgical	0	Surgical treatment	0	5-HT	0	Ineffective	0	Eye symptom	0	Female hormone	0
0	Empower	1	Productivity	0	Caregiver	0	Nerve block	0	Accepting	0	Pain management	0	Mechanism	0	spouse	0	Screening	0	Co-morbid	0	Histamine	0	Tricyclic	0	Comorbid	0	Prevalence	0	Advantage	0	Peripheral	0	Dopamine	0	Assessment	0	Physical examination	0	Referral	0	Attitude	0	Gene-related	0	Calcitonin	0	Beta-blocker	0	church	0	Temporal	0	Barbiturate	0	Lidocaine	0	Spectrum	0	Motivation	0	Menstrual migraine	0	Correlate	0	Outpatient	0	Injury	0	Lab test	0	Efficacy	0	Antihistamine	0	Lactation	0	Secondary headache	0	Trigeminal autonomic cephalalgia	0	Progression	0	Medication overuse	2	Inpatient treatment	0	Intracranial hypertension	0	Botulinum Toxin	0	Serotonin receptor	0	Mindfulness	1	Cranial nerve	0	Episodic migraine	0	Double vision	0	Vestibular migraine	0	surgical	0	Surgical treatment	0	5-HT	0	Ineffective	0	Eye symptom	0	Female hormone	0
0	Empower	0	Productivity	0	Caregiver	0	Nerve block	0	Accepting	0	Pain management	0	Mechanism	0	spouse	0	Screening	0	Co-morbid	0	Histamine	0	Tricyclic	0	Comorbid	0	Prevalence	0	Advantage	0	Peripheral	0	Dopamine	0	Assessment	0	Physical examination	0	Referral	0	Attitude	0	Gene-related	0	Calcitonin	0	Beta-blocker	0	church	0	Temporal	0	Barbiturate	0	Lidocaine	0	Spectrum	0	Motivation	0	Menstrual migraine	0	Correlate	0	Outpatient	0	Injury	0	Lab test	0	Efficacy	0	Antihistamine	0	Lactation	0	Secondary headache	0	Trigeminal autonomic cephalalgia	0	Progression	0	Medication overuse	0	Inpatient treatment	0	Intracranial hypertension	0	Botulinum Toxin	0	Serotonin receptor	0	Mindfulness	0	Cranial nerve	0	Episodic migraine	0	Double vision	0	Vestibular migraine	0	surgical	0	Surgical treatment	0	5-HT	0	Ineffective	0	Eye symptom	0	Female hormone	0
0	Empower	0	Productivity	0	Caregiver	0	Nerve block	0	Accepting	0	Pain management	0	Mechanism	0	spouse	0	Screening	0	Co-morbid	0	Histamine	0	Tricyclic	0	Comorbid	0	Prevalence	0	Advantage	0	Peripheral	0	Dopamine	0	Assessment	0	Physical examination	0	Referral	0	Attitude	0	Gene-related	0	Calcitonin	0	Beta-blocker	0	church	0	Temporal	0	Barbiturate	0	Lidocaine	0	Spectrum	0	Motivation	0	Menstrual migraine	0	Correlate	0	Outpatient	0	Injury	0	Lab test	0	Efficacy	0	Antihistamine	0	Lactation	0	Secondary headache	0	Trigeminal autonomic cephalalgia	0	Progression	0	Medication overuse	0	Inpatient treatment	0	Intracranial hypertension	0	Botulinum Toxin	0	Serotonin receptor	0	Mindfulness	0	Cranial nerve	0	Episodic migraine	0	Double vision	0	Vestibular migraine	0	surgical	0	Surgical treatment	0	5-HT	0	Ineffective	0	Eye symptom	0	Female hormone	0
0	Empower	0	Productivity	0	Caregiver	0	Nerve block	0	Accepting	0	Pain management	0	Mechanism	0	spouse	0	Screening	0	Co-morbid	0	Histamine	0	Tricyclic	0	Comorbid	0	Prevalence	0	Advantage	0	Peripheral	0	Dopamine	0	Assessment	0	Physical examination	0	Referral	0	Attitude	1	Gene-related	0	Calcitonin	0	Beta-blocker	0	church	0	Temporal	0	Barbiturate	0	Lidocaine	0	Spectrum	0	Motivation	0	Menstrual migraine	0	Correlate	0	Outpatient	0	Injury	0	Lab test	0	Efficacy	0	Antihistamine	0	Lactation	0	Secondary headache	0	Trigeminal autonomic cephalalgia	0	Progression	0	Medication overuse	0	Inpatient treatment	0	Intracranial hypertension	0	Botulinum Toxin	0	Serotonin receptor	0	Mindfulness	0	Cranial nerve	0	Episodic migraine	0	Double vision	0	Vestibular migraine	0	surgical	0	Surgical treatment	0	5-HT	0	Ineffective	0	Eye symptom	0	Female hormone	0
0	Empower	0	Productivity	0	Caregiver	0	Nerve block	0	Accepting	0	Pain management	0	Mechanism	0	spouse	0	Screening	0	Co-morbid	0	Histamine	0	Tricyclic	0	Comorbid	0	Prevalence	0	Advantage	0	Peripheral	0	Dopamine	0	Assessment	0	Physical examination	0	Referral	0	Attitude	0	Gene-related	0	Calcitonin	0	Beta-blocker	0	church	0	Temporal	0	Barbiturate	0	Lidocaine	0	Spectrum	1	Motivation	0	Menstrual migraine	0	Correlate	0	Outpatient	0	Injury	0	Lab test	0	Efficacy	0	Antihistamine	0	Lactation	0	Secondary headache	0	Trigeminal autonomic cephalalgia	0	Progression	0	Medication overuse	0	Inpatient treatment	0	Intracranial hypertension	0	Botulinum Toxin	0	Serotonin receptor	0	Mindfulness	0	Cranial nerve	0	Episodic migraine	0	Double vision	0	Vestibular migraine	0	surgical	0	Surgical treatment	0	5-HT	0	Ineffective	0	Eye symptom	0	Female hormone	0
0	Empower	0	Productivity	0	Caregiver	0	Nerve block	0	Accepting	0	Pain management	0	Mechanism	0	spouse	1	Screening	0	Co-morbid	0	Histamine	0	Tricyclic	0	Comorbid	0	Prevalence	0	Advantage	0	Peripheral	0	Dopamine	0	Assessment	0	Physical examination	0	Referral	0	Attitude	0	Gene-related	0	Calcitonin	0	Beta-blocker	0	church	0	Temporal	0	Barbiturate	0	Lidocaine	0	Spectrum	0	Motivation	0	Menstrual migraine	0	Correlate	0	Outpatient	0	Injury	0	Lab test	0	Efficacy	0	Antihistamine	0	Lactation	0	Secondary headache	0	Trigeminal autonomic cephalalgia	0	Progression	1	Medication overuse	0	Inpatient treatment	0	Intracranial hypertension	0	Botulinum Toxin	0	Serotonin receptor	0	Mindfulness	0	Cranial nerve	0	Episodic migraine	0	Double vision	0	Vestibular migraine	0	surgical	0	Surgical treatment	0	5-HT	0	Ineffective	0	Eye symptom	0	Female hormone	0
0	Empower	0	Productivity	0	Caregiver	0	Nerve block	0	Accepting	0	Pain management	0	Mechanism	0	spouse	0	Screening	0	Co-morbid	0	Histamine	0	Tricyclic	0	Comorbid	0	Prevalence	0	Advantage	0	Peripheral	0	Dopamine	0	Assessment	0	Physical examination	0	Referral	0	Attitude	0	Gene-related	0	Calcitonin	0	Beta-blocker	0	church	0	Temporal	0	Barbiturate	0	Lidocaine	0	Spectrum	0	Motivation	0	Menstrual migraine	0	Correlate	0	Outpatient	0	Injury	0	Lab test	0	Efficacy	0	Antihistamine	0	Lactation	0	Secondary headache	2	Trigeminal autonomic cephalalgia	2	Progression	0	Medication overuse	0	Inpatient treatment	0	Intracranial hypertension	0	Botulinum Toxin	0	Serotonin receptor	0	Mindfulness	0	Cranial nerve	0	Episodic migraine	0	Double vision	0	Vestibular migraine	0	surgical	0	Surgical treatment	0	5-HT	0	Ineffective	0	Eye symptom	0	Female hormone	0
0	Empower	0	Productivity	0	Caregiver	0	Nerve block	0	Accepting	0	Pain management	0	Mechanism	0	spouse	0	Screening	0	Co-morbid	0	Histamine	0	Tricyclic	0	Comorbid	0	Prevalence	0	Advantage	0	Peripheral	0	Dopamine	0	Assessment	0	Physical examination	0	Referral	0	Attitude	0	Gene-related	0	Calcitonin	0	Beta-blocker	0	church	0	Temporal	0	Barbiturate	0	Lidocaine	0	Spectrum	0	Motivation	0	Menstrual migraine	0	Correlate	0	Outpatient	0	Injury	0	Lab test	0	Efficacy	0	Antihistamine	0	Lactation	0	Secondary headache	0	Trigeminal autonomic cephalalgia	0	Progression	0	Medication overuse	0	Inpatient treatment	0	Intracranial hypertension	0	Botulinum Toxin	0	Serotonin receptor	0	Mindfulness	0	Cranial nerve	0	Episodic migraine	0	Double vision	0	Vestibular migraine	0	surgical	0	Surgical treatment	0	5-HT	0	Ineffective	0	Eye symptom	0	Female hormone	0
0	Empower	0	Productivity	0	Caregiver	0	Nerve block	0	Accepting	0	Pain management	0	Mechanism	0	spouse	0	Screening	0	Co-morbid	0	Histamine	0	Tricyclic	0	Comorbid	0	Prevalence	0	Advantage	0	Peripheral	0	Dopamine	1	Assessment	0	Physical examination	0	Referral	0	Attitude	0	Gene-related	0	Calcitonin	0	Beta-blocker	0	church	1	Temporal	0	Barbiturate	0	Lidocaine	0	Spectrum	0	Motivation	0	Menstrual migraine	0	Correlate	0	Outpatient	0	Injury	0	Lab test	0	Efficacy	0	Antihistamine	0	Lactation	0	Secondary headache	0	Trigeminal autonomic cephalalgia	0	Progression	0	Medication overuse	0	Inpatient treatment	0	Intracranial hypertension	1	Botulinum Toxin	0	Serotonin receptor	0	Mindfulness	0	Cranial nerve	0	Episodic migraine	0	Double vision	1	Vestibular migraine	0	surgical	0	Surgical treatment	0	5-HT	0	Ineffective	0	Eye symptom	0	Female hormone	0
0	Empower	0	Productivity	0	Caregiver	0	Nerve block	0	Accepting	0	Pain management	0	Mechanism	0	spouse	0	Screening	0	Co-morbid	0	Histamine	0	Tricyclic	0	Comorbid	2	Prevalence	1	Advantage	0	Peripheral	0	Dopamine	0	Assessment	0	Physical examination	0	Referral	0	Attitude	0	Gene-related	0	Calcitonin	0	Beta-blocker	0	church	0	Temporal	0	Barbiturate	0	Lidocaine	0	Spectrum	0	Motivation	0	Menstrual migraine	0	Correlate	0	Outpatient	0	Injury	0	Lab test	0	Efficacy	0	Antihistamine	0	Lactation	0	Secondary headache	0	Trigeminal autonomic cephalalgia	0	Progression	0	Medication overuse	0	Inpatient treatment	0	Intracranial hypertension	0	Botulinum Toxin	0	Serotonin receptor	0	Mindfulness	0	Cranial nerve	0	Episodic migraine	0	Double vision	0	Vestibular migraine	0	surgical	0	Surgical treatment	0	5-HT	0	Ineffective	0	Eye symptom	0	Female hormone	0
0	Empower	0	Productivity	0	Caregiver	0	Nerve block	0	Accepting	0	Pain management	0	Mechanism	0	spouse	0	Screening	0	Co-morbid	0	Histamine	0	Tricyclic	0	Comorbid	0	Prevalence	0	Advantage	0	Peripheral	0	Dopamine	0	Assessment	0	Physical examination	0	Referral	0	Attitude	0	Gene-related	0	Calcitonin	0	Beta-blocker	0	church	0	Temporal	0	Barbiturate	0	Lidocaine	0	Spectrum	0	Motivation	0	Menstrual migraine	0	Correlate	0	Outpatient	0	Injury	0	Lab test	0	Efficacy	0	Antihistamine	0	Lactation	0	Secondary headache	0	Trigeminal autonomic cephalalgia	0	Progression	0	Medication overuse	0	Inpatient treatment	0	Intracranial hypertension	0	Botulinum Toxin	0	Serotonin receptor	0	Mindfulness	0	Cranial nerve	0	Episodic migraine	0	Double vision	0	Vestibular migraine	0	surgical	0	Surgical treatment	0	5-HT	0	Ineffective	0	Eye symptom	0	Female hormone	0
0	Empower	0	Productivity	0	Caregiver	0	Nerve block	0	Accepting	0	Pain management	0	Mechanism	0	spouse	0	Screening	0	Co-morbid	0	Histamine	0	Tricyclic	0	Comorbid	0	Prevalence	0	Advantage	0	Peripheral	0	Dopamine	0	Assessment	0	Physical examination	0	Referral	0	Attitude	0	Gene-related	0	Calcitonin	0	Beta-blocker	0	church	0	Temporal	0	Barbiturate	0	Lidocaine	0	Spectrum	0	Motivation	0	Menstrual migraine	0	Correlate	0	Outpatient	0	Injury	0	Lab test	0	Efficacy	0	Antihistamine	0	Lactation	0	Secondary headache	0	Trigeminal autonomic cephalalgia	0	Progression	0	Medication overuse	0	Inpatient treatment	0	Intracranial hypertension	0	Botulinum Toxin	0	Serotonin receptor	0	Mindfulness	0	Cranial nerve	0	Episodic migraine	0	Double vision	0	Vestibular migraine	0	surgical	0	Surgical treatment	0	5-HT	0	Ineffective	0	Eye symptom	0	Female hormone	0
0	Empower	0	Productivity	0	Caregiver	0	Nerve block	0	Accepting	0	Pain management	0	Mechanism	0	spouse	0	Screening	0	Co-morbid	0	Histamine	0	Tricyclic	0	Comorbid	0	Prevalence	0	Advantage	0	Peripheral	0	Dopamine	0	Assessment	0	Physical examination	0	Referral	0	Attitude	0	Gene-related	0	Calcitonin	0	Beta-blocker	0	church	0	Temporal	0	Barbiturate	0	Lidocaine	0	Spectrum	0	Motivation	0	Menstrual migraine	0	Correlate	0	Outpatient	0	Injury	0	Lab test	0	Efficacy	0	Antihistamine	0	Lactation	0	Secondary headache	0	Trigeminal autonomic cephalalgia	0	Progression	0	Medication overuse	0	Inpatient treatment	0	Intracranial hypertension	0	Botulinum Toxin	0	Serotonin receptor	0	Mindfulness	0	Cranial nerve	0	Episodic migraine	0	Double vision	0	Vestibular migraine	0	surgical	0	Surgical treatment	0	5-HT	0	Ineffective	0	Eye symptom	0	Female hormone	0
0	Empower	2	Productivity	0	Caregiver	0	Nerve block	0	Accepting	0	Pain management	0	Mechanism	0	spouse	0	Screening	0	Co-morbid	0	Histamine	0	Tricyclic	0	Comorbid	0	Prevalence	0	Advantage	0	Peripheral	0	Dopamine	0	Assessment	0	Physical examination	0	Referral	0	Attitude	0	Gene-related	0	Calcitonin	0	Beta-blocker	0	church	0	Temporal	0	Barbiturate	0	Lidocaine	0	Spectrum	0	Motivation	0	Menstrual migraine	0	Correlate	0	Outpatient	0	Injury	0	Lab test	0	Efficacy	0	Antihistamine	0	Lactation	0	Secondary headache	0	Trigeminal autonomic cephalalgia	0	Progression	0	Medication overuse	0	Inpatient treatment	0	Intracranial hypertension	0	Botulinum Toxin	0	Serotonin receptor	0	Mindfulness	0	Cranial nerve	0	Episodic migraine	0	Double vision	0	Vestibular migraine	0	surgical	0	Surgical treatment	0	5-HT	0	Ineffective	0	Eye symptom	0	Female hormone	0
0	Empower	0	Productivity	0	Caregiver	0	Nerve block	0	Accepting	0	Pain management	0	Mechanism	0	spouse	0	Screening	0	Co-morbid	0	Histamine	0	Tricyclic	0	Comorbid	0	Prevalence	0	Advantage	0	Peripheral	0	Dopamine	0	Assessment	0	Physical examination	0	Referral	0	Attitude	0	Gene-related	0	Calcitonin	0	Beta-blocker	0	church	0	Temporal	0	Barbiturate	0	Lidocaine	0	Spectrum	0	Motivation	0	Menstrual migraine	0	Correlate	0	Outpatient	0	Injury	0	Lab test	0	Efficacy	0	Antihistamine	0	Lactation	0	Secondary headache	0	Trigeminal autonomic cephalalgia	0	Progression	0	Medication overuse	0	Inpatient treatment	0	Intracranial hypertension	0	Botulinum Toxin	0	Serotonin receptor	0	Mindfulness	0	Cranial nerve	0	Episodic migraine	0	Double vision	0	Vestibular migraine	0	surgical	1	Surgical treatment	1	5-HT	0	Ineffective	0	Eye symptom	0	Female hormone	0
0	Empower	0	Productivity	0	Caregiver	0	Nerve block	0	Accepting	0	Pain management	0	Mechanism	0	spouse	0	Screening	0	Co-morbid	0	Histamine	0	Tricyclic	0	Comorbid	0	Prevalence	0	Advantage	0	Peripheral	0	Dopamine	0	Assessment	0	Physical examination	0	Referral	0	Attitude	0	Gene-related	0	Calcitonin	0	Beta-blocker	0	church	0	Temporal	0	Barbiturate	0	Lidocaine	0	Spectrum	0	Motivation	0	Menstrual migraine	0	Correlate	0	Outpatient	0	Injury	0	Lab test	0	Efficacy	0	Antihistamine	0	Lactation	0	Secondary headache	0	Trigeminal autonomic cephalalgia	0	Progression	0	Medication overuse	0	Inpatient treatment	0	Intracranial hypertension	0	Botulinum Toxin	0	Serotonin receptor	0	Mindfulness	0	Cranial nerve	0	Episodic migraine	0	Double vision	0	Vestibular migraine	0	surgical	0	Surgical treatment	0	5-HT	0	Ineffective	0	Eye symptom	0	Female hormone	0
0	Empower	0	Productivity	0	Caregiver	0	Nerve block	0	Accepting	0	Pain management	0	Mechanism	0	spouse	0	Screening	0	Co-morbid	0	Histamine	0	Tricyclic	1	Comorbid	0	Prevalence	0	Advantage	0	Peripheral	0	Dopamine	0	Assessment	0	Physical examination	0	Referral	0	Attitude	0	Gene-related	0	Calcitonin	0	Beta-blocker	0	church	0	Temporal	0	Barbiturate	0	Lidocaine	0	Spectrum	0	Motivation	0	Menstrual migraine	1	Correlate	0	Outpatient	0	Injury	0	Lab test	0	Efficacy	0	Antihistamine	0	Lactation	2	Secondary headache	0	Trigeminal autonomic cephalalgia	0	Progression	0	Medication overuse	0	Inpatient treatment	0	Intracranial hypertension	0	Botulinum Toxin	0	Serotonin receptor	0	Mindfulness	0	Cranial nerve	0	Episodic migraine	0	Double vision	0	Vestibular migraine	0	surgical	0	Surgical treatment	0	5-HT	0	Ineffective	0	Eye symptom	0	Female hormone	0
3	Empower	0	Productivity	0	Caregiver	0	Nerve block	0	Accepting	0	Pain management	0	Mechanism	0	spouse	0	Screening	0	Co-morbid	0	Histamine	2	Tricyclic	2	Comorbid	0	Prevalence	0	Advantage	2	Peripheral	0	Dopamine	1	Assessment	0	Physical examination	0	Referral	0	Attitude	0	Gene-related	0	Calcitonin	0	Beta-blocker	0	church	0	Temporal	0	Barbiturate	0	Lidocaine	0	Spectrum	0	Motivation	0	Menstrual migraine	0	Correlate	0	Outpatient	0	Injury	0	Lab test	0	Efficacy	2	Antihistamine	1	Lactation	0	Secondary headache	0	Trigeminal autonomic cephalalgia	0	Progression	1	Medication overuse	0	Inpatient treatment	0	Intracranial hypertension	0	Botulinum Toxin	1	Serotonin receptor	0	Mindfulness	0	Cranial nerve	0	Episodic migraine	0	Double vision	0	Vestibular migraine	0	surgical	0	Surgical treatment	0	5-HT	0	Ineffective	0	Eye symptom	0	Female hormone	0
0	Empower	0	Productivity	0	Caregiver	0	Nerve block	0	Accepting	0	Pain management	0	Mechanism	0	spouse	0	Screening	0	Co-morbid	0	Histamine	0	Tricyclic	0	Comorbid	0	Prevalence	0	Advantage	0	Peripheral	0	Dopamine	0	Assessment	0	Physical examination	0	Referral	0	Attitude	0	Gene-related	0	Calcitonin	0	Beta-blocker	1	church	0	Temporal	0	Barbiturate	0	Lidocaine	0	Spectrum	0	Motivation	0	Menstrual migraine	0	Correlate	0	Outpatient	0	Injury	0	Lab test	0	Efficacy	0	Antihistamine	0	Lactation	0	Secondary headache	0	Trigeminal autonomic cephalalgia	0	Progression	0	Medication overuse	0	Inpatient treatment	0	Intracranial hypertension	0	Botulinum Toxin	0	Serotonin receptor	0	Mindfulness	0	Cranial nerve	0	Episodic migraine	0	Double vision	0	Vestibular migraine	0	surgical	0	Surgical treatment	0	5-HT	0	Ineffective	0	Eye symptom	0	Female hormone	0
0	Empower	0	Productivity	0	Caregiver	0	Nerve block	0	Accepting	0	Pain management	0	Mechanism	0	spouse	0	Screening	3	Co-morbid	3	Histamine	0	Tricyclic	0	Comorbid	1	Prevalence	2	Advantage	0	Peripheral	0	Dopamine	0	Assessment	2	Physical examination	2	Referral	2	Attitude	0	Gene-related	0	Calcitonin	0	Beta-blocker	0	church	0	Temporal	0	Barbiturate	0	Lidocaine	0	Spectrum	0	Motivation	1	Menstrual migraine	0	Correlate	2	Outpatient	2	Injury	1	Lab test	2	Efficacy	0	Antihistamine	0	Lactation	0	Secondary headache	0	Trigeminal autonomic cephalalgia	0	Progression	0	Medication overuse	0	Inpatient treatment	1	Intracranial hypertension	0	Botulinum Toxin	0	Serotonin receptor	0	Mindfulness	0	Cranial nerve	0	Episodic migraine	0	Double vision	0	Vestibular migraine	0	surgical	0	Surgical treatment	0	5-HT	0	Ineffective	0	Eye symptom	0	Female hormone	0
0	Empower	0	Productivity	0	Caregiver	0	Nerve block	0	Accepting	0	Pain management	1	Mechanism	0	spouse	0	Screening	0	Co-morbid	0	Histamine	0	Tricyclic	0	Comorbid	0	Prevalence	0	Advantage	0	Peripheral	0	Dopamine	0	Assessment	0	Physical examination	0	Referral	0	Attitude	0	Gene-related	0	Calcitonin	0	Beta-blocker	0	church	0	Temporal	0	Barbiturate	1	Lidocaine	0	Spectrum	0	Motivation	0	Menstrual migraine	0	Correlate	0	Outpatient	0	Injury	1	Lab test	0	Efficacy	0	Antihistamine	0	Lactation	0	Secondary headache	0	Trigeminal autonomic cephalalgia	0	Progression	0	Medication overuse	0	Inpatient treatment	0	Intracranial hypertension	0	Botulinum Toxin	0	Serotonin receptor	0	Mindfulness	0	Cranial nerve	0	Episodic migraine	0	Double vision	0	Vestibular migraine	0	surgical	0	Surgical treatment	0	5-HT	0	Ineffective	0	Eye symptom	0	Female hormone	0
0	Empower	0	Productivity	0	Caregiver	0	Nerve block	0	Accepting	0	Pain management	0	Mechanism	0	spouse	0	Screening	0	Co-morbid	0	Histamine	0	Tricyclic	0	Comorbid	0	Prevalence	0	Advantage	0	Peripheral	0	Dopamine	0	Assessment	0	Physical examination	0	Referral	0	Attitude	0	Gene-related	0	Calcitonin	0	Beta-blocker	0	church	0	Temporal	0	Barbiturate	0	Lidocaine	0	Spectrum	0	Motivation	0	Menstrual migraine	0	Correlate	0	Outpatient	0	Injury	0	Lab test	0	Efficacy	0	Antihistamine	0	Lactation	0	Secondary headache	0	Trigeminal autonomic cephalalgia	0	Progression	0	Medication overuse	0	Inpatient treatment	0	Intracranial hypertension	0	Botulinum Toxin	0	Serotonin receptor	0	Mindfulness	0	Cranial nerve	0	Episodic migraine	0	Double vision	0	Vestibular migraine	0	surgical	0	Surgical treatment	0	5-HT	0	Ineffective	0	Eye symptom	0	Female hormone	0
0	Empower	0	Productivity	0	Caregiver	0	Nerve block	0	Accepting	0	Pain management	0	Mechanism	0	spouse	0	Screening	0	Co-morbid	0	Histamine	0	Tricyclic	0	Comorbid	0	Prevalence	0	Advantage	0	Peripheral	0	Dopamine	0	Assessment	0	Physical examination	0	Referral	0	Attitude	0	Gene-related	0	Calcitonin	0	Beta-blocker	0	church	0	Temporal	0	Barbiturate	0	Lidocaine	0	Spectrum	0	Motivation	0	Menstrual migraine	0	Correlate	0	Outpatient	0	Injury	0	Lab test	0	Efficacy	0	Antihistamine	0	Lactation	0	Secondary headache	0	Trigeminal autonomic cephalalgia	0	Progression	0	Medication overuse	0	Inpatient treatment	0	Intracranial hypertension	0	Botulinum Toxin	0	Serotonin receptor	0	Mindfulness	0	Cranial nerve	0	Episodic migraine	0	Double vision	0	Vestibular migraine	0	surgical	0	Surgical treatment	0	5-HT	0	Ineffective	0	Eye symptom	0	Female hormone	0
0	Empower	0	Productivity	0	Caregiver	0	Nerve block	0	Accepting	0	Pain management	0	Mechanism	0	spouse	0	Screening	0	Co-morbid	0	Histamine	0	Tricyclic	0	Comorbid	0	Prevalence	0	Advantage	0	Peripheral	0	Dopamine	0	Assessment	0	Physical examination	0	Referral	0	Attitude	0	Gene-related	0	Calcitonin	0	Beta-blocker	0	church	0	Temporal	0	Barbiturate	0	Lidocaine	0	Spectrum	0	Motivation	0	Menstrual migraine	0	Correlate	0	Outpatient	0	Injury	0	Lab test	0	Efficacy	0	Antihistamine	0	Lactation	0	Secondary headache	0	Trigeminal autonomic cephalalgia	0	Progression	0	Medication overuse	0	Inpatient treatment	0	Intracranial hypertension	0	Botulinum Toxin	0	Serotonin receptor	0	Mindfulness	0	Cranial nerve	0	Episodic migraine	0	Double vision	0	Vestibular migraine	0	surgical	0	Surgical treatment	0	5-HT	0	Ineffective	0	Eye symptom	0	Female hormone	0
0	Empower	0	Productivity	0	Caregiver	0	Nerve block	0	Accepting	0	Pain management	0	Mechanism	0	spouse	0	Screening	0	Co-morbid	0	Histamine	0	Tricyclic	0	Comorbid	0	Prevalence	0	Advantage	0	Peripheral	0	Dopamine	0	Assessment	0	Physical examination	0	Referral	0	Attitude	0	Gene-related	0	Calcitonin	0	Beta-blocker	0	church	0	Temporal	0	Barbiturate	0	Lidocaine	0	Spectrum	0	Motivation	0	Menstrual migraine	0	Correlate	0	Outpatient	0	Injury	0	Lab test	0	Efficacy	0	Antihistamine	0	Lactation	0	Secondary headache	0	Trigeminal autonomic cephalalgia	0	Progression	0	Medication overuse	0	Inpatient treatment	0	Intracranial hypertension	0	Botulinum Toxin	0	Serotonin receptor	0	Mindfulness	0	Cranial nerve	0	Episodic migraine	0	Double vision	0	Vestibular migraine	0	surgical	0	Surgical treatment	0	5-HT	0	Ineffective	0	Eye symptom	0	Female hormone	0
0	Empower	0	Productivity	0	Caregiver	0	Nerve block	0	Accepting	0	Pain management	0	Mechanism	0	spouse	0	Screening	0	Co-morbid	0	Histamine	1	Tricyclic	0	Comorbid	0	Prevalence	0	Advantage	0	Peripheral	0	Dopamine	0	Assessment	0	Physical examination	0	Referral	0	Attitude	0	Gene-related	0	Calcitonin	0	Beta-blocker	0	church	0	Temporal	0	Barbiturate	0	Lidocaine	0	Spectrum	0	Motivation	0	Menstrual migraine	0	Correlate	0	Outpatient	0	Injury	0	Lab test	0	Efficacy	0	Antihistamine	1	Lactation	0	Secondary headache	0	Trigeminal autonomic cephalalgia	0	Progression	0	Medication overuse	0	Inpatient treatment	0	Intracranial hypertension	0	Botulinum Toxin	0	Serotonin receptor	0	Mindfulness	0	Cranial nerve	0	Episodic migraine	0	Double vision	0	Vestibular migraine	0	surgical	0	Surgical treatment	0	5-HT	0	Ineffective	0	Eye symptom	0	Female hormone	0
0	Empower	0	Productivity	0	Caregiver	0	Nerve block	0	Accepting	0	Pain management	0	Mechanism	0	spouse	1	Screening	0	Co-morbid	0	Histamine	0	Tricyclic	0	Comorbid	0	Prevalence	0	Advantage	0	Peripheral	0	Dopamine	0	Assessment	0	Physical examination	0	Referral	0	Attitude	1	Gene-related	0	Calcitonin	0	Beta-blocker	0	church	0	Temporal	0	Barbiturate	0	Lidocaine	0	Spectrum	0	Motivation	0	Menstrual migraine	0	Correlate	0	Outpatient	0	Injury	0	Lab test	0	Efficacy	0	Antihistamine	0	Lactation	0	Secondary headache	0	Trigeminal autonomic cephalalgia	0	Progression	0	Medication overuse	0	Inpatient treatment	0	Intracranial hypertension	0	Botulinum Toxin	0	Serotonin receptor	0	Mindfulness	0	Cranial nerve	0	Episodic migraine	0	Double vision	0	Vestibular migraine	0	surgical	0	Surgical treatment	0	5-HT	0	Ineffective	0	Eye symptom	0	Female hormone	0
0	Empower	0	Productivity	0	Caregiver	0	Nerve block	0	Accepting	0	Pain management	0	Mechanism	0	spouse	0	Screening	0	Co-morbid	0	Histamine	0	Tricyclic	0	Comorbid	0	Prevalence	0	Advantage	0	Peripheral	0	Dopamine	0	Assessment	0	Physical examination	0	Referral	0	Attitude	0	Gene-related	0	Calcitonin	0	Beta-blocker	0	church	0	Temporal	0	Barbiturate	0	Lidocaine	0	Spectrum	0	Motivation	0	Menstrual migraine	0	Correlate	0	Outpatient	0	Injury	0	Lab test	0	Efficacy	0	Antihistamine	0	Lactation	0	Secondary headache	0	Trigeminal autonomic cephalalgia	0	Progression	0	Medication overuse	0	Inpatient treatment	0	Intracranial hypertension	0	Botulinum Toxin	0	Serotonin receptor	1	Mindfulness	0	Cranial nerve	0	Episodic migraine	0	Double vision	0	Vestibular migraine	0	surgical	0	Surgical treatment	0	5-HT	0	Ineffective	0	Eye symptom	0	Female hormone	0
0	Empower	0	Productivity	0	Caregiver	0	Nerve block	0	Accepting	0	Pain management	0	Mechanism	0	spouse	0	Screening	0	Co-morbid	0	Histamine	0	Tricyclic	0	Comorbid	0	Prevalence	0	Advantage	0	Peripheral	0	Dopamine	0	Assessment	0	Physical examination	0	Referral	0	Attitude	0	Gene-related	0	Calcitonin	0	Beta-blocker	0	church	0	Temporal	0	Barbiturate	0	Lidocaine	0	Spectrum	0	Motivation	0	Menstrual migraine	0	Correlate	0	Outpatient	0	Injury	0	Lab test	0	Efficacy	0	Antihistamine	0	Lactation	0	Secondary headache	0	Trigeminal autonomic cephalalgia	0	Progression	0	Medication overuse	0	Inpatient treatment	0	Intracranial hypertension	0	Botulinum Toxin	0	Serotonin receptor	0	Mindfulness	0	Cranial nerve	0	Episodic migraine	0	Double vision	0	Vestibular migraine	0	surgical	0	Surgical treatment	0	5-HT	0	Ineffective	0	Eye symptom	0	Female hormone	0
0	Empower	0	Productivity	0	Caregiver	0	Nerve block	0	Accepting	0	Pain management	0	Mechanism	0	spouse	0	Screening	0	Co-morbid	0	Histamine	0	Tricyclic	0	Comorbid	0	Prevalence	0	Advantage	0	Peripheral	0	Dopamine	0	Assessment	0	Physical examination	0	Referral	0	Attitude	0	Gene-related	0	Calcitonin	0	Beta-blocker	0	church	0	Temporal	0	Barbiturate	0	Lidocaine	0	Spectrum	0	Motivation	0	Menstrual migraine	0	Correlate	0	Outpatient	0	Injury	0	Lab test	0	Efficacy	0	Antihistamine	0	Lactation	0	Secondary headache	0	Trigeminal autonomic cephalalgia	0	Progression	0	Medication overuse	0	Inpatient treatment	0	Intracranial hypertension	0	Botulinum Toxin	0	Serotonin receptor	0	Mindfulness	0	Cranial nerve	0	Episodic migraine	0	Double vision	0	Vestibular migraine	0	surgical	0	Surgical treatment	0	5-HT	0	Ineffective	0	Eye symptom	0	Female hormone	0
4		4		4		4		4		4		4		4		4		[illegible]		[illegible]		[illegible]		[illegible]		[illegible]		[illegible]		[illegible]		[illegible]		[illegible]		[illegible]		[illegible]		[illegible]		[illegible]		[illegible]		[illegible]		[illegible]		[illegible]		[illegible]		[illegible]		[illegible]		[illegible]		[illegible]		[illegible]		2		2		2		2		2		2		2		2		2		2		2		2		2		2		2		2		2		2		2		2		2		2		2		2		2		2

P257a	P257b	P258a	P258b	P259a	P259b	P260a	P260b	P261a	P261b	P262a	P262b	P263a	P263b	P264a	P264b	P265a	P265b	P266a	P266b	P267a	P267b	P268a	P268b	P269a	P269b	P270a	P270b	P271a	P271b	P272a	P272b	P273a	P273b	P274a	P274b	P275a	P275b	P276a	P276b	P277a	P277b	P278a	P278b	P279a	P279b	P280a	P280b	P281a	P281b	P282a	P282b	P283a	P283b	P284a	P284b	P285a	P285b	P286a	P286b	P287a	P287b	P288a	P288b	P289a	P289b	P290a	P290b	P291a	P291b	P292a	P292b	P293a	P293b	P294a	P294b	P295a	P295b
Social worker	0	Folate	0	Septoplasty	1	nan	1	Eye pain	1	Angry	1	Emergency care	1	Food allergies	1	Cadasil	1	5HT	1	Anatomy	0	Counselor	0	Phenotype	0	Abnormalities	0	Comorbidity	0	Corticosteroid	0	Mood disorder	0	CT scan	0	Foramen ovale	0	Angiotensin Converting Enzyme	0	Prophylactic treatment	0	Subtype	0	Randomized controlled trial	0	Melatonin	0	Referred pain	0	Comorbidities	0	Behavioral therapy	0	Transmission	0	Father	0	Preemptive	0	Grandfather	0	Adherence	0	Clinician	0	Complicated migraine	0	Placebo effect	0	Consultation	0	Irk	0	Beta-Blockers	0	carer	0
Social worker	1	Folate	1	Septoplasty	0	nan	0	Eye pain	0	Angry	0	Emergency care	0	Food allergies	0	Cadasil	0	5HT	0	Anatomy	0	Counselor	0	Phenotype	0	Abnormalities	0	Comorbidity	0	Corticosteroid	0	Mood disorder	0	CT scan	0	Foramen ovale	0	Angiotensin Converting Enzyme	0	Prophylactic treatment	0	Subtype	0	Randomized controlled trial	0	Melatonin	0	Referred pain	0	Comorbidities	0	Behavioral therapy	0	Transmission	0	Father	0	Preemptive	0	Grandfather	0	Adherence	0	Clinician	0	Complicated migraine	0	Placebo effect	0	Consultation	0	Irk	0	Beta-Blockers	0	carer	0
Social worker	0	Folate	0	Septoplasty	0	nan	0	Eye pain	0	Angry	0	Emergency care	0	Food allergies	0	Cadasil	0	5HT	0	Anatomy	0	Counselor	0	Phenotype	0	Abnormalities	0	Comorbidity	0	Corticosteroid	0	Mood disorder	0	CT scan	0	Foramen ovale	0	Angiotensin Converting Enzyme	0	Prophylactic treatment	0	Subtype	0	Randomized controlled trial	0	Melatonin	0	Referred pain	0	Comorbidities	0	Behavioral therapy	0	Transmission	0	Father	0	Preemptive	0	Grandfather	0	Adherence	0	Clinician	0	Complicated migraine	0	Placebo effect	0	Consultation	0	Irk	0	Beta-Blockers	0	carer	0
Social worker	0	Folate	0	Septoplasty	0	nan	0	Eye pain	0	Angry	0	Emergency care	0	Food allergies	0	Cadasil	0	5HT	0	Anatomy	0	Counselor	0	Phenotype	0	Abnormalities	0	Comorbidity	0	Corticosteroid	0	Mood disorder	0	CT scan	0	Foramen ovale	0	Angiotensin Converting Enzyme	0	Prophylactic treatment	0	Subtype	0	Randomized controlled trial	0	Melatonin	0	Referred pain	0	Comorbidities	0	Behavioral therapy	0	Transmission	0	Father	0	Preemptive	0	Grandfather	0	Adherence	0	Clinician	0	Complicated migraine	0	Placebo effect	0	Consultation	0	Irk	0	Beta-Blockers	0	carer	0
Social worker	0	Folate	0	Septoplasty	0	nan	0	Eye pain	0	Angry	0	Emergency care	0	Food allergies	0	Cadasil	0	5HT	0	Anatomy	0	Counselor	0	Phenotype	0	Abnormalities	0	Comorbidity	0	Corticosteroid	0	Mood disorder	0	CT scan	0	Foramen ovale	0	Angiotensin Converting Enzyme	0	Prophylactic treatment	0	Subtype	0	Randomized controlled trial	0	Melatonin	0	Referred pain	0	Comorbidities	0	Behavioral therapy	0	Transmission	0	Father	0	Preemptive	0	Grandfather	0	Adherence	0	Clinician	0	Complicated migraine	0	Placebo effect	0	Consultation	0	Irk	0	Beta-Blockers	0	carer	0
Social worker	0	Folate	0	Septoplasty	0	nan	0	Eye pain	0	Angry	0	Emergency care	0	Food allergies	0	Cadasil	0	5HT	0	Anatomy	0	Counselor	0	Phenotype	0	Abnormalities	0	Comorbidity	0	Corticosteroid	0	Mood disorder	0	CT scan	0	Foramen ovale	0	Angiotensin Converting Enzyme	0	Prophylactic treatment	0	Subtype	0	Randomized controlled trial	0	Melatonin	0	Referred pain	0	Comorbidities	0	Behavioral therapy	0	Transmission	0	Father	0	Preemptive	0	Grandfather	0	Adherence	0	Clinician	0	Complicated migraine	0	Placebo effect	0	Consultation	0	Irk	0	Beta-Blockers	0	carer	0
Social worker	0	Folate	0	Septoplasty	0	nan	0	Eye pain	0	Angry	0	Emergency care	0	Food allergies	0	Cadasil	0	5HT	0	Anatomy	0	Counselor	0	Phenotype	0	Abnormalities	0	Comorbidity	0	Corticosteroid	0	Mood disorder	0	CT scan	0	Foramen ovale	0	Angiotensin Converting Enzyme	0	Prophylactic treatment	0	Subtype	0	Randomized controlled trial	0	Melatonin	0	Referred pain	0	Comorbidities	0	Behavioral therapy	0	Transmission	0	Father	0	Preemptive	0	Grandfather	0	Adherence	0	Clinician	0	Complicated migraine	0	Placebo effect	0	Consultation	0	Irk	0	Beta-Blockers	0	carer	0
Social worker	0	Folate	0	Septoplasty	0	nan	0	Eye pain	0	Angry	0	Emergency care	0	Food allergies	0	Cadasil	0	5HT	0	Anatomy	0	Counselor	0	Phenotype	0	Abnormalities	0	Comorbidity	0	Corticosteroid	0	Mood disorder	0	CT scan	0	Foramen ovale	0	Angiotensin Converting Enzyme	0	Prophylactic treatment	0	Subtype	0	Randomized controlled trial	0	Melatonin	0	Referred pain	0	Comorbidities	0	Behavioral therapy	0	Transmission	0	Father	0	Preemptive	0	Grandfather	0	Adherence	0	Clinician	0	Complicated migraine	0	Placebo effect	0	Consultation	0	Irk	0	Beta-Blockers	0	carer	1
Social worker	0	Folate	0	Septoplasty	0	nan	0	Eye pain	0	Angry	0	Emergency care	0	Food allergies	0	Cadasil	0	5HT	0	Anatomy	0	Counselor	0	Phenotype	0	Abnormalities	0	Comorbidity	0	Corticosteroid	0	Mood disorder	0	CT scan	0	Foramen ovale	0	Angiotensin Converting Enzyme	0	Prophylactic treatment	0	Subtype	0	Randomized controlled trial	0	Melatonin	0	Referred pain	0	Comorbidities	0	Behavioral therapy	0	Transmission	0	Father	0	Preemptive	0	Grandfather	0	Adherence	0	Clinician	0	Complicated migraine	0	Placebo effect	0	Consultation	0	Irk	0	Beta-Blockers	0	carer	0
Social worker	0	Folate	0	Septoplasty	0	nan	0	Eye pain	0	Angry	0	Emergency care	0	Food allergies	0	Cadasil	0	5HT	0	Anatomy	0	Counselor	0	Phenotype	0	Abnormalities	0	Comorbidity	0	Corticosteroid	0	Mood disorder	0	CT scan	0	Foramen ovale	0	Angiotensin Converting Enzyme	0	Prophylactic treatment	0	Subtype	0	Randomized controlled trial	0	Melatonin	0	Referred pain	0	Comorbidities	0	Behavioral therapy	0	Transmission	0	Father	0	Preemptive	0	Grandfather	0	Adherence	0	Clinician	0	Complicated migraine	0	Placebo effect	0	Consultation	0	Irk	0	Beta-Blockers	0	carer	0
Social worker	0	Folate	0	Septoplasty	0	nan	0	Eye pain	0	Angry	0	Emergency care	0	Food allergies	0	Cadasil	0	5HT	0	Anatomy	0	Counselor	0	Phenotype	0	Abnormalities	0	Comorbidity	0	Corticosteroid	0	Mood disorder	0	CT scan	0	Foramen ovale	0	Angiotensin Converting Enzyme	0	Prophylactic treatment	0	Subtype	0	Randomized controlled trial	0	Melatonin	0	Referred pain	0	Comorbidities	0	Behavioral therapy	0	Transmission	0	Father	0	Preemptive	0	Grandfather	0	Adherence	0	Clinician	0	Complicated migraine	0	Placebo effect	0	Consultation	0	Irk	0	Beta-Blockers	0	carer	0
Social worker	0	Folate	0	Septoplasty	0	nan	0	Eye pain	0	Angry	0	Emergency care	0	Food allergies	0	Cadasil	0	5HT	0	Anatomy	0	Counselor	0	Phenotype	0	Abnormalities	0	Comorbidity	0	Corticosteroid	0	Mood disorder	0	CT scan	0	Foramen ovale	0	Angiotensin Converting Enzyme	0	Prophylactic treatment	0	Subtype	0	Randomized controlled trial	0	Melatonin	0	Referred pain	0	Comorbidities	0	Behavioral therapy	0	Transmission	0	Father	0	Preemptive	0	Grandfather	0	Adherence	0	Clinician	0	Complicated migraine	0	Placebo effect	0	Consultation	0	Irk	0	Beta-Blockers	0	carer	0
Social worker	0	Folate	0	Septoplasty	0	nan	0	Eye pain	0	Angry	0	Emergency care	0	Food allergies	0	Cadasil	0	5HT	0	Anatomy	0	Counselor	0	Phenotype	0	Abnormalities	0	Comorbidity	0	Corticosteroid	0	Mood disorder	0	CT scan	0	Foramen ovale	0	Angiotensin Converting Enzyme	0	Prophylactic treatment	0	Subtype	0	Randomized controlled trial	0	Melatonin	0	Referred pain	0	Comorbidities	0	Behavioral therapy	0	Transmission	0	Father	0	Preemptive	0	Grandfather	0	Adherence	0	Clinician	0	Complicated migraine	0	Placebo effect	0	Consultation	0	Irk	0	Beta-Blockers	0	carer	0
Social worker	0	Folate	0	Septoplasty	0	nan	0	Eye pain	0	Angry	0	Emergency care	0	Food allergies	0	Cadasil	0	5HT	0	Anatomy	0	Counselor	0	Phenotype	0	Abnormalities	0	Comorbidity	0	Corticosteroid	0	Mood disorder	0	CT scan	0	Foramen ovale	0	Angiotensin Converting Enzyme	0	Prophylactic treatment	0	Subtype	0	Randomized controlled trial	0	Melatonin	0	Referred pain	0	Comorbidities	0	Behavioral therapy	0	Transmission	0	Father	0	Preemptive	0	Grandfather	0	Adherence	0	Clinician	0	Complicated migraine	0	Placebo effect	0	Consultation	0	Irk	0	Beta-Blockers	0	carer	0
Social worker	0	Folate	0	Septoplasty	0	nan	0	Eye pain	0	Angry	0	Emergency care	0	Food allergies	0	Cadasil	0	5HT	0	Anatomy	0	Counselor	0	Phenotype	0	Abnormalities	0	Comorbidity	0	Corticosteroid	0	Mood disorder	0	CT scan	0	Foramen ovale	0	Angiotensin Converting Enzyme	0	Prophylactic treatment	0	Subtype	0	Randomized controlled trial	0	Melatonin	0	Referred pain	0	Comorbidities	0	Behavioral therapy	0	Transmission	0	Father	0	Preemptive	0	Grandfather	0	Adherence	0	Clinician	0	Complicated migraine	0	Placebo effect	0	Consultation	0	Irk	0	Beta-Blockers	0	carer	0
Social worker	0	Folate	0	Septoplasty	0	nan	0	Eye pain	0	Angry	0	Emergency care	0	Food allergies	0	Cadasil	0	5HT	0	Anatomy	0	Counselor	0	Phenotype	0	Abnormalities	0	Comorbidity	0	Corticosteroid	0	Mood disorder	0	CT scan	0	Foramen ovale	0	Angiotensin Converting Enzyme	0	Prophylactic treatment	0	Subtype	0	Randomized controlled trial	0	Melatonin	0	Referred pain	0	Comorbidities	0	Behavioral therapy	0	Transmission	0	Father	0	Preemptive	0	Grandfather	0	Adherence	0	Clinician	0	Complicated migraine	0	Placebo effect	0	Consultation	0	Irk	0	Beta-Blockers	0	carer	0
Social worker	0	Folate	0	Septoplasty	0	nan	0	Eye pain	0	Angry	0	Emergency care	0	Food allergies	0	Cadasil	0	5HT	0	Anatomy	0	Counselor	0	Phenotype	0	Abnormalities	0	Comorbidity	0	Corticosteroid	0	Mood disorder	0	CT scan	0	Foramen ovale	0	Angiotensin Converting Enzyme	0	Prophylactic treatment	0	Subtype	0	Randomized controlled trial	0	Melatonin	0	Referred pain	0	Comorbidities	0	Behavioral therapy	0	Transmission	0	Father	0	Preemptive	0	Grandfather	0	Adherence	0	Clinician	0	Complicated migraine	0	Placebo effect	0	Consultation	0	Irk	0	Beta-Blockers	0	carer	0
Social worker	0	Folate	0	Septoplasty	0	nan	0	Eye pain	0	Angry	0	Emergency care	0	Food allergies	0	Cadasil	0	5HT	0	Anatomy	0	Counselor	0	Phenotype	0	Abnormalities	0	Comorbidity	0	Corticosteroid	0	Mood disorder	0	CT scan	0	Foramen ovale	0	Angiotensin Converting Enzyme	0	Prophylactic treatment	0	Subtype	0	Randomized controlled trial	0	Melatonin	0	Referred pain	0	Comorbidities	0	Behavioral therapy	0	Transmission	0	Father	0	Preemptive	0	Grandfather	0	Adherence	0	Clinician	0	Complicated migraine	0	Placebo effect	0	Consultation	0	Irk	0	Beta-Blockers	0	carer	0
Social worker	0	Folate	0	Septoplasty	0	nan	0	Eye pain	0	Angry	0	Emergency care	0	Food allergies	0	Cadasil	0	5HT	0	Anatomy	0	Counselor	0	Phenotype	0	Abnormalities	0	Comorbidity	0	Corticosteroid	0	Mood disorder	0	CT scan	0	Foramen ovale	0	Angiotensin Converting Enzyme	0	Prophylactic treatment	0	Subtype	0	Randomized controlled trial	0	Melatonin	0	Referred pain	0	Comorbidities	0	Behavioral therapy	0	Transmission	0	Father	0	Preemptive	0	Grandfather	0	Adherence	0	Clinician	0	Complicated migraine	0	Placebo effect	0	Consultation	0	Irk	0	Beta-Blockers	0	carer	0
Social worker	0	Folate	0	Septoplasty	0	nan	0	Eye pain	0	Angry	0	Emergency care	0	Food allergies	0	Cadasil	0	5HT	0	Anatomy	0	Counselor	0	Phenotype	0	Abnormalities	0	Comorbidity	0	Corticosteroid	0	Mood disorder	0	CT scan	0	Foramen ovale	0	Angiotensin Converting Enzyme	0	Prophylactic treatment	0	Subtype	0	Randomized controlled trial	0	Melatonin	0	Referred pain	0	Comorbidities	0	Behavioral therapy	0	Transmission	0	Father	0	Preemptive	0	Grandfather	0	Adherence	0	Clinician	0	Complicated migraine	0	Placebo effect	0	Consultation	0	Irk	0	Beta-Blockers	0	carer	0
Social worker	0	Folate	0	Septoplasty	0	nan	0	Eye pain	0	Angry	0	Emergency care	0	Food allergies	0	Cadasil	0	5HT	0	Anatomy	0	Counselor	0	Phenotype	0	Abnormalities	0	Comorbidity	0	Corticosteroid	0	Mood disorder	0	CT scan	0	Foramen ovale	0	Angiotensin Converting Enzyme	0	Prophylactic treatment	0	Subtype	0	Randomized controlled trial	0	Melatonin	0	Referred pain	0	Comorbidities	0	Behavioral therapy	0	Transmission	0	Father	0	Preemptive	0	Grandfather	0	Adherence	0	Clinician	0	Complicated migraine	0	Placebo effect	0	Consultation	0	Irk	0	Beta-Blockers	0	carer	0
Social worker	0	Folate	0	Septoplasty	0	nan	0	Eye pain	0	Angry	0	Emergency care	0	Food allergies	0	Cadasil	0	5HT	0	Anatomy	0	Counselor	0	Phenotype	0	Abnormalities	0	Comorbidity	0	Corticosteroid	0	Mood disorder	0	CT scan	0	Foramen ovale	0	Angiotensin Converting Enzyme	0	Prophylactic treatment	0	Subtype	0	Randomized controlled trial	0	Melatonin	0	Referred pain	0	Comorbidities	0	Behavioral therapy	0	Transmission	0	Father	0	Preemptive	0	Grandfather	0	Adherence	0	Clinician	0	Complicated migraine	0	Placebo effect	0	Consultation	0	Irk	0	Beta-Blockers	0	carer	0
Social worker	0	Folate	0	Septoplasty	0	nan	0	Eye pain	0	Angry	0	Emergency care	0	Food allergies	0	Cadasil	0	5HT	0	Anatomy	0	Counselor	0	Phenotype	0	Abnormalities	0	Comorbidity	0	Corticosteroid	0	Mood disorder	0	CT scan	0	Foramen ovale	0	Angiotensin Converting Enzyme	0	Prophylactic treatment	0	Subtype	0	Randomized controlled trial	0	Melatonin	0	Referred pain	0	Comorbidities	0	Behavioral therapy	0	Transmission	0	Father	0	Preemptive	0	Grandfather	0	Adherence	0	Clinician	0	Complicated migraine	0	Placebo effect	0	Consultation	0	Irk	0	Beta-Blockers	0	carer	0
Social worker	0	Folate	0	Septoplasty	0	nan	0	Eye pain	0	Angry	0	Emergency care	0	Food allergies	0	Cadasil	0	5HT	0	Anatomy	0	Counselor	0	Phenotype	0	Abnormalities	0	Comorbidity	0	Corticosteroid	0	Mood disorder	0	CT scan	0	Foramen ovale	0	Angiotensin Converting Enzyme	0	Prophylactic treatment	0	Subtype	0	Randomized controlled trial	0	Melatonin	0	Referred pain	0	Comorbidities	0	Behavioral therapy	0	Transmission	0	Father	0	Preemptive	0	Grandfather	0	Adherence	0	Clinician	0	Complicated migraine	0	Placebo effect	0	Consultation	0	Irk	0	Beta-Blockers	0	carer	0
Social worker	0	Folate	0	Septoplasty	0	nan	0	Eye pain	0	Angry	0	Emergency care	0	Food allergies	0	Cadasil	0	5HT	0	Anatomy	0	Counselor	0	Phenotype	0	Abnormalities	0	Comorbidity	0	Corticosteroid	0	Mood disorder	0	CT scan	0	Foramen ovale	0	Angiotensin Converting Enzyme	0	Prophylactic treatment	0	Subtype	0	Randomized controlled trial	0	Melatonin	0	Referred pain	0	Comorbidities	0	Behavioral therapy	0	Transmission	0	Father	0	Preemptive	0	Grandfather	0	Adherence	0	Clinician	0	Complicated migraine	0	Placebo effect	0	Consultation	0	Irk	0	Beta-Blockers	0	carer	0
Social worker	0	Folate	0	Septoplasty	0	nan	0	Eye pain	0	Angry	0	Emergency care	0	Food allergies	0	Cadasil	0	5HT	0	Anatomy	0	Counselor	0	Phenotype	0	Abnormalities	0	Comorbidity	0	Corticosteroid	0	Mood disorder	0	CT scan	0	Foramen ovale	0	Angiotensin Converting Enzyme	0	Prophylactic treatment	0	Subtype	0	Randomized controlled trial	0	Melatonin	0	Referred pain	0	Comorbidities	0	Behavioral therapy	0	Transmission	0	Father	0	Preemptive	0	Grandfather	0	Adherence	0	Clinician	0	Complicated migraine	0	Placebo effect	0	Consultation	0	Irk	0	Beta-Blockers	0	carer	0
Social worker	0	Folate	0	Septoplasty	0	nan	0	Eye pain	0	Angry	0	Emergency care	0	Food allergies	0	Cadasil	0	5HT	0	Anatomy	0	Counselor	0	Phenotype	0	Abnormalities	0	Comorbidity	0	Corticosteroid	0	Mood disorder	0	CT scan	0	Foramen ovale	0	Angiotensin Converting Enzyme	0	Prophylactic treatment	0	Subtype	0	Randomized controlled trial	0	Melatonin	0	Referred pain	0	Comorbidities	0	Behavioral therapy	0	Transmission	0	Father	0	Preemptive	0	Grandfather	0	Adherence	0	Clinician	0	Complicated migraine	0	Placebo effect	0	Consultation	0	Irk	0	Beta-Blockers	1	carer	0
Social worker	0	Folate	0	Septoplasty	0	nan	0	Eye pain	0	Angry	0	Emergency care	0	Food allergies	0	Cadasil	0	5HT	0	Anatomy	0	Counselor	0	Phenotype	0	Abnormalities	0	Comorbidity	0	Corticosteroid	0	Mood disorder	0	CT scan	0	Foramen ovale	0	Angiotensin Converting Enzyme	0	Prophylactic treatment	0	Subtype	0	Randomized controlled trial	0	Melatonin	0	Referred pain	0	Comorbidities	0	Behavioral therapy	0	Transmission	0	Father	0	Preemptive	0	Grandfather	0	Adherence	0	Clinician	0	Complicated migraine	0	Placebo effect	0	Consultation	0	Irk	0	Beta-Blockers	0	carer	0
Social worker	0	Folate	0	Septoplasty	0	nan	0	Eye pain	0	Angry	0	Emergency care	0	Food allergies	0	Cadasil	0	5HT	0	Anatomy	0	Counselor	0	Phenotype	0	Abnormalities	0	Comorbidity	0	Corticosteroid	0	Mood disorder	0	CT scan	0	Foramen ovale	0	Angiotensin Converting Enzyme	0	Prophylactic treatment	0	Subtype	0	Randomized controlled trial	0	Melatonin	0	Referred pain	0	Comorbidities	0	Behavioral therapy	0	Transmission	0	Father	0	Preemptive	0	Grandfather	0	Adherence	0	Clinician	0	Complicated migraine	0	Placebo effect	0	Consultation	0	Irk	0	Beta-Blockers	0	carer	0
Social worker	0	Folate	0	Septoplasty	0	nan	0	Eye pain	0	Angry	0	Emergency care	0	Food allergies	0	Cadasil	0	5HT	0	Anatomy	0	Counselor	0	Phenotype	0	Abnormalities	0	Comorbidity	0	Corticosteroid	0	Mood disorder	0	CT scan	0	Foramen ovale	0	Angiotensin Converting Enzyme	0	Prophylactic treatment	0	Subtype	0	Randomized controlled trial	0	Melatonin	0	Referred pain	0	Comorbidities	0	Behavioral therapy	0	Transmission	0	Father	0	Preemptive	0	Grandfather	0	Adherence	0	Clinician	0	Complicated migraine	0	Placebo effect	0	Consultation	0	Irk	0	Beta-Blockers	0	carer	0
Social worker	0	Folate	0	Septoplasty	0	nan	0	Eye pain	0	Angry	0	Emergency care	0	Food allergies	0	Cadasil	0	5HT	0	Anatomy	0	Counselor	0	Phenotype	0	Abnormalities	0	Comorbidity	0	Corticosteroid	0	Mood disorder	0	CT scan	0	Foramen ovale	0	Angiotensin Converting Enzyme	0	Prophylactic treatment	0	Subtype	0	Randomized controlled trial	0	Melatonin	0	Referred pain	0	Comorbidities	0	Behavioral therapy	0	Transmission	0	Father	0	Preemptive	0	Grandfather	0	Adherence	0	Clinician	0	Complicated migraine	0	Placebo effect	0	Consultation	0	Irk	0	Beta-Blockers	0	carer	0
Social worker	0	Folate	0	Septoplasty	0	nan	0	Eye pain	0	Angry	0	Emergency care	0	Food allergies	0	Cadasil	0	5HT	0	Anatomy	0	Counselor	0	Phenotype	0	Abnormalities	0	Comorbidity	0	Corticosteroid	0	Mood disorder	0	CT scan	0	Foramen ovale	0	Angiotensin Converting Enzyme	0	Prophylactic treatment	0	Subtype	0	Randomized controlled trial	0	Melatonin	0	Referred pain	0	Comorbidities	0	Behavioral therapy	0	Transmission	0	Father	0	Preemptive	0	Grandfather	0	Adherence	0	Clinician	0	Complicated migraine	0	Placebo effect	0	Consultation	0	Irk	0	Beta-Blockers	0	carer	0
Social worker	0	Folate	0	Septoplasty	0	nan	0	Eye pain	0	Angry	0	Emergency care	0	Food allergies	0	Cadasil	0	5HT	0	Anatomy	0	Counselor	0	Phenotype	0	Abnormalities	0	Comorbidity	0	Corticosteroid	0	Mood disorder	0	CT scan	0	Foramen ovale	0	Angiotensin Converting Enzyme	0	Prophylactic treatment	0	Subtype	0	Randomized controlled trial	0	Melatonin	0	Referred pain	0	Comorbidities	0	Behavioral therapy	0	Transmission	0	Father	0	Preemptive	0	Grandfather	0	Adherence	0	Clinician	0	Complicated migraine	0	Placebo effect	0	Consultation	0	Irk	0	Beta-Blockers	0	carer	0
Social worker	0	Folate	0	Septoplasty	0	nan	0	Eye pain	0	Angry	0	Emergency care	0	Food allergies	0	Cadasil	0	5HT	0	Anatomy	0	Counselor	0	Phenotype	0	Abnormalities	0	Comorbidity	0	Corticosteroid	0	Mood disorder	0	CT scan	0	Foramen ovale	0	Angiotensin Converting Enzyme	0	Prophylactic treatment	0	Subtype	0	Randomized controlled trial	0	Melatonin	0	Referred pain	0	Comorbidities	0	Behavioral therapy	0	Transmission	0	Father	0	Preemptive	0	Grandfather	0	Adherence	0	Clinician	0	Complicated migraine	0	Placebo effect	0	Consultation	0	Irk	0	Beta-Blockers	0	carer	0
Social worker	0	Folate	0	Septoplasty	0	nan	0	Eye pain	0	Angry	0	Emergency care	0	Food allergies	0	Cadasil	0	5HT	0	Anatomy	0	Counselor	0	Phenotype	0	Abnormalities	0	Comorbidity	0	Corticosteroid	0	Mood disorder	0	CT scan	0	Foramen ovale	0	Angiotensin Converting Enzyme	0	Prophylactic treatment	0	Subtype	0	Randomized controlled trial	0	Melatonin	0	Referred pain	0	Comorbidities	0	Behavioral therapy	0	Transmission	0	Father	0	Preemptive	0	Grandfather	0	Adherence	0	Clinician	0	Complicated migraine	0	Placebo effect	0	Consultation	0	Irk	0	Beta-Blockers	0	carer	0
Social worker	0	Folate	0	Septoplasty	0	nan	0	Eye pain	0	Angry	0	Emergency care	0	Food allergies	0	Cadasil	0	5HT	0	Anatomy	0	Counselor	0	Phenotype	0	Abnormalities	0	Comorbidity	0	Corticosteroid	0	Mood disorder	0	CT scan	0	Foramen ovale	0	Angiotensin Converting Enzyme	0	Prophylactic treatment	0	Subtype	0	Randomized controlled trial	0	Melatonin	0	Referred pain	0	Comorbidities	0	Behavioral therapy	0	Transmission	0	Father	0	Preemptive	0	Grandfather	0	Adherence	0	Clinician	0	Complicated migraine	0	Placebo effect	0	Consultation	0	Irk	1	Beta-Blockers	0	carer	0
Social worker	0	Folate	0	Septoplasty	0	nan	0	Eye pain	0	Angry	0	Emergency care	0	Food allergies	0	Cadasil	0	5HT	0	Anatomy	0	Counselor	0	Phenotype	0	Abnormalities	0	Comorbidity	0	Corticosteroid	0	Mood disorder	0	CT scan	0	Foramen ovale	0	Angiotensin Converting Enzyme	0	Prophylactic treatment	0	Subtype	0	Randomized controlled trial	0	Melatonin	0	Referred pain	0	Comorbidities	0	Behavioral therapy	0	Transmission	0	Father	0	Preemptive	0	Grandfather	0	Adherence	0	Clinician	0	Complicated migraine	0	Placebo effect	0	Consultation	0	Irk	0	Beta-Blockers	0	carer	0
Social worker	0	Folate	0	Septoplasty	0	nan	0	Eye pain	0	Angry	0	Emergency care	0	Food allergies	0	Cadasil	0	5HT	0	Anatomy	0	Counselor	0	Phenotype	0	Abnormalities	0	Comorbidity	0	Corticosteroid	0	Mood disorder	0	CT scan	0	Foramen ovale	0	Angiotensin Converting Enzyme	0	Prophylactic treatment	0	Subtype	0	Randomized controlled trial	0	Melatonin	0	Referred pain	0	Comorbidities	0	Behavioral therapy	0	Transmission	0	Father	0	Preemptive	0	Grandfather	0	Adherence	0	Clinician	0	Complicated migraine	0	Placebo effect	0	Consultation	0	Irk	0	Beta-Blockers	0	carer	0
Social worker	0	Folate	0	Septoplasty	1	nan	1	Eye pain	1	Angry	1	Emergency care	1	Food allergies	1	Cadasil	1	5HT	1	Anatomy	0	Counselor	0	Phenotype	0	Abnormalities	0	Comorbidity	0	Corticosteroid	0	Mood disorder	0	CT scan	0	Foramen ovale	0	Angiotensin Converting Enzyme	0	Prophylactic treatment	0	Subtype	0	Randomized controlled trial	0	Melatonin	0	Referred pain	0	Comorbidities	0	Behavioral therapy	0	Transmission	0	Father	0	Preemptive	0	Grandfather	0	Adherence	0	Clinician	0	Complicated migraine	0	Placebo effect	0	Consultation	0	Irk	0	Beta-Blockers	0	carer	0
Social worker	0	Folate	0	Septoplasty	0	nan	0	Eye pain	0	Angry	0	Emergency care	0	Food allergies	0	Cadasil	0	5HT	0	Anatomy	0	Counselor	0	Phenotype	0	Abnormalities	0	Comorbidity	0	Corticosteroid	0	Mood disorder	0	CT scan	0	Foramen ovale	0	Angiotensin Converting Enzyme	0	Prophylactic treatment	0	Subtype	0	Randomized controlled trial	0	Melatonin	0	Referred pain	0	Comorbidities	0	Behavioral therapy	0	Transmission	0	Father	0	Preemptive	0	Grandfather	0	Adherence	0	Clinician	0	Complicated migraine	0	Placebo effect	0	Consultation	0	Irk	0	Beta-Blockers	0	carer	0
Social worker	0	Folate	0	Septoplasty	0	nan	0	Eye pain	0	Angry	0	Emergency care	0	Food allergies	0	Cadasil	0	5HT	0	Anatomy	0	Counselor	0	Phenotype	0	Abnormalities	0	Comorbidity	0	Corticosteroid	0	Mood disorder	0	CT scan	0	Foramen ovale	0	Angiotensin Converting Enzyme	0	Prophylactic treatment	0	Subtype	0	Randomized controlled trial	0	Melatonin	0	Referred pain	0	Comorbidities	0	Behavioral therapy	0	Transmission	0	Father	0	Preemptive	0	Grandfather	0	Adherence	0	Clinician	0	Complicated migraine	0	Placebo effect	0	Consultation	0	Irk	0	Beta-Blockers	0	carer	0
Social worker	0	Folate	0	Septoplasty	0	nan	0	Eye pain	0	Angry	0	Emergency care	0	Food allergies	0	Cadasil	0	5HT	0	Anatomy	0	Counselor	0	Phenotype	0	Abnormalities	0	Comorbidity	0	Corticosteroid	0	Mood disorder	0	CT scan	0	Foramen ovale	0	Angiotensin Converting Enzyme	0	Prophylactic treatment	0	Subtype	0	Randomized controlled trial	0	Melatonin	0	Referred pain	0	Comorbidities	0	Behavioral therapy	0	Transmission	0	Father	0	Preemptive	0	Grandfather	0	Adherence	0	Clinician	0	Complicated migraine	0	Placebo effect	0	Consultation	1	Irk	0	Beta-Blockers	0	carer	0
Social worker	0	Folate	0	Septoplasty	0	nan	0	Eye pain	0	Angry	0	Emergency care	0	Food allergies	0	Cadasil	0	5HT	0	Anatomy	0	Counselor	0	Phenotype	0	Abnormalities	0	Comorbidity	0	Corticosteroid	0	Mood disorder	0	CT scan	0	Foramen ovale	0	Angiotensin Converting Enzyme	0	Prophylactic treatment	0	Subtype	0	Randomized controlled trial	0	Melatonin	0	Referred pain	0	Comorbidities	0	Behavioral therapy	0	Transmission	0	Father	0	Preemptive	0	Grandfather	0	Adherence	0	Clinician	0	Complicated migraine	0	Placebo effect	0	Consultation	0	Irk	0	Beta-Blockers	0	carer	0
Social worker	0	Folate	0	Septoplasty	0	nan	0	Eye pain	0	Angry	0	Emergency care	0	Food allergies	0	Cadasil	0	5HT	0	Anatomy	0	Counselor	0	Phenotype	0	Abnormalities	0	Comorbidity	0	Corticosteroid	0	Mood disorder	0	CT scan	0	Foramen ovale	0	Angiotensin Converting Enzyme	0	Prophylactic treatment	0	Subtype	0	Randomized controlled trial	0	Melatonin	0	Referred pain	0	Comorbidities	0	Behavioral therapy	0	Transmission	0	Father	0	Preemptive	0	Grandfather	0	Adherence	0	Clinician	0	Complicated migraine	0	Placebo effect	0	Consultation	0	Irk	0	Beta-Blockers	0	carer	0
Social worker	0	Folate	0	Septoplasty	0	nan	0	Eye pain	0	Angry	0	Emergency care	0	Food allergies	0	Cadasil	0	5HT	0	Anatomy	0	Counselor	0	Phenotype	0	Abnormalities	0	Comorbidity	0	Corticosteroid	0	Mood disorder	0	CT scan	0	Foramen ovale	0	Angiotensin Converting Enzyme	0	Prophylactic treatment	0	Subtype	0	Randomized controlled trial	0	Melatonin	0	Referred pain	0	Comorbidities	0	Behavioral therapy	0	Transmission	0	Father	0	Preemptive	0	Grandfather	0	Adherence	0	Clinician	0	Complicated migraine	0	Placebo effect	0	Consultation	0	Irk	0	Beta-Blockers	0	carer	0
Social worker	0	Folate	0	Septoplasty	0	nan	0	Eye pain	0	Angry	0	Emergency care	0	Food allergies	0	Cadasil	0	5HT	0	Anatomy	0	Counselor	0	Phenotype	0	Abnormalities	0	Comorbidity	0	Corticosteroid	0	Mood disorder	0	CT scan	0	Foramen ovale	0	Angiotensin Converting Enzyme	0	Prophylactic treatment	0	Subtype	0	Randomized controlled trial	0	Melatonin	0	Referred pain	0	Comorbidities	0	Behavioral therapy	0	Transmission	0	Father	0	Preemptive	0	Grandfather	0	Adherence	0	Clinician	0	Complicated migraine	0	Placebo effect	1	Consultation	0	Irk	0	Beta-Blockers	0	carer	0
Social worker	0	Folate	0	Septoplasty	0	nan	0	Eye pain	0	Angry	0	Emergency care	0	Food allergies	0	Cadasil	0	5HT	0	Anatomy	0	Counselor	0	Phenotype	0	Abnormalities	0	Comorbidity	0	Corticosteroid	0	Mood disorder	0	CT scan	0	Foramen ovale	0	Angiotensin Converting Enzyme	0	Prophylactic treatment	0	Subtype	0	Randomized controlled trial	0	Melatonin	0	Referred pain	0	Comorbidities	0	Behavioral therapy	0	Transmission	0	Father	0	Preemptive	0	Grandfather	0	Adherence	0	Clinician	0	Complicated migraine	0	Placebo effect	0	Consultation	0	Irk	0	Beta-Blockers	0	carer	0
Social worker	1	Folate	1	Septoplasty	0	nan	0	Eye pain	0	Angry	0	Emergency care	0	Food allergies	0	Cadasil	0	5HT	0	Anatomy	0	Counselor	0	Phenotype	0	Abnormalities	0	Comorbidity	0	Corticosteroid	0	Mood disorder	0	CT scan	0	Foramen ovale	0	Angiotensin Converting Enzyme	0	Prophylactic treatment	0	Subtype	0	Randomized controlled trial	0	Melatonin	0	Referred pain	0	Comorbidities	0	Behavioral therapy	0	Transmission	0	Father	0	Preemptive	0	Grandfather	0	Adherence	0	Clinician	0	Complicated migraine	0	Placebo effect	0	Consultation	0	Irk	0	Beta-Blockers	0	carer	0
Social worker	0	Folate	0	Septoplasty	0	nan	0	Eye pain	0	Angry	0	Emergency care	0	Food allergies	0	Cadasil	0	5HT	0	Anatomy	0	Counselor	0	Phenotype	0	Abnormalities	0	Comorbidity	0	Corticosteroid	0	Mood disorder	0	CT scan	0	Foramen ovale	0	Angiotensin Converting Enzyme	0	Prophylactic treatment	0	Subtype	0	Randomized controlled trial	0	Melatonin	0	Referred pain	0	Comorbidities	0	Behavioral therapy	0	Transmission	0	Father	0	Preemptive	0	Grandfather	0	Adherence	0	Clinician	0	Complicated migraine	0	Placebo effect	0	Consultation	0	Irk	0	Beta-Blockers	0	carer	0
Social worker	0	Folate	0	Septoplasty	0	nan	0	Eye pain	0	Angry	0	Emergency care	0	Food allergies	0	Cadasil	0	5HT	0	Anatomy	0	Counselor	0	Phenotype	0	Abnormalities	0	Comorbidity	0	Corticosteroid	0	Mood disorder	0	CT scan	0	Foramen ovale	0	Angiotensin Converting Enzyme	0	Prophylactic treatment	0	Subtype	0	Randomized controlled trial	0	Melatonin	0	Referred pain	0	Comorbidities	0	Behavioral therapy	0	Transmission	0	Father	0	Preemptive	0	Grandfather	0	Adherence	0	Clinician	0	Complicated migraine	0	Placebo effect	0	Consultation	0	Irk	0	Beta-Blockers	0	carer	0
Social worker	0	Folate	0	Septoplasty	0	nan	0	Eye pain	0	Angry	0	Emergency care	0	Food allergies	0	Cadasil	0	5HT	0	Anatomy	0	Counselor	0	Phenotype	0	Abnormalities	0	Comorbidity	0	Corticosteroid	0	Mood disorder	0	CT scan	0	Foramen ovale	0	Angiotensin Converting Enzyme	0	Prophylactic treatment	0	Subtype	0	Randomized controlled trial	0	Melatonin	0	Referred pain	0	Comorbidities	0	Behavioral therapy	0	Transmission	0	Father	0	Preemptive	0	Grandfather	0	Adherence	0	Clinician	0	Complicated migraine	0	Placebo effect	0	Consultation	0	Irk	0	Beta-Blockers	0	carer	0
Social worker	0	Folate	0	Septoplasty	0	nan	0	Eye pain	0	Angry	0	Emergency care	0	Food allergies	0	Cadasil	0	5HT	0	Anatomy	0	Counselor	0	Phenotype	0	Abnormalities	0	Comorbidity	0	Corticosteroid	0	Mood disorder	0	CT scan	0	Foramen ovale	0	Angiotensin Converting Enzyme	0	Prophylactic treatment	0	Subtype	0	Randomized controlled trial	0	Melatonin	0	Referred pain	0	Comorbidities	0	Behavioral therapy	0	Transmission	0	Father	0	Preemptive	0	Grandfather	0	Adherence	0	Clinician	0	Complicated migraine	0	Placebo effect	0	Consultation	0	Irk	0	Beta-Blockers	0	carer	0
Social worker	0	Folate	0	Septoplasty	0	nan	0	Eye pain	0	Angry	0	Emergency care	0	Food allergies	0	Cadasil	0	5HT	0	Anatomy	0	Counselor	0	Phenotype	0	Abnormalities	0	Comorbidity	0	Corticosteroid	0	Mood disorder	0	CT scan	0	Foramen ovale	0	Angiotensin Converting Enzyme	0	Prophylactic treatment	0	Subtype	0	Randomized controlled trial	0	Melatonin	0	Referred pain	0	Comorbidities	0	Behavioral therapy	0	Transmission	0	Father	0	Preemptive	0	Grandfather	0	Adherence	0	Clinician	0	Complicated migraine	0	Placebo effect	0	Consultation	0	Irk	0	Beta-Blockers	0	carer	0
Social worker	0	Folate	0	Septoplasty	0	nan	0	Eye pain	0	Angry	0	Emergency care	0	Food allergies	0	Cadasil	0	5HT	0	Anatomy	0	Counselor	0	Phenotype	0	Abnormalities	0	Comorbidity	0	Corticosteroid	0	Mood disorder	0	CT scan	0	Foramen ovale	0	Angiotensin Converting Enzyme	0	Prophylactic treatment	0	Subtype	0	Randomized controlled trial	0	Melatonin	0	Referred pain	0	Comorbidities	0	Behavioral therapy	0	Transmission	0	Father	0	Preemptive	0	Grandfather	0	Adherence	0	Clinician	0	Complicated migraine	0	Placebo effect	0	Consultation	0	Irk	0	Beta-Blockers	0	carer	0
Social worker	0	Folate	0	Septoplasty	0	nan	0	Eye pain	0	Angry	0	Emergency care	0	Food allergies	0	Cadasil	0	5HT	0	Anatomy	0	Counselor	0	Phenotype	0	Abnormalities	0	Comorbidity	0	Corticosteroid	0	Mood disorder	0	CT scan	0	Foramen ovale	0	Angiotensin Converting Enzyme	0	Prophylactic treatment	0	Subtype	0	Randomized controlled trial	0	Melatonin	0	Referred pain	0	Comorbidities	0	Behavioral therapy	0	Transmission	0	Father	0	Preemptive	0	Grandfather	0	Adherence	0	Clinician	0	Complicated migraine	1	Placebo effect	0	Consultation	0	Irk	0	Beta-Blockers	0	carer	0
Social worker	0	Folate	0	Septoplasty	0	nan	0	Eye pain	0	Angry	0	Emergency care	0	Food allergies	0	Cadasil	0	5HT	0	Anatomy	0	Counselor	0	Phenotype	0	Abnormalities	0	Comorbidity	0	Corticosteroid	0	Mood disorder	0	CT scan	0	Foramen ovale	0	Angiotensin Converting Enzyme	0	Prophylactic treatment	0	Subtype	0	Randomized controlled trial	0	Melatonin	0	Referred pain	0	Comorbidities	0	Behavioral therapy	0	Transmission	0	Father	0	Preemptive	0	Grandfather	0	Adherence	0	Clinician	0	Complicated migraine	0	Placebo effect	0	Consultation	0	Irk	0	Beta-Blockers	0	carer	0
Social worker	0	Folate	0	Septoplasty	0	nan	0	Eye pain	0	Angry	0	Emergency care	0	Food allergies	0	Cadasil	0	5HT	0	Anatomy	0	Counselor	0	Phenotype	0	Abnormalities	0	Comorbidity	0	Corticosteroid	0	Mood disorder	0	CT scan	0	Foramen ovale	0	Angiotensin Converting Enzyme	0	Prophylactic treatment	0	Subtype	0	Randomized controlled trial	0	Melatonin	0	Referred pain	0	Comorbidities	0	Behavioral therapy	0	Transmission	0	Father	0	Preemptive	0	Grandfather	0	Adherence	0	Clinician	0	Complicated migraine	0	Placebo effect	0	Consultation	0	Irk	0	Beta-Blockers	0	carer	0
Social worker	0	Folate	0	Septoplasty	0	nan	0	Eye pain	0	Angry	0	Emergency care	0	Food allergies	0	Cadasil	0	5HT	0	Anatomy	0	Counselor	0	Phenotype	0	Abnormalities	0	Comorbidity	0	Corticosteroid	0	Mood disorder	0	CT scan	0	Foramen ovale	0	Angiotensin Converting Enzyme	0	Prophylactic treatment	0	Subtype	0	Randomized controlled trial	0	Melatonin	0	Referred pain	0	Comorbidities	0	Behavioral therapy	0	Transmission	0	Father	0	Preemptive	0	Grandfather	0	Adherence	0	Clinician	0	Complicated migraine	0	Placebo effect	0	Consultation	0	Irk	0	Beta-Blockers	0	carer	0
Social worker	0	Folate	0	Septoplasty	0	nan	0	Eye pain	0	Angry	0	Emergency care	0	Food allergies	0	Cadasil	0	5HT	0	Anatomy	0	Counselor	0	Phenotype	0	Abnormalities	0	Comorbidity	0	Corticosteroid	0	Mood disorder	0	CT scan	0	Foramen ovale	0	Angiotensin Converting Enzyme	0	Prophylactic treatment	0	Subtype	0	Randomized controlled trial	0	Melatonin	0	Referred pain	0	Comorbidities	0	Behavioral therapy	0	Transmission	0	Father	0	Preemptive	0	Grandfather	0	Adherence	0	Clinician	1	Complicated migraine	0	Placebo effect	0	Consultation	0	Irk	0	Beta-Blockers	0	carer	0
Social worker	0	Folate	0	Septoplasty	0	nan	0	Eye pain	0	Angry	0	Emergency care	0	Food allergies	0	Cadasil	0	5HT	0	Anatomy	0	Counselor	0	Phenotype	0	Abnormalities	0	Comorbidity	0	Corticosteroid	0	Mood disorder	0	CT scan	0	Foramen ovale	0	Angiotensin Converting Enzyme	0	Prophylactic treatment	0	Subtype	0	Randomized controlled trial	0	Melatonin	0	Referred pain	0	Comorbidities	0	Behavioral therapy	0	Transmission	0	Father	0	Preemptive	0	Grandfather	0	Adherence	0	Clinician	0	Complicated migraine	0	Placebo effect	0	Consultation	0	Irk	0	Beta-Blockers	0	carer	0
Social worker	0	Folate	0	Septoplasty	0	nan	0	Eye pain	0	Angry	0	Emergency care	0	Food allergies	0	Cadasil	0	5HT	0	Anatomy	0	Counselor	0	Phenotype	0	Abnormalities	0	Comorbidity	0	Corticosteroid	0	Mood disorder	0	CT scan	0	Foramen ovale	0	Angiotensin Converting Enzyme	0	Prophylactic treatment	0	Subtype	0	Randomized controlled trial	0	Melatonin	0	Referred pain	0	Comorbidities	0	Behavioral therapy	0	Transmission	0	Father	0	Preemptive	0	Grandfather	0	Adherence	0	Clinician	0	Complicated migraine	0	Placebo effect	0	Consultation	0	Irk	0	Beta-Blockers	0	carer	0
Social worker	0	Folate	0	Septoplasty	0	nan	0	Eye pain	0	Angry	0	Emergency care	0	Food allergies	0	Cadasil	0	5HT	0	Anatomy	0	Counselor	0	Phenotype	0	Abnormalities	0	Comorbidity	0	Corticosteroid	0	Mood disorder	0	CT scan	0	Foramen ovale	0	Angiotensin Converting Enzyme	0	Prophylactic treatment	0	Subtype	0	Randomized controlled trial	0	Melatonin	0	Referred pain	0	Comorbidities	0	Behavioral therapy	0	Transmission	0	Father	0	Preemptive	0	Grandfather	0	Adherence	0	Clinician	0	Complicated migraine	0	Placebo effect	0	Consultation	0	Irk	0	Beta-Blockers	0	carer	0
Social worker	0	Folate	0	Septoplasty	0	nan	0	Eye pain	0	Angry	0	Emergency care	0	Food allergies	0	Cadasil	0	5HT	0	Anatomy	0	Counselor	0	Phenotype	0	Abnormalities	0	Comorbidity	0	Corticosteroid	0	Mood disorder	0	CT scan	0	Foramen ovale	0	Angiotensin Converting Enzyme	0	Prophylactic treatment	0	Subtype	0	Randomized controlled trial	0	Melatonin	0	Referred pain	0	Comorbidities	0	Behavioral therapy	0	Transmission	0	Father	1	Preemptive	1	Grandfather	1	Adherence	1	Clinician	0	Complicated migraine	0	Placebo effect	0	Consultation	0	Irk	0	Beta-Blockers	0	carer	0
Social worker	0	Folate	0	Septoplasty	0	nan	0	Eye pain	0	Angry	0	Emergency care	0	Food allergies	0	Cadasil	0	5HT	0	Anatomy	0	Counselor	0	Phenotype	0	Abnormalities	0	Comorbidity	0	Corticosteroid	0	Mood disorder	0	CT scan	0	Foramen ovale	0	Angiotensin Converting Enzyme	0	Prophylactic treatment	0	Subtype	0	Randomized controlled trial	0	Melatonin	0	Referred pain	0	Comorbidities	0	Behavioral therapy	0	Transmission	0	Father	0	Preemptive	0	Grandfather	0	Adherence	0	Clinician	0	Complicated migraine	0	Placebo effect	0	Consultation	0	Irk	0	Beta-Blockers	0	carer	0
Social worker	0	Folate	0	Septoplasty	0	nan	0	Eye pain	0	Angry	0	Emergency care	0	Food allergies	0	Cadasil	0	5HT	0	Anatomy	0	Counselor	0	Phenotype	0	Abnormalities	0	Comorbidity	0	Corticosteroid	0	Mood disorder	0	CT scan	0	Foramen ovale	0	Angiotensin Converting Enzyme	0	Prophylactic treatment	0	Subtype	0	Randomized controlled trial	0	Melatonin	0	Referred pain	0	Comorbidities	0	Behavioral therapy	0	Transmission	0	Father	0	Preemptive	0	Grandfather	0	Adherence	0	Clinician	0	Complicated migraine	0	Placebo effect	0	Consultation	0	Irk	0	Beta-Blockers	0	carer	0
Social worker	0	Folate	0	Septoplasty	0	nan	0	Eye pain	0	Angry	0	Emergency care	0	Food allergies	0	Cadasil	0	5HT	0	Anatomy	0	Counselor	0	Phenotype	0	Abnormalities	0	Comorbidity	0	Corticosteroid	0	Mood disorder	0	CT scan	0	Foramen ovale	0	Angiotensin Converting Enzyme	0	Prophylactic treatment	0	Subtype	0	Randomized controlled trial	0	Melatonin	0	Referred pain	0	Comorbidities	0	Behavioral therapy	0	Transmission	0	Father	0	Preemptive	0	Grandfather	0	Adherence	0	Clinician	0	Complicated migraine	0	Placebo effect	0	Consultation	0	Irk	0	Beta-Blockers	0	carer	0
Social worker	0	Folate	0	Septoplasty	0	nan	0	Eye pain	0	Angry	0	Emergency care	0	Food allergies	0	Cadasil	0	5HT	0	Anatomy	0	Counselor	0	Phenotype	0	Abnormalities	0	Comorbidity	0	Corticosteroid	0	Mood disorder	0	CT scan	0	Foramen ovale	0	Angiotensin Converting Enzyme	0	Prophylactic treatment	0	Subtype	0	Randomized controlled trial	0	Melatonin	0	Referred pain	0	Comorbidities	0	Behavioral therapy	0	Transmission	0	Father	0	Preemptive	0	Grandfather	0	Adherence	0	Clinician	0	Complicated migraine	0	Placebo effect	0	Consultation	0	Irk	0	Beta-Blockers	0	carer	0
Social worker	0	Folate	0	Septoplasty	0	nan	0	Eye pain	0	Angry	0	Emergency care	0	Food allergies	0	Cadasil	0	5HT	0	Anatomy	0	Counselor	0	Phenotype	0	Abnormalities	0	Comorbidity	0	Corticosteroid	0	Mood disorder	0	CT scan	0	Foramen ovale	0	Angiotensin Converting Enzyme	0	Prophylactic treatment	0	Subtype	0	Randomized controlled trial	0	Melatonin	0	Referred pain	0	Comorbidities	1	Behavioral therapy	1	Transmission	1	Father	0	Preemptive	0	Grandfather	0	Adherence	0	Clinician	0	Complicated migraine	0	Placebo effect	0	Consultation	0	Irk	0	Beta-Blockers	0	carer	0
Social worker	0	Folate	0	Septoplasty	0	nan	0	Eye pain	0	Angry	0	Emergency care	0	Food allergies	0	Cadasil	0	5HT	0	Anatomy	0	Counselor	0	Phenotype	0	Abnormalities	0	Comorbidity	0	Corticosteroid	0	Mood disorder	0	CT scan	0	Foramen ovale	0	Angiotensin Converting Enzyme	0	Prophylactic treatment	0	Subtype	0	Randomized controlled trial	0	Melatonin	0	Referred pain	1	Comorbidities	0	Behavioral therapy	0	Transmission	0	Father	0	Preemptive	0	Grandfather	0	Adherence	0	Clinician	0	Complicated migraine	0	Placebo effect	0	Consultation	0	Irk	0	Beta-Blockers	0	carer	0
Social worker	0	Folate	0	Septoplasty	0	nan	0	Eye pain	0	Angry	0	Emergency care	0	Food allergies	0	Cadasil	0	5HT	0	Anatomy	0	Counselor	0	Phenotype	0	Abnormalities	0	Comorbidity	0	Corticosteroid	0	Mood disorder	0	CT scan	0	Foramen ovale	0	Angiotensin Converting Enzyme	0	Prophylactic treatment	0	Subtype	0	Randomized controlled trial	0	Melatonin	0	Referred pain	0	Comorbidities	0	Behavioral therapy	0	Transmission	0	Father	0	Preemptive	0	Grandfather	0	Adherence	0	Clinician	0	Complicated migraine	0	Placebo effect	0	Consultation	0	Irk	0	Beta-Blockers	0	carer	0
Social worker	0	Folate	0	Septoplasty	0	nan	0	Eye pain	0	Angry	0	Emergency care	0	Food allergies	0	Cadasil	0	5HT	0	Anatomy	0	Counselor	0	Phenotype	0	Abnormalities	0	Comorbidity	0	Corticosteroid	0	Mood disorder	0	CT scan	0	Foramen ovale	0	Angiotensin Converting Enzyme	0	Prophylactic treatment	0	Subtype	0	Randomized controlled trial	0	Melatonin	0	Referred pain	0	Comorbidities	0	Behavioral therapy	0	Transmission	0	Father	0	Preemptive	0	Grandfather	0	Adherence	0	Clinician	0	Complicated migraine	0	Placebo effect	0	Consultation	0	Irk	0	Beta-Blockers	0	carer	0
Social worker	0	Folate	0	Septoplasty	0	nan	0	Eye pain	0	Angry	0	Emergency care	0	Food allergies	0	Cadasil	0	5HT	0	Anatomy	0	Counselor	0	Phenotype	0	Abnormalities	0	Comorbidity	0	Corticosteroid	0	Mood disorder	0	CT scan	0	Foramen ovale	0	Angiotensin Converting Enzyme	0	Prophylactic treatment	0	Subtype	0	Randomized controlled trial	0	Melatonin	0	Referred pain	0	Comorbidities	0	Behavioral therapy	0	Transmission	0	Father	0	Preemptive	0	Grandfather	0	Adherence	0	Clinician	0	Complicated migraine	0	Placebo effect	0	Consultation	0	Irk	0	Beta-Blockers	0	carer	0
Social worker	0	Folate	0	Septoplasty	0	nan	0	Eye pain	0	Angry	0	Emergency care	0	Food allergies	0	Cadasil	0	5HT	0	Anatomy	0	Counselor	0	Phenotype	0	Abnormalities	0	Comorbidity	0	Corticosteroid	0	Mood disorder	0	CT scan	0	Foramen ovale	0	Angiotensin Converting Enzyme	0	Prophylactic treatment	0	Subtype	0	Randomized controlled trial	0	Melatonin	0	Referred pain	0	Comorbidities	0	Behavioral therapy	0	Transmission	0	Father	0	Preemptive	0	Grandfather	0	Adherence	0	Clinician	0	Complicated migraine	0	Placebo effect	0	Consultation	0	Irk	0	Beta-Blockers	0	carer	0
Social worker	0	Folate	0	Septoplasty	0	nan	0	Eye pain	0	Angry	0	Emergency care	0	Food allergies	0	Cadasil	0	5HT	0	Anatomy	0	Counselor	0	Phenotype	0	Abnormalities	0	Comorbidity	0	Corticosteroid	0	Mood disorder	0	CT scan	0	Foramen ovale	0	Angiotensin Converting Enzyme	0	Prophylactic treatment	0	Subtype	0	Randomized controlled trial	0	Melatonin	0	Referred pain	0	Comorbidities	0	Behavioral therapy	0	Transmission	0	Father	0	Preemptive	0	Grandfather	0	Adherence	0	Clinician	0	Complicated migraine	0	Placebo effect	0	Consultation	0	Irk	0	Beta-Blockers	0	carer	0
Social worker	0	Folate	0	Septoplasty	0	nan	0	Eye pain	0	Angry	0	Emergency care	0	Food allergies	0	Cadasil	0	5HT	0	Anatomy	0	Counselor	0	Phenotype	0	Abnormalities	0	Comorbidity	0	Corticosteroid	0	Mood disorder	0	CT scan	0	Foramen ovale	0	Angiotensin Converting Enzyme	0	Prophylactic treatment	0	Subtype	0	Randomized controlled trial	0	Melatonin	0	Referred pain	0	Comorbidities	0	Behavioral therapy	0	Transmission	0	Father	0	Preemptive	0	Grandfather	0	Adherence	0	Clinician	0	Complicated migraine	0	Placebo effect	0	Consultation	0	Irk	0	Beta-Blockers	0	carer	0
Social worker	0	Folate	0	Septoplasty	0	nan	0	Eye pain	0	Angry	0	Emergency care	0	Food allergies	0	Cadasil	0	5HT	0	Anatomy	0	Counselor	0	Phenotype	0	Abnormalities	0	Comorbidity	0	Corticosteroid	0	Mood disorder	0	CT scan	0	Foramen ovale	0	Angiotensin Converting Enzyme	0	Prophylactic treatment	0	Subtype	0	Randomized controlled trial	0	Melatonin	0	Referred pain	0	Comorbidities	0	Behavioral therapy	0	Transmission	0	Father	0	Preemptive	0	Grandfather	0	Adherence	0	Clinician	0	Complicated migraine	0	Placebo effect	0	Consultation	0	Irk	0	Beta-Blockers	0	carer	0
Social worker	0	Folate	0	Septoplasty	0	nan	0	Eye pain	0	Angry	0	Emergency care	0	Food allergies	0	Cadasil	0	5HT	0	Anatomy	0	Counselor	0	Phenotype	0	Abnormalities	0	Comorbidity	0	Corticosteroid	0	Mood disorder	0	CT scan	0	Foramen ovale	1	Angiotensin Converting Enzyme	1	Prophylactic treatment	1	Subtype	1	Randomized controlled trial	1	Melatonin	1	Referred pain	0	Comorbidities	0	Behavioral therapy	0	Transmission	0	Father	0	Preemptive	0	Grandfather	0	Adherence	0	Clinician	0	Complicated migraine	0	Placebo effect	0	Consultation	0	Irk	0	Beta-Blockers	0	carer	0
Social worker	0	Folate	0	Septoplasty	0	nan	0	Eye pain	0	Angry	0	Emergency care	0	Food allergies	0	Cadasil	0	5HT	0	Anatomy	0	Counselor	0	Phenotype	0	Abnormalities	0	Comorbidity	0	Corticosteroid	0	Mood disorder	0	CT scan	1	Foramen ovale	0	Angiotensin Converting Enzyme	0	Prophylactic treatment	0	Subtype	0	Randomized controlled trial	0	Melatonin	0	Referred pain	0	Comorbidities	0	Behavioral therapy	0	Transmission	0	Father	0	Preemptive	0	Grandfather	0	Adherence	0	Clinician	0	Complicated migraine	0	Placebo effect	0	Consultation	0	Irk	0	Beta-Blockers	0	carer	0
Social worker	0	Folate	0	Septoplasty	0	nan	0	Eye pain	0	Angry	0	Emergency care	0	Food allergies	0	Cadasil	0	5HT	0	Anatomy	0	Counselor	1	Phenotype	1	Abnormalities	1	Comorbidity	1	Corticosteroid	1	Mood disorder	1	CT scan	0	Foramen ovale	0	Angiotensin Converting Enzyme	0	Prophylactic treatment	0	Subtype	0	Randomized controlled trial	0	Melatonin	0	Referred pain	0	Comorbidities	0	Behavioral therapy	0	Transmission	0	Father	0	Preemptive	0	Grandfather	0	Adherence	0	Clinician	0	Complicated migraine	0	Placebo effect	0	Consultation	0	Irk	0	Beta-Blockers	0	carer	0
Social worker	0	Folate	0	Septoplasty	0	nan	0	Eye pain	0	Angry	0	Emergency care	0	Food allergies	0	Cadasil	0	5HT	0	Anatomy	0	Counselor	0	Phenotype	0	Abnormalities	0	Comorbidity	0	Corticosteroid	0	Mood disorder	0	CT scan	0	Foramen ovale	0	Angiotensin Converting Enzyme	0	Prophylactic treatment	0	Subtype	0	Randomized controlled trial	0	Melatonin	0	Referred pain	0	Comorbidities	0	Behavioral therapy	0	Transmission	0	Father	0	Preemptive	0	Grandfather	0	Adherence	0	Clinician	0	Complicated migraine	0	Placebo effect	0	Consultation	0	Irk	0	Beta-Blockers	0	carer	0
Social worker	0	Folate	0	Septoplasty	0	nan	0	Eye pain	0	Angry	0	Emergency care	0	Food allergies	0	Cadasil	0	5HT	0	Anatomy	0	Counselor	0	Phenotype	0	Abnormalities	0	Comorbidity	0	Corticosteroid	0	Mood disorder	0	CT scan	0	Foramen ovale	0	Angiotensin Converting Enzyme	0	Prophylactic treatment	0	Subtype	0	Randomized controlled trial	0	Melatonin	0	Referred pain	0	Comorbidities	0	Behavioral therapy	0	Transmission	0	Father	0	Preemptive	0	Grandfather	0	Adherence	0	Clinician	0	Complicated migraine	0	Placebo effect	0	Consultation	0	Irk	0	Beta-Blockers	0	carer	0
Social worker	0	Folate	0	Septoplasty	0	nan	0	Eye pain	0	Angry	0	Emergency care	0	Food allergies	0	Cadasil	0	5HT	0	Anatomy	0	Counselor	0	Phenotype	0	Abnormalities	0	Comorbidity	0	Corticosteroid	0	Mood disorder	0	CT scan	0	Foramen ovale	0	Angiotensin Converting Enzyme	0	Prophylactic treatment	0	Subtype	0	Randomized controlled trial	0	Melatonin	0	Referred pain	0	Comorbidities	0	Behavioral therapy	0	Transmission	0	Father	0	Preemptive	0	Grandfather	0	Adherence	0	Clinician	0	Complicated migraine	0	Placebo effect	0	Consultation	0	Irk	0	Beta-Blockers	0	carer	0
Social worker	0	Folate	0	Septoplasty	0	nan	0	Eye pain	0	Angry	0	Emergency care	0	Food allergies	0	Cadasil	0	5HT	0	Anatomy	0	Counselor	0	Phenotype	0	Abnormalities	0	Comorbidity	0	Corticosteroid	0	Mood disorder	0	CT scan	0	Foramen ovale	0	Angiotensin Converting Enzyme	0	Prophylactic treatment	0	Subtype	0	Randomized controlled trial	0	Melatonin	0	Referred pain	0	Comorbidities	0	Behavioral therapy	0	Transmission	0	Father	0	Preemptive	0	Grandfather	0	Adherence	0	Clinician	0	Complicated migraine	0	Placebo effect	0	Consultation	0	Irk	0	Beta-Blockers	0	carer	0
Social worker	0	Folate	0	Septoplasty	0	nan	0	Eye pain	0	Angry	0	Emergency care	0	Food allergies	0	Cadasil	0	5HT	0	Anatomy	0	Counselor	0	Phenotype	0	Abnormalities	0	Comorbidity	0	Corticosteroid	0	Mood disorder	0	CT scan	0	Foramen ovale	0	Angiotensin Converting Enzyme	0	Prophylactic treatment	0	Subtype	0	Randomized controlled trial	0	Melatonin	0	Referred pain	0	Comorbidities	0	Behavioral therapy	0	Transmission	0	Father	0	Preemptive	0	Grandfather	0	Adherence	0	Clinician	0	Complicated migraine	0	Placebo effect	0	Consultation	0	Irk	0	Beta-Blockers	0	carer	0
Social worker	0	Folate	0	Septoplasty	0	nan	0	Eye pain	0	Angry	0	Emergency care	0	Food allergies	0	Cadasil	0	5HT	0	Anatomy	1	Counselor	0	Phenotype	0	Abnormalities	0	Comorbidity	0	Corticosteroid	0	Mood disorder	0	CT scan	0	Foramen ovale	0	Angiotensin Converting Enzyme	0	Prophylactic treatment	0	Subtype	0	Randomized controlled trial	0	Melatonin	0	Referred pain	0	Comorbidities	0	Behavioral therapy	0	Transmission	0	Father	0	Preemptive	0	Grandfather	0	Adherence	0	Clinician	0	Complicated migraine	0	Placebo effect	0	Consultation	0	Irk	0	Beta-Blockers	0	carer	0
Social worker	0	Folate	0	Septoplasty	0	nan	0	Eye pain	0	Angry	0	Emergency care	0	Food allergies	0	Cadasil	0	5HT	0	Anatomy	0	Counselor	0	Phenotype	0	Abnormalities	0	Comorbidity	0	Corticosteroid	0	Mood disorder	0	CT scan	0	Foramen ovale	0	Angiotensin Converting Enzyme	0	Prophylactic treatment	0	Subtype	0	Randomized controlled trial	0	Melatonin	0	Referred pain	0	Comorbidities	0	Behavioral therapy	0	Transmission	0	Father	0	Preemptive	0	Grandfather	0	Adherence	0	Clinician	0	Complicated migraine	0	Placebo effect	0	Consultation	0	Irk	0	Beta-Blockers	0	carer	0
Social worker	0	Folate	0	Septoplasty	0	nan	0	Eye pain	0	Angry	0	Emergency care	0	Food allergies	0	Cadasil	0	5HT	0	Anatomy	0	Counselor	0	Phenotype	0	Abnormalities	0	Comorbidity	0	Corticosteroid	0	Mood disorder	0	CT scan	0	Foramen ovale	0	Angiotensin Converting Enzyme	0	Prophylactic treatment	0	Subtype	0	Randomized controlled trial	0	Melatonin	0	Referred pain	0	Comorbidities	0	Behavioral therapy	0	Transmission	0	Father	0	Preemptive	0	Grandfather	0	Adherence	0	Clinician	0	Complicated migraine	0	Placebo effect	0	Consultation	0	Irk	0	Beta-Blockers	0	carer	0
Social worker	0	Folate	0	Septoplasty	0	nan	0	Eye pain	0	Angry	0	Emergency care	0	Food allergies	0	Cadasil	0	5HT	0	Anatomy	0	Counselor	0	Phenotype	0	Abnormalities	0	Comorbidity	0	Corticosteroid	0	Mood disorder	0	CT scan	0	Foramen ovale	0	Angiotensin Converting Enzyme	0	Prophylactic treatment	0	Subtype	0	Randomized controlled trial	0	Melatonin	0	Referred pain	0	Comorbidities	0	Behavioral therapy	0	Transmission	0	Father	0	Preemptive	0	Grandfather	0	Adherence	0	Clinician	0	Complicated migraine	0	Placebo effect	0	Consultation	0	Irk	0	Beta-Blockers	0	carer	0
Social worker	0	Folate	0	Septoplasty	0	nan	0	Eye pain	0	Angry	0	Emergency care	0	Food allergies	0	Cadasil	0	5HT	0	Anatomy	0	Counselor	0	Phenotype	0	Abnormalities	0	Comorbidity	0	Corticosteroid	0	Mood disorder	0	CT scan	0	Foramen ovale	0	Angiotensin Converting Enzyme	0	Prophylactic treatment	0	Subtype	0	Randomized controlled trial	0	Melatonin	0	Referred pain	0	Comorbidities	0	Behavioral therapy	0	Transmission	0	Father	0	Preemptive	0	Grandfather	0	Adherence	0	Clinician	0	Complicated migraine	0	Placebo effect	0	Consultation	0	Irk	0	Beta-Blockers	0	carer	0
Social worker	0	Folate	0	Septoplasty	0	nan	0	Eye pain	0	Angry	0	Emergency care	0	Food allergies	0	Cadasil	0	5HT	0	Anatomy	0	Counselor	0	Phenotype	0	Abnormalities	0	Comorbidity	0	Corticosteroid	0	Mood disorder	0	CT scan	0	Foramen ovale	0	Angiotensin Converting Enzyme	0	Prophylactic treatment	0	Subtype	0	Randomized controlled trial	0	Melatonin	0	Referred pain	0	Comorbidities	0	Behavioral therapy	0	Transmission	0	Father	0	Preemptive	0	Grandfather	0	Adherence	0	Clinician	0	Complicated migraine	0	Placebo effect	0	Consultation	0	Irk	0	Beta-Blockers	0	carer	0
	2		2		2		2		2		2		2		2		2		2		1		1		1		1		1		1		1		1		1		1		1		1		1		1		1		1		1		1		1		1		1		1		1		1		1		1		1		1		1
